# Supplementary material for: Enantioselective synthesis of chiral α,α-dialkyl indoles and related azoles by cobalt-catalyzed hydroalkylation and regioselectivity switch
Source: Nat Commun. 2024 May 6;15:3783. doi: 10.1038/s41467-024-48175-7 (PMC11074313; doi:10.1038/s41467-024-48175-7)
Supplement: Supplementary file 1 — Supplementary Information [file 41467_2024_48175_MOESM1_ESM.pdf]

Supplementary Information

Enantioselective Synthesis of Chiral *α,α*-Dialkyl Indoles and Related Azoles by Cobalt-Catalyzed Hydroalkylation and Regioselectivity Switch

Jiangtao Ren[1,2,5], Zheng Sun[1,5], Shuang Zhao[1,3], Jinyuan Huang[1,3], Yukun Wang[1], Cheng Zhang[1,3], Jinhai Huang[1], Chenhao Zhang[1], Ruipu Zhang[1,3], Zhihan Zhang*[4], Xu Ji*[1,3] & Zhihui Shao*[1,2]

[1] Key Laboratory of Medicinal Chemistry for Natural Resource, Ministry of Education, School of Chemical Science and Technology, and State Key Laboratory for Conservation and Utilization of Bio-Resources in Yunnan, Yunnan University, Kunming 650500, China. E-mail: zhihui_shao@hotmail.com

[2] Southwest United Graduate School, Kunming 650092, China.

[3] School of Pharmacy, Yunnan University, Kunming 650500, China. E-mail: jixu@ynu.edu.cn

[4] College of Chemistry, Central China Normal University, Wuhan 430079, China. E-mail:_ zhihanzhang@ccnu.edu.cn

[5] These authors contributed equally: Jiangtao Ren, Zheng Sun

Table of Contents

1. General Information 1

2. Optimization of the reaction Conditions 2

3. Synthesis *N*-Alkenyl Indoles 8

4. Preparation of Alkyl Halides 18

5. Single crystal X-ray diffraction data 24

6. Co-Catalyzed Synthesis of *N*-Alkylated Indoles 28

7. Synthetic transformations 68

8. Drug Activity Research 76

9. Mechanistic Study 78

10. Computational details 86

11. NMR and HPLC Spectra 89

12. References 224

1. General Information

All reactions were set up in a 10 mL Telfon-screw capped test tubes (unless otherwise noted) under an inert Argon (Ar) atmosphere. Solvents were purified under Ar using a solvent purification system.

Analytical thin layer chromatography (TLC) was performed using silica gel plates. Visualisation was by ultraviolet fluorescence, and/or phosphomolybdic acid, and/or $KMnO_4$.

Flash column chromatography (FC) was performed using *Qingdao* (200-300 mesh) silica gel.

NMR: ^{1}H NMR, ^{13}C NMR, ^{19}F NMR spectra were recorded on Bruker Avance 400 MHz, 500 MHz, 600 MHz Spectrometer. ^{1}H and ^{13}C chemical shifts were referenced internally to residual solvent peaks relative to TMS (δ = 0 ppm) at 299 K. Chemical shifts (δ (ppm)) are reported relative to TMS (δ (^{1}H) 0.0 ppm, δ (^{13}C) 0.0 ppm). The solvents' residual proton resonance and the respective carbon resonance (for $CHCl_3$; δ (^{1}H) 7.26 ppm, δ (^{13}C) 77.0 ppm were used for calibration.)

HPLC spectra were recorded on an *Agilent* HPLC. Column, eluent and retention times for HPLC analysis used for the determination of enantiomeric ratios are given below in the details of the relevant experiments.

Optical rotations were measured on a *JASCO* DIP-370 polarimeter.

High-resolution mass spectra (HRMS) was recorded on a VG Auto Spec-3000 spectrometer.

All reagents were either prepared according to known literatures or purchased from *Energy-chemical, TCI*, *Bide-pharmatech* and *Tansoole*.

2. Optimization of the reaction Conditions

General Procedure : To an oven-dried 10.0 mL Teflon-screw cap test tube containing a magnetic stir was charged with a **Co-salt** (10 mol%) and the corresponding ligand (15 mol%) under an inert Argon atmosphere using glove-box techniques.Subsequently, anhudrous solvent (0.5 mL) was added, and the mixture was stirred for 10 minutes at room temperature. Then Base (2.0 equiv), **1a** (15.7 mg, 0.10 mmol, 1.0 equiv), (3-iodopropyl)benzene **2a** (49.2 mg, 0.20 mmol, 2.0 equiv), $(OEt)_2MeSiH$ (32.0 uL, 0.20 mmol, 2.0 equiv) were sequentially added. Afterwards, the tube was sealed with airtight electrical tapes and removed from the glove box and stirred at the corresponding temperature for 24 hours at 500 rpm unless otherwise noted. After the reaction was completed, the reaction mixture was diluted with saturated NH_4Cl (aq., 0.5 mL) and EtOAc (3.0 mL). Then, the aqueous phase was extracted with EtOAc (2 × 3.0 mL). The combined organic phases were dried over Na_2SO_4, and the volatiles were removed to afford the crude product. The crude mixture was purified by flash column chromatography on silica gel using a mixture of PE/EtOAc as eluent to obtain the desired product.

Supplementary Table 1. Screening of reaction parameters for the synthesis of *N*-Alkylated Indoles[a]

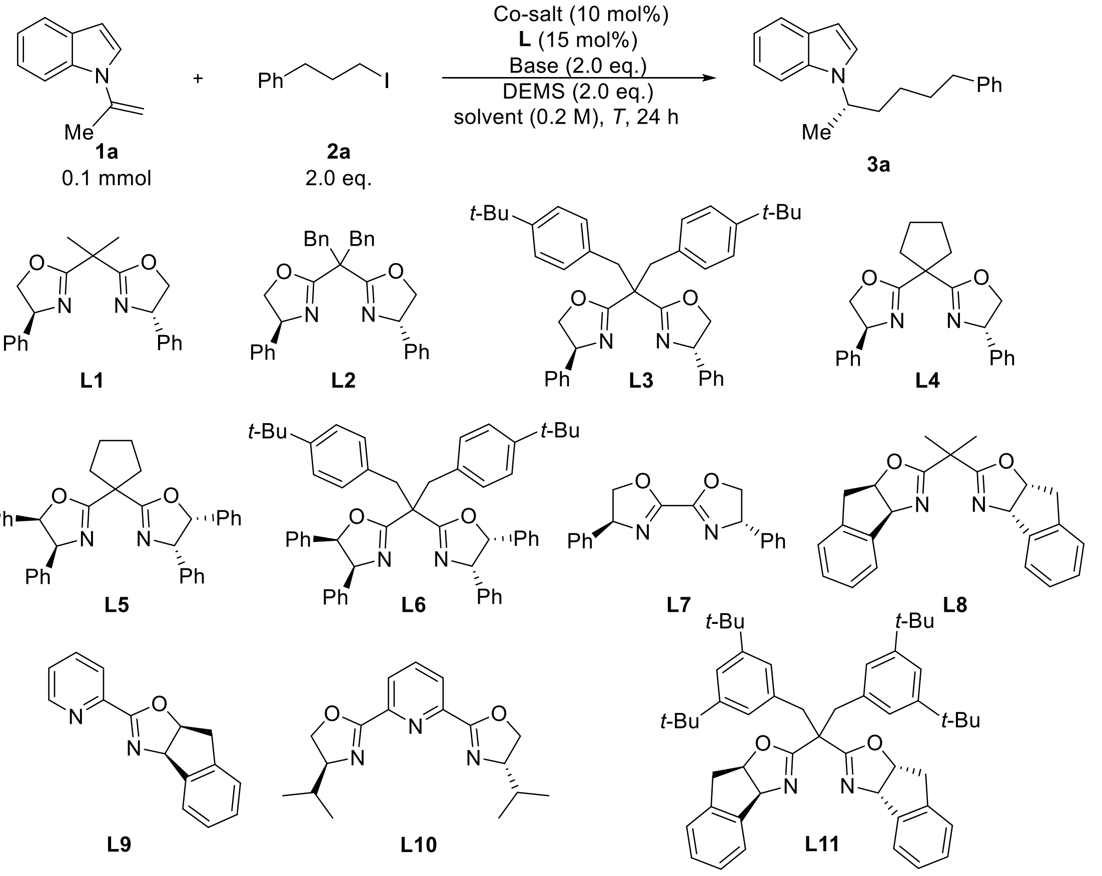

Entry	Co-salt	L	Base	Solvent	*T*	Yield (%)[b]	er[c]
1	Co(acac)$_2$	**L6**	CsF	DME	0 °C	trace	N.D.
2	Co(OAc)$_2$	**L6**	CsF	DME	0 °C	trace	N.D.
3	CoCl$_2$(PPh$_3$)$_2$	**L6**	CsF	DME	0 °C	65	69:31
4	CoI$_2$	**L6**	CsF	DME	0 °C	90	95:5
5	NiBr$_2$•DME	**L6**	CsF	DME	0 °C	9	69:31
6	NiBr$_2$•DME	**L6**	CsF	DMA	0 °C	trace	N.D.
7	NiBr$_2$•DME	**L6**	CsF	DMF	0 °C	trace	N.D.
5	NiBr$_2$•DME	**L6**	CsF	THF	0 °C	trace	N.D.
6	CoBr$_2$•DME	**L6**	CsF	DME	0 °C	98	95:5
7	CoBr$_2$•DME	**L1**	CsF	DME	0 °C	88	93:7
8	CoBr$_2$•DME	**L2**	CsF	DME	0 °C	82	93.5:6.5

9	$CoBr_2$•DME	**L3**	CsF	DME	0 °C	68	93:7
10	$CoBr_2$•DME	**L4**	CsF	DME	0 °C	81	93.5:6.5
11	$CoBr_2$•DME	**L5**	CsF	DME	0 °C	66	95:5
12	$CoBr_2$•DME	**L7**	CsF	DME	0 °C	trace	N.D.
13	$CoBr_2$•DME	**L8**	CsF	DME	0 °C	68	94:6
14	$CoBr_2$•DME	**L9**	CsF	DME	0 °C	trace	N.D.
15	$CoBr_2$•DME	**L10**	CsF	DME	0 °C	trace	N.D.
16	$CoBr_2$•DME	**L11**	CsF	DME	0 °C	87	80:20
17	$CoBr_2$•DME	**L6**	KF	DME	0 °C	6	94.5:5.5
18	$CoBr_2$•DME	**L6**	K_2CO_3	DME	0 °C	57	94:6
19	$CoBr_2$•DME	**L6**	LiO*t*Bu	DME	0 °C	35	90:10
20	$CoBr_2$•DME	**L6**	NaI	DME	0 °C	trace	N.D.
21	$CoBr_2$•DME	**L6**	Na_2CO_3	DME	0 °C	0	N.D.
22	$CoBr_2$•DME	**L6**	K_3PO_4	DME	0 °C	9	94.5:5.5
23	$CoBr_2$•DME	**L6**	CsF	EtOH	0 °C	0	N.D.
24	$CoBr_2$•DME	**L6**	CsF	DCM	0 °C	0	N.D.
25	$CoBr_2$•DME	**L6**	CsF	THF	0 °C	75	95:5
26	$CoBr_2$•DME	**L6**	CsF	DMA	0 °C	48	57:43
27	$CoBr_2$•DME	**L6**	CsF	MeCN	0 °C	trace	N.D.
28	$CoBr_2$•DME	**L6**	CsF	DME	rt	54	94.5:5.5
29	$CoBr_2$•DME	**L6**	CsF	DME	40 °C	66	95:5

[a] Conditions: All reactions were carried out with Co-salt (10 mol%), Ligand **L** (15 mol%), **1a** (0.10 mmol, 1.0 equiv), **2a** (0.20 mmol, 2.0 equiv), $(OEt)_2MeSiH$ (0.20 mmol), Base (0.20 mmol) and solvent (0.5 mL) at the corresponding temperature (*T*) for 24 hours. [b] Isolated yields. [c] er was determined by HPLC.

Supplementary Table 2. Screening of ligands[a]

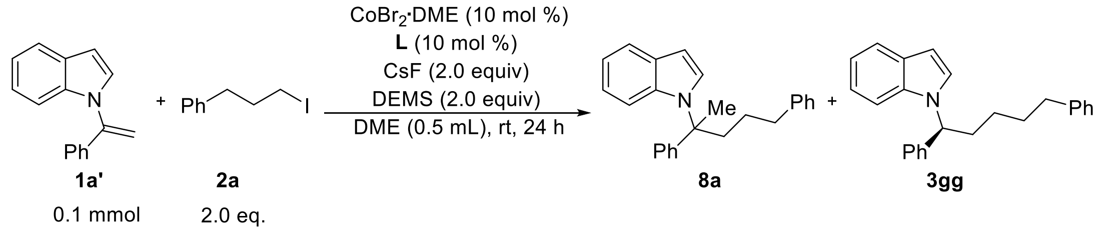

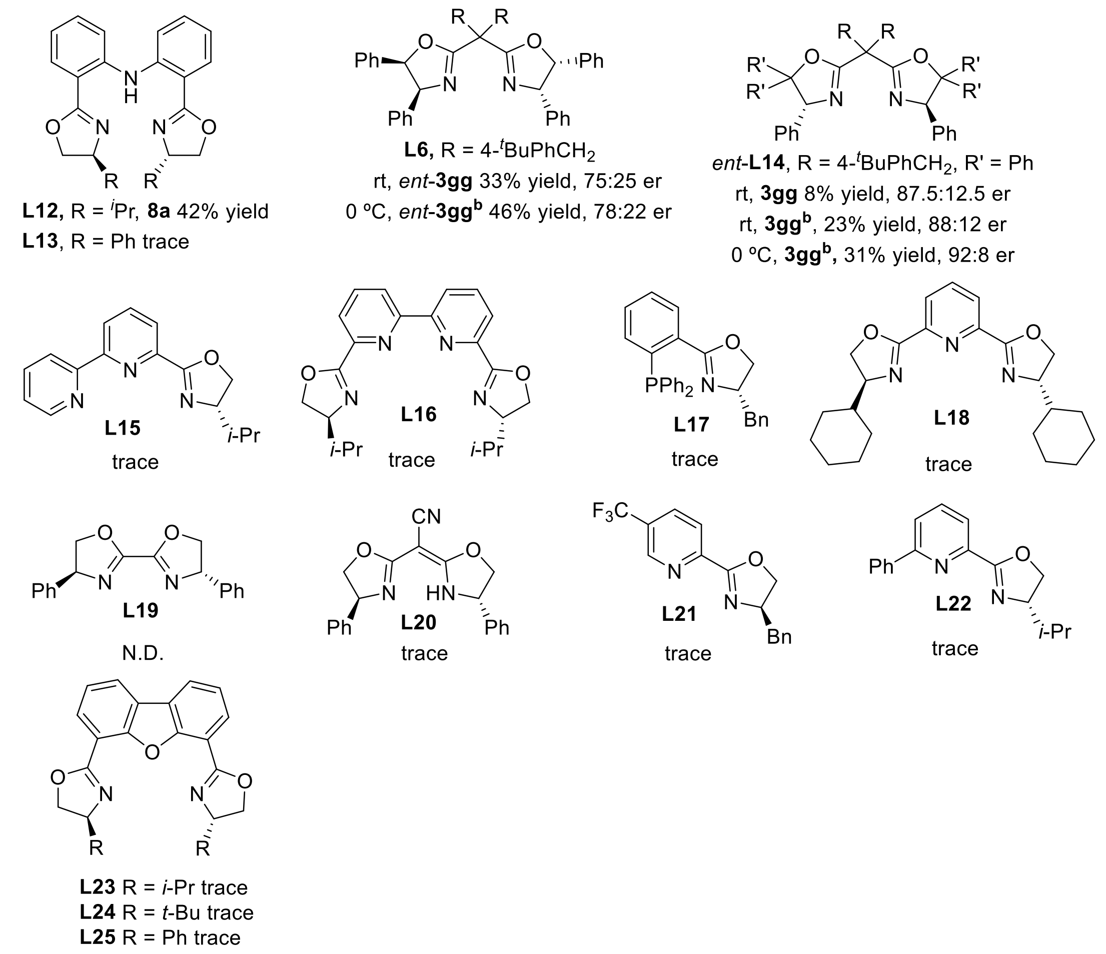

[a] Conditions: All reactions were carried out with $CoBr_2$•DME (10 mol%), Ligand **L** (10 mol%), **1a'** (0.10 mmol, 1.0 equiv), **2a** (0.20 mmol, 2.0 equiv), $(OEt)_2MeSiH$ (0.20 mmol), CsF (0.20 mmol) and DME (0.5 mL) at the rt for 24 hours. Isolated yields. er was determined by HPLC. [b] Ligand **L** (15 mol%).

Supplementary Table 3. Screening of [H] source and Solvents[a]

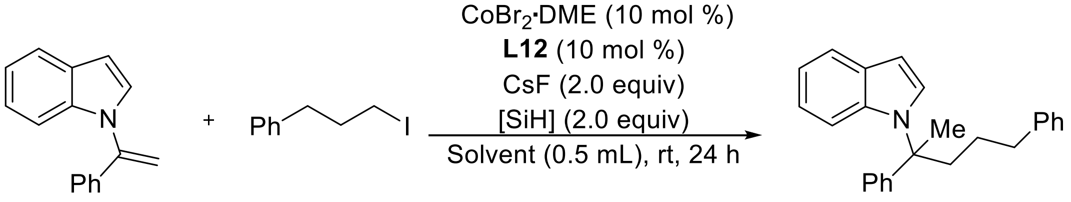

1a' 0.1 mmol + **2a** 2.0 eq. → **8a**

Entry	Solvent	[SiH]	Yield (%)[b]
1	DME	DEMS	42
2	MeOH	DEMS	N.D.
3	MeCN	DEMS	trace
4	DCM	DEMS	trace
5	THF	DEMS	44
6	THF	Ph_2SiH_2	53
7	THF	DMMS	trace
8	THF	$Ph(Me)_2SiH$	trace
9	THF	Et_2SiH_2	N.D.
10	THF	(i-Pr)$_3$SiH	trace
11	THF	$PhMeSiH_2$	48
12	THF	$(EtO)_3SiH$	trace

[a] Conditions: All reactions were carried out with CoBr2•DME (10 mol%), Ligand **L12** (10 mol%), **1a'** (0.10 mmol, 1.0 equiv), **2a** (0.20 mmol, 2.0 equiv), **[SiH]** (0.20 mmol), CsF (0.20 mmol) and **Solvent** (0.5 mL) at the corresponding temperature (T) for 24 hours. [b] Isolated yields.

Supplementary Table 4. Screening of reaction parameters for the synthesis of *N*-Alkyl indoles[a]

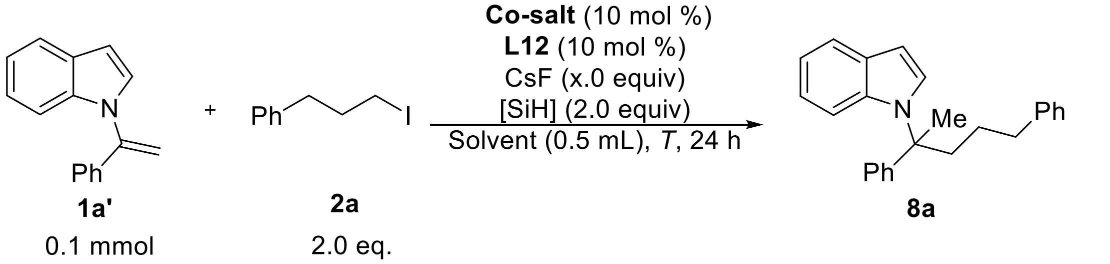

Entry	Co-salt	CsF (eq.)	[SiH]	Slovent	*T*	Yield (%)[b]
1	$CoBr_2$•DME	2.0	Ph_2SiH_2	THF	rt	53
2	$CoBr_2$•DME	3.0	Ph_2SiH_2	THF	rt	55
3	$CoBr_2$•DME	4.0	Ph_2SiH_2	THF	rt	48
4	$CoBr_2$•DME	3.0	Ph_2SiH_2	THF	0 °C	N.D.
5	$CoBr_2$•DME	3.0	Ph_2SiH_2	THF	15 °C	trace
6	$CoBr_2$•DME	3.0	Ph_2SiH_2	THF	40 °C	64
7	$CoBr_2$•DME	3.0	Ph_2SiH_2	THF	50 °C	60
8	$CoBr_2$•DME	3.0	Ph_2SiH_2	THF	60 °C	53
9	$CoBr_2$•DME	3.0	Ph_2SiH_2	THF	70 °C	37
10	$CoBr_2$•DME	3.0	Ph_2SiH_2	1,4-dioxane	40 °C	58
11	$CoBr_2$•DME	3.0	Ph_2SiH_2	MeOH	40 °C	N.D.
12	$CoBr_2$•DME	3.0	Ph_2SiH_2	PhOMe	40 °C	N.D.
13	$CoBr_2$•DME	3.0	Ph_2SiH_2	EtOAc	40 °C	N.D.
14	$CoBr_2$•DME	2.0	Ph_2SiH_2	THF	40 °C	65
15	$CoBr_2$•DME	2.0	Ph_2SiH_2	THF	40 °C	71[c]
16	$CoBr_2$•DME	2.0	DEMS	THF	40 °C	73[c]
17	CoI_2	2.0	DEMS	THF	40 °C	79[c]

[a] Conditions: All reactions were carried out with **Co-salt** (10 mol%), Ligand **L12** (10 mol%), **1a'** (0.10 mmol, 1.0 equiv), **2a** (0.20 mmol, 2.0 equiv), **[SiH]** (0.20 mmol), CsF (x equiv) and **Solvent** (0.5 mL) at the corresponding temperature (*T*) for 24 hours. [b] Isolated yields. [c] **Ligand L12** (12 mol%)

3. Synthesis *N*-Alkenyl Indoles

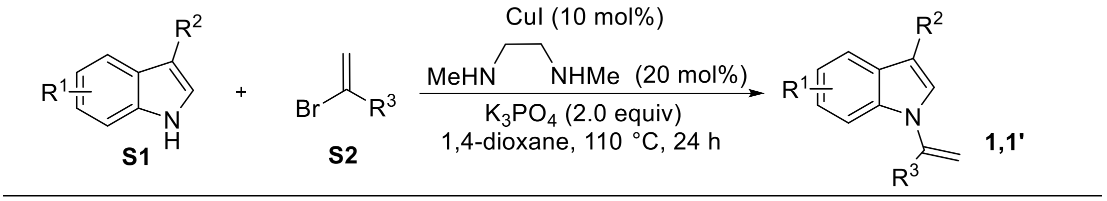

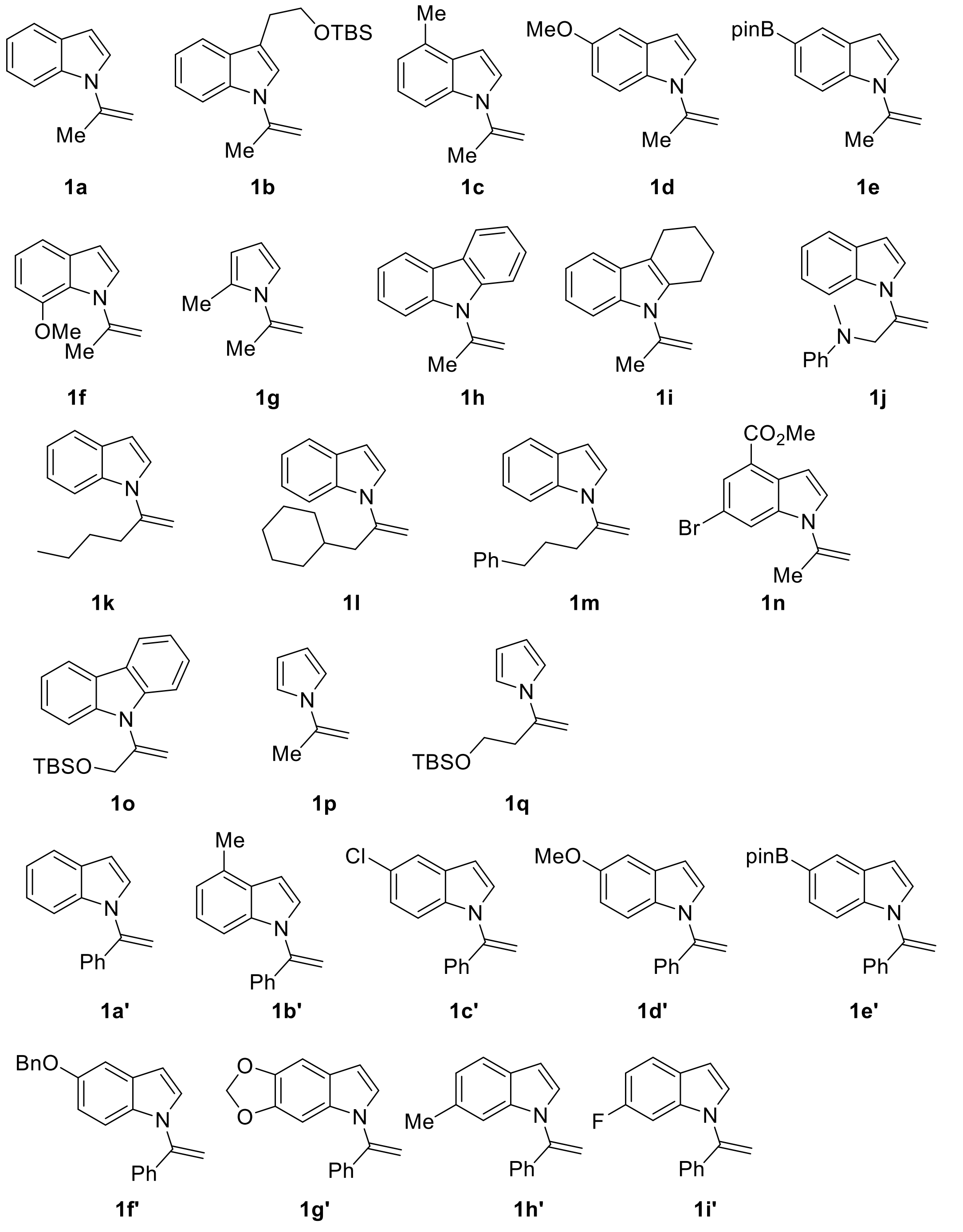

Supplementary Figure 1. Structural formula of *N*-Alkenyl Indoles

Following a reported procedure[1], CuI (10 mol%), substrate indole (12.0 mmol, 1.2

equiv.) and K_3PO_4 (20.0 mmol, 2.0 equiv.) were added to pre-dried a flask with a Teflon-lined septum. The flask was then evacuated and backfilled with Argon (3 cycles). Alkenyl bromide (10.0 mmol, 1.0 equiv), *N,N*-dimethylethane-1,2-diamine (20 mol%), and 1,4-dioxane (0.50 M) were added by syringe at room temperature. The flaske was then sealed and the reaction mixture was stirred at 110 °C for 24 h. The reaction was cooled to room temperature. Ethyl acetate (10.0 mL) was added and stirred for 10 min. The deposition was separated and washed with ethyl acetate (20.0 mL × 3). The organic phase was combined. The solvent was removed under vacuum. The crude product was purified by colum chromatography on silica gel to give corresponding *N*-alkenyl indol products **1a-1q, 1a'-1i',** all spectral data of **1a**[1], **1h**[1], **1p**[2], **1a'**[1], **1d'**[3], **1i'**[3].

3-(2-((*tert*-butyldimethylsilyl)oxy)ethyl)-1-(prop-1-en-2-yl)-1*H*-indole

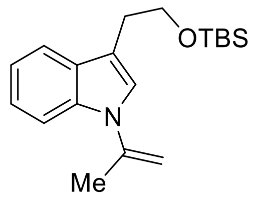

1b

Colorless liquid, 61% yield, flash column chromatography (SiO_2, 50:1 PE/EtOAc).

^{1}H NMR (500 MHz, $CDCl_3$) δ 7.63-7.60 (m, 2H), 7.24-7.21 (m, 1H), 7.16-7.13 (m, 1H), 7.09 (s, 1H), 5.14 (s, 1H), 5.03 (d, *J* = 1.3 Hz, 1H), 3.90 (t, *J* = 7.3 Hz, 2H), 2.99 (t, *J* = 7.3 Hz, 2H), 2.27 (s, 3H), 0.91 (s, 9H), 0.04 (s, 6H).

^{13}C NMR (126 MHz, $CDCl_3$) δ 140.6, 135.6, 129.2, 124.2, 122.1, 119.6, 119.0, 113.4, 111.9, 104.6, 63.6, 28.8, 26.0, 22.0, 18.4, -5.3.

HRMS (ESI) m/z: $[M + H]^+$ Calcd for $C_{19}H_{30}NOSi^+$ 316.2091; Found 316.2085.

4-methyl-1-(prop-1-en-2-yl)-1*H*-indole

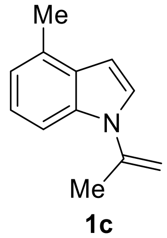

1c

Colorless liquid, 67% yield, flash column chromatography (SiO_2, PE).

^{1}H NMR (400 MHz, $CDCl_3$) δ 7.47 (d, *J* = 8.4 Hz, 1H), 7.21 (d, *J* = 3.2 Hz, 1H), 7.13

(t, J = 7.7 Hz, 1H), 6.93 (d, J = 7.1 Hz, 1H), 6.59 (d, J = 3.0 Hz, 1H), 5.16 (s, 1H), 5.06 (s, 1H), 2.56 (s, 3H), 2.27 (s, 3H).

^{13}C NMR (101 MHz, CDCl$_3$) δ 140.8, 135.1, 130.4, 129.1, 125.6, 122.2, 120.3, 109.4, 105.8, 101.3, 22.0, 18.7.

HRMS (ESI) m/z: $[M + H]^+$ Calcd for $C_{12}H_{14}N^+$ 172.1121; Found 172.1124.

5-methoxy-1-(prop-1-en-2-yl)-1*H*-indole

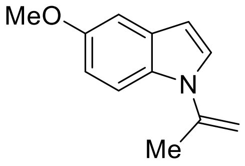

1d

White Solid, 77% yield, flash column chromatography (SiO_2, 100:1 PE/EtOAc).

^{1}H NMR (500 MHz, CDCl$_3$) δ 7.55 (d, J = 9.0 Hz, 1H), 7.22 (d, J = 3.3 Hz, 1H), 7.09 (d, J = 2.5 Hz, 1H), 6.89 (dd, J = 9.0, 2.5 Hz, 1H), 6.50 (d, J = 3.3 Hz, 1H), 5.15 (s, 1H), 5.02 (s, 1H), 3.87 (s, 3H), 2.28 (s, 3H).

^{13}C NMR (126 MHz, CDCl$_3$) δ 154.3, 140.7, 130.6, 130.0, 126.6, 112.7, 112.2, 104.5, 102.6, 102.5, 55.8, 21.9.

HRMS (ESI) m/z: $[M + H]^+$ Calcd for $C_{12}H_{14}NO^+$ 188.1070; Found 188.1065.

1-(prop-1-en-2-yl)-5-(4,4,5,5-tetramethyl-1,3,2-dioxaborolan-2-yl)-1*H*-indole

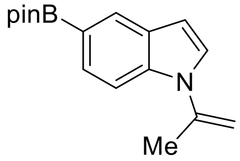

1e

White Solid, 48% yield, flash column chromatography (SiO_2, 50:1 PE/EtOAc).

^{1}H NMR (500 MHz, CDCl$_3$) δ 8.15 (s, 1H), 7.68-7.60 (m, 2H), 7.21 (d, J = 3.3 Hz, 1H), 6.59 (s, 1H), 5.18 (s, 1H), 5.09 (s, 1H), 2.28 (s, 3H), 1.37 (s, 12H).

^{13}C NMR (101 MHz, CDCl$_3$) δ 140.6, 137.3, 129.0, 128.8, 128.2, 126.3, 111.1, 106.2, 103.5, 83.6, 24.9, 22.0.

HRMS (ESI) m/z: $[M + H]^+$ Calcd for $C_{17}H_{23}BNO_2^+$ 283.1853; Found 283.1857.

7-methoxy-1-(prop-1-en-2-yl)-1*H*-indole

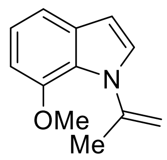

1f

White Solid, 24% yield, flash column chromatography (SiO_2, PE).

^{1}H NMR (500 MHz, $CDCl_3$) δ 7.28-7.27 (m, 1H), 7.09-7.05 (m, 2H), 6.70 (d, *J* = 7.6 Hz, 1H), 6.51-6.50 (m, 1H), 5.12 (s, 1H), 5.08 (s, 1H), 3.94 (s, 3H), 2.26 (s, 3H).

^{13}C NMR (126 MHz, $CDCl_3$) δ 147.0, 145.2, 131.5, 129.5, 125.5, 120.4, 113.7, 110.0, 103.2, 102.0, 55.6, 23.2.

HRMS (ESI) m/z: $[M + H]^+$ Calcd for $C_{12}H_{14}NO^+$ 188.1070; Found 188.1072.

2-methyl-1-(prop-1-en-2-yl)-1*H*-pyrrole

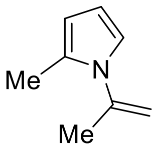

1g

Colorless liquid, 32% yield, flash column chromatography (SiO_2, PE).

^{1}H NMR (400 MHz, Acetone-*d_6*) δ 6.69-6.68 (m, 1H), 5.97 (t, *J* = 3.1 Hz, 1H), 5.84-5.83 (m, 1H), 5.03 (d, *J* = 1.2 Hz, 1H), 4.83 (s, 1H), 2.22 (s, 3H), 2.11 (s, 3H).

^{13}C NMR (101 MHz, Acetone-*d_6*) δ 142.17, 127.53, 119.22, 108.24, 107.64, 107.35, 21.84, 12.52.

HRMS (ESI) m/z: $[M + H]^+$ Calcd for $C_8H_{12}N^+$ 122.0964; Found 122.0965.

9-(prop-1-en-2-yl)-2,3,4,9-tetrahydro-1*H*-carbazole

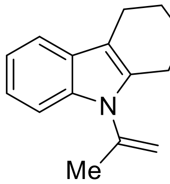

1i

White solid, 86% yield, flash column chromatography (SiO_2, PE).

^{1}H NMR (600 MHz, $CDCl_3$) δ 7.54 (d, *J* = 7.8 Hz, 1H), 7.38 (d, *J* = 8.1 Hz, 1H), 7.21-7.13 (m, 2H), 5.42 (d, *J* = 0.8 Hz, 1H), 5.14 (s, 1H), 2.82-2.80 (m, 2H), 2.76-2.74 (m, 2H), 2.19 (s, 3H), 1.99-1.92 (m, 4H).

^{13}C NMR (151 MHz, $CDCl_3$) δ 140.4, 136.0, 134.9, 127.5, 120.9, 119.1, 117.6, 113.6,

110.4, 110.1, 23.4, 23.1, 22.9, 21.9, 21.0.

HRMS (ESI) m/z: $[M + H]^+$ Calcd for $C_{15}H_{18}N^+$ 212.1434; Found 212.1432.

***N*-(2-(1*H*-indol-1-yl)allyl)-*N*-methylaniline**

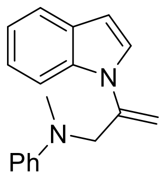

1j

White solid, 75% yield, flash column chromatography (SiO_2, 50:1 PE/EtOAc).

^{1}H NMR (600 MHz, CDCl$_3$) δ 7.63 (d, *J* = 7.8 Hz, 1H), 7.55-7.54 (m, 1H), 7.26-7.22 (m, 3H), 7.17 (d, *J* = 3.3 Hz, 1H), 7.16-7.13 (m, 1H), 6.76-6.74 (m, 3H), 6.58 (d, *J* = 3.2 Hz, 1H), 5.37 (s, 1H), 5.30 (s, 1H), 4.26 (s, 2H), 2.98 (s, 3H).

^{13}C NMR (151 MHz, CDCl$_3$) δ 148.9, 140.6, 136.0, 129.2, 129.0, 126.3, 122.3, 121.0, 120.3, 117.1, 112.4, 111.0, 108.8, 103.2, 56.5, 38.4.

HRMS (ESI) m/z: $[M + H]^+$ Calcd for $C_{18}H_{19}N_2^+$ 263.1543; Found 263.1546.

1-(hex-1-en-2-yl)-1*H*-indole

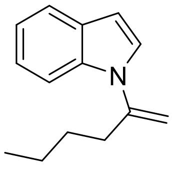

1k

Colorless oil, 53% yield, flash column chromatography (SiO_2, PE).

^{1}H NMR (600 MHz, Acetone-*d$_6$*) δ 7.58 (t, *J* = 8.3 Hz, 2H), 7.38 (d, *J* = 3.2 Hz, 1H), 7.18 (t, *J* = 7.7 Hz, 1H), 7.07 (t, J = 7.4 Hz, 1H), 6.57 (d, *J* = 3.2 Hz, 1H), 5.21 (s, 1H), 5.16 (s, 1H), 2.67 (t, *J* = 6.6 Hz, 2H), 1.34-1.33 (m, 4H), 0.84 (t, *J* = 6.7 Hz, 3H).

^{13}C NMR (151 MHz, Acetone-*d$_6$*) δ 145.3, 135.9, 129.4, 126.6, 122.0, 120.8, 119.9, 111.3, 106.1, 102.7, 34.7, 21.8, 13.2.

HRMS (ESI) m/z: $[M + H]^+$ Calcd for $C_{14}H_{18}N^+$ 200.1434; Found 200.1433.

1-(3-cyclohexylprop-1-en-2-yl)-1*H*-indole

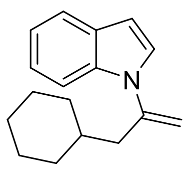

1l

White solid, 26% yield, flash column chromatography (SiO_2, PE).

^{1}H NMR (600 MHz, Acetone-*d$_6$*) δ 7.59 (t, *J* = 8.6 Hz, 2H), 7.39 (d, *J* = 3.2 Hz, 1H), 7.17 (t, *J* = 7.7 Hz, 1H), 7.07 (t, *J* = 7.4 Hz, 1H), 6.56 (d, *J* = 3.2 Hz, 1H), 5.18 (d, *J* = 5.6 Hz, 2H), 2.59 (d, *J* = 7.1 Hz, 2H), 1.70 (d, *J* = 11.8 Hz, 2H), 1.62-1.53 (m, 3H), 1.22-1.16 (m, 1H), 1.14-1.02 (m, 3H), 0.98-0.92 (m, 2H).

^{13}C NMR (151 MHz, Acetone-*d$_6$*) δ 143.8, 135.9, 129.5, 126.7, 122.0, 120.8, 119.9, 111.4, 106.9, 102.7, 42.9, 35.1, 32.7, 26.2, 25.9.

HRMS (ESI) m/z: $[M + H]^+$ Calcd for $C_{17}H_{22}N^+$ 240.1747; Found 240.1747.

1-(5-phenylpent-1-en-2-yl)-1*H*-indole

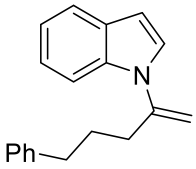

1m

Colorless oil, 50% yield, flash column chromatography (SiO_2, PE).

^{1}H NMR (600 MHz, Acetone-*d$_6$*) δ 7.59 (dd, *J* = 8.1, 3.0 Hz, 2H), 7.36 (d, *J* = 3.2 Hz, 1H), 7.23-7.17 (m, 3H), 7.13 (t, *J* = 7.3 Hz, 1H), 7.10-7.07 (m, 3H), 6.57 (d, *J* = 3.2 Hz, 1H), 5.24 (s, 1H), 5.20 (s, 1H), 2.71 (t, *J* = 7.4 Hz, 2H), 2.62 (d, *J* = 7.8 Hz, 2H), 1.68-1.63 (m, 2H).

^{13}C NMR (151 MHz, Acetone-*d$_6$*) δ 145.0, 141.9, 135.9, 129.4, 128.3, 128.3, 126.6, 125.7, 122.0, 120.8, 120.0, 111.2, 106.7, 102.8, 34.7, 34.5, 28.8.

HRMS (ESI) m/z: $[M + H]^+$ Calcd for $C_{19}H_{20}N^+$ 262.1590; Found 262.1589.

methyl 6-bromo-1-(prop-1-en-2-yl)-1*H*-indole-4-carboxylate

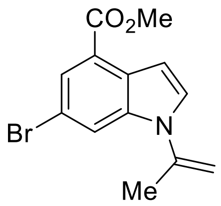

1n

White solid, 34% yield, flash column chromatography (SiO_2, 4:1 PE/DCM).

^{1}H NMR (400 MHz, $CDCl_3$) δ 8.01 (d, *J* = 1.7 Hz, 1H), 7.92 (dd, *J* = 1.7, 0.8 Hz, 1H), 7.31 (d, *J* = 3.3 Hz, 1H), 7.19 (dd, *J* = 3.3, 0.7 Hz, 1H), 5.20 (d, *J* = 1.2 Hz, 1H), 5.16 (s, 1H), 3.98 (s, 3H), 2.26 (s, 3H).

^{13}C NMR (101 MHz, $CDCl_3$) δ 166.6, 140.1, 137.0, 128.8, 127.7, 126.4, 122.8, 118.9, 114.6, 108.3, 104.0, 52.02, 21.98.

HRMS (ESI) m/z: $[M + H]^+$ Calcd for $C_{13}H_{13}BrNO_2^+$ 294.0124; Found 294.0119.

9-(3-((*tert*-butyldimethylsilyl)oxy)prop-1-en-2-yl)-9*H*-carbazole

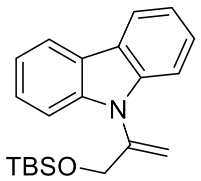

1o

Colorless oil, 73% yield, flash column chromatography (SiO_2, 100:1 PE/EtOAc).

^{1}H NMR (400 MHz, Acetone-*d*$_6$) δ 8.13 (d, *J* = 7.8 Hz, 2H), 7.46-7.41(m, 4H), 7.27-7.20 (m, 2H), 6.00 (t, *J* = 1.5 Hz, 1H), 5.54 (s, 1H), 4.50 (s, 2H), 0.83 (s, 9H), -0.02 (s, 6H).

^{13}C NMR (101 MHz, Acetone-*d*$_6$) δ 143.7, 140.7, 125.9, 123.1, 120.1, 119.6, 114.5, 109.9, 62.3, 25.3, 17.8, -6.2.

HRMS (ESI) m/z: $[M + H]^+$ Calcd for $C_{21}H_{28}NOSi^+$ 338.1935; Found 338.1935.

1-(4-((*tert*-butyldimethylsilyl)oxy)but-1-en-2-yl)-1*H*-pyrrole

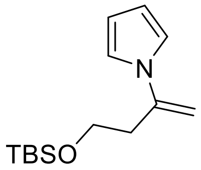

1q

Colorless oil, 67% yield, flash column chromatography (SiO_2, 100:1 PE/EtOAc).

^{1}H NMR (400 MHz, Acetone-*d*$_6$) δ 7.01 (t, *J* = 2.2 Hz, 2H), 6.14 (t, *J* = 1.9 Hz, 2H), 5.04 (s, 1H), 4.68 (s, 1H), 3.78 (t, *J* = 6.4 Hz, 2H), 2.77 (td, *J* = 6.4, 0.8 Hz, 2H), 0.88 (d, *J* = 1.2 Hz, 9H), 0.01 (d, *J* = 0.8 Hz, 6H).

^{13}C NMR (101 MHz, Acetone-*d*$_6$) δ 143.0, 118.5, 109.3, 99.7, 60.9, 37.0, 25.4, 17.9, -6.2.

HRMS (ESI) m/z: $[M + H]^+$ Calcd for $C_{14}H_{26}NOSi^+$ 252.1778; Found 252.1780.

4-methyl-1-(1-phenylvinyl)-1*H*-indole

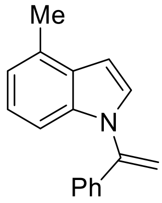

1b'

White solid, 70% yield, flash column chromatography (SiO_2, PE).

^{1}H NMR (500 MHz, $CDCl_3$) δ 7.40-7.33 (m, 5H), 7.21-7.20 (m, 1H), 7.07-6.95 (m, 3H), 6.67 (dd, *J* = 5.9, 3.3 Hz, 1H), 5.61 (d, *J* = 5.0 Hz, 1H), 5.39 (d, *J* = 5.4 Hz, 1H), 2.63-2.61 (m, 3H).

^{13}C NMR (126 MHz, $CDCl_3$) δ 145.1, 137.1, 136.1, 130.3, 129.1, 129.0, 128.5, 128.1, 126.9, 122.1, 120.4, 109.5, 108.2, 101.5, 18.7.

HRMS (ESI) m/z: $[M + H]^+$ Calcd for $C_{17}H_{16}N^+$ 234.1277; Found 234.1283.

5-chloro-1-(1-phenylvinyl)-1*H*-indole

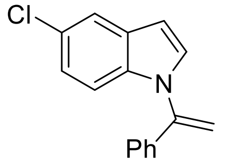

1c'

Yellow solid, 47% yield, flash column chromatography (SiO_2, 100:1 PE/EtOAc).

^{1}H NMR (500 MHz, $CDCl_3$) δ 7.64-7.63 (m, 1H), 7.42-7.29 (m, 5H), 7.23 (t, *J* = 3.7 Hz, 1H), 7.08-7.01 (m, 2H), 6.59-6.57 (m, 1H), 5.62 (d, *J* = 3.7 Hz, 1H), 5.38 (d, *J* = 3.5 Hz, 1H).

^{13}C NMR (126 MHz, $CDCl_3$) δ 144.7, 136.6, 134.7, 130.3, 129.9, 129.3, 128.7, 126.8, 125.8, 122.3, 120.2, 112.8, 108.5, 102.6.

HRMS (ESI) m/z: $[M + H]^+$ Calcd for $C_{16}H_{13}ClN^+$ 254.0731; Found 254.0730

1-(1-phenylvinyl)-5-(4,4,5,5-tetramethyl-1,3,2-dioxaborolan-2-yl)-1*H*-indole

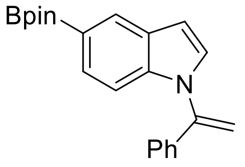

1e'

White solid, 43% yield, flash column chromatography (SiO_2, 20:1 PE/EtOAc).

^{1}H NMR (500 MHz, $CDCl_3$) δ 8.19 (s, 1H), 7.56 (dd, *J* = 8.4, 0.8 Hz, 1H), 7.39-7.32 (m, 3H), 7.30-7.28 (m, 2H), 7.17 (d, *J* = 3.3 Hz, 1H), 7.14 (d, *J* = 8.3 Hz, 1H), 6.64 (d, *J* = 2.9 Hz, 1H), 5.62 (s, 1H), 5.39 (s, 1H), 1.36 (s, 12H).

^{13}C NMR (126 MHz, $CDCl_3$) δ 144.8, 138.4, 136.8, 129.2, 128.9, 128.81, 128.77, 128.6, 128.1, 126.9, 111.2, 108.4, 103.6, 83.4, 24.9.

HRMS (ESI) m/z: $[M + H]^+$ Calcd for $C_{22}H_{25}BNO_2^+$ 345.2009; Found 345.2011.

5-(benzyloxy)-1-(1-phenylvinyl)-1*H*-indole

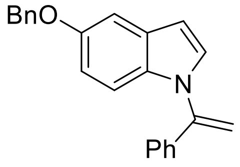

1f'

White solid, 49% yield, flash column chromatography (SiO_2, 30:1 PE/EtOAc).

^{1}H NMR (500 MHz, $CDCl_3$) δ 7.52-7.50 (m, 2H), 7.43-7.34 (m, 8H), 7.22 (d, *J* = 2.5 Hz, 1H), 7.19 (d, *J* = 3.4 Hz, 1H), 7.05 (d, *J* = 8.5 Hz, 1H), 6.88 (dd, *J* = 8.9, 2.5 Hz, 1H), 6.57 (d, *J* = 3.2 Hz, 1H), 5.55 (s, 1H), 5.36 (s, 1H), 5.14 (s, 2H).

^{13}C NMR (126 MHz, $CDCl_3$) δ 153.6, 145.1, 137.6, 137.0, 131.8, 129.8, 129.2, 129.1, 128.54, 128.48, 127.7, 127.5, 127.0, 112.71, 112.66, 107.4, 104.1, 102.8, 70.7.

HRMS (ESI) m/z: $[M + H]^+$ Calcd for $C_{23}H_{20}NO^+$ 326.1539; Found 326.1541.

5-(1-phenylvinyl)-5*H*-[1,3]dioxolo[4,5-*f*]indole

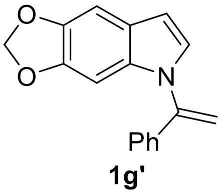

1g'

Gray solid, 72% yield, flash column chromatography (SiO_2, 40:1 PE/EtOAc).

^{1}H NMR (400 MHz, $CDCl_3$) δ 7.35-7.26 (m, 5H), 7.04 (d, *J* = 3.2 Hz, 1H), 7.00 (s,

1H), 6.54 (s, 1H), 6.47 (d, *J* = 3.2 Hz, 1H), 5.86 (s, 2H), 5.52 (s, 1H), 5.31 (s, 1H).

^{13}C NMR (101 MHz, $CDCl_3$) δ 145.0, 144.7, 143.1, 136.7, 131.4, 129.2, 128.6, 127.4, 126.9, 123.0, 108.0, 103.1, 100.6, 99.2, 93.0.

HRMS (ESI) m/z: $[M + H]^+$ Calcd for $C_{17}H_{14}NO_2^+$ 264.1019; Found 264.1023.

6-methyl-1-(1-phenylvinyl)-1*H*-indole

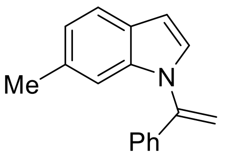

1h'

White solid, 67% yield, flash column chromatography (SiO_2, PE).

^{1}H NMR (500 MHz, $CDCl_3$) δ 7.56 (d, *J* = 8.0 Hz, 1H), 7.41-7.32 (m, 5H), 7.10 (d, *J* = 3.3 Hz, 1H), 7.01-6.98 (m, 2H), 6.59-6.58 (m, 1H), 5.62 (s, 1H), 5.39 (s, 1H), 2.39 (s, 3H).

^{13}C NMR (151 MHz, $CDCl_3$) δ 144.9, 137.1, 136.9, 131.8, 129.1, 128.5, 128.1, 127.0, 126.9, 122.0, 120.5, 111.7, 108.1, 102.8, 21.8.

HRMS (ESI) m/z: $[M + H]^+$ Calcd for $C_{17}H_{16}N^+$ 234.1277; Found 234.1277.

4. Preparation of Alkyl Halides

Alkyl Halides were purchased from commercial sources (**2a**, **2b**, **2e-2h**, **2m**, **2o**, **2p**, **2q**, **2x-2ac**).

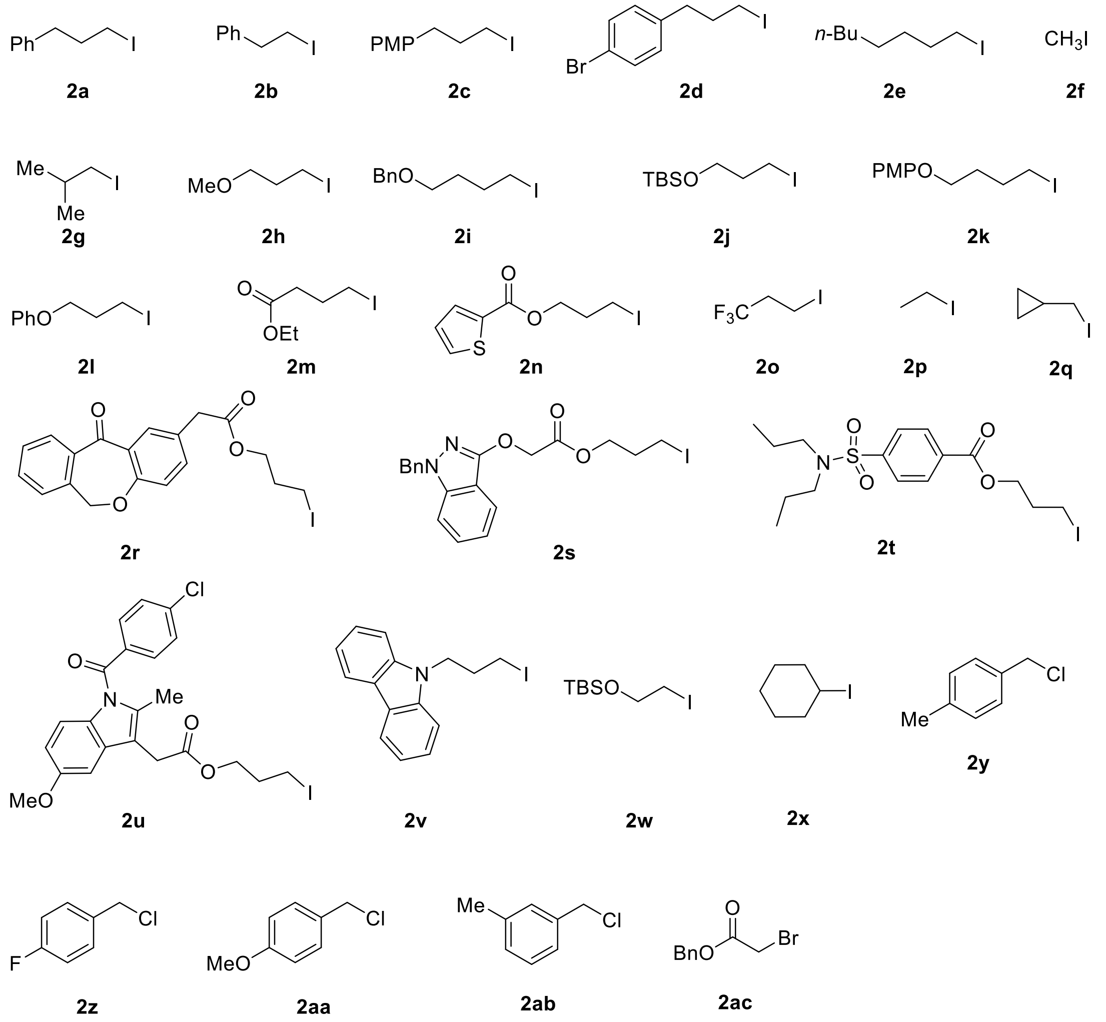

Supplementary Figure 2. Structural formula of Alkyl Halides

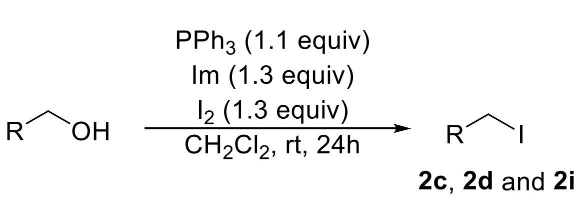

Following a reported procedure[4], Alcohol (10.0 mmol, 1.0 equiv) was dissolved in 50 mL of dry CH_2Cl_2, filled three times with Ar, and triphenylphosphine (11.0 mmol, 1.1 equiv) and imidazole (13.0 mmol, 1.3 equiv) were added at room temperature. Iodine (13.0 mmol, 1.3 equiv) was added in portions at 0 °C and covered with tin foil. The reaction was then allowed to rise to room temperature for 24 h. This reaction was

quenched by the addition of H_2O, the aqueous layer was extracted with CH_2Cl_2 and the combined organic layers were washed with an saturated sodium thiosulfate solution, dried with Na_2SO_4, filtered and concentrated under reduced pressure. The crude product was purified by colum chromatography on silica gel to give corresponding alkyl iodide compounds **2c**[4], **2d**[4] and **2i**[4].

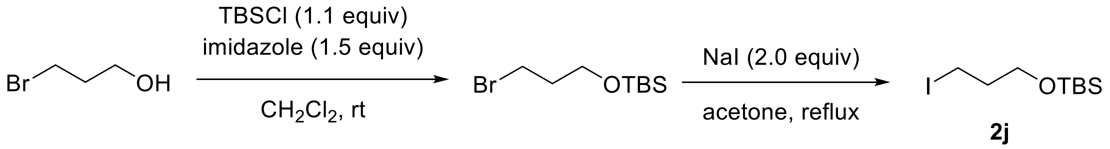

Following a reported procedure[5]. Step 1: To a solution of 3-bromo-1-propanol (20.0 mmol, 1.0 equiv) and imidazole (30.0 mmol, 1.5 equiv) in CH_2Cl_2 (50 mL) was added *tert*-butylchlorodimethylsilane (22.0 mmol, 1.1 equiv) at room temperature. After stirring for 30 min, the reaction mixture was diluted with CH_2Cl_2 and quenched with saturated NH_4Cl. After separation of the phases, the aqueous phase was extracted with CH_2Cl_2 and the combined organic phases were washed with brine, dried over Na_2SO_4 and filtered. The filtrate was concentrated in vacuo and the residue was used in the next reaction without further purification.

Step 2: To a solution of the above crude product in acetone (100 mL) was added sodium iodide (40.0 mmol, 2.0 equiv) at room temperature and the solution was heated at reflux for 12 h. The reaction mixture was quenched with saturated sodium thiosulfate solution. The resulting solution was extracted three times with EtOAc. The combined organic phases were washed with brine, dried over Na_2SO_4 and filtered. The filtrate was concentrated in vacuo and the residue was purified by flash column chromatography to give alkyl iodide compounds **2j**[5].

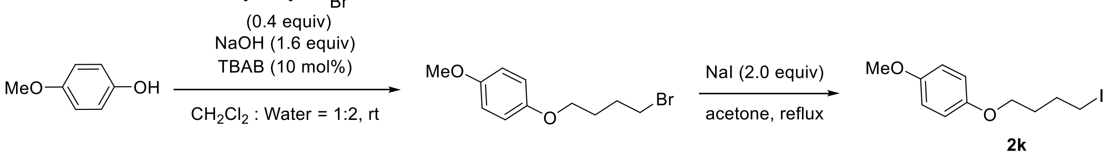

Following a reported procedure[6]. Step 1: Sodium hydroxide (32.0 mmol, 1.6 eq) and 4-methoxyphenol (20.0 mmol, 1.0 equiv) were dissolved in water (60 mL), then 1,3-dibromopropane (8.0 mmol, 0.4 equiv) and tetra-*N*-buthyl ammonium bromide (2.0

mmol, 10 mol%) dissolved in dichloromethane (30 mL) was added. The mixture was stirred for 24 h. The water phase was extracted three times with dichloromethane (100 mL). Combined organic extracts were evaporated, and the crude product was purified by column chromatography.

Step 2: To a solution of the above crude product (10.0 mmol, 1.0 equiv) in acetone (50 mL) was added sodium iodide (20.0 mmol, 2.0 equiv) at room temperature and the solution was heated at reflux for 12 h. The reaction mixture was quenched with saturated sodium thiosulfate solution. The resulting solution was extracted three times with EtOAc. The combined organic phases were washed with brine, dried over Na_2SO_4 and filtered. The filtrate was concentrated in vacuo and the residue was purified by flash column chromatography to give alkyl iodide compounds **2k**[6].

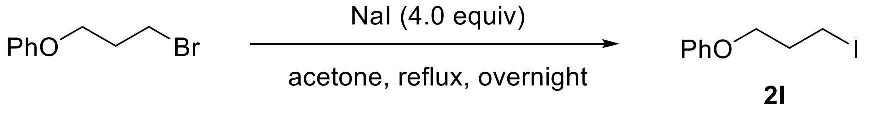

Following a reported procedure[7], In a 100 mL round-bottom flask, (3-bromopropoxy)benzene (10.0 mmol, 1.0 equiv) and NaI (40.0 mmol, 4 equiv) were dissolved in acetone (30 mL). The resulting solution was stirred overnight at reflux temperature. After cooling to room temperature, CH_2Cl_2 was added until the complete precipitation of salts. The mixture was filtered and the solvent was evaporated under vacuum. Then, the mixture was extracted with EtOAc and 0.1 M aqueous $Na_2S_2O_3$ solution. The combined organic phases were washed with saturated brine and dried over anhydrous Na_2SO_4, then filtered and concentrated by rotary evaporation. The crude product was purified by column chromatography on a silica gel (SiO_2, PE/EtOAc) to obtain the desired alkyl iodide **2l**[7].

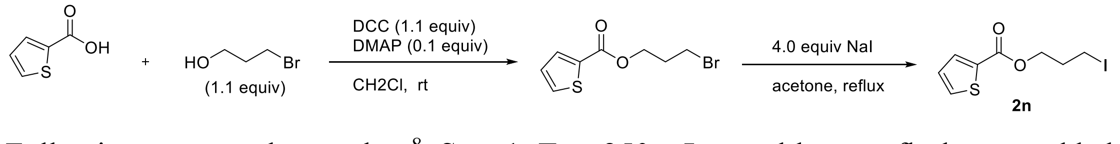

Following a reported procedure[8]. Step 1: To a 250 mL round-bottom flask were added thiophene-2-carboxylic acid (20.0 mmol, 1.0 equiv), DMAP (2.0 mmol, 10 mol%), anhydrous CH_2Cl_2 (50 mL), and 3-bromopropan-1-ol (22.0 mmol, 1.1 equiv). A solution of DCC (22.0 mmol, 1.1 equiv) in CH_2Cl_2 (10 mL) was added dropwise at 0

°C. After stirring for 12 h at room temperature, the reaction mixture was filtered with a pad of celite. The filtrate was concentrated, and the residue was purified with silica gel chromatography (SiO_2, PE/EtOAc) to give 3-bromopropyl thiophene-2-carboxylate.

Step 2: To a solution of the 3-bromopropyl thiophene-2-carboxylate (10.0 mmol, 1.0 equiv)in acetone (50 mL) was added sodium iodide (40.0 mmol, 4.0 equiv) at room temperature and the solution was heated at reflux for 12 h. The reaction mixture was quenched with saturated sodium thiosulfate solution. The resulting solution was extracted three times with EtOAc. The combined organic phases were washed with brine, dried over Na_2SO_4 and filtered. The filtrate was concentrated in vacuo and the residue was purified by flash column chromatography to give alkyl iodide compounds **2n**[8].

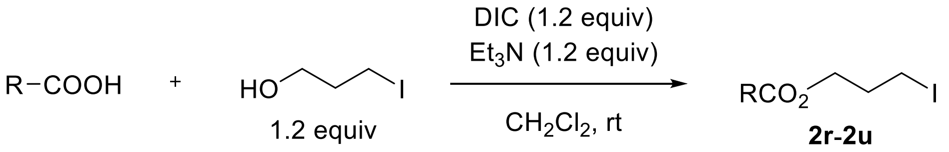

Following a reported procedure[9], To a stirred solution of carboxylic acid (10.0 mmol, 1.0 equiv.) in dry CH_2Cl_2 (25 mL) at 0 °C under a Argon atmosphere was added *N*,*N'*-diisopropylcarbodiimide (12.0 mmol, 1.2 equiv.). After 10 minutes, 3-iodo-1-propanol (12.0 mmol, 1.2 equiv.) and Et_3N (12.0 mmol 1.2 equiv.) were added to it. The resulting reaction mixture was allowed to warm to room temperature and the stirring was continued for overnight. The solution was diluted with CH_2Cl_2 and filtered through a plug of silica gel. The solvent was removed in *vacuo*. Flash column chromatography (SiO_2, PE/EtOAc) afforded the desired product **2r**[9]**, 2s, 2t**[9]**, 2u**[9]**.**

3-iodopropyl 2-((1-benzyl-1*H*-indazol-3-yl)oxy)acetate

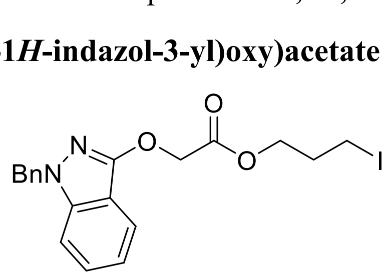

2s

White solid, 54% yield, flash column chromatography (SiO_2, 5:1 PE/EtOAc).

^{1}H NMR (600 MHz, $CDCl_3$) δ 7.72 (d, *J* = 8.1 Hz, 1H), 7.32 (t, *J* = 7.7 Hz, 1H), 7.29-7.22 (m, 3H), 7.18 (d, *J* = 8.5 Hz, 1H), 7.14 (d, *J* = 7.3 Hz, 2H), 7.06 (t, *J* = 7.5 Hz, 1H),

5.36 (s, 2H), 4.96 (s, 2H), 4.23 (t, $J = 5.9$ Hz, 2H), 3.07 (t, $J = 6.8$ Hz, 2H), 2.10-2.05 (m, 2H).

^{13}C NMR (151 MHz, CDCl$_3$) δ 168.8, 154.7, 141.8, 137.3, 128.6, 127.6, 127.5, 127.0, 120.0, 119.5, 112.3, 108.9, 65.4, 64.5, 52.3, 32.2, 1.1.

HRMS (ESI) m/z: $[M + H]^+$ Calcd for $C_{19}H_{20}IN_2O^+$ 451.0513; Found 451.0512.

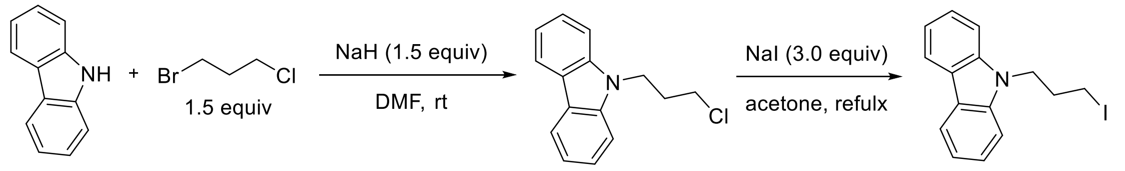

2v

Following a reported procedure[10]. Step 1: A 250 mL oven-dried round-bottom flask equipped with a Teflon-coated magnetic stirrer was charged with carbazole (20.0 mmol, 1.0 equiv) and sodium hydride (50.0 mmol, 1.5 equiv, 60% NaH suspension in oil used). The flask was degassed and refilled with Ar (this process was repeated for 3 times). Anhydrous DMF (80 mL) was then slowly added into the flask under Ar, and the reaction mixture was stirred at room temperature for 2 h. 1-Bromo-3-chloropropane (30.0 mmol, 1.5 equiv) was the added into the flask under Ar via syringe, and the reaction mixture was stirred at room temperature overnight. The reaction mixture was then washed with CH_2Cl_2 (50 mL) and water (200 mL). The aqueous solution was further washed with CH_2Cl_2 (2 x 50 mL). The combined organic layer was dried with anhydrous Na_2SO_4, filtered, and concentrated with the aid of a rotary evaporator. The residue was dried in vacuo to afford a crude product of in approximately quantitative yield.

Step 2: A 250 mL round-bottom flask equipped with a Teflon-coated magnetic stirrer was charged with 9-(3-chloropropyl)-9*H*-carbazole (~20.0 mmol. ~1.0 equiv), sodium iodide (60.0 mmol, 3.0 equiv), and acetone (100 mL). The reaction mixture was heated at reflux for 12 h. After cooling to room temperature, the reaction mixture was washed with CH_2Cl_2 (50 mL) and water (200 mL). The aqueous solution was further washed with CH_2Cl_2 (2 x 50 mL). The combined organic fractions were concentrated in *vacuo* with the aid of a rotary evaporator. The residue was purified by flash column chromatography with silica gel using a mixture of hexanes and EtOAc as an eluent to

afford the alkyl iodide product **2v**[10].

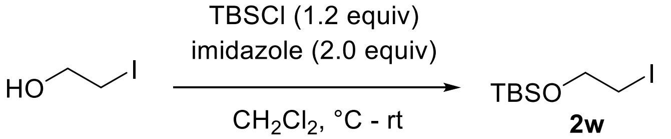

Following a reported procedure[11], to a solution of 2-iodoethanol (20.0 mmol, 1.0 equiv) in CH_2Cl_2 (50 mL) and imidazole (40.0 mmol, 2.0 equiv) was added *t*-butyldimethylsilyl chloride (24.0 mmol, 1.2 equiv) at 0 °C. After 30 min, the reaction mixture was allowed to warm to rt. After 2 h, the reaction mixture was diluted with CH_2Cl_2 (50 mL) and the organic layer was washed with saturated aqueous sodium bicarbonate solution (2 × 50 mL) and washed with brine (50 mL). The organic layer was dried over Na_2SO_4, the dried solution was filtered and the filtrate was concentrated. The crude product was purified by colum chromatography on silica gel to give corresponding alkyl iodide compounds **2w**[11].

5. Single crystal X-ray diffraction data

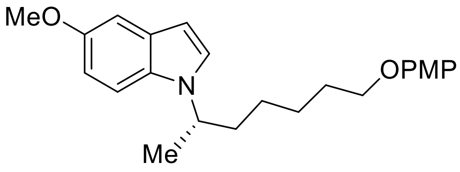

X-Ray of **3o** (CCDC 2258190)

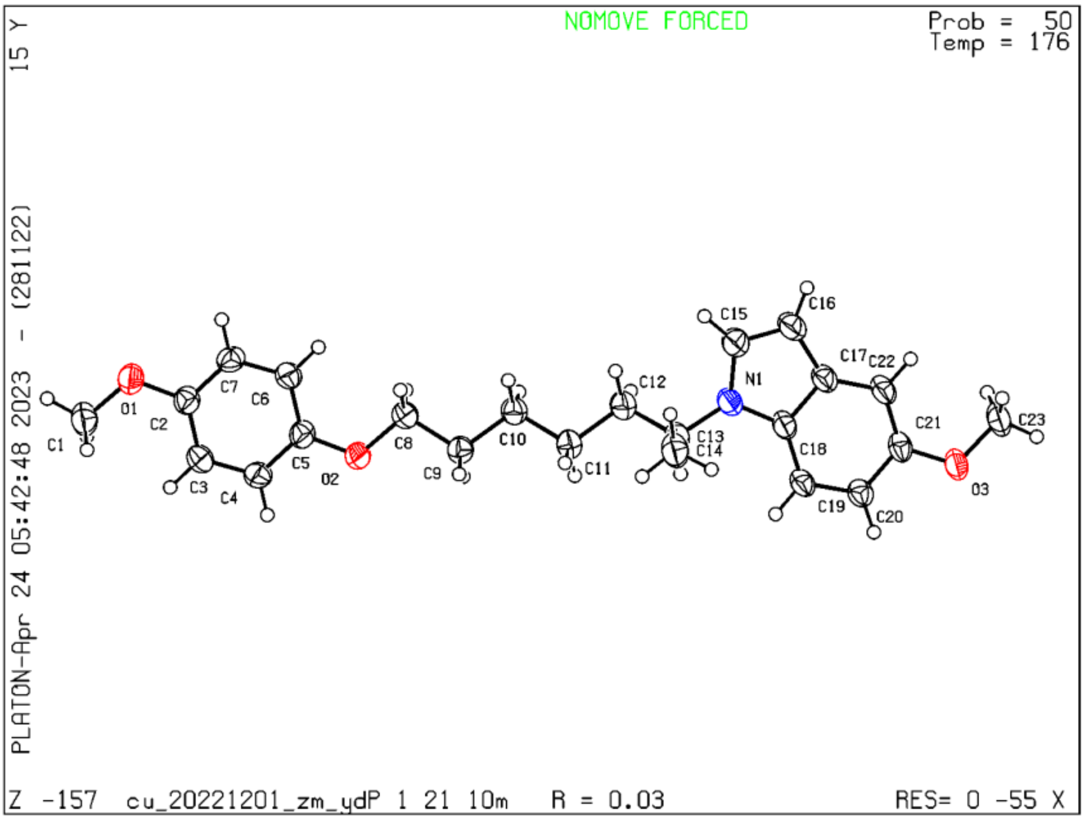

Supplementary Figure 3. Crystal data and structure of **3o**

Supplementary Table 5. Crystal data and structure refinement for cu_20221201_ZM_YD_RJT_SP_0m.

Identification code	cu_20221201_ZM_YD_RJT_SP_0m
Empirical formula	$C_{23}H_{29}NO_3$
Formula weight	367.47
Temperature/K	176.15
Crystal system	monoclinic
Space group	$P2_1$
a/Å	10.8536(2)

b/Å	7.4102(2)
c/Å	13.1050(3)
α/°	90
β/°	107.9040(10)
γ/°	90
Volume/Å3	1002.96(4)
Z	2
ρ_{calc}g/cm^3	1.217
μ/mm^{-1}	0.632
F(000)	396.0
Crystal size/mm^3	0.13 × 0.12 × 0.11
Radiation	CuKα (λ = 1.54178)
2Θ range for data collection/°	7.088 to 136.292
Index ranges	-13 ≤ h ≤ 13, -8 ≤ k ≤ 8, -15 ≤ l ≤ 15
Reflections collected	16795
Independent reflections	3646 [R_{int} = 0.0362, R_{sigma} = 0.0264]
Data/restraints/parameters	3646/1/248
Goodness-of-fit on F^2	1.050
Final R indexes [I>=2σ (I)]	R_1 = 0.0326, wR_2 = 0.0859
Final R indexes [all data]	R_1 = 0.0362, wR_2 = 0.0890
Largest diff. peak/hole / e Å^{-3}	0.11/-0.15
Flack parameter	0.1(3)

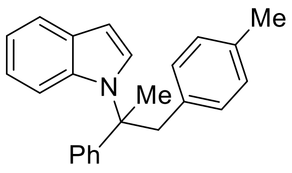

X-Ray of **8r** (CCDC 2321724)

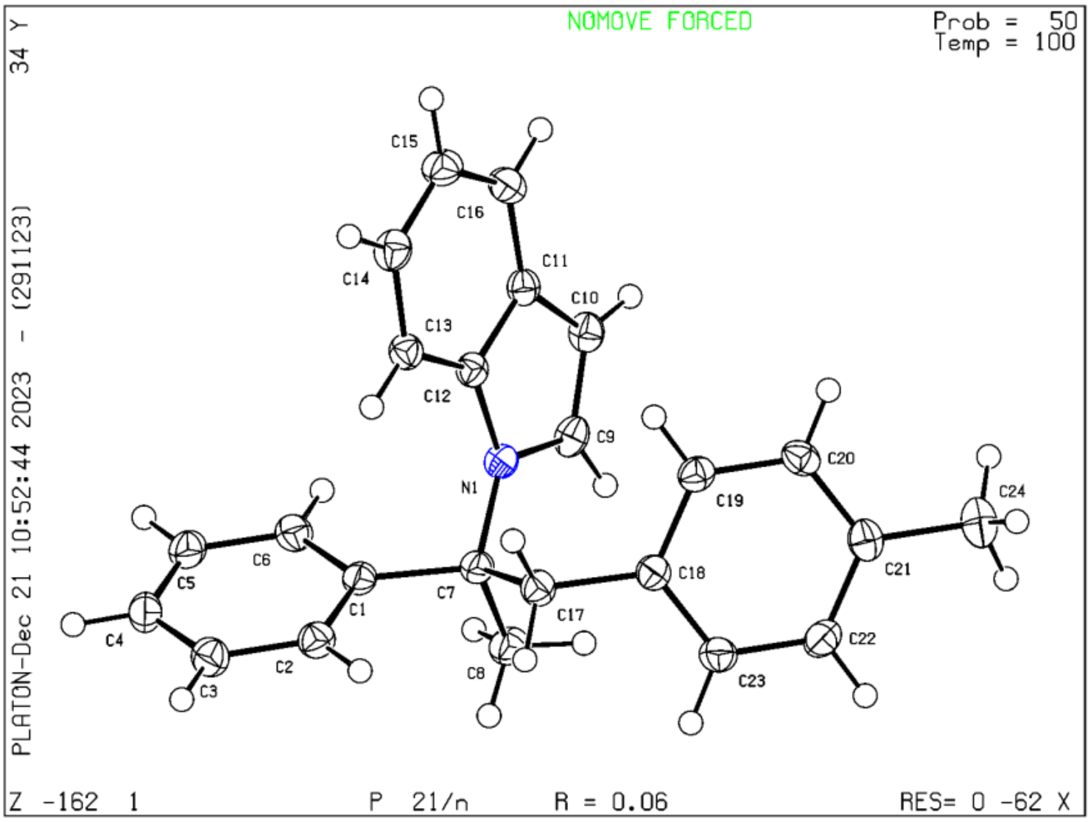

Supplementary Figure 4. Crystal data and structure of **8r**

Supplementary Table 6. Sample and crystal data for mo_231220F.

Identification code	mo_231220F	
Chemical formula	$C_{24}H_{23}N$	
Formula weight	325.43 g/mol	
Wavelength	0.71073 Å	
Crystal size	0.210 x 0.240 x 0.370 mm	
Crystal system	monoclinic	
Space group	P 1 21/n 1	
Unit cell dimensions	a = 7.5593(3) Å	α = 90°
	b = 13.5945(5) Å	β = 101.6210(10)°

	c = 18.0846(7) Å	γ = 90°
Volume	1820.37(12) $Å^3$	
Z	4	
Density (calculated)	1.187 g/cm^3	
Absorption coefficient	0.068 mm^{-1}	
F(000)	696	

Supplementary Table 7. Data collection and structure refinement for mo_231220F.

Theta range for data collection	1.89 to 28.33°	
Index ranges	-10<=h<=10, -15<=k<=18, -24<=l<=24	
Reflections collected	28479	
Independent reflections	4535 [R(int) = 0.0603]	
Max. and min. transmission	0.7457 and 0.7083	
Structure solution technique	direct methods	
Structure solution program	SHELXT 2018/2 (Sheldrick, 2018)	
Refinement method	Full-matrix least-squares on F^2	
Refinement program	SHELXL 2018/3 (Sheldrick, 2015)	
Function minimized	Σ w(F_o^2 - F_c^2)2	
Data / restraints / parameters	4535 / 0 / 228	
Goodness-of-fit on F2	1.062	
Final R indices	3612 data; I>2σ(I)	R1 = 0.0570, wR2 = 0.1178
	all data	R1 = 0.0776, wR2 = 0.1305
Weighting scheme	$w=1/[\sigma^2(F_o^2)+(0.0385P)^2+1.3826P]$	
	where $P=(F_o^2+2F_c^2)/3$	
Largest diff. peak and hole	0.319 and -0.337 $eÅ^{-3}$	
R.M.S. deviation from mean	0.053 $eÅ^{-3}$	

6. Co-Catalyzed Synthesis of *N*-Alkylated Indoles

6.1 *anti*-Markovnikov hydroalkylation of alkenes

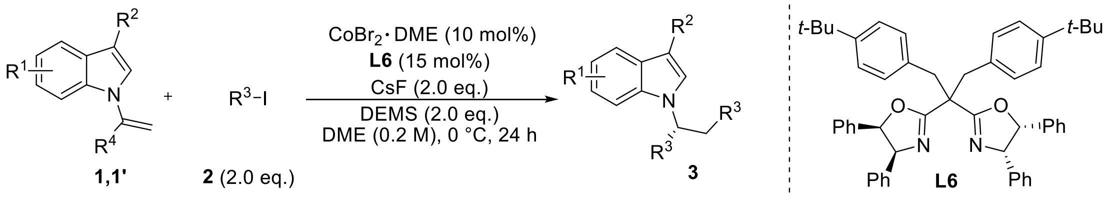

General Procedure 1 (GP1): To an oven-dried 10.0 mL Teflon-screw cap test tube containing a magnetic stir was charged with $CoBr_2$•DME (3.1 mg, 10 mol%) and ligand **L6** (11.3mg, 15 mol%) under a Argon atmosphere using glove-box techniques. Subsequently, anhydrous DME (0.5 mL) was added, and the mixture was stirred for 10 minutes at room temperature. Then, CsF (30.4 mg, 0.20 mmol, 2.0 equiv.), *N*-alkenyl indole **1** or **1'** (0.10 mmol, 1.0 equiv), alkyl iodide **2** (0.20 mmol, 2.0 equiv.) and $(OEt)_2MeSiH$ (32 uL, 0.20 mmol, 2.0 equiv.) were sequentially added. Afterwards, the tube was sealed with airtight electrical tapes and removed from the glove box and stirred at 0 °C for 24 hours at 500 rpm. After the reaction was completed, the reaction mixture was diluted with saturated NH_4Cl (aq., 2.0 mL) and EtOAc (3.0 mL). The aqueous phase was extracted with EtOAc (2 x 3.0 mL) and the combined organic phases were concentrated in vacuo. The crude mixture was purified by flash column chromatography on silica gel using a mixture of PE/EtOAc as eluent to obtain the desired product **3**.

(*S*)-1-(6-phenylhexan-2-yl)-1*H*-indole (3a)

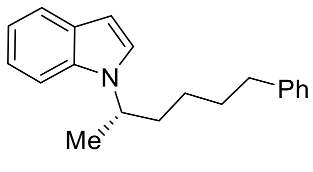

3a

Prepared according to **GP1** with *N*-alkenyl indole **1a** (15.7 mg, 0.10 mmol, 1.0 equiv.) and **2a** (49.2 mg, 0.20 mmol, 2.0 equiv.). Flash column chromatography (SiO_2, PE) afforded the desired product **3a** as a colorless oil (27.1 mg, 98%).

^{1}H NMR (500 MHz, $CDCl_3$) δ 7.64 (d, *J* = 7.9 Hz, 1H), 7.36 (d, *J* = 8.3 Hz, 1H), 7.26-7.23 (m, 2H), 7.21-7.14 (m, 3H), 7.11-7.08 (m, 3H), 6.53 (d, *J* = 3.1 Hz, 1H), 4.50-4.44

(m, 1H), 2.57-2.47 (m, 2H), 1.98-1.91 (m, 1H), 1.86-1.79 (m, 1H), 1.64-1.55 (m, 2H), 1.49 (d, *J* = 6.8 Hz, 3H), 1.35-1.19 (m, 2H).

^{13}C NMR (126 MHz, $CDCl_3$) δ 142.4, 135.9, 128.5, 128.3, 128.2, 125.7, 124.0, 121.1, 120.9, 119.2, 109.4, 101.3, 51.5, 37.0, 35.7, 31.2, 26.1, 21.3.

HRMS (ESI) m/z: $[M + H]^+$ Calcd for $C_{20}H_{24}N^+$ 278.1903; Found 278.1902.

HPLC: 95:5 er determined by analytical HPLC, Daicel CHIRALCEL® OD-H column, 25 °C, Hexane:*i*-PrOH = 95:5, 1.0 mL/min, 254 nm, t_{major} = 7.9 min, t_{minor} = 8.9 min.

$[\alpha]_D^{20}$ = -9.0 (*c* = 1.1, $CHCl_3$).

(*S*)-1-(5-phenylpentan-2-yl)-1*H*-indole (3b)

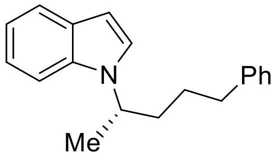

3b

Prepared according to **GP1** with *N*-alkenyl indole **1a** (15.7 mg, 0.10 mmol, 1.0 equiv.) and **2b** (46.4 mg, 0.20 mmol, 2.0 equiv.). Flash column chromatography (SiO_2, PE) afforded the desired product **3b** as a colorless oil (18.7 mg, 71%).

^{1}H NMR (500 MHz, $CDCl_3$) δ 7.66 (d, *J* = 7.9 Hz, 1H), 7.38 (d, *J* = 8.3 Hz, 1H), 7.28-7.27 (m, 1H), 7.26-7.25 (m, 1H), 7.23-7.10 (m, 6H), 6.55 (d, *J* = 3.2 Hz, 1H), 4.55-4.48 (m, 1H), 2.64-2.55 (m, 2H), 2.01-1.93 (m, 1H), 1.90-1.83 (m, 1H), 1.65-1.52 (m, 2H), 1.51 (d, *J* = 6.7 Hz, 3H).

^{13}C NMR (126 MHz, $CDCl_3$) δ 141.8, 135.9, 128.5, 128.34, 128.30, 125.8, 123.9, 121.2, 121.0, 119.2, 109.4, 101.5, 51.5, 36.6, 35.5, 28.0, 21.4.

HRMS (ESI) m/z: $[M + H]^+$ Calcd for $C_{19}H_{22}N^+$ 264.1747; Found 264.1750.

HPLC: 5:95 er determined by analytical HPLC, Daicel CHIRALPAK® AD-H column, 25 °C, Hexane:*i*-PrOH = 95:5, 1.0 mL/min, 254 nm, t_{minor} = 6.5 min, t_{major} = 6.9 min.

$[\alpha]_D^{20}$ = -25.5 (*c* = 0.55, $CHCl_3$).

(*S*)-1-(6-(4-methoxyphenyl)hexan-2-yl)-1*H*-indole (3c)

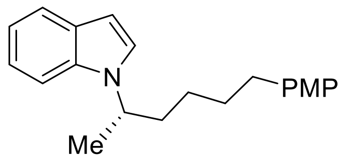

3c

Prepared according to **GP1** with *N*-alkenyl indole **1a** (15.7 mg, 0.10 mmol, 1.0 equiv.) and **2c** (55.2 mg, 0.20 mmol, 2.0 equiv.). Flash column chromatography (SiO_2, 100:1 PE/EtOAc) afforded the desired product **3c** as a colorless oil (30.1 mg, 98%).

^{1}H NMR (500 MHz, $CDCl_3$) δ 7.67 (d, *J* = 7.9 Hz, 1H), 7.39 (d, *J* = 8.3 Hz, 1H), 7.24-7.20 (m, 2H), 7.13 (t, *J* = 7.4 Hz, 1H), 7.04 (d, *J* = 8.6 Hz, 2H), 6.82 (d, *J* = 8.6 Hz, 2H), 6.56 (d, *J* = 3.1 Hz, 1H), 4.53-4.46 (m, 1H), 3.80 (s, 3H), 2.51-2.47 (m, 2H), 2.00-1.93 (m, 1H), 1.88-1.81 (m, 1H), 1.62-1.55 (m, 2H), 1.52 (d, *J* = 6.8 Hz, 3H), 1.37-1.29 (m, 1H), 1.27-1.20 (m, 1H).

^{13}C NMR (126 MHz, $CDCl_3$) δ 157.7, 135.9, 134.4, 129.2, 128.4, 124.0, 121.1, 120.9, 119.1, 113.7, 109.4, 101.3, 55.2, 51.5, 37.0, 34.7, 31.4, 26.0, 21.3.

HRMS (ESI) m/z: $[M + H]^+$ Calcd for $C_{21}H_{26}NO^+$ 308.2009; Found 308.2012.

HPLC: 94.5:5.5 er determined by analytical HPLC, Daicel CHIRALCEL® OD-H column, 25 °C, Hexane:*i*-PrOH = 95:5, 1.0 mL/min, 254 nm, t_{major} = 10.2 min, t_{minor} = 15.0 min.

$[\alpha]_D^{20}$ = -11.5 (*c* = 1.2, $CHCl_3$).

(*S*)-1-(6-(4-bromophenyl)hexan-2-yl)-5-methoxy-1*H*-indole (3d)

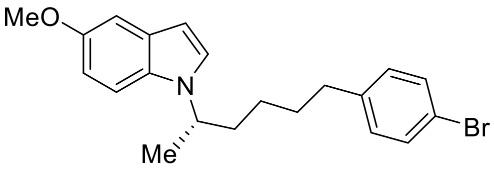

3d

Prepared according to **GP1** with *N*-alkenyl indole **1d** (18.7 mg, 0.10 mmol, 1.0 equiv.) and **2d** (65.0 mg, 0.20 mmol, 2.0 equiv.). Flash column chromatography (SiO_2, 100:1 PE/EtOAc) afforded the desired product **3d** as a colorless oil (32.4 mg, 84%).

^{1}H NMR (500 MHz, $CDCl_3$) δ 7.36 (d, *J* = 8.3 Hz, 2H), 7.26 (d, *J* = 8.9 Hz, 1H), 7.13 (dd, *J* = 11.6, 2.8 Hz, 2H), 6.96 (d, *J* = 8.3 Hz, 2H), 6.88 (dd, *J* = 8.9, 2.5 Hz, 1H), 6.46 (d, *J* = 3.1 Hz, 1H), 4.44-4.37 (m, 1H), 3.87 (s, 3H), 2.52-2.42 (m, 2H), 1.96-1.89 (m, 1H), 1.85-1.77 (m, 1H), 1.58-1.52 (m ,2H), 1.50 (d, *J* = 6.8 Hz, 3H), 1.32-1.24 (m, 1H), 1.22-1.15 (m, 1H).

^{13}C NMR (126 MHz, $CDCl_3$) δ 153.8, 141.2, 131.3, 131.2, 130.1, 128.7, 124.5, 119.4, 111.6, 110.1, 102.5, 100.9, 55.9, 51.68, 36.9, 35.0, 31.0, 25.9, 21.3.

HRMS (ESI) m/z: $[M + H]^+$ Calcd for $C_{21}H_{25}BrNO^+$ 386.1114; Found 386.1117.

HPLC: 95.5:4.5 er determined by analytical HPLC, Daicel CHIRALPAK® IB-3 column, 25 °C, Hexane:*i*-PrOH = 80:20, 0.8 mL/min, 254 nm, t_{major} = 10.4 min, t_{minor} = 13.3 min.

$[\alpha]_D^{20}$ = -5.8 (*c* = 1.3, $CHCl_3$).

(*S*)-5-methoxy-1-(undecan-2-yl)-1*H*-indole (3e)

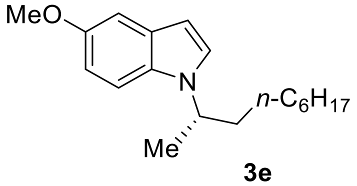

3e

Prepared according to **GP1** with *N*-alkenyl indole **1d** (18.7 mg, 0.10 mmol, 1.0 equiv.) and **2e** (48.0 mg, 0.20 mmol, 2.0 equiv.). Flash column chromatography (SiO_2, 100:1 PE/EtOAc) afforded the desired product **3e** as a colorless oil (30.2 mg, 67%).

^{1}H NMR (500 MHz, $CDCl_3$) δ 7.27 (d, *J* = 9.2 Hz, 1H), 7.13 (dd, *J* = 26.1, 2.7 Hz, 2H), 6.87 (dd, *J* = 8.9, 2.4 Hz, 1H), 6.45 (d, *J* = 3.0 Hz, 1H), 4.45-4.38 (m, 1H), 3.86 (s, 3H), 1.93-1.86 (m, 1H), 1.82-1.75 (m, 1H), 1.49 (d, *J* = 6.8 Hz, 3H), 1.30-1.23 (m, 14H), 0.88 (t, *J* = 7.0 Hz, 3H).

^{13}C NMR (126 MHz, $CDCl_3$) δ 153.8, 131.3, 128.7, 124.6, 111.5, 110.2, 102.5, 100.7, 55.9, 51.8, 37.1, 31.8, 29.5, 29.42, 29.39, 29.2, 26.4, 22.6, 21.3, 14.1.

HRMS (ESI) m/z: $[M + H]^+$ Calcd for $C_{20}H_{32}NO^+$ 302.2478; Found 302.2481.

HPLC: 95:5 er determined by analytical HPLC, Daicel CHIRALCEL® OJ-H column, 25 °C, Hexane:*i*-PrOH = 90:10, 1.0 mL/min, 254 nm, t_{major} = 5.9 min, t_{minor} = 7.2 min.

$[\alpha]_D^{20}$ = -11.5 (*c* = 0.8, $CHCl_3$).

(*S*)-1-(*sec*-butyl)-1*H*-indole

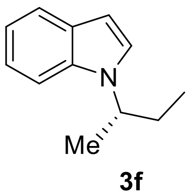

3f

Prepared according to **GP1** with *N*-alkenyl indole **1a** (15.7 mg, 0.10 mmol, 1.0 equiv.)

and **2f** (28.4 mg, 0.20 mmol, 2.0 equiv.). Flash column chromatography (SiO_2, PE) afforded the desired product **3f** as a colorless oil (11.8 mg, 68%).

^{1}H NMR (500 MHz, $CDCl_3$) δ 7.68 (d, *J* = 7.9 Hz, 1H), 7.42 (d, *J* = 8.3 Hz, 1H), 7.25-7.22 (m, 2H), 7.14 (t, *J* = 7.4 Hz, 1H), 6.57 (d, *J* = 3.0 Hz, 1H), 4.48-4.41 (m, 1H), 2.00-1.86 (m, 2H), 1.54 (d, *J* = 6.8 Hz, 3H), 0.87 (t, *J* = 7.4 Hz, 3H).

^{13}C NMR (126 MHz, $CDCl_3$) δ 136.0, 128.4, 124.0, 121.1, 120.9, 119.1, 109.5, 101.2, 53.0, 30.1, 20.9, 10.9.

HRMS (ESI) m/z: $[M + H]^+$ Calcd for $C_{12}H_{16}N^+$ 174.1277; Found 174.1278.

HPLC: 95.5:4.5 er determined by analytical HPLC, Daicel CHIRALCEL® OD-H column, 25 °C, Hexane:*i*-PrOH = 97:3, 1.0 mL/min, 254 nm, t_{major} = 5.1 min, t_{minor} = 5.5 min.

$[\alpha]_D^{20}$ = +2.6 (*c* = 0.57, $CHCl_3$).

(*S*)-1-(5-methylhexan-2-yl)-1*H*-indole (3g)

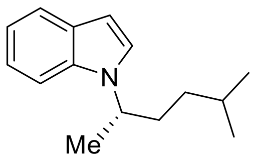

3g

Prepared according to **GP1** with *N*-alkenyl indole **1a** (15.7 mg, 0.10 mmol, 1.0 equiv.) and **2g** (36.8 mg, 0.20 mmol, 2.0 equiv.). Flash column chromatography (SiO_2, PE) afforded the desired product **3g** as a colorless oil (17.0 mg, 79%).

^{1}H NMR (500 MHz, $CDCl_3$) δ 7.64 (d, J = 7.9 Hz, 1H), 7.38 (d, J = 8.3 Hz, 1H), 7.22 -7.19 (m, 2H), 7.10 (t, J = 7.7 Hz, 1H), 6.54 (d, J = 3.1 Hz, 1H), 4.49-4.42 (m, 1H), 1.95-1.89 (m, 1H), 1.85-1.78 (m, 1H), 1.55-1.50 (m, 4H), 1.22-1.15 (m, 1H), 1.10-1.03 (m, 1H), 0.85 (d, J = 6.6 Hz, 6H).

^{13}C NMR (126 MHz, $CDCl_3$) δ 135.9, 128.5, 123.9, 121.1, 120.9, 119.1, 109.4, 101.3, 51.9, 35.5, 35.0, 27.9, 22.5, 22.4, 21.4.

HRMS (ESI) m/z: $[M + H]^+$ Calcd for $C_{15}H_{22}N^+$ 216.1747; Found 216.1750.

HPLC: 95:5 er determined by analytical HPLC, Daicel CHIRALCEL® OD-H column, 25 °C, Hexane:*i*-PrOH = 98:2, 0.8 mL/min, 254 nm, t_{major} = 5.7 min, t_{minor} = 6.0 min.

$[\alpha]_D^{20}$ = -14.9 (*c* = 0.46, $CHCl_3$).

(*S*)-1-(6,6,6-trifluorohexan-2-yl)-1*H*-indole (3h)

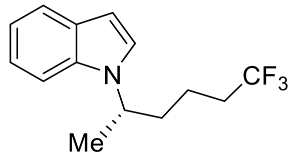

3h

Prepared according to **GP1** with *N*-alkenyl indole **1a** (15.7 mg, 0.10 mmol, 1.0 equiv.) and **2o** (44.8 mg, 0.20 mmol, 2.0 equiv.). Flash column chromatography (SiO_2, 100:1 PE/EtOAc) afforded the desired product **3h** as a colorless oil (19.9 mg, 78%).

^{1}H NMR (500 MHz, $CDCl_3$) δ 7.65 (d, J = 7.9 Hz, 1H), 7.37 (d, J = 8.3 Hz, 1H), 7.22 (t, J = 7.6 Hz, 1H), 7.17 (d, J = 3.2 Hz, 1H), 7.12 (t, J = 7.4 Hz, 1H), 6.56 (d, J = 3.2 Hz, 1H), 4.55-4.48 (m, 1H), 2.08-1.88 (m, 4H), 1.58-1.52 (m, 4H), 1.47-1.39 (m, 1H).

^{13}C NMR (126 MHz, $CDCl_3$) δ 135.8, 128.5, 126.9 (d, $J_{C\text{-}F}$ = 276.3 Hz), 123.7, 121.4, 121.1, 119.4, 109.2, 101.9, 51.2, 36.1, 33.4 (q, $J_{C\text{-}F}$ = 28.7 Hz), 21.2, 19.0 (q, $J_{C\text{-}F}$ = 3.0 Hz).

^{19}F NMR (471 MHz, $CDCl_3$) δ -66.16.

HRMS (ESI) m/z: $[M + H]^+$ Calcd for $C_{14}H_{17}F_3N^+$ 256.1308; Found 256.1308.

HPLC: 5:95 er determined by analytical HPLC, Daicel CHIRALCEL® OJ-H column, 25 °C, Hexane:*i*-PrOH = 88:12, 1.0 mL/min, 254 nm, t_{minor} = 7.9 min, t_{major} = 8.3 min.

$[\alpha]_D^{20}$ = -10.1 (c = 0.84, $CHCl_3$).

ethyl (*S*)-6-(1H-indol-1-yl)heptanoate (3i)

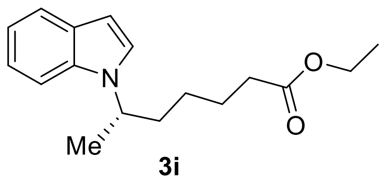

3i

Prepared according to **GP1** with *N*-alkenyl indole **1a** (15.7 mg, 0.10 mmol, 1.0 equiv.) and **2m** (48.4 mg, 0.20 mmol, 2.0 equiv.). Flash column chromatography (SiO_2, 50:1 PE/EtOAc) afforded the desired product **3i** as a colorless oil (22.9 mg, 84%).

^{1}H NMR (500 MHz, $CDCl_3$) δ 7.63 (d, J = 7.9 Hz, 1H), 7.37 (d, J = 8.3 Hz, 1H), 7.21 -7.17 (m, 2H), 7.09 (t, J = 7.4 Hz, 1H), 6.53 (d, J = 3.2 Hz, 1H), 4.53-4.46 (m, 1H), 4.09 (q, J = 7.1 Hz, 2H), 2.22 (t, J = 7.5 Hz, 2H), 1.98-1.90 (m, 1H), 1.87-1.80 (m, 1H), 1.65-1.60 (m, 2H), 1.51 (d, J = 6.8 Hz, 3H), 1.34-1.25 (m, 1H), 1.25-1.14 (m, 4H).

^{13}C NMR (126 MHz, CDCl$_3$) δ 173.5, 135.9, 128.4, 123.9, 121.2, 120.9, 119.2, 109.4, 101.4, 60.2, 51.4, 36.8, 34.1, 25.9, 24.7, 21.3, 14.2.

HRMS (ESI) m/z: $[M + H]^+$ Calcd for $C_{17}H_{24}NO_2^+$ 274.1802; Found 274.1809.

HPLC: 96:4 er determined by analytical HPLC, Daicel CHIRALCEL® OD-H column, 25 °C, Hexane:*i*-PrOH = 95:5, 1.0 mL/min, 254 nm, t_{major} = 10.9 min, t_{minor} = 14.0 min.

$[\alpha]_D^{20}$ = -8.8 (*c* = 0.92, $CHCl_3$).

(*S*)-5-(1*H*-indol-1-yl)hexyl thiophene-2-carboxylate (3j)

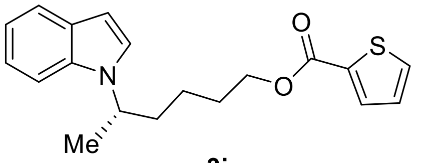

3j

Prepared according to **GP1** with *N*-alkenyl indole **1a** (15.7 mg, 0.10 mmol, 1.0 equiv.) and **2n** (59.2 mg, 0.20 mmol, 2.0 equiv.). Flash column chromatography (SiO_2, 30:1 PE/EtOAc) afforded the desired product **3j** as a colorless oil (20.3 mg, 62%).

^{1}H NMR (500 MHz, CDCl$_3$) δ 7.72 (dd, *J* = 3.7, 1.2 Hz, 1H), 7.63 (d, *J* = 7.9 Hz, 1H), 7.54 (dd, *J* = 5.0, 1.2 Hz, 1H), 7.38 (d, *J* = 8.3 Hz, 1H), 7.21-7.18 (m, 2H), 7.11-7.08 (m, 2H), 6.53 (d, *J* = 3.2 Hz, 1H), 4.52 (dq, *J* = 13.3, 6.7 Hz, 1H), 4.22 (td, *J* = 6.6, 1.6 Hz, 2H), 2.03-1.95 (m, 1H), 1.93-1.86 (m, 1H), 1.77-1.67 (m, 2H), 1.53 (d, *J* = 6.8 Hz, 3H), 1.44-1.35 (m, 1H), 1.33-1.25 (m, 1H).

^{13}C NMR (126 MHz, CDCl$_3$) δ 162.2, 135.9, 133.9, 133.3, 132.2, 128.5, 127.7, 123.9, 121.2, 120.9, 119.2, 109.4, 101.5, 64.7, 51.4, 36.7, 28.4, 22.8, 21.3.

HRMS (ESI) m/z: $[M + H]^+$ Calcd for $C_{19}H_{22}NO_2S^+$ 328.1366; Found 328.1365.

HPLC: 94.5:5.5 er determined by analytical HPLC, Daicel CHIRALPAK® IB-3 column, 25 °C, Hexane:*i*-PrOH = 80:20, 1.0 mL/min, 254 nm, t_{major} = 8.1 min, t_{minor} = 12.0 min.

$[\alpha]_D^{20}$ = -7.9 (*c* = 0.82, $CHCl_3$).

(*S*)-1-(6-methoxyhexan-2-yl)-1*H*-indole (3k)

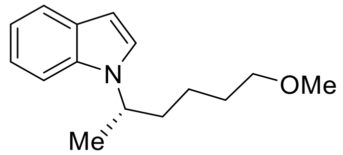

3k

Prepared according to **GP1** with *N*-alkenyl indole **1a** (15.7 mg, 0.10 mmol, 1.0 equiv.) and **2h** (40.0 mg, 0.20 mmol, 2.0 equiv.). Flash column chromatography (SiO_2, 100:1 PE/EtOAc) afforded the desired product **3k** as a colorless oil (18.0 mg, 78%).

^{1}H NMR (500 MHz, $CDCl_3$) δ 7.64 (d, *J* = 7.9 Hz, 1H), 7.38 (d, *J* = 8.3 Hz, 1H), 7.22-7.19 (m, 2H), 7.10 (t, *J* = 7.0 Hz, 1H), 6.54 (d, *J* = 3.2 Hz, 1H), 4.53-4.46 (m, 1H), 3.31-3.29 (m, 5H), 1.99-1.91 (m, 1H), 1.88-1.81 (m, 1H), 1.59-1.53 (m, 2H), 1.52 (d, *J* = 6.8 Hz, 3H), 1.40-1.31 (m, 1H), 1.29-1.20 (m, 1H).

^{13}C NMR (126 MHz, $CDCl_3$) δ 135.9, 128.4, 123.9, 121.1, 120.9, 119.1, 109.4, 101.4, 72.5, 58.5, 51.5, 37.0, 29.4, 23.1, 21.3.

HRMS (ESI) m/z: $[M + H]^+$ Calcd for $C_{15}H_{22}NO^+$ 232.1696; Found 232.1694.

HPLC: 96.5:3.5 er determined by analytical HPLC, Daicel CHIRALCEL® OJ-H column, 25 °C, Hexane:*i*-PrOH = 95:5, 1.0 mL/min, 254 nm, t_{major} = 12.6 min, t_{minor} = 15.8 min.

$[\alpha]_D^{20}$ = -12.6 (*c* = 0.72, $CHCl_3$).

(*S*)-1-(7-(benzyloxy)heptan-2-yl)-1*H*-indole (3l)

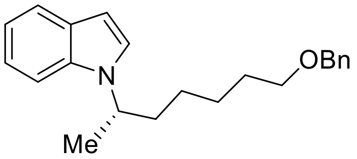

3l

Prepared according to **GP1** with *N*-alkenyl indole **1a** (15.7 mg, 0.10 mmol, 1.0 equiv.) and **2i** (58.0 mg, 0.20 mmol, 2.0 equiv.). Flash column chromatography (SiO_2, 60:1 PE/EtOAc) afforded the desired product **3l** as a colorless oil (31.5 mg, 98%).

^{1}H NMR (500 MHz, $CDCl_3$) δ 7.65 (d, *J* = 7.9 Hz, 1H), 7.39-7.28 (m, 6H), 7.23-7.19 (m, 2H), 7.13-7.10 (m, 1H), 6.55 (d, *J* = 3.1 Hz, 1H), 4.53-4.48 (m, 3H), 3.42 (t, *J* = 6.5 Hz, 2H), 1.98-1.90 (m, 1H), 1.86-1.79 (m, 1H), 1.60-1.54 (m, 2H), 1.51 (d, *J* = 6.8 Hz, 3H), 1.42-1.35 (m, 2H), 1.33-1.26 (m, 1H), 1.25-1.16 (m, 1H).

^{13}C NMR (126 MHz, $CDCl_3$) δ 138.6, 135.9, 128.4, 128.3, 127.6, 127.5, 123.9, 121.1, 120.9, 119.1, 109.4, 101.3, 72.8, 70.2, 51.5, 37.1, 29.5, 26.2, 26.0, 21.3.

HRMS (ESI) m/z: $[M + H]^+$ Calcd for $C_{22}H_{28}NO^+$ 322.2165; Found 322.2170.

HPLC: 5:95 er determined by analytical HPLC, Daicel CHIRALPAK® IB-3 column,

25 °C, Hexane:*i*-PrOH = 90:10, 1.0 mL/min, 254 nm, t_{minor} = 8.0 min, t_{major} = 9.2 min.

[*α*]$_D^{20}$ = -10.7 (*c* = 1.3, $CHCl_3$).

(*S*)-1-(6-((*tert*-butyldimethylsilyl)oxy)hexan-2-yl)-1*H*-indole (3m)

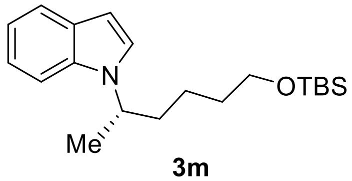

3m

Prepared according to **GP1** with *N*-alkenyl indole **1a** (15.7 mg, 0.10 mmol, 1.0 equiv.) and **2j** (60.0 mg, 0.20 mmol, 2.0 equiv.). Flash column chromatography (SiO_2, PE) afforded the desired product **3n** as a colorless oil (22.6 mg, 68%).

^{1}H NMR (500 MHz, $CDCl_3$) δ 7.64 (d, *J* = 7.8 Hz, 1H), 7.39 (d, *J* = 8.3 Hz, 1H), 7.22-7.19 (m, 2H), 7.10 (t, *J* = 7.4 Hz, 1H), 6.54 (d, *J* = 3.1 Hz, 1H), 4.54-4.47 (m, 1H), 3.54 (t, *J* = 6.5 Hz, 2H), 1.97-1.81 (m, 2H), 1.53-1.46 (m, 5H), 1.38-1.28 (m, 1H), 1.26-1.17 (m, 1H), 0.88 (s, 9H), 0.01 (d, *J* = 2.6 Hz, 6H).

^{13}C NMR (126 MHz, $CDCl_3$) δ 135.9, 128.4, 123.9, 121.1, 120.9, 119.1, 109.4, 101.3, 62.8, 51.5, 37.0, 32.5, 25.9, 22.7, 21.3, 18.3, -5.3.

HRMS (ESI) m/z: $[M + H]^+$ Calcd for $C_{20}H_{34}NOSi^+$ 332.2404; Found 332.2406.

HPLC: 95:5 er determined by analytical HPLC, Daicel CHIRALPAK® IB-3 column, 25 °C, Hexane:*i*-PrOH = 100:0, 1.0 mL/min, 254 nm, t_{major} = 22.8 min, t_{minor} = 29.8 min.

[*α*]$_D^{20}$ = -8.9 (*c* = 1.1, $CHCl_3$).

(*S*)-5-methoxy-1-(6-phenoxyhexan-2-yl)-1*H*-indole (3n)

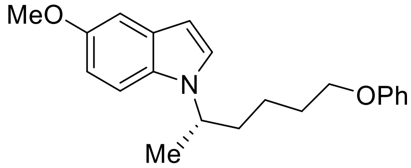

3n

Prepared according to **GP1** with *N*-alkenyl indole **1d** (18.7 mg, 0.10 mmol, 1.0 equiv.) and **2l** (52.4 mg, 0.20 mmol, 2.0 equiv.). Flash column chromatography (SiO_2, 50:1 PE/EtOAc) afforded the desired product **3n** as a colorless oil (28.7 mg, 89%).

^{1}H NMR (500 MHz, $CDCl_3$) δ 7.26-7.23 (m, 3H), 7.14 (d, *J* = 3.1 Hz, 1H), 7.09 (d, *J* = 2.4 Hz, 1H), 6.91 (t, *J* = 7.3 Hz, 1H), 6.86-6.82 (m, 3H), 6.44 (d, *J* = 3.1 Hz, 1H), 4.46-4.39 (m, 1H), 3.87-3.84 (m, 5H), 1.99-1.92 (m, 1H), 1.89-1.82 (m, 1H), 1.77-1.67

(m, 2H), 1.49 (d, J = 6.8 Hz, 3H), 1.44-1.38 (m, 1H), 1.35-1.28 (m, 1H).

^{13}C NMR (126 MHz, CDCl$_3$) δ 158.9, 153.9, 131.2, 129.4, 128.7, 124.5, 120.5, 114.5, 111.6, 110.1, 102.6, 100.9, 67.4, 55.9, 51.7, 36.8, 29.0, 23.0, 21.3.

HRMS (ESI) m/z: $[M + H]^+$ Calcd for $C_{21}H_{26}NO_2^+$ 324.1958; Found 324.1962.

HPLC: 95:5 er determined by analytical HPLC, Daicel CHIRALPAK® IB-3 column, 25 °C, Hexane:*i*-PrOH = 80:20, 0.8 mL/min, 254 nm, t_{major} = 22.8 min, t_{minor} = 25.9 min.

$[\alpha]_D^{20}$ = -6.7 (c = 1.2, $CHCl_3$).

(*S*)-5-methoxy-1-(7-(4-methoxyphenoxy)heptan-2-yl)-1*H*-indole (3o)

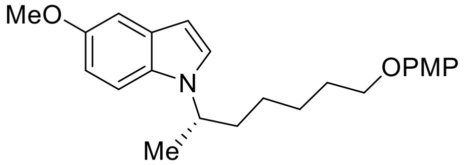

3o

Prepared according to **GP1** with *N*-alkenyl indole **1d** (18.7 mg, 0.10 mmol, 1.0 equiv.) and **2k** (61.2 mg, 0.20 mmol, 2.0 equiv.). Flash column chromatography (SiO_2, 30:1 PE/EtOAc) afforded the desired product **3o** as a white solid (35.2 mg, 96%).

^{1}H NMR (500 MHz, CDCl$_3$) δ 7.24 (d, J = 2.1 Hz, 1H), 7.14 (d, J = 2.8 Hz, 1H), 7.09 (d, J = 2.2 Hz, 1H), 6.85 (dd, J = 8.9, 2.3 Hz, 1H), 6.79 (q, J = 9.3 Hz, 4H), 6.43 (d, J = 2.8 Hz, 1H), 4.45-4.38 (m, 1H), 3.84 (s, 3H), 3.82 (t, J = 6.4 Hz, 2H), 3.76 (s, 3H), 1.95-1.88 (m, 1H), 1.84-1.77 (m, 1H), 1.70-1.62 (m, 2H), 1.49 (d, J = 6.8 Hz, 3H), 1.46-1.38 (m, 2H), 1.34-1.26 (m, 1H), 1.24-1.16 (m, 1H).

^{13}C NMR (151 MHz, CDCl$_3$) δ 155.9, 153.7, 153.2, 136.6, 122.9, 122.8, 121.4, 115.4, 114.6, 108.9, 101.3, 93.4, 68.4, 55.8, 55.7, 51.4, 37.0, 29.2, 26.2, 25.9, 21.3.

HRMS (ESI) m/z: $[M + H]^+$ Calcd for $C_{23}H_{30}NO_3^+$ 368.2220; Found 368.2225.

HPLC: 5.5:94.5 er determined by analytical HPLC, Daicel CHIRALPAK® AD-H column, 25 °C, Hexane:*i*-PrOH = 80:20, 1.0 mL/min, 254 nm, t_{minor} = 11.5 min, t_{major} = 14.6 min.

$[\alpha]_D^{20}$ = -4.7 (c = 0.62, $CHCl_3$).

(*S*)-9-(5-(5-methoxy-1H-indol-1-yl)hexyl)-9*H*-carbazole (3p)

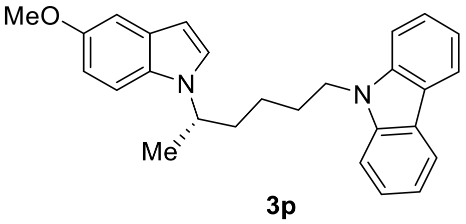

3p

Prepared according to **GP1** with *N*-alkenyl indole **1d** (18.7 mg, 0.10 mmol, 1.0 equiv.) and **2v** (67.0 mg, 0.20 mmol, 2.0 equiv.). Flash column chromatography (SiO_2, 50:1 PE/EtOAc) afforded the desired product **3p** as a colorless oil (39.3 mg, 99%).

^{1}H NMR (500 MHz, $CDCl_3$) δ 8.11 (d, J = 7.7 Hz, 2H), 7.45 (t, J = 7.7 Hz, 2H), 7.30 (d, J = 8.2 Hz, 2H), 7.24 (dd, J = 14.9, 7.6 Hz, 3H), 7.12 (dd, J = 11.8, 2.7 Hz, 2H), 6.89 (dd, J = 8.9, 2.4 Hz, 1H), 6.47 (d, J = 3.0 Hz, 1H), 4.39-4.32 (m, 1H), 4.24-4.13 (m, 2H), 3.89 (s, 3H), 1.95-1.83 (m, 2H), 1.81-1.74 (m, 2H), 1.45 (d, J = 6.8 Hz, 3H), 1.35-1.30 (m, 1H), 1.28-1.21 (m, 1H).

^{13}C NMR (126 MHz, $CDCl_3$) δ 153.9, 140.3, 131.3, 128.7, 125.6, 124.4, 122.8, 120.3, 118.8, 111.7, 110.1, 108.5, 102.6, 101.1, 55.9, 51.5, 42.8, 37.0, 28.6, 24.2, 21.2.

HRMS (ESI) m/z: $[M + H]^+$ Calcd for $C_{27}H_{29}N_2O^+$ 397.2274; Found 397.2278.

HPLC: 4.5:95.5 er determined by analytical HPLC, Daicel CHIRALPAK® AD-H column, 25 °C, Hexane:*i*-PrOH = 90:10, 1.0 mL/min, 254 nm, t_{minor} = 13.6 min, t_{major} = 14.3 min.

$[\alpha]_D^{20}$ = -9.7 (c = 1.6, $CHCl_3$).

(*S*)-3-(2-((tert-butyldimethylsilyl)oxy)ethyl)-1-(6-(4-methoxyphenyl)hexan-2-yl)-1*H*-indole (3q)

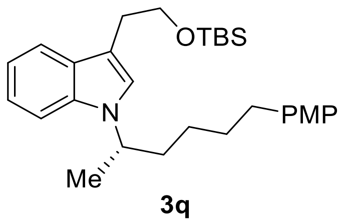

3q

Prepared according to **GP1** with *N*-alkenyl indole **1b** (31.6 mg, 0.10 mmol, 1.0 equiv.) and **2c** (55.2 mg, 0.20 mmol, 2.0 equiv.). Flash column chromatography (SiO_2, 80:1 PE/EtOAc) afforded the desired product **3q** as a colorless oil (24.7 mg, 53%).

^{1}H NMR (500 MHz, $CDCl_3$) δ 7.60 (d, J = 7.8 Hz, 1H), 7.33 (d, J = 8.3 Hz, 1H), 7.20-

7.18 (m, 1H), 7.11-7.08 (m, 1H), 7.03-7.02 (m, 3H), 6.82-6.79 (m, 2H), 4.46-4.40 (m, 1H), 3.89 (t, *J* = 7.3 Hz, 2H), 3.79 (s, 3H), 3.00 (t, *J* = 7.2 Hz, 2H), 2.52-2.44 (m, 2H), 1.95-1.88 (m, 1H), 1.83-1.78 (m, 1H), 1.60-1.53 (m, 2H), 1.47 (d, *J* = 6.8 Hz, 3H), 1.34-1.19 (m, 2H), 0.92 (s, 9H), 0.04 (s, 6H).

^{13}C NMR (126 MHz, $CDCl_3$) δ 157.7, 136.1, 134.5, 129.2, 128.0, 122.0, 121.0, 119.0, 118.5, 113.7, 112.0, 109.3, 63.9, 55.2, 51.2, 37.0, 34.8, 31.5, 29.1, 26.1, 26.0, 21.3, 18.4, -5.3.

HRMS (ESI) m/z: $[M + H]^+$ Calcd for $C_{29}H_{44}NO_2Si^+$ 466.3136; Found 466.3141.

HPLC: 94:6 er determined by analytical HPLC, Daicel CHIRALCEL® OD-H column, 25 °C, Hexane:*i*-PrOH = 95:5, 1.0 mL/min, 254 nm, t_{major} = 5.5 min, t_{minor} = 7.1 min.

$[\alpha]_D^{20}$ = -1.4 (*c* = 0.68, $CHCl_3$).

(*S*)-1-(6-(4-methoxyphenyl)hexan-2-yl)-4-methyl-1*H*-indole (3r)

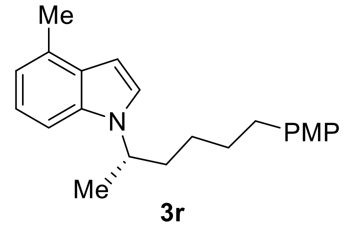

3r

Prepared according to **GP1** with *N*-alkenyl indole **1c** (17.1 mg, 0.10 mmol, 1.0 equiv.) and **2c** (55.2 mg, 0.20 mmol, 2.0 equiv.). Flash column chromatography (SiO_2, 80:1 PE/EtOAc) afforded the desired product **3r** as a colorless oil (28.6 mg, 89%).

^{1}H NMR (500 MHz, $CDCl_3$) δ 7.23 (d, *J* = 8.3 Hz, 1H), 7.19 (d, *J* = 3.2 Hz, 1H), 7.15-7.11 (m, 1H), 7.03 (d, *J* = 8.5 Hz, 2H), 6.92 (d, *J* = 7.0 Hz, 1H), 6.81 (d, *J* = 8.5 Hz, 2H), 6.56 (d, *J* = 3.1 Hz, 1H), 4.51-4.44 (m, 1H), 3.80 (s, 3H), 2.59 (s, 3H), 2.49 (td, *J* = 8.0, 3.3 Hz, 2H), 2.00-1.93 (m, 1H), 1.88-1.80 (m, 1H), 1.63-1.55 (m, 2H), 1.51 (d, *J* = 6.8 Hz, 3H), 1.37-1.31 (m, 1H), 1.29-1.21 (m, 1H).

^{13}C NMR (126 MHz, $CDCl_3$) δ 157.7, 135.6, 134.5, 130.3, 129.2, 128.3, 123.3, 121.3, 119.4, 113.7, 107.1, 99.8, 55.2, 51.6, 37.0, 34.8, 31.4, 26.0, 21.3, 18.7.

HRMS (ESI) m/z: $[M + H]^+$ Calcd for $C_{22}H_{28}NO^+$ 322.2165; Found 322.2166.

HPLC: 95:5 er determined by analytical HPLC, Daicel CHIRALCEL® OD-H column, 25 °C, Hexane:*i*-PrOH = 95:5, 1.0 mL/min, 254 nm, t_{major} = 10.2 min, t_{minor} = 14.7 min.

[α]$_D^{20}$ = -5.5 (c = 1.1, $CHCl_3$).

(*S*)-1-(6-(4-methoxyphenyl)hexan-2-yl)-5-(4,4,5,5-tetramethyl-1,3,2-dioxaborolan-2-yl)-1*H*-indole (3s)

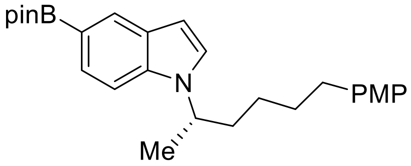

3s

Prepared according to **GP1** with *N*-alkenyl indole **1e** (28.3 mg, 0.10 mmol, 1.0 equiv.) and **2c** (55.2 mg, 0.20 mmol, 2.0 equiv.). Flash column chromatography (SiO_2, 10:1 PE/EtOAc) afforded the desired product **3s** as a colorless oil (28.6 mg, 66%).

^{1}H NMR (500 MHz, $CDCl_3$) δ 8.18 (s, 1H), 7.65 (d, J = 9.1 Hz, 1H), 7.36 (d, J = 8.4 Hz, 1H), 7.16 (d, J = 3.2 Hz, 1H), 7.01 (d, J = 8.6 Hz, 2H), 6.80-6.79 (m, 2H), 6.55 (d, J = 3.2 Hz, 1H), 4.52-4.46 (m, 1H), 3.78 (s, 3H), 2.46 (td, J = 7.9, 3.5 Hz, 2H), 1.97-1.89 (m, 1H), 1.85-1.78 (m, 1H), 1.60-1.52 (m, 2H), 1.50 (d, J = 6.8 Hz, 3H), 1.38 (s, 12H), 1.29-1.25 (m, 1H), 1.23-1.14 (m, 1H).

^{13}C NMR (126 MHz, $CDCl_3$) δ 157.7, 137.9, 134.4, 129.2, 129.0, 128.2, 127.3, 124.1, 113.7, 108.9, 102.2, 83.3, 55.2, 51.5, 37.0, 34.7, 31.4, 26.0, 24.9, 21.3.

HRMS (ESI) m/z: $[M + H]^+$ Calcd for $C_{27}H_{37}BNO_3^+$433.2897; Found 433.2894.

HPLC: 7:93 er determined by analytical HPLC, Daicel CHIRALPAK® AD-H column, 25 °C, Hexane:*i*-PrOH = 90:10, 1.0 mL/min, 254 nm, t_{minor} = 6.3 min, t_{major} = 9.3 min.

[α]$_D^{20}$ = -16.8 (c = 0.56, $CHCl_3$).

(*S*)-1-(6-((*tert*-butyldimethylsilyl)oxy)hexan-2-yl)-5-methoxy-1*H*-indole (3t)

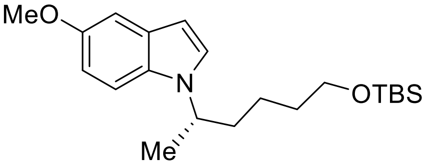

3t

Prepared according to **GP1** with *N*-alkenyl indole **1d** (18.7 mg, 0.10 mmol, 1.0 equiv.) and **2j** (60.0 mg, 0.20 mmol, 2.0 equiv.). Flash column chromatography (SiO_2, 100:1 PE/EtOAc) afforded the desired product **3t** as a colorless oil (26.8 mg, 74%).

^{1}H NMR (500 MHz, $CDCl_3$) δ 7.25 (d, J = 7.5 Hz, 1H), 7.14 (d, J = 3.0 Hz, 1H), 7.09 (d, J = 2.4 Hz, 1H), 6.86 (dd, J = 8.9, 2.4 Hz, 1H), 6.43 (d, J = 3.0 Hz, 1H), 4.45-4.38

(m, 1H), 3.85 (s, 3H), 3.53 (t, J = 6.5 Hz, 2H), 1.94-1.87 (m, 1H), 1.84-1.77 (m, 1H), 1.50-1.44 (m, 5H), 1.34-1.26 (m, 1H), 1.22-1.17 (m, 1H), 0.86 (s, 9H), 0.00 (d, J = 2.2 Hz, 6H).

^{13}C NMR (126 MHz, CDCl$_3$) δ 153.8, 131.3, 128.7, 124.6, 111.6, 110.1, 102.6, 100.8, 62.9, 55.9, 51.8, 37.0, 32.5, 25.9, 22.8, 21.3, 18.3, -5.3.

HRMS (ESI) m/z: [M + H]$^+$ Calcd for $C_{21}H_{36}NO_2Si^+$ 362.2510; Found 362.2513.

HPLC: 95:5 er determined by analytical HPLC, Daicel CHIRALCEL® OJ-H column, 25 °C, Hexane:*i*-PrOH = 90:10, 1.0 mL/min, 254 nm, t_{major} = 5.0 min, t_{minor} = 5.8 min.

$[\alpha]_D^{20}$ = -5.8 (c = 1.1, $CHCl_3$).

(*S*)-5-methoxy-1-(6-phenylhexan-2-yl)-1*H*-indole (3u)

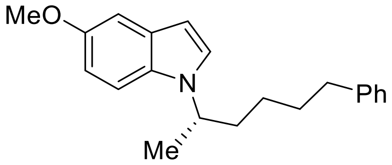

3u

Prepared according to **GP1** with *N*-alkenyl indole **1d** (18.7 mg, 0.10 mmol, 1.0 equiv.) and **2a** (49.2 mg, 0.20 mmol, 2.0 equiv.). Flash column chromatography (SiO_2, 100:1 PE/EtOAc) afforded the desired product **3u** as a colorless oil (26.7 mg, 87%).

^{1}H NMR (500 MHz, CDCl$_3$) δ 7.29-7.26 (m, 3H), 7.19 (t, J = 7.4 Hz, 1H), 7.16 (d, J = 3.0 Hz, 1H), 7.13-7.12 (m, 3H), 6.89 (dd, J = 8.9, 2.4 Hz, 1H), 6.47 (d, J = 3.0 Hz, 1H), 4.46-4.39 (m, 1H), 3.88 (s, 3H), 2.58-2.50 (m, 2H), 1.99-1.91 (m, 1H), 1.87-1.80 (m, 1H), 1.67-1.57 (m, 2H), 1.51 (d, J = 6.8 Hz, 3H), 1.37-1.20 (m, 2H).

^{13}C NMR (126 MHz, CDCl$_3$) δ 153.9, 142.4, 131.3, 128.7, 128.3, 128.2, 125.7, 124.6, 111.6, 110.2, 102.6, 100.8, 55.9, 51.7, 37.0, 35.7, 31.2, 26.1, 21.3.

HRMS (ESI) m/z: [M + H]$^+$ Calcd for $C_{21}H_{26}NO^+$308.2009; Found 308.2013.

HPLC: 95:5 er determined by analytical HPLC, Daicel CHIRALPAK® IB-3 column, 25 °C, Hexane:*i*-PrOH = 95:5, 1.0 mL/min, 254 nm, t_{major} = 8.0 min, t_{minor} = 11.3 min.

$[\alpha]_D^{20}$ = -9.8 (c = 1.1, $CHCl_3$).

(*S*)-7-methoxy-1-(6-phenylhexan-2-yl)-1*H*-indole (3v)

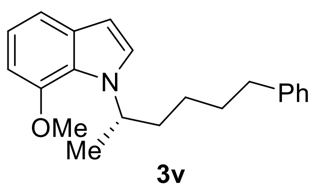

3v

Prepared according to **GP1** with *N*-alkenyl indole **1f** (18.7 mg, 0.10 mmol, 1.0 equiv.) and **2a** (49.2 mg, 0.20 mmol, 2.0 equiv.). Flash column chromatography (SiO_2, PE) afforded the desired product **3v** as a colorless oil (19.6 mg, 64%).

^{1}H NMR (500 MHz, $CDCl_3$) δ 7.28-7.26 (m, 1H), 7.25-7.23 (m, 2H), 7.19-7.16 (m, 2H), 7.13 (d, J = 7.0 Hz, 2H), 7.00 (t, J = 7.8 Hz, 1H), 6.64 (d, J = 7.7 Hz, 1H), 6.51 (d, J = 3.2 Hz, 1H), 5.37-5.36 (m, 1H), 3.92 (s, 3H), 2.57-2.52 (m, 2H), 1.94-1.87 (m, 1H), 1.80-1.73 (m, 1H), 1.69-1.56 (m, 2H), 1.47 (d, J = 6.8 Hz, 3H), 1.40-1.31 (m, 1H), 1.29-1.22 (m, 1H).

^{13}C NMR (126 MHz, $CDCl_3$) δ 147.6, 142.6, 130.4, 128.3, 128.2, 125.9, 125.6, 123.9, 119.3, 113.7, 102.5, 101.8, 55.2, 52.8, 38.1, 35.8, 31.2, 25.9, 22.2.

HRMS (ESI) m/z: $[M + H]^+$ Calcd for $C_{21}H_{26}NO^+$ 308.2009; Found 308.2008.

HPLC: 95:5 er determined by analytical HPLC, Daicel CHIRALPAK® IC-3 column, 25 °C, Hexane:*i*-PrOH = 98:2, 0.8 mL/min, 254 nm, t_{major} = 9.1 min, t_{minor} = 9.7 min.

$[\alpha]_D^{20}$ = -37.5 (c = 0.58, $CHCl_3$).

(*S*)-9-(6-(4-methoxyphenyl)hexan-2-yl)-2,3,4,9-tetrahydro-1*H*-carbazole (3w)

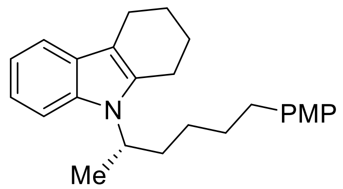

3w

Prepared according to **GP1** with *N*-alkenyl indole **1i** (21.1 mg, 0.10 mmol, 1.0 equiv.) and **2c** (55.2 mg, 0.20 mmol, 2.0 equiv.). Flash column chromatography (SiO_2, 50:1 PE/EtOAc) afforded the desired product **3w** as a colorless oil (26.1 mg, 72%).

^{1}H NMR (500 MHz, $CDCl_3$) δ 7.50 (d, J = 7.4 Hz, 1H), 7.42 (d, J = 7.2 Hz, 1H), 7.14-7.06 (m, 2H), 7.02 (d, J = 8.6 Hz, 2H), 6.83-6.80 (m, 2H), 4.35 (s, 1H), 3.80 (s, 3H), 2.77-2.72 (m, 4H), 2.53-2.42 (m, 2H), 2.18 (s, 1H), 1.99-1.83 (m, 5H), 1.58-1.49 (m, 5H), 1.34-1.25 (m, 1H), 1.19-1.10 (m, 1H).

^{13}C NMR (151 MHz, CDCl$_3$) δ 157.6, 135.3, 134.5, 129.2, 120.0, 118.2, 117.8, 113.6, 55.2, 51.1, 35.5, 34.7, 31.5, 26.6, 23.6, 23.1, 21.1, 20.4.

HRMS (ESI) m/z: $[M + H]^+$ Calcd for $C_{25}H_{32}NO^+$ 362.2478; Found 362.2483.

HPLC: 96:4 er determined by analytical HPLC, Daicel CHIRALCEL® OD-3 column, 25 °C, Hexane:*i*-PrOH = 98:2, 1.0 mL/min, 254 nm, t_{major} = 6.8 min, t_{minor} = 8.0 min.

$[\alpha]_D^{20}$ = +18.6 (*c* = 0.69, CHCl$_3$).

(*S*)-9-(6-(4-methoxyphenyl)hexan-2-yl)-9*H*-carbazole (3x)

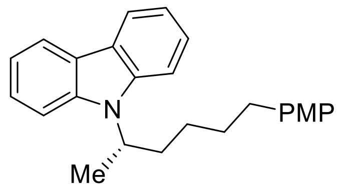

3x

Prepared according to **GP1** with *N*-alkenyl carbazole **1h** (20.7 mg, 0.10 mmol, 1.0 equiv.) and **2c** (55.2 mg, 0.20 mmol, 2.0 equiv.). Flash column chromatography (SiO$_2$, 50:1 PE/EtOAc) afforded the desired product **3x** as a colorless oil (20.7 mg, 58%).

^{1}H NMR (500 MHz, CDCl$_3$) δ 8.14 (d, *J* = 7.7 Hz, 2H), 7.51 (d, *J* = 6.3 Hz, 2H), 7.45 (t, *J* = 7.6 Hz, 2H), 7.24 (t, *J* = 7.6 Hz, 2H), 6.96 (d, *J* = 8.5 Hz, 2H), 6.77 (d, *J* = 8.5 Hz, 2H), 4.82-4.74 (m, 1H), 3.78 (s, 3H), 2.47-2.32 (m, 3H), 2.03-1.95 (m, 1H), 1.69 (d, *J* = 7.0 Hz, 3H), 1.61-1.56 (m, 1H), 1.55-1.49 (m, 1H), 1.36-1.27 (m, 1H), 1.19-1.10 (m, 1H).

^{13}C NMR (126 MHz, CDCl$_3$) δ 157.6, 139.9, 134.5, 129.1, 125.3, 123.2, 120.3, 118.5, 113.6, 110.0, 55.2, 51.2, 34.74, 34.68, 31.4, 26.5, 19.4.

HRMS (ESI) m/z: $[M + H]^+$ Calcd for $C_{25}H_{28}NO^+$ 358.2165; Found 358.2171.

HPLC: 95.5:4.5 er determined by analytical HPLC, Daicel CHIRALPAK® AD-H column, 25 °C, Hexane:*i*-PrOH = 95:5, 1.0 mL/min, 254 nm, t_{major} = 7.1 min, t_{minor} = 7.5 min.

$[\alpha]_D^{20}$ = -6.3 (*c* = 0.83, CHCl$_3$).

(*S*)-2,6-di(9*H*-carbazol-9-yl)hexan-1-ol (3y)[12]

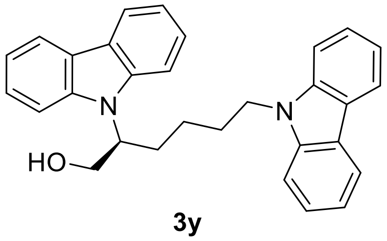

3y

Prepared according to **GP1** with *N*-alkenyl carbazole **1o** (33.8 mg, 0.10 mmol, 1.0 equiv.), **2v** (67.0 mg, 0.20 mmol, 2.0 equiv.) and *ent*-**L6** instead of **L6**. At the end of the reaction, the crude product was obtained by filtration through a short silica gel column and concentrated under reduced pressure. The crude product was dissolved in 1.0 mL THF, followed by slow dropwise addition of 1.0 mL TBAF (1.0 M in THF), stirred for 2 hours. After the reaction was completed, the reaction mixture was diluted with saturated NH_4Cl (aq., 1.0 mL) and EtOAc (2.0 mL). The aqueous phase was extracted with EtOAc (2 x 2.0 mL) and the combined organic phases were concentrated in vacuo. Flash column chromatography (SiO_2, 4:1 PE/EtOAc) afforded the desired product **3y** as a white solid (29.8 mg, 69%, over two steps).

^{1}H NMR (400 MHz, $CDCl_3$) δ 8.11-8.04 (m, 4H), 7.40-7.34 (m, 6H), 7.24-7.14 (m, 6H), 4.69-4.61 (m, 1H), 4.25 (dd, *J* = 11.4, 9.1 Hz, 1H), 4.12-3.97 (m, 2H), 3.89 (dd, *J* = 11.1, 4.6 Hz, 1H), 2.32-2.22 (m, 1H), 1.90-1.54 (m, 4H), 1.25-1.11 (m, 2H).

^{13}C NMR (101 MHz, $CDCl_3$) δ 140.2, 125.6, 122.7, 120.3, 119.2, 118.8, 108.5, 63.5, 58.2, 42.6, 28.9, 28.5, 24.3.

HRMS (ESI) m/z: $[M + H]^+$ Calcd for $C_{30}H_{29}N_2O^+$ 433.2274; Found 433.2279.

HPLC: 6:94 er determined by analytical HPLC, Daicel CHIRALPAK® IB-3 column, 25 °C, Hexane:*i*-PrOH = 80:20, 1.0 mL/min, 254 nm, t_{minor} = 22.4 min, t_{major} = 30.1 min.

$[\alpha]_D^{21}$ = +9.9 (*c* = 0.28, CH_2Cl_2).

(*R*)-4-(1*H*-pyrrol-1-yl)pentan-1-ol (3z)[13]

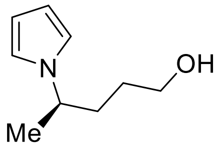

3z

Prepared according to **GP1** with *N*-alkenyl pyrrole **1p** (10.7 mg, 0.10 mmol, 1.0 equiv.), **2w** (57.2 mg, 0.20 mmol, 2.0 equiv.) and *ent*-**L6** instead of **L6**. At the end of the reaction,

the crude product was obtained by filtration through a short silica gel column and concentrated under reduced pressure. The crude product was dissolved in 1.0 mL THF, followed by slow dropwise addition of 1.0 mL TBAF (1.0 M in THF), stirred for 2 hours. After the reaction was completed, the reaction mixture was diluted with saturated NH_4Cl (aq., 1.0 mL) and EtOAc (2.0 mL). The aqueous phase was extracted with EtOAc (2 x 2.0 mL) and the combined organic phases were concentrated in vacuo. Flash column chromatography (SiO_2, 5:1 PE/EtOAc) afforded the desired product **3z** as a colorless oil (8.3 mg, 54%, over two steps).

^{1}H NMR (400 MHz, $CDCl_3$) δ 6.70 (t, J = 2.1 Hz, 2H), 6.14 (t, J = 2.1 Hz, 2H), 4.09-4.01 (m, 1H), 3.62-3.53 (m, 2H), 1.80 (q, J = 7.6 Hz, 2H), 1.47 (d, J = 6.8 Hz, 3H), 1.39-1.30 (m, 2H).

^{13}C NMR (101 MHz, $CDCl_3$) δ 118.4, 107.6, 62.4, 55.4, 34.5, 29.5, 22.4.

HRMS (ESI) m/z: $[M + H]^+$ Calcd for $C_9H_{16}NO^+$ 154.1226; Found 154.1229.

HPLC: 91:9 er determined by analytical HPLC, Daicel CHIRALCEL® OD-H column, 25 °C, Hexane:*i*-PrOH = 85:15, 1.0 mL/min, 254 nm, t_{major} = 6.0 min, t_{minor} = 7.4 min.

$[\alpha]_D^{20}$ =-12.3 (c = 0.18, MeOH).

(*R*)-3-(1*H*-pyrrol-1-yl)hexan-1-ol (3aa)

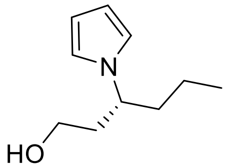

3aa

Prepared according to **GP1** with *N*-alkenyl pyrrole **1q** (25.1 mg, 0.10 mmol, 1.0 equiv.) and **2p** (47.1 mg, 0.30 mmol, 3.0 equiv.). At the end of the reaction, the crude product was obtained by filtration through a short silica gel column and concentrated under reduced pressure. The crude product was dissolved in 1.0 mL THF, followed by slow dropwise addition of 1.0 mL TBAF (1.0 M in THF), stirred for 2 hours. After the reaction was completed, the reaction mixture was diluted with saturated NH_4Cl (aq., 1.0 mL) and EtOAc (2.0 mL). The aqueous phase was extracted with EtOAc (2 x 2.0 mL) and the combined organic phases were concentrated in vacuo. Flash column chromatography (SiO_2, 5:1 PE/EtOAc) afforded the desired product **3aa** as a colorless

oil (11.4 mg, 68%, over two steps).

^{1}H NMR (400 MHz, Acetone-*d$_6$*) δ 6.71 (t, *J* = 2.1 Hz, 2H), 5.99 (t, *J* = 2.1 Hz, 2H), 4.17-4.09 (m, 1H), 3.53-3.51 (m, 1H), 3.44-3.38 (m, 1H), 3.30-3.23 (m, 1H), 1.94-1.87 (m, 2H), 1.80-1.66 (m, 2H), 1.23-1.04 (m, 2H), 0.85 (t, *J* = 7.37 Hz, 3H).

^{13}C NMR (101 MHz, Acetone-*d$_6$*) δ 118.7, 107.2, 58.1, 56.2, 39.6, 38.5, 19.2, 13.2.

HRMS (ESI) m/z: $[M + H]^+$ Calcd for $C_{10}H_{18}NO^+$ 168.1383; Found 168.1381.

HPLC: 93:7 er determined by analytical HPLC, Daicel CHIRALCEL® OD-H column, 25 °C, Hexane:*i*-PrOH = 90:10, 1.0 mL/min, 220 nm, t_{major} = 5.8 min, t_{minor} = 6.9 min.

$[\alpha]_D^{20}$ =-12.7 (*c* = 0.5, $CHCl_3$).

(*S*)-1-(6-(4-methoxyphenyl)hexan-2-yl)-2-methyl-1*H*-pyrrole (3bb)

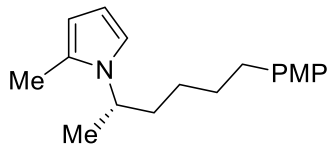

3bb

Prepared according to **GP1** with *N*-alkenyl pyrrole **1g** (12.1 mg, 0.10 mmol, 1.0 equiv.) and **2c** (55.2 mg, 0.20 mmol, 2.0 equiv.). Flash column chromatography (SiO_2, 100:1 PE/EtOAc) afforded the desired product **3bb** as a yellow oil (15.4 mg, 57%).

^{1}H NMR (400 MHz, $CDCl_3$) δ 7.06 (d, *J* = 8.6 Hz, 2H), 6.82 (d, *J* = 8.6 Hz, 2H), 6.64-6.63 (m, 1H), 6.09 (t, *J* = 3.0 Hz, 1H), 5.85 (s, 1H), 4.08-3.99 (m, 1H), 3.79 (s, 3H), 2.57-2.46 (m, 2H), 2.22 (s, 3H), 1.82-1.65 (m, 2H), 1.60-1.54 (m, 2H), 1.37 (d, *J* = 6.7 Hz, 3H), 1.32-1.18 (m, 2H).

^{13}C NMR (101 MHz, $CDCl_3$) δ 157.6, 134.5, 129.2, 128.0, 115.4, 113.7, 106.8, 105.8, 55.2, 51.2, 37.8, 34.8, 31.6, 26.1, 22.1, 12.3.

HRMS (ESI) m/z: $[M + H]^+$ Calcd for $C_{18}H_{26}NO^+$ 272.2009; Found 272.2008.

HPLC: 96:4 er determined by analytical HPLC, Daicel CHIRALPAK® IJ-3 column, 25 °C, Hexane:*i*-PrOH = 98:2, 1.0 mL/min, 220 nm, t_{major} = 19.5 min, t_{minor} = 23.4 min.

$[\alpha]_D^{20}$ = +13.4 (*c* = 0.42, $CHCl_3$).

(*R*)-1-(8-(4-methoxyphenyl)-1-phenyloctan-4-yl)-1*H*-indole (3cc)

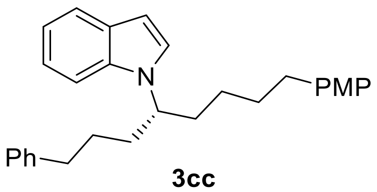

3cc

Prepared according to **GP1** with *N*-alkenyl indole **1m** (26.1 mg, 0.10 mmol, 1.0 equiv.) and **2c** (55.2 mg, 0.20 mmol, 2.0 equiv.). Flash column chromatography (SiO_2, 50:1 PE/EtOAc) afforded the desired product **3cc** as a colorless oil (33.0 mg, 80%).

^{1}H NMR (500 MHz, $CDCl_3$) δ 7.63 (d, *J* = 7.9 Hz, 1H), 7.33 (d, *J* = 8.3 Hz, 1H), 7.24-7.20 (m, 2H), 7.19-7.13 (m, 2H), 7.10-7.07 (m, 2H), 7.03 (d, *J* = 7.5 Hz, 2H), 6.96 (d, *J* = 8.4 Hz, 2H), 6.77 (d, *J* = 8.4 Hz, 2H), 6.53 (d, *J* = 3.0 Hz, 1H), 4.30-4.25 (m, 1H), 3.76 (s, 3H), 2.57-2.47 (m, 2H), 2.45-2.35 (m, 2H), 1.93-1.77 (m, 4H), 1.53-1.34 (m, 4H), 1.24-1.14 (m, 1H), 1.13-1.04 (m, 1H).

^{13}C NMR (126 MHz, $CDCl_3$) δ 157.6, 141.8, 136.6, 134.5, 129.1, 128.4, 128.33, 128.26, 125.8, 124.3, 121.2, 120.9, 119.1, 113.7, 109.5, 101.6, 56.3, 55.2, 35.8, 35.5, 35.4, 34.7, 31.4, 27.9, 25.9.

HRMS (ESI) m/z: $[M + H]^+$ Calcd for $C_{29}H_{34}NO^+$ 412.2635; Found 412.2633.

HPLC: 95:5 er determined by analytical HPLC, Daicel CHIRALCEL® OD-H column, 25 °C, Hexane:*i*-PrOH = 90:10, 1.0 mL/min, 254 nm, t_{major} = 11.7 min, t_{minor} = 14.8 min.

$[\alpha]_D^{20}$ = +0.9 (*c* = 1.3, $CHCl_3$).

(*S*)-1-(1-(4-methoxyphenyl)nonan-5-yl)-1*H*-indole (3dd)

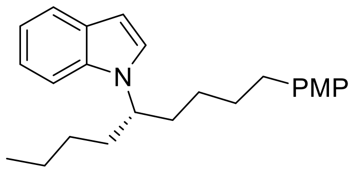

3dd

Prepared according to **GP1** with *N*-alkenyl indole **1k** (19.9 mg, 0.10 mmol, 1.0 equiv.) and **2c** (55.2 mg, 0.20 mmol, 2.0 equiv.). Flash column chromatography (SiO_2, 80:1 PE/EtOAc) afforded the desired product **3dd** as a colorless oil (31.2 mg, 89%).

^{1}H NMR (500 MHz, $CDCl_3$) δ 7.65 (d, *J* = 7.8 Hz, 1H), 7.37 (d, *J* = 8.2 Hz, 1H), 7.21-7.18 (m, 1H), 7.14-7.09 (m, 2H), 7.00 (d, *J* = 8.6 Hz, 2H), 6.79 (d, *J* = 8.6 Hz, 2H), 6.55 (d, *J* = 3.1 Hz, 1H), 4.28 (tt, *J* = 9.3, 5.2 Hz, 1H), 3.79 (s, 3H), 2.49-2.38 (m, 2H), 1.95-

1.79 (m, 4H), 1.61-1.56 (m, 1H), 1.54-1.46 (m, 1H), 1.33-1.10 (m, 5H), 1.09-1.04 (m, 1H), 0.81 (t, *J* = 7.3 Hz, 3H).

^{13}C NMR (126 MHz, CDCl$_3$) δ 157.6, 136.6, 134.5, 129.1, 128.3, 124.3, 121.1, 120.9, 119.0, 113.7, 109.5, 101.4, 56.4, 55.2, 35.8, 35.6, 34.7, 31.5, 28.5, 26.0, 22.4, 13.9.

HRMS (ESI) m/z: $[M + H]^+$ Calcd for $C_{24}H_{32}NO^+$ 350.2478; Found 350.2476.

HPLC: 96.5:3.5 er determined by analytical HPLC, Daicel CHIRALCEL® OD-H column, 25 °C, Hexane:*i*-PrOH = 97:3, 1.0 mL/min, 254 nm, t_{major} = 7.7 min, t_{minor} = 11.1 min.

$[\alpha]_D^{20}$ = -0.8 (*c* = 1.6, CHCl$_3$).

(*R*)-*N*-(2-(1*H*-indol-1-yl)-6-(4-methoxyphenyl)hexyl)-*N*-methylaniline (3ee)

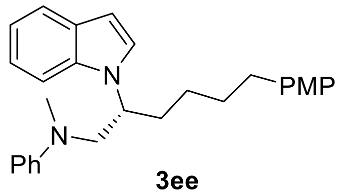

3ee

Prepared according to **GP1** with *N*-alkenyl indole **1j** (26.2 mg, 0.10 mmol, 1.0 equiv.) and **2c** (55.2 mg, 0.20 mmol, 2.0 equiv.). Flash column chromatography (SiO$_2$, 30:1 PE/EtOAc) afforded the desired product **3ee** as a colorless oil (28.9 mg, 70%).

^{1}H NMR (600 MHz, CDCl$_3$) δ 7.63-7.62 (m, 1H), 7.26-7.23 (m, 2H), 7.12-7.06 (m, 4H), 6.98 (d, *J* = 8.6 Hz, 2H), 6.79-6.77 (m, 2H), 6.74 (t, *J* = 7.3 Hz, 1H), 6.60-6.57 (m, 3H), 4.63-4.59 (m, 1H), 3.86 (dd, *J* = 15.2, 5.0 Hz, 1H), 3.77 (s, 3H), 3.51 (dd, *J* = 15.2, 8.3 Hz, 1H), 2.49-2.39 (m, 5H), 2.05-1.91 (m, 2H), 1.61-1.51 (m, 2H), 1.30-1.19 (m, 2H).

^{13}C NMR (151 MHz, CDCl$_3$) δ 157.7, 148.3, 136.5, 134.3, 129.3, 129.1, 128.5, 124.3, 121.4, 120.8, 119.3, 116.4, 113.7, 111.7, 109.7, 102.2, 57.8, 55.2, 54.5, 38.8, 34.7, 32.5, 31.4, 25.7.

HRMS (ESI) m/z: $[M + H]^+$ Calcd for $C_{28}H_{33}N_2O^+$ 413.2587; Found 413.2593.

HPLC: 94:6 er determined by analytical HPLC, Daicel CHIRALPAK® IB-3 column, 25 °C, Hexane:*i*-PrOH = 95:5, 1.0 mL/min, 254 nm, t_{major} = 12.6 min, t_{minor} = 16.1 min.

$[\alpha]_D^{20}$ = -52.6 (*c* = 1.0, CHCl$_3$).

(*R*)-1-(1-cyclohexyl-6-phenylhexan-2-yl)-1*H*-indole (3ff)

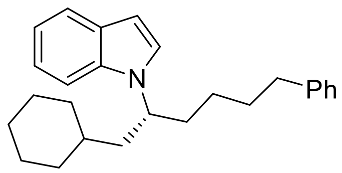

3ff

Prepared according to **GP1** with *N*-alkenyl indole **1l** (23.9 mg, 0.10 mmol, 1.0 equiv.) and **2a** (49.2 mg, 0.20 mmol, 2.0 equiv.). Flash column chromatography (SiO_2, PE) afforded the desired product **3ff** as a colorless oil (28.8 mg, 80%).

^{1}H NMR (600 MHz, $CDCl_3$) δ 7.63 (d, *J* = 7.9 Hz, 1H), 7.35 (d, *J* = 8.3 Hz, 1H), 7.22 (t, *J* = 7.5 Hz, 2H), 7.19-7.08 (m, 4H), 7.07-7.03 (m, 2H), 6.53 (d, *J* = 3.1 Hz, 1H), 4.42-4.38 (m, 1H), 2.50-2.41 (m, 2H), 1.87-1.77 (m, 4H), 1.64-1.48 (m, 7H), 1.25-1.17 (m, 1H), 1.11-1.00 (m, 5H), 0.93-0.84 (m, 2H).

^{13}C NMR (151 MHz, $CDCl_3$) δ 142.4, 136.5, 128.4, 128.3, 128.2, 125.6, 124.4, 121.1, 120.9, 119.0, 109.5, 101.5, 53.6, 43.6, 36.3, 35.7, 34.2, 33.8, 33.0, 31.3, 26.4, 26.1, 26.0, 25.9.

HRMS (ESI) m/z: $[M + H]^+$ Calcd for $C_{26}H_{34}N^+$ 360.2686; Found 360.2684.

HPLC: 92.5:7.5 er determined by analytical HPLC, Daicel CHIRALCEL® OD-H column, 25 °C, Hexane:*i*-PrOH = 99:1, 1.0 mL/min, 254 nm, t_{major} = 13.0 min, t_{minor} = 14.3 min.

$[\alpha]_D^{20}$ = -4.2 (*c* = 1.1, $CHCl_3$).

(*S*)-1-(1,5-diphenylpentyl)-1*H*-indole (3gg)

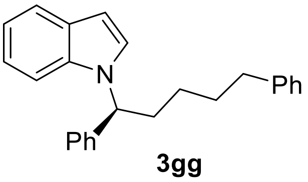

3gg

Prepared according to **GP1** with *N*-alkenyl indole **1a'** (21.9 mg, 0.10 mmol, 1.0 equiv.), **2a** (49.2 mg, 0.20 mmol, 2.0 equiv.) and *ent*-**L14** instead of **L6**. Flash column chromatography (SiO_2, PE) afforded the desired product **3gg** as a colorless oil (10.5 mg, 31%).

^{1}H NMR (500 MHz, $CDCl_3$) δ 7.62 (d, *J* = 7.8 Hz, 1H), 7.30-7.23 (m, 6H), 7.22 (d, *J* = 7.2 Hz, 1H), 7.16 (t, *J* = 7.5 Hz, 3H), 7.10 (dt, *J* = 20.7, 7.2 Hz, 4H), 6.56 (d, *J* = 2.7

Hz, 1H), 5.44 (d, J = 7.6 Hz, 1H), 2.60-2.51 (m, 2H), 2.40-2.23 (m, 2H), 1.70-1.65 (m, 2H), 1.42-1.36 (m, 2H).

^{13}C NMR (126 MHz, $CDCl_3$) δ 142.2, 141.7, 136.3, 128.6, 128.31, 128.28, 127.4, 126.3, 125.7, 125.6, 124.8, 121.4, 120.8, 119.5, 109.8, 101.7, 59.6, 35.6, 35.3, 31.2, 26.4.

HRMS (ESI) m/z: $[M + H]^+$ Calcd for $C_{25}H_{26}N^+$ 340.2060; Found 340.2062.

HPLC: 92:8 er determined by analytical HPLC, Daicel CHIRALPAK® IB-3 column, 25 °C, Hexane:*i*-PrOH = 98:2, 1.0 mL/min, 254 nm, t_{major} = 7.3 min, t_{minor} = 9.3 min.

$[\alpha]_D^{20}$ = -21.3 (c = 1.2, $CHCl_3$).

(*S*)-5-(1*H*-indol-1-yl)hexyl 2-(11-oxo-6,11-dihydrodibenzo[*b,e*]oxepin-2-yl)acetate (3hh)

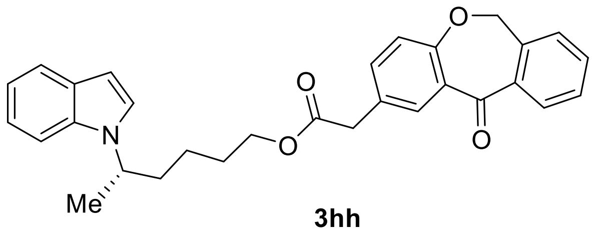

3hh

Prepared according to **GP1** with *N*-alkenyl indole **1a** (15.7 mg, 0.10 mmol, 1.0 equiv.) and **2r** (87.2 mg, 0.20 mmol, 2.0 equiv.). Flash column chromatography (SiO_2, 10:1 PE/EtOAc) afforded the desired product **3hh** as a colorless oil (26.7 mg, 57%).

^{1}H NMR (500 MHz, $CDCl_3$) δ 8.11 (d, J = 2.3 Hz, 1H), 7.90 (d, J = 7.7 Hz, 1H), 7.62 (d, J = 7.9 Hz, 1H), 7.56 (td, J = 7.4, 1.1 Hz, 1H), 7.47 (t, J = 8.0 Hz, 1H), 7.37-7.35 (m, 3H), 7.19-7.15 (m, 2H), 7.08 (t, J = 7.4 Hz, 1H), 7.01 (d, J = 8.4 Hz, 1H), 6.52 (d, J = 3.2 Hz, 1H), 5.18 (s, 2H), 4.47 (dq, J = 13.3, 6.7 Hz, 1H), 4.06-3.99 (m, 2H), 3.56 (s, 2H), 1.96-1.88 (m, 1H), 1.85-1.78 (m, 1H), 1.63-1.56 (m, 2H), 1.49 (d, J = 6.8 Hz, 3H), 1.33-1.25 (m, 1H), 1.24-1.15 (m, 1H).

^{13}C NMR (126 MHz, $CDCl_3$) δ 190.7, 171.3, 160.4, 140.4, 136.3, 135.9, 135.6, 132.7, 132.4, 129.5, 129.2, 128.5, 127.9, 127.8, 125.2, 123.9, 121.2, 121.0, 120.9, 119.2, 109.4, 101.5, 73.6, 64.5, 51.4, 40.2, 36.6, 28.3, 22.7, 21.3.

HRMS (ESI) m/z: $[M + H]^+$ Calcd for $C_{30}H_{30}NO_4^+$ 468.2169; Found 468.2166.

HPLC: 4:96 er determined by analytical HPLC, Daicel CHIRALPAK® AD-H column,

25 °C, Hexane:*i*-PrOH = 80:20, 1.0 mL/min, 254 nm, t_{minor} = 24.0 min, t_{major} = 26.9 min.

[*α*]$_D^{20}$ = -9.3 (*c* = 0.29, $CHCl_3$).

(*S*)-5-(1*H*-indol-1-yl)hexyl 4-(*N,N*-dipropylsulfamoyl)benzoate (3ii)

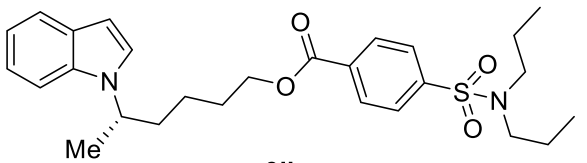

3ii

Prepared according to **GP1** with *N*-alkenyl indole **1a** (15.7 mg, 0.10 mmol, 1.0 equiv.) and **2t** (90.6 mg, 0.20 mmol, 2.0 equiv.). Flash column chromatography (SiO_2, 10:1 PE/EtOAc) afforded the desired product **3ii** as a colorless oil (42.2 mg, 87%).

^{1}H NMR (500 MHz, CDCl$_3$) δ 8.02 (d, *J* = 8.5 Hz, 2H), 7.84 (d, *J* = 8.6 Hz, 2H), 7.63 (d, *J* = 7.8 Hz, 1H), 7.38 (d, *J* = 8.2 Hz, 1H), 7.20-7.17 (m, 2H), 7.09 (t, *J* = 7.8 Hz, 1H), 6.53 (d, *J* = 3.1 Hz, 1H), 4.57-4.50 (m, 1H), 4.30-4.22 (m, 2H), 3.12-3.09 (m, 4H), 2.04-1.97 (m, 1H), 1.94-1.87 (m, 1H), 1.82-1.69 (m, 2H), 1.60-1.53 (m, 7H), 1.44-1.35 (m, 1H), 1.34-1.26 (m, 1H), 0.88 (t, *J* = 7.4 Hz, 6H).

^{13}C NMR (126 MHz, CDCl$_3$) δ 165.2, 144.1, 135.9, 133.5, 130.1, 128.4, 126.9, 123.8, 121.2, 121.0, 119.2, 109.3, 101.6, 65.1, 51.3, 49.9, 36.7, 28.3, 22.8, 21.9, 21.4, 11.1.

HRMS (ESI) m/z: $[M + H]^+$ Calcd for $C_{27}H_{37}N_2O_4S^+$ 485.2469; Found 485.2470.

HPLC: 5:95 er determined by analytical HPLC, Daicel CHIRALPAK® AD-H column, 25 °C, Hexane:*i*-PrOH = 85:15, 1.0 mL/min, 254 nm, t_{minor} = 10.3 min, t_{major} = 16.3 min.

[*α*]$_D^{20}$ = -14.9 (*c* = 0.81, $CHCl_3$).

(*S*)-5-(1*H*-indol-1-yl)hexyl 2-((1-benzyl-1*H*-indazol-3-yl)oxy)acetate (3jj)

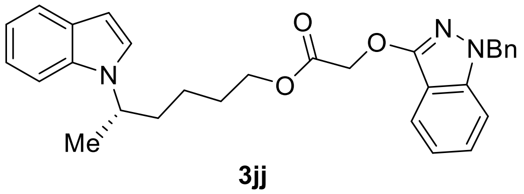

3jj

Prepared according to **GP1** with *N*-alkenyl indole **1a** (15.7 mg, 0.10 mmol, 1.0 equiv.) and **2s** (90.0 mg, 0.20 mmol, 2.0 equiv.). Flash column chromatography (SiO_2, 15:1 PE/EtOAc) afforded the desired product 3**jj** as a colorless oil (41.5 mg, 86%).

^{1}H NMR (500 MHz, CDCl$_3$) δ 7.72 (d, *J* = 8.1 Hz, 1H), 7.61 (d, *J* = 7.9 Hz, 1H), 7.32-7.30 (m, 2H), 7.26-7.16 (m, 5H), 7.12-7.04 (m, 5H), 6.50 (d, *J* = 3.1 Hz, 1H), 5.33 (s,

2H), 4.90 (s, 2H), 4.40-4.34 (m, 1H), 4.11-4.03 (m, 2H), 1.89-1.81 (m, 1H), 1.77-1.70 (m, 1H), 1.57-1.51 (m, 2H), 1.43 (d, *J* = 6.8 Hz, 3H), 1.26-1.18 (m, 1H), 1.17-1.08 (m, 1H).

^{13}C NMR (126 MHz, CDCl$_3$) δ 168.9, 154.8, 141.7, 137.3, 135.8, 128.5, 128.4, 127.5, 127.49, 127.0, 123.9, 121.2, 121.0, 120.1, 119.4, 119.2, 112.5, 109.4, 108.9, 101.5, 65.5, 64.6, 52.3, 51.3, 36.5, 28.2, 22.6, 21.2.

HRMS (ESI) m/z: $[M + H]^+$ Calcd for $C_{30}H_{32}N_3O_3^+$ 482.2438; Found 482.2434.

HPLC: 6:94 er determined by analytical HPLC, Daicel CHIRALPAK® AD-H column, 25 °C, Hexane:*i*-PrOH = 90:10, 1.0 mL/min, 254 nm, t_{minor} = 16.1 min, t_{major} = 17.5 min.

$[\alpha]_D^{20}$ = -2.5 (*c* = 1.7, CHCl$_3$).

(*S*)-5-(1*H*-indol-1-yl)hexyl 2-(1-(4-chlorobenzoyl)-5-methoxy-2-methyl-1*H*-indol-3-yl)acetate (3kk)

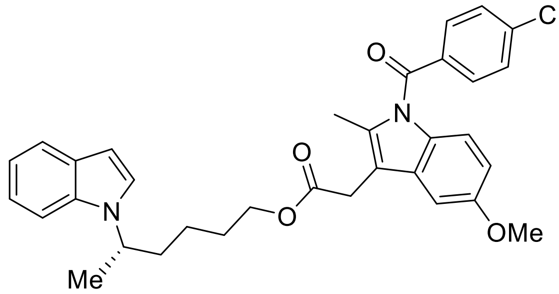

3kk

Prepared according to **GP1** with *N*-alkenyl indole **1a** (15.7 mg, 0.10 mmol, 1.0 equiv.) and **2u** (105.2 mg, 0.20 mmol, 2.0 equiv.). Flash column chromatography (SiO_2, 8:1 PE/EtOAc) afforded the desired product **3kk** as a colorless oil (29.0 mg, 52%).

^{1}H NMR (500 MHz, CDCl$_3$) δ 7.66-7.61 (m, 3H), 7.47 (d, *J* = 8.5 Hz, 2H), 7.33 (d, *J* = 8.3 Hz, 1H), 7.18 (t, *J* = 7.1 Hz, 1H), 7.13 (d, *J* = 3.2 Hz, 1H), 7.08 (t, *J* = 7.4 Hz, 1H), 6.95 (d, *J* = 2.5 Hz, 1H), 6.89 (d, *J* = 9.0 Hz, 1H), 6.68 (dd, *J* = 9.0, 2.5 Hz, 1H), 6.51 (d, *J* = 3.2 Hz, 1H), 4.45-4.38 (m, 1H), 4.07-3.98 (m, 2H), 3.82 (s, 3H), 3.60 (s, 2H), 2.34 (s, 3H), 1.93-1.85 (m, 1H), 1.81-1.74 (m, 1H), 1.62-1.59 (m, 1H), 1.58-1.55 (m, 1H), 1.47 (d, *J* = 6.8 Hz, 3H), 1.25-1.22 (m, 1H), 1.19-1.12 (m, 1H).

^{13}C NMR (126 MHz, CDCl$_3$) δ 170.8, 168.2, 156.0, 139.2, 135.88, 135.85, 133.9, 131.1, 130.8, 130.7, 129.1, 128.4, 123.8, 121.2, 121.0, 119.2, 114.9, 112.6, 111.5, 109.3, 101.51, 101.47, 64.6, 55.7, 51.4, 36.6, 30.4, 28.3, 22.7, 21.2, 13.3.

HRMS (ESI) m/z: $[M + H]^+$ Calcd for $C_{33}H_{34}ClN_2O_4^+$ 557.2202; Found 557.2198.

HPLC: 5:95 er determined by analytical HPLC, Daicel CHIRALPAK® AD-H column, 25 °C, Hexane:*i*-PrOH = 70:30, 1.0 mL/min, 254 nm, t_{minor} = 13.5 min, t_{minor} = 14.4 min.

$[\alpha]_D^{20}$ = -7.5 (*c* = 0.96, $CHCl_3$).

methyl (*S*)-6-bromo-1-(*sec*-butyl)-1*H*-indole-4-carboxylate (3ll)[14]

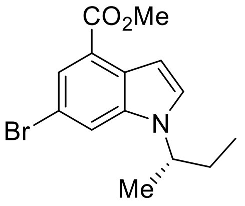

3ll

Prepared according to **GP1** with *N*-alkenyl indole **1n** (29.3 mg, 0.10 mmol, 1.0 equiv.), **2f** (46.8 mg, 0.30 mmol, 3.0 equiv.), CoI_2 as Co-salt and **L14** instead of **L6**. Flash column chromatography (SiO_2, 20:1 PE/EtOAc) afforded the desired product **3ll** as a colorless oil (21.4 mg, 69%).

^{1}H NMR (400 MHz, $CDCl_3$) δ 7.99 (d, *J* = 1.6 Hz, 1H), 7.71 (s, 1H), 7.30 (d, *J* = 3.3 Hz, 1H), 7.13 (d, *J* = 3.2 Hz, 1H), 4.36 (m, 1H), 3.98 (s, 3H), 1.88 (m, 2H), 1.51 (d, *J* = 6.8 Hz, 3H), 0.82 (t, *J* = 7.4 Hz, 3H).

^{13}C NMR (101 MHz, $CDCl_3$) δ 166.9, 137.7, 126.9, 125.6, 122.6, 116.9, 113.7, 102.9, 53.5, 51.9, 30.0, 20.9, 10.8.

HRMS (ESI) m/z: $[M + H]^+$ Calcd for $C_{14}H_{17}BrNO_2^+$ 310.0437; Found 310.0434.

HPLC: 91.5:8.5 er determined by analytical HPLC, Daicel CHIRALPAK® ID column, 25 °C, Hexane:*i*-PrOH = 98:2, 1.0 mL/min, 254 nm, t_{major} = 8.5 min, t_{minor} = 9.4 min.

$[\alpha]_D^{20}$ = -16.1 (*c* = 0.32, $CHCl_3$).

6.2 Markovnikov hydroalkylation of alkenes

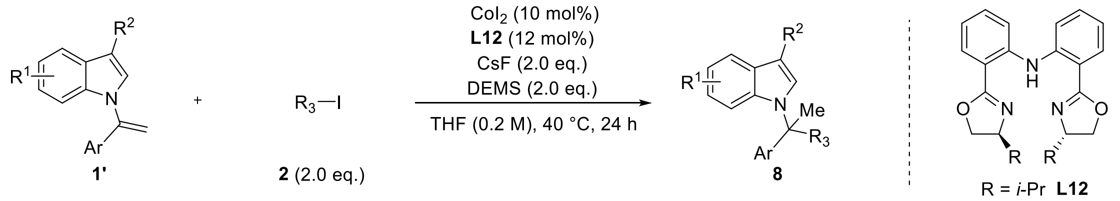

General Procedure 2 (GP2): To an oven-dried 10.0 mL Teflon-screw cap test tube containing a magnetic stir was charged with CoI_2 (3.1 mg, 10 mol%) and ligand **L12**

(4.7 mg, 12 mol%) under a Argon atmosphere using glove-box techniques. Subsequently, anhydrous THF (0.5 mL) was added, and the mixture was stirred for 10 minutes at room temperature. Then, CsF (30.4 mg, 0.20 mmol, 2.0 equiv.), *N*-alkenyl indole **1'** (0.10 mmol, 1.0 equiv), alkyl iodide **2** (0.20 mmol, 2.0 equiv.) and $(OEt)_2MeSiH$ (32 uL, 0.20 mmol, 2.0 equiv.) were sequentially added. Afterwards, the tube was sealed with airtight electrical tapes and removed from the glove box and stirred at 40 °C for 24 hours at 500 rpm. After the reaction was completed, the reaction mixture was diluted with saturated NH_4Cl (aq., 2.0 mL) and EtOAc (3.0 mL). The aqueous phase was extracted with EtOAc (2 x 3.0 mL) and the combined organic phases were concentrated in vacuo. The crude mixture was purified by flash column chromatography on silica gel using a mixture of PE/EtOAc as eluent to obtain the desired product **8**.

1- (2,5-diphenylpentan-2-yl)-1*H*-indole (8a)

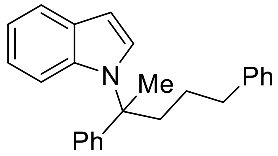

8a

Prepared according to **GP2** with *N*-alkenyl indole **1a'** (21.9 mg, 0.10 mmol, 1.0 equiv.) and **2a** (49.2 mg, 0.20 mmol, 2.0 equiv.). Flash column chromatography (SiO_2, PE) afforded the desired product **8a** as a colorless oil (26.8 mg, 79%).

^{1}H NMR (500 MHz, CDCl$_3$) δ 7.60 (d, *J* = 7.9 Hz, 1H), 7.44 (d, *J* = 3.3 Hz, 1H), 7.27-7.19 (m, 5H), 7.15-7.10 (m, 3H), 7.01-6.97 (m, 3H), 6.83 (t, *J* = 7.8 Hz, 1H), 6.58 (d, *J* = 8.4 Hz, 1H), 6.53 (d, *J* = 3.2 Hz, 1H), 2.60 (td, *J* = 12.9, 4.5 Hz, 1H), 2.51 (t, *J* = 7.5 Hz, 2H), 2.31 (td, *J* = 12.7, 4.3 Hz, 1H), 1.88 (s, 3H), 1.60-1.51 (m, 1H), 1.11-1.02 (m, 1H).

^{13}C NMR (126 MHz, CDCl$_3$) δ 146.5, 141.7, 135.2, 129.9, 128.5, 128.3, 126.9, 126.2, 125.8, 125.5, 120.71, 120.69, 119.1, 113.7, 100.6, 63.0, 39.4, 35.7, 28.4, 25.0.

HRMS (ESI) m/z: $[M + H]^+$ Calcd for $C_{25}H_{26}N^+$ 340.2060; Found 340.2059.

1-(5-(4-methoxyphenyl)-2-phenylpentan-2-yl)-1*H*-indole (8b)

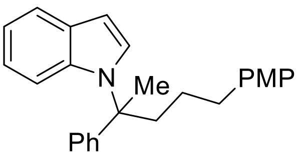

8b

Prepared according to **GP2** with *N*-alkenyl indole **1a'** (21.9 mg, 0.10 mmol, 1.0 equiv.) and **2c** (55.2 mg, 0.20 mmol, 2.0 equiv.). Flash column chromatography (SiO_2, 50:1 PE/EtOAc) afforded the desired product **8b** as a colorless oil (24.1 mg, 65%).

^{1}H NMR (400 MHz, $CDCl_3$) δ 7.60 (d, *J* = 7.9 Hz, 1H), 7.44 (d, *J* = 3.3 Hz, 1H), 7.28-7.19 (m, 3H), 7.12-7.10 (m, 2H), 6.99 (t, *J* = 7.4 Hz, 1H), 6.91 (d, *J* = 8.5 Hz, 2H), 6.82 (t, *J* = 7.7 Hz, 1H), 6.76-6.73 (m, 2H), 6.57 (d, *J* = 8.5 Hz, 1H), 6.53 (d, *J* = 3.3 Hz, 1H), 3.76 (s, 3H), 2.57 (td, *J* = 12.7, 4.4 Hz, 1H), 2.45 (t, *J* = 7.5 Hz, 2H), 2.28 (td, *J* = 12.7, 4.3 Hz, 1H), 1.87 (s, 3H), 1.57-1.46 (m, 1H), 1.07-0.96 (m, 1H).

^{13}C NMR (101 MHz, $CDCl_3$) δ 157.7, 146.5, 135.1, 133.7, 129.9, 129.1, 128.5, 126.9, 126.2, 125.5, 120.67, 120.65, 119.1, 113.7, 100.5, 63.0, 55.2, 39.3, 34.7, 28.4, 25.2.

HRMS (ESI) m/z: $[M + H]^+$ Calcd for $C_{26}H_{28}NO^+$ 370.2165; Found 370.2167.

1-(5-(4-bromophenyl)-2-phenylpentan-2-yl)-1*H*-indole (8c)

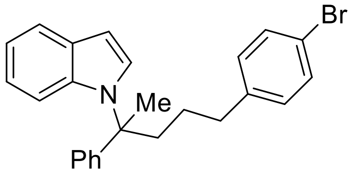

8c

Prepared according to **GP2** with *N*-alkenyl indole **1a'** (21.9 mg, 0.10 mmol, 1.0 equiv.) and **2d** (65.0 mg, 0.20 mmol, 2.0 equiv.). Flash column chromatography (SiO_2, PE) afforded the desired product **8c** as a colorless oil (27.3 mg, 65%).

^{1}H NMR (400 MHz, $CDCl_3$) δ 7.62 (d, *J* = 7.9 Hz, 1H), 7.45 (d, *J* = 3.2 Hz, 1H), 7.31-7.28 (m, 3H), 7.26-7.22 (m, 2H), 7.12 (d, *J* = 7.3 Hz, 2H), 7.01 (t, *J* = 7.4 Hz, 1H), 6.85-6.81 (m, 3H), 6.56-6.55 (m, 2H), 2.58 (td, *J* = 12.7, 4.2 Hz, 1H), 2.51-2.40 (m, 2H), 2.28 (td, *J* = 12.6, 4.3 Hz, 1H), 1.89 (s, 3H), 1.60-1.49 (m, 1H), 1.03-0.92 (m, 1H).

^{13}C NMR (101 MHz, $CDCl_3$) δ 146.4, 140.5, 135.0, 131.3, 130.0, 129.9, 128.5, 127.0, 126.2, 125.4, 120.7, 120.7, 119.5, 119.2, 113.6, 100.6, 62.9, 38.8, 34.9, 28.6, 24.7.

HRMS (ESI) m/z: $[M + H]^+$ Calcd for $C_{25}H_{25}BrN^+$ 418.1165; Found 418.1164.

1-(2-phenylbutan-2-yl)-1*H*-indole (8d)

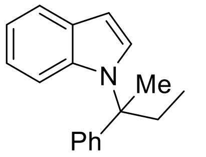

8d

Prepared according to **GP2** with *N*-alkenyl indole **1a'** (21.9 mg, 0.10 mmol, 1.0 equiv.) and **2p** (31.2 mg, 0.20 mmol, 2.0 equiv.), Ph_2SiH_2 instead of DEMS. Flash column chromatography (SiO_2, PE) afforded the desired product **8d** as a colorless oil (15.2 mg,61%).

^{1}H NMR (400 MHz, $CDCl_3$) δ 7.62 (d, *J* = 7.9 Hz, 1H), 7.52 (d, *J* = 3.3 Hz, 1H), 7.31-7.28 (m, 2H), 7.26-7.23 (m, 1H), 7.18-7.16 (m, 2H), 7.01 (t, *J* = 7.4 Hz, 1H), 6.86 (t, *J* = 7.7 Hz, 1H), 6.64 (d, *J* = 8.4 Hz, 1H), 6.56 (d, *J* = 3.2 Hz, 1H), 2.65 (dq, *J* = 14.6, 7.4 Hz, 1H), 2.35 (dt, *J* = 13.5, 7.2 Hz, 1H), 1.90 (s, 3H), 0.63 (t, *J* = 7.3 Hz, 3H).

^{13}C NMR (101 MHz, $CDCl_3$) δ 146.6, 135.1, 129.9, 128.5, 126.9, 126.5, 125.6, 120.7, 120.6, 119.1, 113.7, 100.3, 63.4, 32.4, 27.8, 8.1.

HRMS (ESI) m/z: $[M + H]^+$ Calcd for $C_{18}H_{20}N^+$ 250.1590; Found 250.1590.

1-(5-methoxy-2-phenylpentan-2-yl)-1*H*-indole (8e)

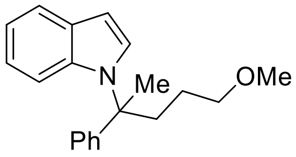

8e

Prepared according to **GP2** with *N*-alkenyl indole **1a'** (21.9 mg, 0.10 mmol, 1.0 equiv.) and **2h** (40.0 mg, 0.20 mmol, 2.0 equiv.). Flash column chromatography (SiO_2, 100:1 PE/EtOAc) afforded the desired product **8e** as a colorless oil (21.4 mg, 73%).

^{1}H NMR (500 MHz, $CDCl_3$) δ 7.60 (d, *J* = 7.9 Hz, 1H), 7.51 (d, *J* = 3.3 Hz, 1H), 7.30-7.27 (m, 2H), 7.25-7.22 (m, 1H), 7.17-7.16 (m, 2H), 7.00 (t, *J* = 7.4 Hz, 1H), 6.87-6.84 (m, 1H), 6.64 (d, *J* = 8.4 Hz, 1H), 6.55 (d, *J* = 3.1 Hz, 1H), 3.30-3.25 (m, 1H), 3.23-3.18 (m, 4H), 2.63 (td, *J* = 12.8, 4.5 Hz, 1H), 2.43 (td, *J* = 13.2, 4.3 Hz, 1H), 1.92 (s, 3H), 1.54-1.47 (m, 1H), 0.98-0.89 (m, 1H).

^{13}C NMR (126 MHz, $CDCl_3$) δ 146.5, 135.1, 130.0, 128.5, 126.9, 126.3, 125.6, 120.72, 120.69, 119.1, 113.7, 100.6, 72.4, 63.0, 58.4, 36.6, 28.4, 23.9.

HRMS (ESI) m/z: [M + H]$^+$ Calcd for $C_{20}H_{24}NO^+$ 294.1852; Found 294.1851.

1-(5-((tert-butyldimethylsilyl)oxy)-2-phenylpentan-2-yl)-1*H*-indole (8f)

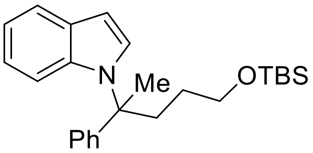

8f

Prepared according to **GP2** with *N*-alkenyl indole **1a'** (21.9 mg, 0.10 mmol, 1.0 equiv.) and **2j** (60.0 mg, 0.20 mmol, 2.0 equiv.). Flash column chromatography (SiO_2, 100:1 PE/EtOAc) afforded the desired product **8f** as a colorless oil (20.1 mg, 51%).

^{1}H NMR (500 MHz, CDCl$_3$) δ 7.60 (d, *J* = 7.9 Hz, 1H), 7.52 (d, *J* = 3.3 Hz, 1H), 7.30-7.27 (m, 2H), 7.25-7.22 (m, 1H), 7.19-7.15 (m, 2H), 7.01-6.97 (m, 1H), 6.87-6.83 (m, 1H), 6.64 (dd, *J* = 8.4, 0.6 Hz, 1H), 6.55 (dd, *J* = 3.3, 0.6 Hz, 1H), 3.54-3.46 (m, 2H), 2.60 (td, *J* = 12.8, 4.6 Hz, 1H), 2.42 (td, *J* = 13.2, 4.2 Hz, 1H), 1.92 (s, 3H), 1.50-1.39 (m, 1H), 0.96-0.86 (m, 10H), -0.02 (d, *J* = 2.6 Hz, 6H).

^{13}C NMR (126 MHz, CDCl$_3$) δ 146.6, 135.2, 134.4, 130.0, 128.5, 126.9, 126.3, 125.6, 120.7, 119.1, 113.7, 100.5, 63.0, 62.8, 36.6, 28.4, 27.1, 25.9, 18.3, -5.4.

HRMS (ESI) m/z: [M + H]$^+$ Calcd for $C_{25}H_{36}NOSi^+$ 394.2561; Found 394.2563.

1-(5-phenoxy-2-phenylpentan-2-yl)-1*H*-indole (8g)

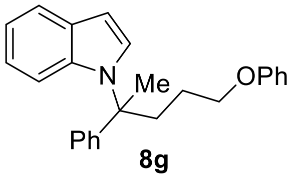

8g

Prepared according to **GP2** with *N*-alkenyl indole **1a'** (21.9 mg, 0.10 mmol, 1.0 equiv.) and **2l** (52.4 mg, 0.20 mmol, 2.0 equiv.). Flash column chromatography (SiO_2, 100:1 PE/EtOAc) afforded the desired product **8g** as a colorless oil (22.0 mg, 62%).

^{1}H NMR (400 MHz, CDCl$_3$) δ 7.62 (d, *J* = 7.9 Hz, 1H), 7.53 (d, *J* = 3.4 Hz, 1H), 7.32-7.27 (m, 3H), 7.25-7.17 (m, 4H), 7.01 (t, *J* = 7.3 Hz, 1H), 6.94-6.85 (m, 2H), 6.79 (d, *J* = 7.9 Hz, 2H), 6.65 (d, *J* = 8.4 Hz, 1H), 6.57 (d, *J* = 3.2 Hz, 1H), 3.85-3.74 (m, 2H), 2.74 (td, *J* = 12.7, 4.5 Hz, 1H), 2.57 (td, *J* = 12.6, 4.4 Hz, 1H), 1.96 (s, 3H), 1.80-1.70 (m, 1H), 1.17-1.07 (m, 1H).

^{13}C NMR (101 MHz, CDCl$_3$) δ 158.8, 146.4, 135.1, 130.0, 129.4, 128.6, 127.0, 126.3,

125.5, 120.8, 120.6, 119.2, 114.4, 113.6, 100.7, 67.5, 62.9, 36.3, 28.7, 23.8.

HRMS (ESI) m/z: $[M + H]^+$ Calcd for $C_{25}H_{26}NO^+$ 356.2009; Found 356.2010.

1-(6-(benzyloxy)-2-phenylhexan-2-yl)-1*H*-indole (8h)

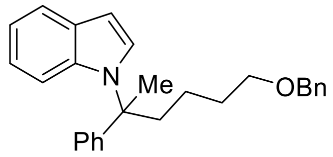

8h

Prepared according to **GP2** with *N*-alkenyl indole **1a'** (21.9 mg, 0.10 mmol, 1.0 equiv.) and **2i** (58.0 mg, 0.20 mmol, 2.0 equiv.), Ph_2SiH_2 instead of DEMS. Flash column chromatography (SiO_2, 50:1 PE/EtOAc) afforded the desired product **8h** as a colorless oil (22.2 mg, 58%).

^{1}H NMR (600 MHz, $CDCl_3$) δ 7.59 (d, *J* = 7.9 Hz, 1H), 7.47 (d, *J* = 3.4 Hz, 1H), 7.32-7.30 (m, 2H), 7.26-7.21 (m, 6H), 7.12 (d, *J* = 7.6 Hz, 2H), 6.98 (t, *J* = 7.4 Hz, 1H), 6.83 (t, *J* = 7.7 Hz, 1H), 6.61 (d, *J* = 8.4 Hz, 1H), 6.53 (d, *J* = 3.1 Hz, 1H), 4.37 (s, 2H), 3.36-3.28 (m, 2H), 2.58 (td, *J* = 12.8, 4.3 Hz, 1H), 2.30 (td, *J* = 12.7, 4.2 Hz, 1H), 1.89 (s, 3H), 1.58-1.47 (m, 2H), 1.37-1.30 (m, 1H), 0.80-0.72 (m, 1H).

^{13}C NMR (151 MHz, $CDCl_3$) δ 146.6, 138.5, 135.1, 129.9, 128.5, 128.3, 127.6, 127.5, 126.9, 126.3, 125.5, 120.7, 120.6, 119.1, 113.6, 100.5, 72.8, 69.9, 63.1, 39.7, 29.7, 28.4, 20.3.

HRMS (ESI) m/z: $[M + H]^+$ Calcd for $C_{27}H_{30}NO^+$ 384.2322; Found 384.2324.

1-(6-(4-methoxyphenyl)-2-phenylhexan-2-yl)-1*H*-indole (8i)

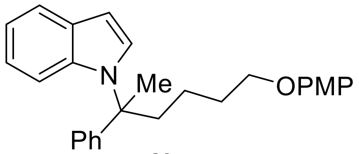

8i

Prepared according to **GP2** with *N*-alkenyl indole **1a'** (21.9 mg, 0.10 mmol, 1.0 equiv.) and **2k** (61.2 mg, 0.20 mmol, 2.0 equiv.), CoBr•DME instead of CoI_2. Flash column chromatography (SiO_2, 50:1 PE/EtOAc) afforded the desired product **8i** as a white solid (29.6 mg, 74%).

^{1}H NMR (400 MHz, $CDCl_3$) δ 7.62 (d, *J* = 7.9 Hz, 1H), 7.50 (d, *J* = 3.3 Hz, 1H), 7.31-7.27 (m, 2H), 7.24-7.22 (m, 1H), 7.16 (d, *J* = 7.2 Hz, 2H), 7.01 (t, *J* = 7.4 Hz, 1H), 6.87-

6.78 (m, 3H), 6.73 (d, J = 9.1 Hz, 2H), 6.63 (d, J = 8.4 Hz, 1H), 6.56 (d, J = 3.2 Hz, 1H), 3.81-3.70 (m, 5H), 2.65 (td, J = 12.8, 4.3 Hz, 1H), 2.37 (td, J = 12.7, 4.3 Hz, 1H), 1.93 (s, 3H), 1.76-1.61 (m, 2H), 1.49-1.38 (m, 1H), 0.87-0.76 (m, 1H).

^{13}C NMR (101 MHz, $CDCl_3$) δ 153.7, 153.0, 146.5, 135.1, 129.9, 128.5, 126.9, 126.3, 125.5, 120.72, 120.68, 119.1, 115.4, 114.5, 113.6, 100.5, 68.1, 63.1, 55.7, 39.5, 29.3, 28.5, 20.2.

HRMS (ESI) m/z: $[M + H]^+$ Calcd for $C_{27}H_{30}NO_2^+$ 400.2271; Found 400.2276.

1-(2,5-diphenylpentan-2-yl)-4-methyl-1*H*-indole (8j)

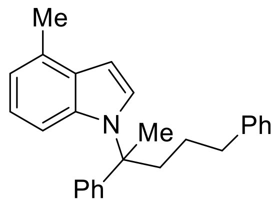

8j

Prepared according to **GP2** with *N*-alkenyl indole **1b'** (23.3 mg, 0.10 mmol, 1.0 equiv.) and **2a** (49.2 mg, 0.20 mmol, 2.0 equiv.). Flash column chromatography (SiO_2, PE) afforded the desired product **8j** as a colorless oil (24.4 mg, 69%).

^{1}H NMR (500 MHz, $CDCl_3$) δ 7.44 (d, J = 3.2 Hz, 1H), 7.28-7.21 (m, 5H), 7.16 (t, J = 7.3 Hz, 1H), 7.12-7.11 (m, 2H), 7.03 (d, J = 7.1 Hz, 2H), 6.81 (d, J = 7.0 Hz, 1H), 6.76 (t, J = 7.7 Hz, 1H), 6.56 (d, J = 2.8 Hz, 1H), 6.45 (d, J = 8.3 Hz, 1H), 2.61 (td, J = 12.8, 4.3 Hz, 1H), 2.56 (s, 3H), 2.53 (t, J = 7.5 Hz, 2H), 2.32 (td, J = 13.2, 4.2 Hz, 1H), 1.89 (s, 3H), 1.60-1.53 (m, 1H), 1.15-1.08 (m, 1H).

^{13}C NMR (126 MHz, $CDCl_3$) δ 146.6, 141.7, 134.9, 129.9, 129.8, 128.5, 128.29, 128.27, 126.9, 125.8, 125.6, 120.8, 119.4, 111.4, 99.0, 63.1, 39.6, 35.7, 28.3, 25.1, 18.8.

HRMS (ESI) m/z: $[M + H]^+$ Calcd for $C_{26}H_{28}N^+$ 354.2216; Found 354.2215.

5-chloro-1-(2,5-diphenylpentan-2-yl)-1*H*-indole (8k)

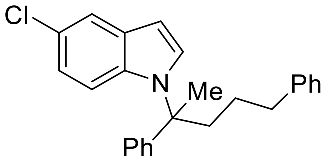

8k

Prepared according to **GP2** with *N*-alkenyl indole **1c'** (25.4 mg, 0.10 mmol, 1.0 equiv.)

and **2a** (49.2 mg, 0.20 mmol, 2.0 equiv.). Flash column chromatography (SiO_2, PE) afforded the desired product **8k** as a colorless oil (25.1 mg, 67%).

^{1}H NMR (600 MHz, $CDCl_3$) δ 7.55 (m, 1H), 7.44 (d, *J* = 3.2 Hz, 1H), 7.27-7.22 (m, 3H), 7.21-7.13 (m, 3H), 7.08 (d, *J* = 7.3 Hz, 2H), 6.98 (d, *J* = 7.3 Hz, 2H), 6.75 (dd, *J* = 8.8, 1.6 Hz, 1H), 6.46-6.44 (m, 2H), 2.56-2.46 (m, 3H), 2.31-2.27 (m, 1H), 1.86 (s, 3H), 1.59-1.51 (m, 1H), 1.04-0.96 (m, 1H).

^{13}C NMR (151 MHz, $CDCl_3$) δ 146.1, 141.5, 133.5, 130.9, 128.6, 128.3, 128.2, 127.6, 127.1, 125.8, 125.4, 125.0, 121.0, 120.0, 114.5, 100.2, 63.2, 39.1, 35.6, 28.4, 25.0.

HRMS (ESI) m/z: $[M + H]^+$ Calcd for $C_{25}H_{25}ClN^+$ 374.1670; Found 374.1672.

5-methoxy-1-(5-phenoxy-2-phenylpentan-2-yl)-1*H*-indole (8l)

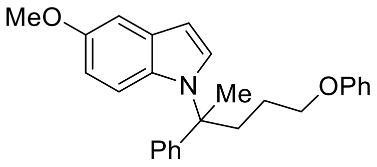

8l

Prepared according to **GP2** with *N*-alkenyl indole **1d'** (24.9 mg, 0.10 mmol, 1.0 equiv.) and **2l** (52.4 mg, 0.20 mmol, 2.0 equiv.). Flash column chromatography (SiO_2, 100:1 PE/EtOAc) afforded the desired product **8l** as a colorless oil (26.2 mg, 68%).

^{1}H NMR (500 MHz, $CDCl_3$) δ 7.46 (d, *J* = 8.6 Hz, 1H), 7.42 (d, *J* = 3.4 Hz, 1H), 7.31-7.20 (m, 7H), 6.91 (t, *J* = 7.3 Hz, 1H), 6.79 (d, *J* = 7.8 Hz, 2H), 6.68 (dd, *J* = 8.6, 2.2 Hz, 1H), 6.48 (d, *J* = 3.2 Hz, 1H), 6.08 (d, *J* = 2.0 Hz, 1H), 3.85-3.75 (m, 2H), 3.48 (s, 3H), 2.72 (td, *J* = 12.7, 4.5 Hz, 1H), 2.54 (td, *J* = 13.2, 4.4 Hz, 1H), 1.94 (s, 3H), 1.79-1.70 (m, 1H), 1.22-1.12 (m, 1H).

^{13}C NMR (126 MHz, $CDCl_3$) δ 158.8, 155.0, 146.2, 135.8, 129.4, 128.6, 127.0, 125.6, 125.2, 124.2, 121.1, 120.6, 114.4, 109.1, 100.6, 97.5, 67.5, 62.8, 55.3, 36.2, 28.4, 23.9.

HRMS (ESI) m/z: $[M + H]^+$ Calcd for $C_{26}H_{28}NO_2^+$ 386.2115; Found 386.2116.

5-(benzyloxy)-1-(5-phenoxy-2-phenylpentan-2-yl)-1*H*-indole (8m)

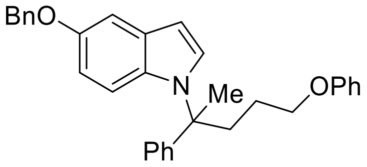

8m

Prepared according to **GP2** with *N*-alkenyl indole **1f'** (32.5 mg, 0.10 mmol, 1.0 equiv.) and **2l** (52.4 mg, 0.20 mmol, 2.0 equiv.), $CoBr_2$•DME instead of CoI_2. Flash column chromatography (SiO_2, 30:1 PE/EtOAc) afforded the desired product **8m** as a colorless oil (27.7 mg, 60%).

^{1}H NMR (500 MHz, $CDCl_3$) δ 7.48-7.35 (m, 5H), 7.31-7.22 (m, 6H), 7.18-7.14 (m, 3H), 6.91 (t, *J* = 7.3 Hz, 1H), 6.79 (d, *J* = 8.3 Hz, 2H), 6.61 (d, *J* = 9.0 Hz, 1H), 6.53 (d, *J* = 9.0 Hz, 1H), 6.47 (d, *J* = 2.9 Hz, 1H), 5.03 (s, 2H), 3.83-3.76 (m, 2H), 2.70 (td, *J* = 12.8, 4.2 Hz, 1H), 2.54 (td, J = 13.3, 4.2 Hz, 1H), 1.92 (s, 3H), 1.79-1.69 (m, 1H), 1.15-1.07 (m, 1H).

^{13}C NMR (126 MHz, $CDCl_3$) δ 158.8, 153.0, 146.5, 137.8, 130.6, 130.4, 129.4, 128.6, 128.5, 127.7, 127.5, 127.1, 126.9, 125.5, 120.6, 114.4, 114.3, 111.7, 103.7, 100.4, 70.6, 67.5, 62.9, 36.4, 28.7, 23.8.

HRMS (ESI) m/z: $[M + H]^+$ Calcd for $C_{32}H_{32}NO_2^+$ 462.2428; Found 462.2425.

1-(6-(benzyloxy)-2-phenylhexan-2-yl)-5-(4,4,5,5-tetramethyl-1,3,2-dioxaborolan-2-yl)-1*H*-indole (8n)

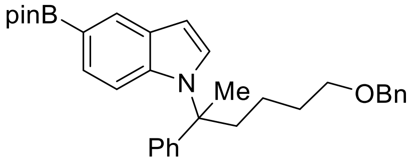

8n

Prepared according to **GP2** with *N*-alkenyl indole **1e'** (34.5 mg, 0.10 mmol, 1.0 equiv.) and **2i** (58.0 mg, 0.20 mmol, 2.0 equiv.). Flash column chromatography (SiO_2, 10:1 PE/EtOAc) afforded the desired product **8n** as a colorless oil (31.6 mg, 62%).

^{1}H NMR (500 MHz, $CDCl_3$) δ 8.14 (s, 1H), 7.46 (d, *J* = 3.3 Hz, 1H), 7.33-7.27 (m, 4H), 7.25-7.20 (m, 5H), 7.10 (d, *J* = 7.1 Hz, 2H), 6.61 (d, *J* = 8.5 Hz, 1H), 6.56 (d, *J* = 3.2 Hz, 1H), 4.38 (s, 2H), 3.37-3.27 (m, 2H), 2.59 (td, *J* = 12.8, 4.3 Hz, 1H), 2.30 (td, *J* = 12.7, 4.2 Hz, 1H), 1.90 (s, 3H), 1.55-1.46 (m, 2H), 1.38-1.31 (m, 13H), 0.80-0.71 (m, 1H).

^{13}C NMR (126 MHz, $CDCl_3$) δ 146.6, 138.5, 137.1, 129.7, 128.7, 128.5, 128.3, 127.6, 127.5, 126.9, 126.7, 126.5, 125.5, 113.1, 101.2, 83.3, 63.3, 39.6, 29.7, 28.5, 24.89,

24.85, 20.4.

HRMS (ESI) m/z: $[M + H]^+$ Calcd for $C_{33}H_{41}BNO_3^+$ 509.3210; Found 509.3211.

6-fluoro-1-(5-methoxy-2-phenylpentan-2-yl)-1*H*-indole (8o)

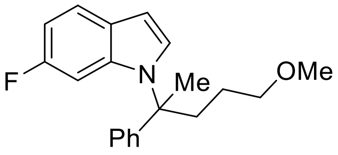

8o

Prepared according to **GP2** with *N*-alkenyl indole **1i'** (23.7 mg, 0.10 mmol, 1.0 equiv.) and **2h** (40.0 mg, 0.20 mmol, 2.0 equiv.), Ph_2SiH_2 instead of DEMS. Flash column chromatography (SiO_2, 20:1 PE/EtOAc) afforded the desired product **8o** as a colorless oil (20.2 mg, 65%).

^{1}H NMR (600 MHz, $CDCl_3$) δ 7.50-7.47 (m, 2H), 7.31-7.28 (m, 2H), 7.26-7.24 (m, 1H), 7.15 (d, J = 7.6 Hz, 2H), 6.76 (t, J = 9.0 Hz, 1H), 6.51 (s, 1H), 6.30 (d, J = 11.0 Hz, 1H), 3.29-3.20 (m, 5H), 2.57 (td, J = 12.8, 4.1 Hz, 1H), 2.40 (td, J = 12.9, 3.9 Hz, 1H), 1.90 (s, 3H), 1.53-1.46 (m, 1H), 0.97-0.90 (m, 1H).

^{13}C NMR (151 MHz, $CDCl_3$) δ 158.6 (d, J_{C-F} = 236.0 Hz), 145.8, 135.0 (d, J_{C-F} = 12.3 Hz), 128.7, 127.2, 126.8 (d, J_{C-F} = 3.5 Hz), 126.3, 125.5, 121.2 (d, J_{C-F} = 10.2 Hz), 108.0 (d, J_{C-F} = 24.8 Hz), 100.7, 99.9 (d, J_{C-F} = 27.1 Hz), 72.3, 63.1, 58.4, 36.3, 28.3, 24.0.

^{19}F NMR (376 MHz, $CDCl_3$) δ -121.43.

HRMS (ESI) m/z: $[M + H]^+$ Calcd for $C_{20}H_{23}FNO^+$ 312.1758; Found 312.1755.

1-(2,5-diphenylpentan-2-yl)-6-methyl-1*H*-indole (8p)

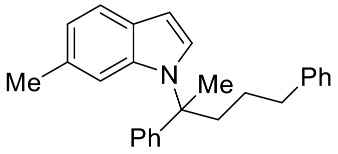

8p

Prepared according to **GP2** with *N*-alkenyl indole **1h'** (23.3 mg, 0.10 mmol, 1.0 equiv.) and **2a** (49.2 mg, 0.20 mmol, 2.0 equiv.), Ph_2SiH_2 instead of DEMS. Flash column chromatography (SiO_2, PE) afforded the desired product **8p** as a colorless oil (24.4 mg, 69%).

^{1}H NMR (500 MHz, $CDCl_3$) δ 7.48 (d, J = 7.9 Hz, 1H), 7.36 (d, J = 2.0 Hz, 1H), 7.26-

7.19 (m, 5H), 7.15-7.10 (m, 3H), 7.00 (d, *J* = 7.1 Hz, 2H), 6.83 (d, *J* = 7.8 Hz, 1H), 6.47 (d, *J* = 1.3 Hz, 1H), 6.35 (s, 1H), 2.60-2.50 (m, 3H), 2.31-2.25 (m, 1H), 2.17 (s, 3H), 1.87 (s, 3H), 1.57-1.55 (m, 1H), 1.11-1.02 (m, 1H).

^{13}C NMR (126 MHz, $CDCl_3$) δ 146.6, 141.7, 135.6, 130.1, 128.5, 128.3, 128.2, 127.8, 126.8, 125.8, 125.7, 125.5, 121.0, 120.2, 113.6, 100.3, 62.9, 39.3, 35.6, 28.4, 24.9, 21.9.

HRMS (ESI) m/z: $[M + H]^+$ Calcd for $C_{26}H_{28}N^+$ 354.2216; Found 354.2219.

5-(5-phenoxy-2-phenylpentan-2-yl)-5*H*-[1,3]dioxolo[4,5-*f*]indole (8q)

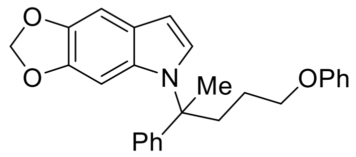

8q

Prepared according to **GP2** with *N*-alkenyl indole **1g'** (26.3 mg, 0.10 mmol, 1.0 equiv.) and **2l** (52.4 mg, 0.20 mmol, 2.0 equiv.). Flash column chromatography (SiO_2, 20:1 PE/EtOAc) afforded the desired product **8q** as a colorless oil (23.6 mg, 59%).

^{1}H NMR (600 MHz, $CDCl_3$) δ 7.39 (d, *J* = 3.0 Hz, 1H), 7.32-7.29 (m, 2H), 7.25-7.23 (m, 3H), 7.18 (d, *J* = 7.5 Hz, 2H), 6.97 (s, 1H), 6.92 (t, *J* = 7.3 Hz, 1H), 6.80 (d, *J* = 8.0 Hz, 2H), 6.43 (d, *J* = 2.6 Hz, 1H), 6.12 (s, 1H), 5.81 (d, *J* = 4.2 Hz, 2H), 3.85-3.75 (m, 2H), 2.68 (td, *J* = 12.8, 4.3 Hz, 1H), 2.52 (td, *J* = 13.0, 4.2 Hz, 1H), 1.91 (s, 3H), 1.77-1.70 (m, 1H), 1.16-1.09 (m, 1H).

^{13}C NMR (151 MHz, $CDCl_3$) δ 158.8, 146.2, 143.8, 142.4, 130.1, 129.4, 128.7, 127.2, 125.5, 125.0, 123.9, 120.6, 114.4, 100.8, 100.3, 99.0, 94.5, 67.4, 62.8, 36.0, 28.7, 23.7.

HRMS (ESI) m/z: $[M + H]^+$ Calcd for $C_{26}H_{26}NO_3^+$ 400.1907; Found 400.1910.

1-(2-phenyl-1-(p-tolyl)propan-2-yl)-1H-indole (8r)

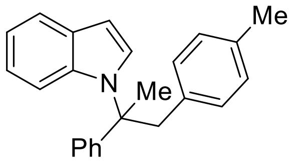

8r

Prepared according to **GP2** with *N*-alkenyl indole **1a'** (21.9 mg, 0.10 mmol, 1.0 equiv.) and **2y** (28.1 mg, 0.20 mmol, 2.0 equiv.). Flash column chromatography (SiO_2, PE) afforded the desired product **8r** as a white solid (18.6 mg, 57%).

^{1}H NMR (500 MHz, $CDCl_3$) δ 7.65 (d, *J* = 7.9 Hz, 1H), 7.33-7.27 (m, 3H), 7.21-7.19

(m, 2H), 7.13 (d, J = 3.3 Hz, 1H), 7.06-7.03 (m, 1H), 6.93-6.90 (m, 1H), 6.86 (d, J = 7.8 Hz, 2H), 6.73 (d, J = 8.4 Hz, 1H), 6.45 (d, J = 3.3 Hz, 1H), 6.28 (d, J = 7.9 Hz, 2H), 3.97 (d, J = 12.9 Hz, 1H), 3.49 (d, J = 12.9 Hz, 1H), 2.25 (s, 3H), 1.79 (s, 3H).

^{13}C NMR (126 MHz, CDCl$_3$) δ 146.5, 136.1, 135.1, 132.9, 130.3, 130.1, 128.6, 128.4, 127.0, 126.7, 125.7, 120.9, 120.7, 119.1, 114.0, 100.2, 63.3, 43.3, 29.0, 21.0.

HRMS (ESI) m/z: $[M + H]^+$ Calcd for $C_{24}H_{24}N^+$ 326.1903; Found 326.1903.

1-(1-(4-methoxyphenyl)-2-phenylpropan-2-yl)-1*H*-indole (8s)

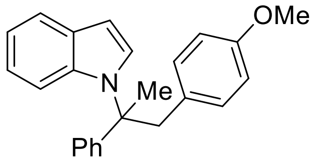

8s

Prepared according to **GP2** with *N*-alkenyl indole **1a'** (21.9 mg, 0.10 mmol, 1.0 equiv.) and **2aa** (31.3 mg, 0.20 mmol, 2.0 equiv.). Flash column chromatography (SiO_2, 40:1 PE/EtOAc) afforded the desired product **8s** as a white solid (22.5 mg, 66%).

^{1}H NMR (400 MHz, CDCl$_3$) δ 7.65 (d, J = 7.9 Hz, 1H), 7.33-7.28 (m, 3H), 7.21-7.19 (m, 2H), 7.11 (d, J = 3.3 Hz, 1H), 7.05 (t, J = 7.4 Hz, 1H), 6.91 (t, J = 7.6 Hz, 1H), 6.72 (d, J = 8.4 Hz, 1H), 6.59 (d, J = 8.6 Hz, 2H), 6.44 (d, J = 3.2 Hz, 1H), 6.29 (d, J = 8.6 Hz, 2H), 3.94 (d, J = 13.0 Hz, 1H), 3.72 (s, 3H), 3.47 (d, J = 13.0 Hz, 1H), 1.77 (s, 3H).

^{13}C NMR (101 MHz, CDCl$_3$) δ 158.2, 146.4, 135.1, 131.3, 130.0, 128.6, 128.0, 127.0, 126.7, 125.7, 120.9, 120.7, 119.1, 114.0, 113.0, 100.2, 63.4, 55.1, 42.7, 28.9.

HRMS (ESI) m/z: $[M + H]^+$ Calcd for $C_{24}H_{24}NO^+$ 342.1852; Found 342.1853.

1-(1-(4-fluorophenyl)-2-phenylpropan-2-yl)-1H-indole (8t)

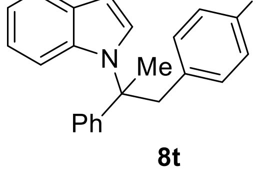

8t

Prepared according to **GP2** with *N*-alkenyl indole **1a'** (21.9 mg, 0.10 mmol, 1.0 equiv.) and **2z** (28.9 mg, 0.20 mmol, 2.0 equiv.). Flash column chromatography (SiO_2, PE) afforded the desired product **8t** as a white solid (19.8 mg, 60%).

^{1}H NMR (400 MHz, CDCl$_3$) δ 7.65 (d, J = 7.9 Hz, 1H), 7.34-7.28 (m, 3H), 7.21-7.19 (m, 2H), 7.08-7.04 (m, 2H), 6.92 (t, J = 7.4 Hz, 1H), 6.76-6.70 (m, 3H), 6.44 (d, J = 3.1

Hz, 1H), 6.32 (dd, J = 8.4, 5.6 Hz, 2H), 3.98 (d, J = 13.0 Hz, 1H), 3.50 (d, J = 13.0 Hz, 1H), 1.77 (s, 3H).

^{13}C NMR (101 MHz, CDCl$_3$) δ 161.8 (d, J_{C-F} = 244.9 Hz), 146.2, 135.0, 131.8, 131.7, 131.6, 130.0, 128.7, 127.2, 126.6, 125.6, 120.9 (d, J_{C-F} = 15.6 Hz), 119.2,114.5 (d, J_{C-F} = 21.1 Hz), 113.9, 100.3, 63.2, 42.6, 29.0.

^{19}F NMR (376 MHz, CDCl3) δ -116.3

HRMS (ESI) m/z: $[M + H]^+$ Calcd for $C_{23}H_{21}FN^+$ 330.1653; Found 330.1648.

1-(2-phenyl-1-(m-tolyl)propan-2-yl)-1H-indole (8u)

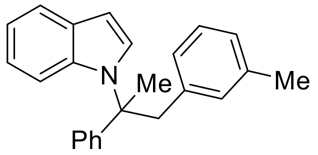

8u

Prepared according to **GP2** with *N*-alkenyl indole **1a'** (21.9 mg, 0.10 mmol, 1.0 equiv.) and **2ab** (28.1 mg, 0.20 mmol, 2.0 equiv.). Flash column chromatography (SiO_2, PE) afforded the desired product **8u** as a colorless oil (16.9 mg, 52%).

^{1}H NMR (400 MHz, CDCl$_3$) δ 7.67 (d, J = 7.9 Hz, 1H), 7.35-7.29 (m, 3H), 7.25-7.23 (m, 2H), 7.12 (d, J = 3.3 Hz, 1H), 7.07 (t, J = 7.4 Hz, 1H), 6.98-6.92 (m, 3H), 6.74 (d, J = 8.4 Hz, 1H), 6.46 (d, J = 3.2 Hz, 1H), 6.21 (d, J = 6.3 Hz, 1H), 6.17 (s, 1H), 3.98 (d, J = 12.8 Hz, 1H), 3.50 (d, J = 12.8 Hz, 1H), 2.14 (s, 3H), 1.80 (s, 3H).

^{13}C NMR (101 MHz, CDCl$_3$) δ 146.4, 137.1, 135.9, 135.2, 131.3, 130.1, 128.6, 127.4, 127.3, 127.2, 127.0, 126.8, 125.7, 120.9, 120.7, 119.1, 114.0, 100.1, 63.3, 43.6, 29.0, 21.2.

HRMS (ESI) m/z: $[M + H]^+$ Calcd for $C_{24}H_{24}N^+$ 326.1903; Found 326.1903.

6.3 Some inferior results in substrate expanding

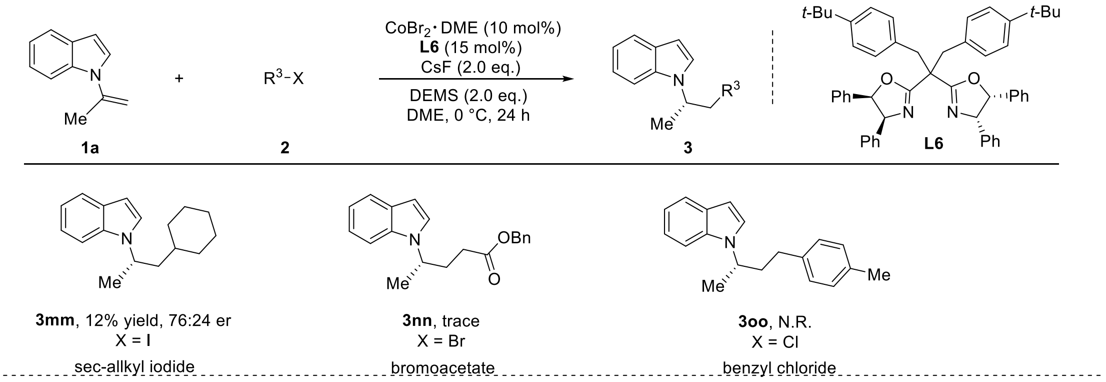

Supplementary Figure 5. Some inferior results in *anti*-Markovnikov hydroalkylation

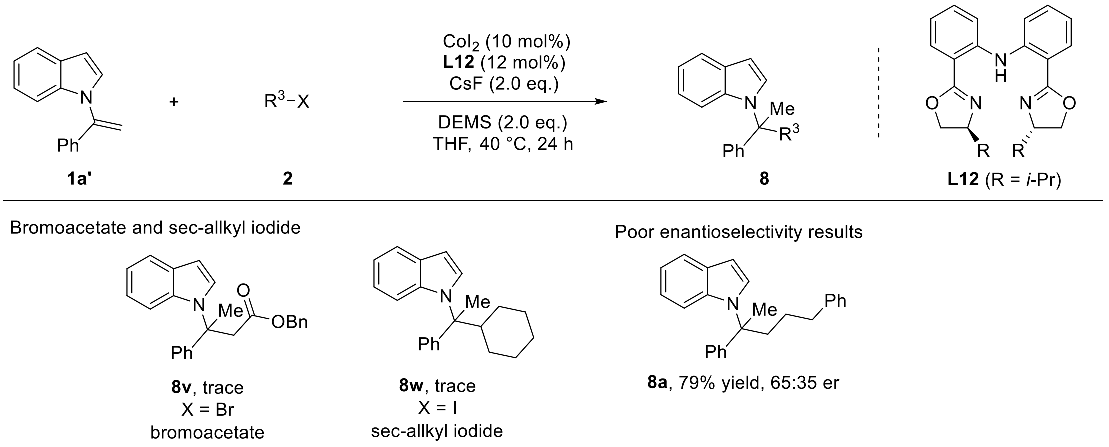

Supplementary Figure 6. Some inferior results in Markovnikov hydroalkylation

(*S*)-1-(1-cyclohexylpropan-2-yl)-1*H*-indole (3mm)

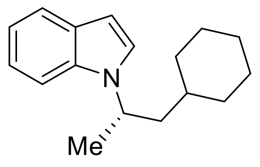

3mm

Prepared according to **GP1** with *N*-alkenyl indole **1a** (28.3 mg, 0.10 mmol, 1.0 equiv.) and **2x** (42.0 mg, 0.20 mmol, 2.0 equiv.). Flash column chromatography (SiO_2, PE) afforded the desired product **3mm** as a colorless oil (2.9 mg, 12%).

^{1}H NMR (400 MHz, CDCl$_3$) δ 7.64 (d, *J* = 7.8 Hz, 1H), 7.39 (d, *J* = 8.3 Hz, 1H), 7.22-7.19 (m, 2H), 7.10 (t, *J* = 7.4 Hz, 1H), 6.53 (d, *J* = 3.2 Hz, 1H), 4.67-4.58 (m, 1H), 1.92-1.85 (m, 1H), 1.79 (d, *J* = 12.6 Hz, 1H), 1.67-1.60 (m, 5H), 1.47 (d, *J* = 6.7 Hz, 3H), 1.21-1.09 (m, 4H), 1.00-0.88 (m, 2H).

^{13}C NMR (101 MHz, CDCl$_3$) δ 135.8, 128.4, 124.0, 121.1, 120.9, 119.1, 109.4, 101.3,

48.7, 44.8, 34.3, 33.4, 33.3, 26.4, 26.1, 26.0, 21.8.

HRMS (ESI) m/z: $[M + H]^+$ Calcd for $C_{17}H_{24}N^+$ 242.1903; Found 242.1906.

HPLC: 24:76 er determined by analytical HPLC, Daicel CHIRALPAK® AD-H column, 25 °C, Hexane:*i*-PrOH = 100:0, 1.0 mL/min, 254 nm, t_{minor} = 5.2 min, t_{major} = 5.5 min.

7. Synthetic transformations

7.1 Synthesis of GSK 126 intermediate

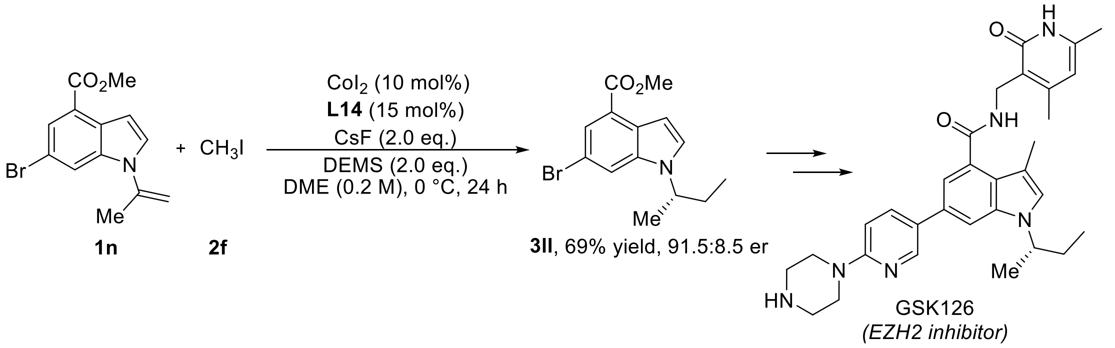

Supplementary Figure 7. Synthesis of GSK 126 intermediate

methyl (*S*)-6-bromo-1-(*sec*-butyl)-1*H*-indole-4-carboxylate (3ll)[15]

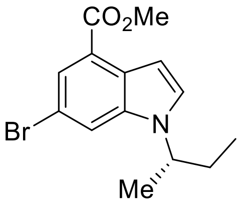

3ll

Prepared according to **GP1** with *N*-alkenyl indole **1n** (29.3 mg, 0.10 mmol, 1.0 equiv.), **2f** (46.8 mg, 0.30 mmol, 3.0 equiv.), CoI_2 as Co-salt and **L14** instead of **L6**. Flash column chromatography (SiO_2, 20:1 PE/EtOAc) afforded the desired product **3ll** as a colorless oil (21.4 mg, 69%).

^{1}H NMR (400 MHz, $CDCl_3$) δ 7.99 (d, J = 1.6 Hz, 1H), 7.71 (s, 1H), 7.30 (d, J = 3.3 Hz, 1H), 7.13 (d, J = 3.2 Hz, 1H), 4.36 (m, 1H), 3.98 (s, 3H), 1.88 (m, 2H), 1.51 (d, J = 6.8 Hz, 3H), 0.82 (t, J = 7.4 Hz, 3H).

^{13}C NMR (101 MHz, $CDCl_3$) δ 166.9, 137.7, 126.9, 125.6, 122.6, 116.9, 113.7, 102.9, 53.5, 51.9, 30.0, 20.9, 10.8.

HRMS (ESI) m/z: $[M + H]^+$ Calcd for $C_{14}H_{17}BrNO_2^+$ 310.0437; Found 310.0434.

HPLC: 91.5:8.5 er determined by analytical HPLC, Daicel CHIRALPAK® ID column, 25 °C, Hexane:*i*-PrOH = 98:2, 1.0 mL/min, 254 nm, t_{major} = 8.5 min, t_{minor} = 9.4 min.

$[\alpha]_D^{20}$ = -16.1 (c = 0.32, $CHCl_3$).

7.2 Synthesis of (-)-monomorine intermediate

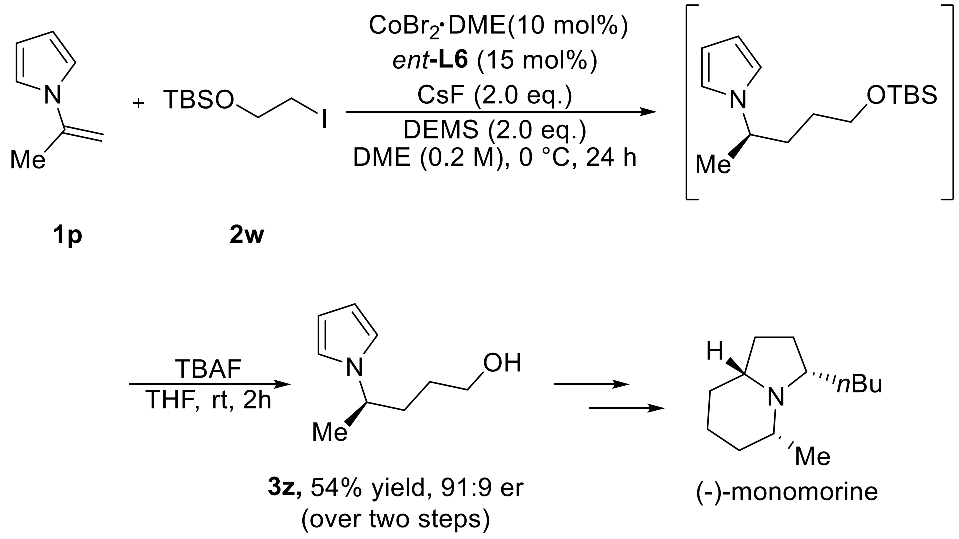

Supplementary Figure 8. Synthesis of (-)-monomorine intermediate

(*R*)-4-(1*H*-pyrrol-1-yl)pentan-1-ol (3z)[14]

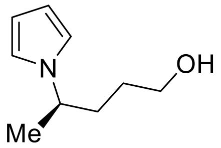

3z

Prepared according to **GP1** with *N*-alkenyl pyrrole **1p** (10.7 mg, 0.10 mmol, 1.0 equiv.), **2w** (57.2 mg, 0.20 mmol, 2.0 equiv.) and *ent*-**L6** instead of **L6**. At the end of the reaction, the crude product was obtained by filtration through a short silica gel column and concentrated under reduced pressure. The crude product was dissolved in 1.0 mL THF, followed by slow dropwise addition of 1.0 mL TBAF (1.0 M in THF), stirred for 2 hours. After the reaction was completed, the reaction mixture was diluted with saturated NH_4Cl (aq., 1.0 mL) and EtOAc (2.0 mL). The aqueous phase was extracted with EtOAc (2 x 2.0 mL) and the combined organic phases were concentrated in vacuo. Flash column chromatography (SiO_2, 5:1 PE/EtOAc) afforded the desired product **3z** as a colorless oil (8.3 mg, 54%, over two steps).

^{1}H NMR (400 MHz, $CDCl_3$) δ 6.70 (t, *J* = 2.1 Hz, 2H), 6.14 (t, *J* = 2.1 Hz, 2H), 4.09-4.01 (m, 1H), 3.62-3.53 (m, 2H), 1.80 (q, *J* = 7.6 Hz, 2H), 1.47 (d, *J* = 6.8 Hz, 3H), 1.39-1.30 (m, 2H).

^{13}C NMR (101 MHz, $CDCl_3$) δ 118.4, 107.6, 62.4, 55.4, 34.5, 29.5, 22.4.

HRMS (ESI) m/z: $[M + H]^+$ Calcd for $C_9H_{16}NO^+$ 154.1226; Found 154.1229.

HPLC: 91:9 er determined by analytical HPLC, Daicel CHIRALCEL® OD-H column, 25 °C, Hexane:*i*-PrOH = 85:15, 1.0 mL/min, 254 nm, t_{major} = 6.0 min, t_{minor} = 7.4 min. **[α]$_D^{20}$** = -12.3 (*c* = 0.18, MeOH).

7.3 Synthesis of (-)-indolizidine 167B intermediate

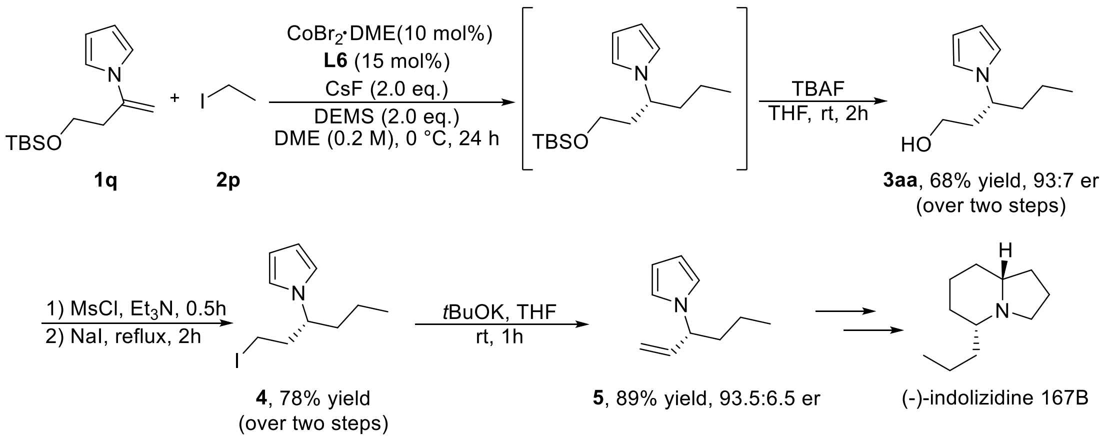

Supplementary Figure 9. Synthesis of (-)-indolizidine 167B intermediate

(*R*)-3-(1*H*-pyrrol-1-yl)hexan-1-ol (3aa)

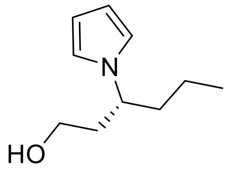

3aa

Prepared according to **GP1** with *N*-alkenyl pyrrole **1q** (25.1 mg, 0.10 mmol, 1.0 equiv.) and **2p** (47.1 mg, 0.30 mmol, 3.0 equiv.). At the end of the reaction, the crude product was obtained by filtration through a short silica gel column and concentrated under reduced pressure. The crude product was dissolved in 1.0 mL THF, followed by slow dropwise addition of 1.0 mL TBAF (1.0 M in THF), stirred for 2 hours. After the reaction was completed, the reaction mixture was diluted with saturated NH_4Cl (aq., 1.0 mL) and EtOAc (2.0 mL). The aqueous phase was extracted with EtOAc (2 x 2.0 mL) and the combined organic phases were concentrated in vacuo. Flash column chromatography (SiO_2, 5:1 PE/EtOAc) afforded the desired product **3aa** as a colorless oil (11.4 mg, 68%, over two steps).

^{1}H NMR (400 MHz, Acetone-*d*$_6$) δ 6.71 (t, *J* = 2.1 Hz, 2H), 5.99 (t, *J* = 2.1 Hz, 2H), 4.17-4.09 (m, 1H), 3.53-3.51 (m, 1H), 3.44-3.38 (m, 1H), 3.30-3.23 (m, 1H), 1.94-1.87 (m, 2H), 1.80-1.66 (m, 2H), 1.23-1.04 (m, 2H), 0.85 (t, *J* = 7.37 Hz, 3H).

^{13}C NMR (101 MHz, Acetone-d_6) δ 118.7, 107.2, 58.1, 56.2, 39.6, 38.5, 19.2, 13.12.

HRMS (ESI) m/z: $[M + H]^+$ Calcd for $C_{10}H_{18}NO^+$ 168.1383; Found 168.1381.

HPLC: 93:7 er determined by analytical HPLC, Daicel CHIRALCEL® OD-H column, 25 °C, Hexane:*i*-PrOH = 90:10, 1.0 mL/min, 220 nm, t_{major} = 5.8 min, t_{minor} = 6.9 min.

$[\alpha]_D^{20}$ = -12.7 (c = 0.5, $CHCl_3$).

(*R*)-1-(1-iodohexan-3-yl)-1*H*-pyrrole (4)

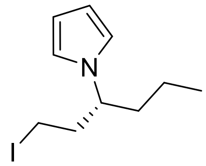

4

Preparation of **4**: Methods according to the literature[16]. pyrrole **3aa** (33.5 mg, 0.20 mmol, 1.0 equiv.) was stirred in dry CH_2Cl_2 (2.0 mL) at 0 °C under an atmosphere of Ar. Et_3N (70 μl, 0.50 mmol, 2.5 equiv.) and MsCl (24 μl, 0.30 mmol, 1.5 equiv.) was added and the reaction solution was stirred at 0 °C for 0.5 h. The reaction was directly subject to a short pad of silica gel with dichloromethane as eluent. Removal of the solvent afforded the crude product, which was used for next step without further purification. The crude product was then dissolved in acetone (3.0 mL), followed by addition of NaI (149.9 mg, 1.0 mmol, 5.0 equiv.) The resulting reaction mixture was brought to reflux for 2 h. At the end of the reaction, the reaction solution was concentrated. Flash column chromatography (SiO_2, PE) afforded the desired product **4** as a colorless oil (43.2 mg, 78%, over two steps).

^{1}H NMR (600 MHz, $CDCl_3$) δ 6.68 (t, J = 2.1 Hz, 2H), 6.15 (t, J = 2.1 Hz, 2H), 4.03-3.99 (m, 1H), 3.08-3.04 (m, 1H), 2.76-2.72 (m, 1H), 2.18-2.15 (m, 2H), 1.83-1.76 (m, 1H), 1.71-1.66 (m, 1H), 1.27-1.14 (m, 2H), 0.89 (t, J = 7.4 Hz, 3H).

^{13}C NMR (151 MHz, $CDCl_3$) δ 118.8, 107.9, 59.8, 40.1, 38.0, 19.4, 13.7, 2.9.

HRMS (ESI) m/z: $[M + H]^+$ Calcd for $C_{10}H_{17}IN^+$ 278.0400; Found 278.0396.

$[\alpha]_D^{20}$ = -58.7 (c = 0.95, $CHCl_3$).

(*R*)-1-(hex-1-en-3-yl)-1*H*-pyrrole (5)[17,18]

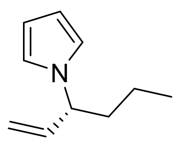

5

Preparation of **5**: Methods according to the literature[16]. pyrrole **4** (30.5 mg, 0.11 mmol, 1.0 equiv.) was stirred in THF (1.5 mL) at room temperature under an atmosphere of Ar. *t*BuOK (61.7 mg, 0.55 mmol, 5.0 equiv.) was added and the reaction mixture was stirred for 1 h before quenching with 1N HCl. The resulting mixture was extracted wit EtOAc (2mL x 3). Removal of the combined organic phases and flash column chromatography (SiO_2, PE) afforded the desired product **5** as a colorless oil (14.6 mg, 89%).

^{1}H NMR (400 MHz, $CDCl_3$) δ 6.69 (t, J = 2.1 Hz, 2H), 6.16 (t, J = 2.1 Hz, 2H), 6.01-5.93 (m, 1H), 5.14-5.01 (m, 2H), 4.42 (q, J = 6.5 Hz, 1H), 1.92-1.77 (m, 2H), 1.33-1.27 (m, 1H), 1.24-1.20 (m, 1H), 0.92 (t, J = 7.4 Hz, 3H).

^{13}C NMR (101 MHz, $CDCl_3$) δ 138.9, 119.1, 115.6, 107.7, 61.7, 37.1, 19.3, 13.7.

HRMS (ESI) m/z: $[M + H]^+$ Calcd for $C_{10}H_{16}N^+$ 150.1277; Found 150.1271.

HPLC: 6.5:93.5 er determined by analytical HPLC, Daicel CHIRALCEL® OJ-3 column, 25 °C, Hexane:*i*-PrOH = 100:0, 0.5 mL/min, 220 nm, t_{minor} = 15.0 min, t_{major} = 15.6 min.

$[\alpha]_D^{26}$ = -27.9 (c = 0.32, MeOH).

7.4 Synthesis of dicarbazole monomer (Can also be used as an intermediate in synthesis dicarboxylated hybrid monomer Py_2/Cbz_3)

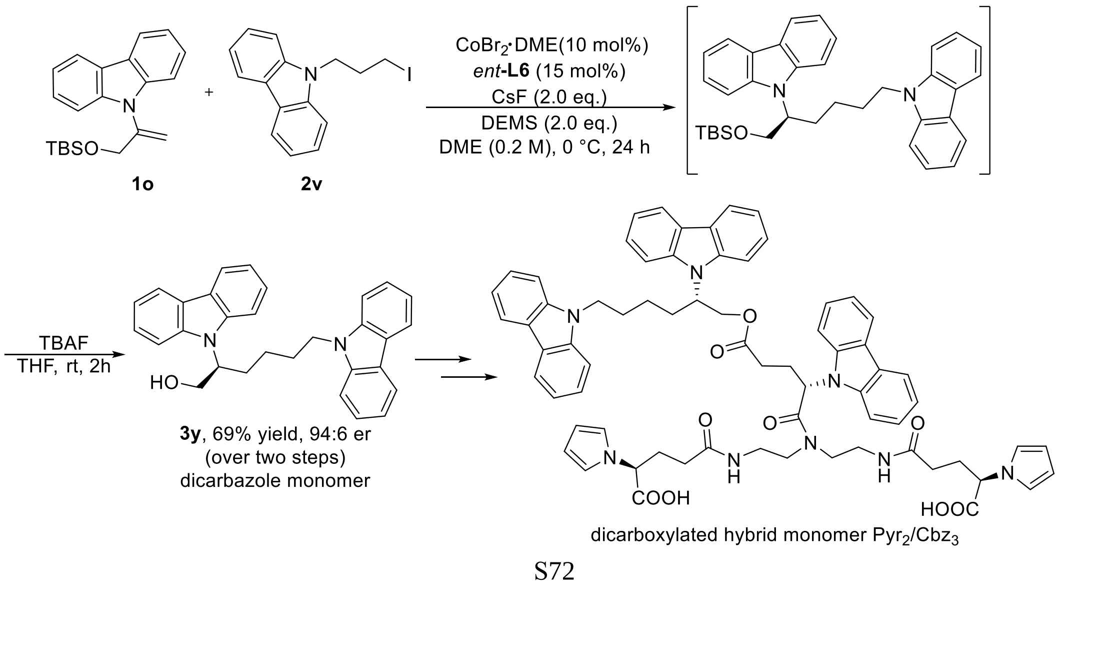

Supplementary Figure 10. Synthesis of dicarbazole monomer (Can also be used as an intermediate in synthesis dicarboxylated hybrid monomer Py_2/Cbz_3)

(*S*)-2,6-di(9*H*-carbazol-9-yl)hexan-1-ol (3y)[12,13]

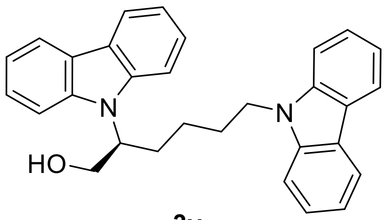

3y

Prepared according to **GP1** with *N*-alkenyl carbazole **1o** (33.8 mg, 0.10 mmol, 1.0 equiv.), **2v** (67.0 mg, 0.20 mmol, 2.0 equiv.) and *ent*-**L6** instead of **L6**. At the end of the reaction, the crude product was obtained by filtration through a short silica gel column and concentrated under reduced pressure. The crude product was dissolved in 1.0 mL THF, followed by slow dropwise addition of 1.0 mL TBAF (1.0 M in THF), stirred for 2 hours. After the reaction was completed, the reaction mixture was diluted with saturated NH_4Cl (aq., 1.0 mL) and EtOAc (2.0 mL). The aqueous phase was extracted with EtOAc (2 x 2.0 mL) and the combined organic phases were concentrated in vacuo. Flash column chromatography (SiO_2, 4:1 PE/EtOAc) afforded the desired product **3y** as a white solid (29.8 mg, 69%, over two steps).

^{1}H NMR (400 MHz, $CDCl_3$) δ 8.11-8.04 (m, 4H), 7.40-7.34 (m, 6H), 7.24-7.14 (m, 6H), 4.69-4.61 (m, 1H), 4.25 (dd, J = 11.4, 9.1 Hz, 1H), 4.12-3.97 (m, 2H), 3.89 (dd, J = 11.1, 4.6 Hz, 1H), 2.32-2.22 (m, 1H), 1.90-1.54 (m, 4H), 1.25-1.11 (m, 2H).

^{13}C NMR (101 MHz, $CDCl_3$) δ 140.2, 125.6, 122.7, 120.3, 119.2, 118.8, 108.5, 63.5, 58.2, 42.6, 28.9, 28.5, 24.3.

HRMS (ESI) m/z: $[M + H]^+$ Calcd for $C_{30}H_{29}N_2O^+$ 433.2274; Found 433.2279.

HPLC: 6:94 er determined by analytical HPLC, Daicel CHIRALPAK® IB-3 column, 25 °C, Hexane:*i*-PrOH = 80:20, 1.0 mL/min, 254 nm, t_{minor} = 22.4 min, t_{major} = 30.1 min.

$[\alpha]_D^{21}$ = +9.9 (c = 0.28, CH_2Cl_2).

7.5 Reduction of indole to indoline

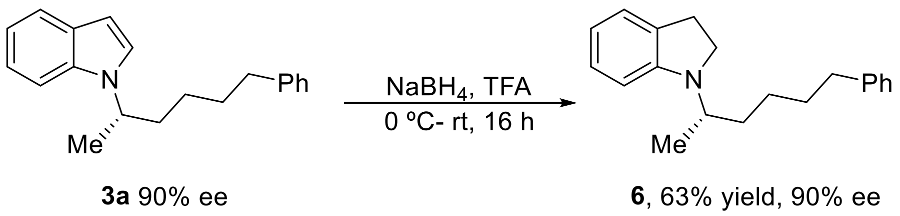

Supplementary Figure 11. Reduction of indole to indoline

(*S*)-1-(6-phenylhexan-2-yl)indoline (6)

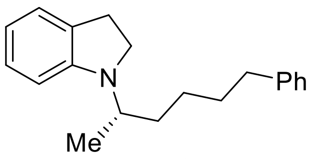

6

Preparation of **6**: Methods according to the literature[19]. indole **3a** (27.7 mg, 0.10 mmol, 1.0 equiv.) was stirred in TFA (0.7 mL) at 0 °C under an atmosphere of Ar. $NaBH_4$ (11.3 mg, 0.30 mmol, 3.0 equiv.) was added and bring the reaction mixture to room temperature and stirred for 16 h before quenching with saturated aqueous sodium bicarbonate solution. The resulting mixture was extracted wit EtOAc (2mL x 3). Removal of the combined organic phases and flash column chromatography (SiO_2, 50:1 PE/EtOAc) afforded the desired product **6** as a colorless oil (17.6 mg, 63%).

^{1}H NMR (400 MHz, $CDCl_3$) δ 7.31-7.28 (m, 2H), 7.21-7.18 (m, 3H), 7.07-7.04 (m, 2H), 6.60 (t, *J* = 7.3 Hz, 1H), 6.40 (d, *J* = 8.1 Hz, 1H), 3.65-3.57 (m, 1H), 3.32 (td, *J* = 8.7, 1.9 Hz, 2H), 2.95 (t, *J* = 8.5 Hz, 2H), 2.63 (t, *J* = 7.7 Hz, 2H), 1.71-1.61 (m, 3H), 1.53-1.40 (m, 3H), 1.09 (d, *J* = 6.6 Hz, 3H).

^{13}C NMR (101 MHz, $CDCl_3$) δ 151.5, 142.6, 130.0, 128.4, 128.2, 127.2, 125.6, 124.3, 116.5, 106.6, 50.0, 45.5, 35.9, 33.7, 31.4, 28.1, 26.6, 15.2.

HRMS (ESI) m/z: $[M + H]^+$ Calcd for $C_{20}H_{26}N^+$ 280.2060; Found 280.2056.

HPLC: 95:5 er determined by analytical HPLC, Daicel CHIRALPAK® IB-3 column, 25 °C, Hexane:*i*-PrOH = 98:2, 1.0 mL/min, 254 nm, t_{major} = 4.6 min, t_{minor} = 5.1 min.

$[\alpha]_D^{20}$ = +16.5 (*c* = 0.41, $CHCl_3$).

7.6 Oxidation of indole to isatin

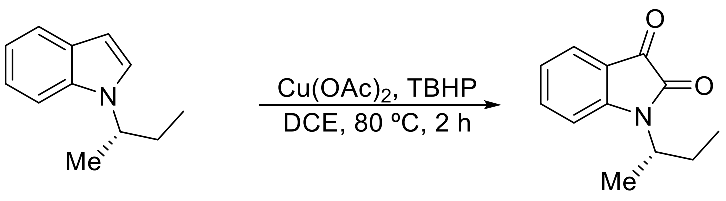

3f 91% ee　　　　**7**, 73% yield, 91% ee

Supplementary Figure 12. Oxidation of indole to isatin

(*S*)-1-(*sec*-butyl)indoline-2,3-dione (7)

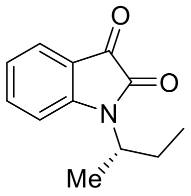

7

Preparation of **7**: Methods according to the literature[20]. $Cu(OAc)_2$ (12.0 mg, 0.06 mmol, 0.3 equiv.) and indole **3f** (34.7 mg, 0.20 mmol, 1.0 equiv.) were dissolved in DCE (2mL) in a glass pressure tube, followed by the addition of TBHP (1mL, 70% solution in water) and filling with Ar at 80 °C for 2 h. After cooling to room temperature, the reaction was quenched by the addition of saturated sodium thiosulfate solution. The resulting mixture was extracted with EtOAc (3mL x 3). Removal of the combined organic phases and flash column chromatography (SiO_2, 6:1 PE/EtOAc) afforded the desired product **7** as a red oil (29.7 mg, 73%).

^{1}H NMR (400 MHz, $CDCl_3$) δ 7.61 (dd, J = 7.4, 1.0 Hz, 1H), 7.55 (td, J = 7.9, 1.4 Hz, 1H), 7.11-7.07 (m, 1H), 7.01 (d, J = 8.0 Hz, 1H), 4.33-4.24 (m, 1H), 2.09-1.95 (m, 1H), 1.87-1.77 (m, 1H), 1.48 (d, J = 7.0 Hz, 3H), 0.92 (t, J = 7.4 Hz, 3H).

^{13}C NMR (101 MHz, $CDCl_3$) δ 183.8, 158.1, 150.8, 138.1, 125.6, 123.3, 117.8, 111.4, 50.8, 26.3, 17.6, 11.3.

HRMS (ESI) m/z: $[M + H]^+$ Calcd for $C_{12}H_{14}NO_2^+$ 204.1019; Found 204.1022.

HPLC: 95.5:4.5 er determined by analytical HPLC, Daicel CHIRALPAK® AS-H column, 25 °C, Hexane:*i*-PrOH = 90:10, 1.0 mL/min, 254 nm, t_{major} = 10.1 min, t_{minor} = 11.4 min.

$[\alpha]_D^{20}$ = +28.8 (c = 0.41, $CHCl_3$).

8. Drug Activity Research

8.1 Cell Culture

Human immortal keratinocyte line (HaCaT) cells were purchased from KUNMING CELL BANK.CAS (Kunming, China). HaCaT cells were cultured in Dulbecco's modified eagle medium (DMEM) containing 10% fetal bovine serum (FBS) and 1% (v/v) antibiotic (100U/mL penicillin and 100μg/mL streptomycin) in 10-cm Petri dishes. When the cells reached to 70%-80% confluence, they were subcultured, froze or used for experiments.

8.2 UV irradiation

The HaCaT cells were washed with 1×PBS and then covered with a thin layer of PBS to avoid drying. Subsequently, cells were exposed to different doses of UVB (0-300 mJ/cm^2) using a UVB irradiation machine (SCIENTZ03-a, Ningbo Scientz BIOTECHNOLOGY Co., Ltd., China). Then, removed PBS and added a fresh serum-free medium for culturing for another 6 hours.

8.3 MTT assay

The 3-(4,5-dimethylthiazol-2-yl)-2,5-diphenyltetrazolium bromide (MTT) assay was applied to assess the effects of N-Alkylated Indoles on UVB-induced damage in HaCaT cells and evaluated the influence of the indicated compounds on the cell viability. HaCaT cells (5x10^{4}cells/well) were seeded in 96-well plates in culture medium for 24h, then irradiated by 150 mJ/cm^2 UVB. After that, the cells were treated with 35μg/mL different N-Alkylated Indoles or (-)-Epigallocatechin Gallate sulfate (EGCG) for 6h. Subsequently, the cells were washed with culture medium and further incubated with 5mg/mL MTT at 37°C for 4h. Next, the culture medium was removed, and 100μL DMSO was added to the wells. Finally, the purple formazan dye aggregates in the cell were detected using a microplate reader (SpectraMax M2, Molecular Devices) at 490 nm absorbance. For measuring the influence of the indicated compounds on the cell viability of the HaCaT cells, the cells (5x10^{4}cells/well) were seeded in 96-well

plates in culture medium for 24h, and then treated with 35μg/mL indicated compounds for another 24h. After that, the cell viability was tested by the MTT assay described above.

8.4. Statistical analysis

Statistics were calculated with software GraphPad Prism 9.5.0. The data are presented as mean $\pm$ SD. Differences between groups were determined by one-way ANOVA analysis and multiple comparisons. Adjustments were made for multiple comparisons. All p values adopted adjusted p values and $p < 0.05$ were considered statistically significant ($^{*}p < 0.05$, $^{**}p < 0.01$, $^{***}p < 0.001$, $^{****}p < 0.0001$; $^{\#}p < 0.05$, $^{\#\#}p < 0.01$, $^{\#\#\#}p < 0.001$, $^{\#\#\#\#}p < 0.0001$).

9. Mechanistic Study

9.1 Nonlinear effect study

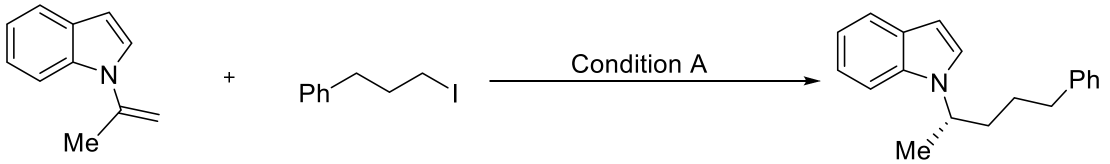

1a **2a** (2.0 eq.) **3a**

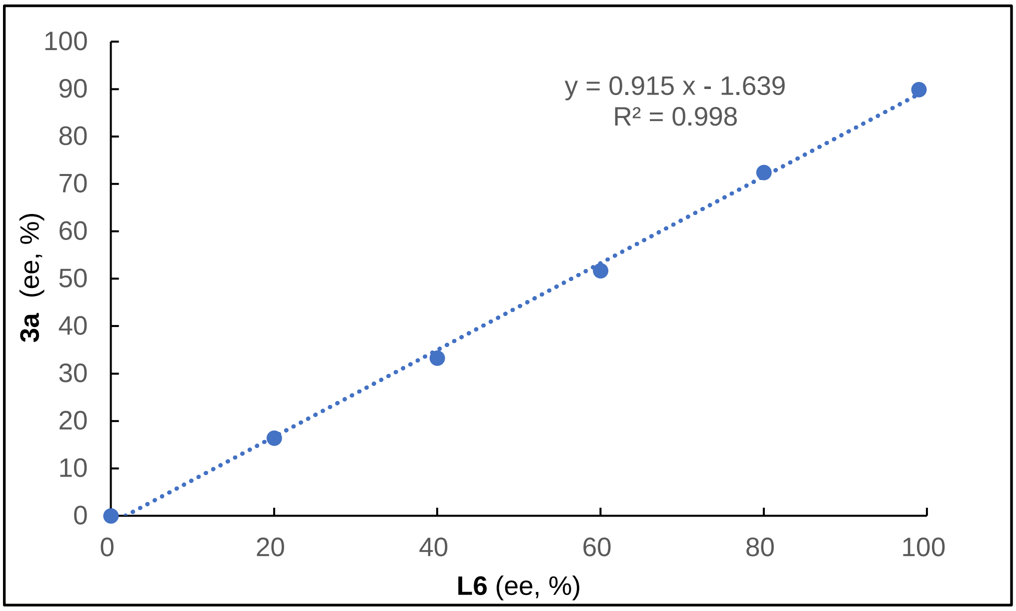

ee (%), L6	0	20	40	60	80	99
ee (%), 3a	0	16.4	33.3	51.7	72.4	89.9

Supplementary Figure 13. Nonlinear effect study

In a Argon-filled glove box, five 10-mL scintillation vials were charged with the specified amount of (*S,S*)-**L6** and (*R,R*)- **L6** to provide the enantiomeric composition of **L6** as specified. To each of these vials, $CoBr_2$•DME (3.1 mg, 10 mol%) and DME (0.5 mL) was added and these vials were stirred to homogeneity (10 min). Once homogeneity was reached, CsF (30.4 mg, 0.20 mmol, 2.0 equiv), *N*-alkenyl indole **1a** (0.20 mmol, 1.0 equiv), alkyl iodide **2a** (49.2 mg, 0.2 mmol, 2.0 equiv) and $(OEt)_2MeSiH$ (32.0 uL, 0.20 mmol, 2.0 equiv.) were sequentially added. The reaction vials were brought outside of the glove box, and stirred for 24 h. Afterwards, EtOAc (2.0 mL) and saturated NH_4Cl (aq., 1.0 mL) were added to the reaction mixture. The

organic phase was separated and the aqueous phase was extracted with EtOAc (2x2.0 mL). The combined organic phases were dried over Na_2SO_4, and the volatiles were removed to afford the crude product. Then, the mixture was purified by PTLC and the enantiomeric excess (ee) was determined by HPLC analysis.

9.2 Radical clock experiments

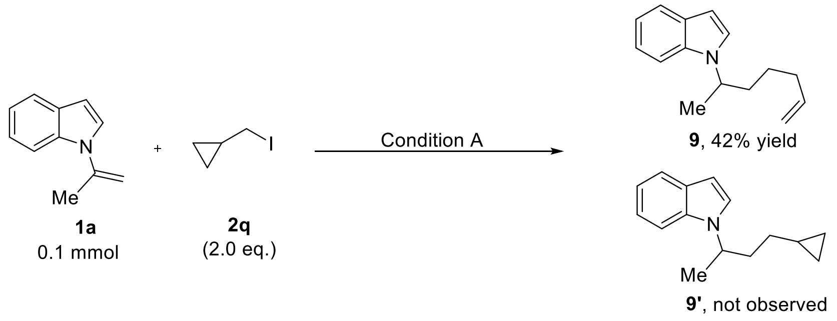

Supplementary Figure 14. Radical clock experiments results

To an oven-dried 10.0 mL Teflon-screw cap test tube containing a magnetic stir was charged with $CoBr_2$•DME (3.1 mg, 10 mol%) and ligand **L6** (11.3mg, 15 mol%) under a Argon atmosphere using glove-box techniques. Subsequently, anhydrous DME (0.5 mL) was added, and the mixture was stirred for 10 minutes at room temperature. Then, CsF (30.4 mg, 0.20 mmol, 2.0 equiv.), **1a** (0.10 mmol, 1.0 equiv), alkyl iodide **2q** (0.20 mmol, 2.0 equiv.) and $(OEt)_2MeSiH$ (32 uL, 0.20 mmol, 2.0 equiv.) were sequentially added. Afterwards, the tube was sealed with airtight electrical tapes and removed from the glove box and stirred at 0 °C for 24 hours at 500 rpm. After the reaction was completed, the reaction mixture was diluted with saturated NH_4Cl (aq., 2.0 mL) and EtOAc (3.0 mL). The aqueous phase was extracted with EtOAc (2 x 3.0 mL) and the combined organic phases were concentrated in vacuo. The crude mixture was purified by flash column chromatography on silica gel using PE as eluent to obtain the ring-opened product **9** colorless oil (8.9 mg, 42%), not observed the cyclopropyl ring retained product **9'**, it was revealed that the activation of alkyl halides proceeded through a radical pathway.

1-(hept-6-en-2-yl)-1*H*-indole (9)

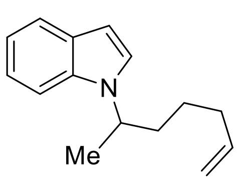

9

^{1}H NMR (600 MHz, CDCl$_3$) δ 7.63 (d, *J* = 7.8 Hz, 1H), 7.37 (d, *J* = 8.3 Hz, 1H), 7.21-7.18 (m, 2H), 7.09 (t, *J* = 7.4 Hz, 1H), 6.53 (s, 1H), 5.72 (td, *J* = 16.6, 6.9 Hz, 1H), 4.98-4.92 (m, 2H), 4.52-4.47 (m, 1H), 2.03 (q, *J* = 7.3 Hz, 2H), 1.95-1.89 (m, 1H), 1.85-1.79 (m, 1H), 1.51 (d, *J* = 6.5 Hz, 3H), 1.40-1.32 (m, 1H), 1.30-1.23 (m, 1H).

^{13}C NMR (151 MHz, CDCl$_3$) δ 138.3, 135.9, 128.4, 123.9, 121.1, 120.9, 119.1, 114.9, 109.4, 101.4, 51.4, 36.5, 33.4, 25.6, 21.4.

HRMS (ESI) m/z: [M + H]$^+$ Calcd for $C_{15}H_{20}N^+$ 214.1590; Found 214.1589.

9.3 Radical trapping experiment

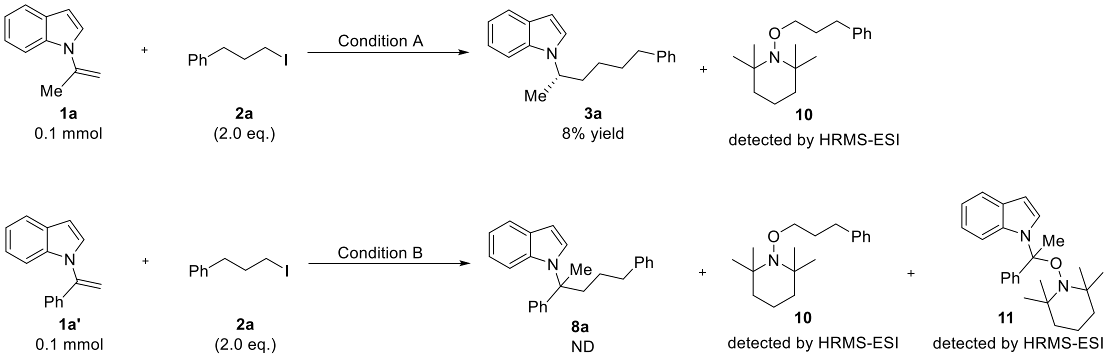

Supplementary Figure 15. Radical trapping experiment results

Condition A: To an oven-dried 10.0 mL Teflon-screw cap test tube containing a magnetic stir was charged with CoBr$_2$•DME (3.1 mg, 10 mol%) and ligand **L6** (11.3mg, 15 mol%) under a Argom atmosphere using glove-box techniques. Subsequently, anhydrous DME (0.5 mL) was added, and the mixture was stirred for 10 minutes at room temperature. Then, CsF (30.4 mg, 0.20 mmol, 2.0 equiv.), **1a** (0.10 mmol, 1.0 equiv), **2a** (0.20 mmol, 2.0 equiv.), TEMPO (23.4 mg, 0.15 mmol, 1.5 equiv) and (OEt)$_2$MeSiH (32 uL, 0.20 mmol, 2.0 equiv.) were sequentially added. Afterwards, the tube was sealed with airtight electrical tapes and removed from the glove box and stirred at 0 °C for 24 hours at 500 rpm. After the reaction was completed, the reaction mixture was diluted with saturated NH$_4$Cl (aq., 2.0 mL) and EtOAc (3.0 mL). The

aqueous phase was extracted with EtOAc (2 x 3.0 mL) and the combined organic phases were concentrated in vacuo. The crude mixture was purified by flash column chromatography on silica gel using PE as eluent to obtain the product **3a** (8% yield), **10** was determined by HRMS-ESI.

TEMPO was added under Condition B. Product **8a** was not detected, while **10** and **11** were detected by HRMS-ESI.

2,2,6,6-tetramethyl-1-(3-phenylpropoxy)piperidine

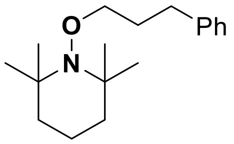

10

Conditon A: **HRMS** (ESI) m/z: $[M + H]^+$ Calcd for $C_{18}H_{30}NO^+$ 276.2322; Found 276.2322.

Condition B: **HRMS** (ESI) m/z: $[M + H]^+$ Calcd for $C_{18}H_{30}NO^+$ 276.2322; Found 276.2328.

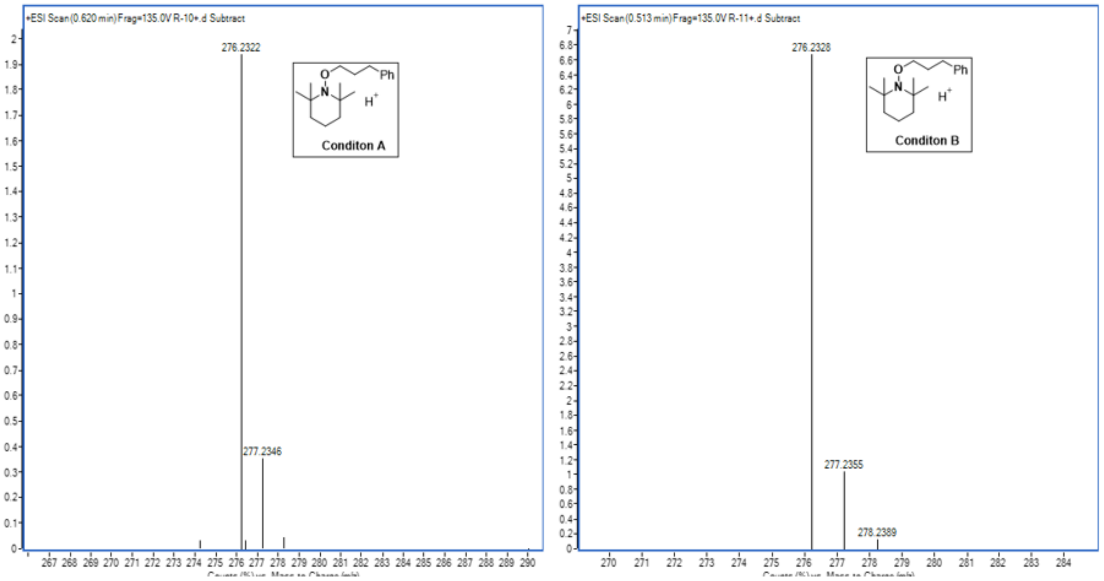

Supplementary Figure 16. HRMS results for **10**

1-(1-phenyl-1-((2,2,6,6-tetramethylpiperidin-1-yl)oxy)ethyl)-1*H*-indole

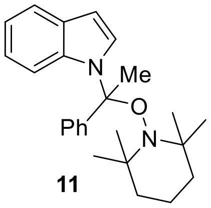

11

Condition B: **HRMS** (ESI) m/z: $[M + H]^+$ Calcd for $C_{25}H_{33}N_2O^+$ 377.2587; Found 377.2587.

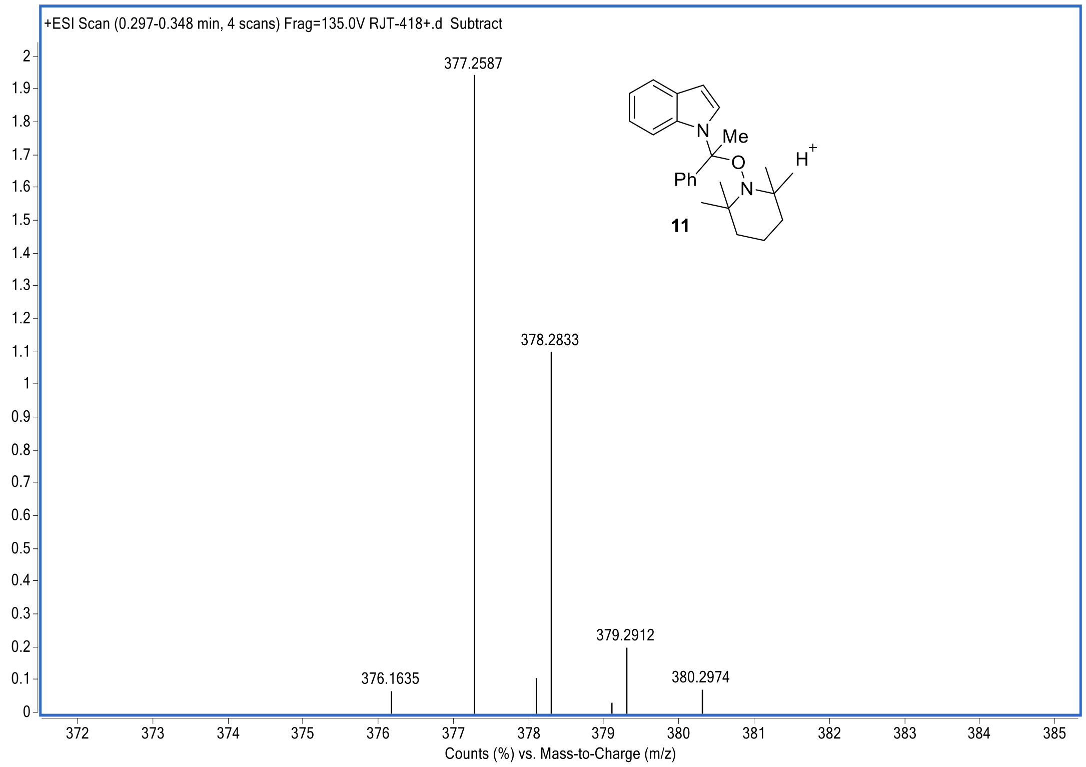

Supplementary Figure 17. HRMS results for **11**

9.4 Deuterium Labelling Experiment

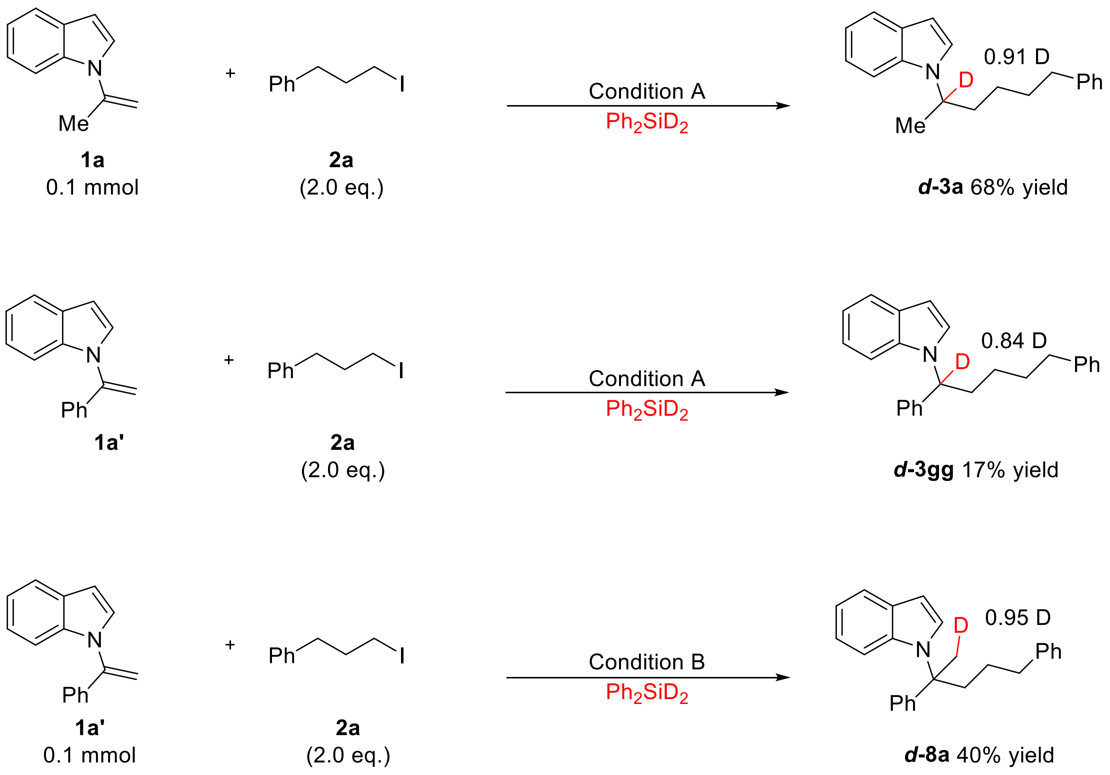

Supplementary Figure 18. Deuterium labelling experiment results

To an oven-dried 10.0 mL Teflon-screw cap test tube containing a magnetic stir was charged with $CoBr_2$•DME (3.1 mg, 10 mol%) and ligand **L6** (11.3mg, 15 mol%) under a Argon atmosphere using glove-box techniques. Subsequently, anhydrous DME (0.5 mL) was added, and the mixture was stirred for 10 minutes at room temperature. Then, CsF (30.4 mg, 0.20 mmol, 2.0 equiv.), **1a** (0.10 mmol, 1.0 equiv), **2a** (0.20 mmol, 2.0 equiv.) and Ph_2SiD_2 (40 uL, 0.20 mmol, 2.0 equiv.) were sequentially added. Afterwards, the tube was sealed with airtight electrical tapes and removed from the glove box and stirred at 0 °C for 24 hours at 500 rpm. After the reaction was completed, the reaction mixture was diluted with saturated NH_4Cl (aq., 2.0 mL) and EtOAc (3.0 mL). The aqueous phase was extracted with EtOAc (2 x 3.0 mL) and the combined organic phases were concentrated in vacuo. The crude mixture was purified by flash column chromatography on silica gel using PE as eluent to obtain the product ***d*-3a** (68% yield).

1-(6-phenylhexan-2-yl-2-*d*)-1*H*-indole (*d*-3a)

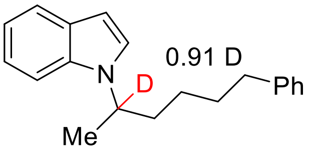

***d*-3a**

^{1}H NMR (500 MHz, $CDCl_3$) δ 7.62 (d, J = 7.9 Hz, 1H), 7.35 (d, J = 8.3 Hz, 1H), 7.25-7.22 (m, 2H), 7.20-7.13 (m, 3H), 7.09-7.07 (m, 3H), 6.52 (d, J = 3.2 Hz, 1H), 4.50-4.43 (m, 0.09H), 2.56-2.47 (m, 2H), 1.96-1.90 (m, 1H), 1.84-1.78 (m, 1H), 1.63-1.54 (m, 2H), 1.48 (s, 3H), 1.35-1.19 (m, 2H).

To an oven-dried 10.0 mL Teflon-screw cap test tube containing a magnetic stir was charged with $CoBr_2$•DME (3.1 mg, 10 mol%) and ligand **L6** (11.3mg, 15 mol%) under a Argon atmosphere using glove-box techniques. Subsequently, anhydrous DME (0.5 mL) was added, and the mixture was stirred for 10 minutes at room temperature. Then, CsF (30.4 mg, 0.20 mmol, 2.0 equiv.), **1a'** (0.10 mmol, 1.0 equiv), **2a** (0.20 mmol, 2.0 equiv.) and Ph_2SiD_2 (40 uL, 0.20 mmol, 2.0 equiv.) were sequentially added. Afterwards, the tube was sealed with airtight electrical tapes and removed from the glove box and stirred at 0 °C for 24 hours at 500 rpm. After the reaction was completed, the reaction

mixture was diluted with saturated NH_4Cl (aq., 2.0 mL) and EtOAc (3.0 mL). The aqueous phase was extracted with EtOAc (2 x 3.0 mL) and the combined organic phases were concentrated in vacuo. The crude mixture was purified by flash column chromatography on silica gel using PE as eluent to obtain the product ***d*-3gg** (17% yield).

1-(1,5-diphenylpentyl-1-*d*)-1*H*-indole (*d*-3gg)

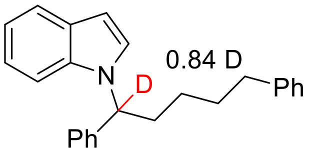

***d*-3gg**

^{1}H NMR (500 MHz, $CDCl_3$) δ 7.62 (d, *J* = 7.7 Hz, 1H), 7.29-7.26 (m, 3H), 7.25-7.21 (m, 3H), 7.19-7.13 (m, 4H), 7.11-7.06 (m, 4H), 6.56 (d, *J* = 3.2 Hz, 1H), 5.43 (dd, *J* = 9.0, 6.5 Hz, 0.16H), 2.58-2.54 (m, 2H), 2.39-2.33 (m, 1H), 2.30-2.24 (m, 1H), 1.72-1.64 (m, 2H), 1.42-1.36 (m, 2H).

To an oven-dried 10.0 mL Teflon-screw cap test tube containing a magnetic stir was charged with CoI_2 (3.1 mg, 10 mol%) and ligand **L12** (4.7 mg, 12 mol%) under a Argon atmosphere using glove-box techniques. Subsequently, anhydrous THF (0.5 mL) was added, and the mixture was stirred for 10 minutes at room temperature. Then, CsF (30.4 mg, 0.20 mmol, 2.0 equiv.), **1a'** (0.10 mmol, 1.0 equiv), **2a** (0.20 mmol, 2.0 equiv.) and Ph_2SiD_2 (40 uL, 0.20 mmol, 2.0 equiv.) were sequentially added. Afterwards, the tube was sealed with airtight electrical tapes and removed from the glove box and stirred at 40 °C for 24 hours at 500 rpm. After the reaction was completed, the reaction mixture was diluted with saturated NH_4Cl (aq., 2.0 mL) and EtOAc (3.0 mL). The aqueous phase was extracted with EtOAc (2 x 3.0 mL) and the combined organic phases were concentrated in vacuo. The crude mixture was purified by flash column chromatography on silica gel using PE as eluent to obtain the product ***d*-8a (**40% yield).

1-(2,5-diphenylpentan-2-yl-1-*d*)-1*H*-indole (*d*-8a)

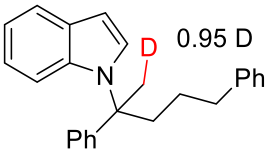

***d*-8a**

^{1}**H NMR (500 MHz, CDCl**$_3$**)** δ 7.59 (d, *J* = 7.9 Hz, 1H), 7.43 (d, *J* = 3.3 Hz, 1H), 7.25-7.19 (m, 5H), 7.11-7.10 (m, 3H), 7.00-6.97 (m, 3H), 6.84-6.81 (m, 1H), 6.58 (d, *J* = 8.4 Hz, 1H), 6.53 (d, *J* = 2.8 Hz, 1H), 2.59 (td, *J* = 12.8, 4.5 Hz, 1H), 2.50 (t, *J* = 7.5 Hz, 2H), 2.30 (td, *J* = 12.7, 4.3 Hz, 1H), 1.87 (m, 2.05H), 1.60-1.51 (m, 1H), 1.13-1.04 (m, 1H).

9.5 UV-Visible Experiment

Following Condition A, the reaction was run on a 0.20 mmol scale based on the **1a** & **2a**, For UV-vis analysis, the reaction mixture solution (100 μL) was taken through a syringe and dissolved in 4mL of DME. The insoluble material was filtered by using a syringe filter, the filtrate was poured into a colorimetric dish to test ultraviolet-visible spectroscopy. The yield was determined via ^{1}H NMR analysis with 1,3,5-trimethoxybenzene as the internal standard.

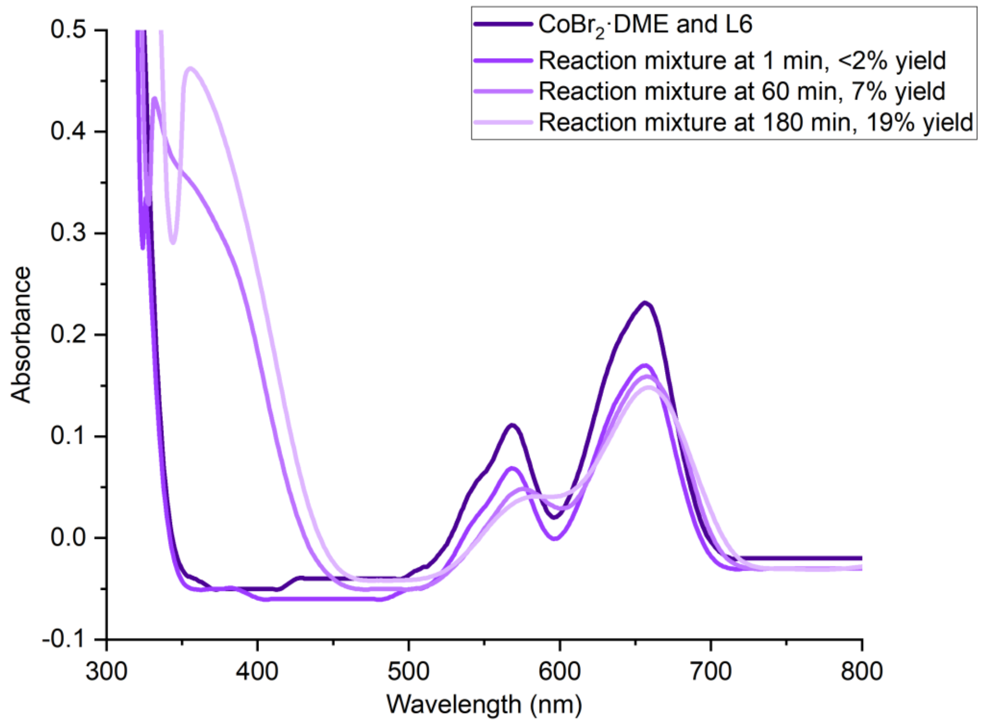

Supplementary Figure 19. UV-Visible spectroscopy of reaction mixture solution (**1a** & **2a**)

10. Computational details

All of the calculations were performed using the Gaussian 16 program[21]. Structures were optimized at the (U)B3LYP level of density functional theory[22] with Grimme's D3(BJ) dispersion correction[23] in gas phase. For optimizations, Ahlrichs's def2SVP basis set was used for all atoms[24]. Frequency calculations have been performed to verify the optimized structures as local minima or transition state and to obtain Gibbs free energy at 298 K. Intrinsic reaction coordinate (IRC) calculations were carried out to make sure that every transition state links relevant intermediates[25]. The electronic energies were further refined by carrying out single-point energy calculations using (U)TPSSh functional[26,27] with Grimme's D3(BJ) dispersion correction. This functional has been shown to give accurate energy differences among different spin states of first-row transition metal complexes.[28-31] The def2TZVP basis set was applied for all atoms[24]. The PCM solvation model with THF as the solvent was employed to account for solvation effect[32]. The three-dimensional (3D) structures were depicted using CYLview software.

10.1 Additional Computational Results

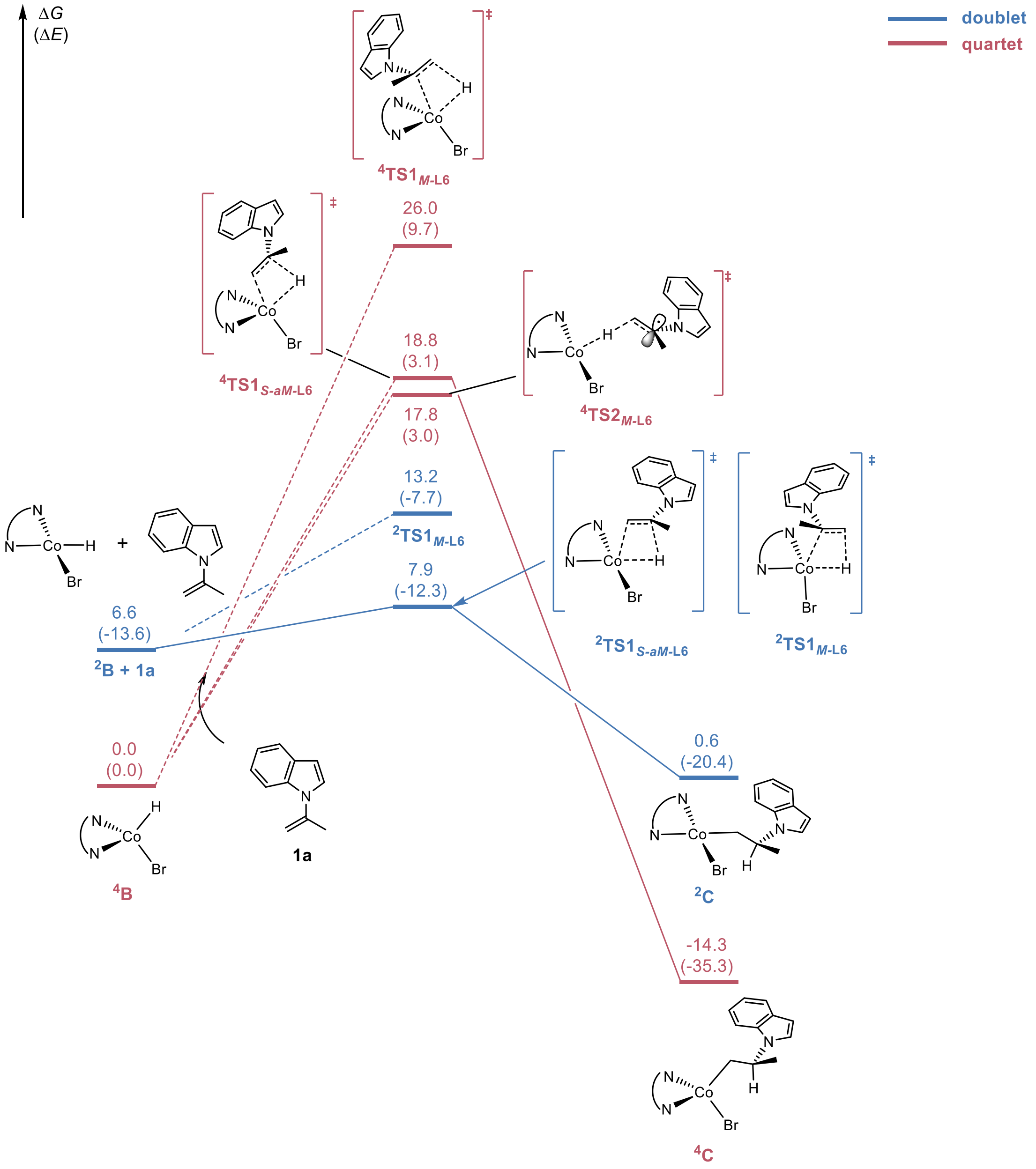

Supplementary Figure 20. Energy profiles on the reaction between **L6**Co–H complex and alkene **1a** in doublet state and quartet state. All energies are given in kcal/mol.

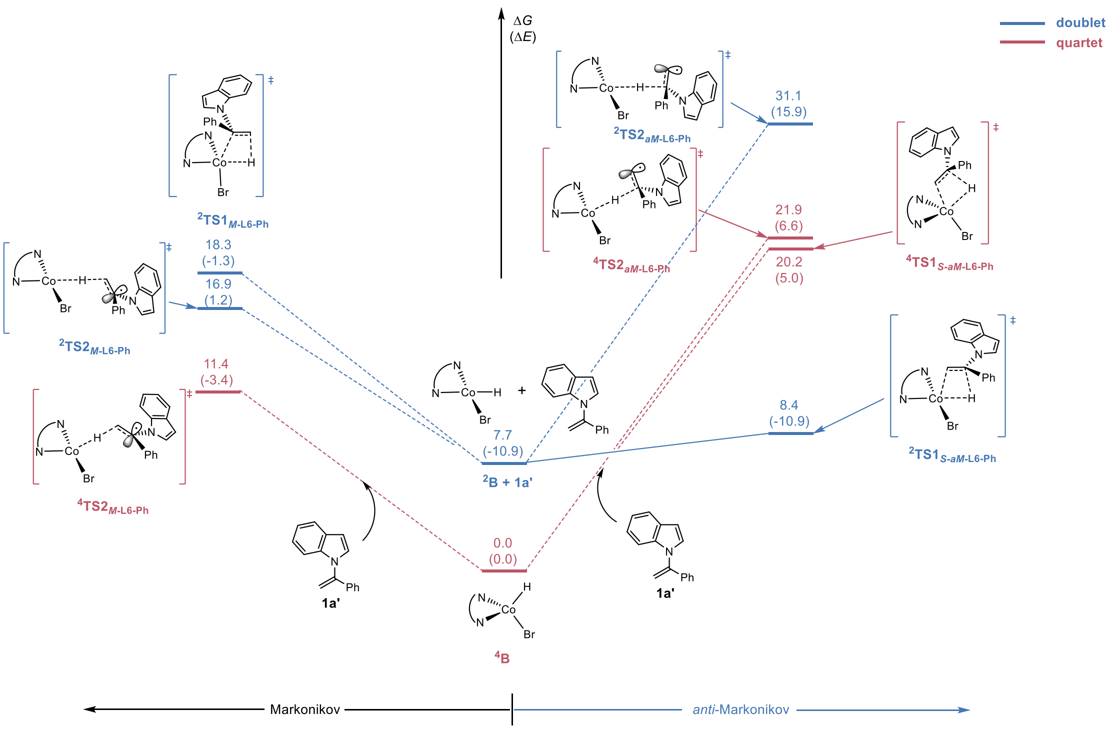

Supplementary Figure 21. Energy profiles on the reaction between **L6**Co–H complex and alkene **1a'** in doublet state and quartet state. All energies are given in kcal/mol.

11. NMR and HPLC Spectra

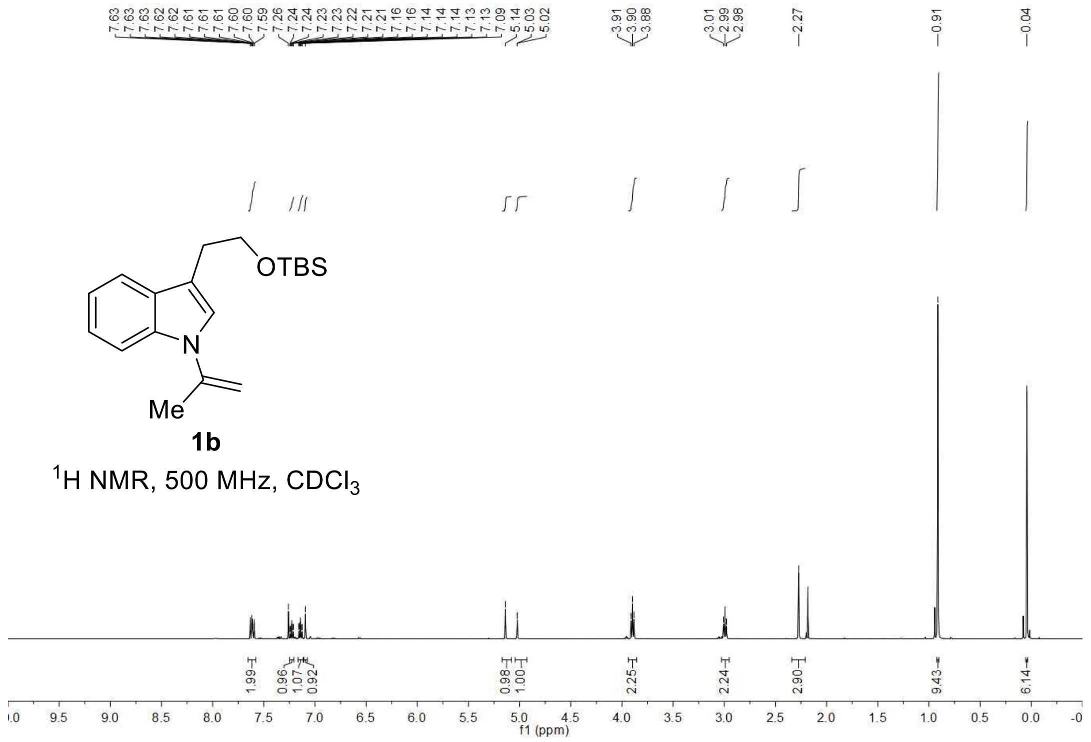

Supplementary Figure 22. ^{1}H NMR spectrum of compound **1b**

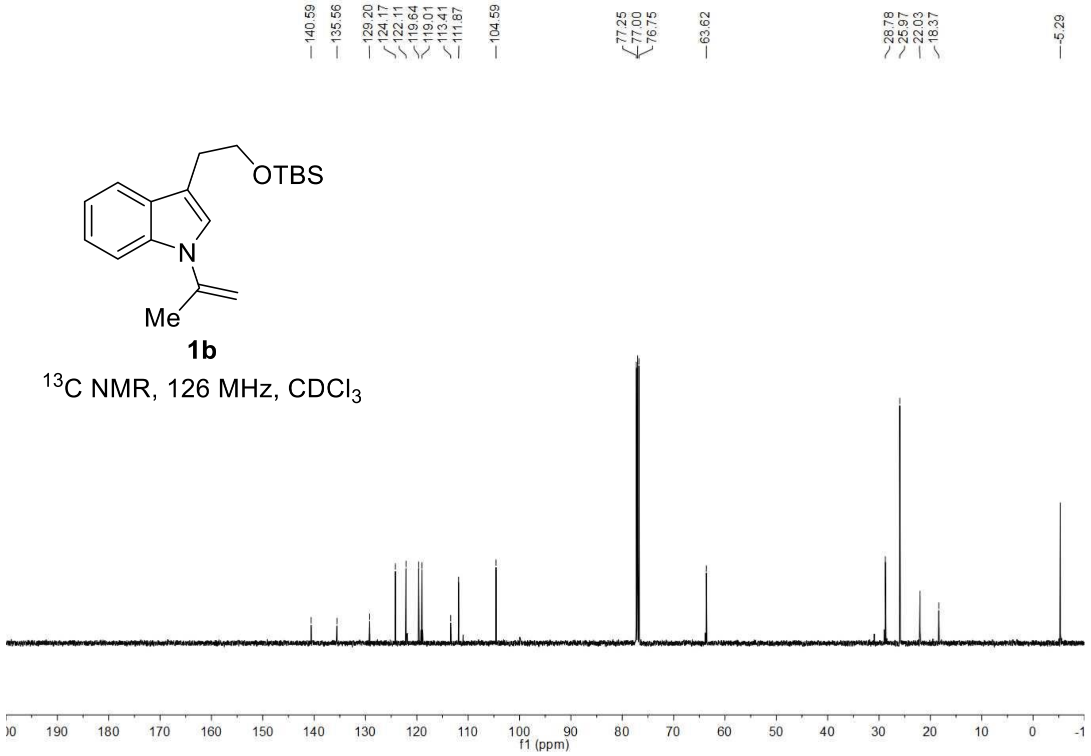

Supplementary Figure 23. ^{13}C NMR spectrum of compound **1b**

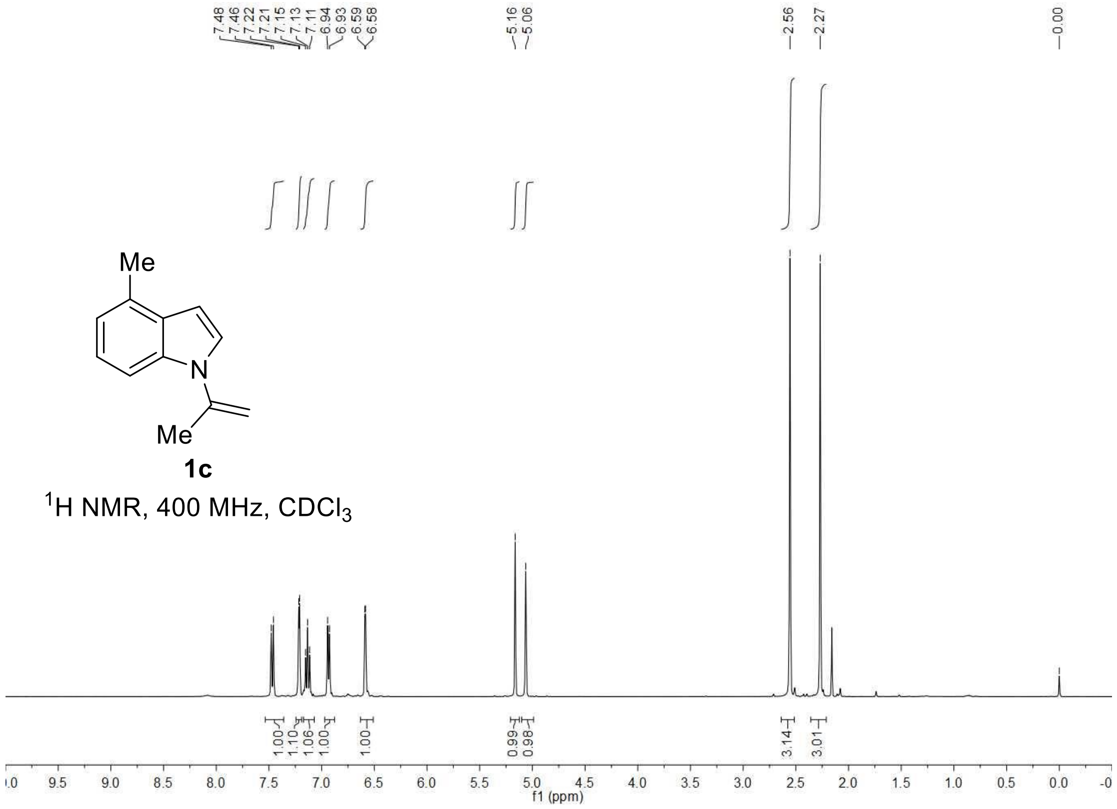

Supplementary Figure 24. ^{1}H NMR spectrum of compound **1c**

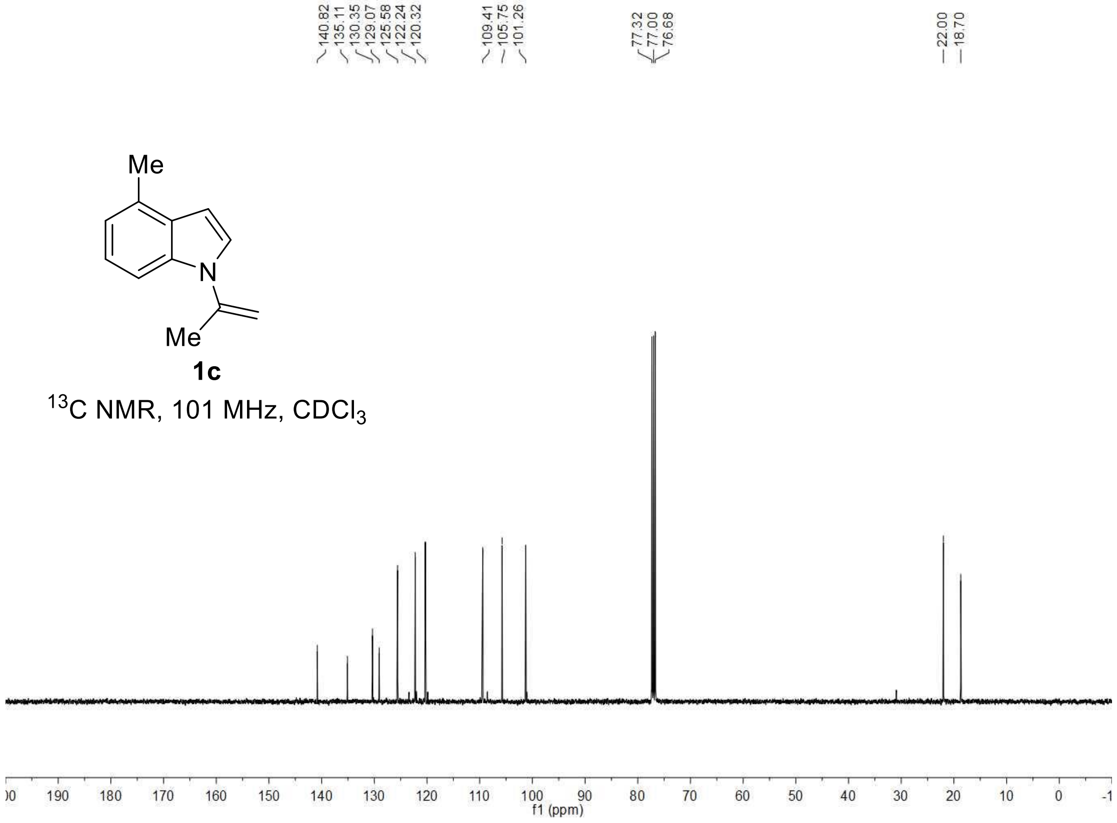

Supplementary Figure 25. ^{13}C NMR spectrum of compound **1c**

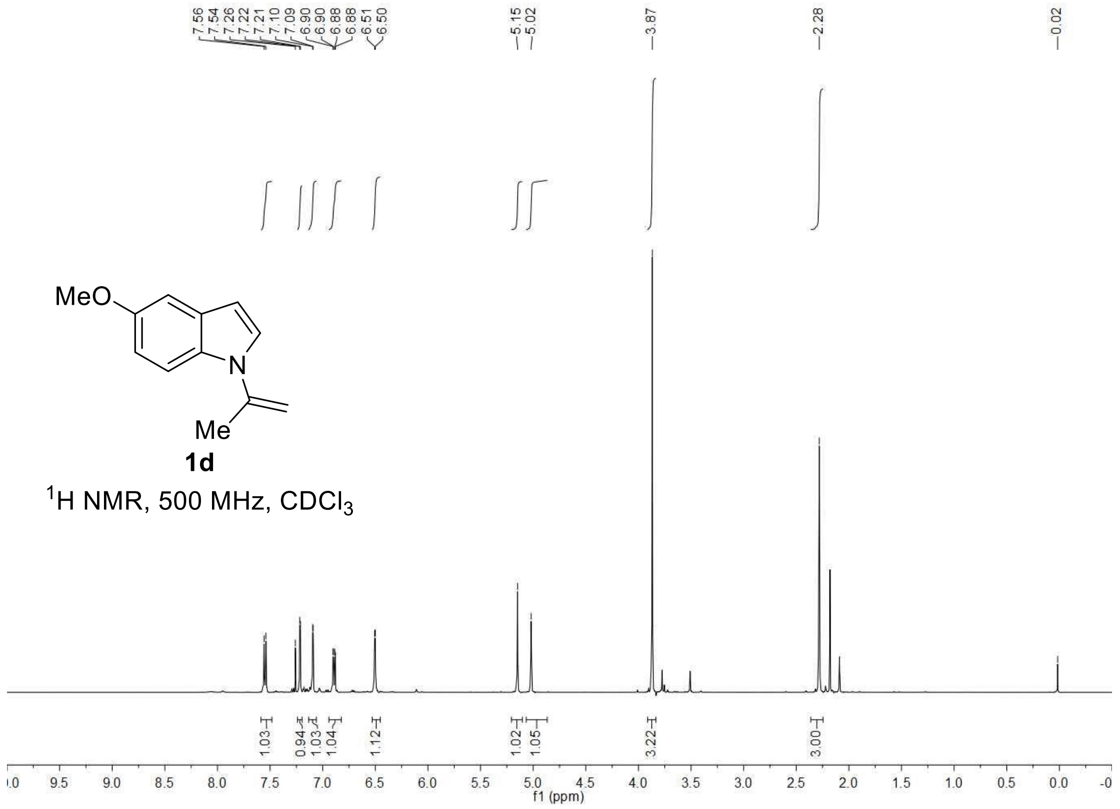

Supplementary Figure 26. ^{1}H NMR spectrum of compound **1d**

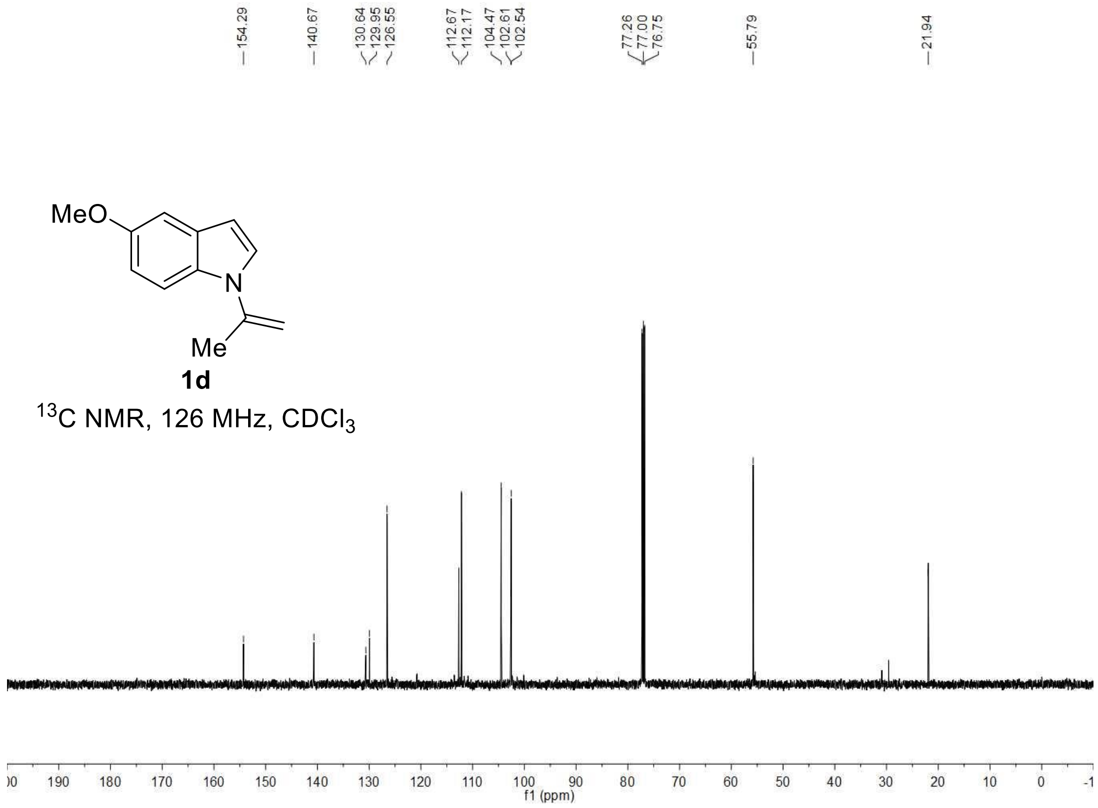

Supplementary Figure 27. ^{13}C NMR spectrum of compound **1d**

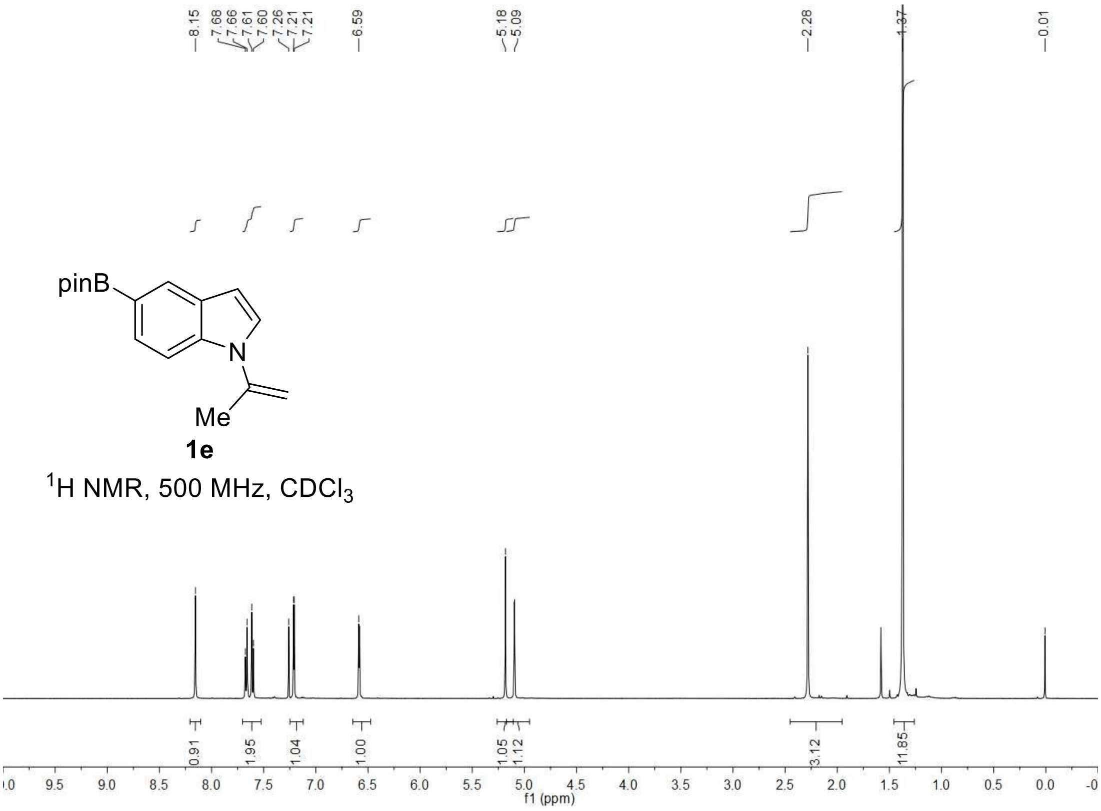

Supplementary Figure 28. ^{1}H NMR spectrum of compound **1e**

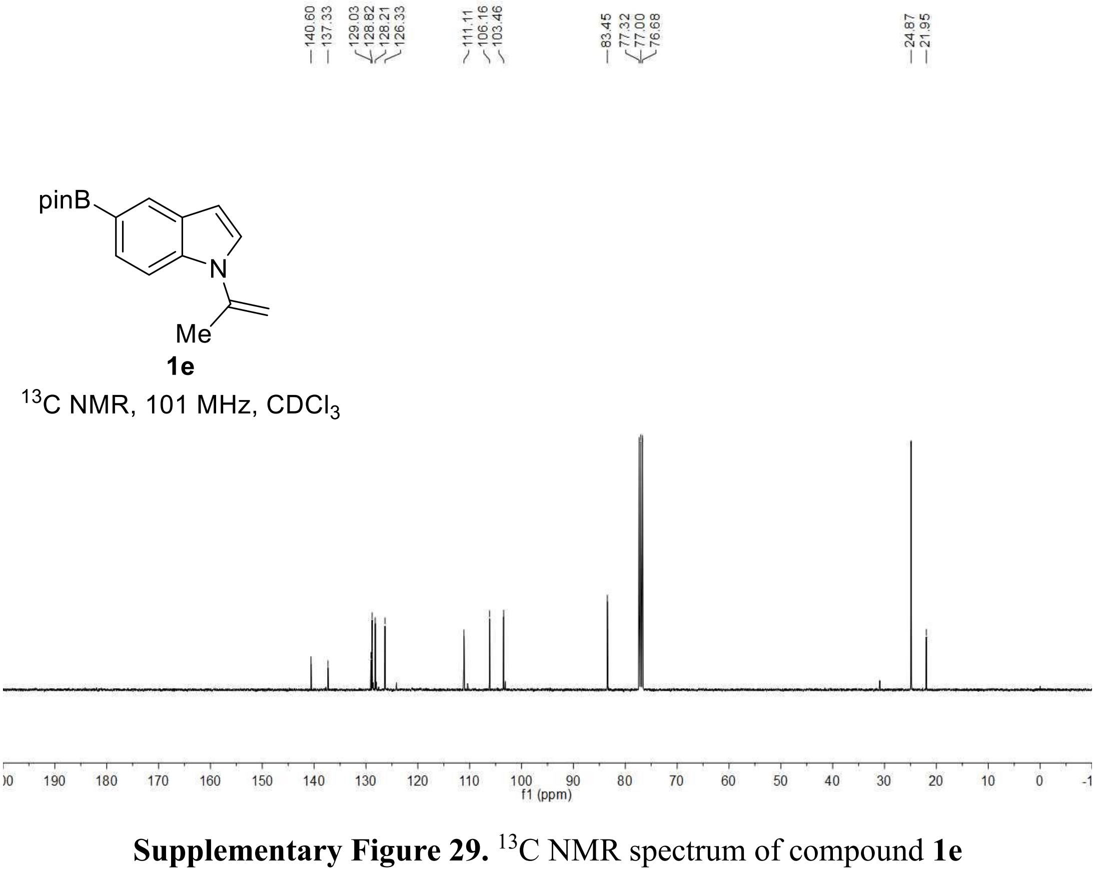

Supplementary Figure 29. ^{13}C NMR spectrum of compound **1e**

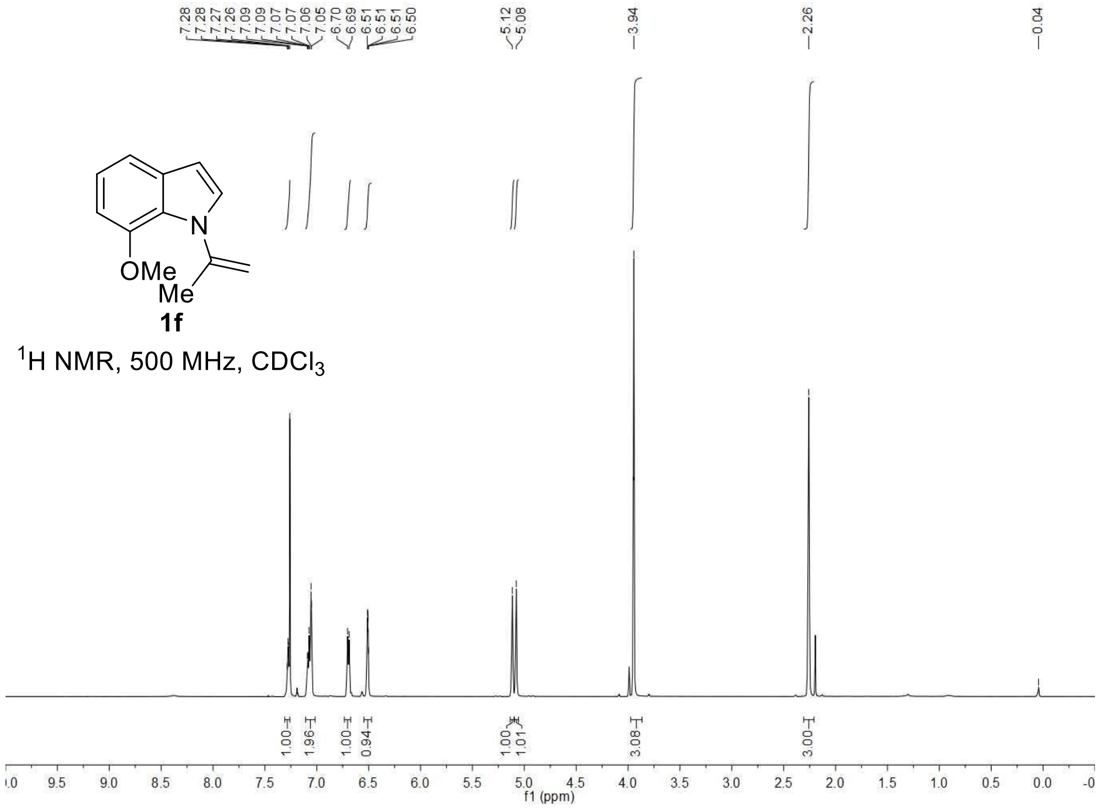

Supplementary Figure 30. ^{1}H NMR spectrum of compound **1f**

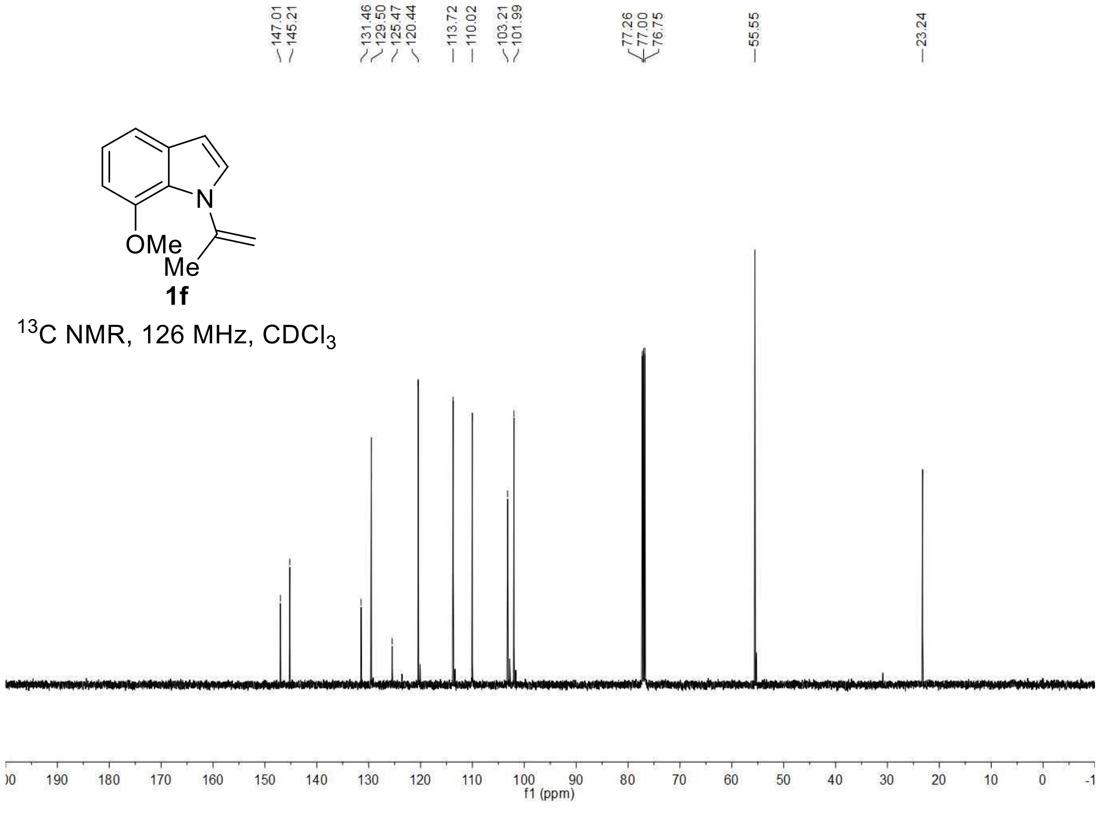

Supplementary Figure 31. ^{13}C NMR spectrum of compound **1f**

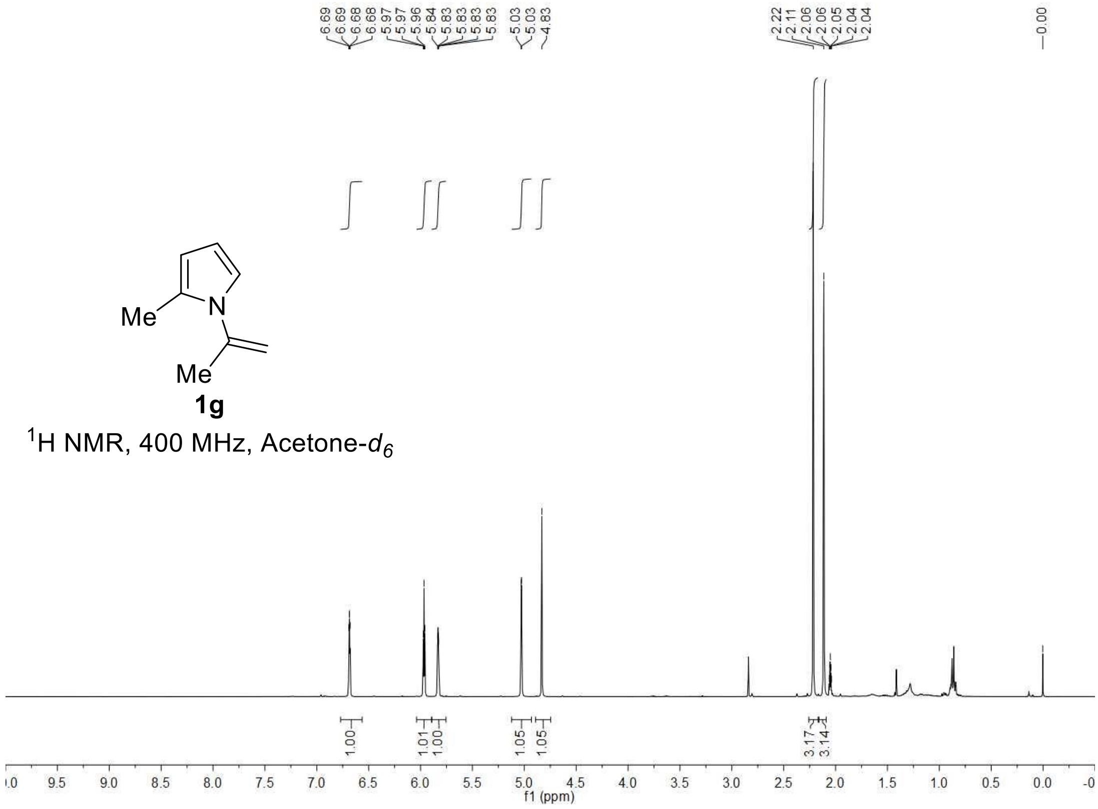

Supplementary Figure 32. ^{1}H NMR spectrum of compound **1g**

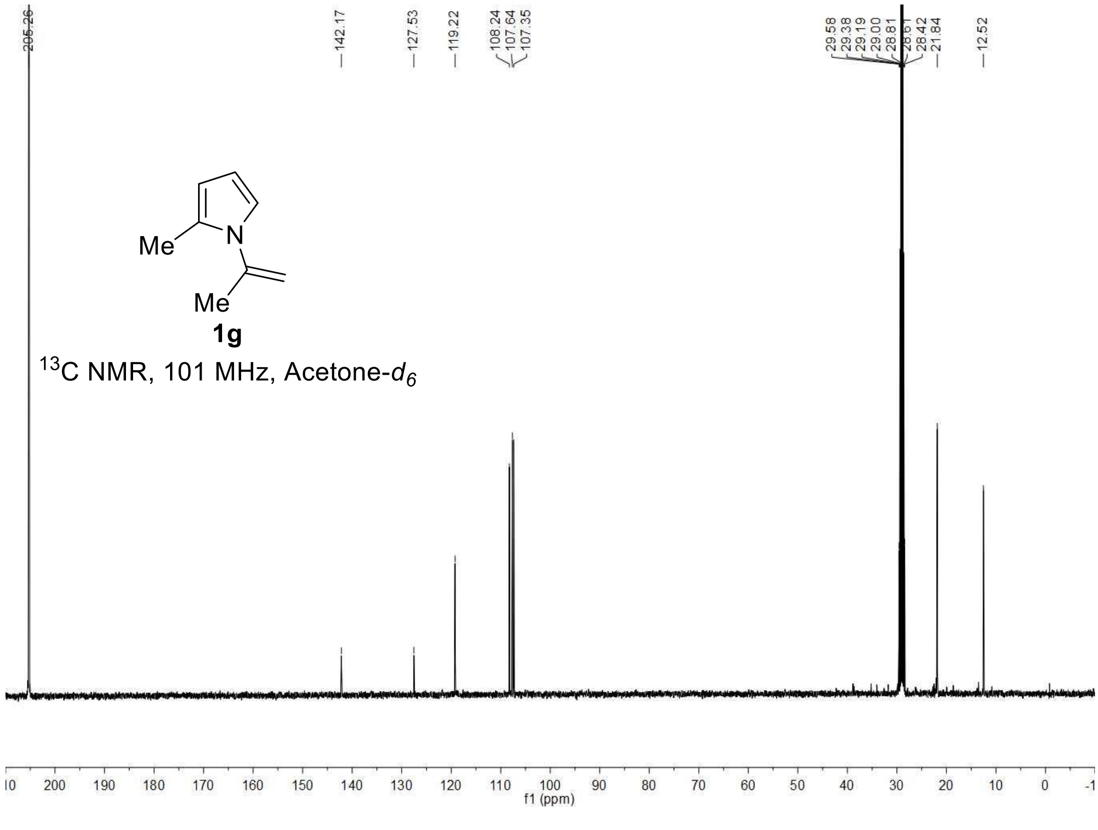

Supplementary Figure 33. ^{13}C NMR spectrum of compound **1g**

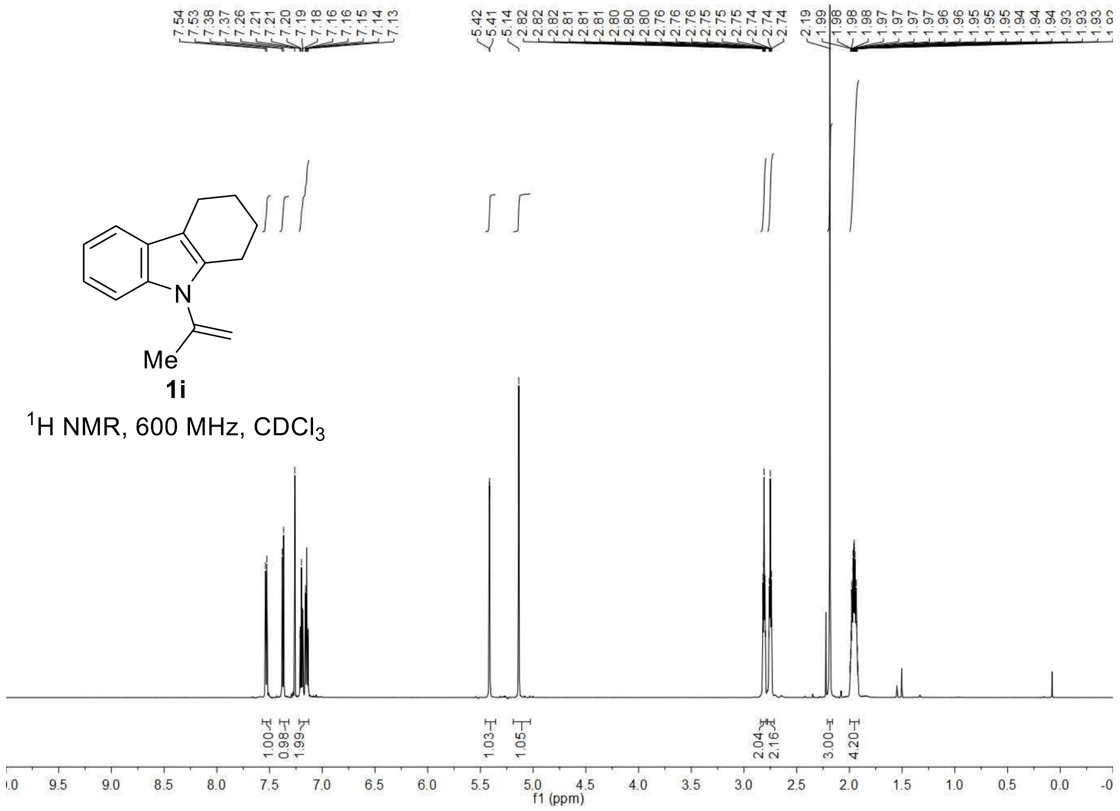

Supplementary Figure 34. ^{1}H NMR spectrum of compound **1i**

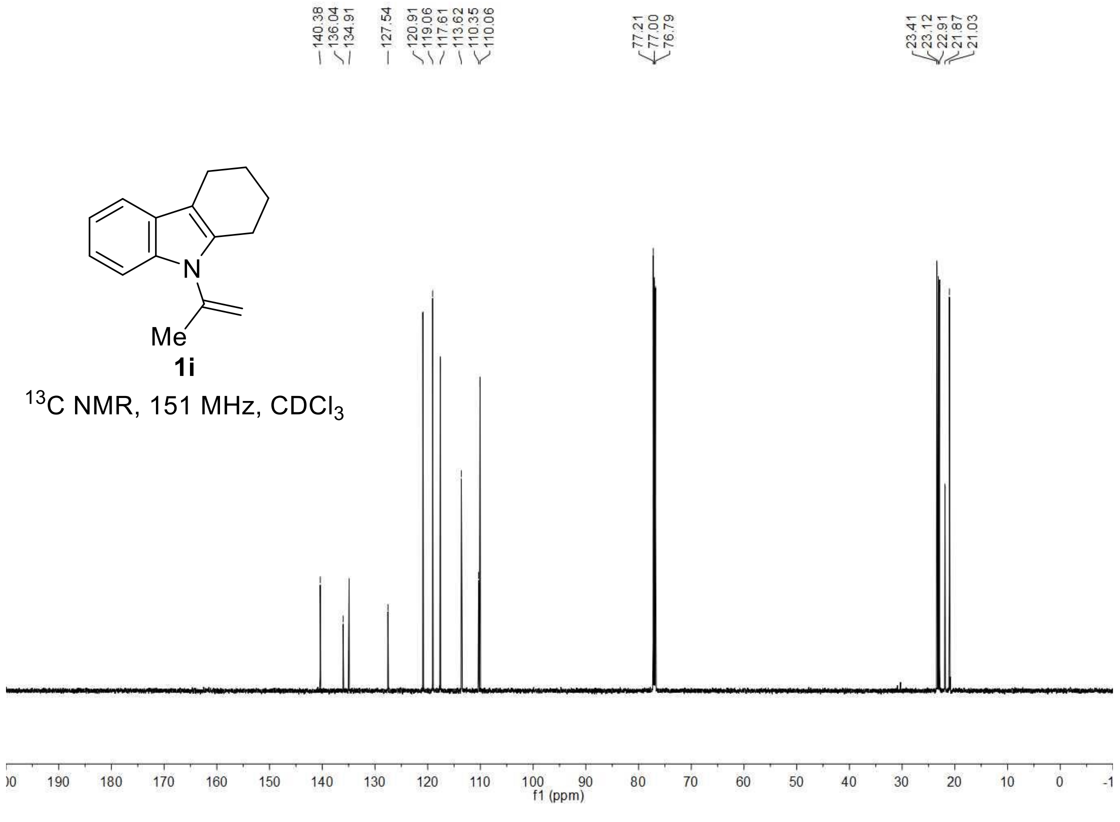

Supplementary Figure 35. ^{13}C NMR spectrum of compound **1i**

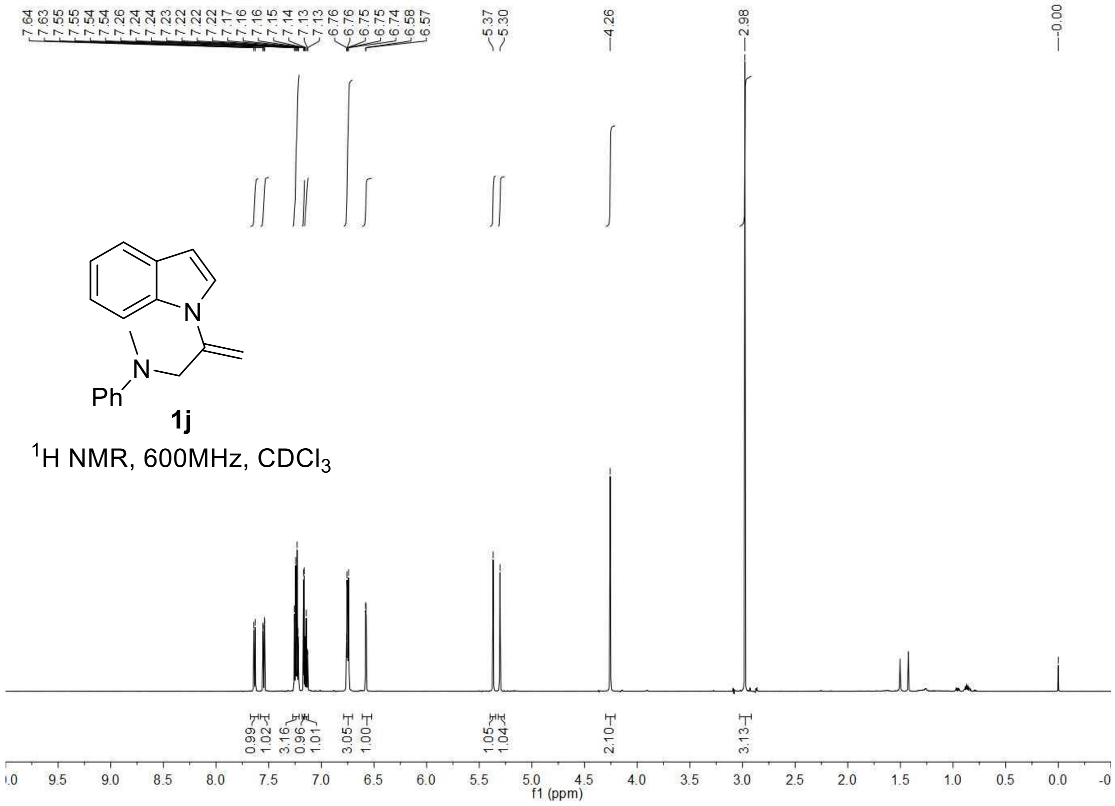

Supplementary Figure 36. ^{1}H NMR spectrum of compound **1j**

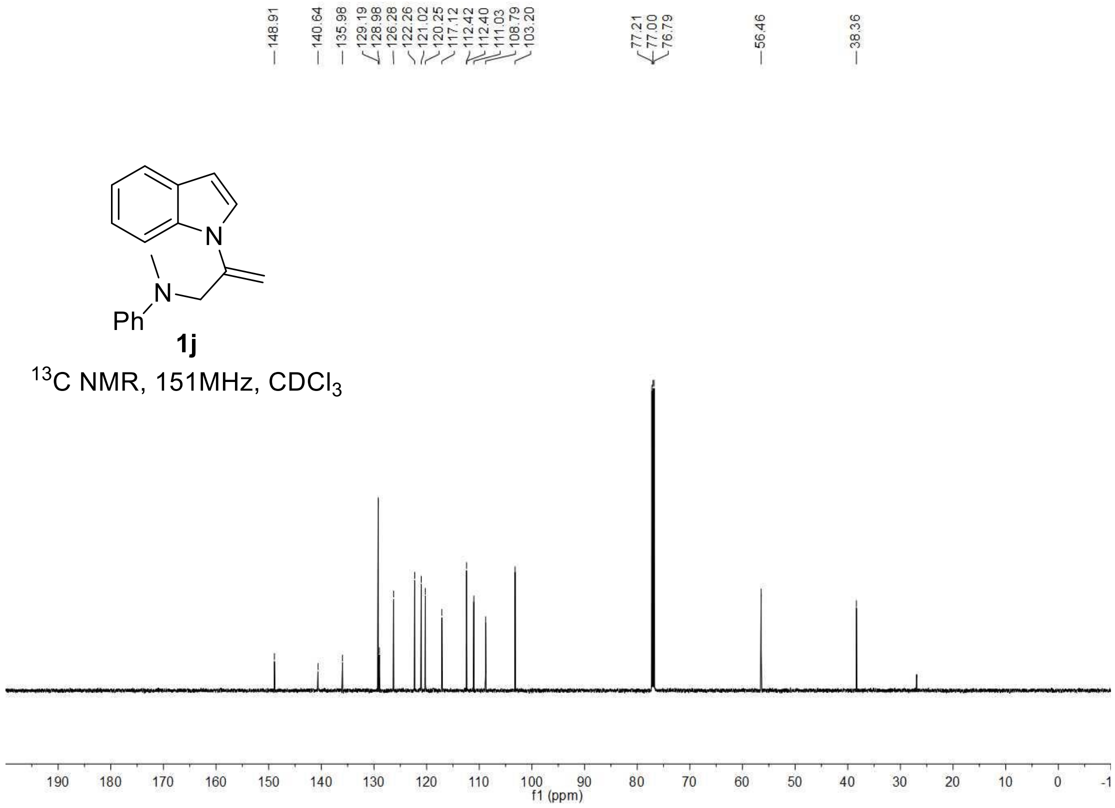

Supplementary Figure 37. ^{13}C NMR spectrum of compound **1j**

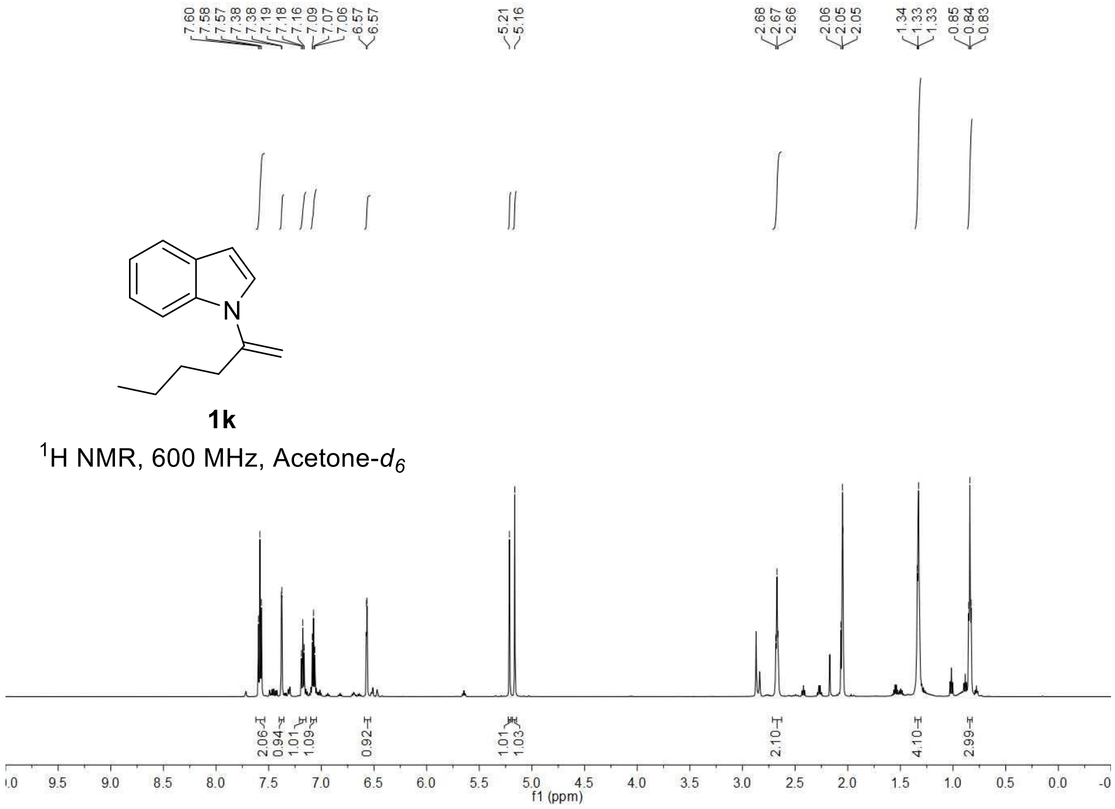

Supplementary Figure 38. ^{1}H NMR spectrum of compound **1k**

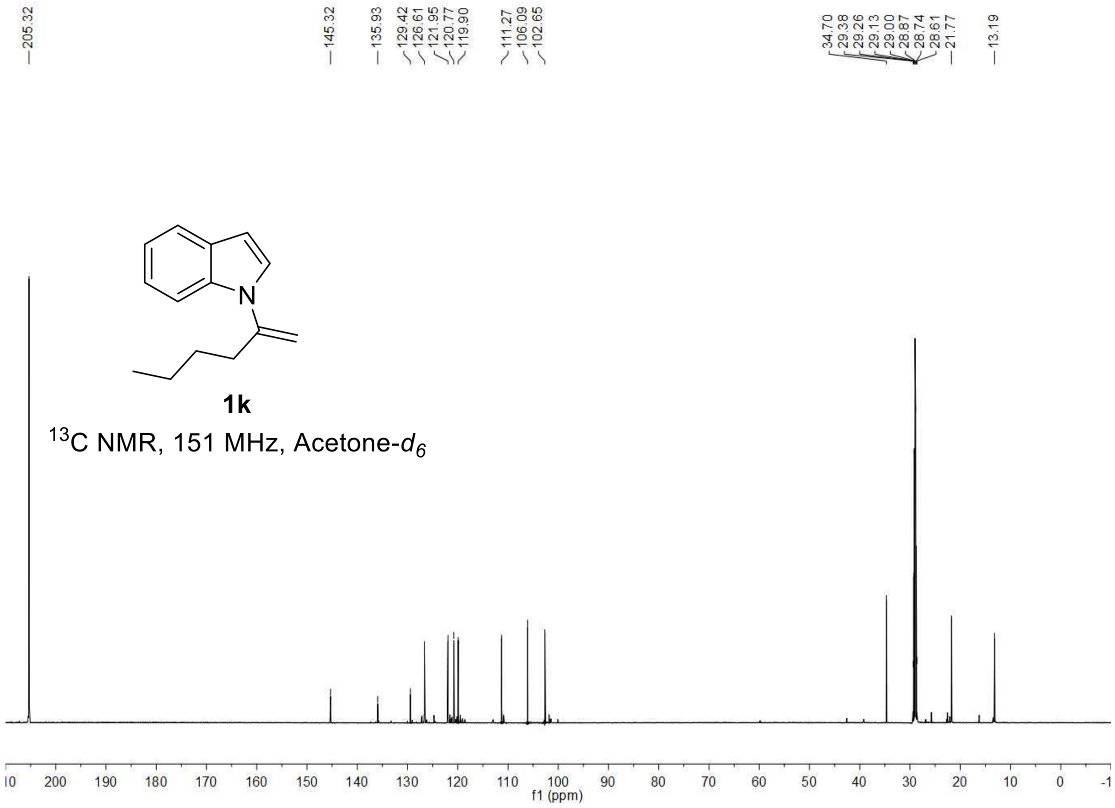

Supplementary Figure 39. ^{13}C NMR spectrum of compound **1k**

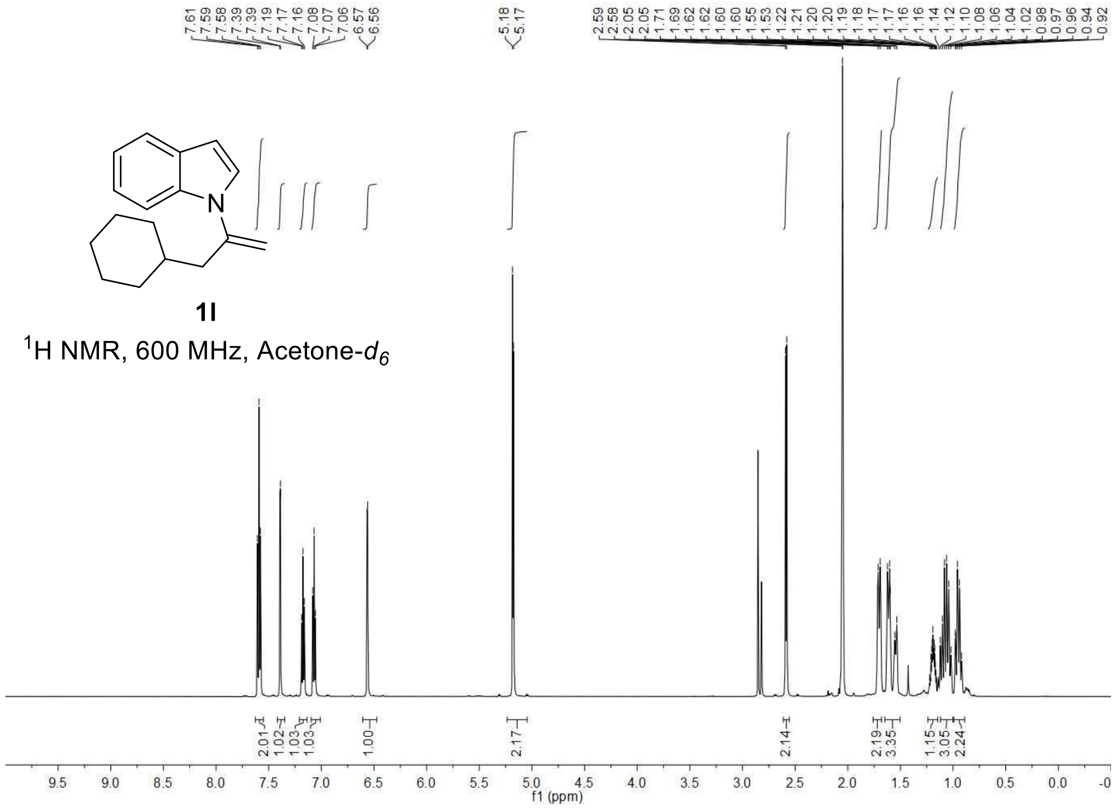

Supplementary Figure 40. ^{1}H NMR spectrum of compound **1l**

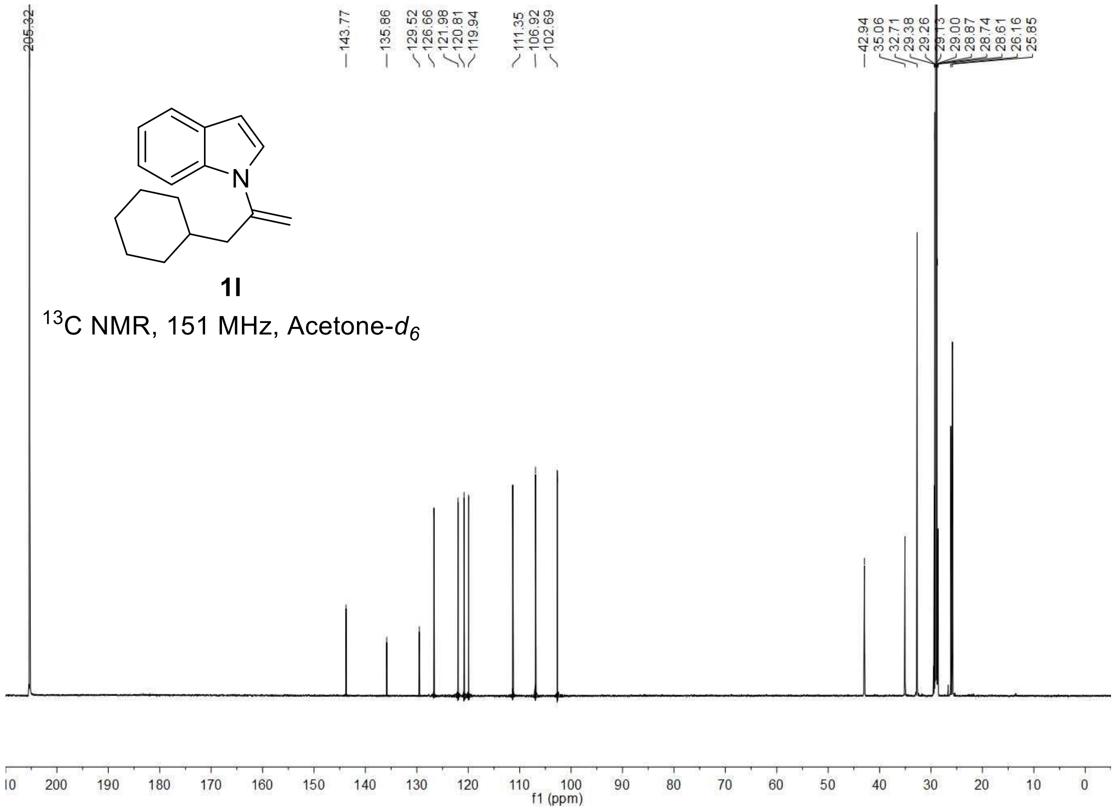

Supplementary Figure 41. ^{13}C NMR spectrum of compound **1l**

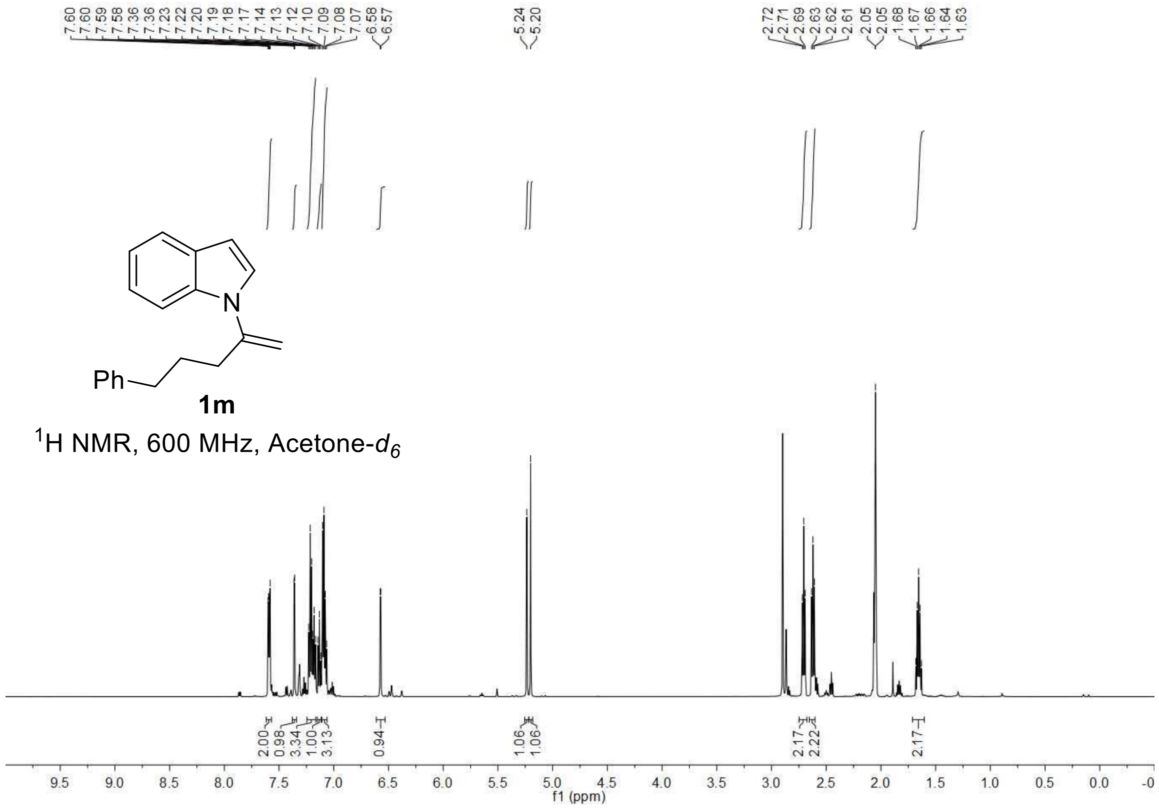

Supplementary Figure 42. ^{1}H NMR spectrum of compound **1m**

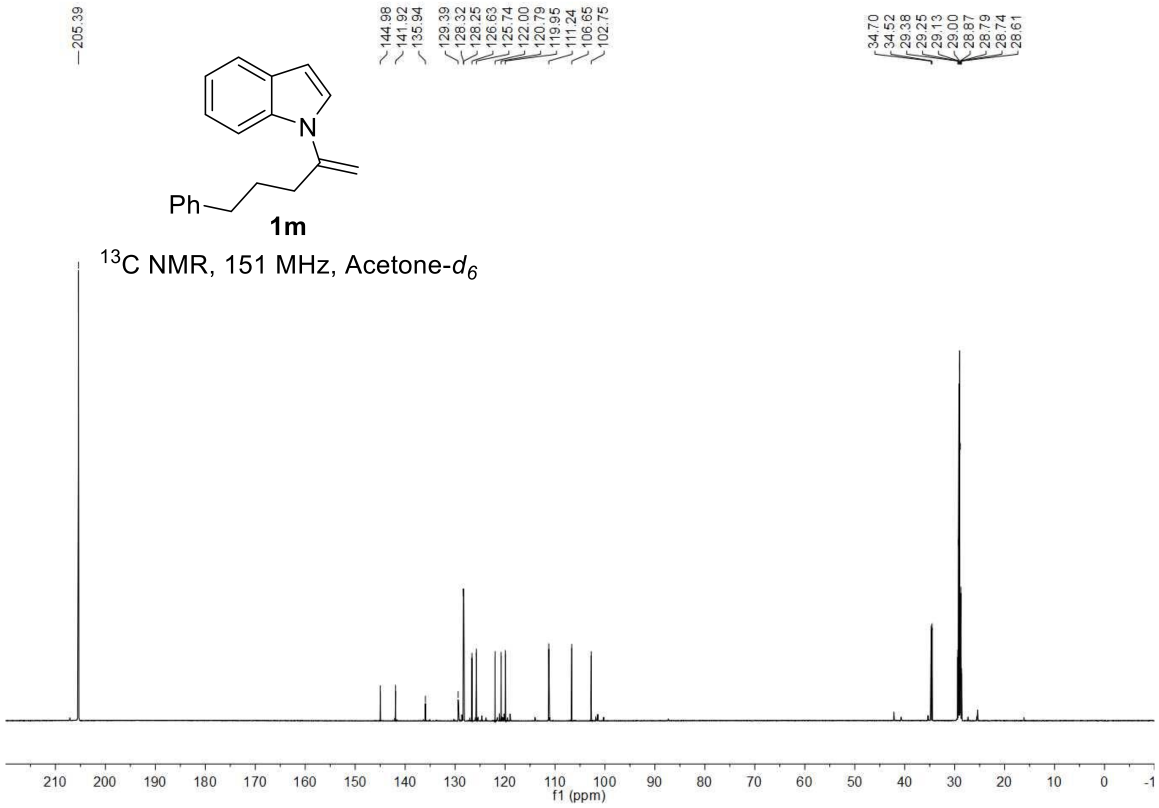

Supplementary Figure 43. ^{13}C NMR spectrum of compound **1m**

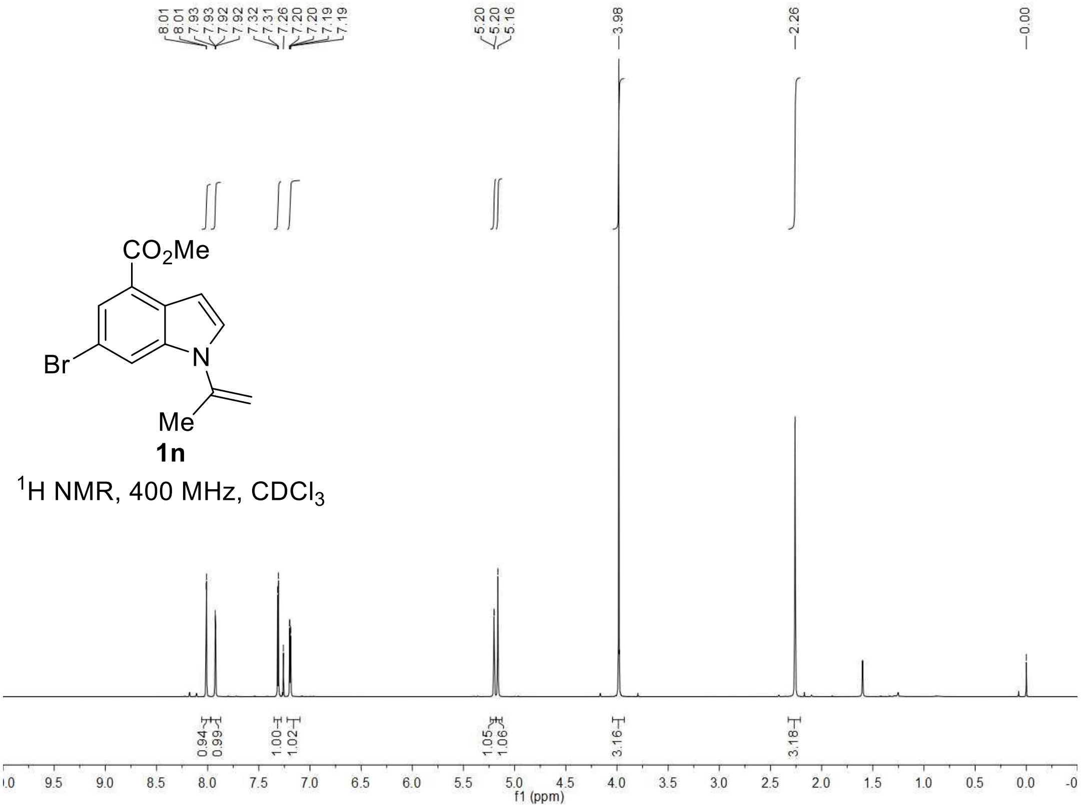

Supplementary Figure 44. ^{1}H NMR spectrum of compound **1n**

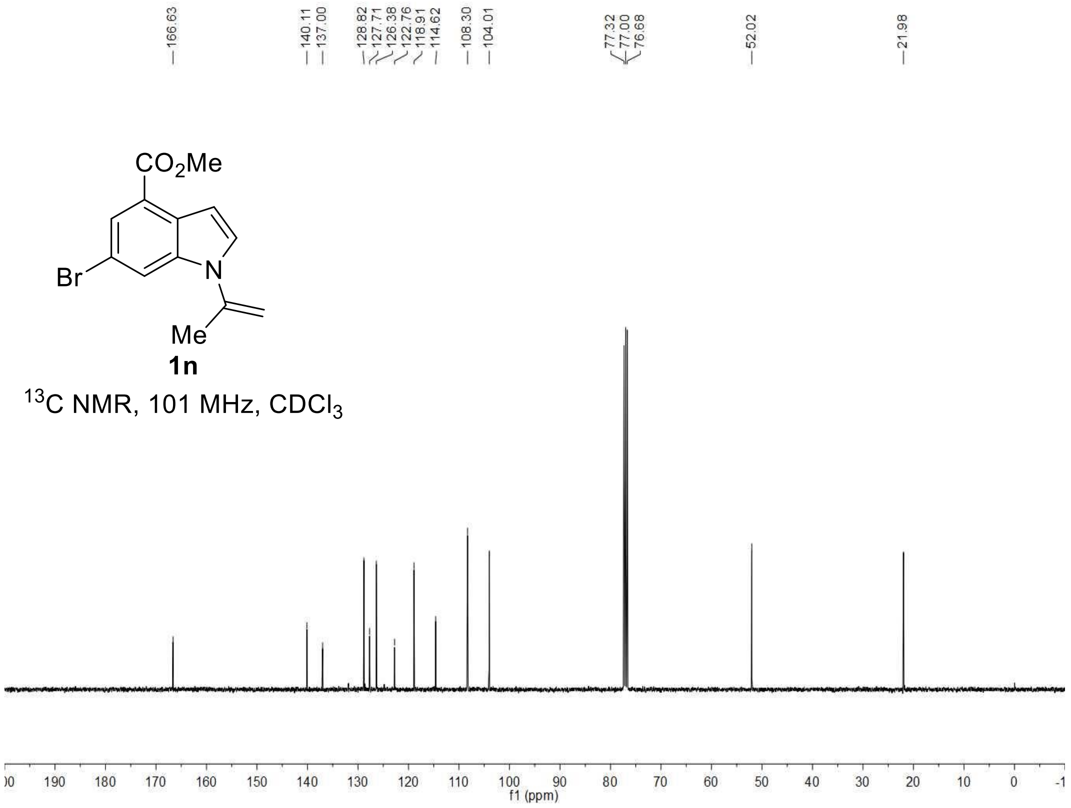

Supplementary Figure 45. ^{13}C NMR spectrum of compound **1n**

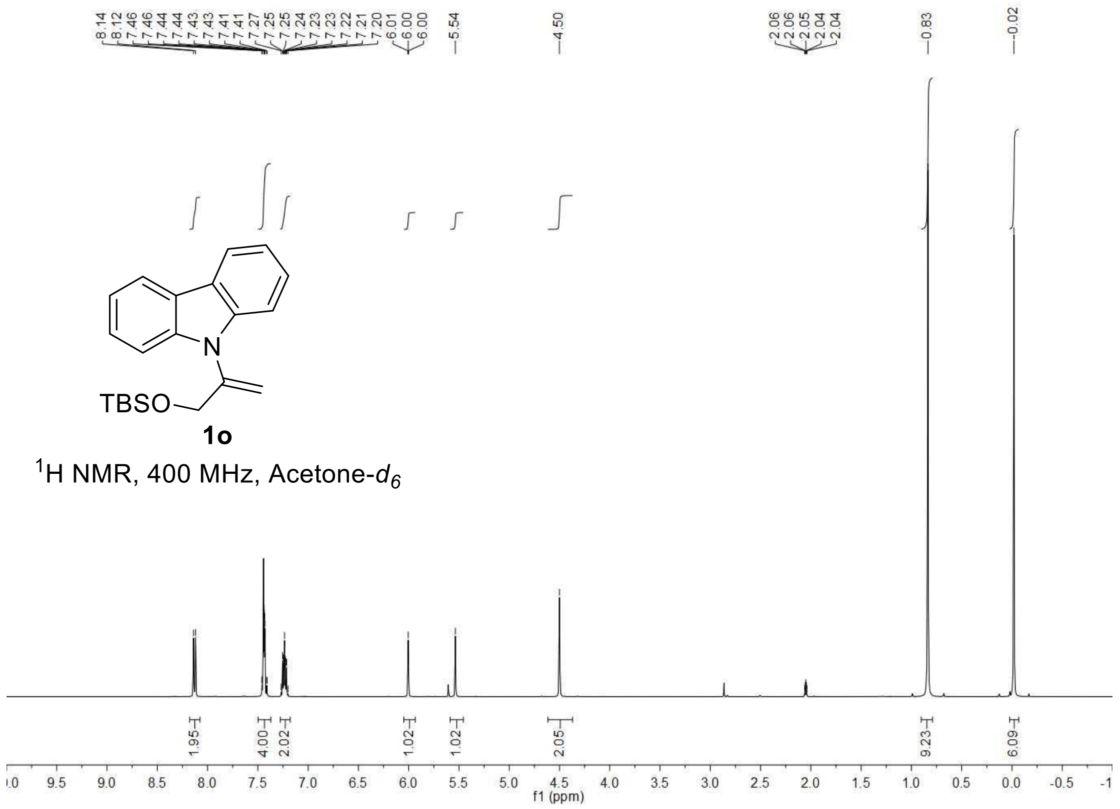

Supplementary Figure 46. ^{1}H NMR spectrum of compound **1o**

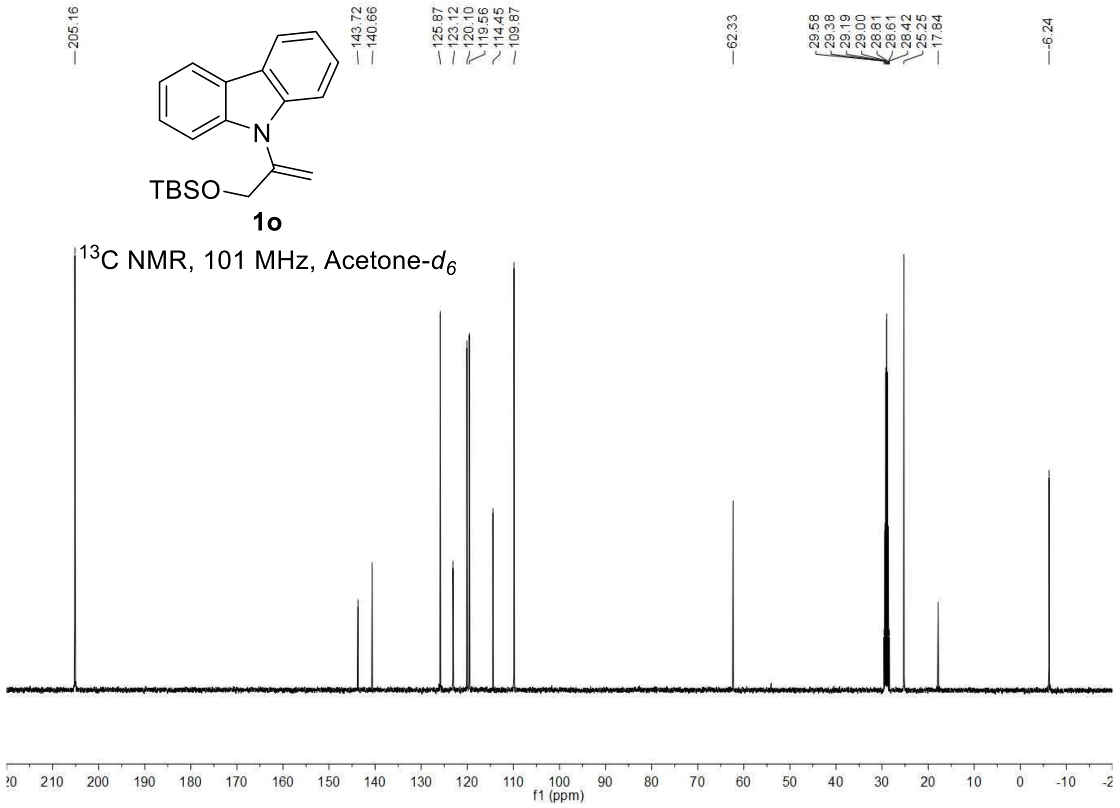

Supplementary Figure 47. ^{13}C NMR spectrum of compound **1o**

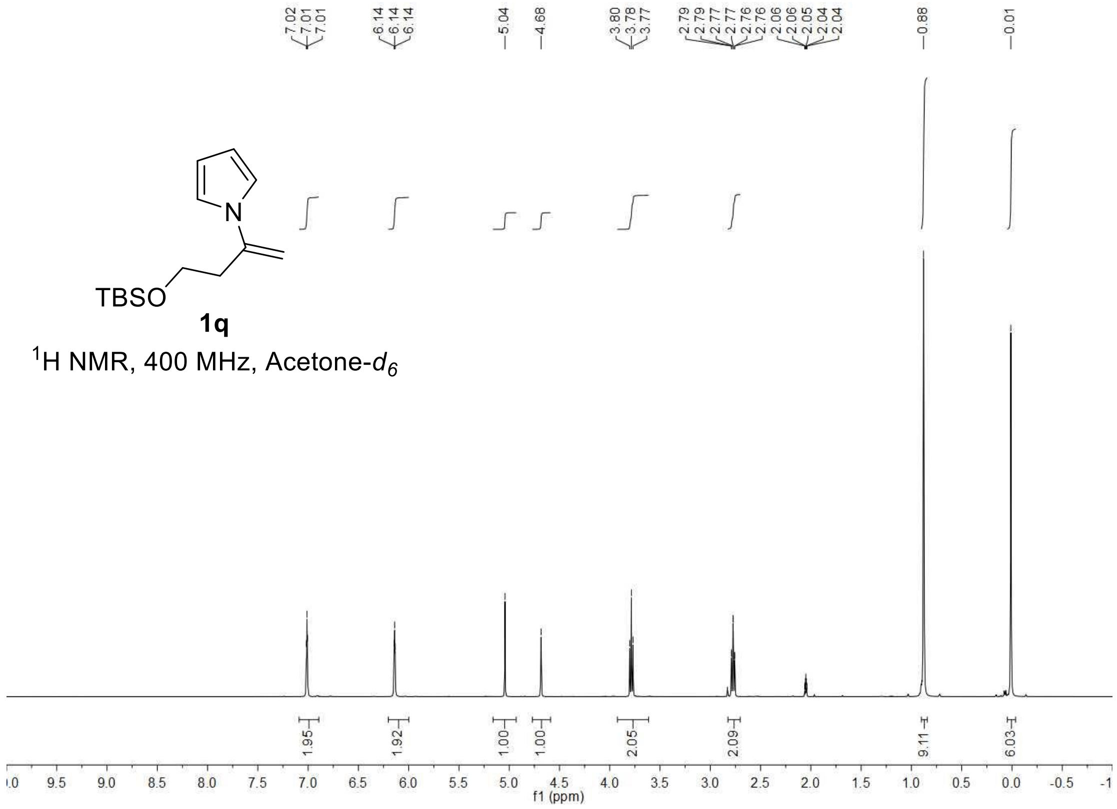

Supplementary Figure 48. ^{1}H NMR spectrum of compound **1q**

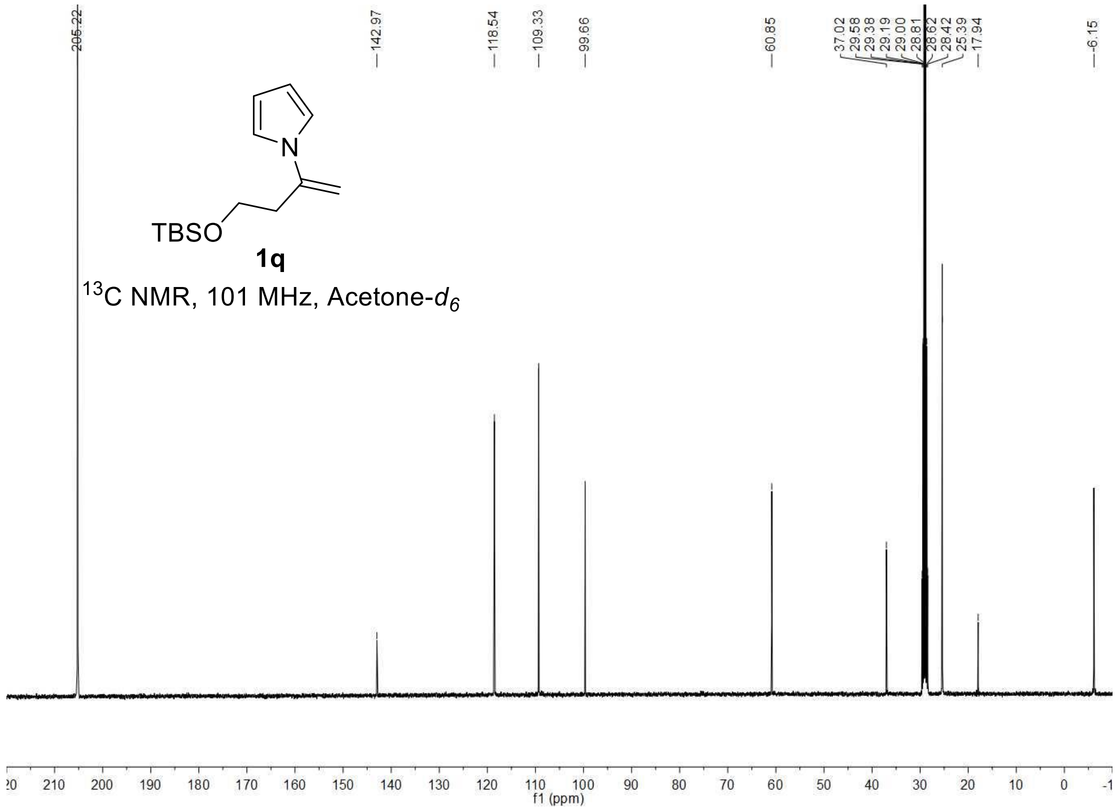

Supplementary Figure 49. ^{13}C NMR spectrum of compound **1q**

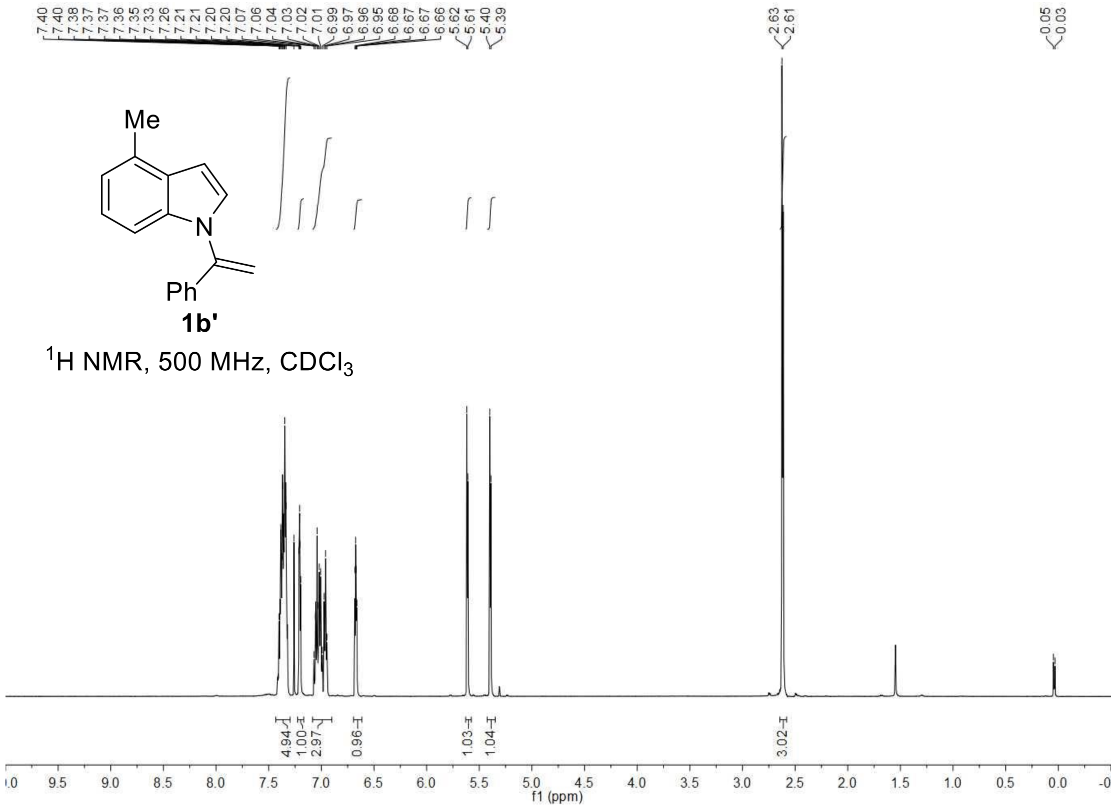

Supplementary Figure 50. ^{1}H NMR spectrum of compound **1b'**

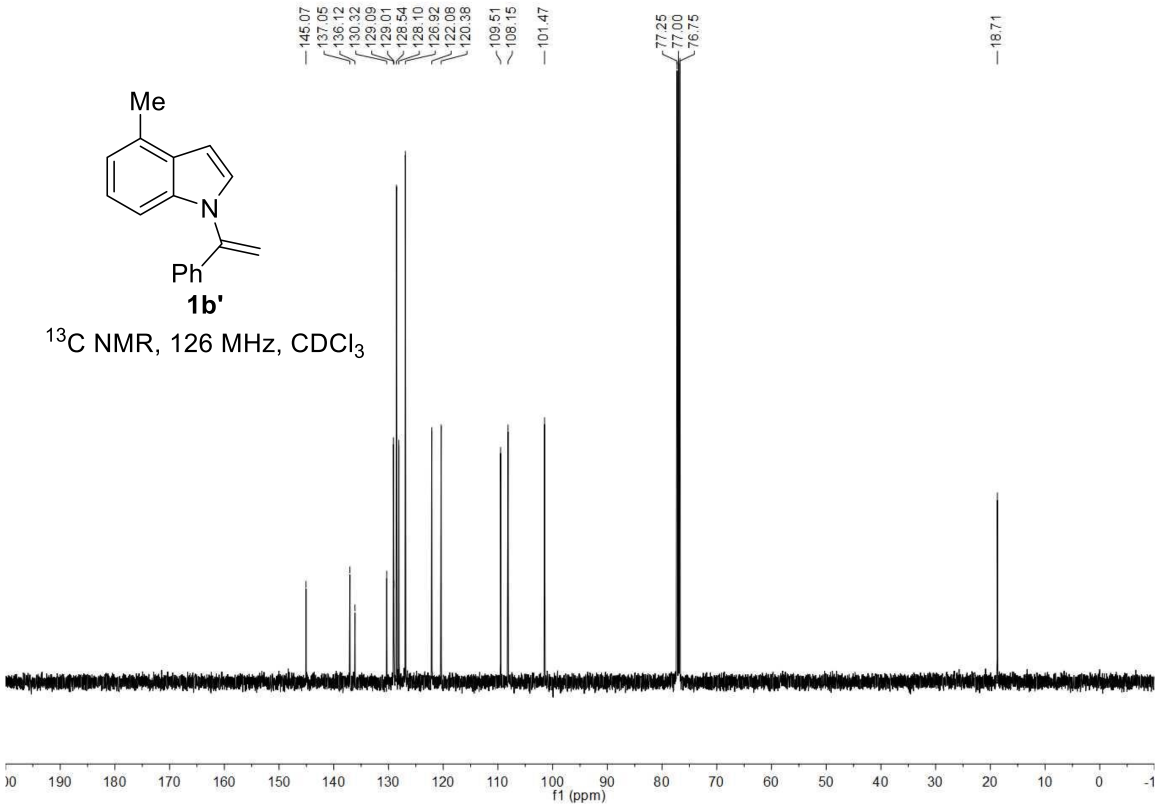

Supplementary Figure 51. ^{13}C NMR spectrum of compound **1b'**

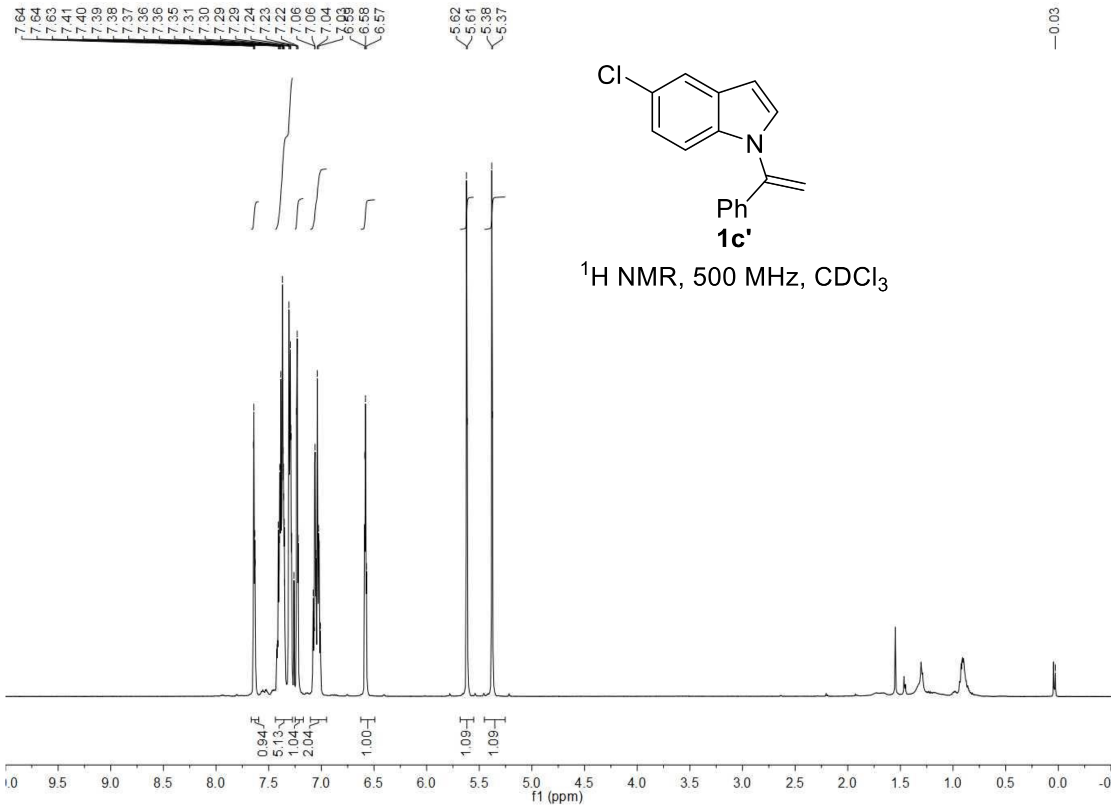

Supplementary Figure 52. ^{1}H NMR spectrum of compound **1c'**

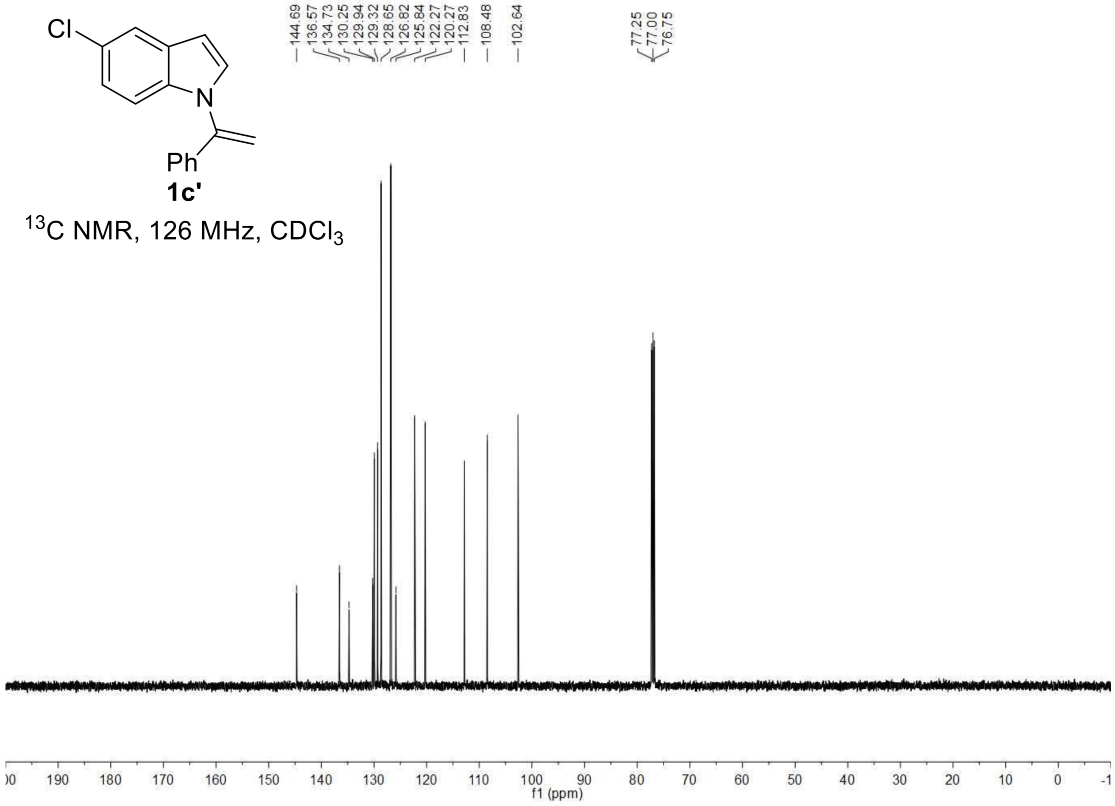

Supplementary Figure 53. ^{13}C NMR spectrum of compound **1c'**

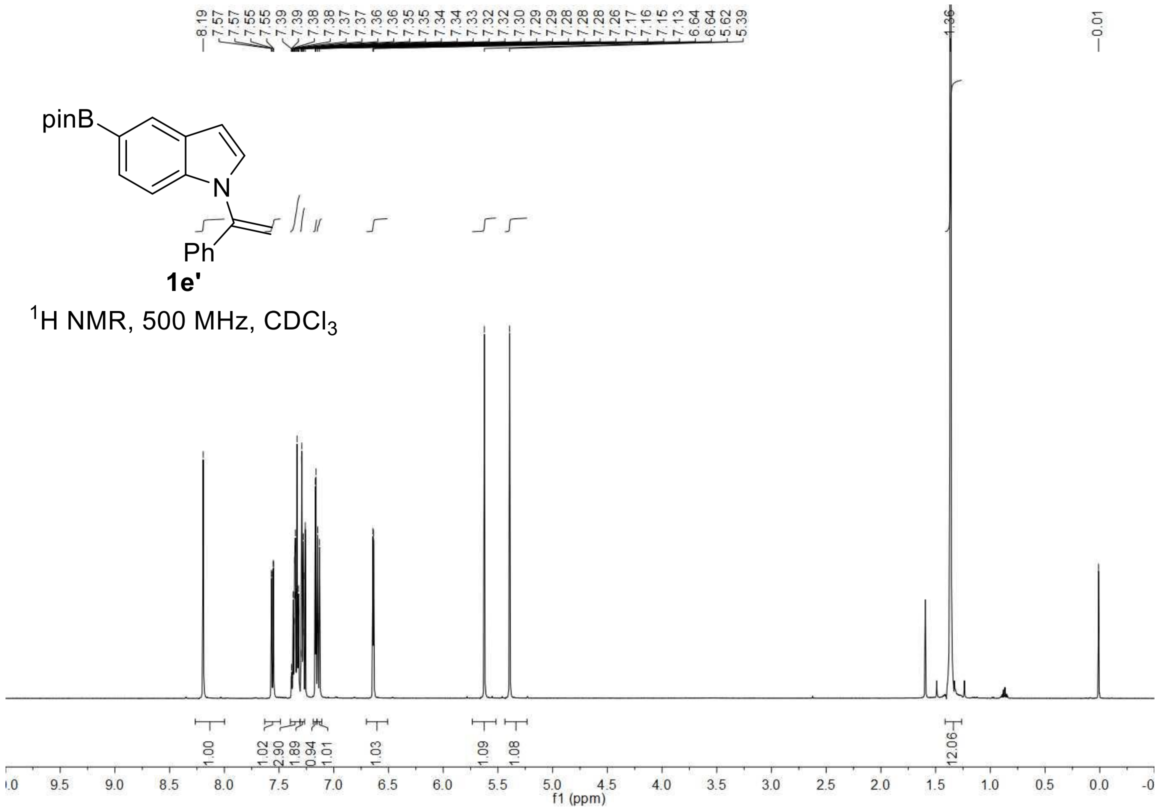

Supplementary Figure 54. ^{1}H NMR spectrum of compound **1e'**

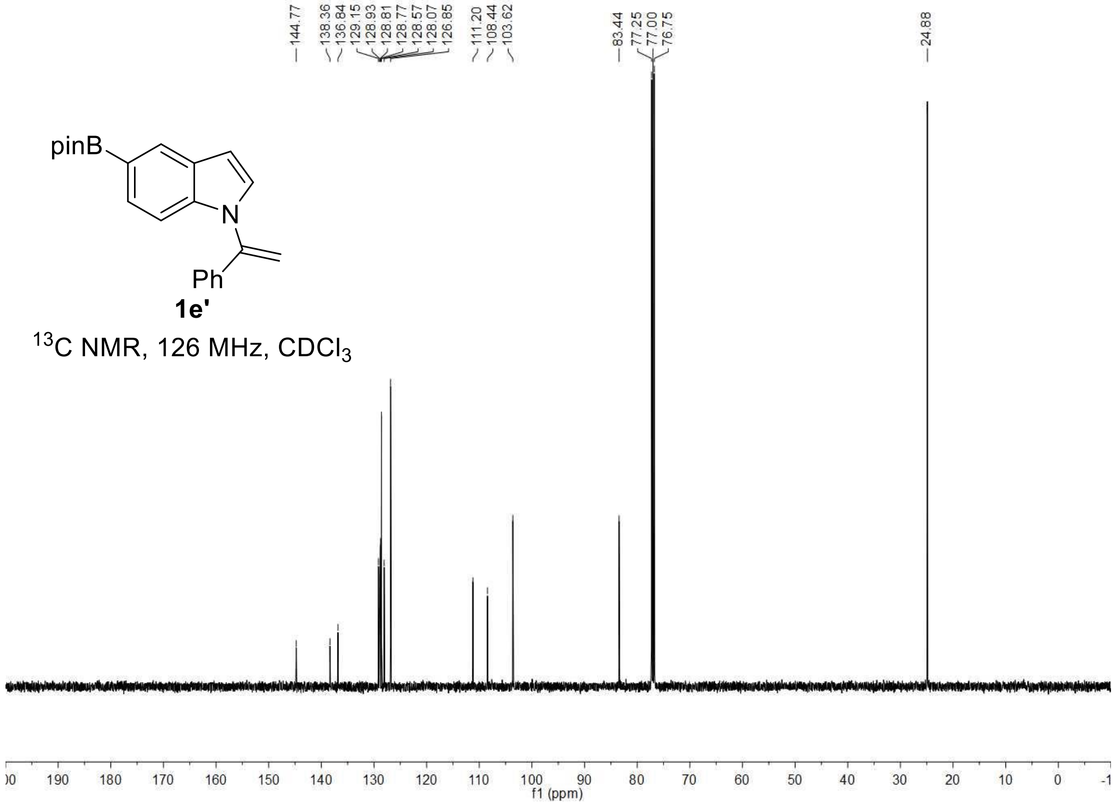

Supplementary Figure 55. ^{13}C NMR spectrum of compound **1e'**

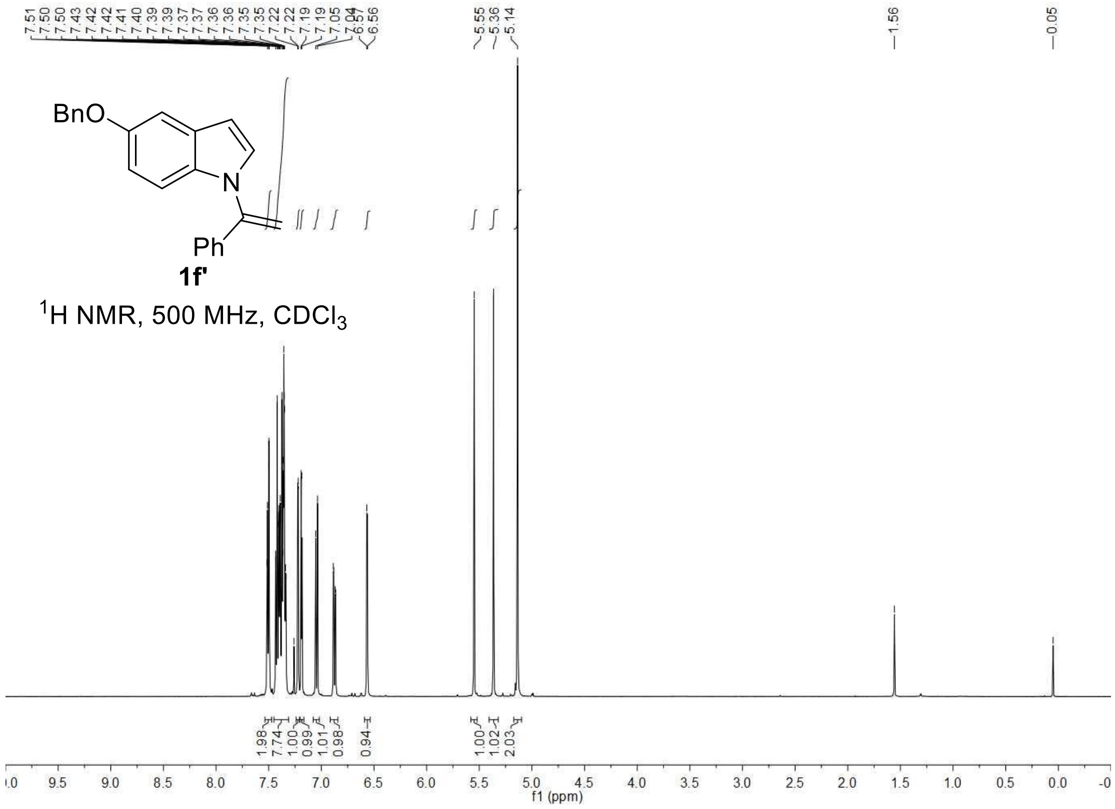

Supplementary Figure 56. ^{1}H NMR spectrum of compound **1f'**

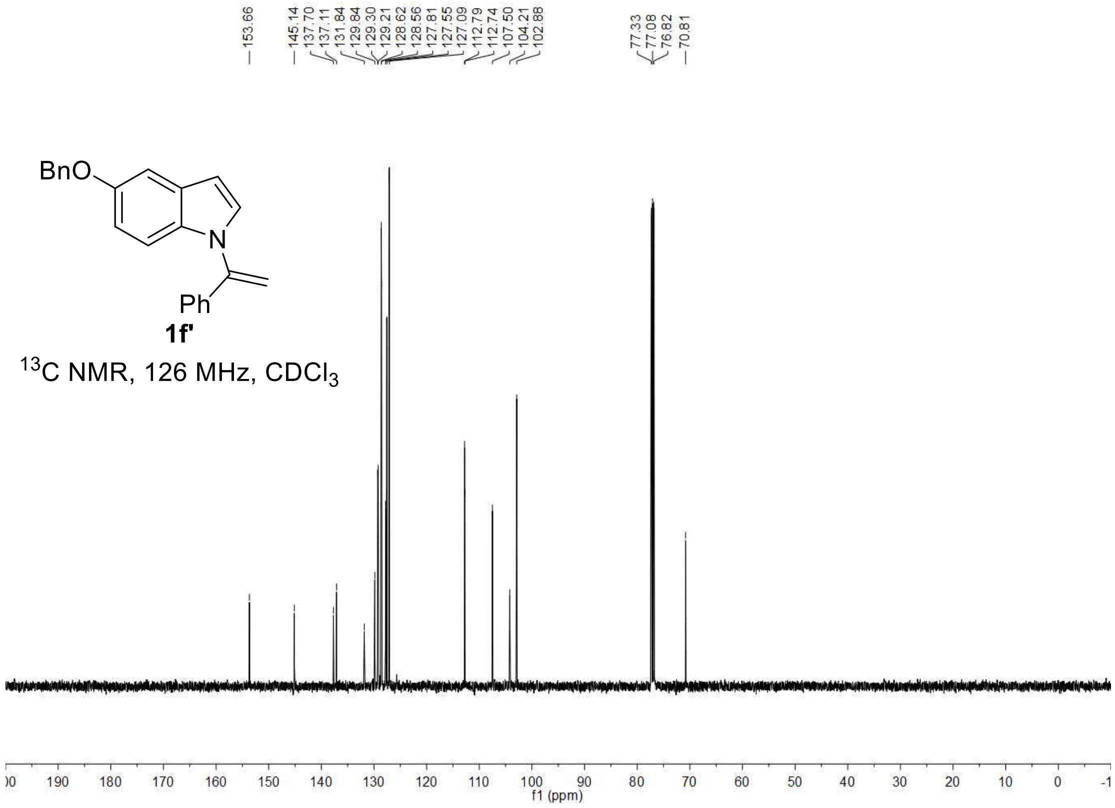

Supplementary Figure 57. ^{13}C NMR spectrum of compound **1f'**

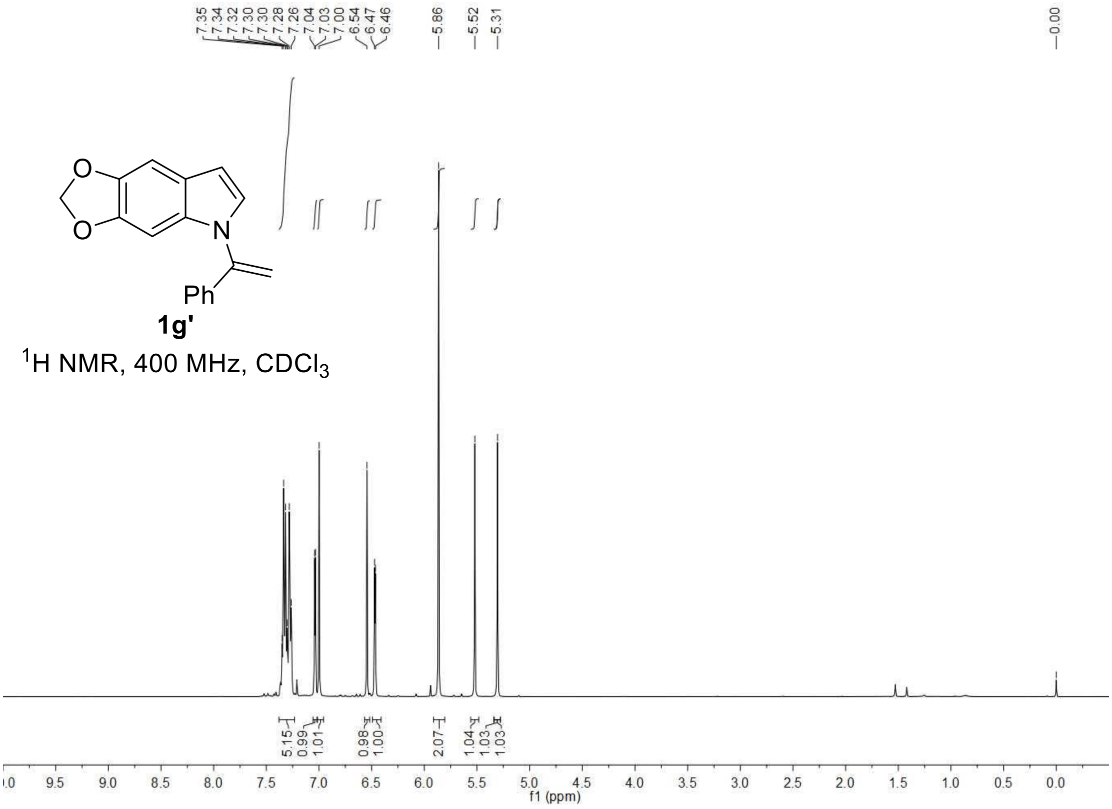

Supplementary Figure 58. ^{1}H NMR spectrum of compound **1g'**

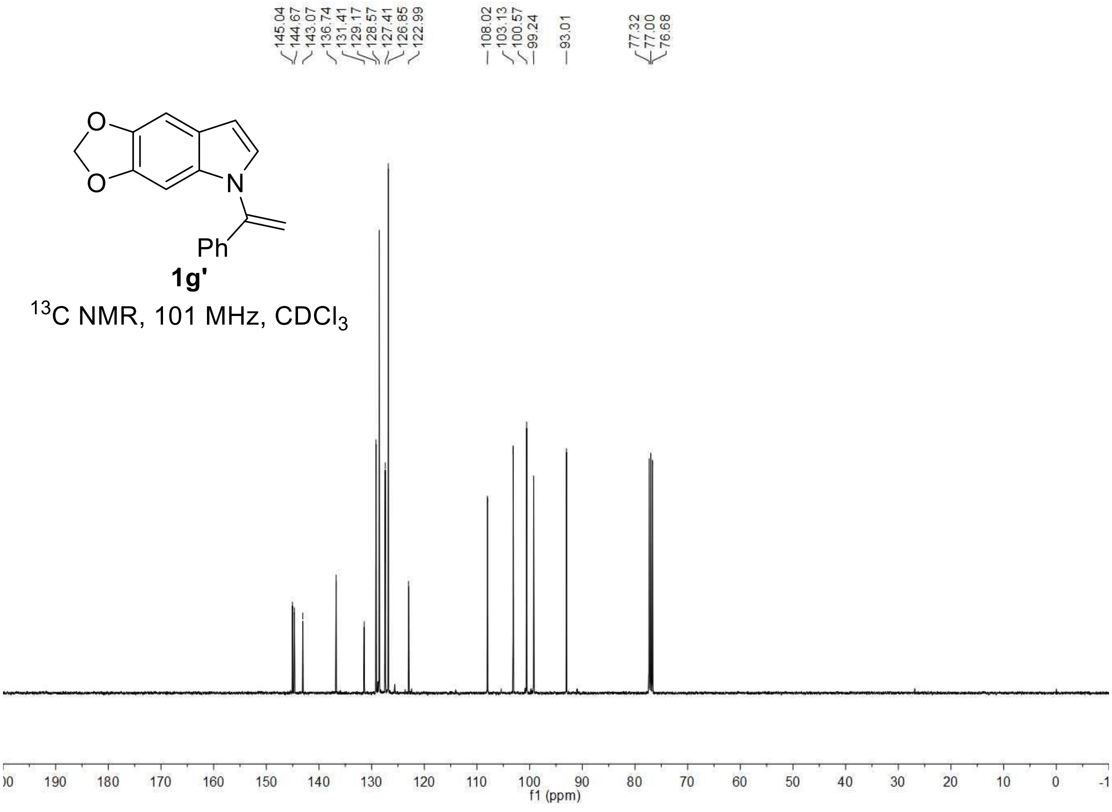

Supplementary Figure 59. ^{13}C NMR spectrum of compound **1g'**

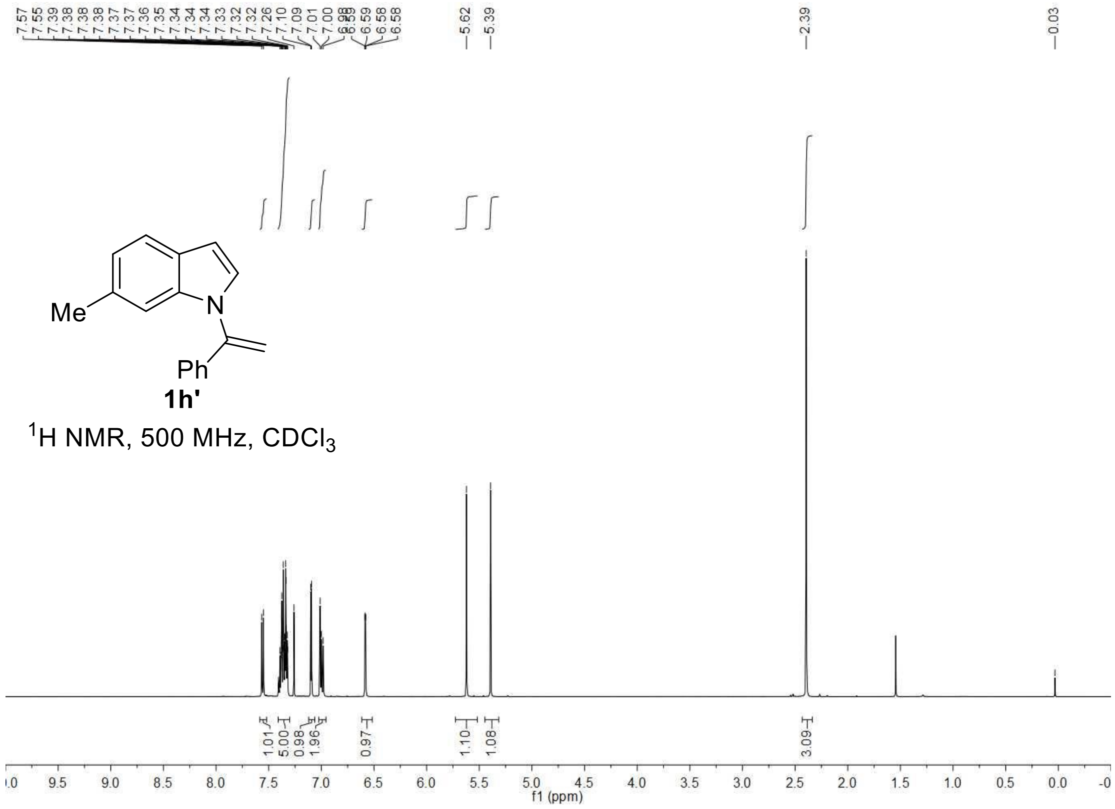

Supplementary Figure 60. ^{1}H NMR spectrum of compound **1h'**

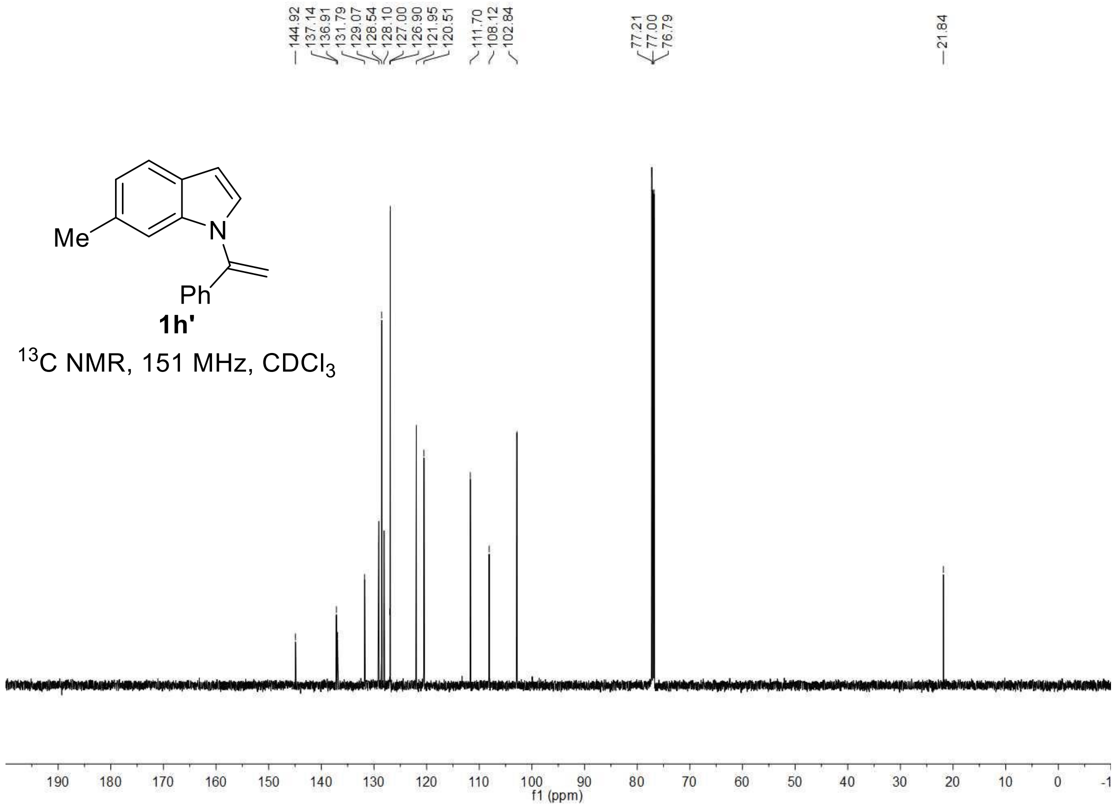

Supplementary Figure 61. ^{13}C NMR spectrum of compound **1h'**

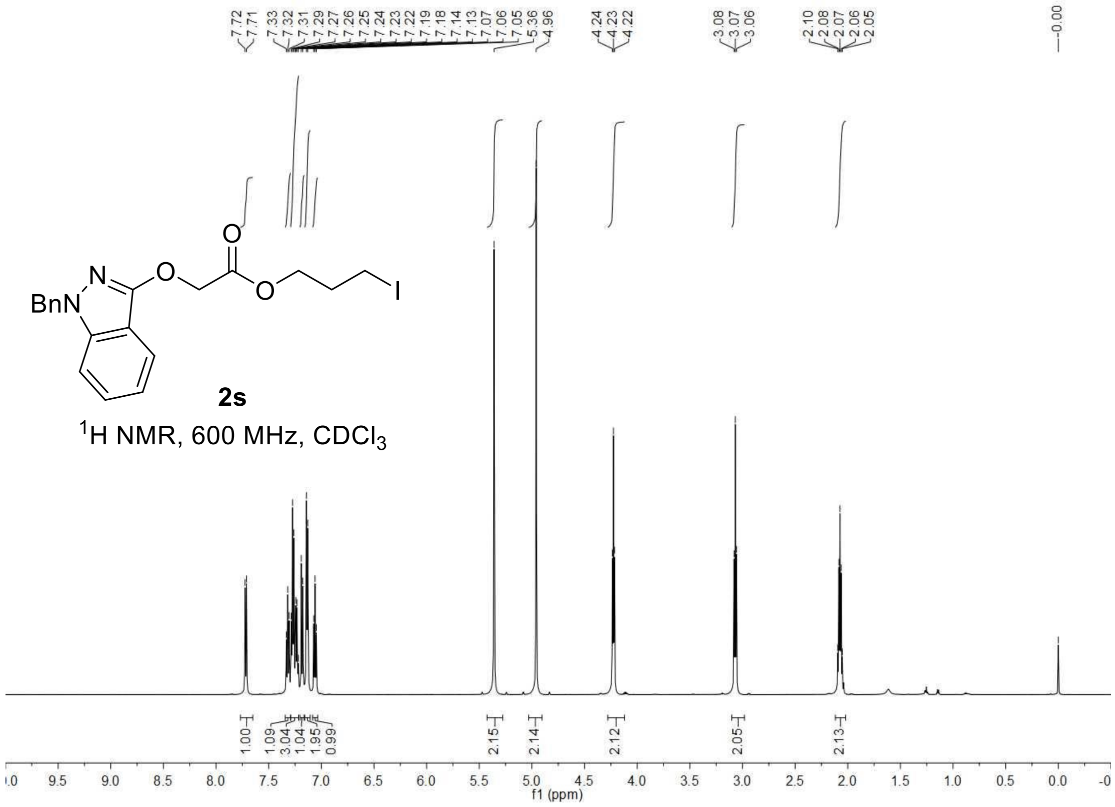

Supplementary Figure 62. ^{1}H NMR spectrum of compound **2s**

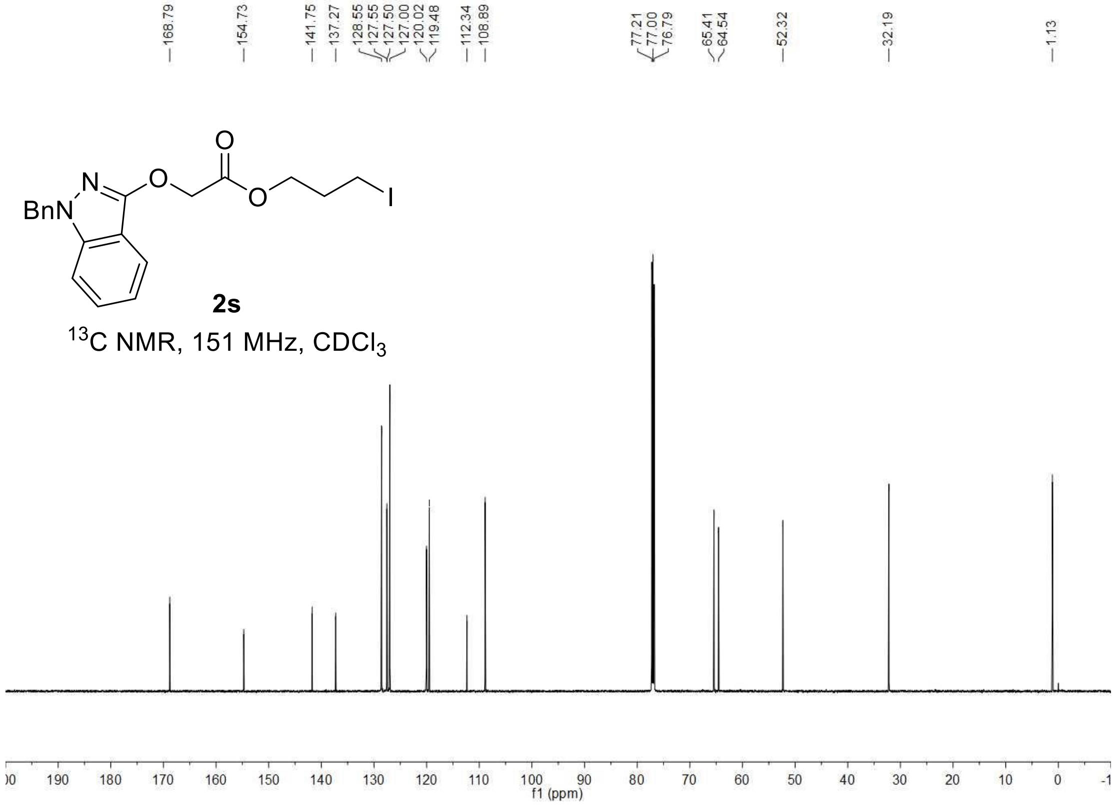

Supplementary Figure 63. ^{13}C NMR spectrum of compound **2s**

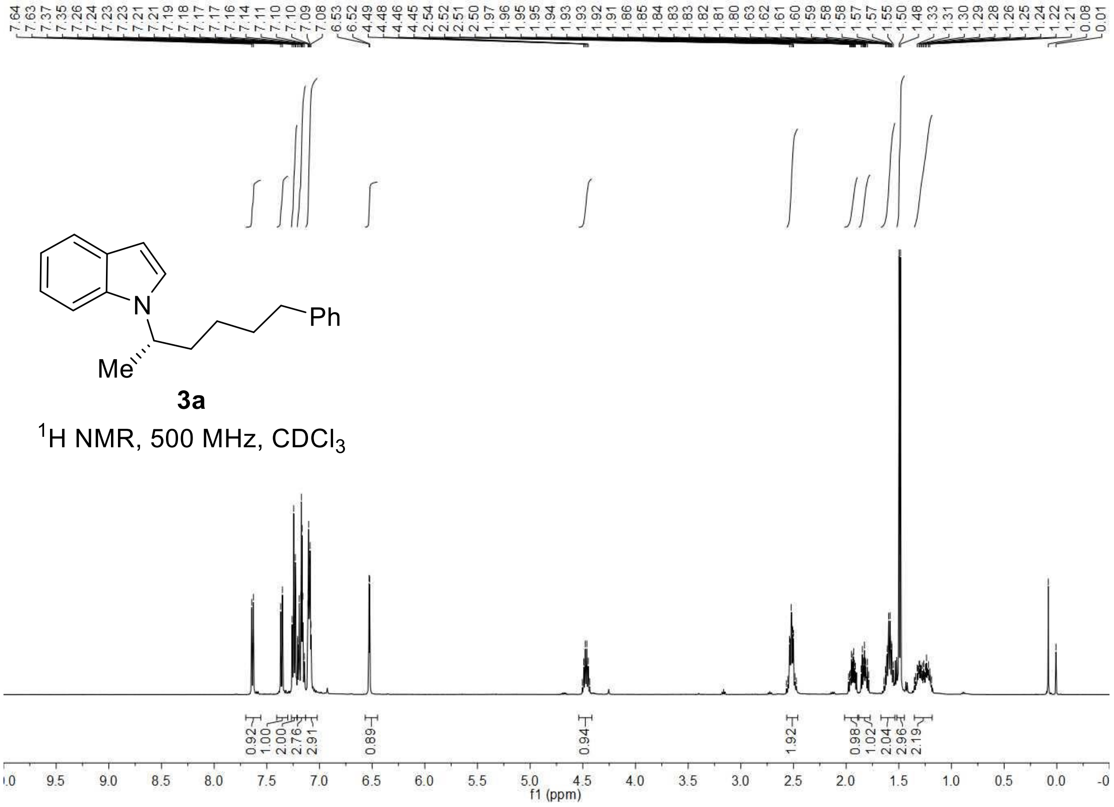

Supplementary Figure 64. ^{1}H NMR spectrum of compound **3a**

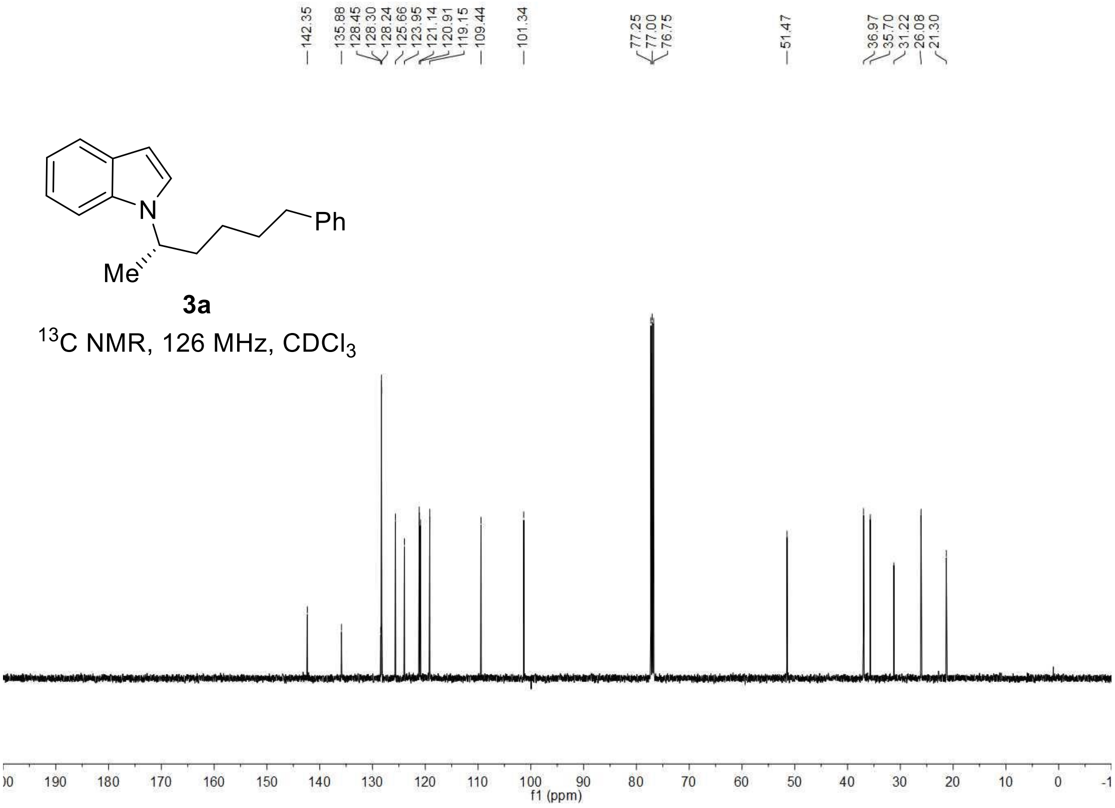

Supplementary Figure 65. ^{13}C NMR spectrum of compound **3a**

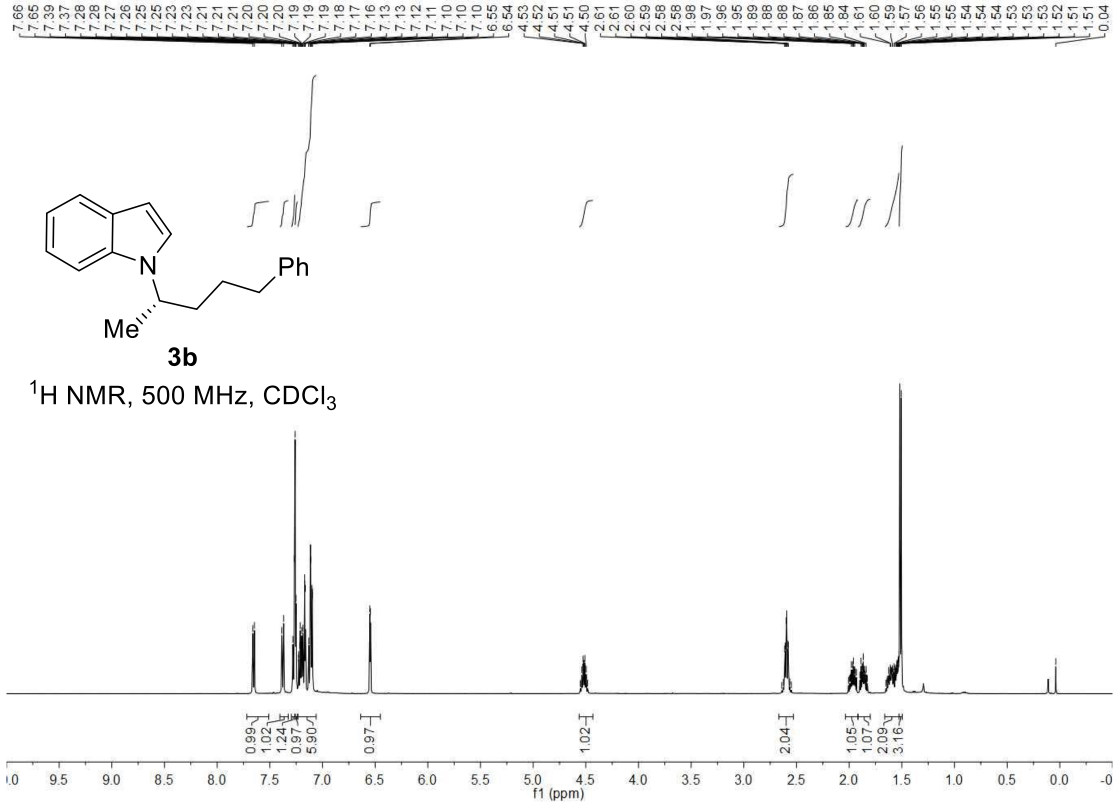

Supplementary Figure 66. ^{1}H NMR spectrum of compound **3b**

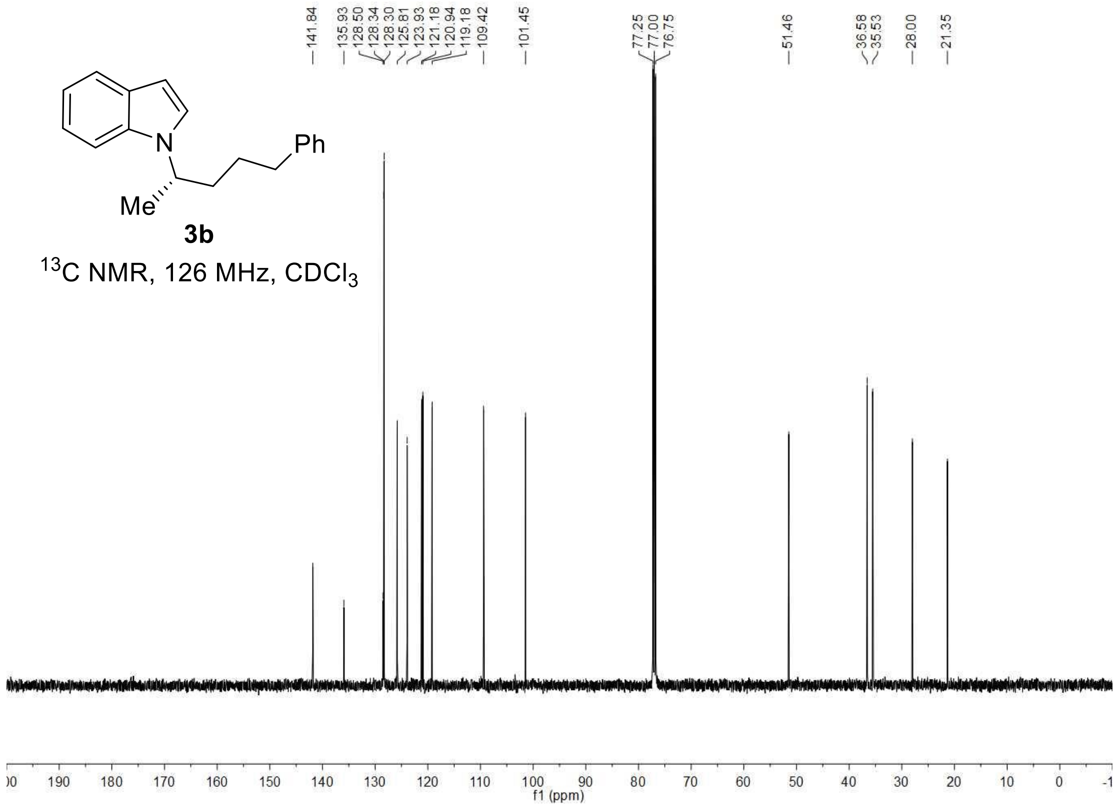

Supplementary Figure 67. ^{13}C NMR spectrum of compound **3b**

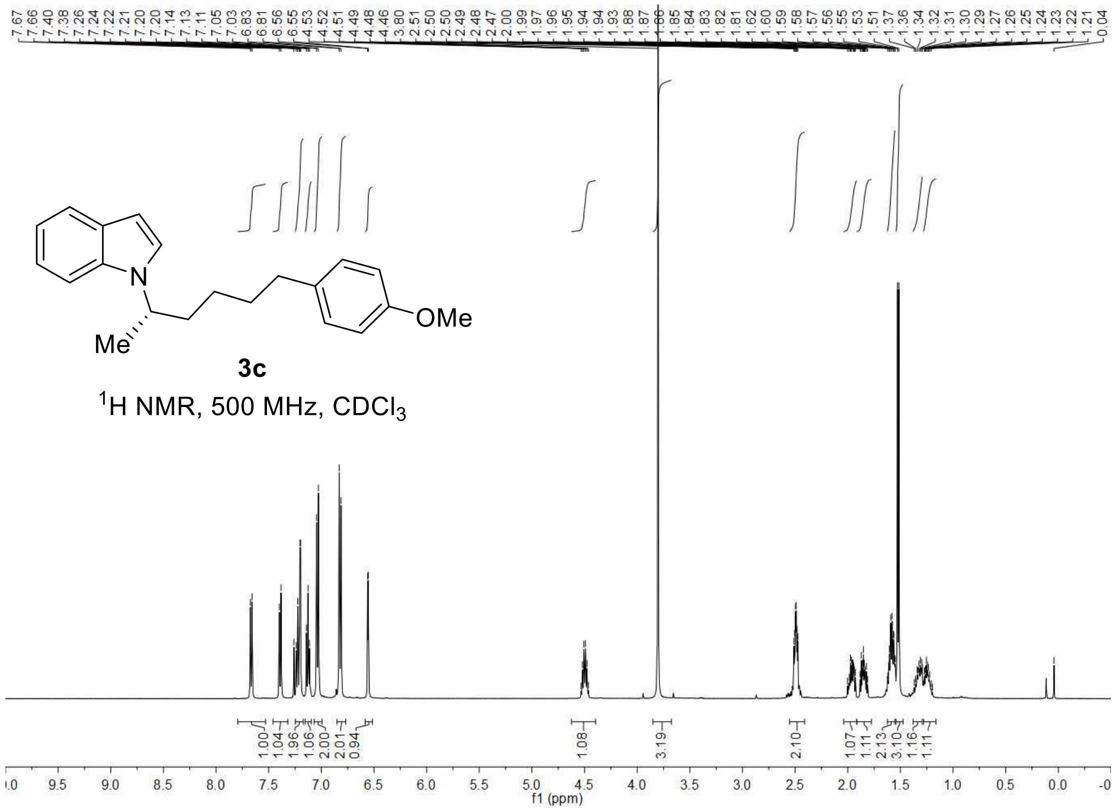

Supplementary Figure 68. ^{1}H NMR spectrum of compound **3c**

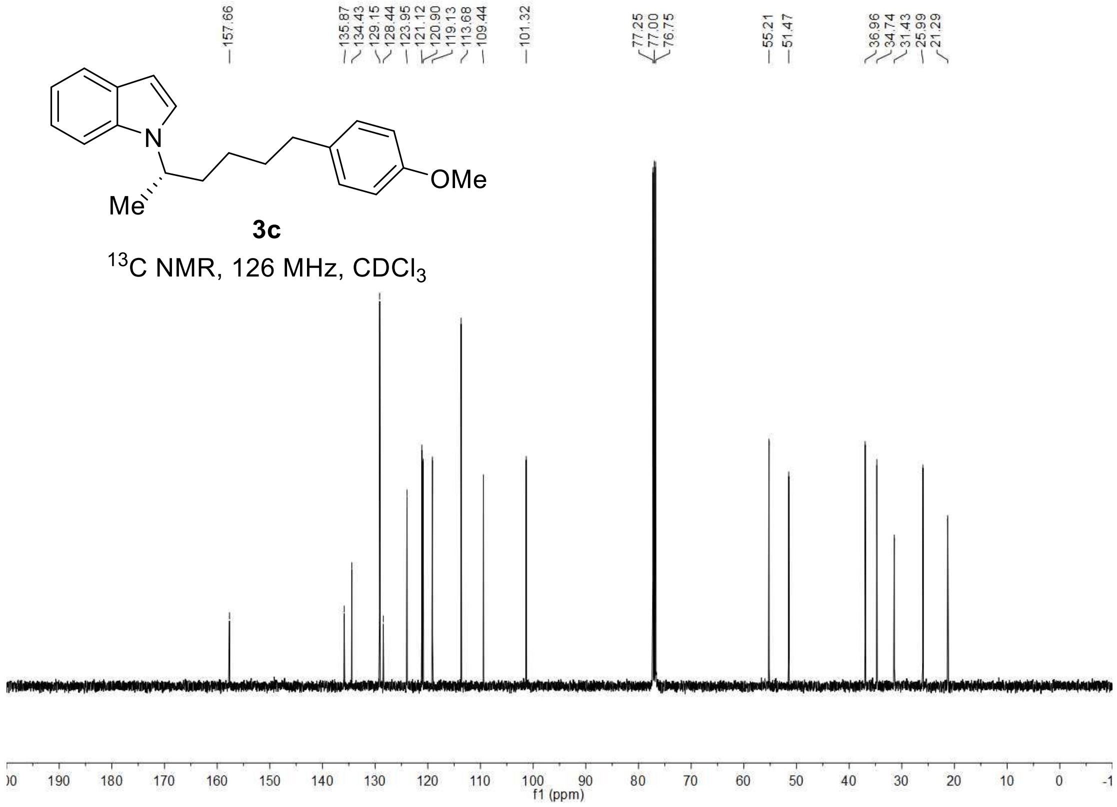

Supplementary Figure 69. ^{13}C NMR spectrum of compound **3c**

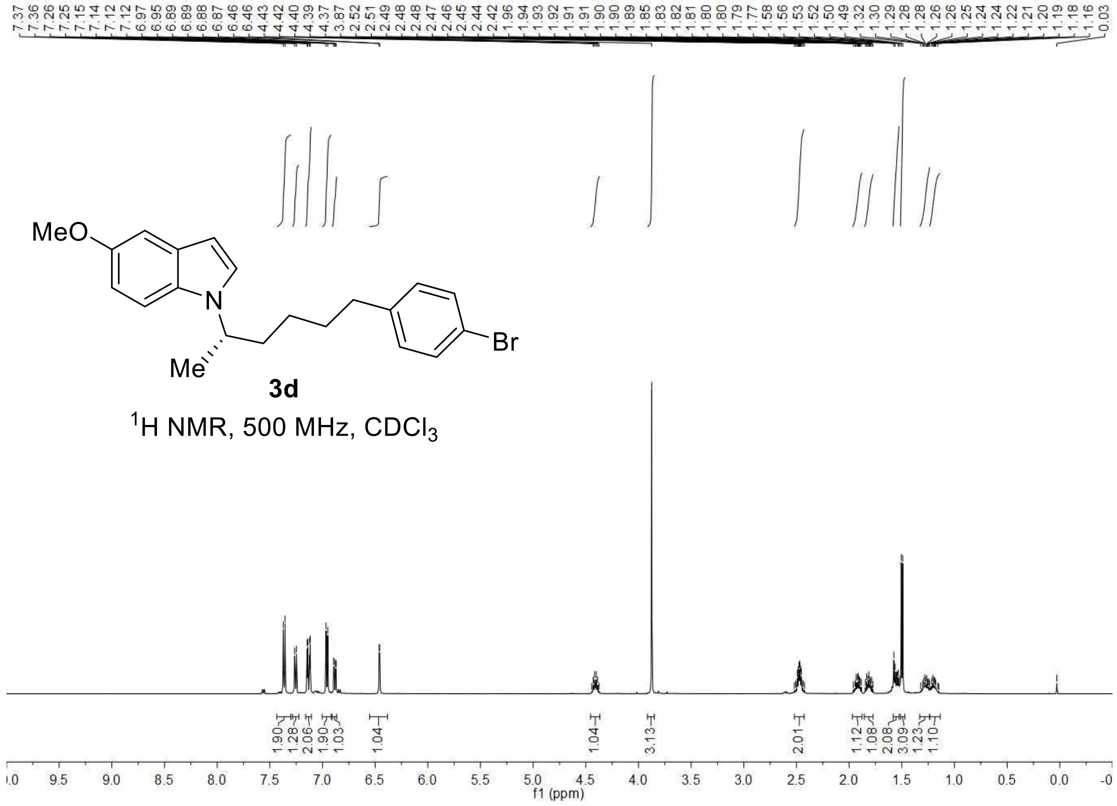

Supplementary Figure 70. ^{1}H NMR spectrum of compound **3d**

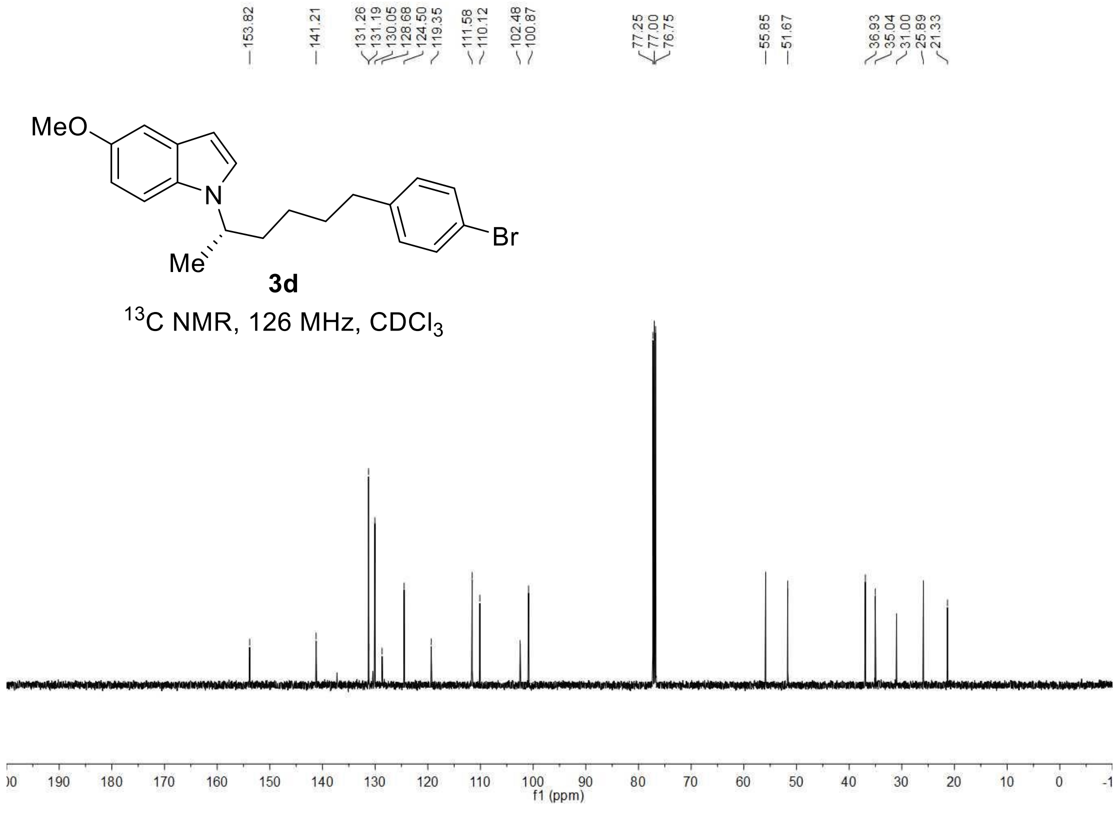

Supplementary Figure 71. ^{13}C NMR spectrum of compound **3d**

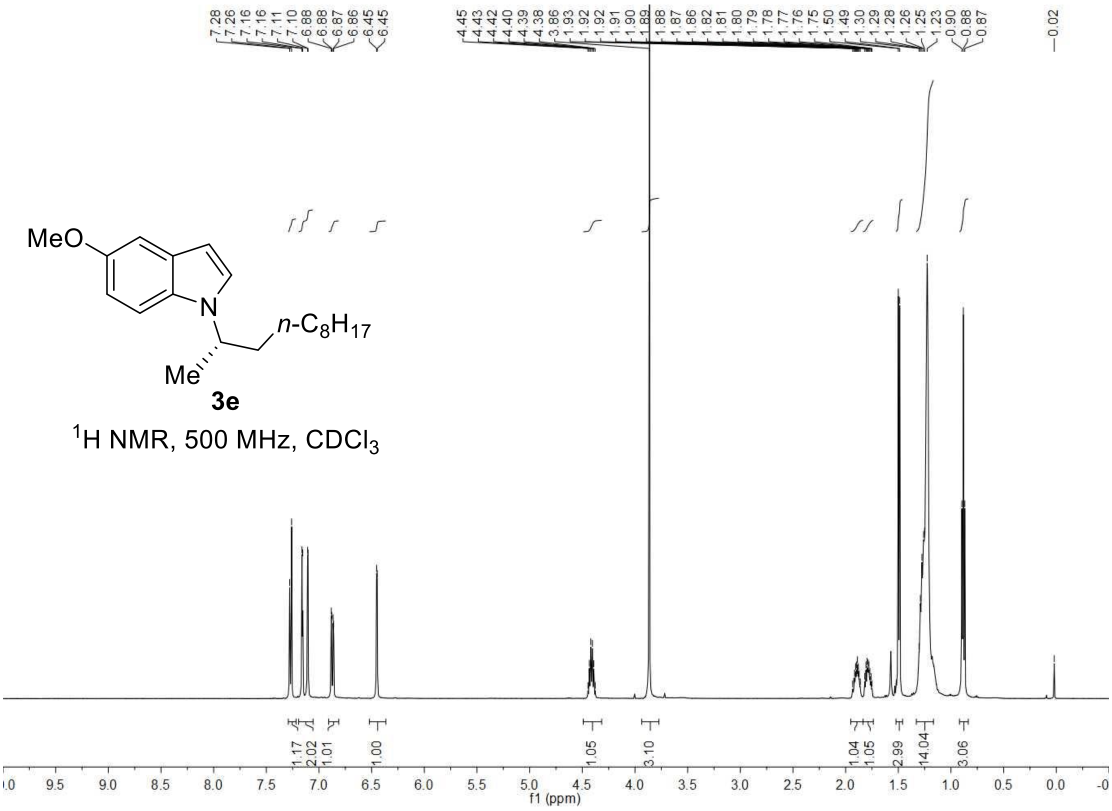

Supplementary Figure 72. ^{1}H NMR spectrum of compound **3e**

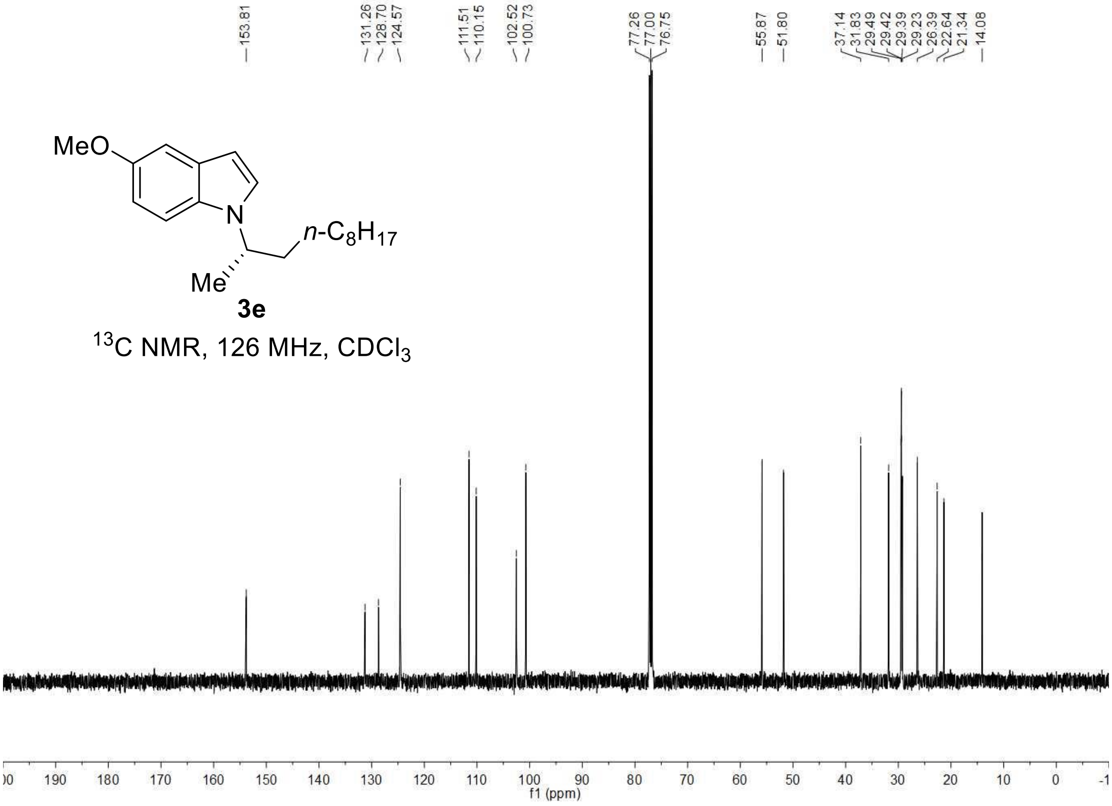

Supplementary Figure 73. ^{13}C NMR spectrum of compound **3e**

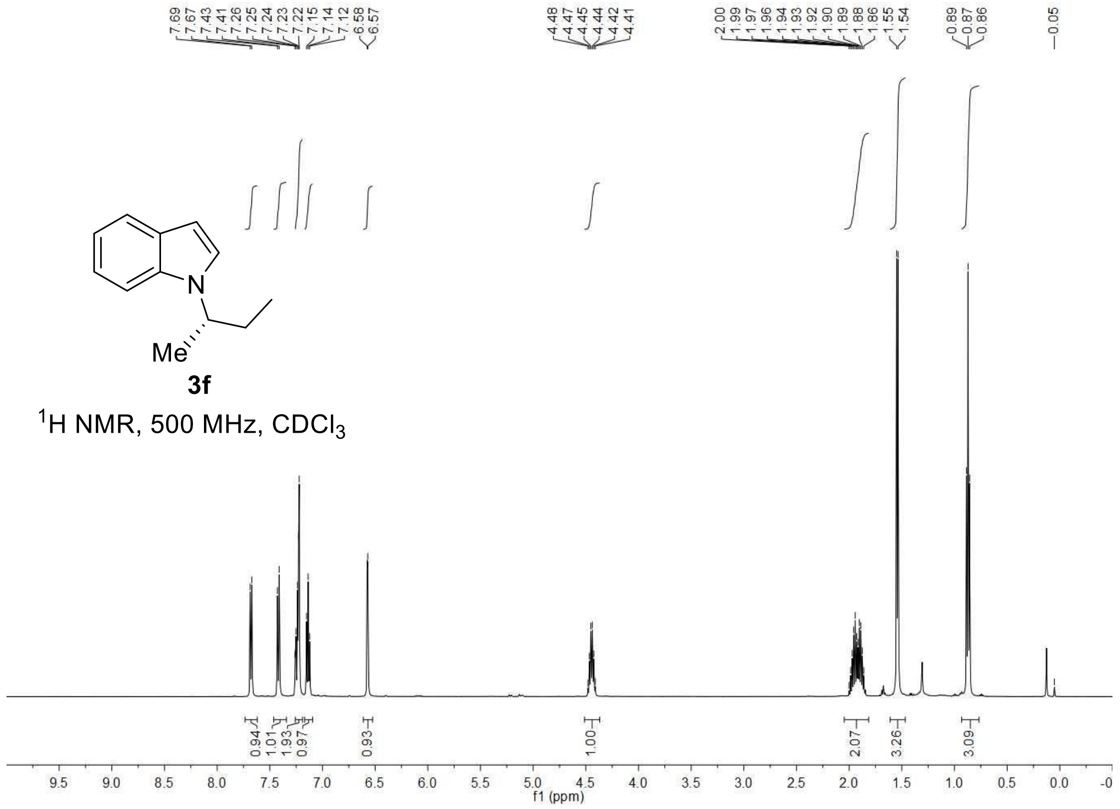

Supplementary Figure 74. ^{1}H NMR spectrum of compound **3f**

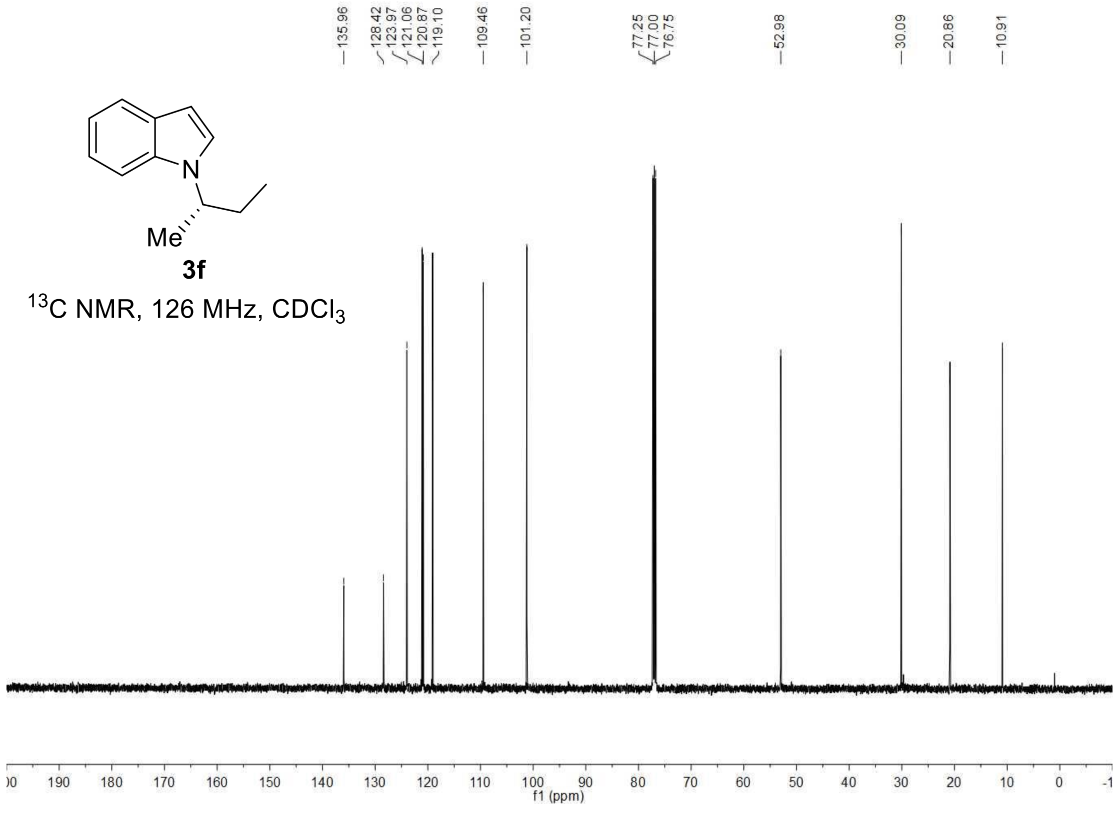

Supplementary Figure 75. ^{13}C NMR spectrum of compound **3f**

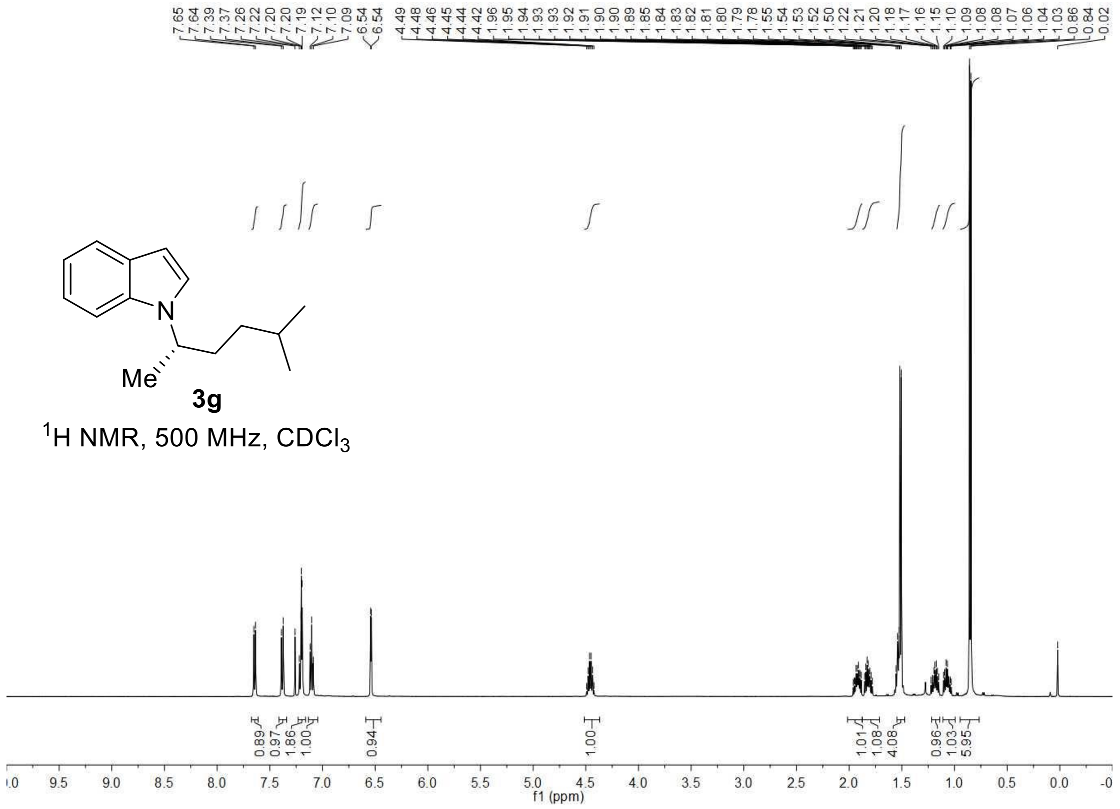

Supplementary Figure 76. ^{1}H NMR spectrum of compound **3g**

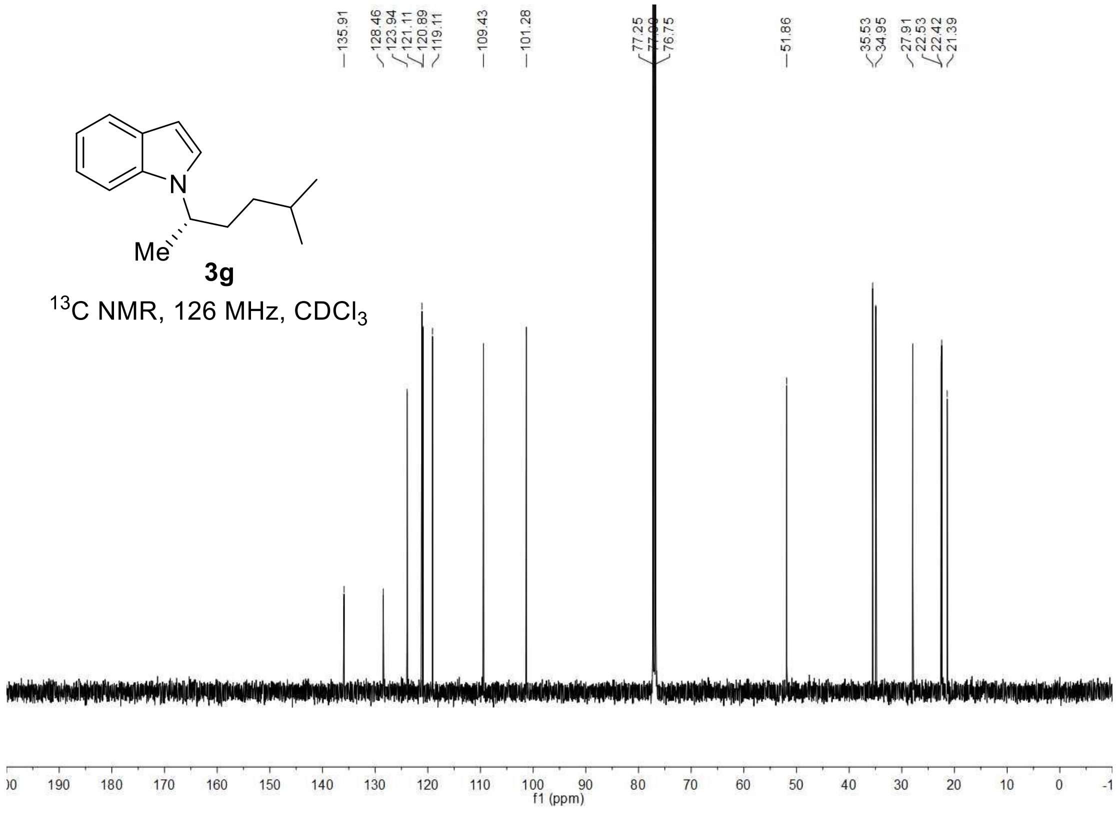

Supplementary Figure 77. ^{13}C NMR spectrum of compound **3g**

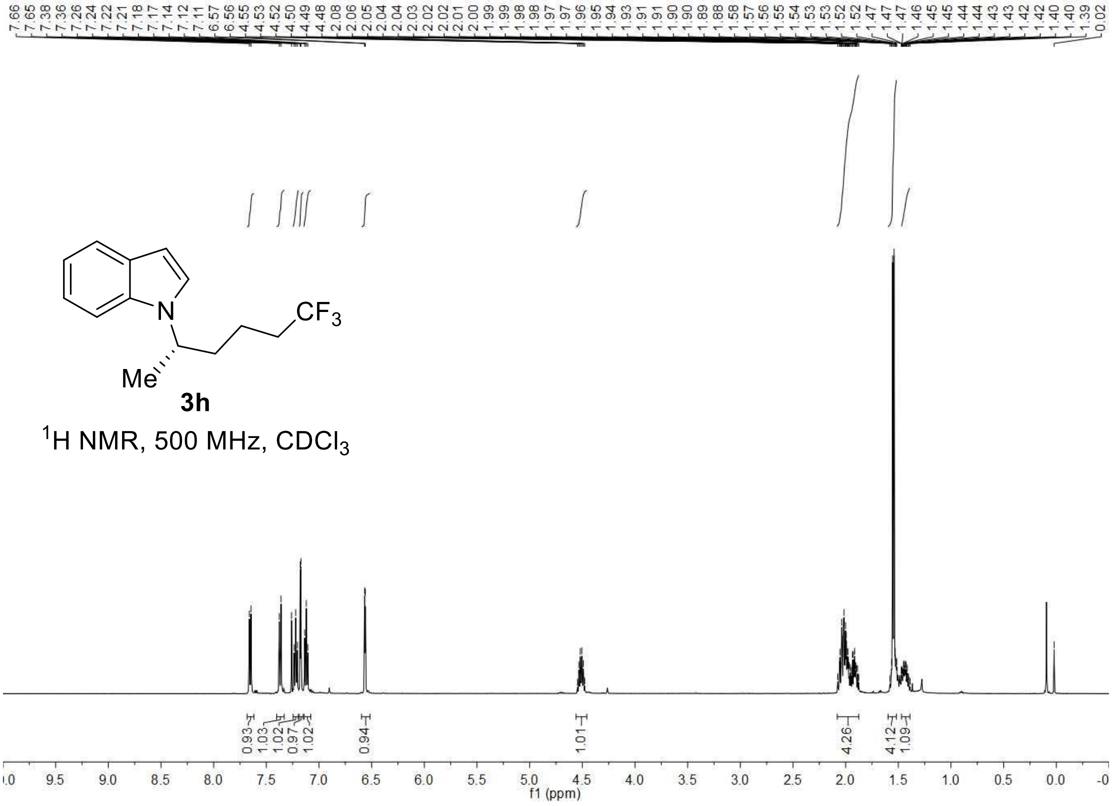

Supplementary Figure 78. ^{1}H NMR spectrum of compound **3h**

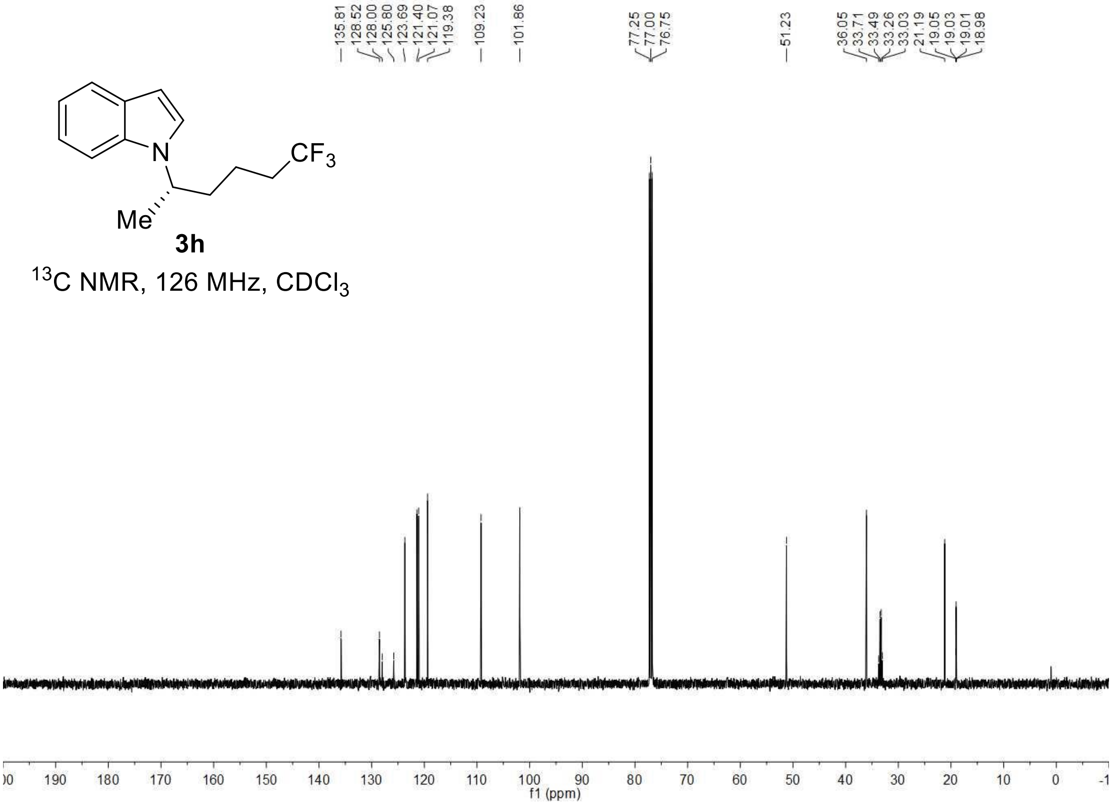

Supplementary Figure 79. ^{13}C NMR spectrum of compound **3h**

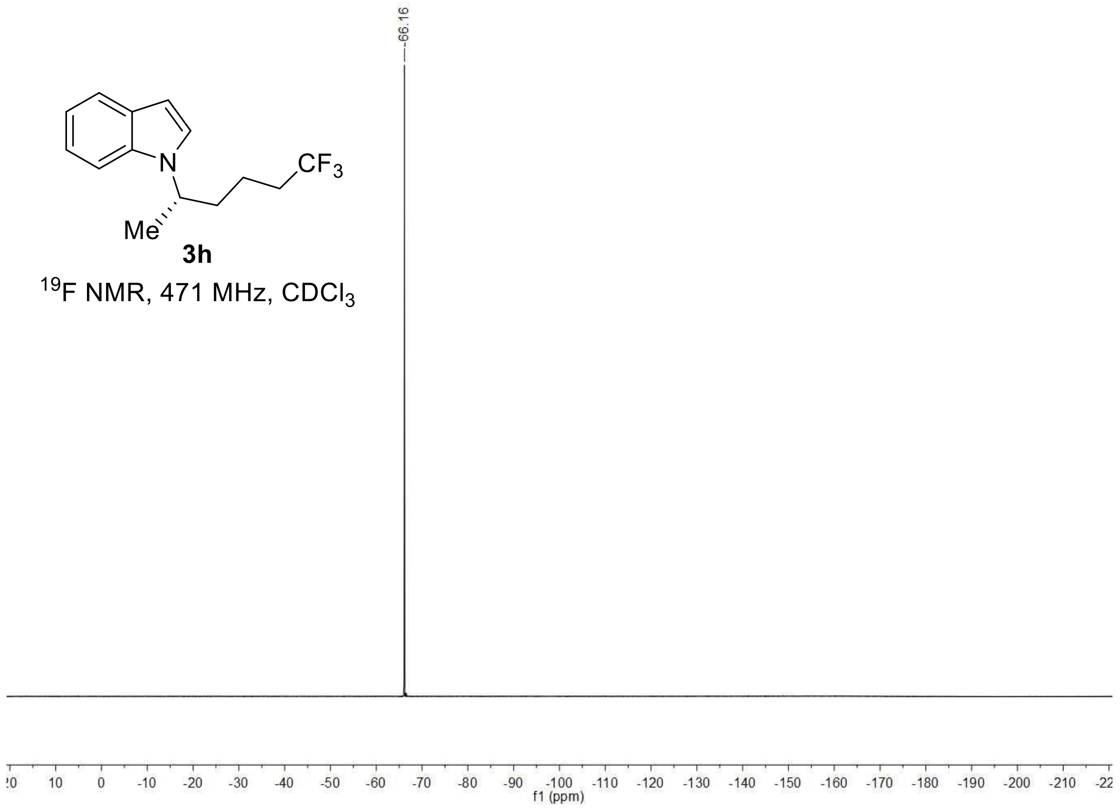

Supplementary Figure 80. ^{19}F NMR spectrum of compound **3h**

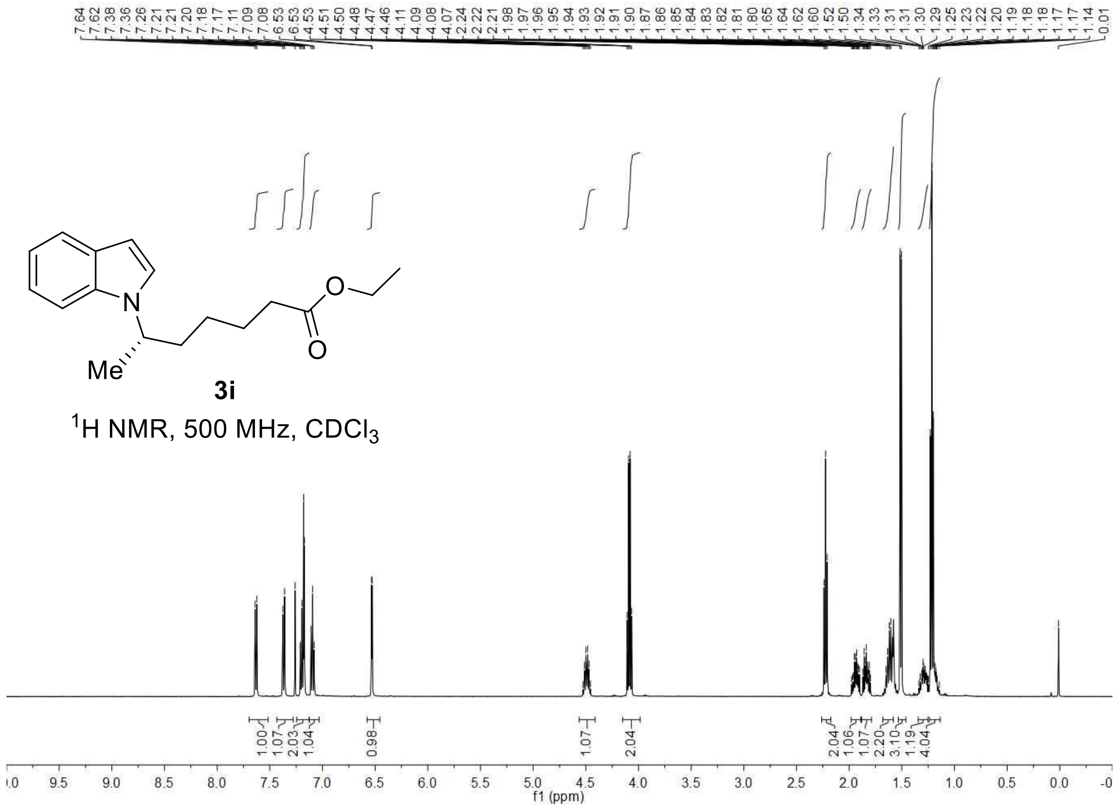

Supplementary Figure 81. ^{1}H NMR spectrum of compound **3i**

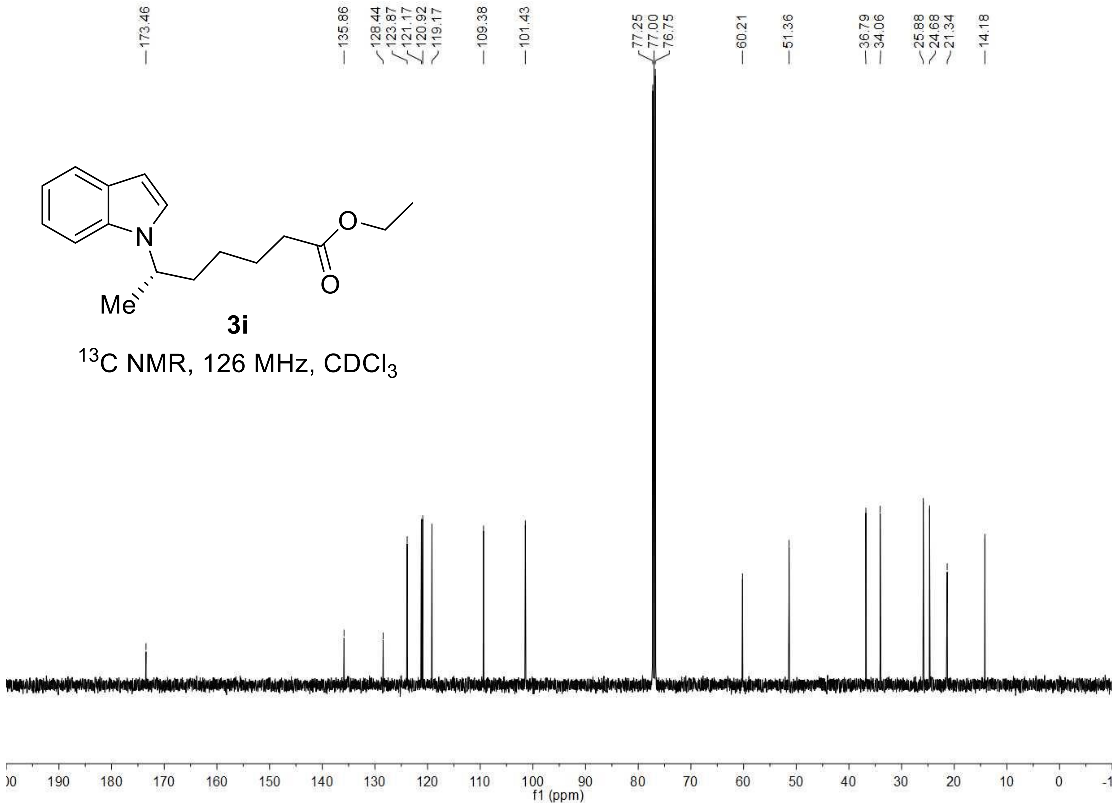

Supplementary Figure 82. ^{13}C NMR spectrum of compound **3i**

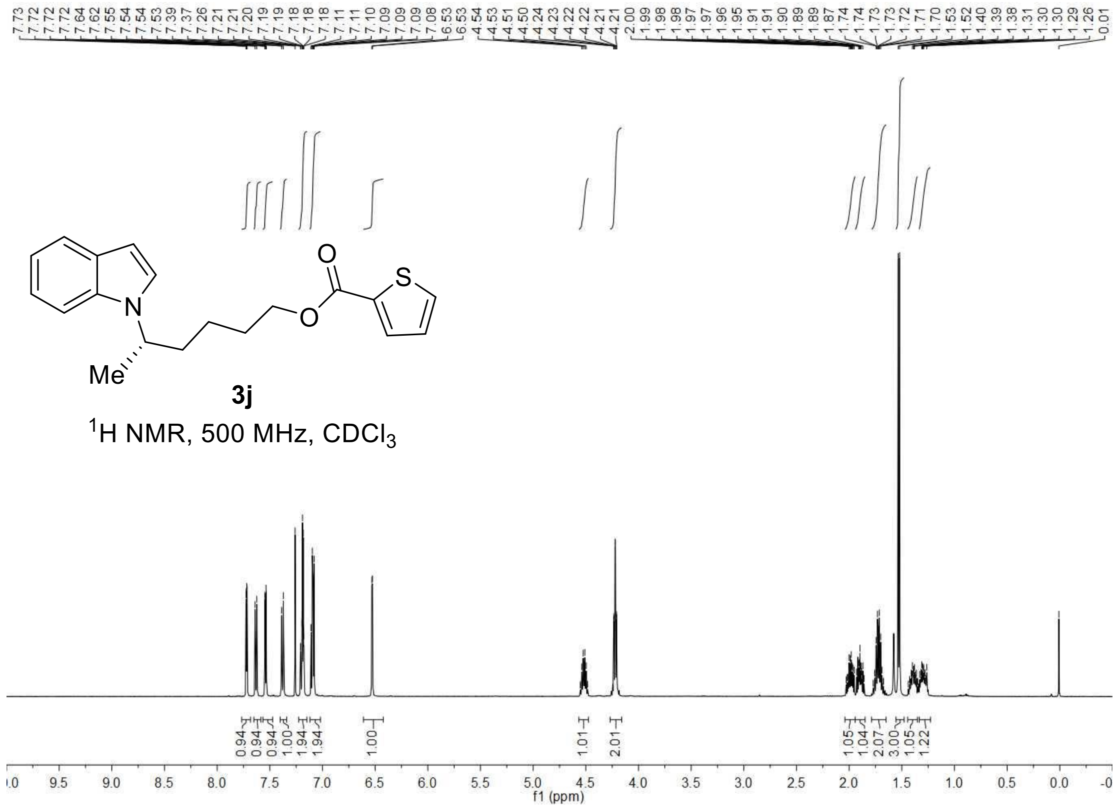

Supplementary Figure 83. ^{1}H NMR spectrum of compound **3j**

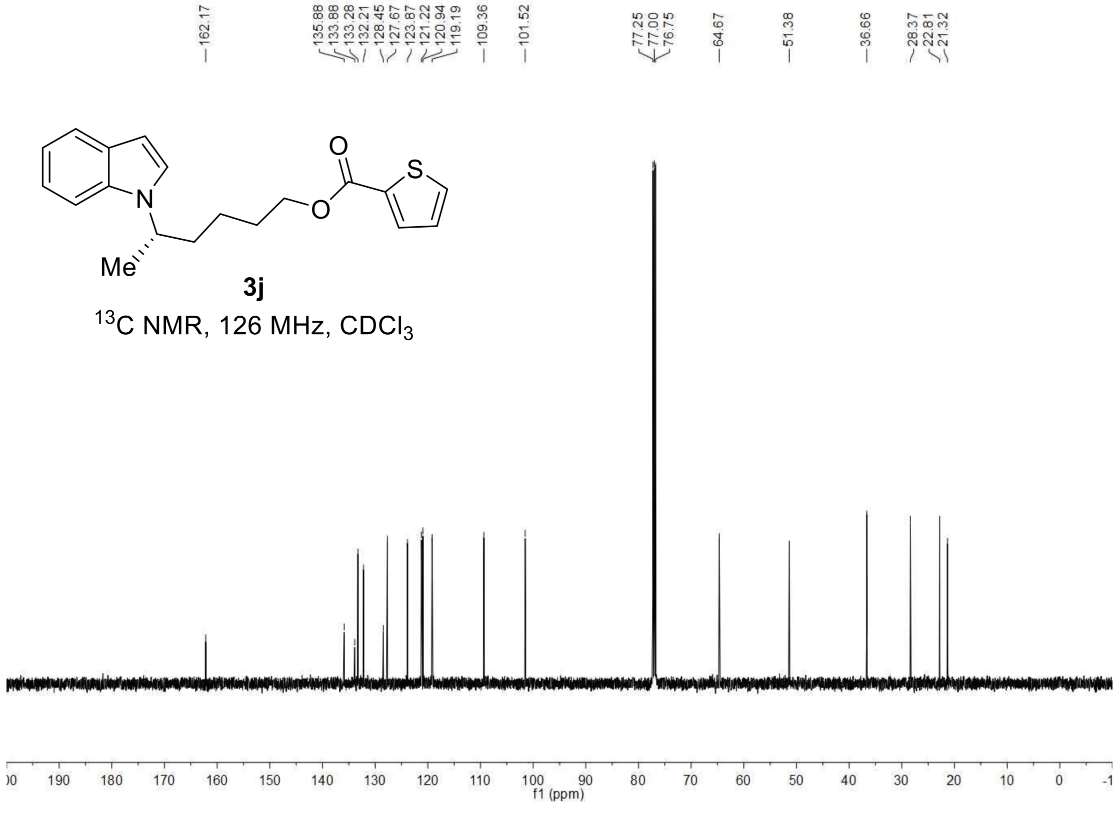

Supplementary Figure 84. ^{13}C NMR spectrum of compound **3j**

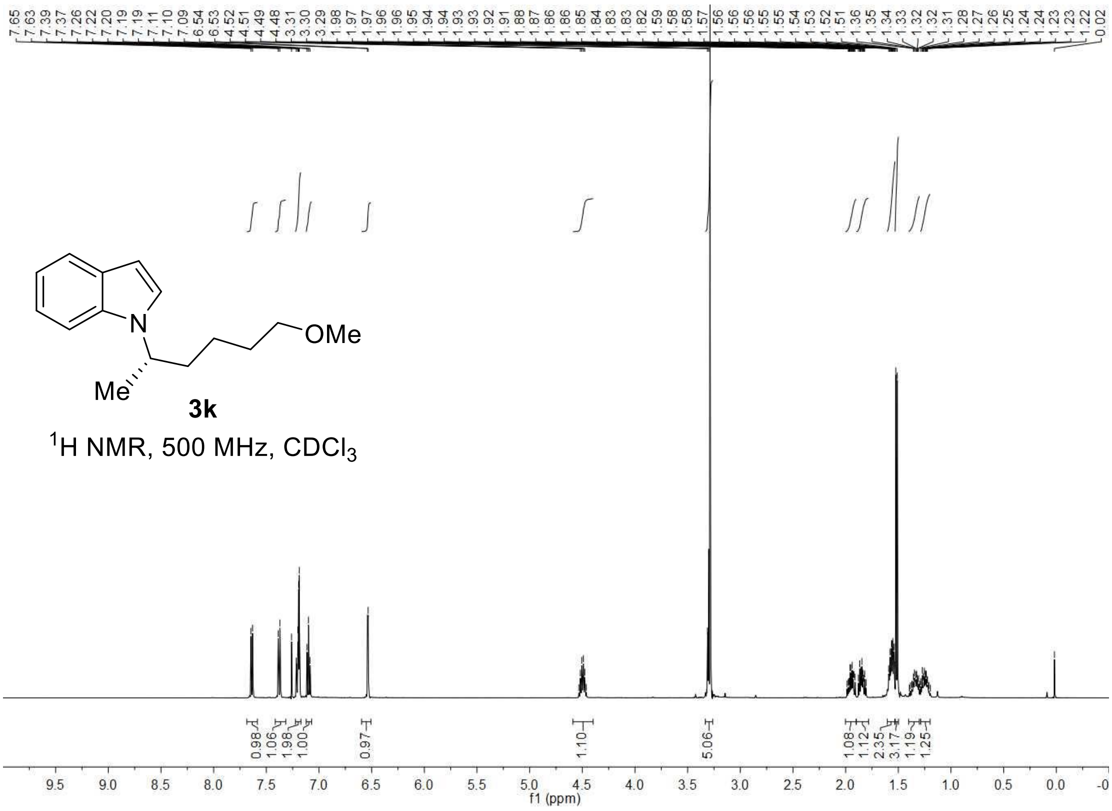

Supplementary Figure 85. ^{1}H NMR spectrum of compound **3k**

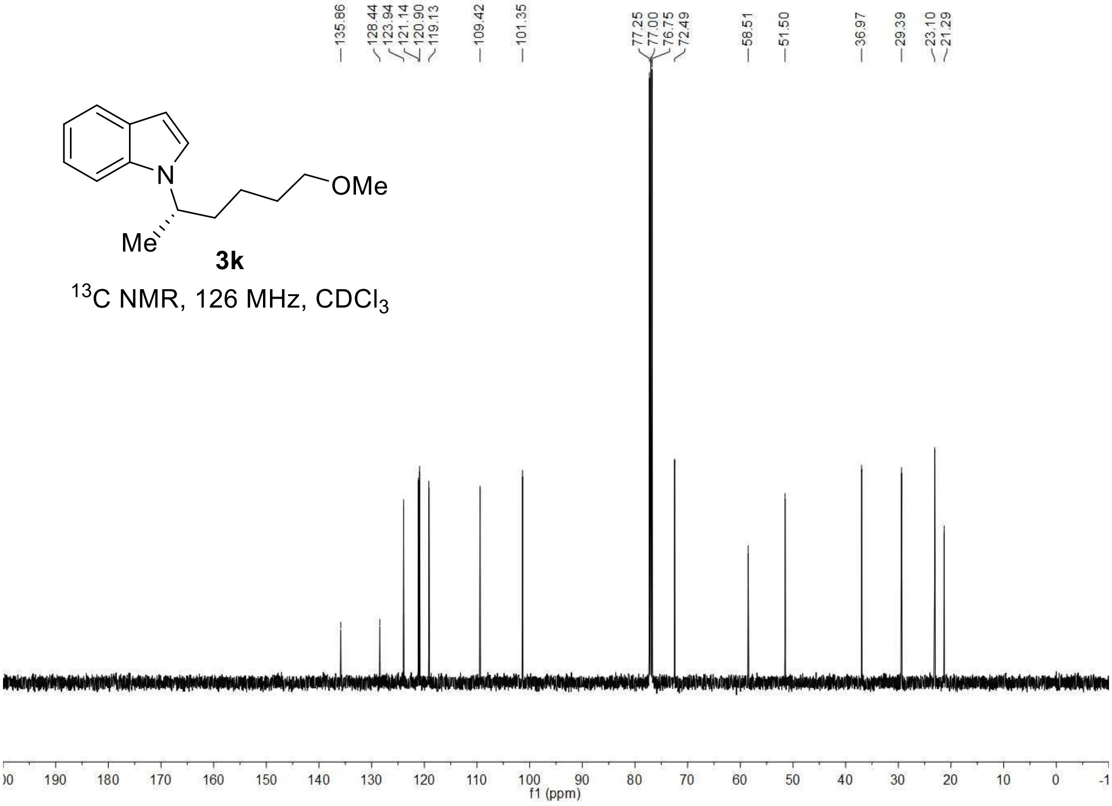

Supplementary Figure 86. ^{13}C NMR spectrum of compound **3k**

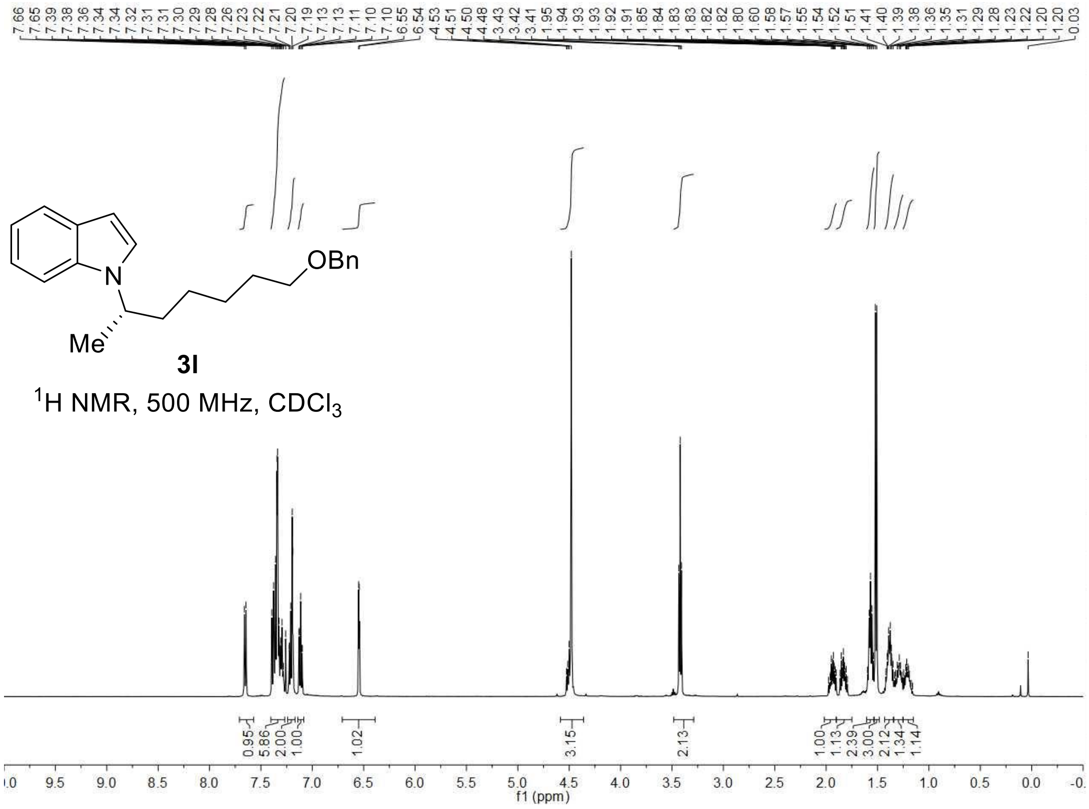

Supplementary Figure 87. ^{1}H NMR spectrum of compound **3l**

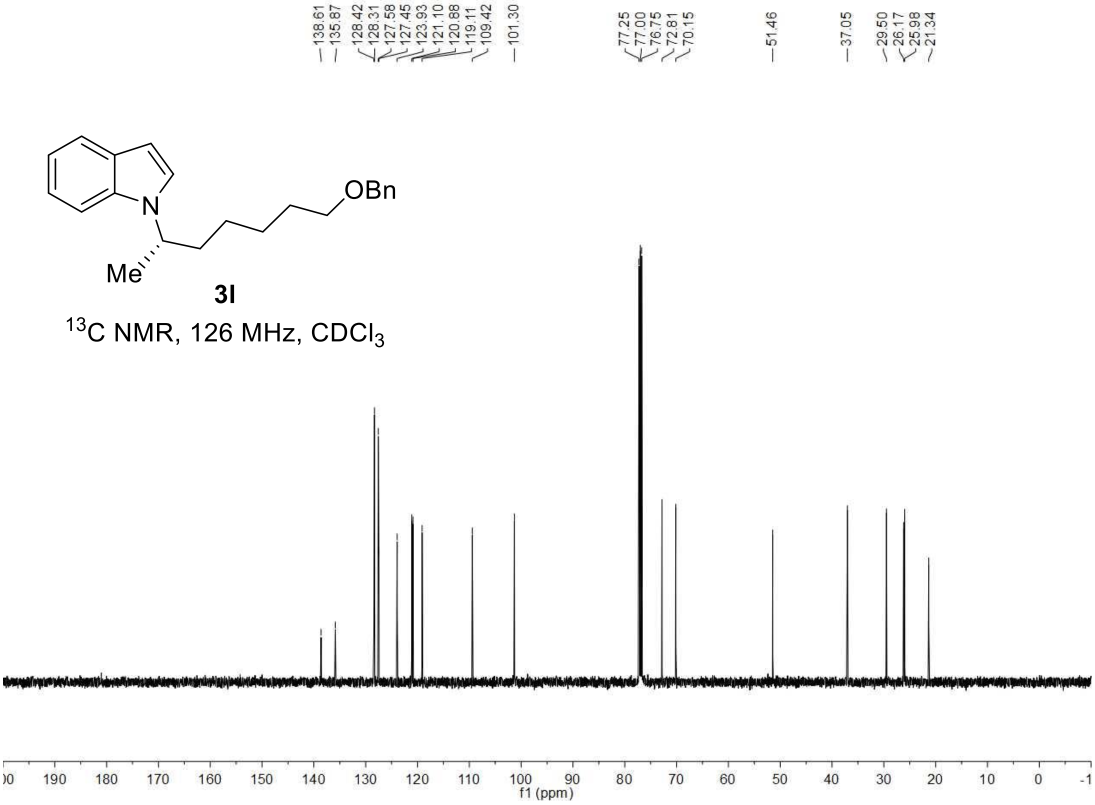

Supplementary Figure 88. ^{13}C NMR spectrum of compound **3l**

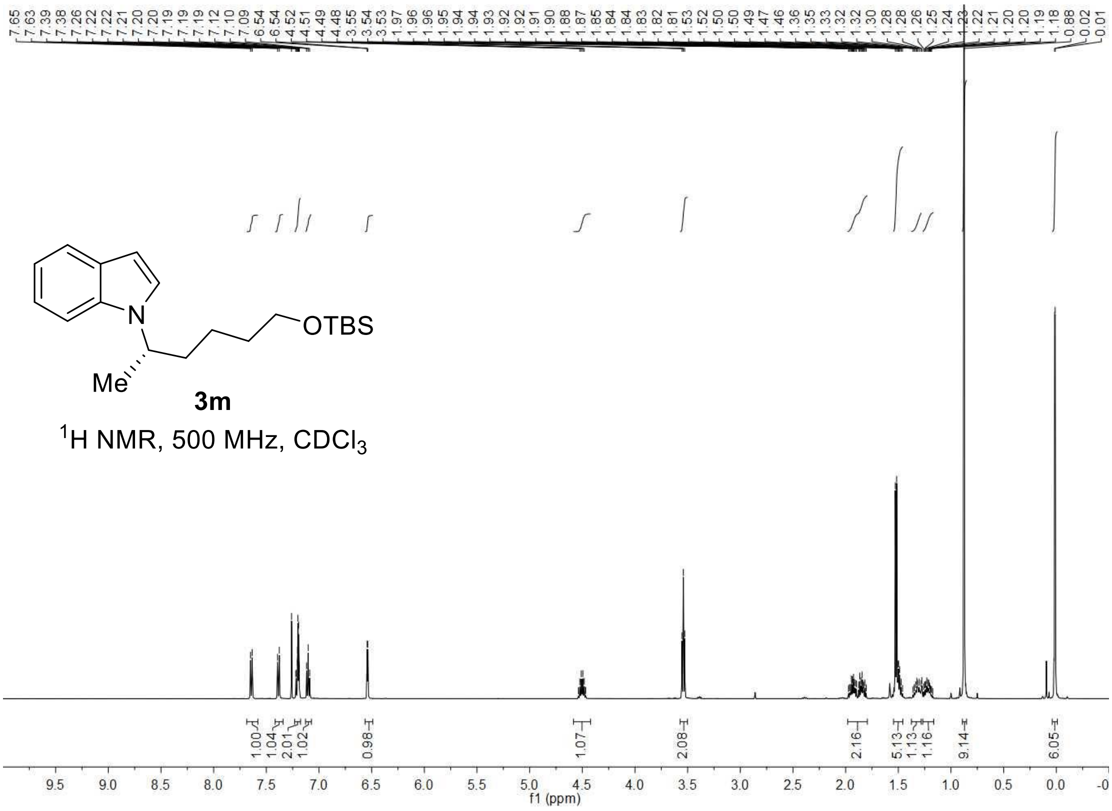

Supplementary Figure 89. ^{1}H NMR spectrum of compound **3m**

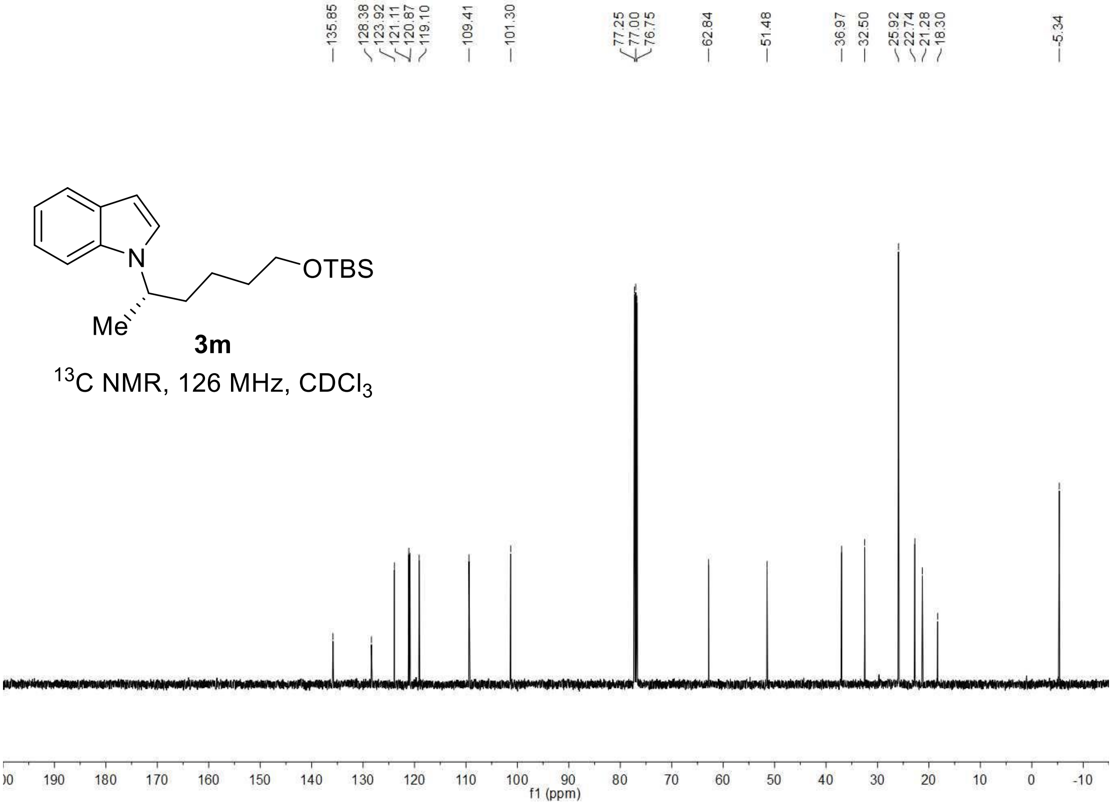

Supplementary Figure 90. ^{13}C NMR spectrum of compound **3m**

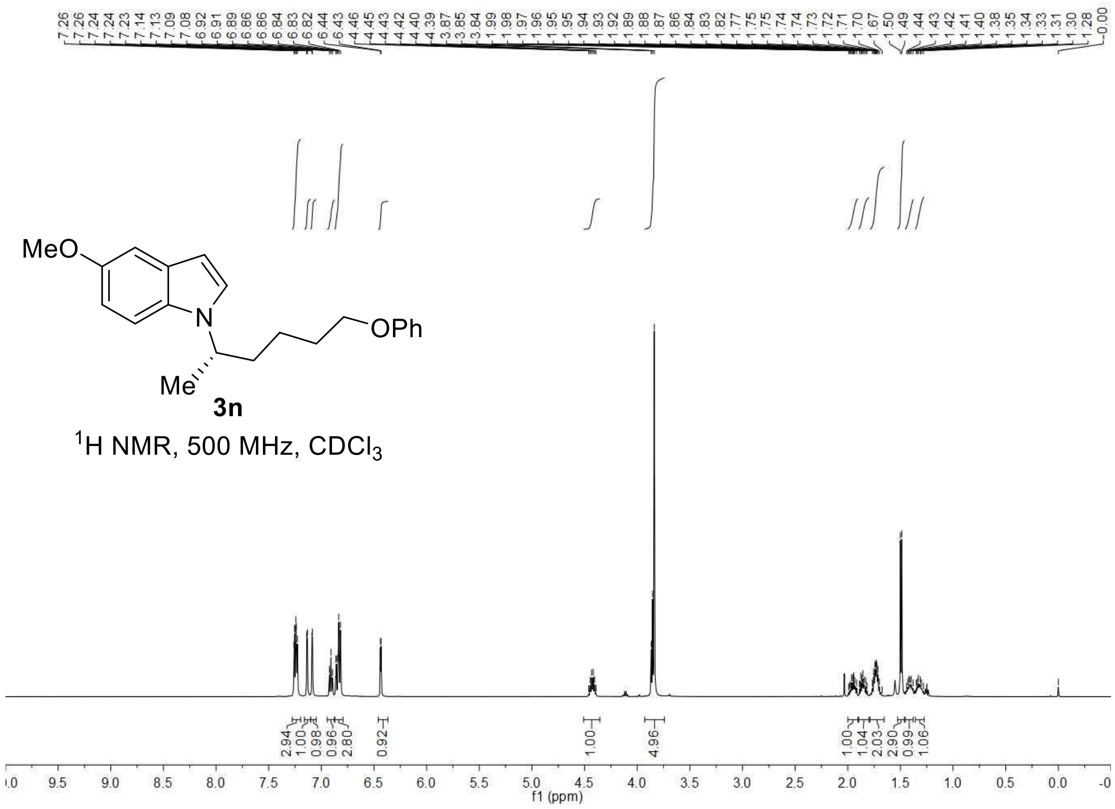

Supplementary Figure 91. ^{1}H NMR spectrum of compound **3n**

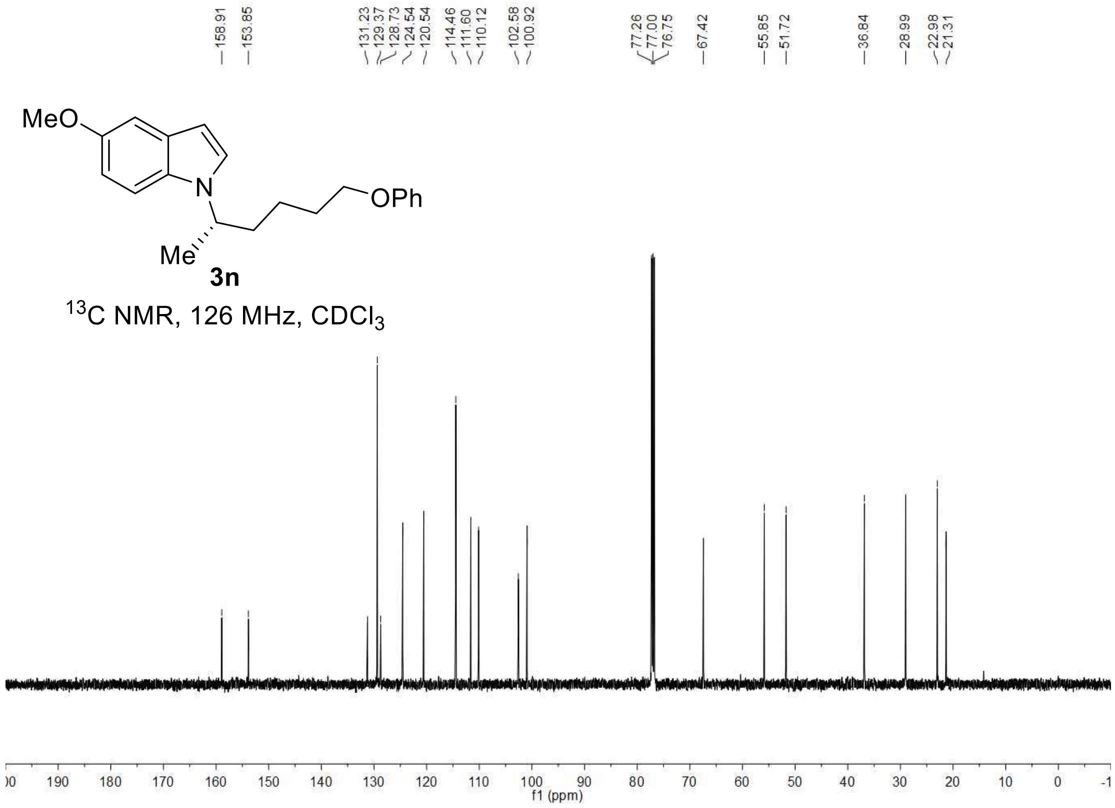

Supplementary Figure 92. ^{13}C NMR spectrum of compound **3n**

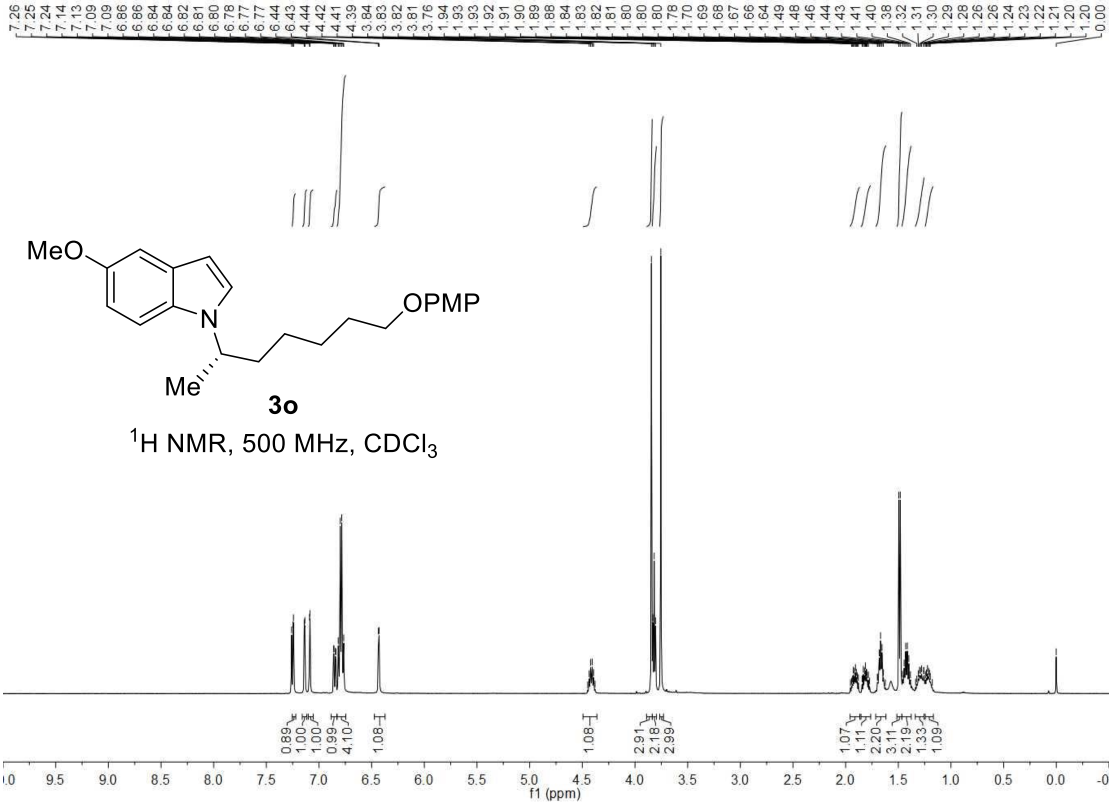

Supplementary Figure 93. ^{1}H NMR spectrum of compound **3o**

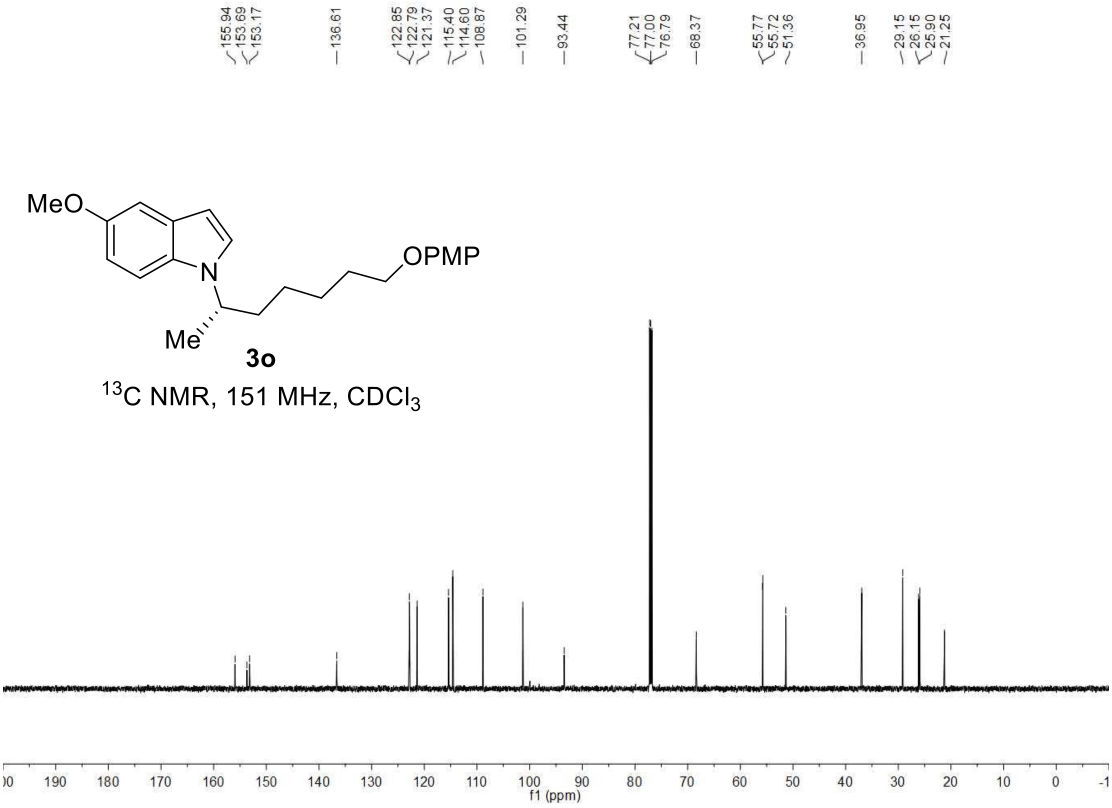

Supplementary Figure 94. ^{13}C NMR spectrum of compound **3o**

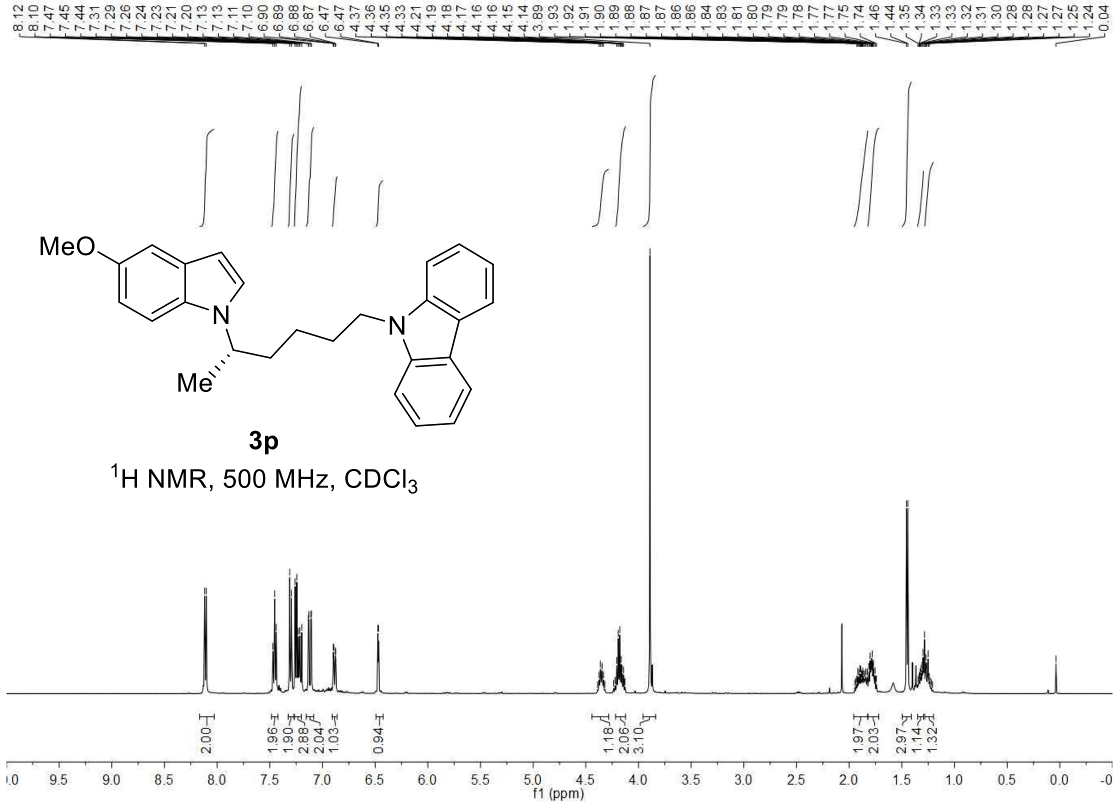

Supplementary Figure 95. ^{1}H NMR spectrum of compound **3p**

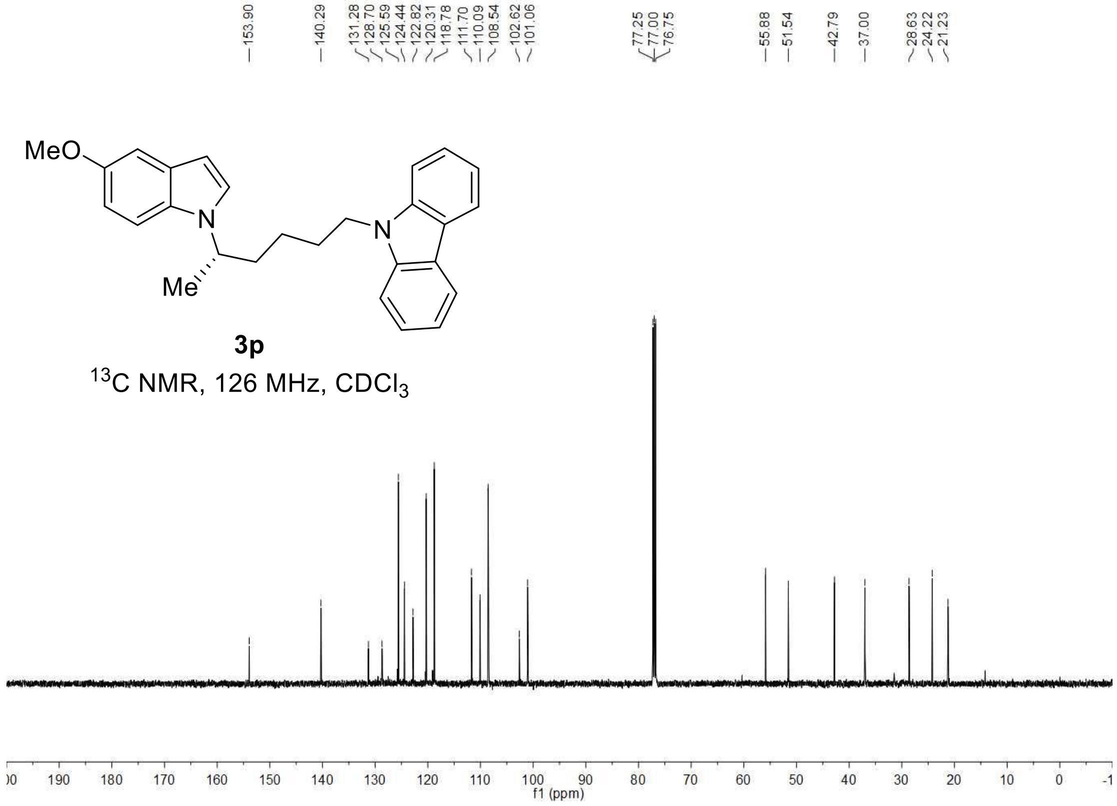

Supplementary Figure 96. ^{13}C NMR spectrum of compound **3p**

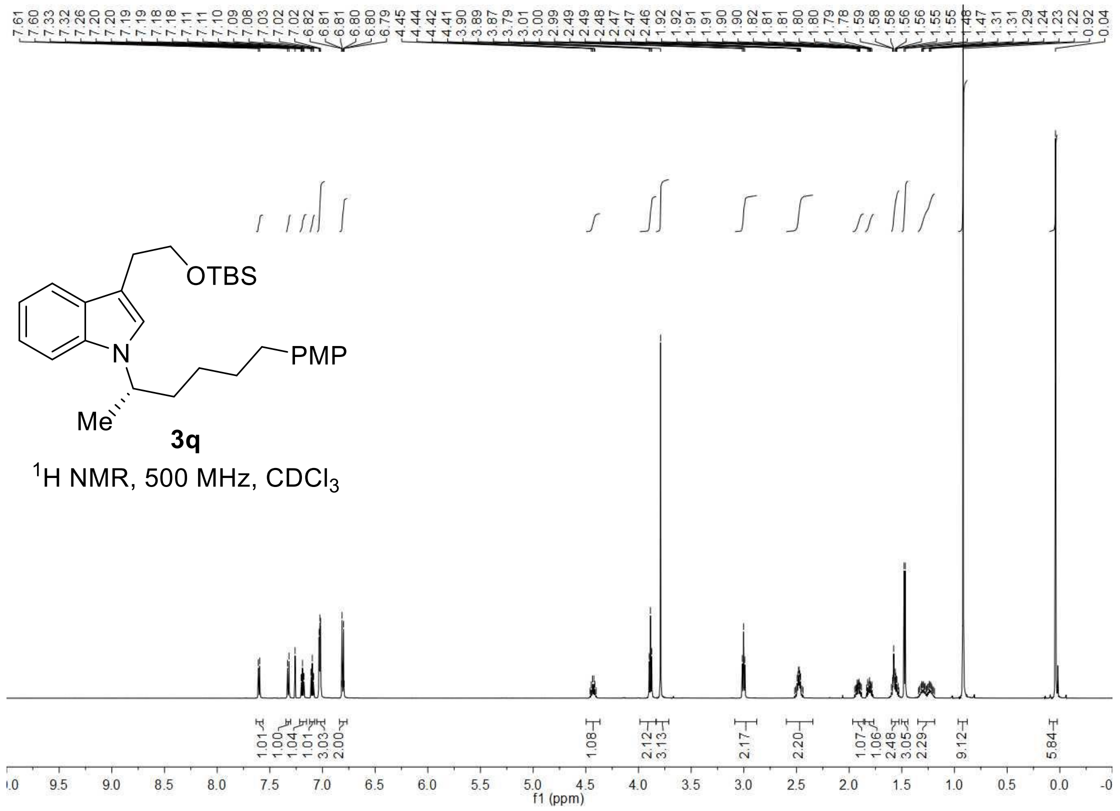

Supplementary Figure 97. ^{1}H NMR spectrum of compound **3q**

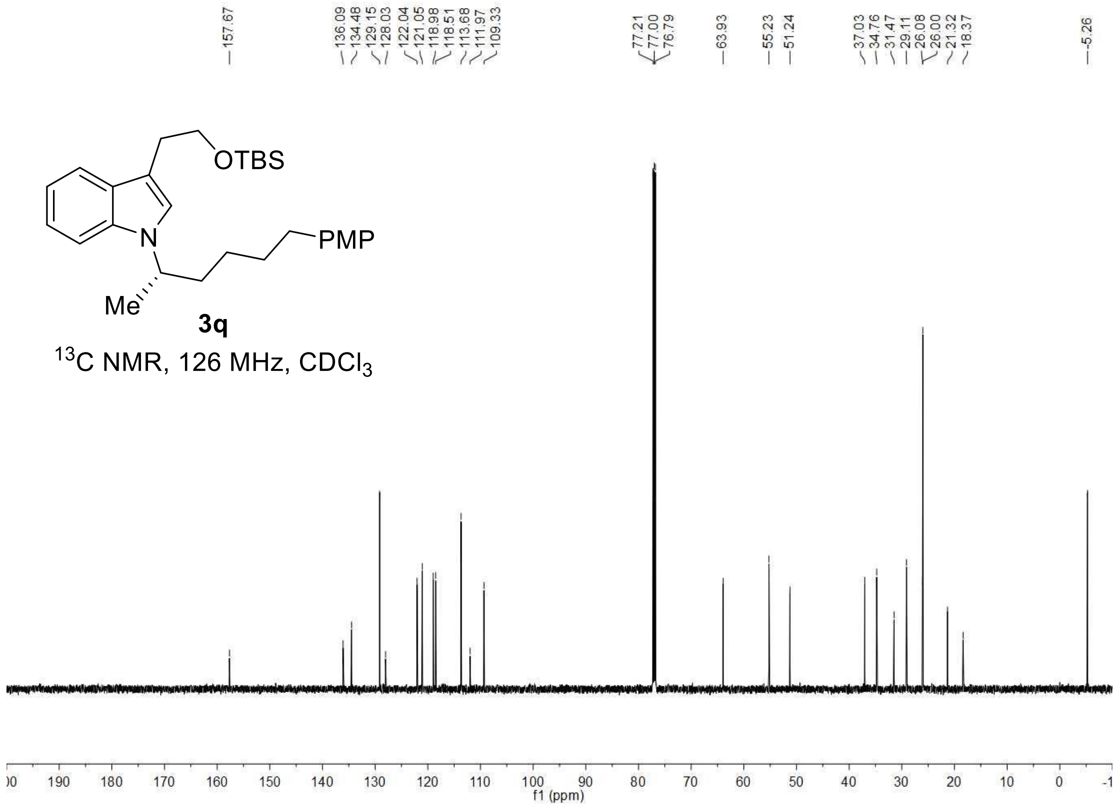

Supplementary Figure 98. ^{13}C NMR spectrum of compound **3q**

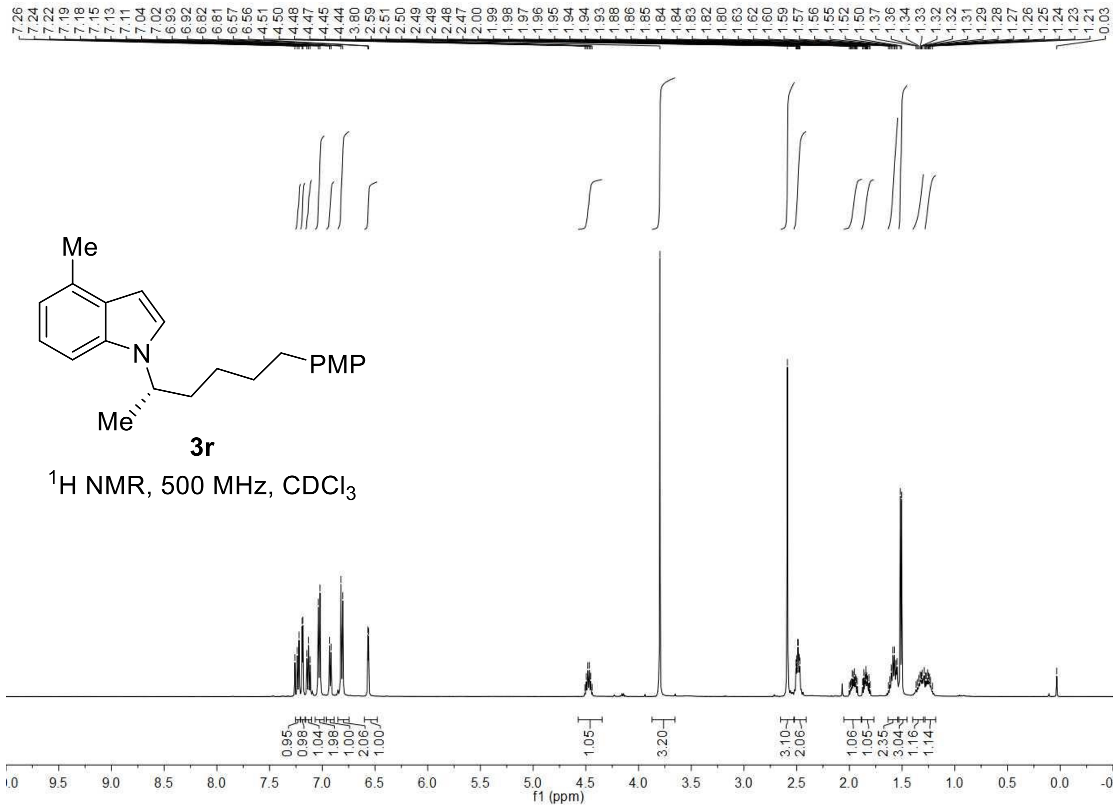

Supplementary Figure 99. ^{1}H NMR spectrum of compound **3r**

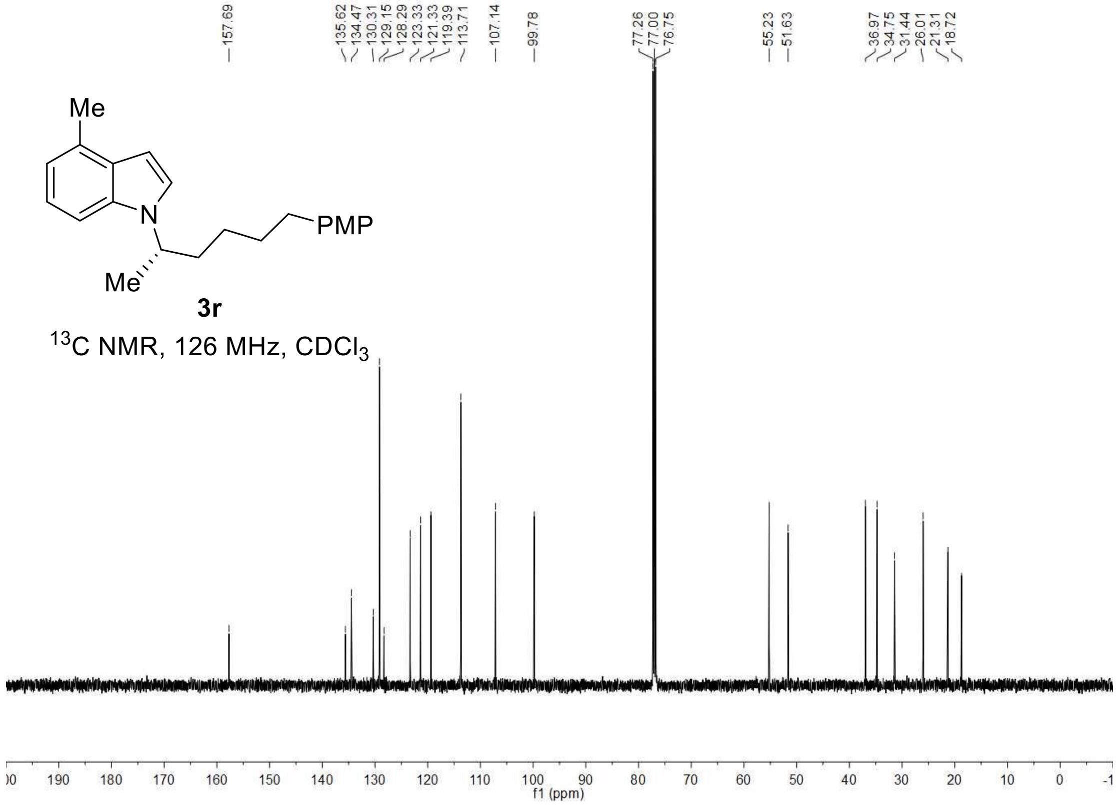

Supplementary Figure 100. ^{13}C NMR spectrum of compound **3r**

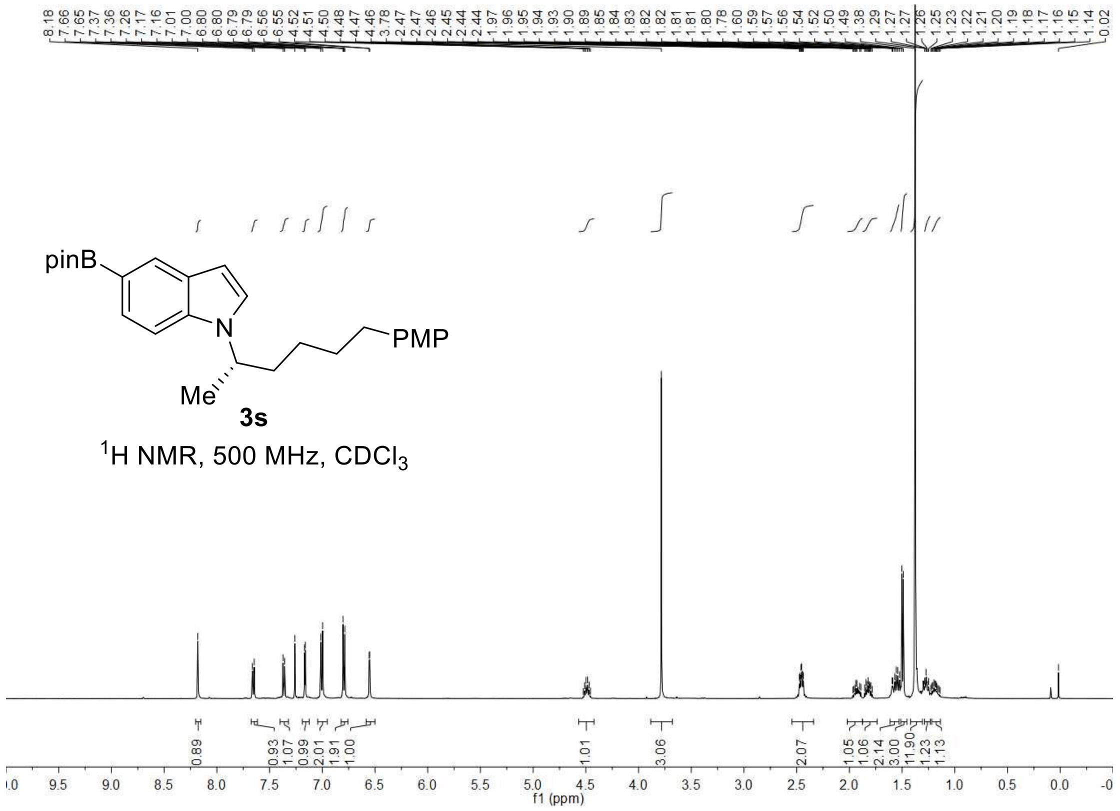

Supplementary Figure 101. ^{1}H NMR spectrum of compound **3s**

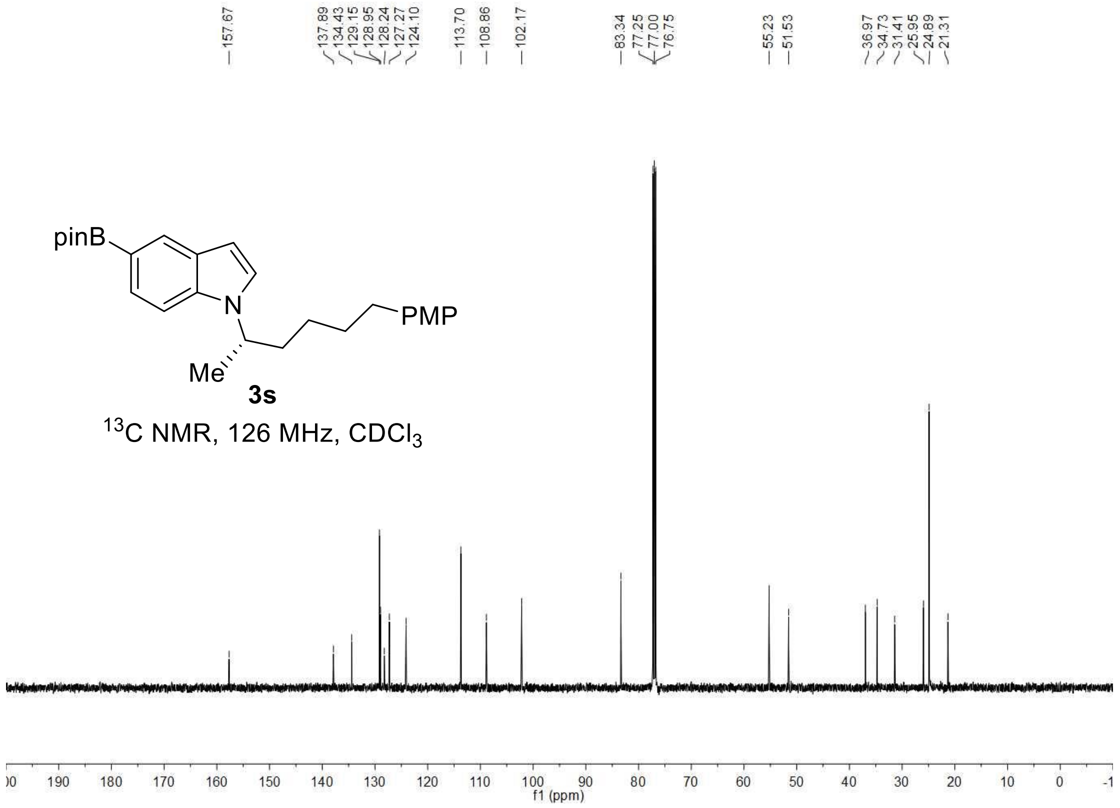

Supplementary Figure 102. ^{13}C NMR spectrum of compound **3s**

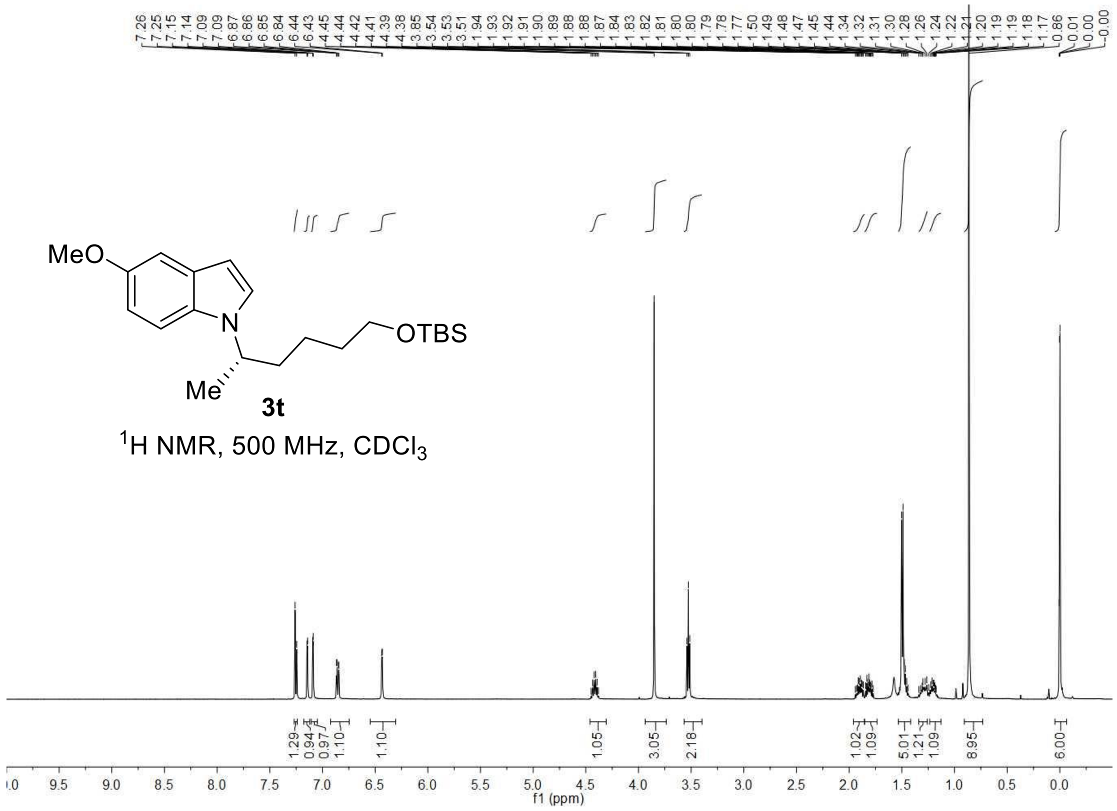

Supplementary Figure 103. ^{1}H NMR spectrum of compound **3t**

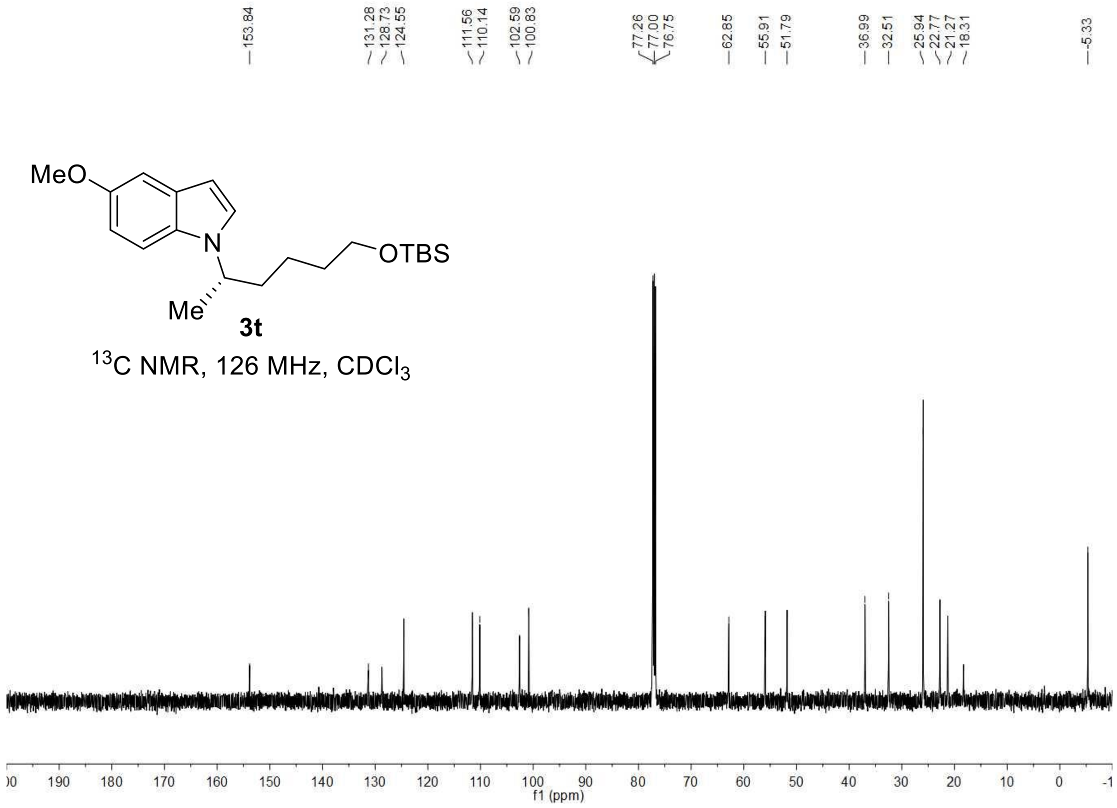

Supplementary Figure 104. ^{13}C NMR spectrum of compound **3t**

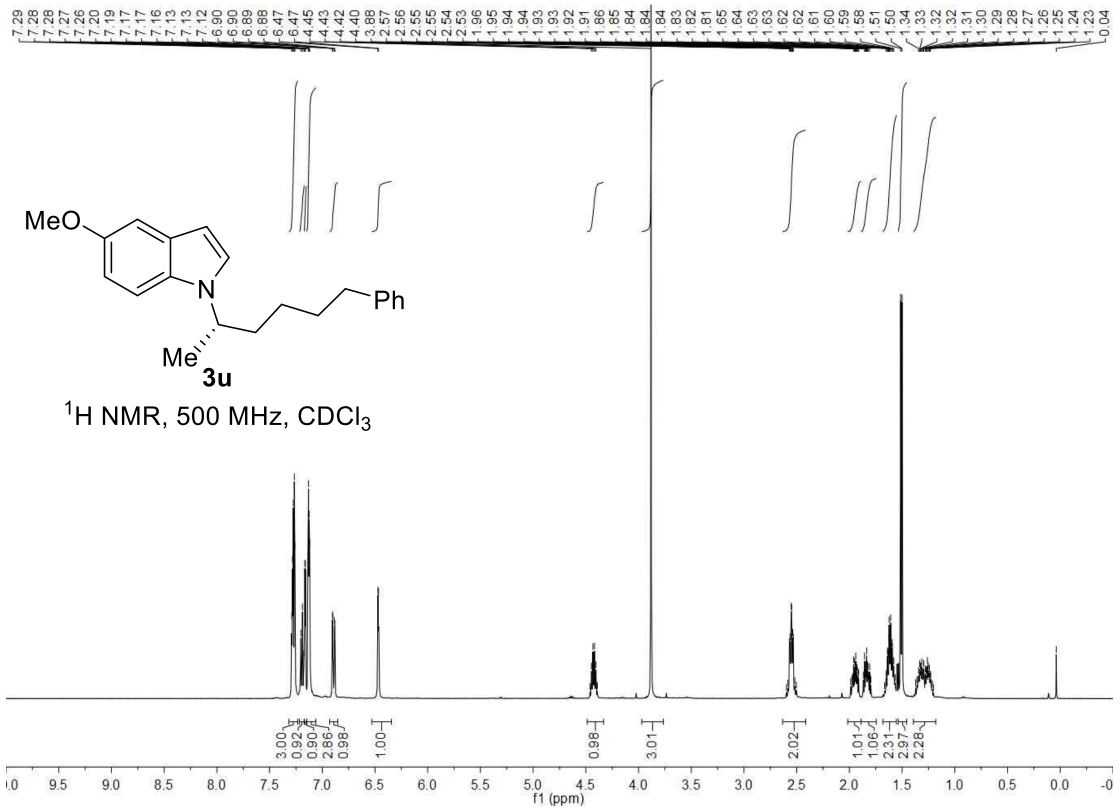

Supplementary Figure 105. ^{1}H NMR spectrum of compound **3u**

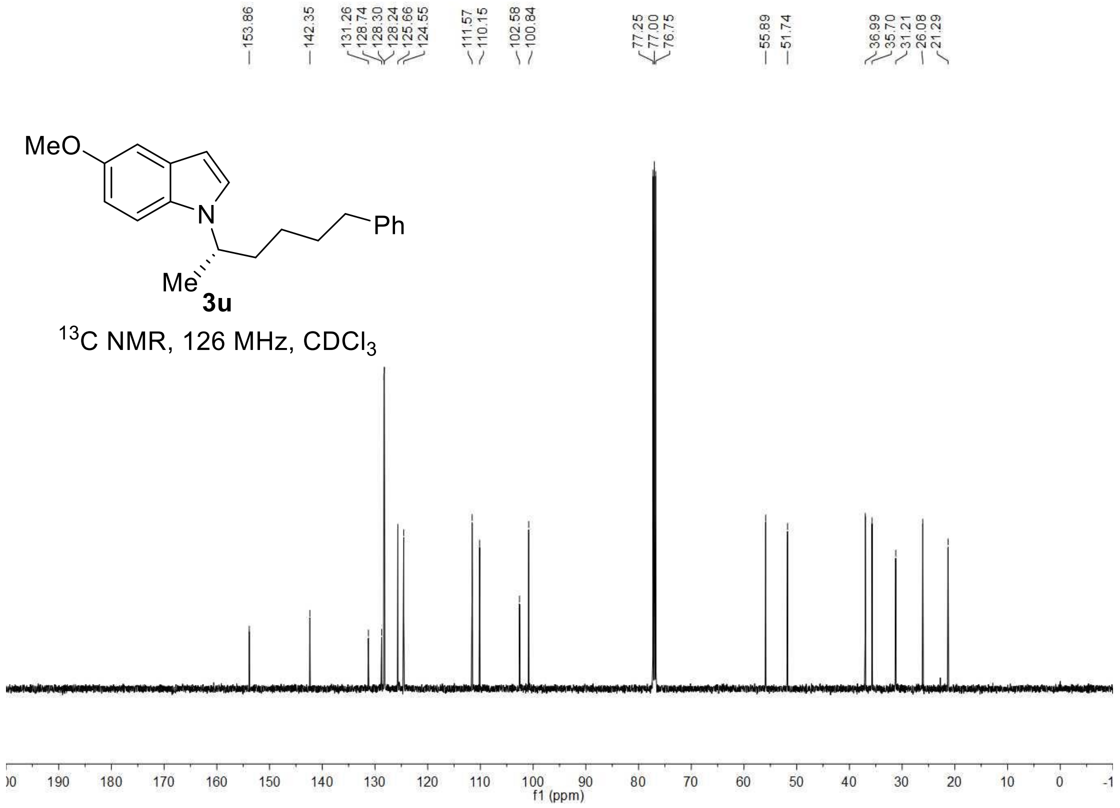

Supplementary Figure 106. ^{13}C NMR spectrum of compound **3u**

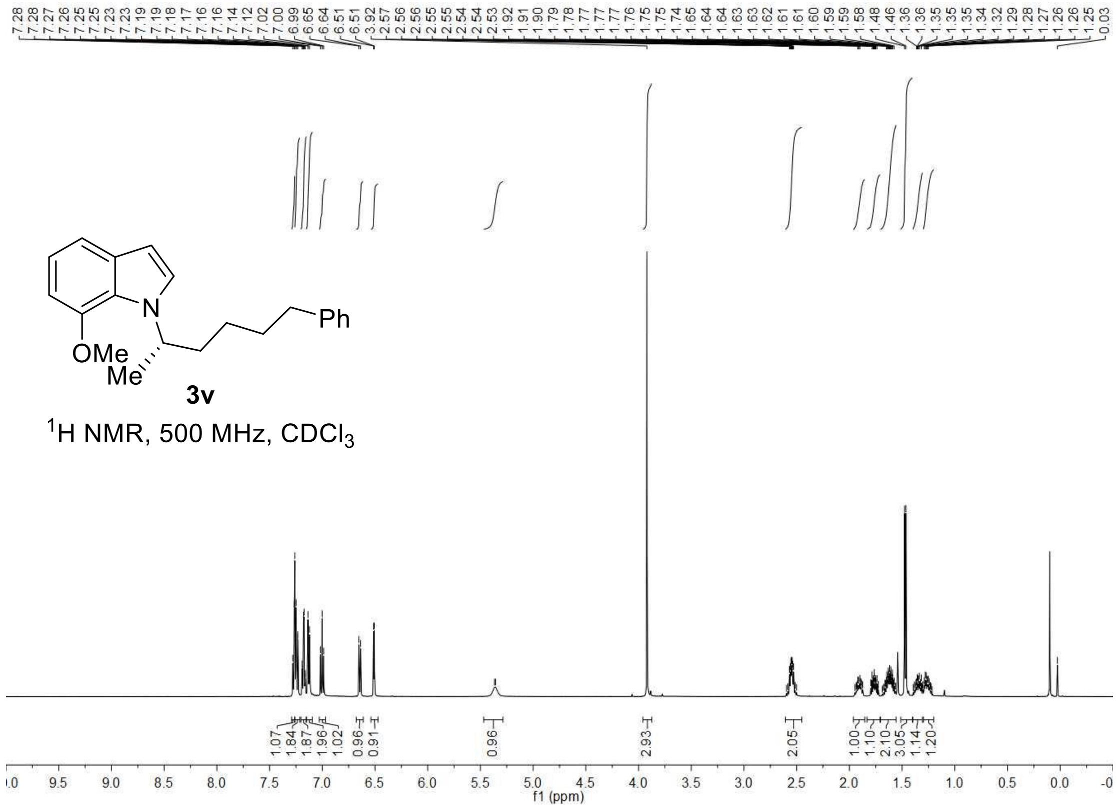

Supplementary Figure 107. ^{1}H NMR spectrum of compound **3v**

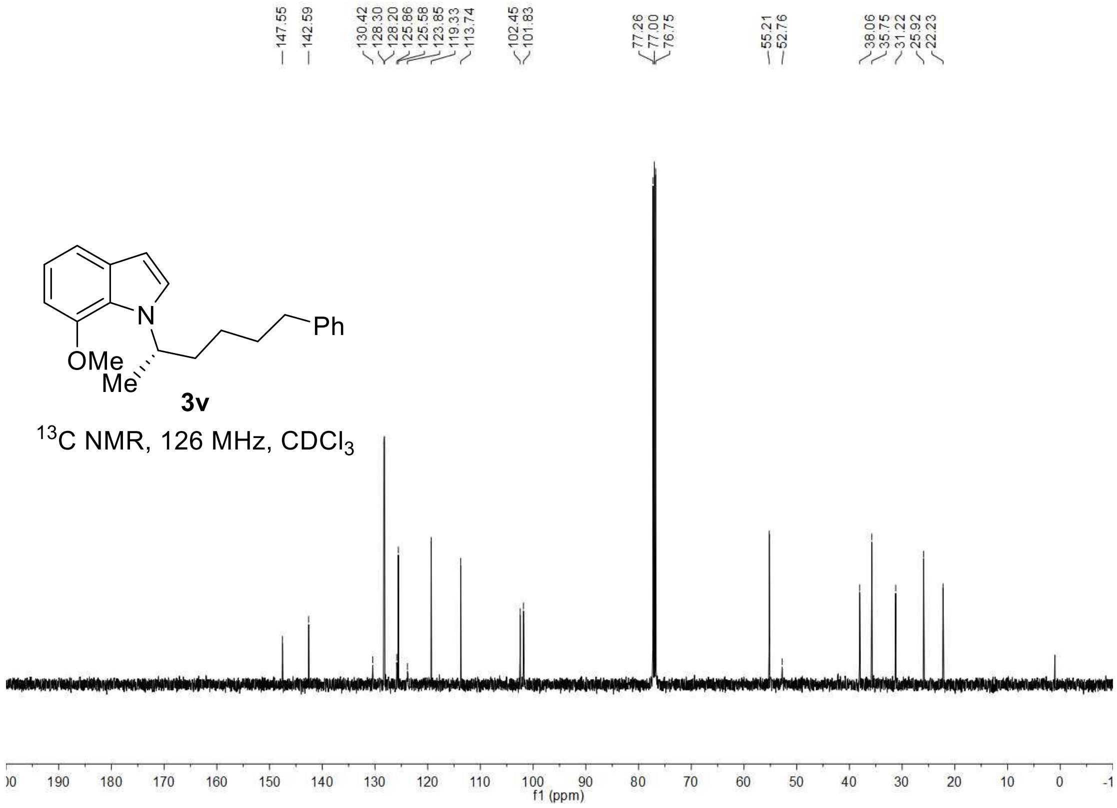

Supplementary Figure 108. ^{13}C NMR spectrum of compound **3v**

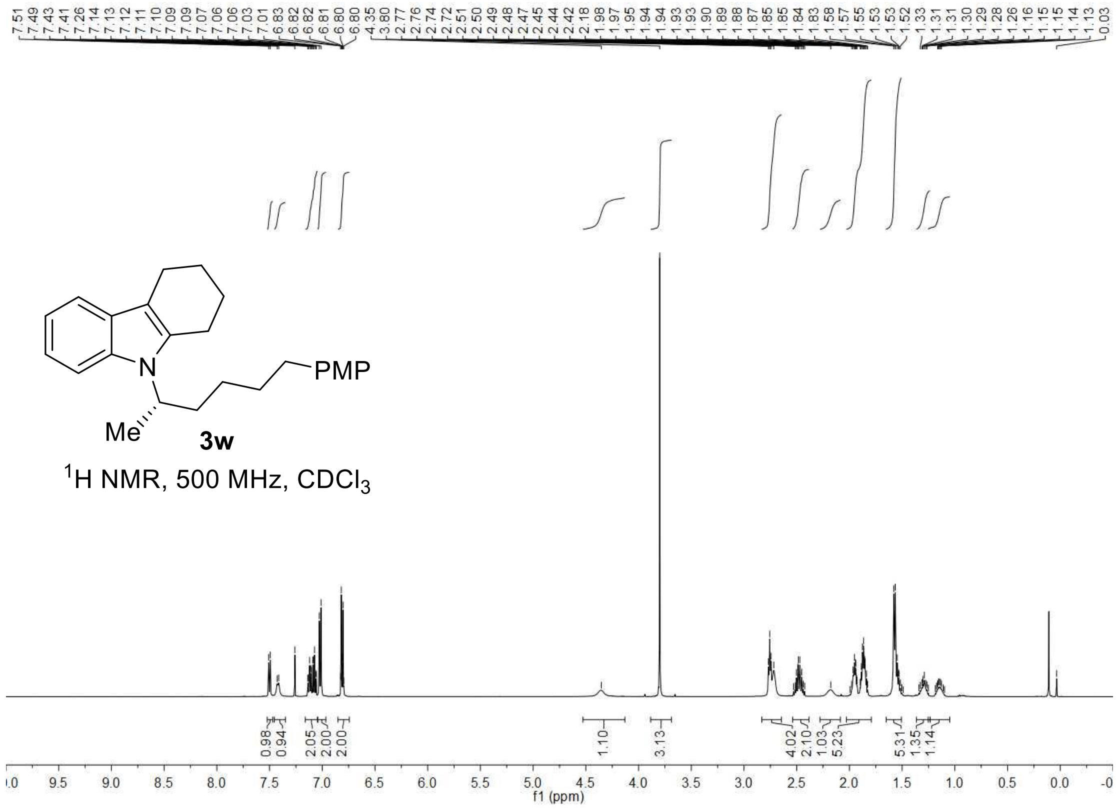

Supplementary Figure 109. ^{1}H NMR spectrum of compound **3w**

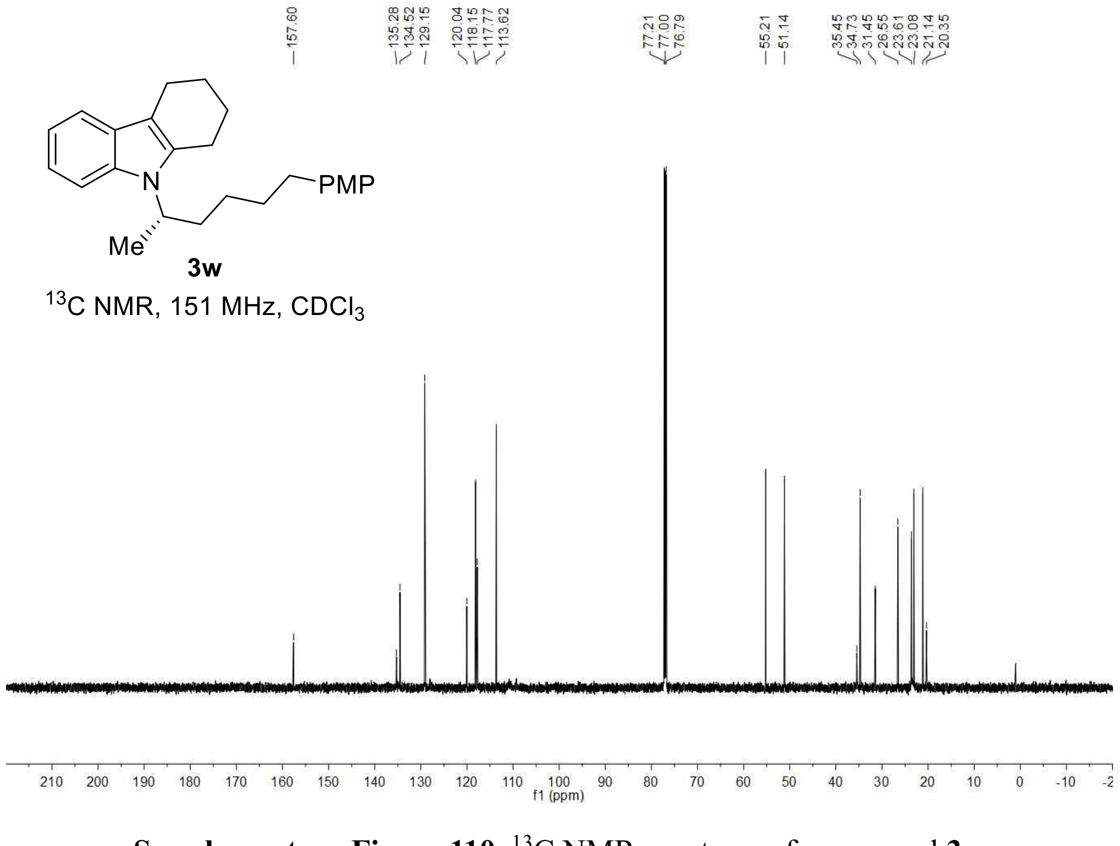

Supplementary Figure 110. ^{13}C NMR spectrum of compound **3w**

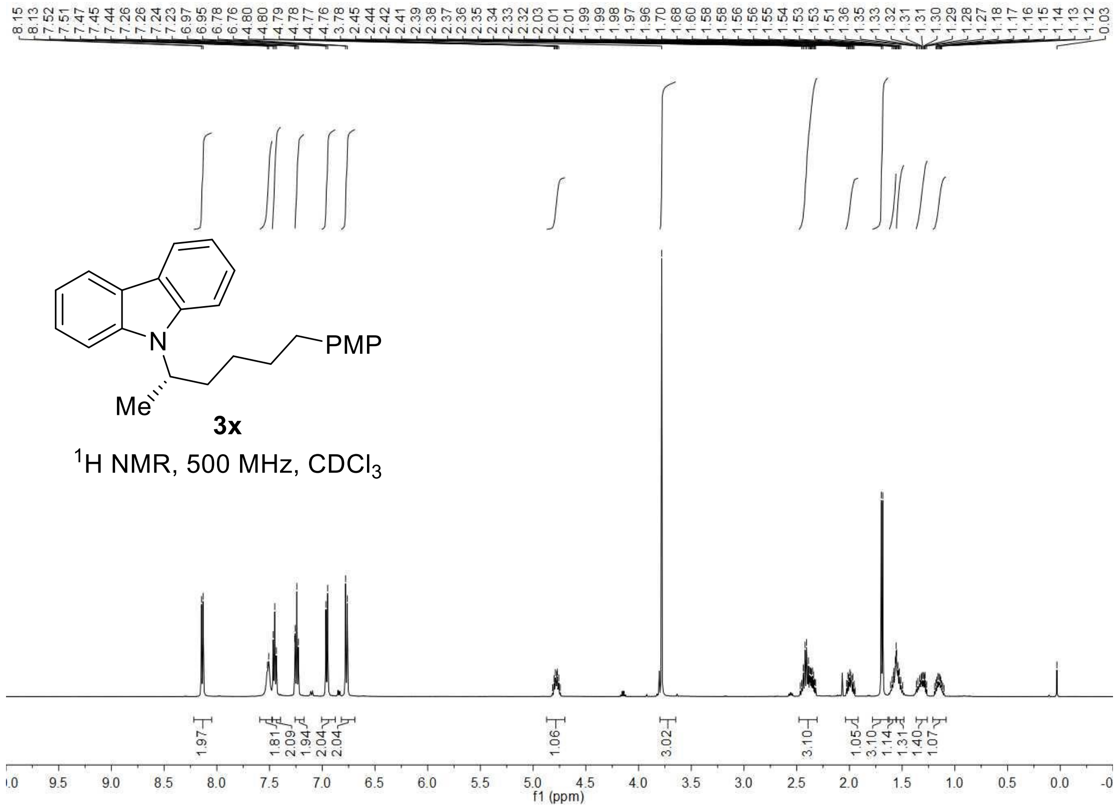

Supplementary Figure 111. ^{1}H NMR spectrum of compound **3x**

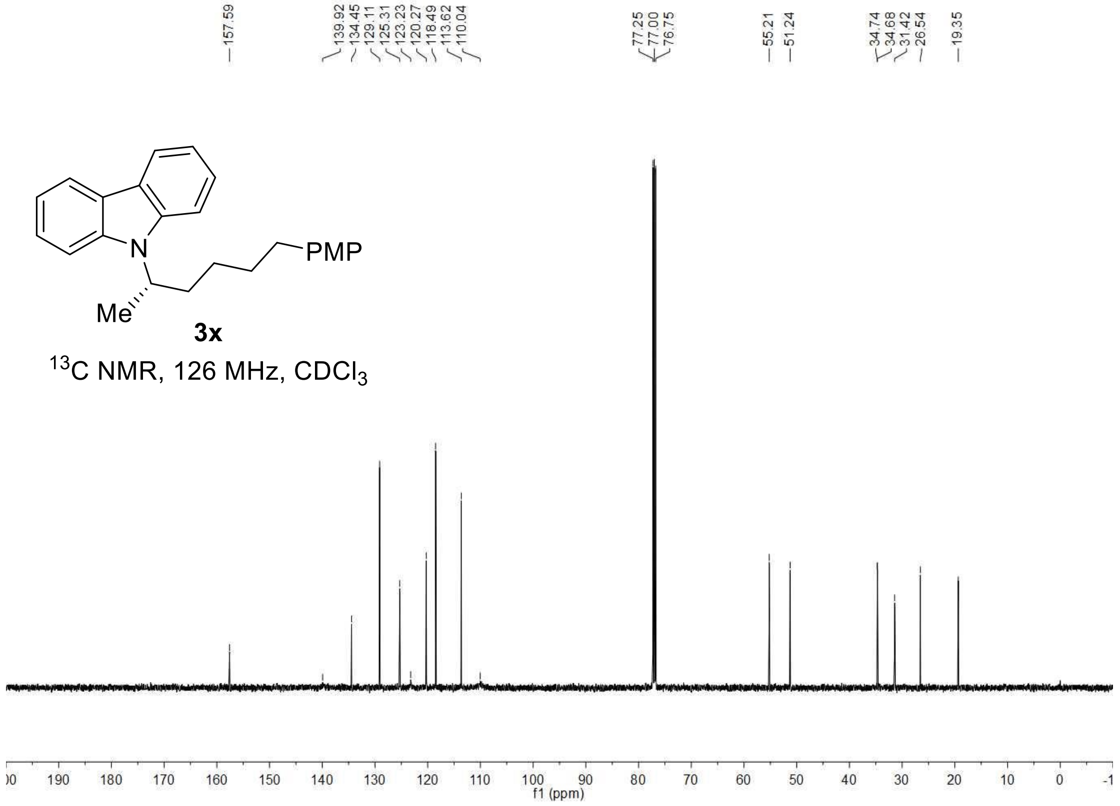

Supplementary Figure 112. ^{13}C NMR spectrum of compound **3x**

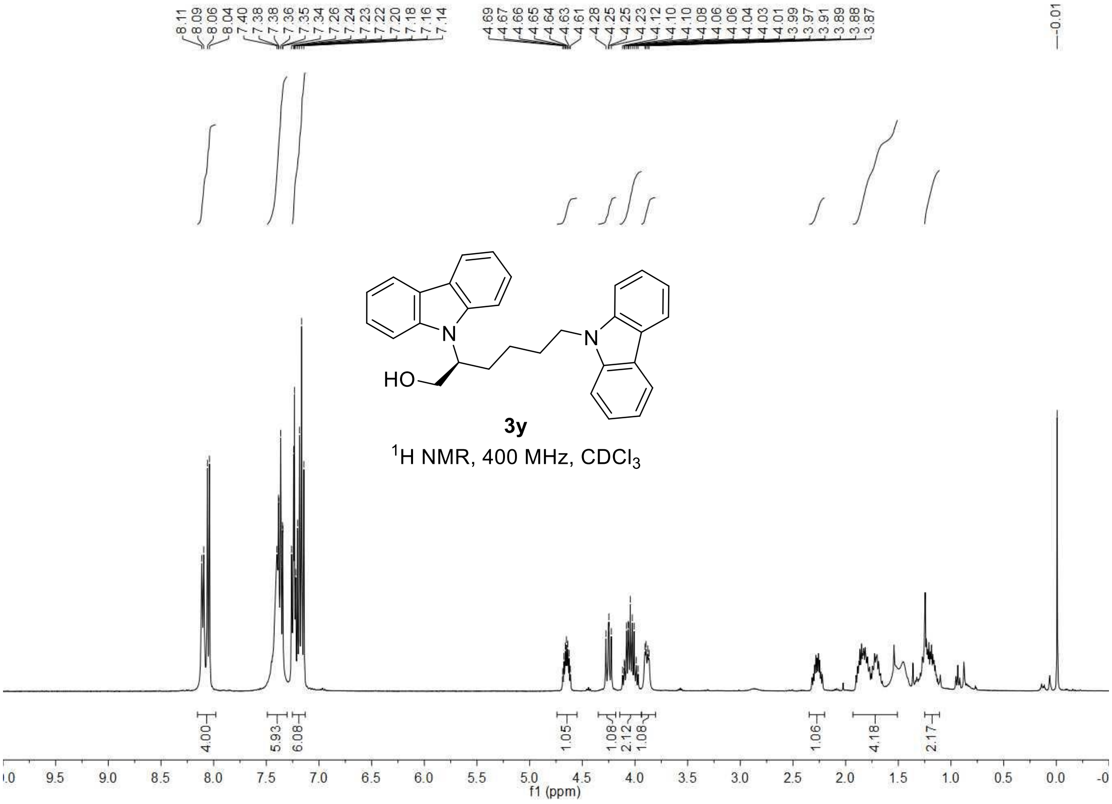

Supplementary Figure 113. ^{1}H NMR spectrum of compound **3y**

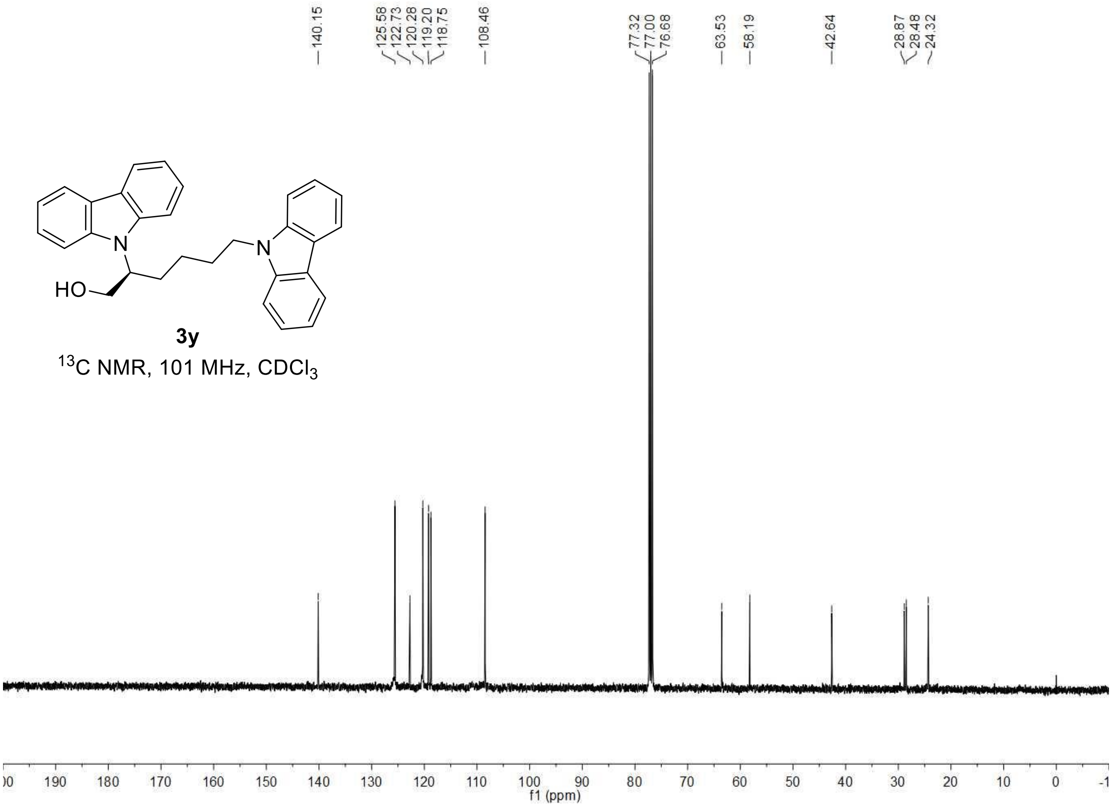

Supplementary Figure 114. ^{13}C NMR spectrum of compound **3y**

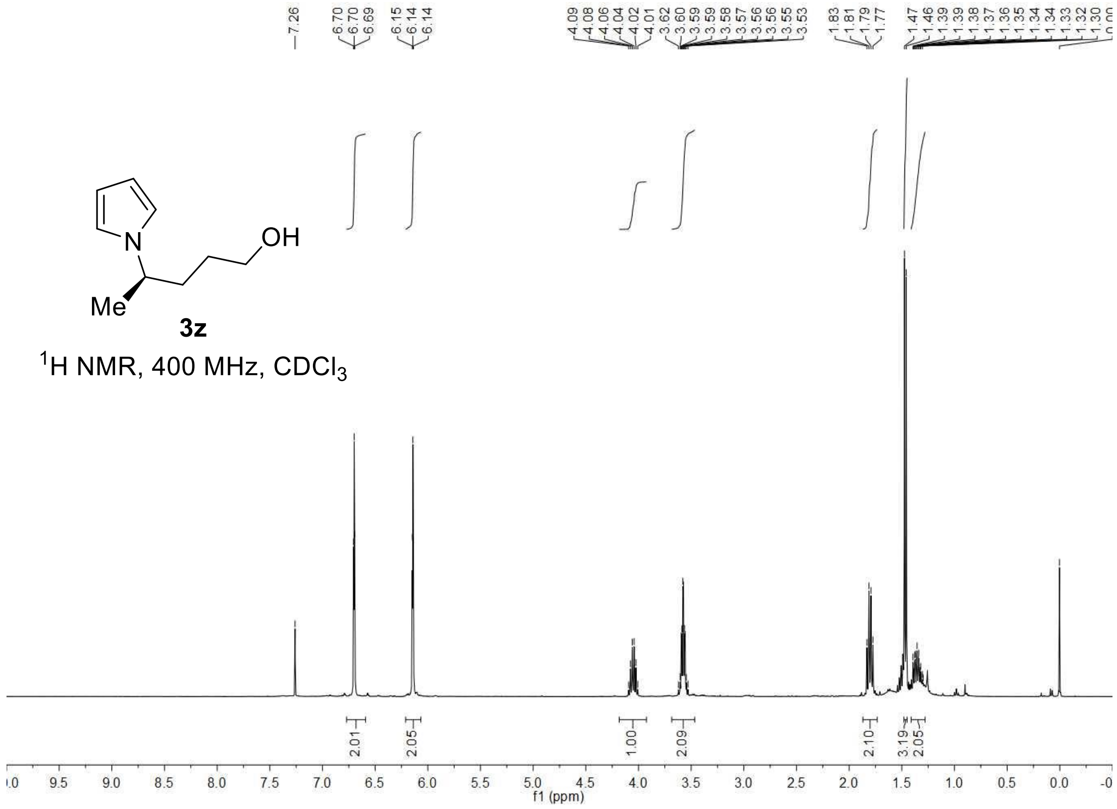

Supplementary Figure 115. ^{1}H NMR spectrum of compound **3z**

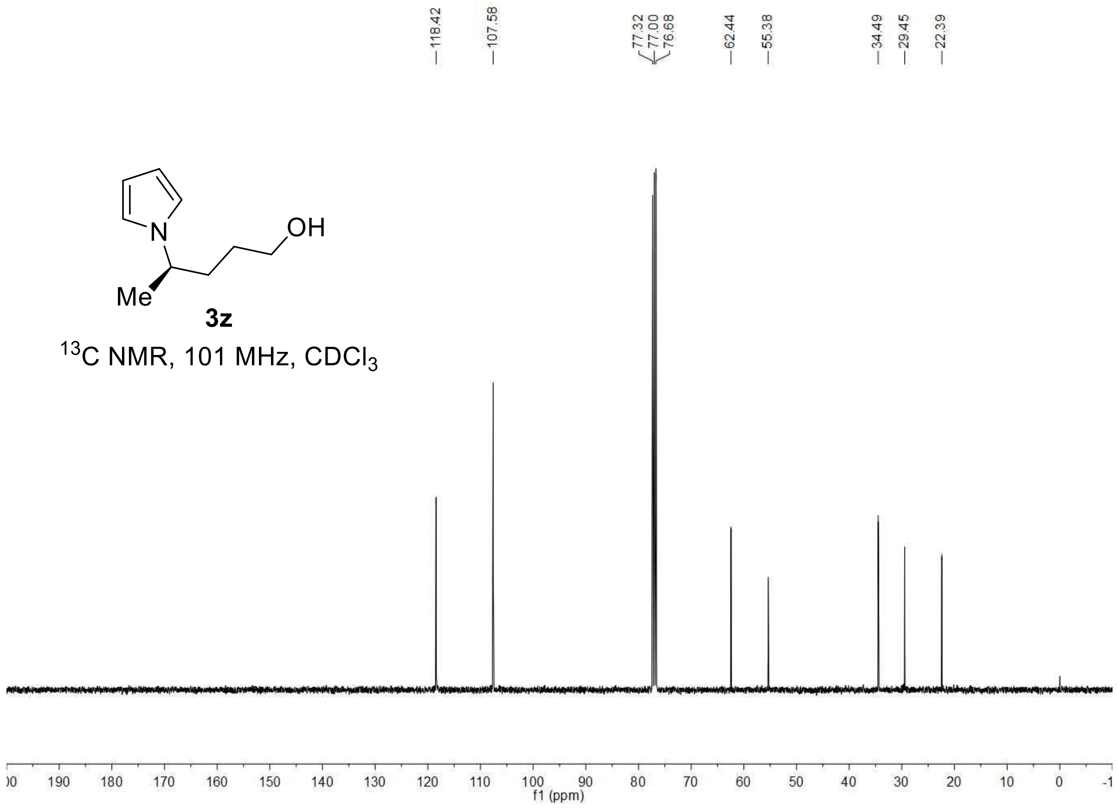

Supplementary Figure 116. ^{13}C NMR spectrum of compound **3z**

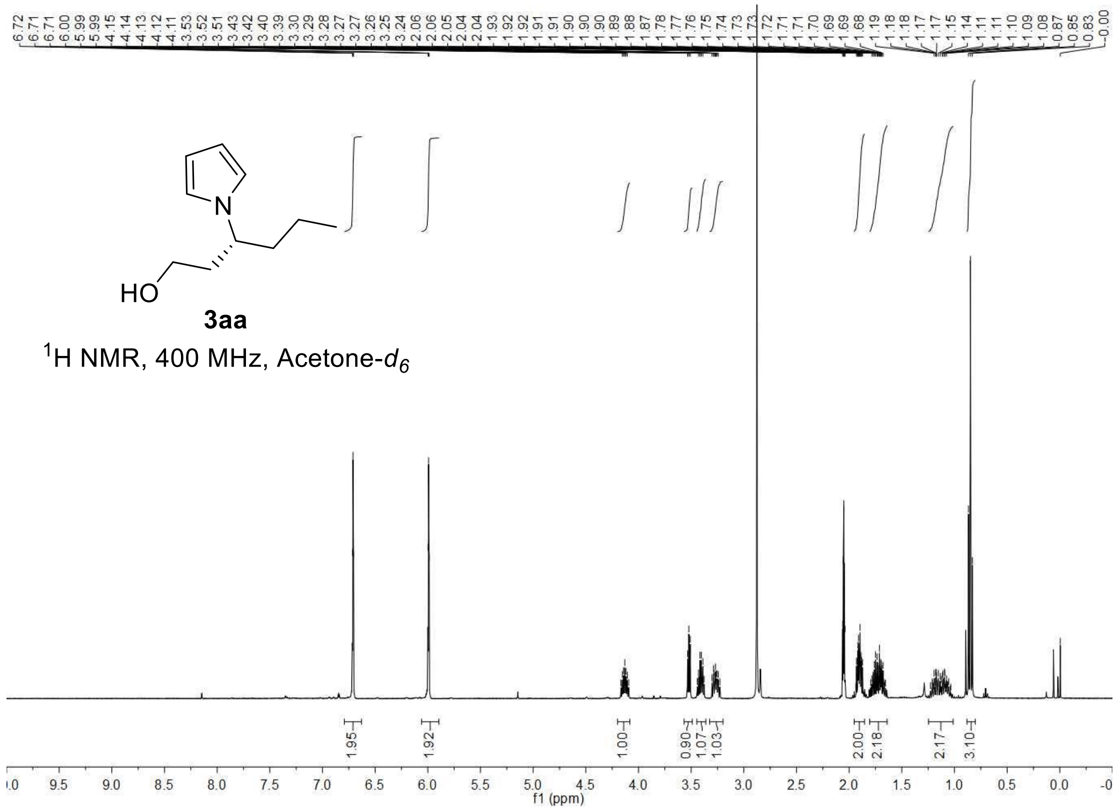

Supplementary Figure 117. ^{1}H NMR spectrum of compound **3aa**

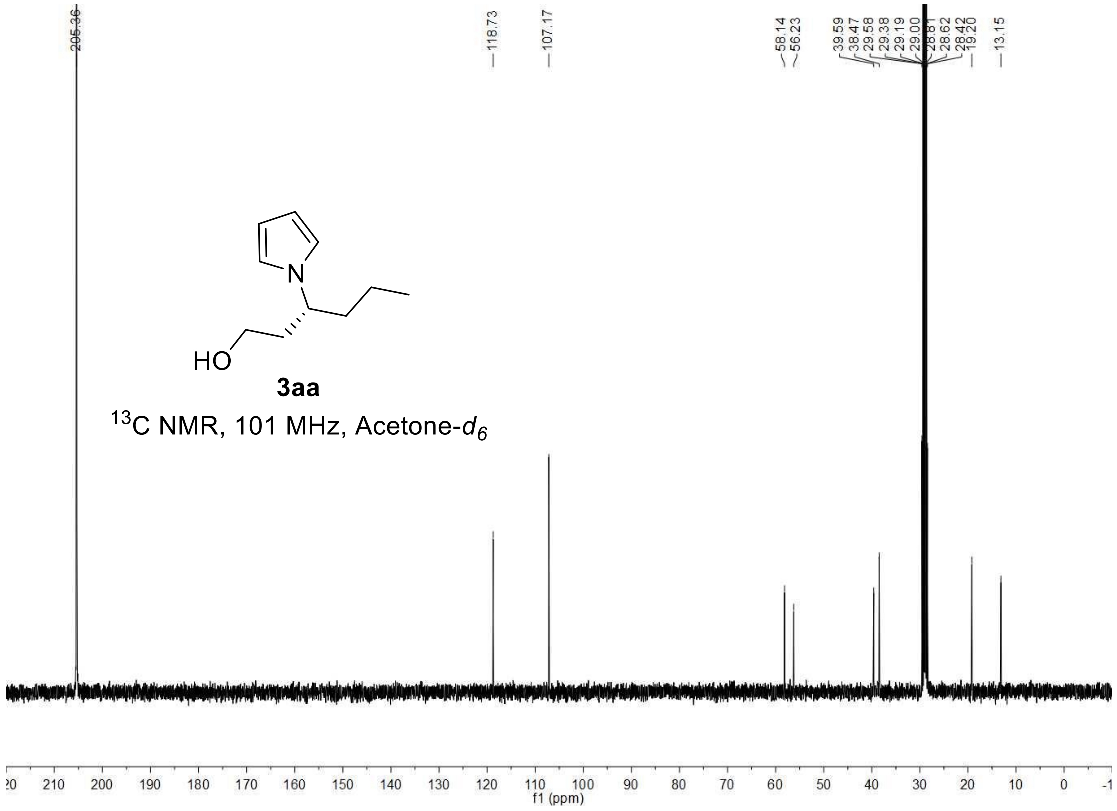

Supplementary Figure 118. ^{13}C NMR spectrum of compound **3aa**

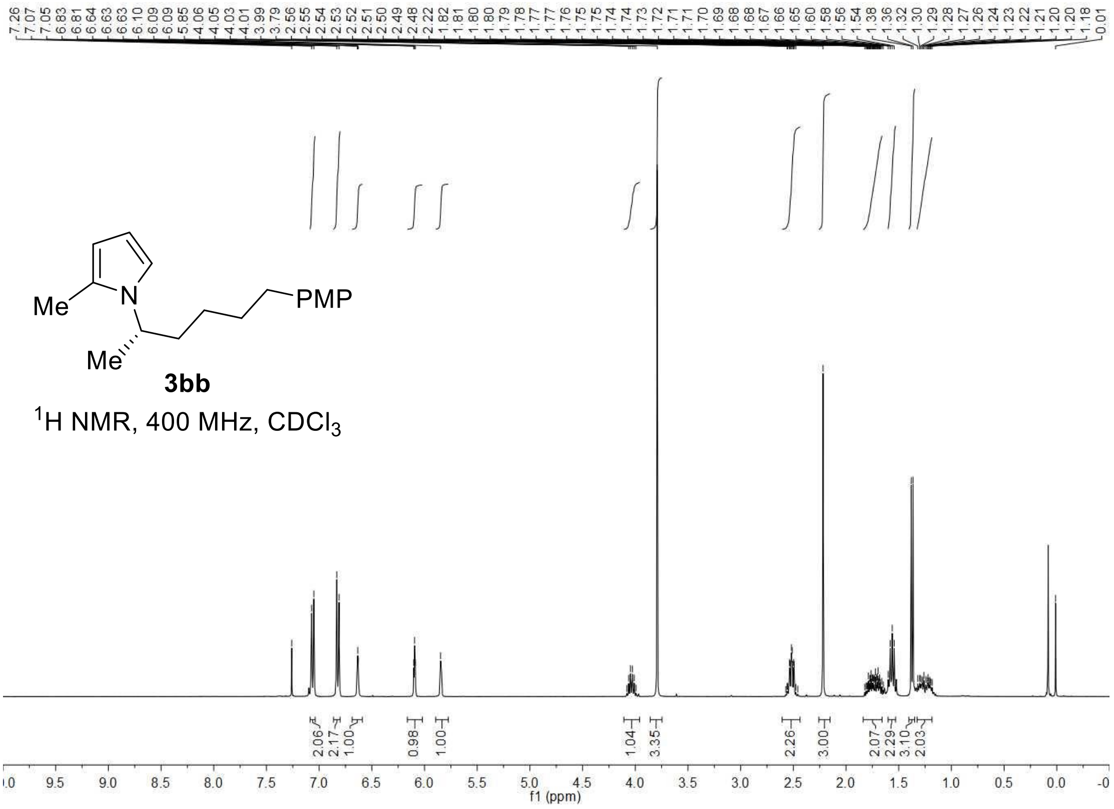

Supplementary Figure 119. ^{1}H NMR spectrum of compound **3bb**

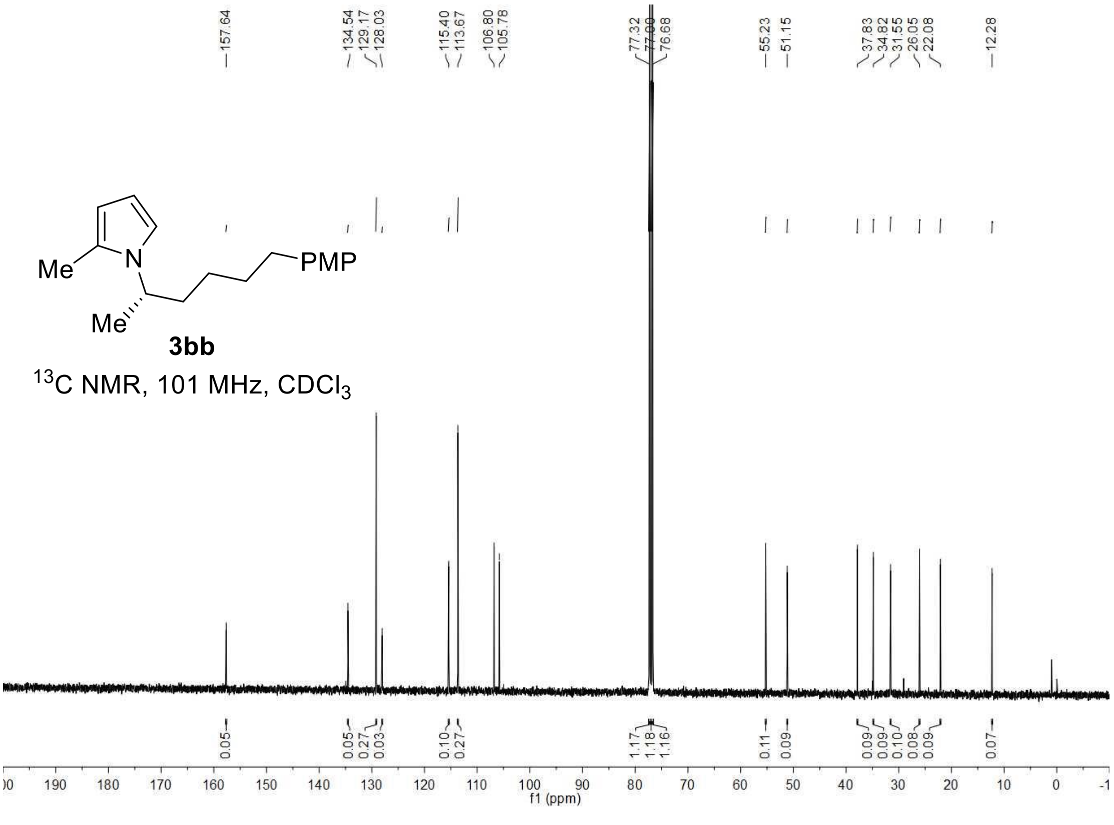

Supplementary Figure 120. ^{13}C NMR spectrum of compound **3bb**

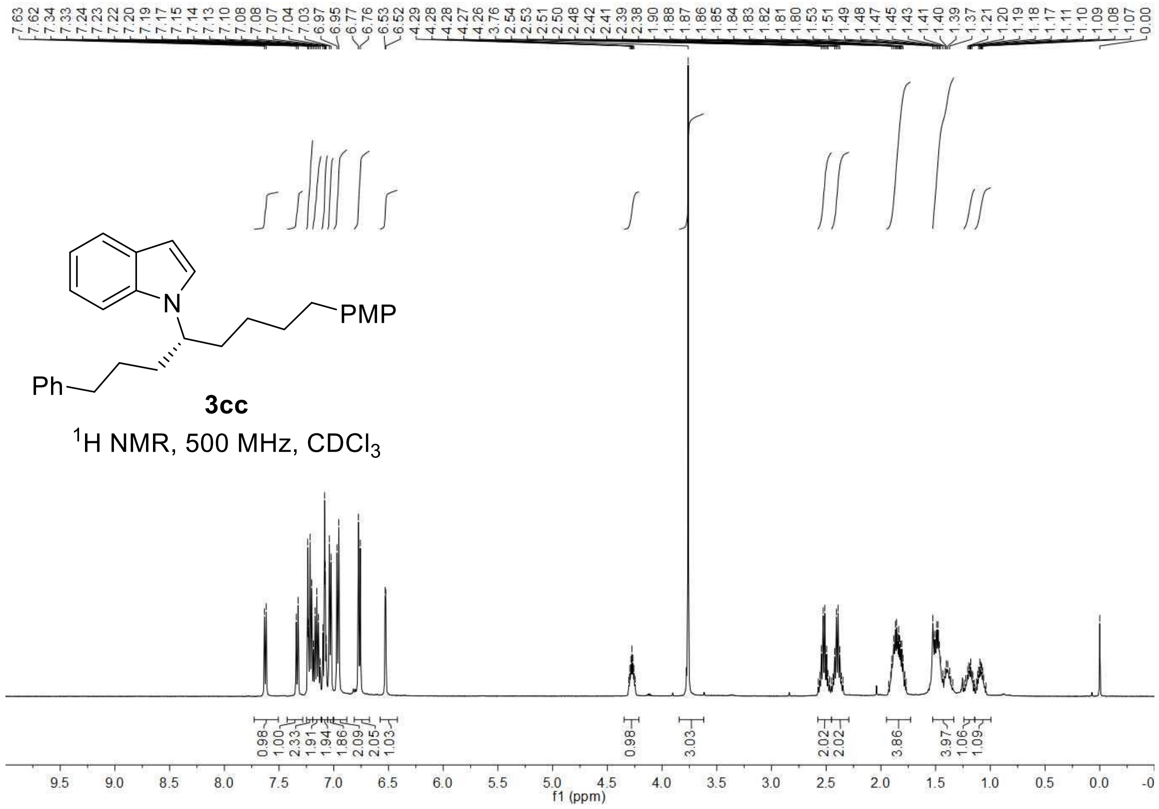

Supplementary Figure 121. ^{1}H NMR spectrum of compound **3cc**

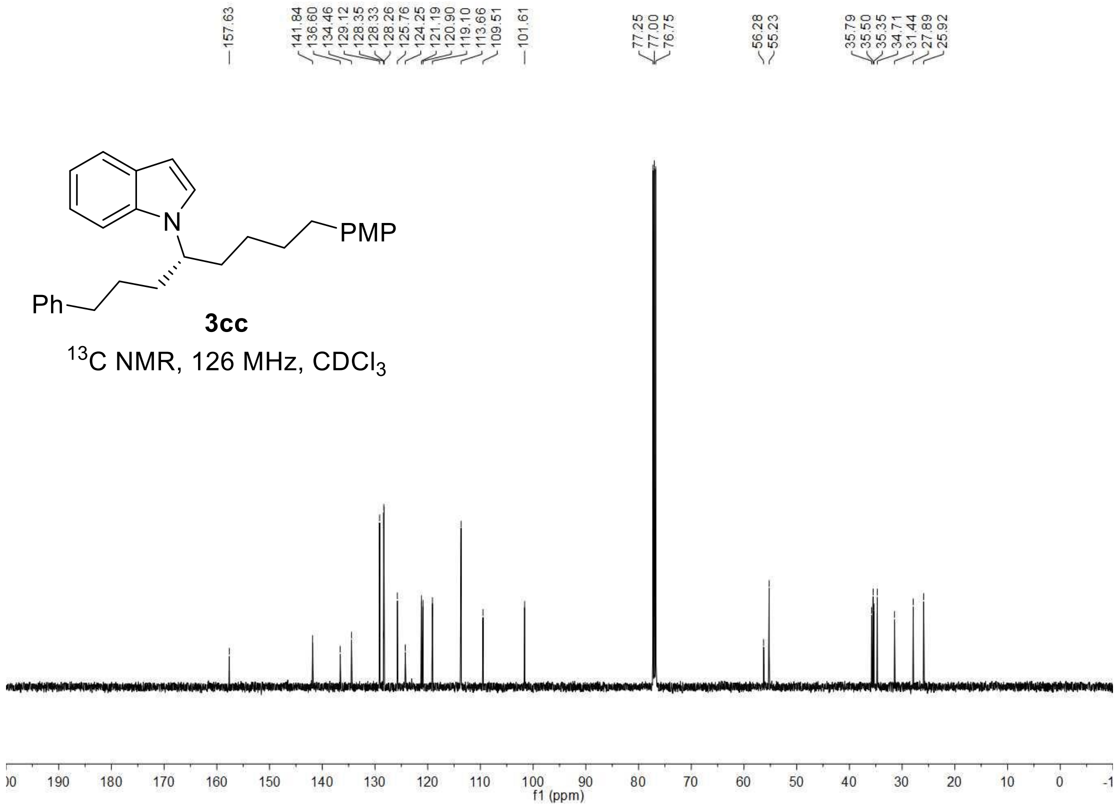

Supplementary Figure 122. ^{13}C NMR spectrum of compound **3cc**

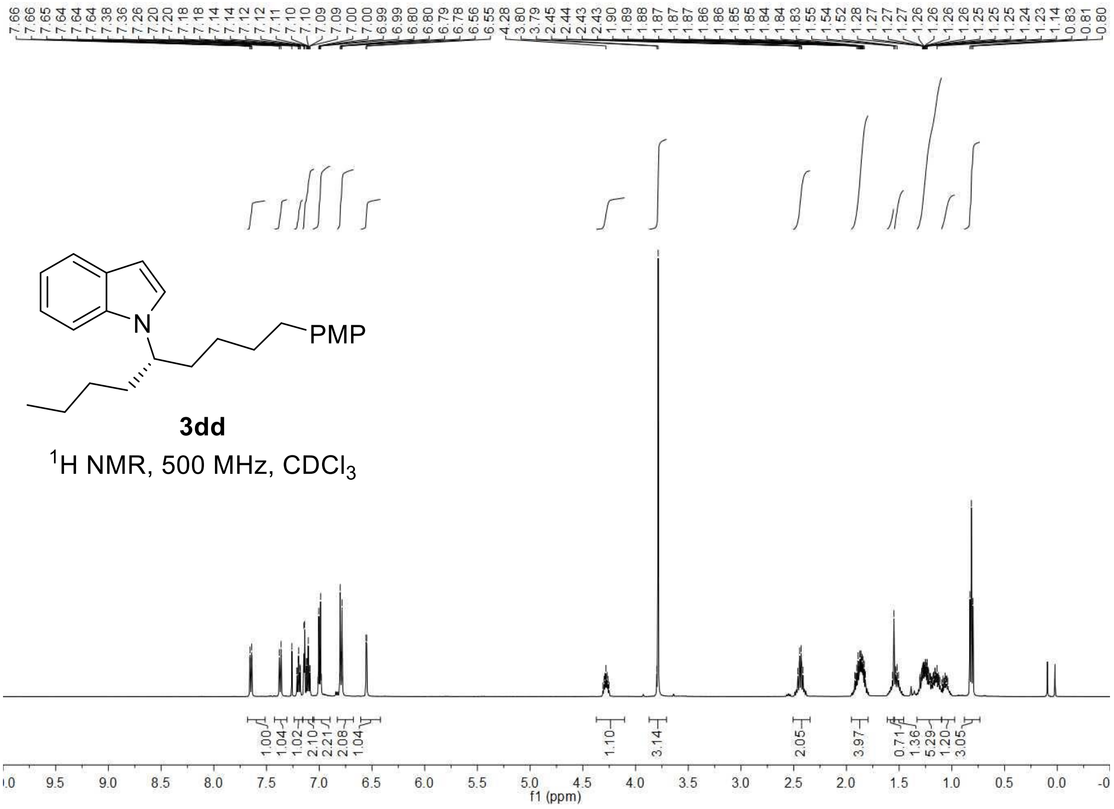

Supplementary Figure 123. ^{1}H NMR spectrum of compound **3dd**

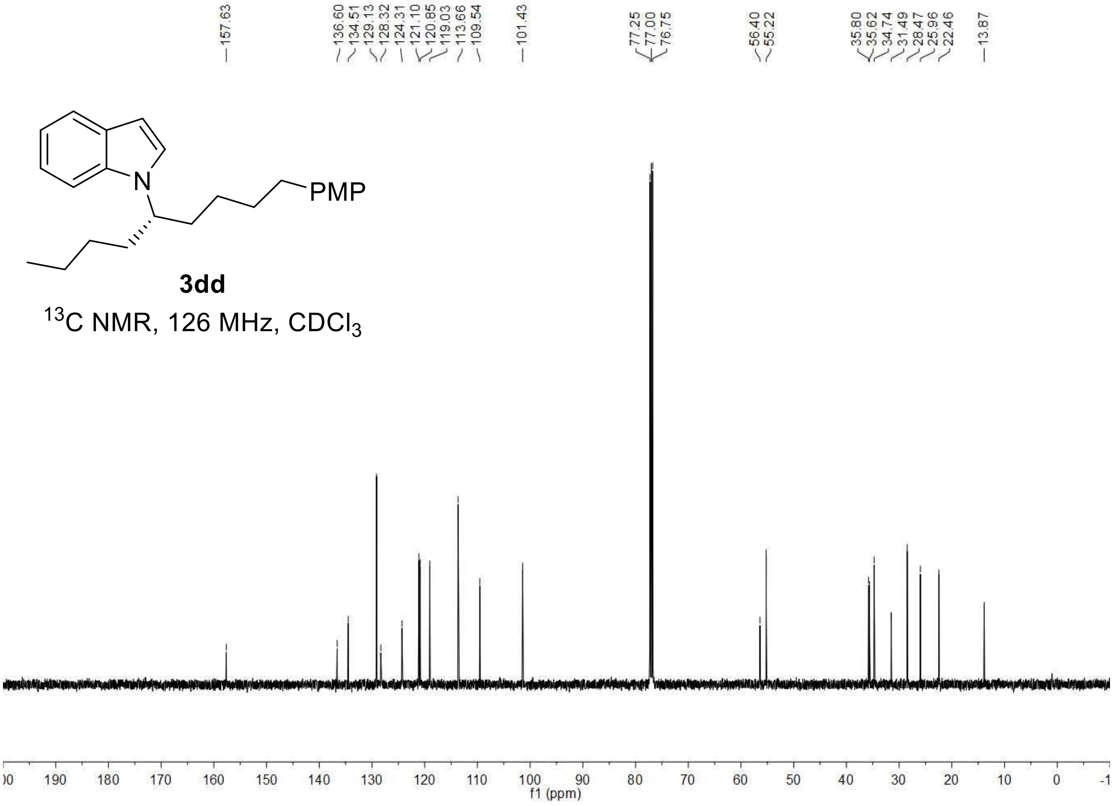

Supplementary Figure 124. ^{13}C NMR spectrum of compound **3dd**

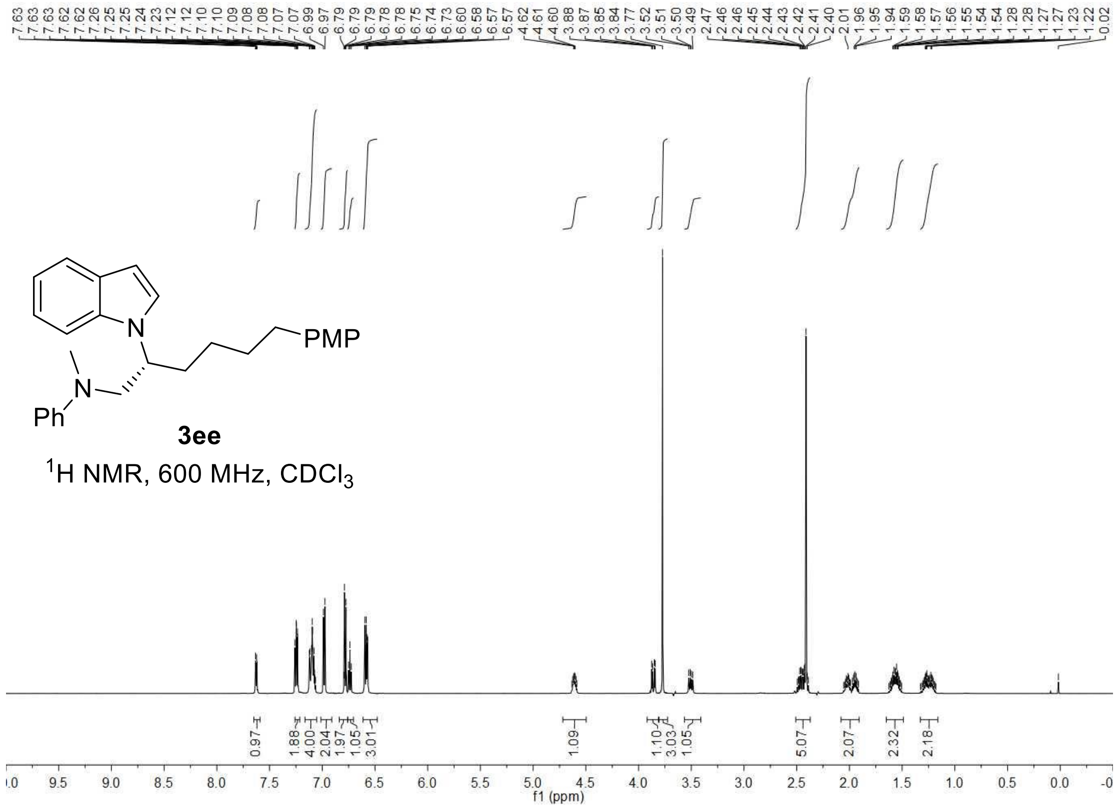

Supplementary Figure 125. ^{1}H NMR spectrum of compound **3ee**

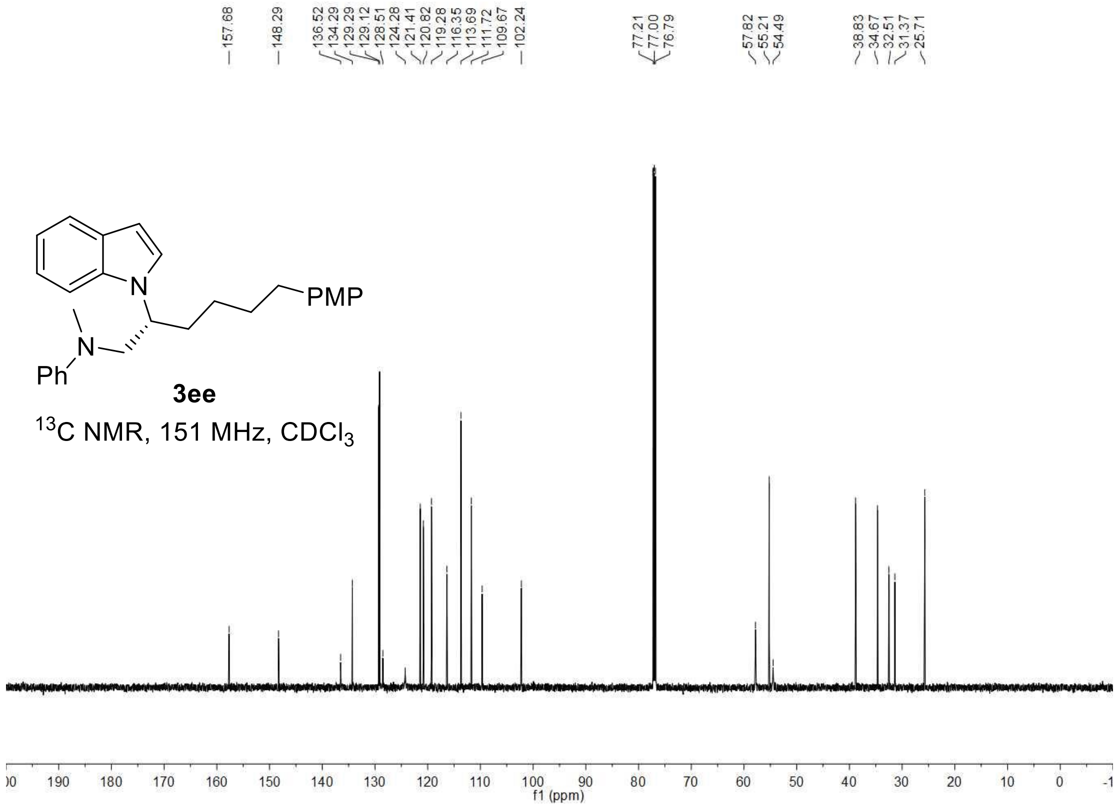

Supplementary Figure 126. ^{13}C NMR spectrum of compound **3ee**

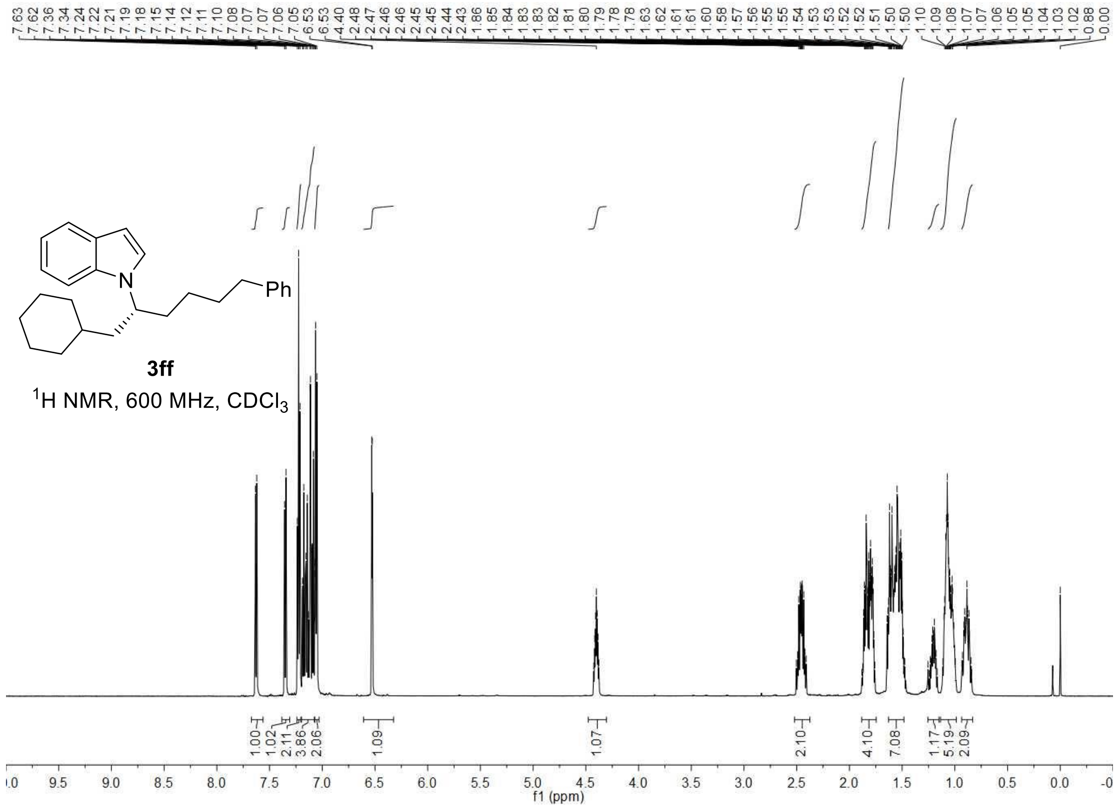

Supplementary Figure 127. ^{1}H NMR spectrum of compound **3ff**

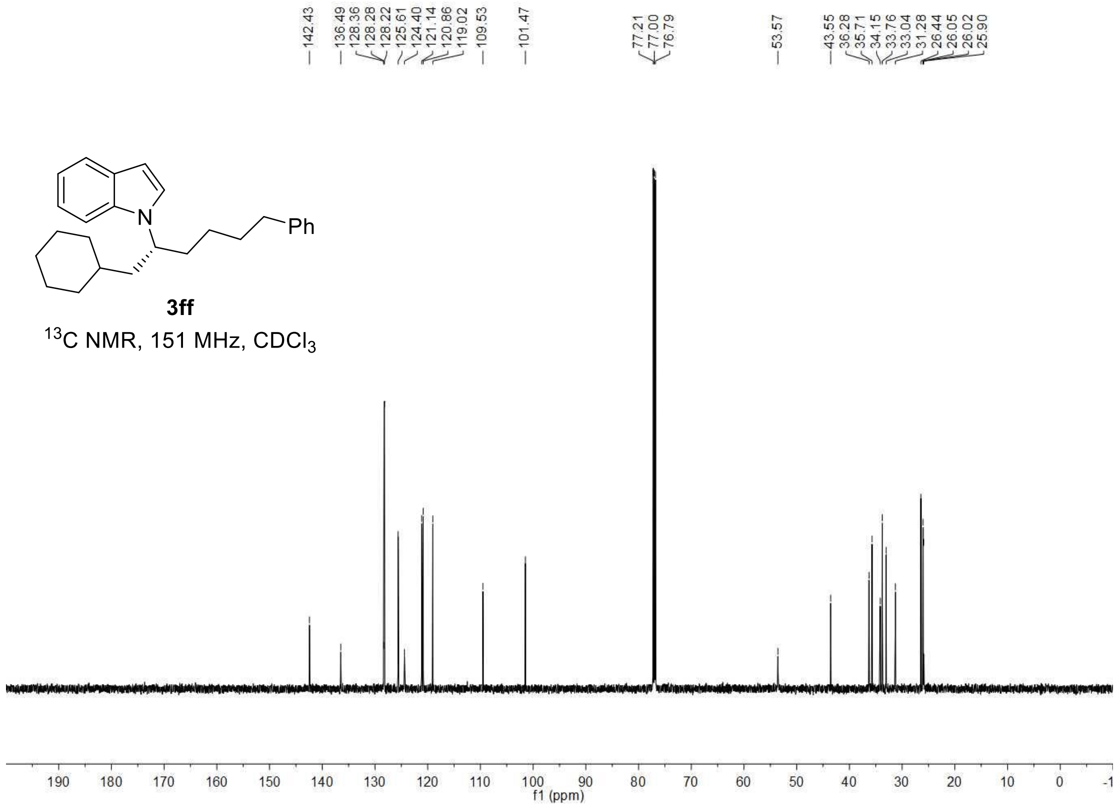

Supplementary Figure 128. ^{13}C NMR spectrum of compound **3ff**

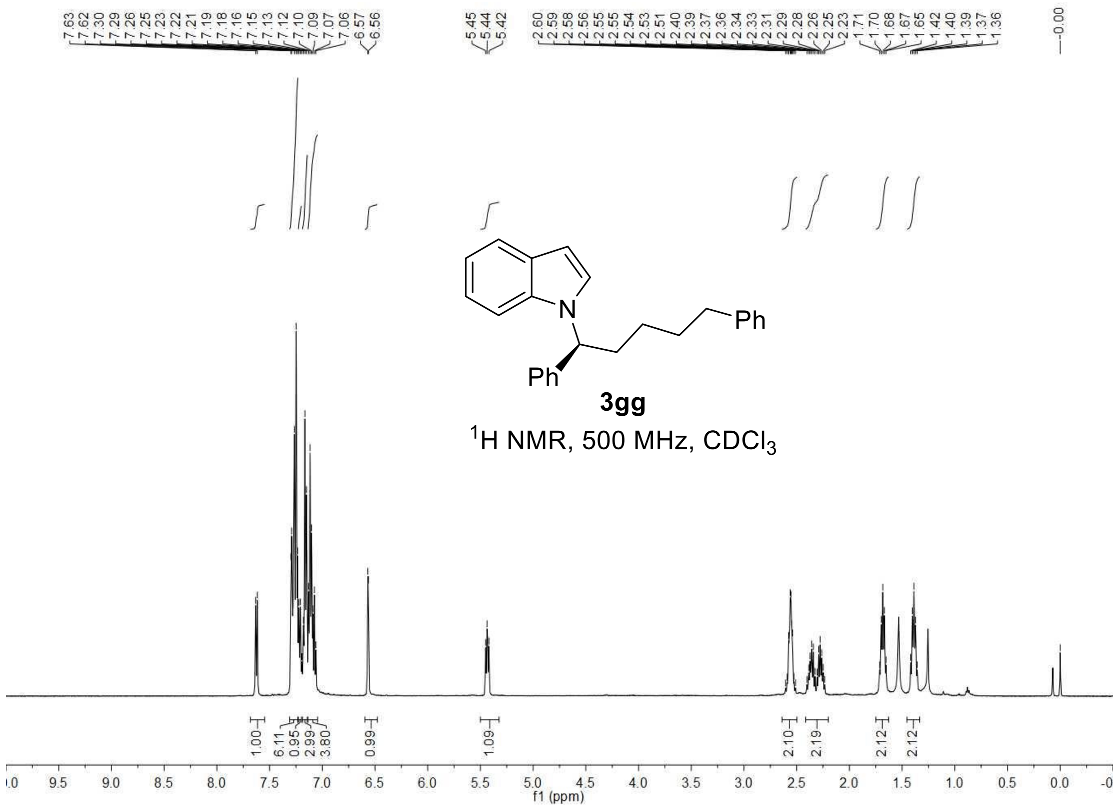

Supplementary Figure 129. ^{1}H NMR spectrum of compound **3gg**

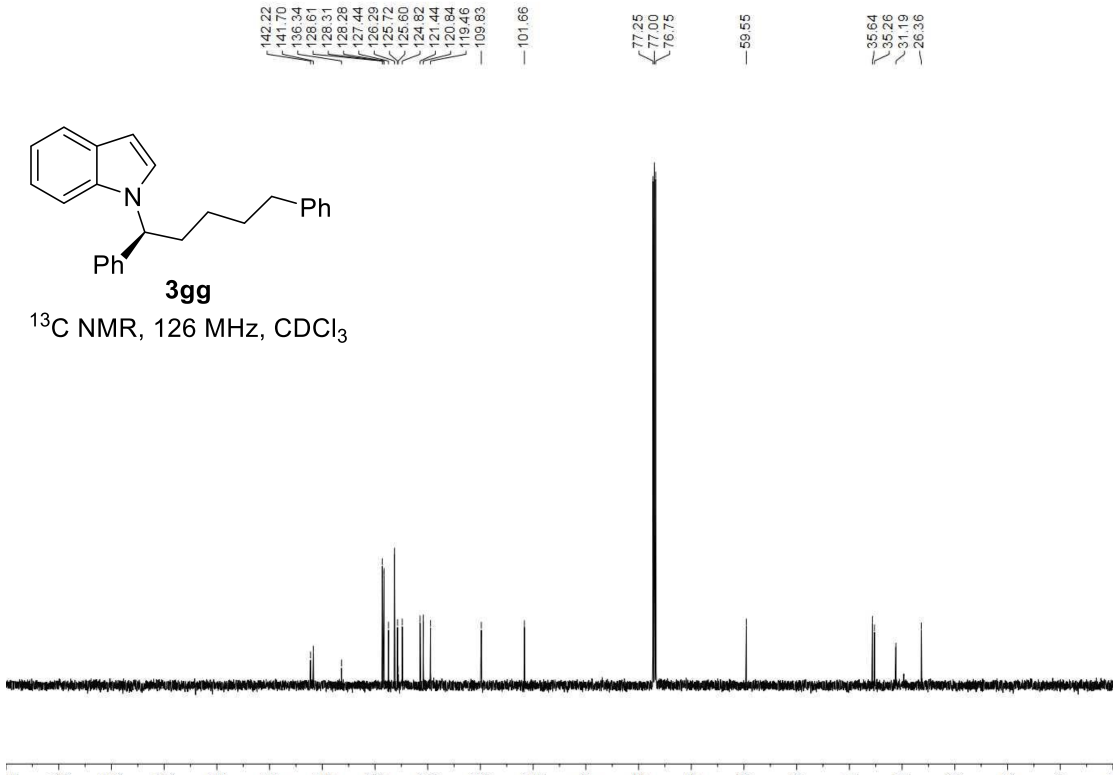

Supplementary Figure 130. ^{13}C NMR spectrum of compound **3gg**

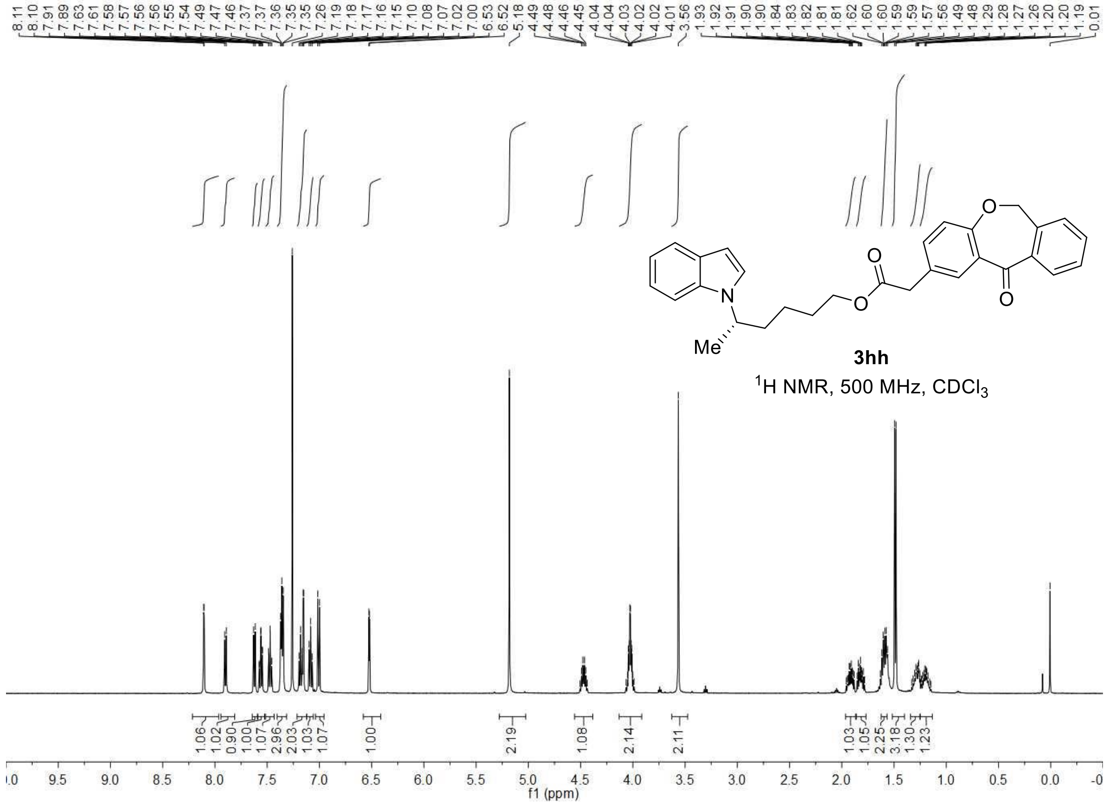

Supplementary Figure 131. ^{1}H NMR spectrum of compound **3hh**

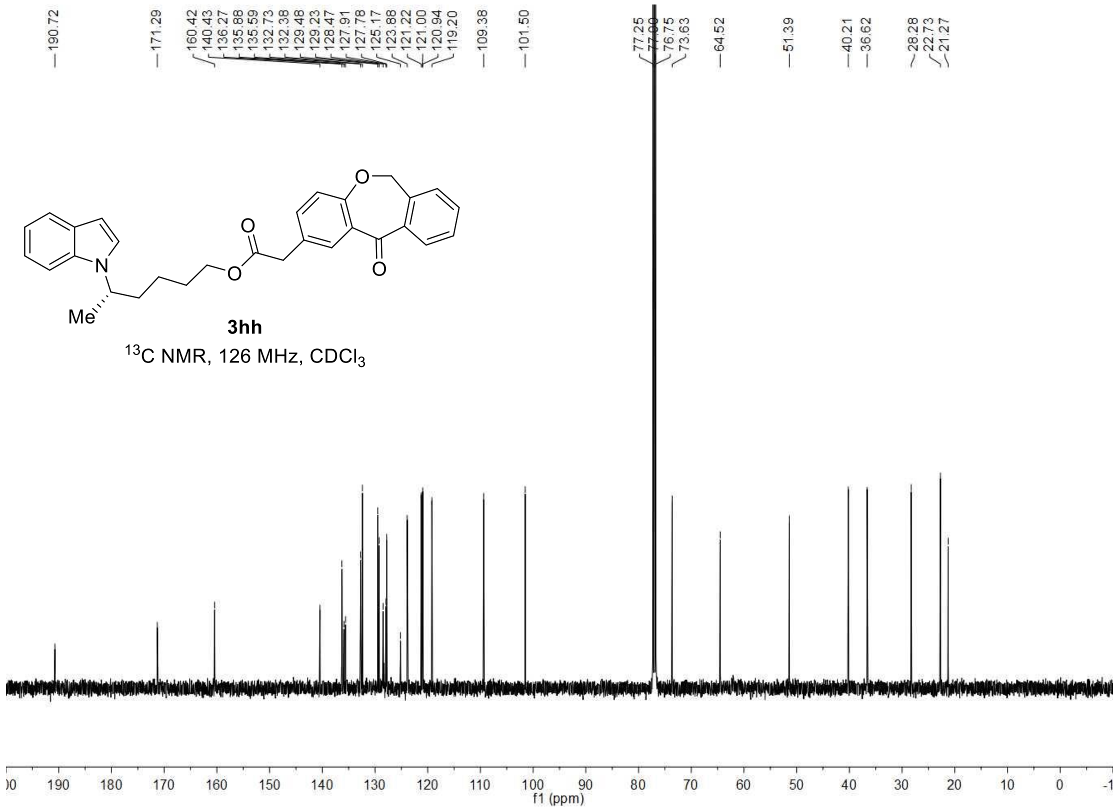

Supplementary Figure 132. ^{13}C NMR spectrum of compound **3hh**

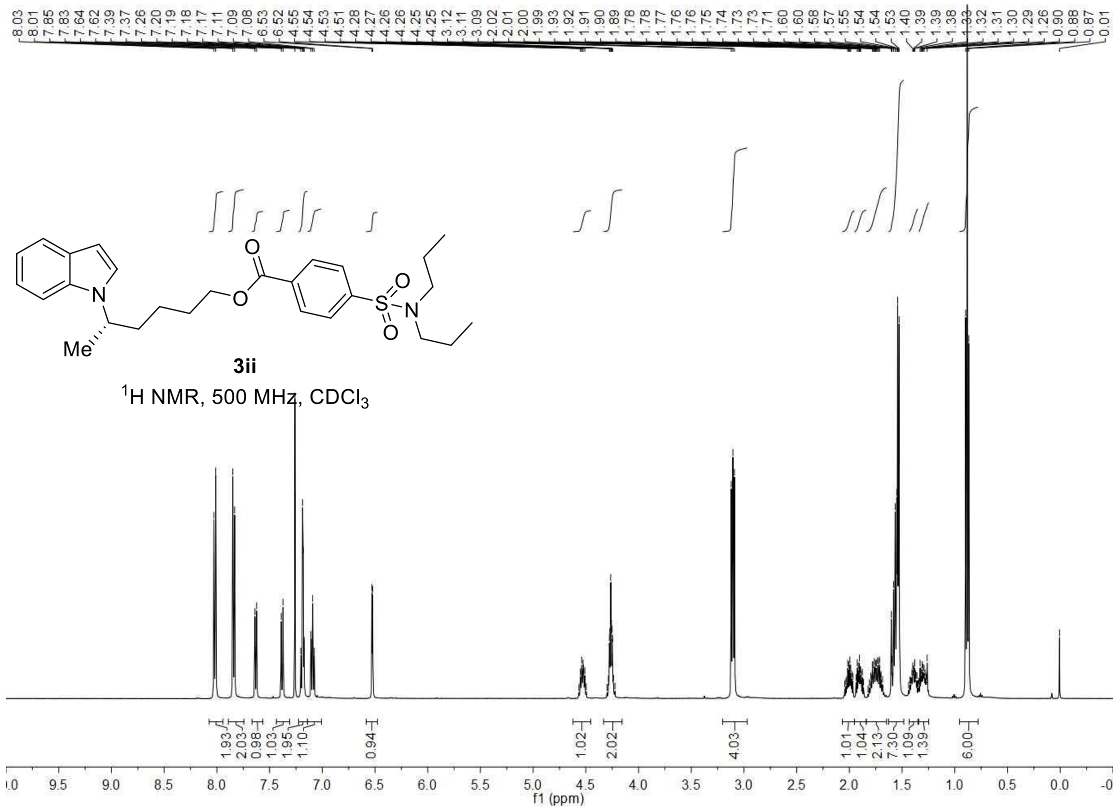

Supplementary Figure 133. ^{1}H NMR spectrum of compound **3ii**

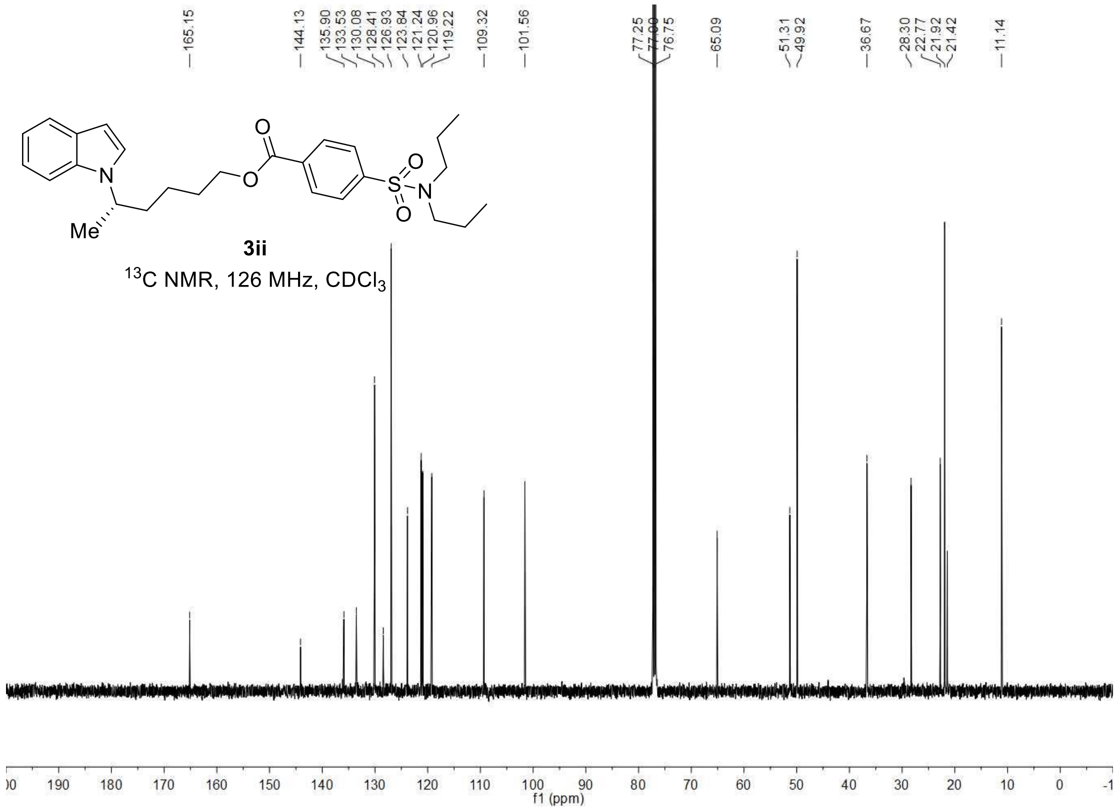

Supplementary Figure 134. ^{13}C NMR spectrum of compound **3ii**

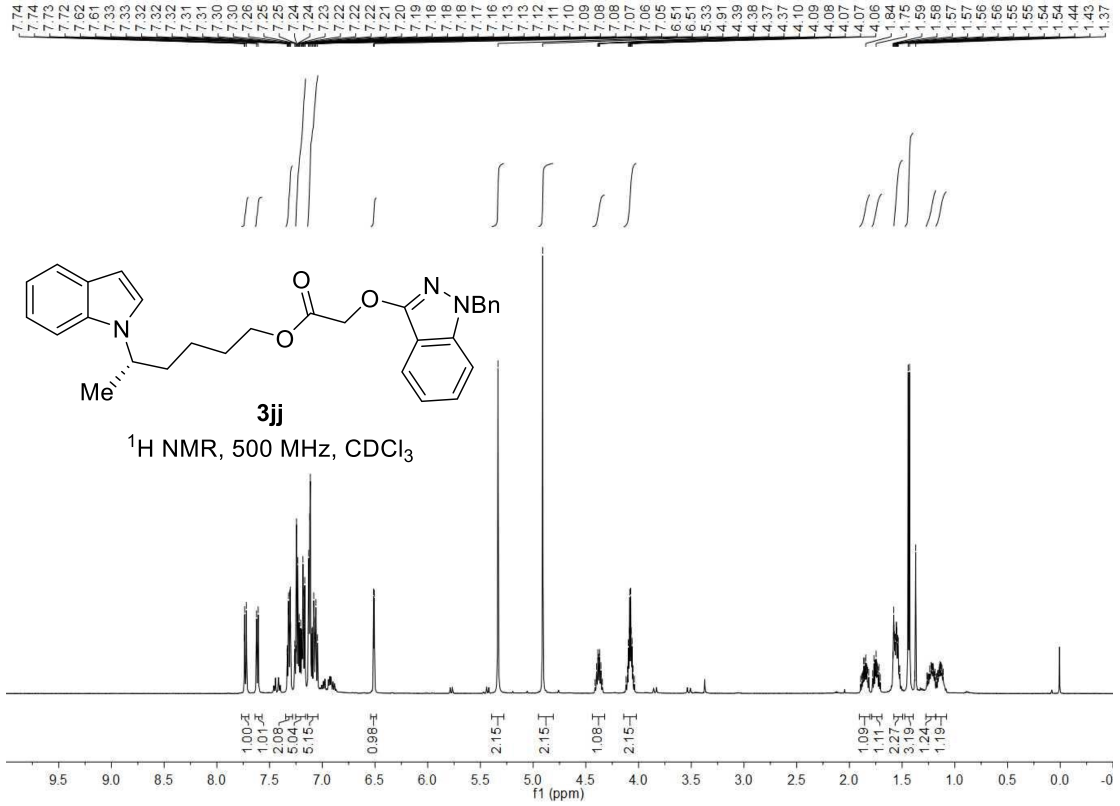

Supplementary Figure 135. ^{1}H NMR spectrum of compound **3jj**

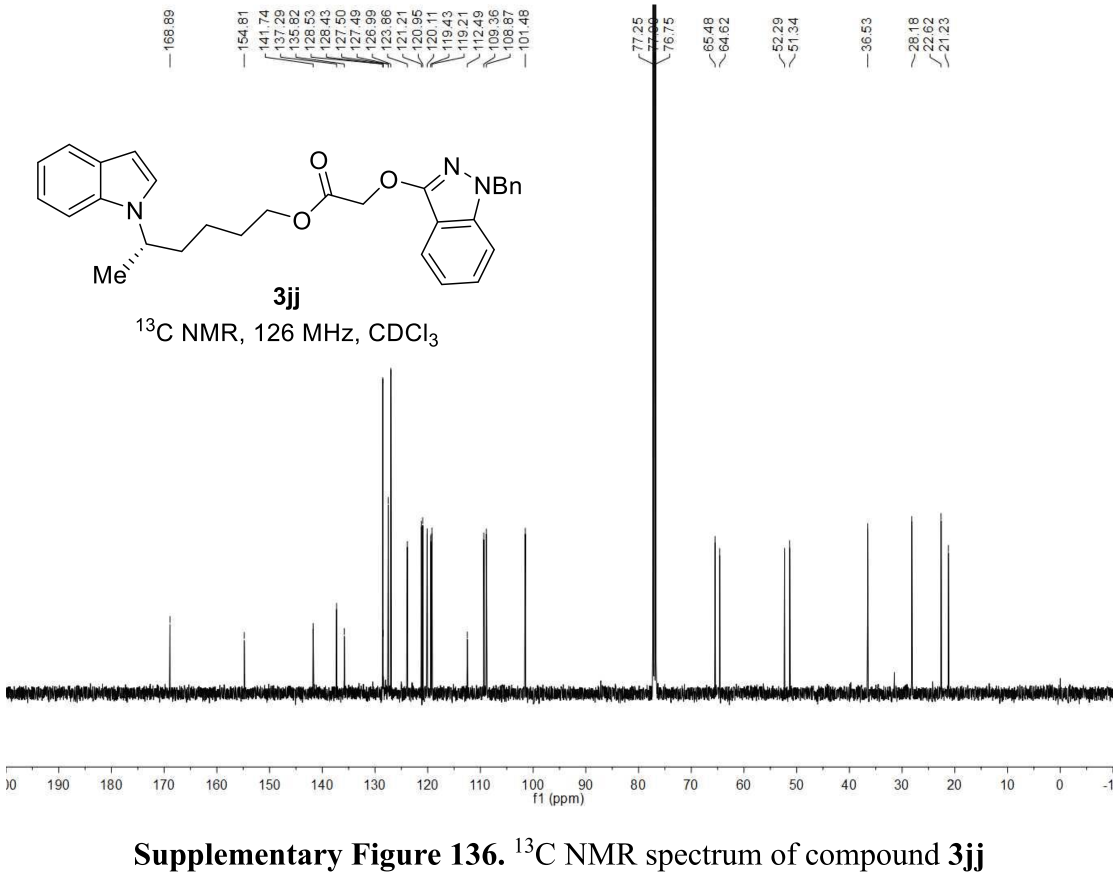

Supplementary Figure 136. ^{13}C NMR spectrum of compound **3jj**

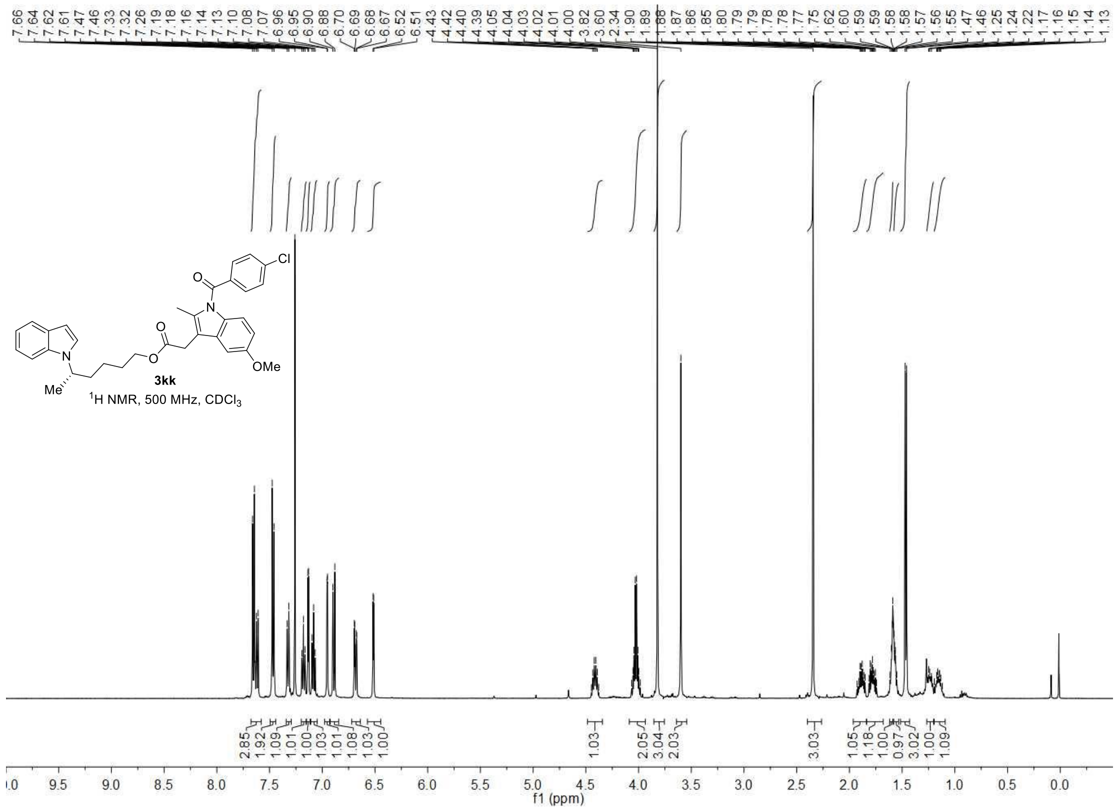

Supplementary Figure 137. ^{1}H NMR spectrum of compound **3kk**

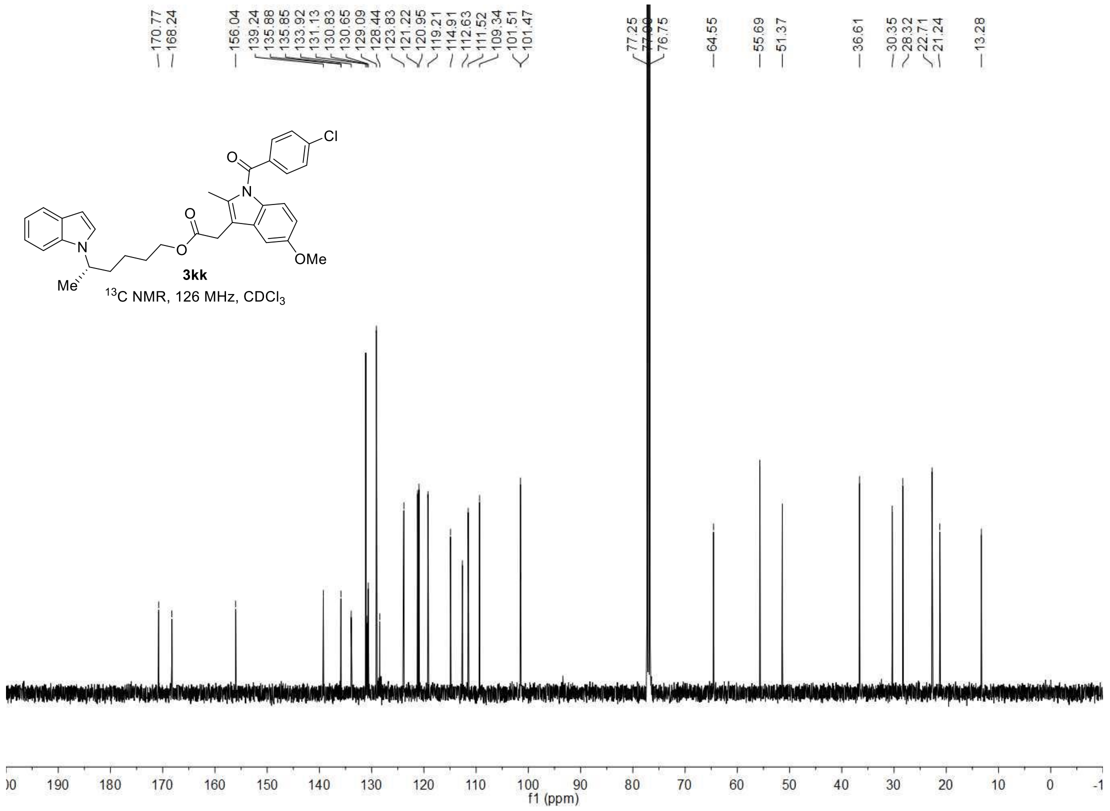

Supplementary Figure 138. ^{13}C NMR spectrum of compound **3kk**

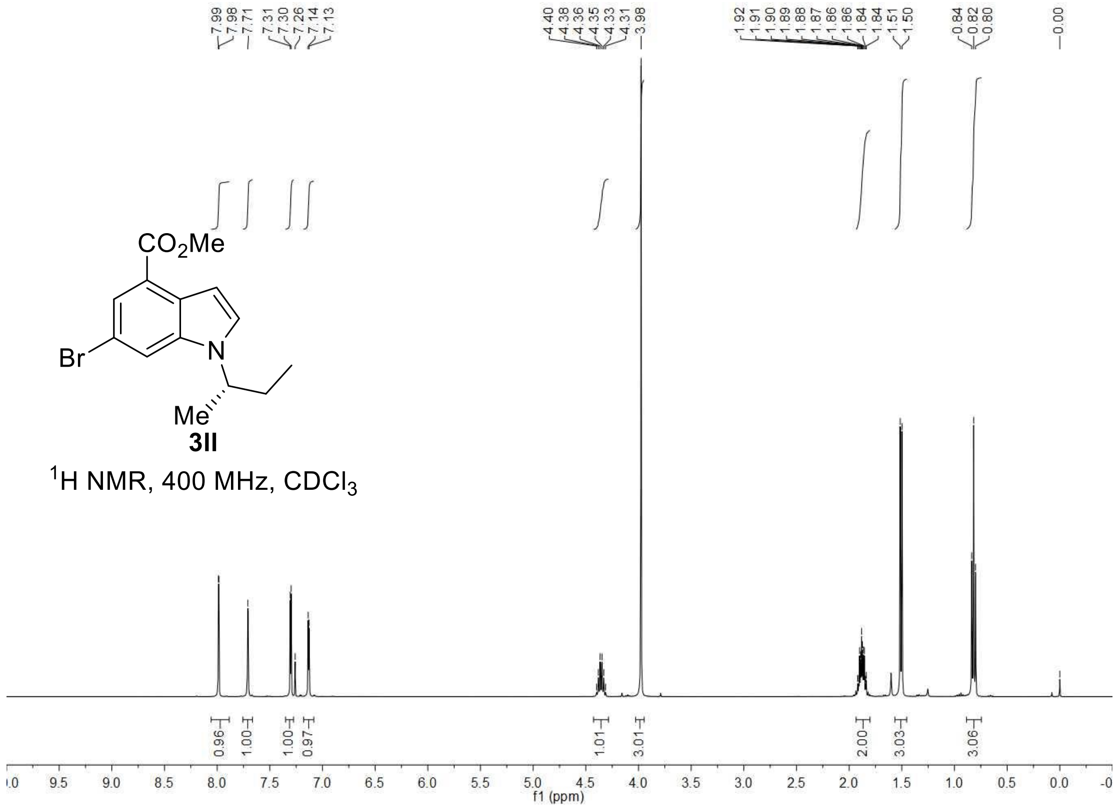

Supplementary Figure 139. ^{1}H NMR spectrum of compound **3ll**

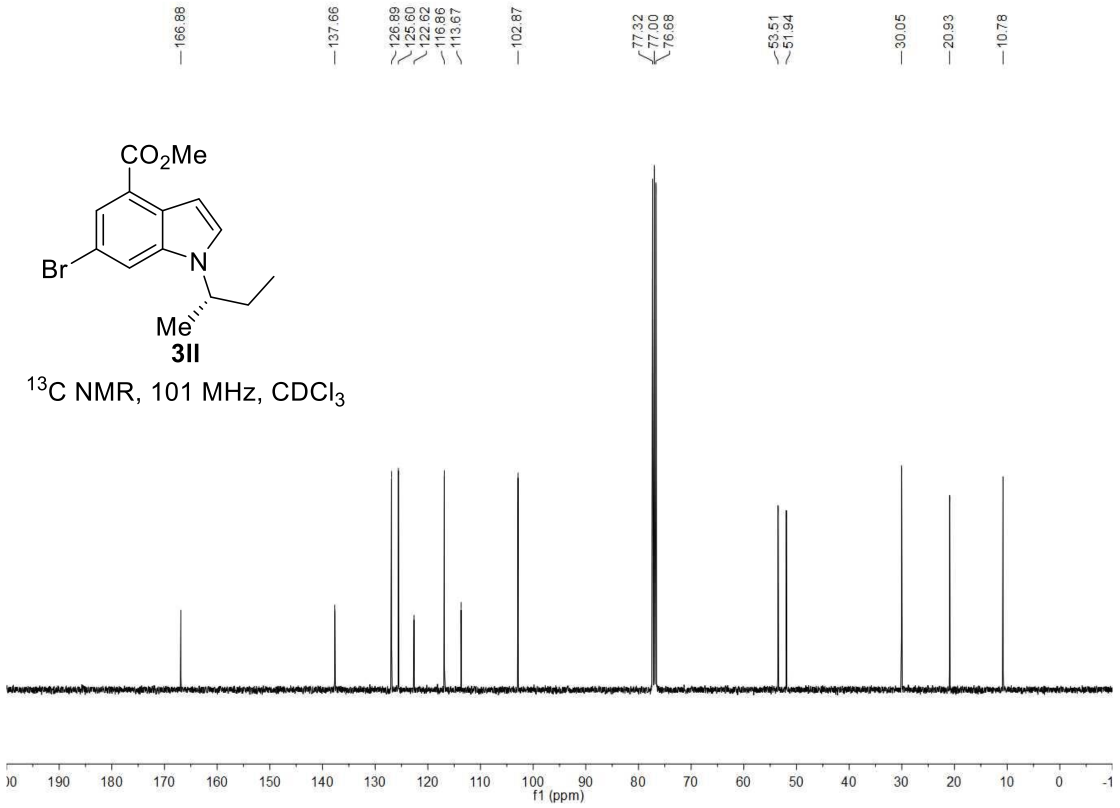

Supplementary Figure 140. ^{13}C NMR spectrum of compound **3ll**

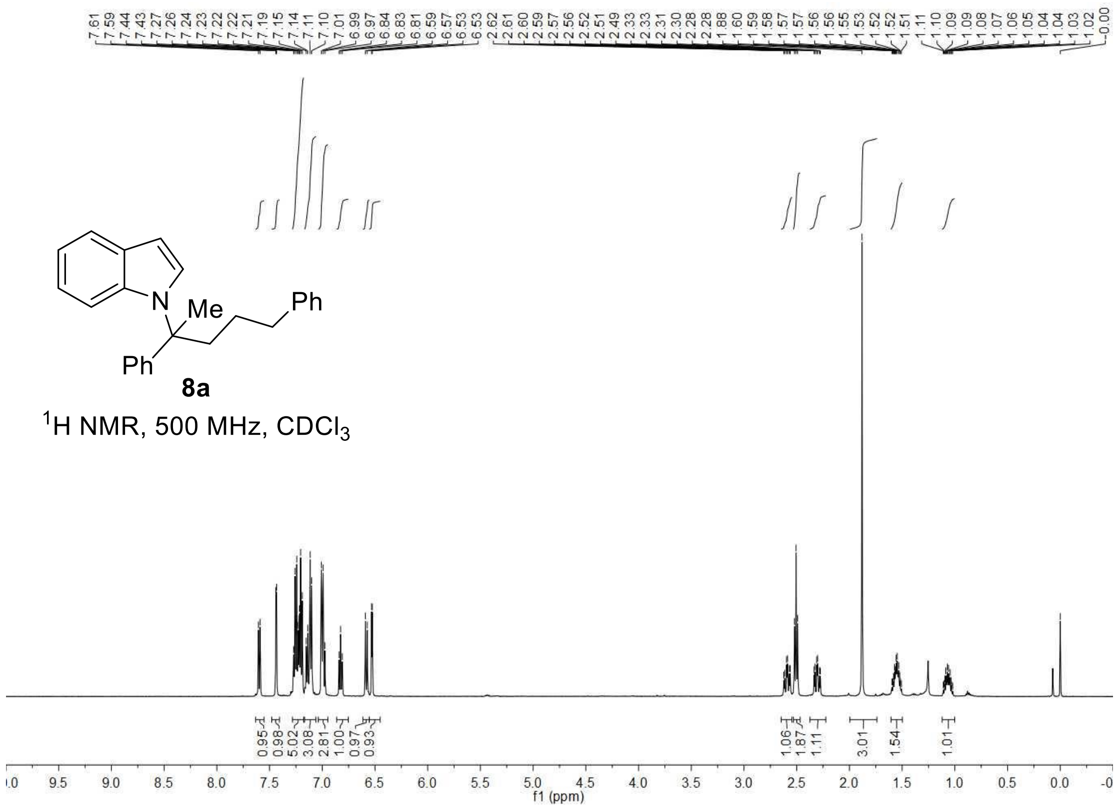

Supplementary Figure 141. ^{1}H NMR spectrum of compound **8a**

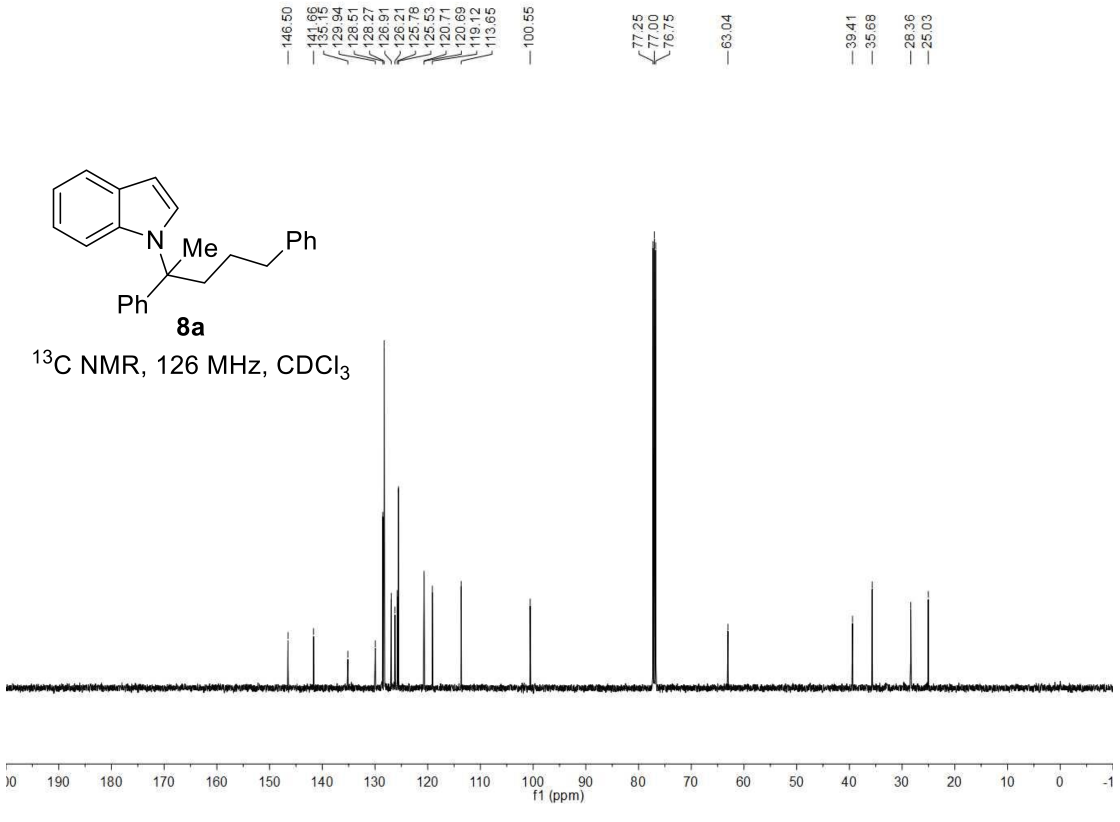

Supplementary Figure 142. ^{13}C NMR spectrum of compound **8a**

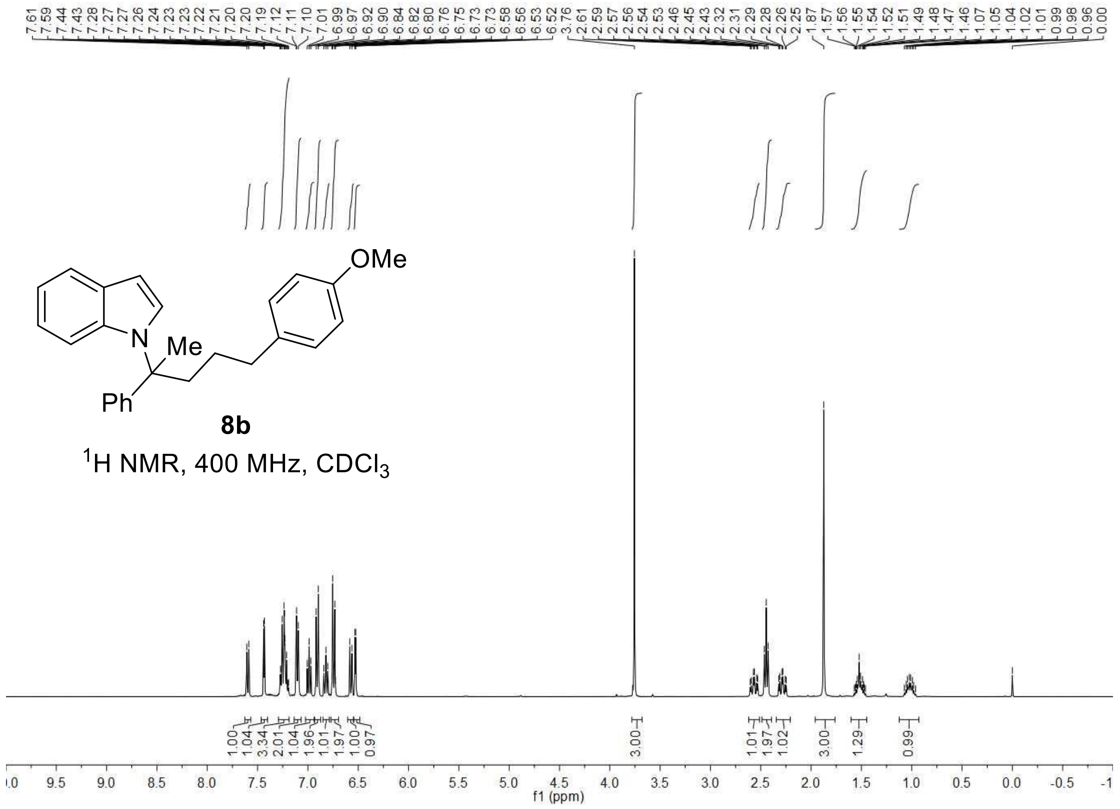

Supplementary Figure 143. ^{1}H NMR spectrum of compound **8b**

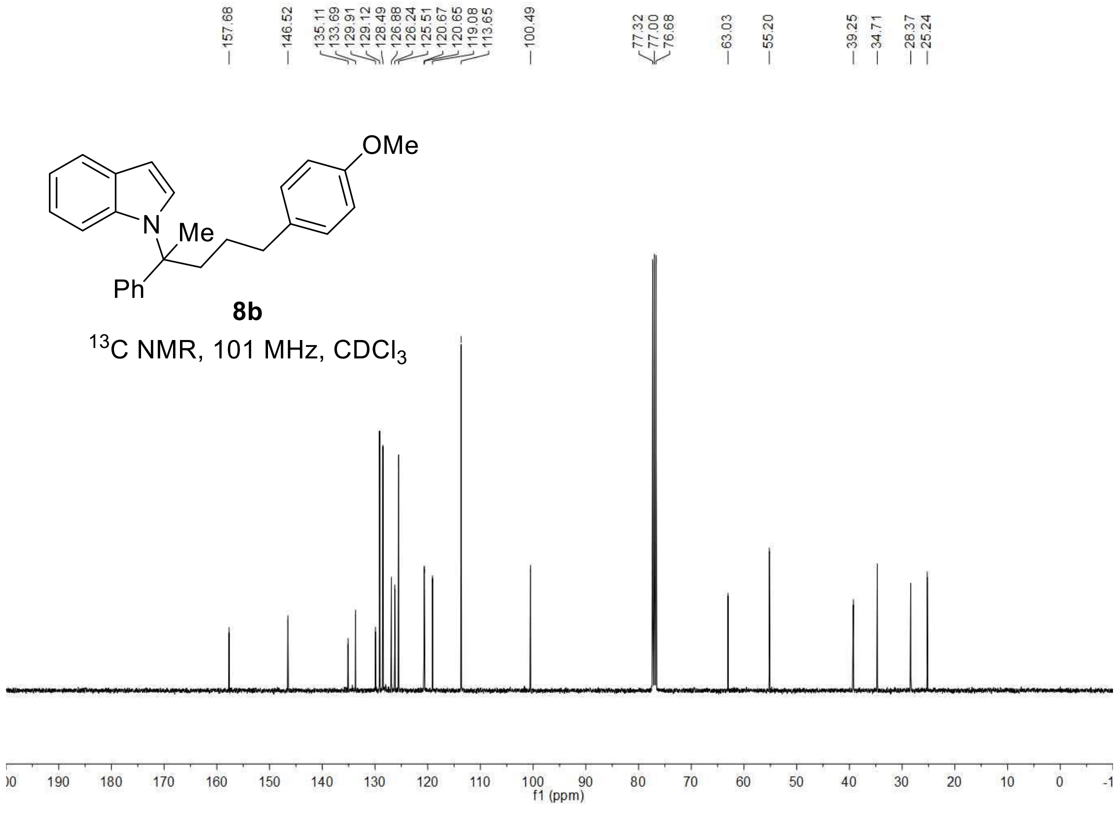

Supplementary Figure 144. ^{13}C NMR spectrum of compound **8b**

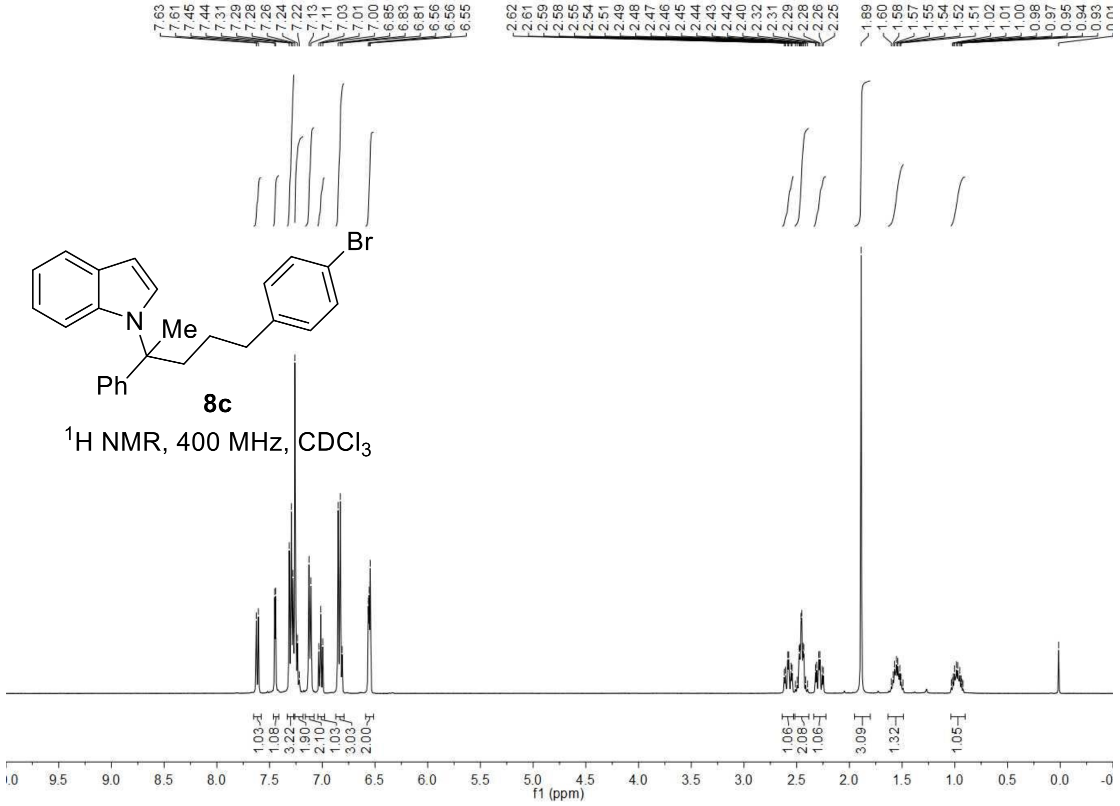

Supplementary Figure 145. ^{1}H NMR spectrum of compound **8c**

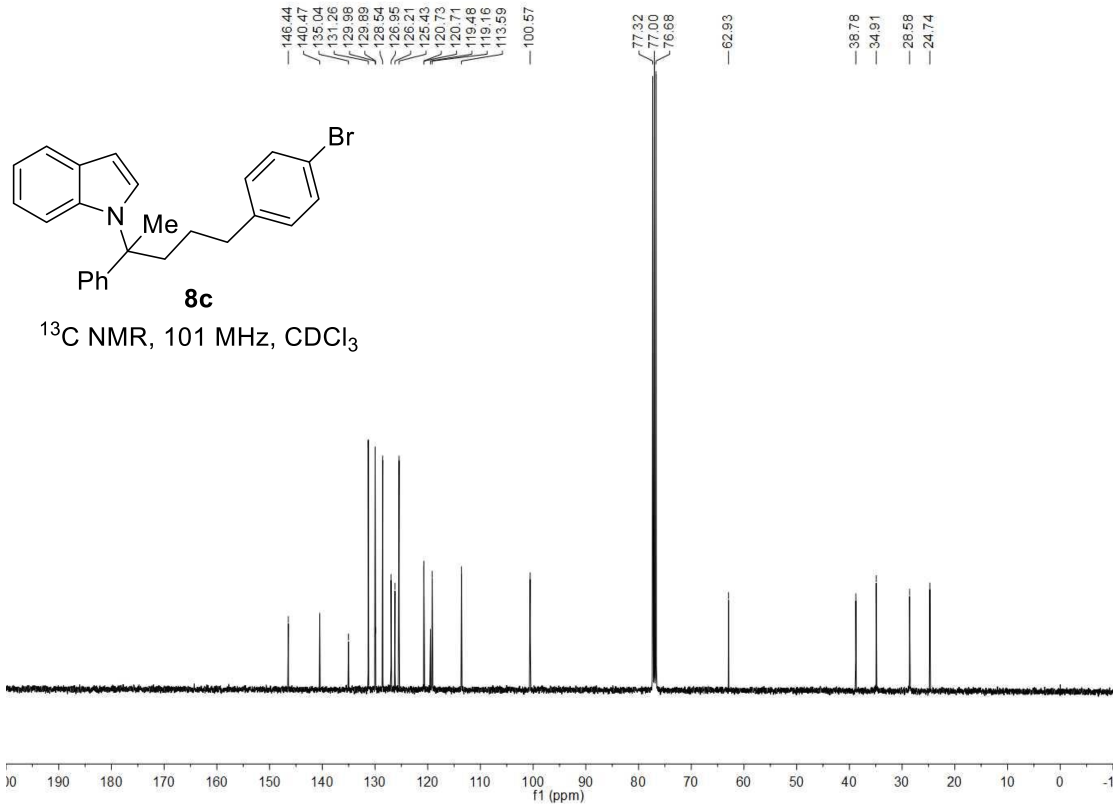

Supplementary Figure 146. ^{13}C NMR spectrum of compound **8c**

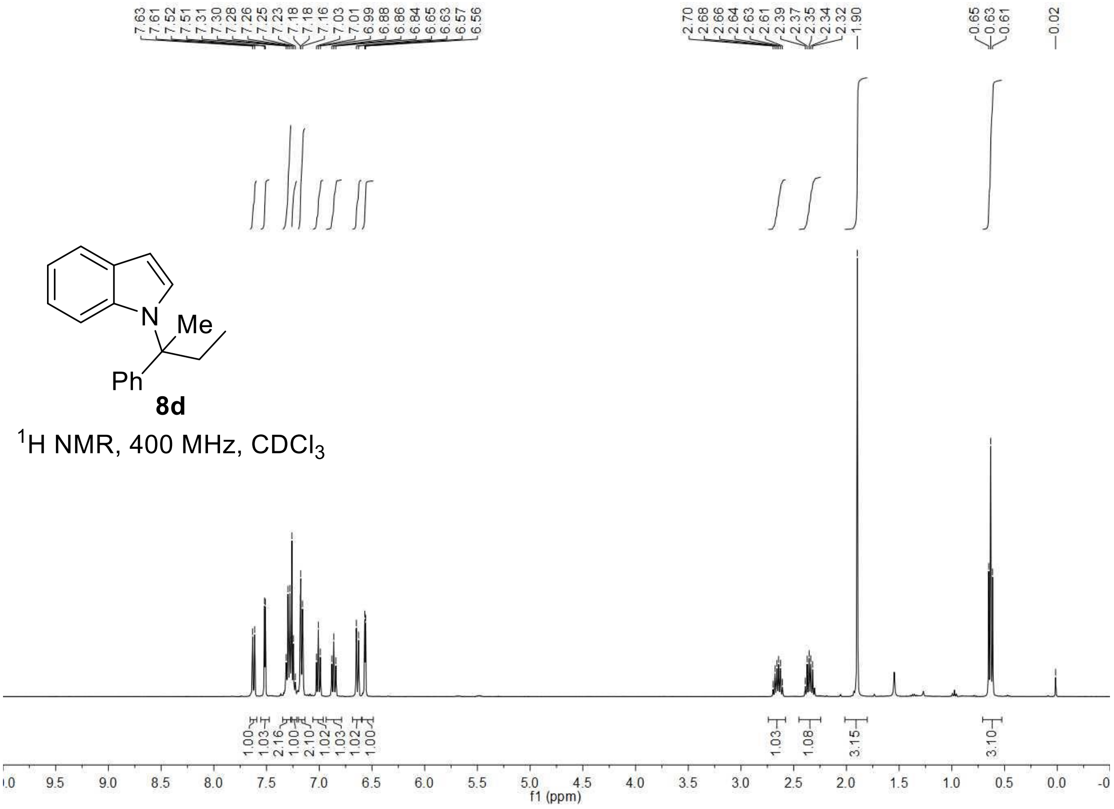

Supplementary Figure 147. ^{1}H NMR spectrum of compound **8d**

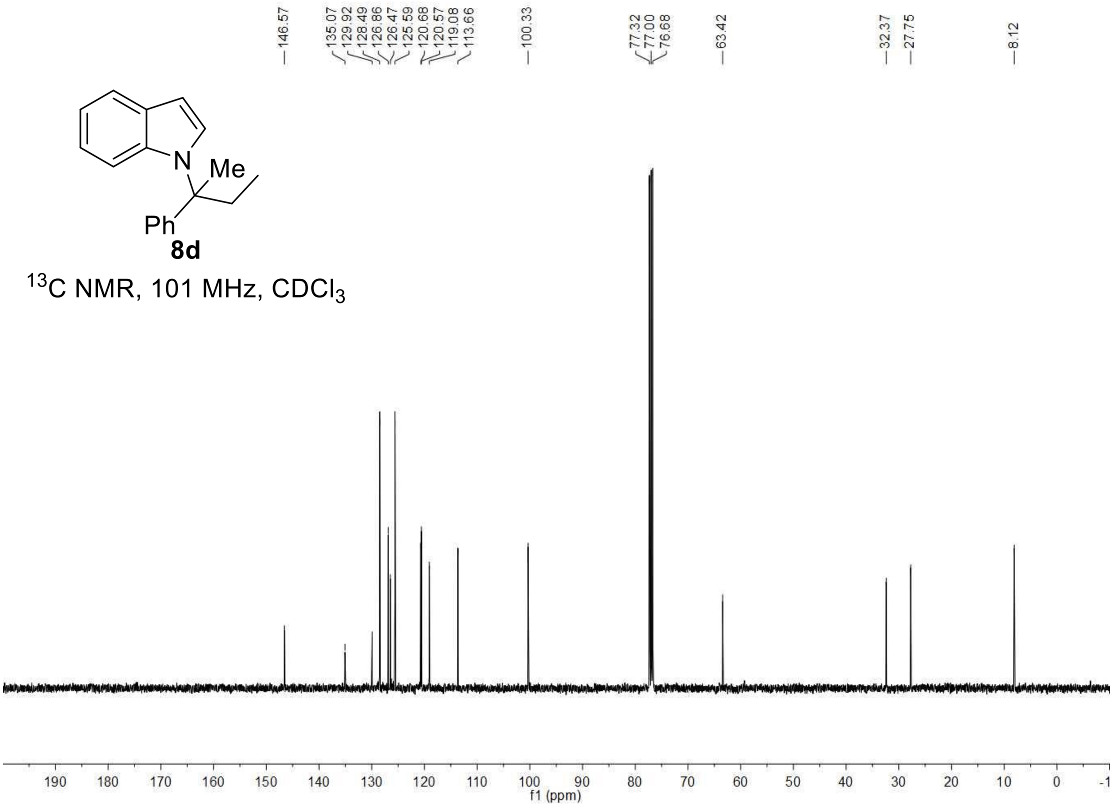

Supplementary Figure 148. ^{13}C NMR spectrum of compound **8d**

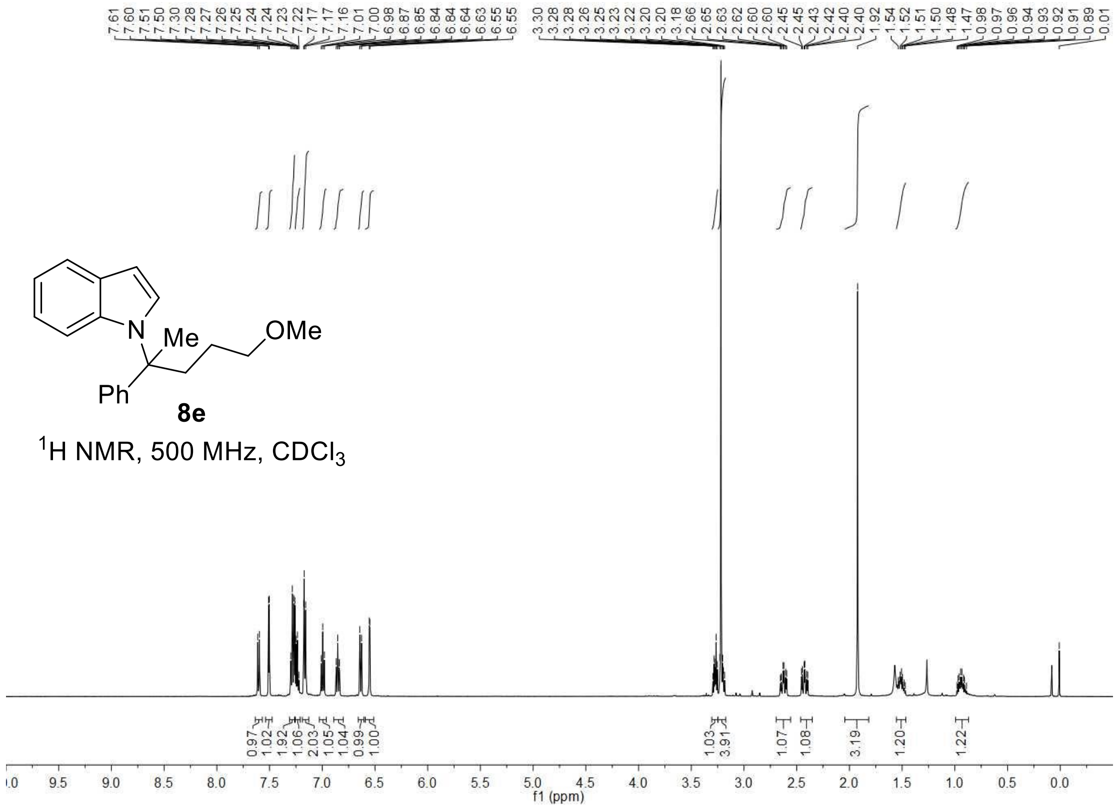

Supplementary Figure 149. ^{1}H NMR spectrum of compound **8e**

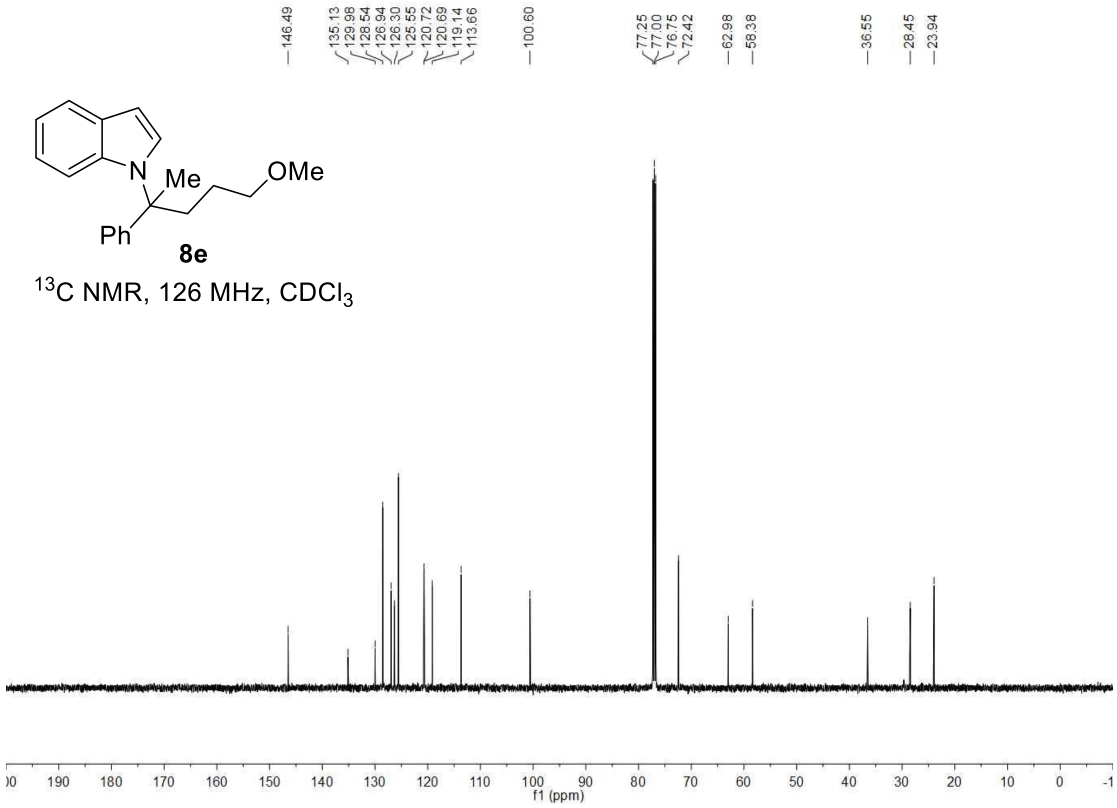

Supplementary Figure 150. ^{13}C NMR spectrum of compound **8e**

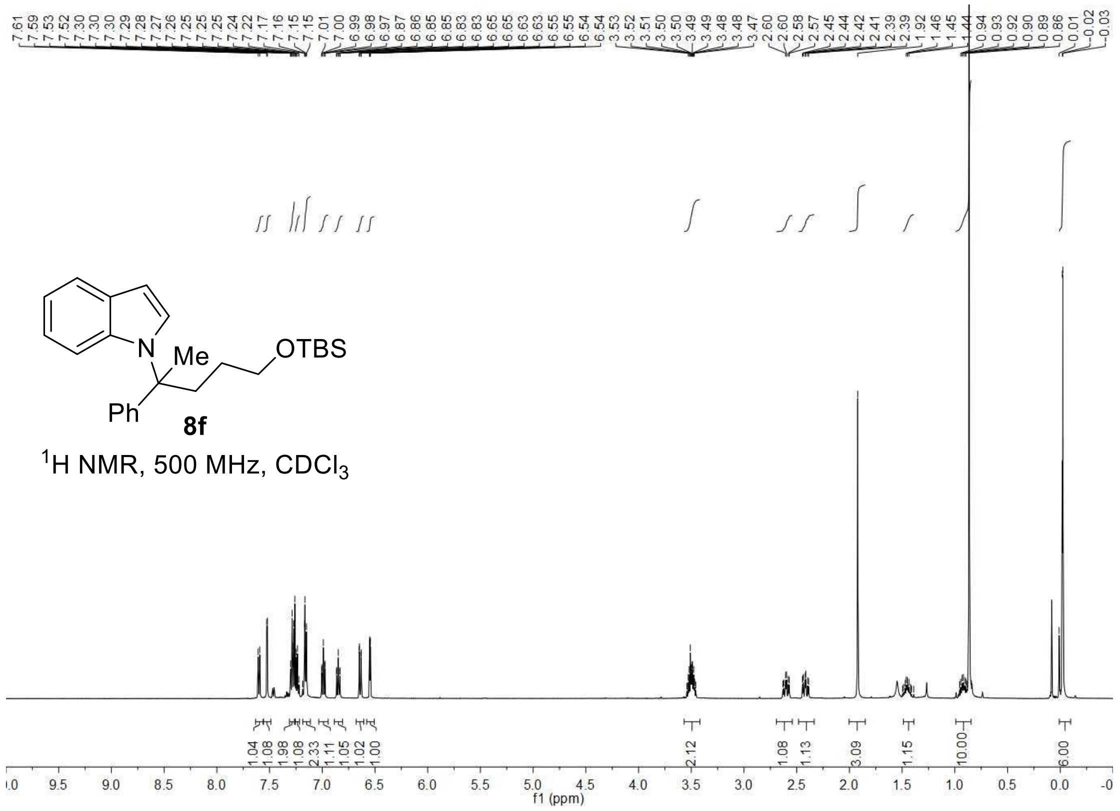

Supplementary Figure 151. ^{1}H NMR spectrum of compound **8f**

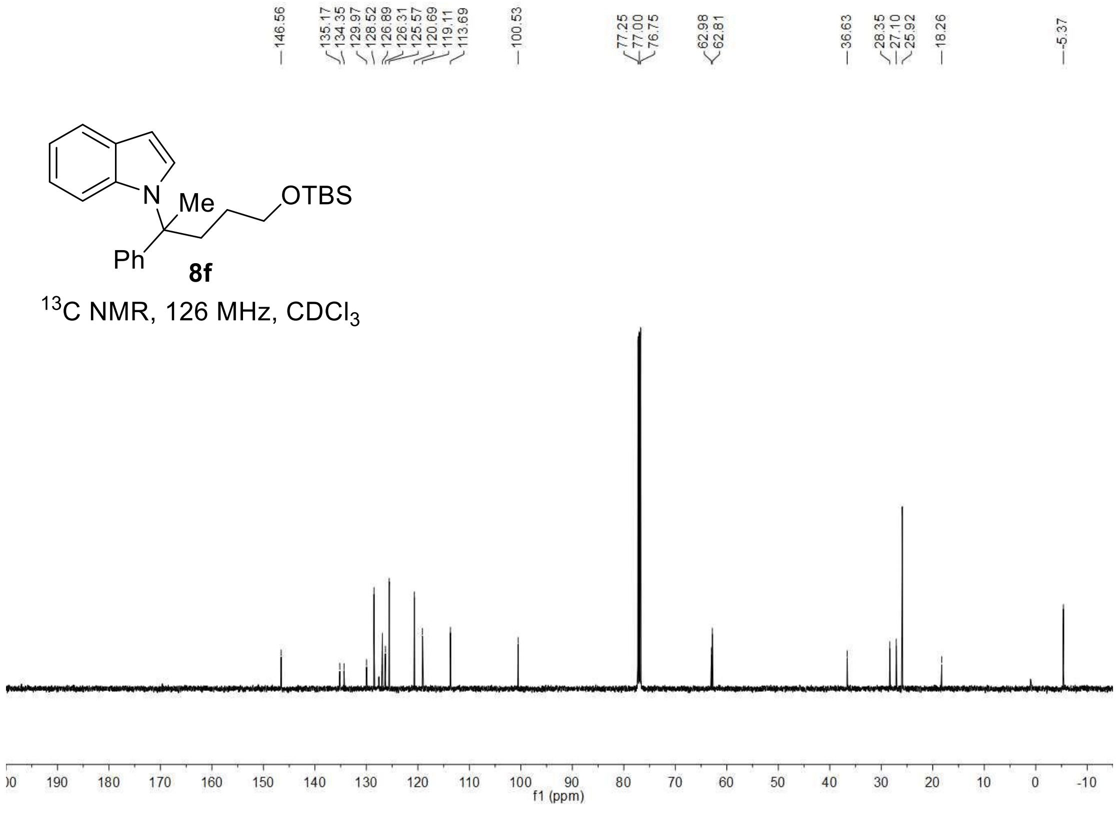

Supplementary Figure 152. ^{13}C NMR spectrum of compound **8f**

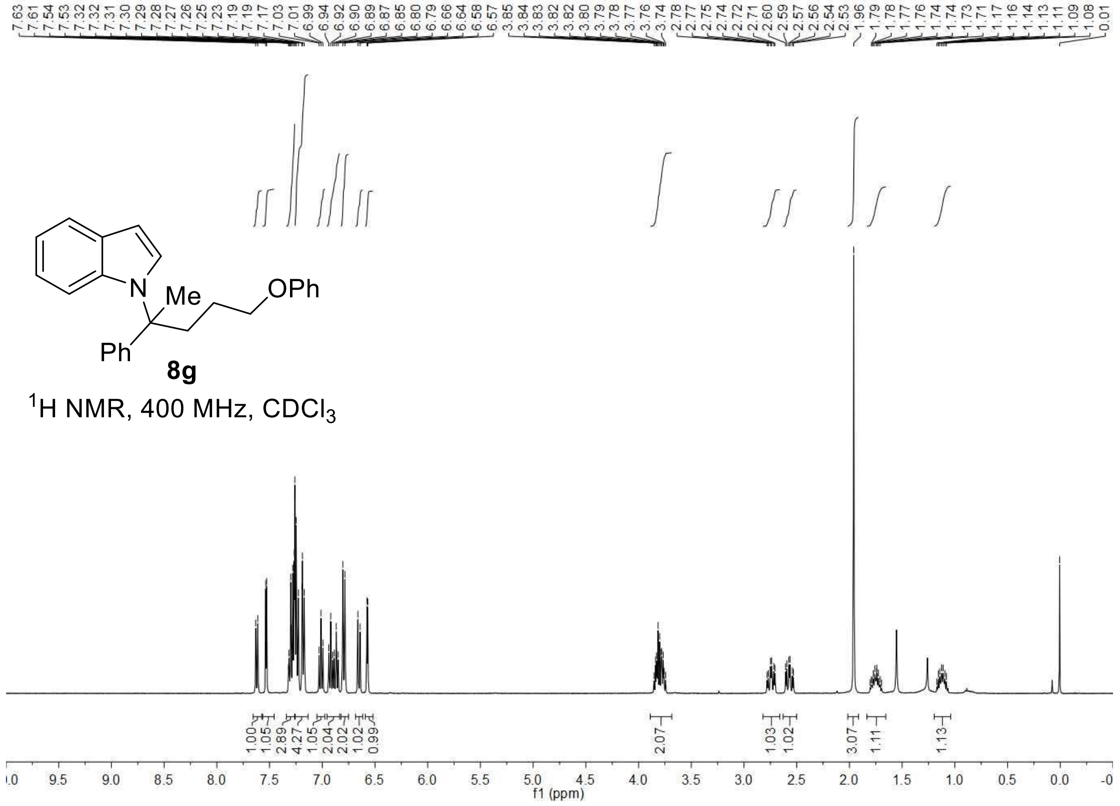

Supplementary Figure 153. ^{1}H NMR spectrum of compound **8g**

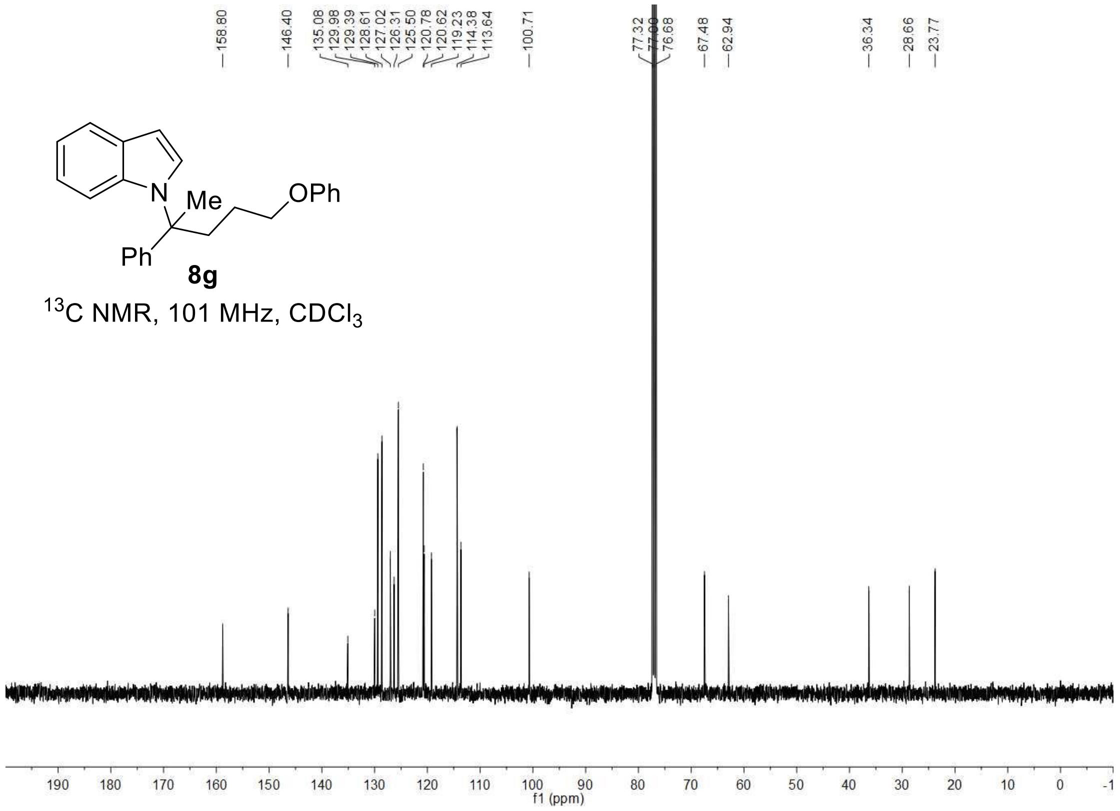

Supplementary Figure 154. ^{13}C NMR spectrum of compound **8g**

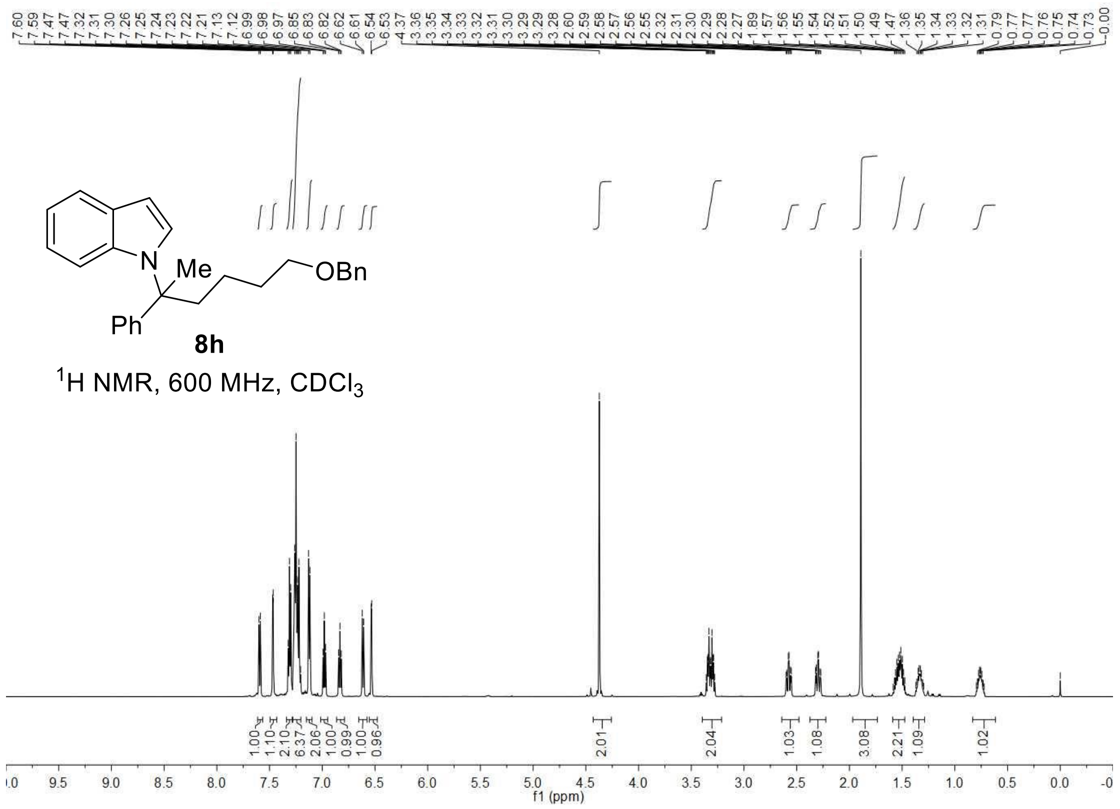

Supplementary Figure 155. ^{1}H NMR spectrum of compound **8h**

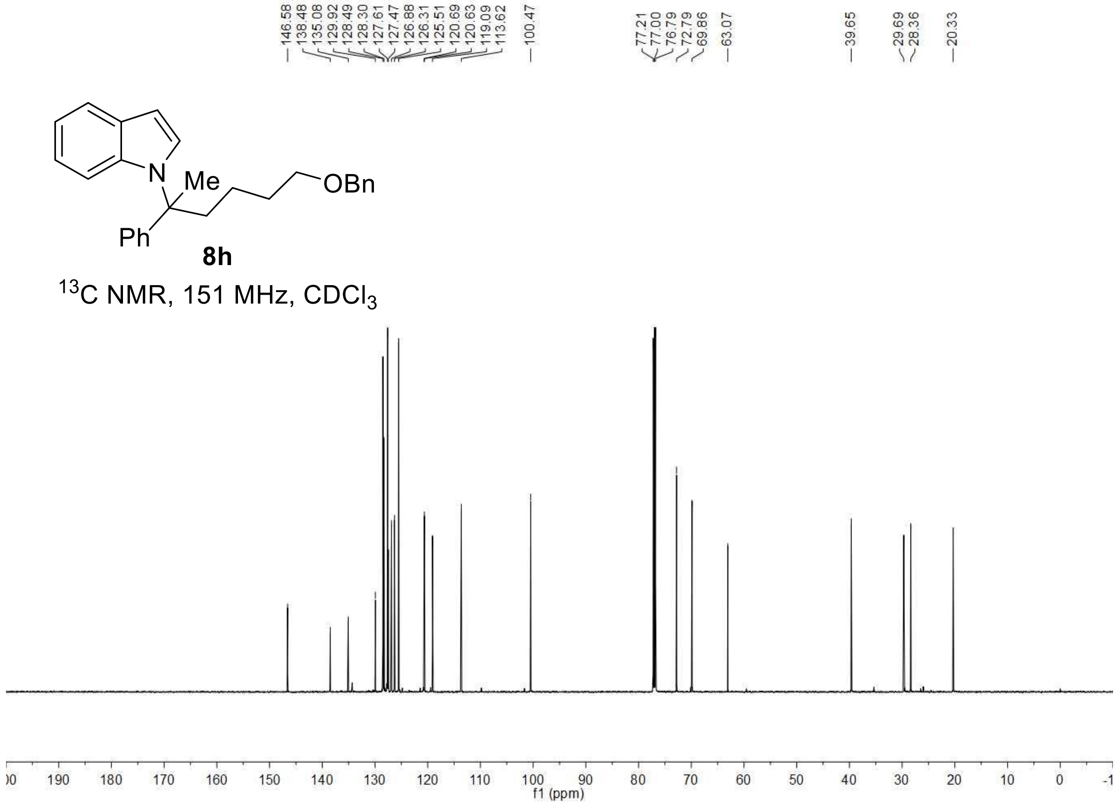

Supplementary Figure 156. ^{13}C NMR spectrum of compound **8h**

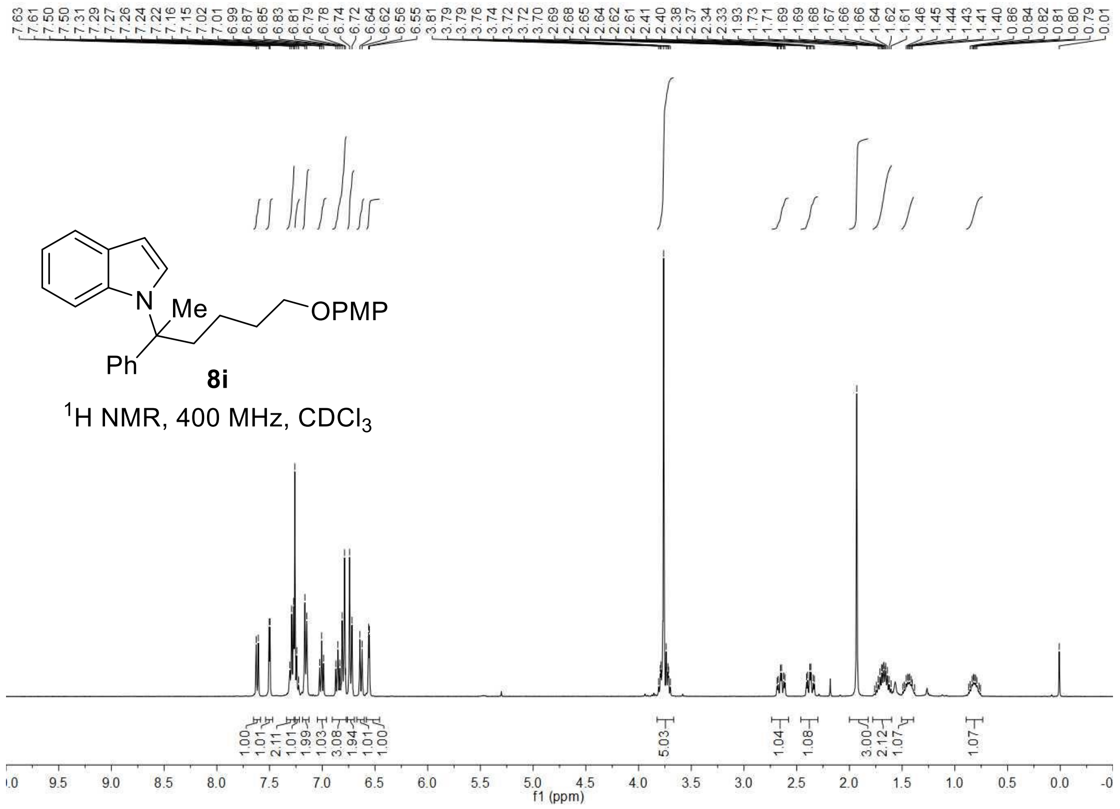

Supplementary Figure 157. ^{1}H NMR spectrum of compound **8i**

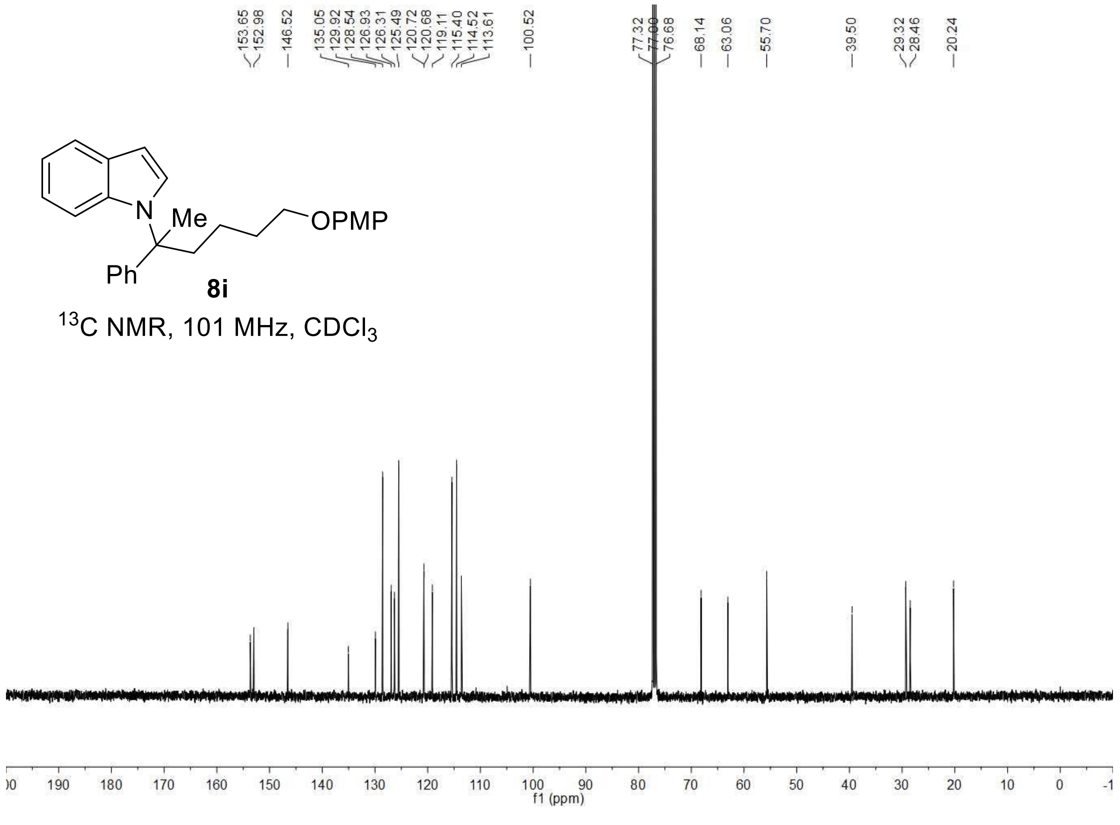

Supplementary Figure 158. ^{13}C NMR spectrum of compound **8i**

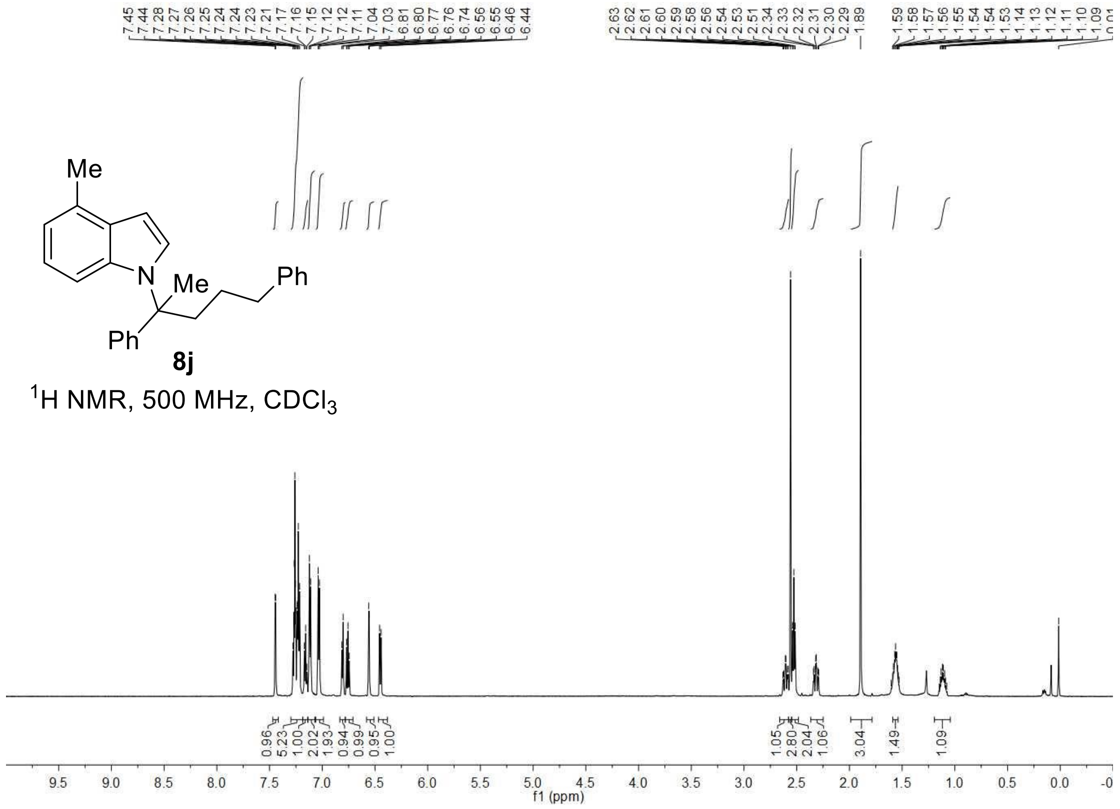

Supplementary Figure 159. ^{1}H NMR spectrum of compound **8j**

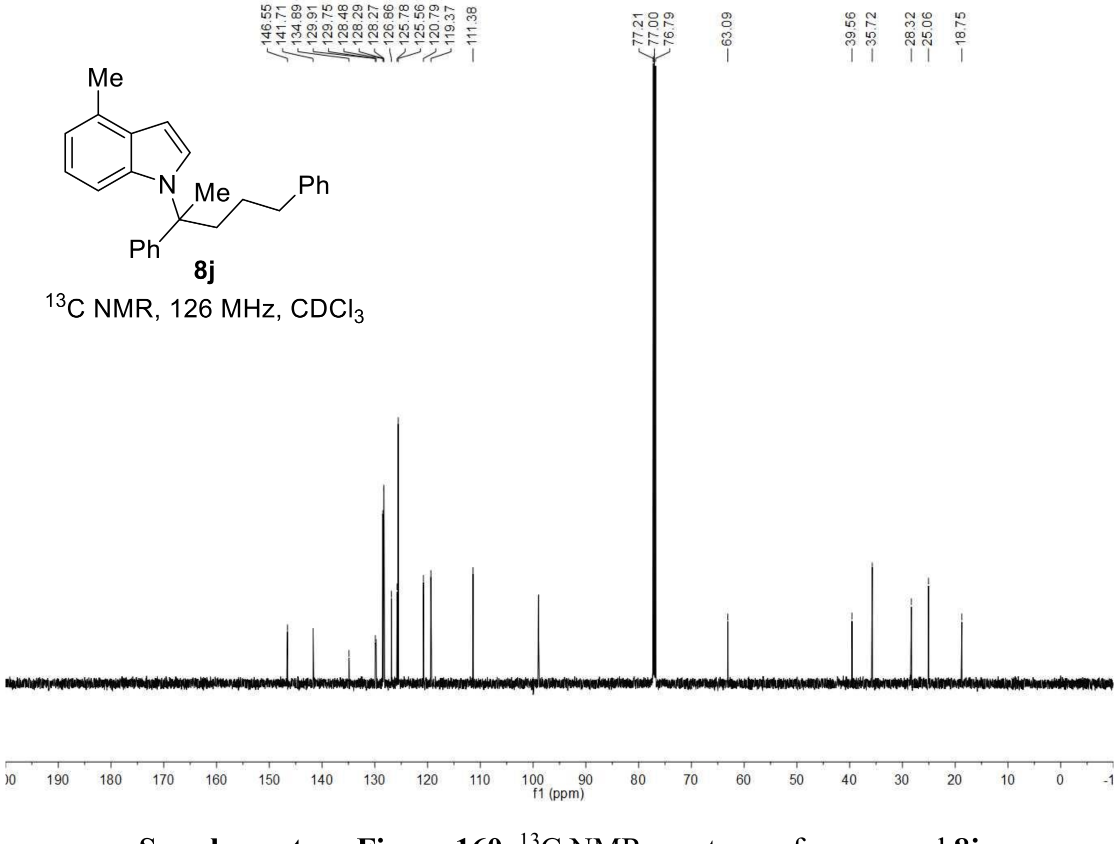

Supplementary Figure 160. ^{13}C NMR spectrum of compound **8j**

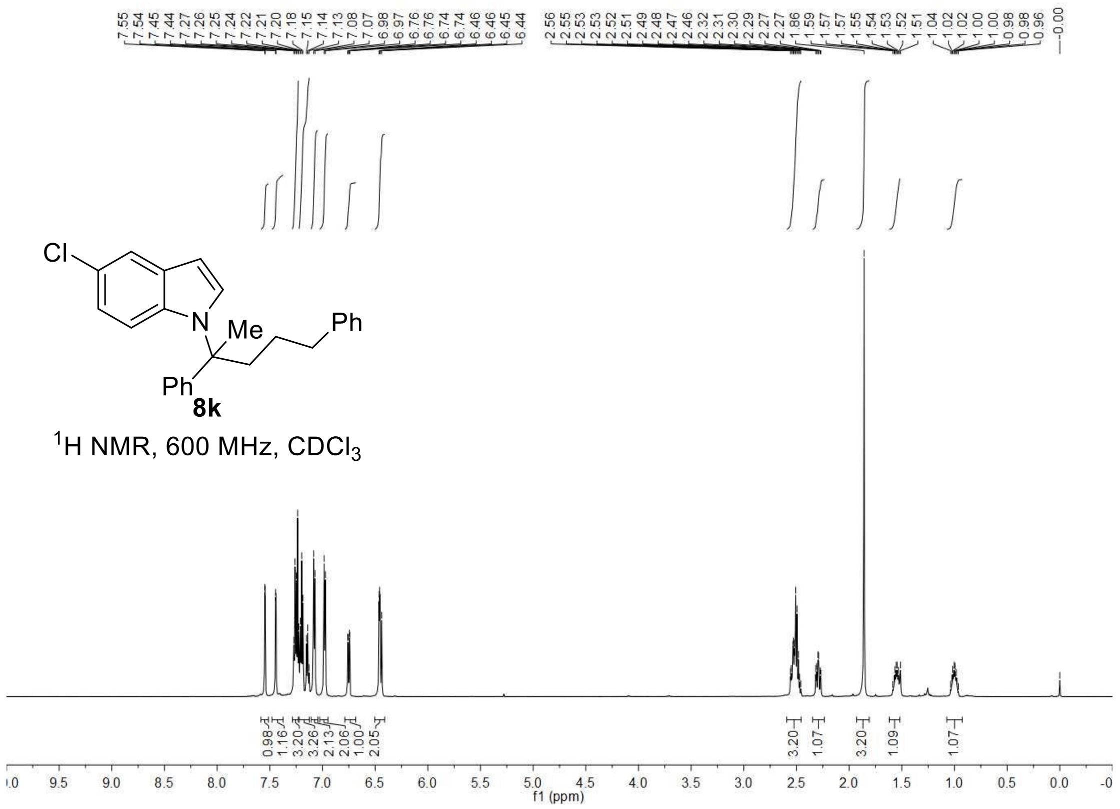

Supplementary Figure 161. ^{1}H NMR spectrum of compound **8k**

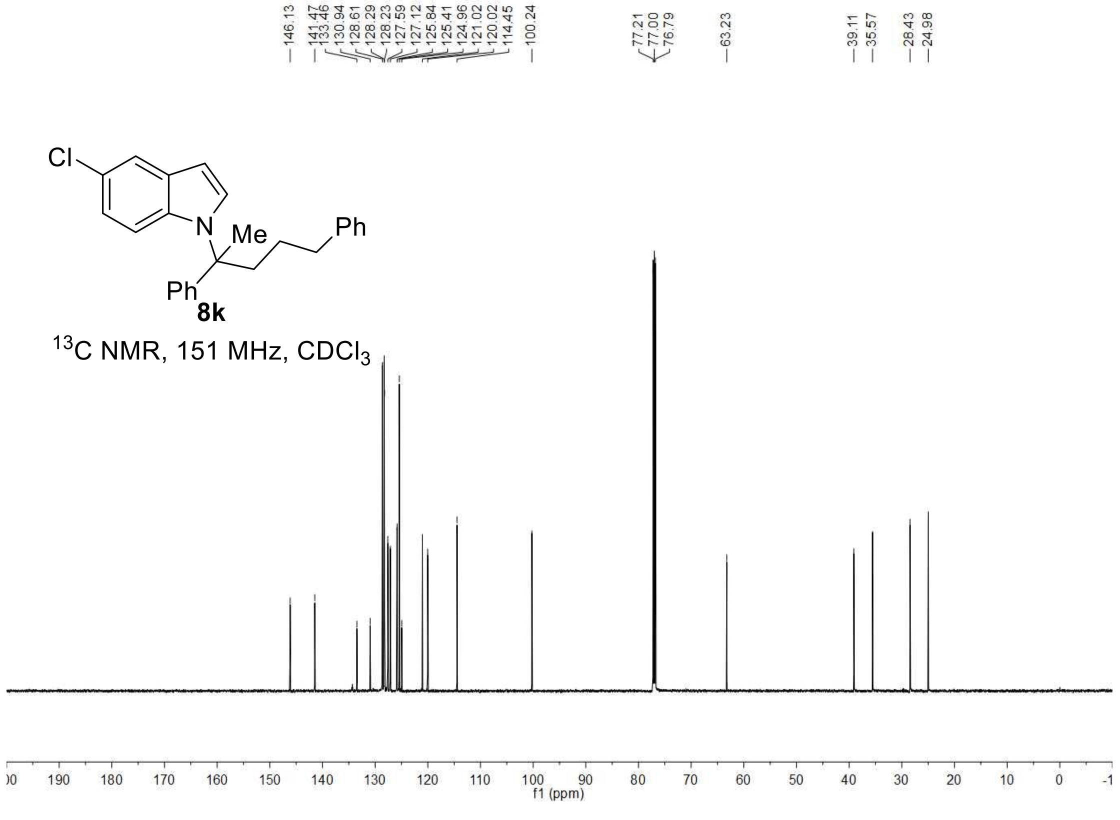

Supplementary Figure 162. ^{13}C NMR spectrum of compound **8k**

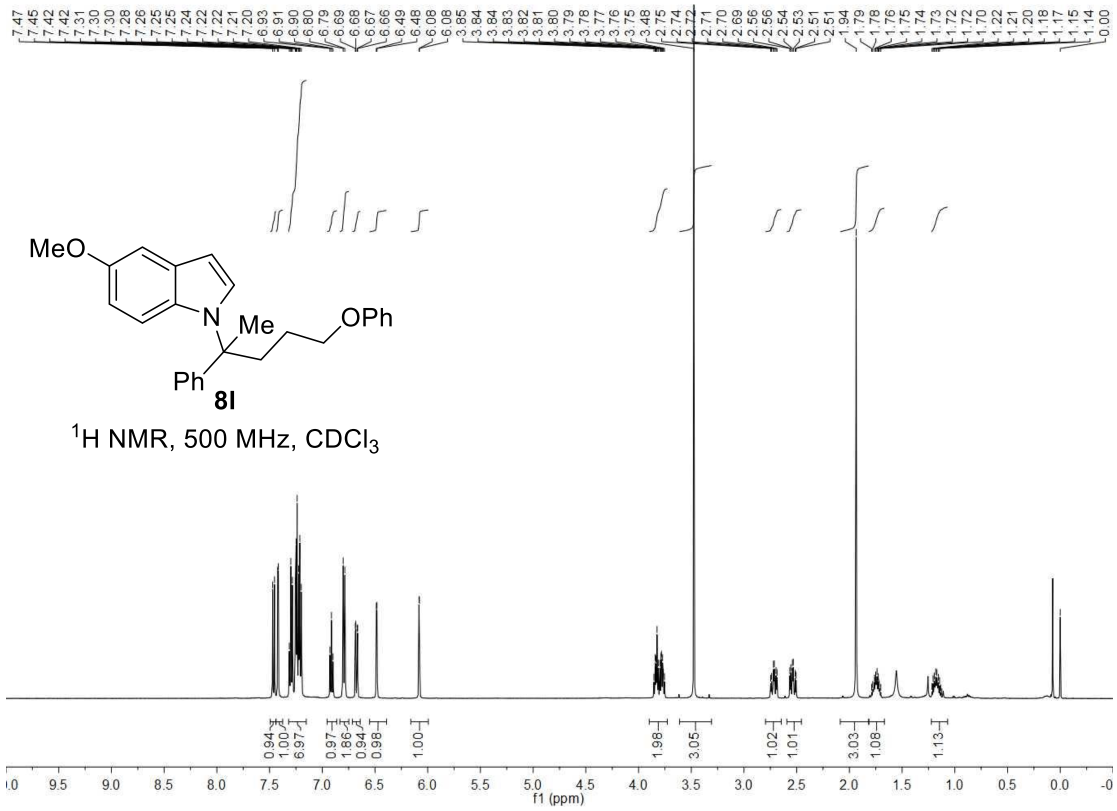

Supplementary Figure 163. ^{1}H NMR spectrum of compound **8l**

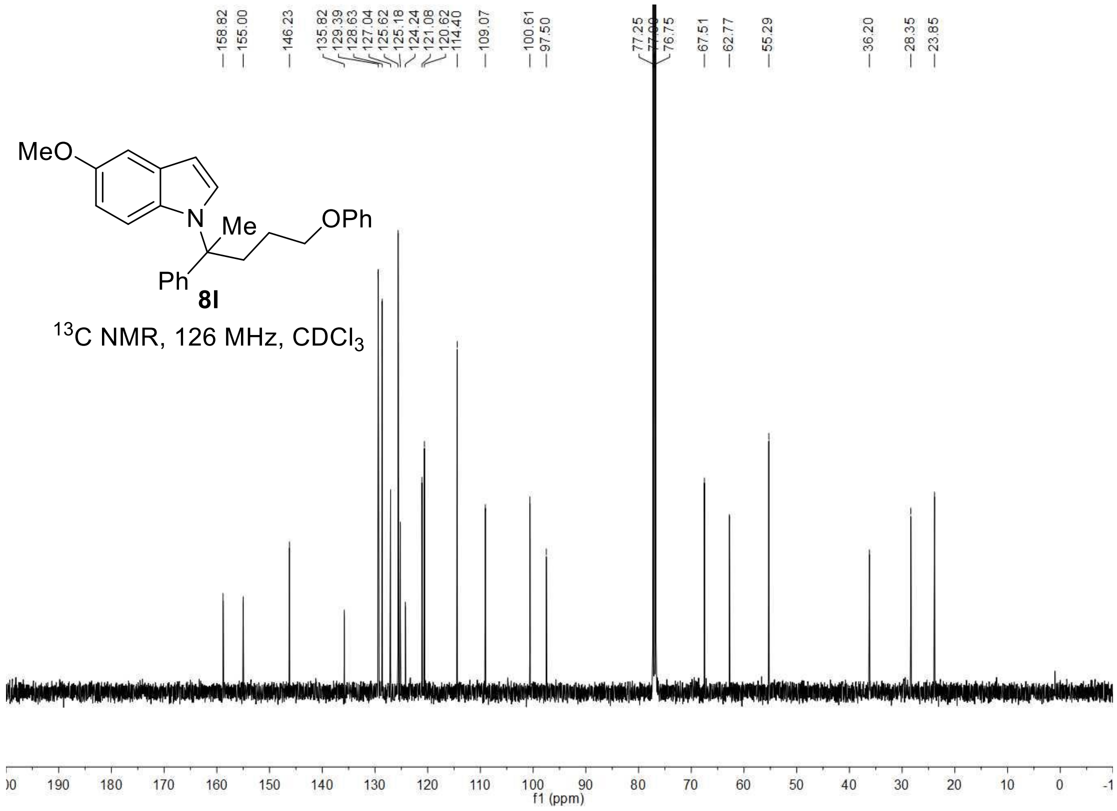

Supplementary Figure 164. ^{13}C NMR spectrum of compound **8l**

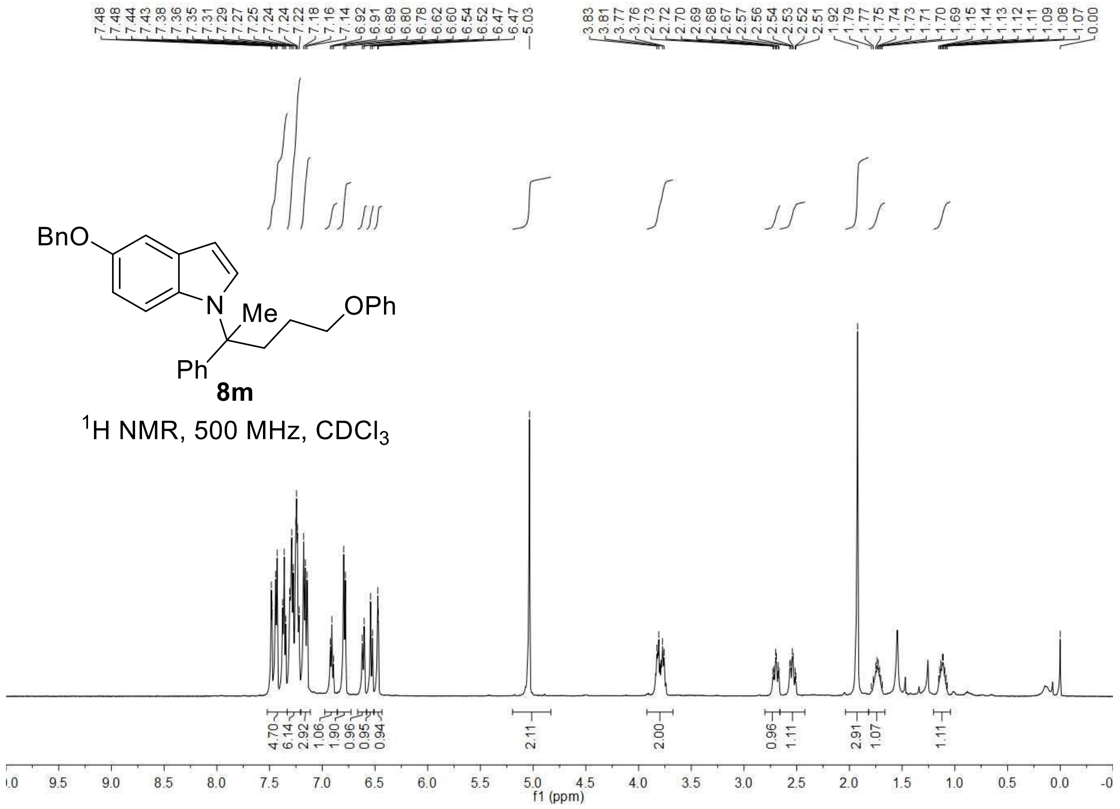

Supplementary Figure 165. ^{1}H NMR spectrum of compound **8m**

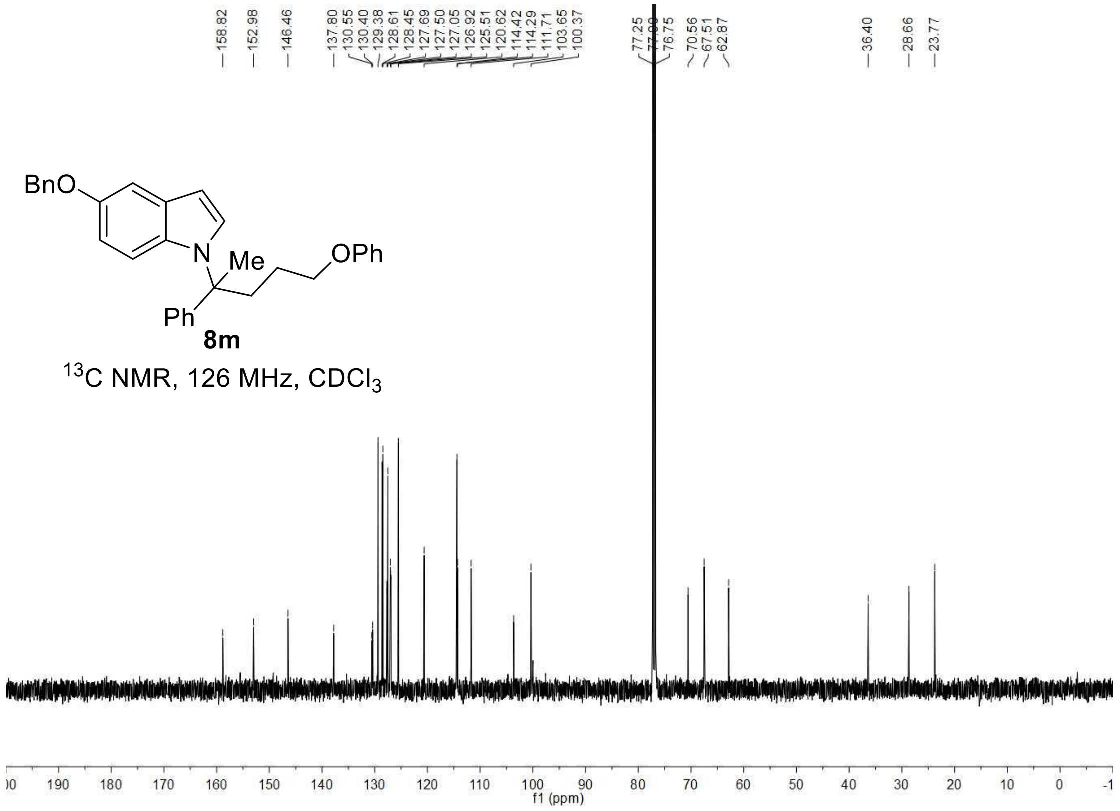

Supplementary Figure 166. ^{13}C NMR spectrum of compound **8m**

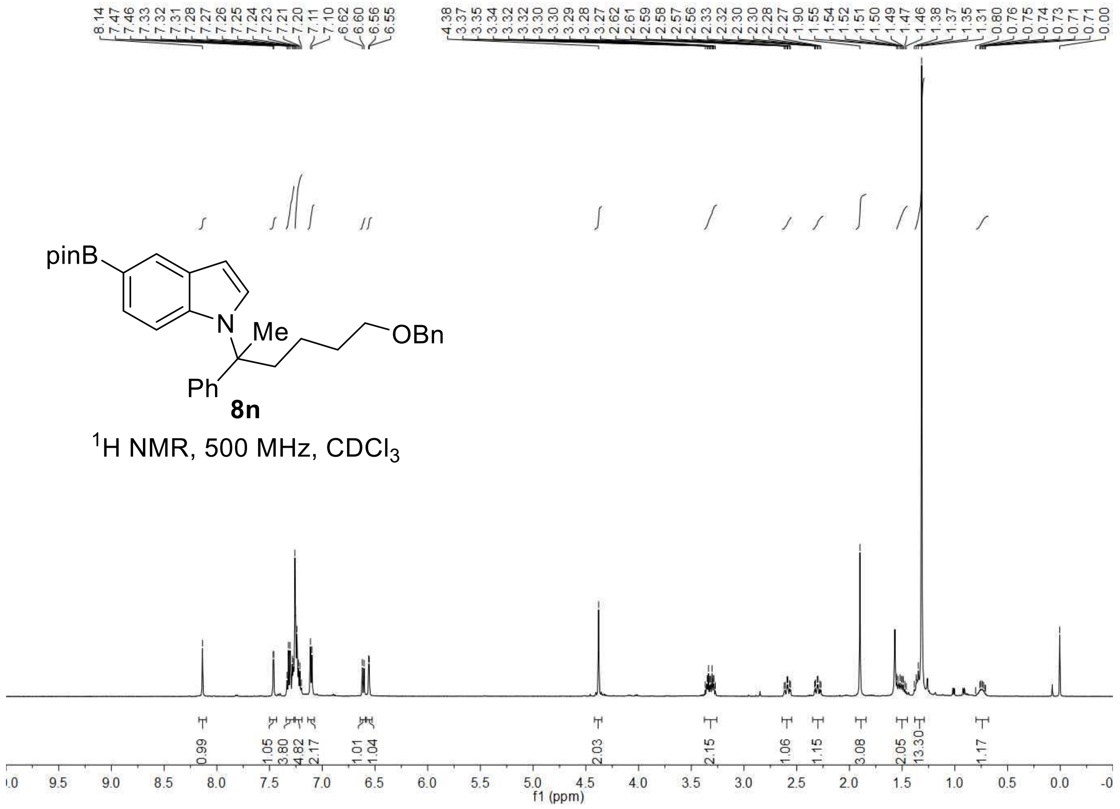

Supplementary Figure 167. ^{1}H NMR spectrum of compound **8n**

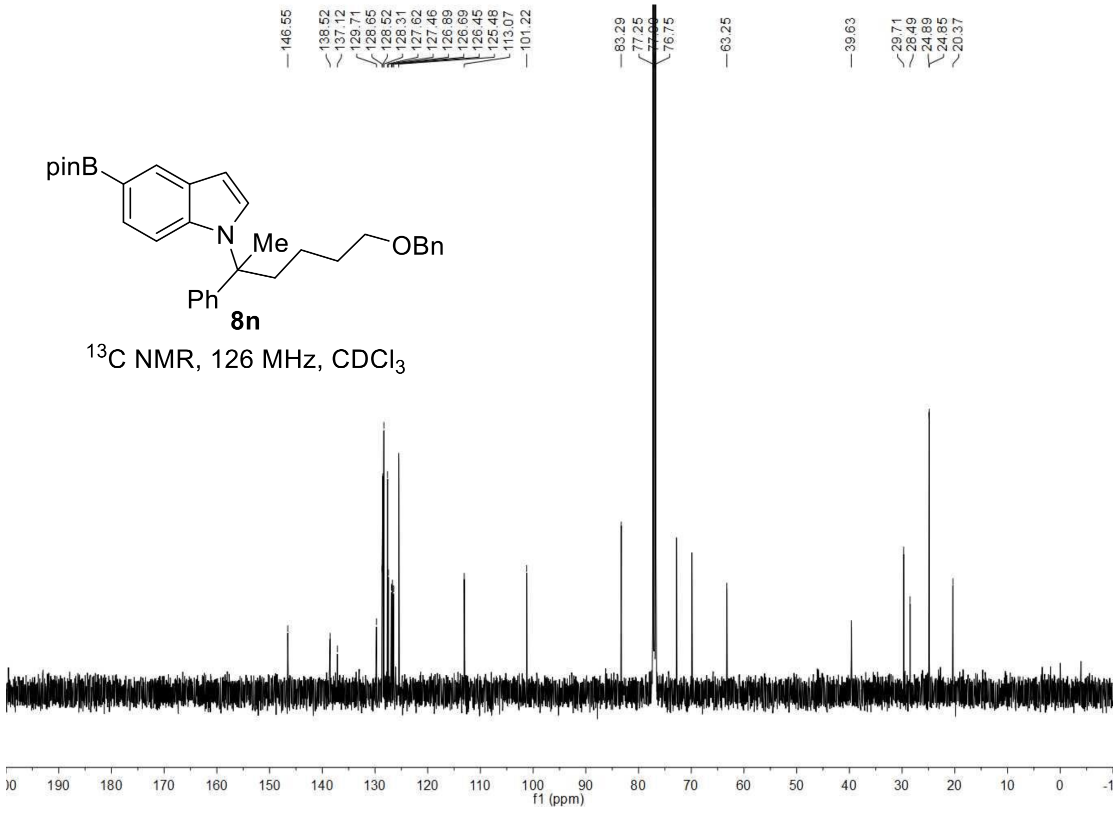

Supplementary Figure 168. ^{13}C NMR spectrum of compound **8n**

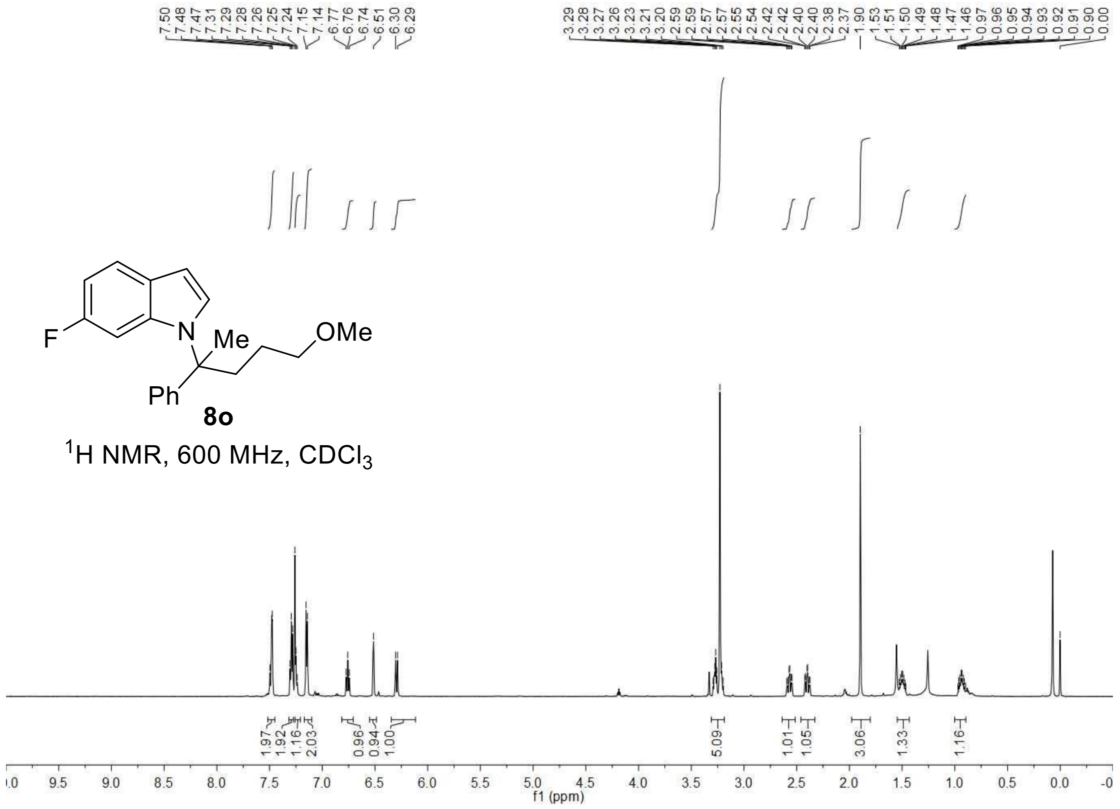

Supplementary Figure 169. ^{1}H NMR spectrum of compound **8o**

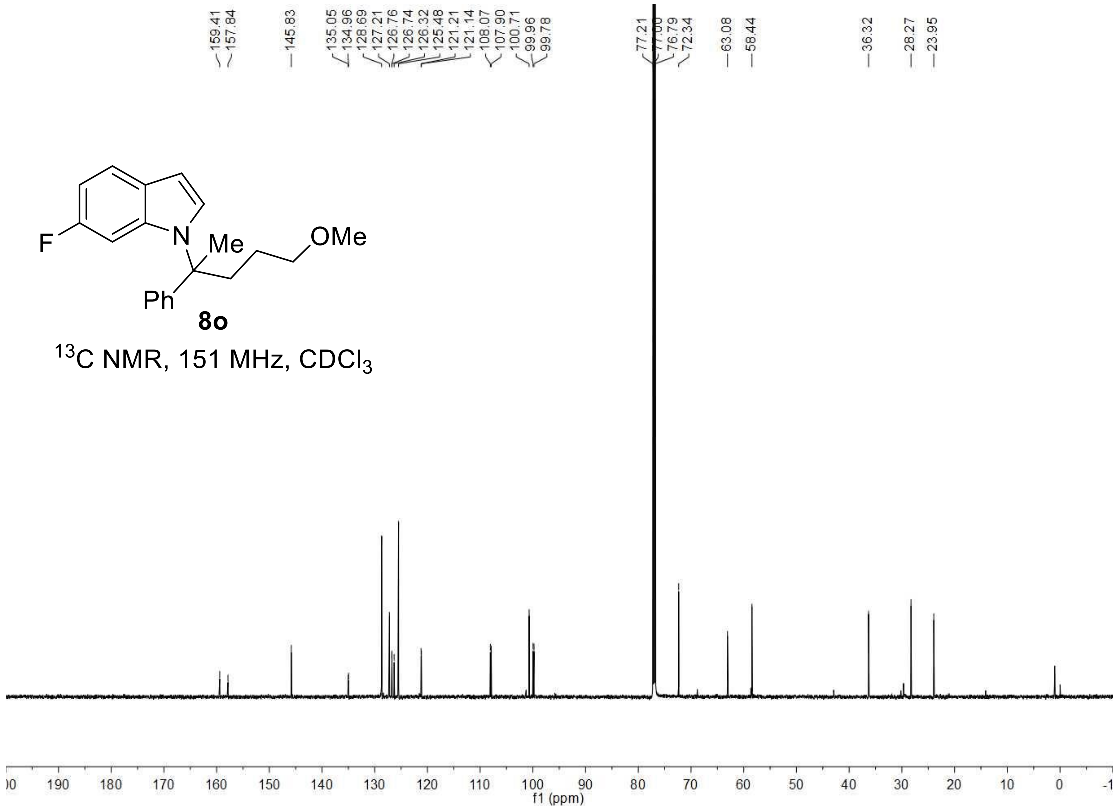

Supplementary Figure 170. ^{13}C NMR spectrum of compound **8o**

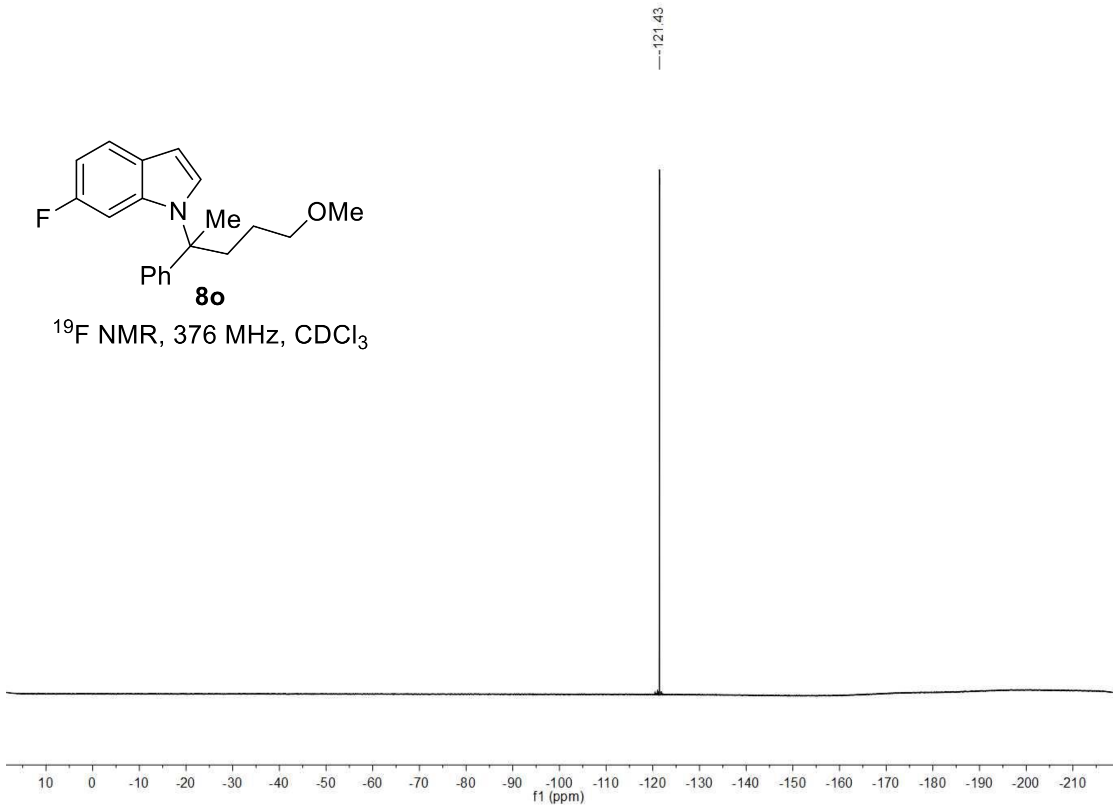

Supplementary Figure 171. ^{19}F NMR spectrum of compound **8o**

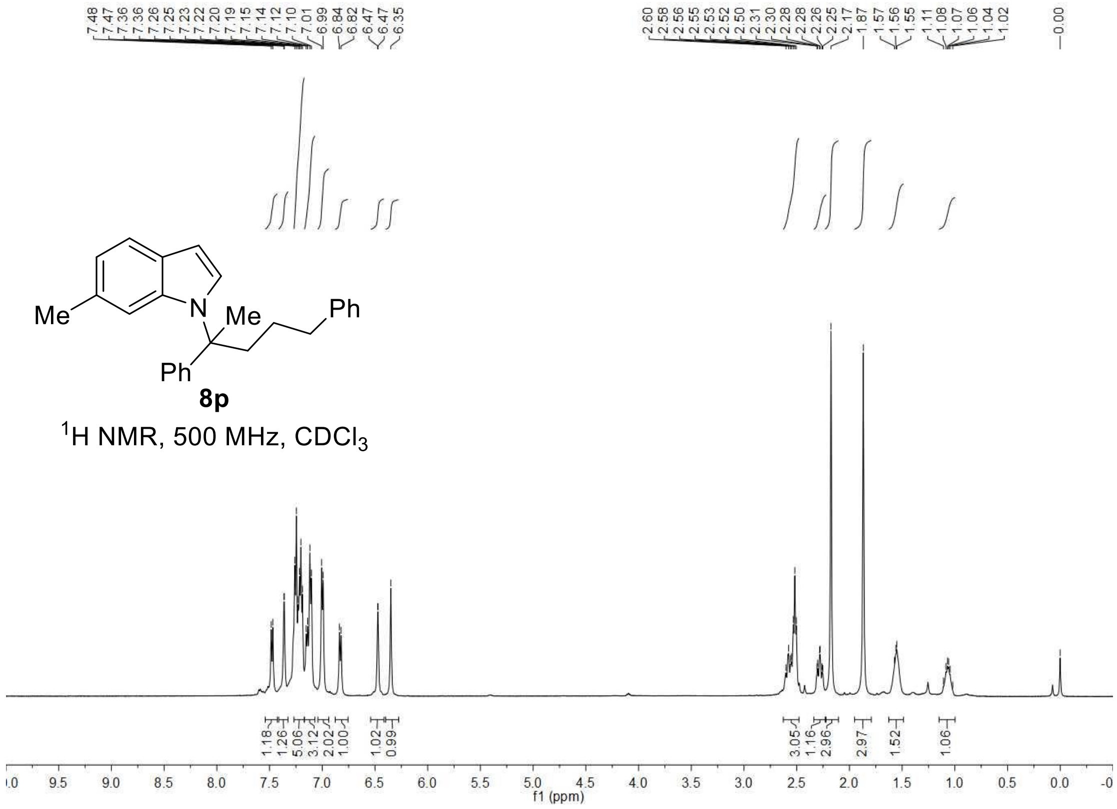

Supplementary Figure 172. ^{1}H NMR spectrum of compound **8p**

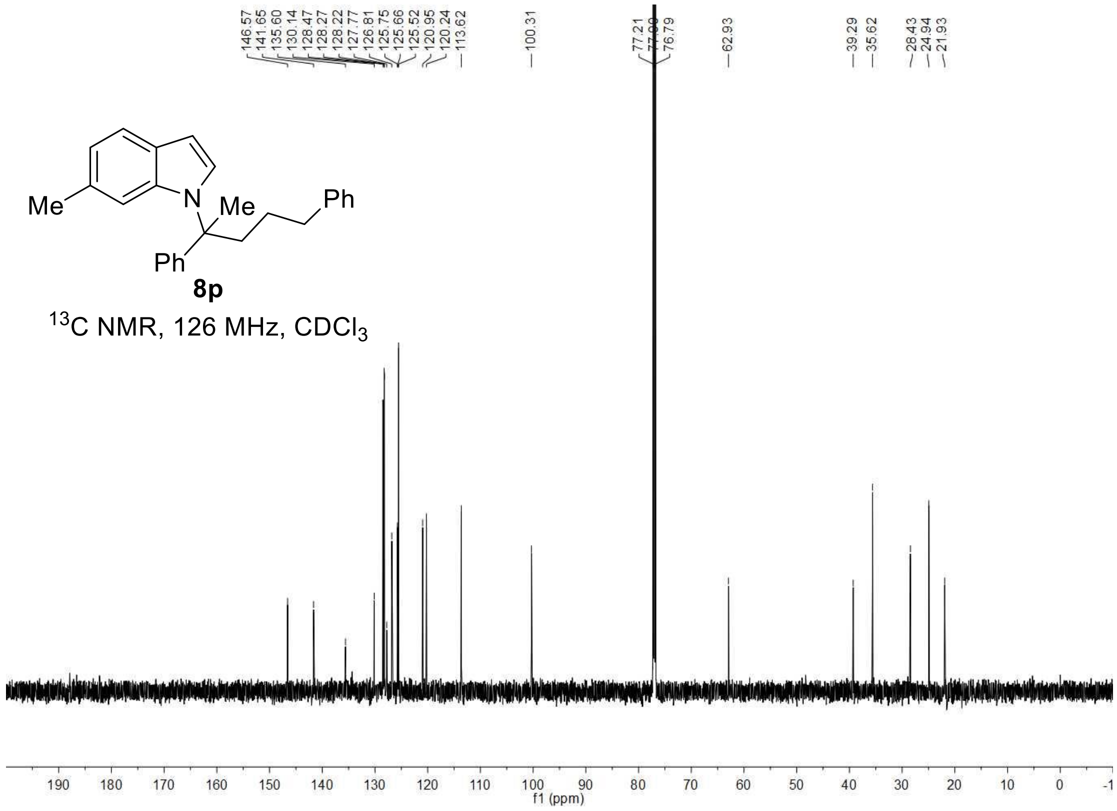

Supplementary Figure 173. ^{13}C NMR spectrum of compound **8p**

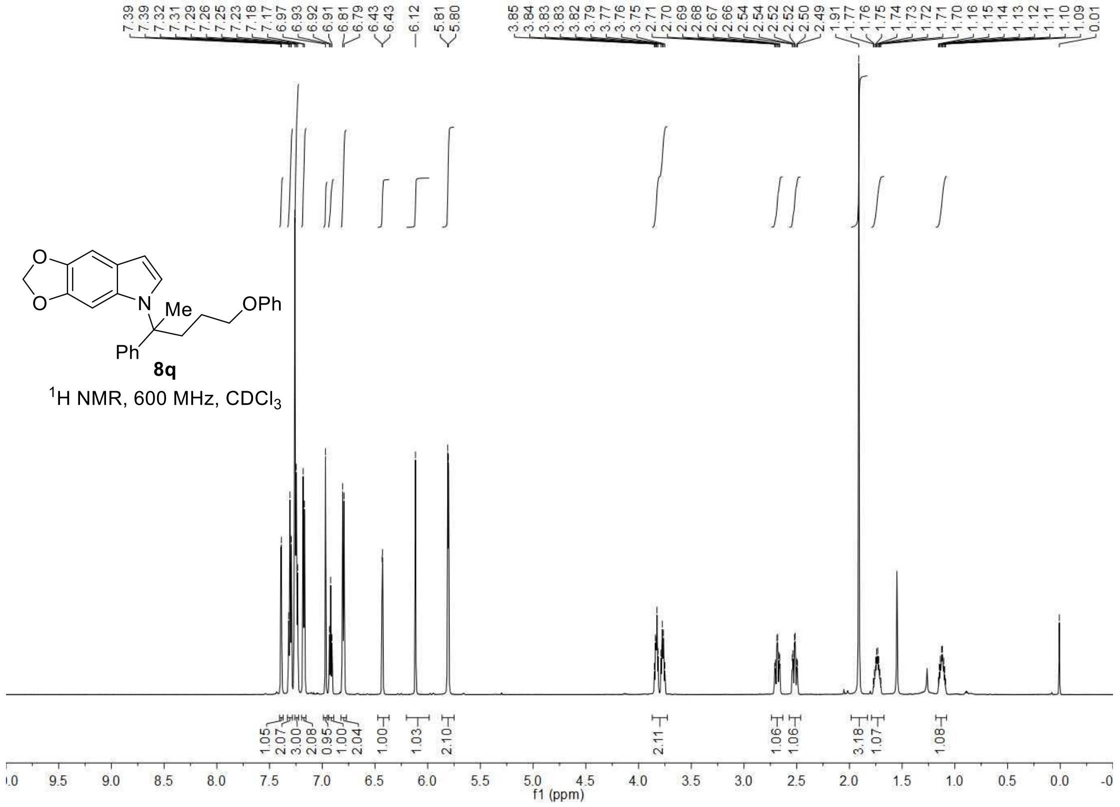

Supplementary Figure 174. ^{1}H NMR spectrum of compound **8q**

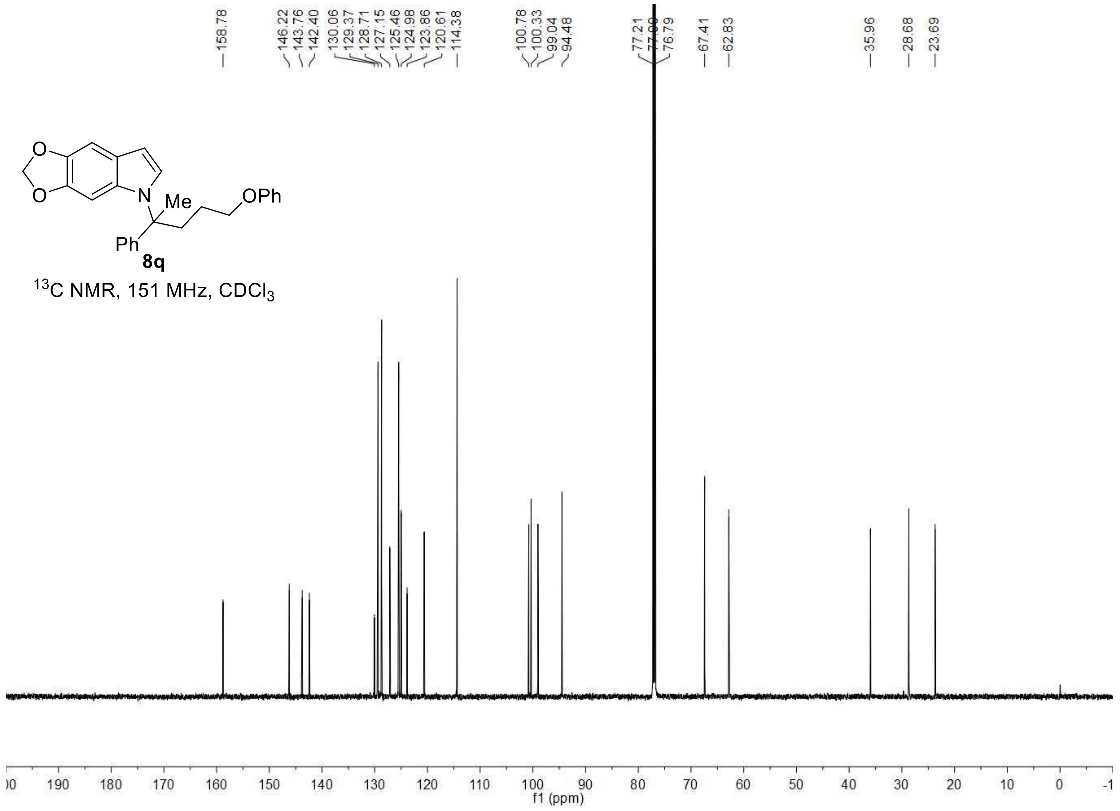

Supplementary Figure 175. ^{13}C NMR spectrum of compound **8q**

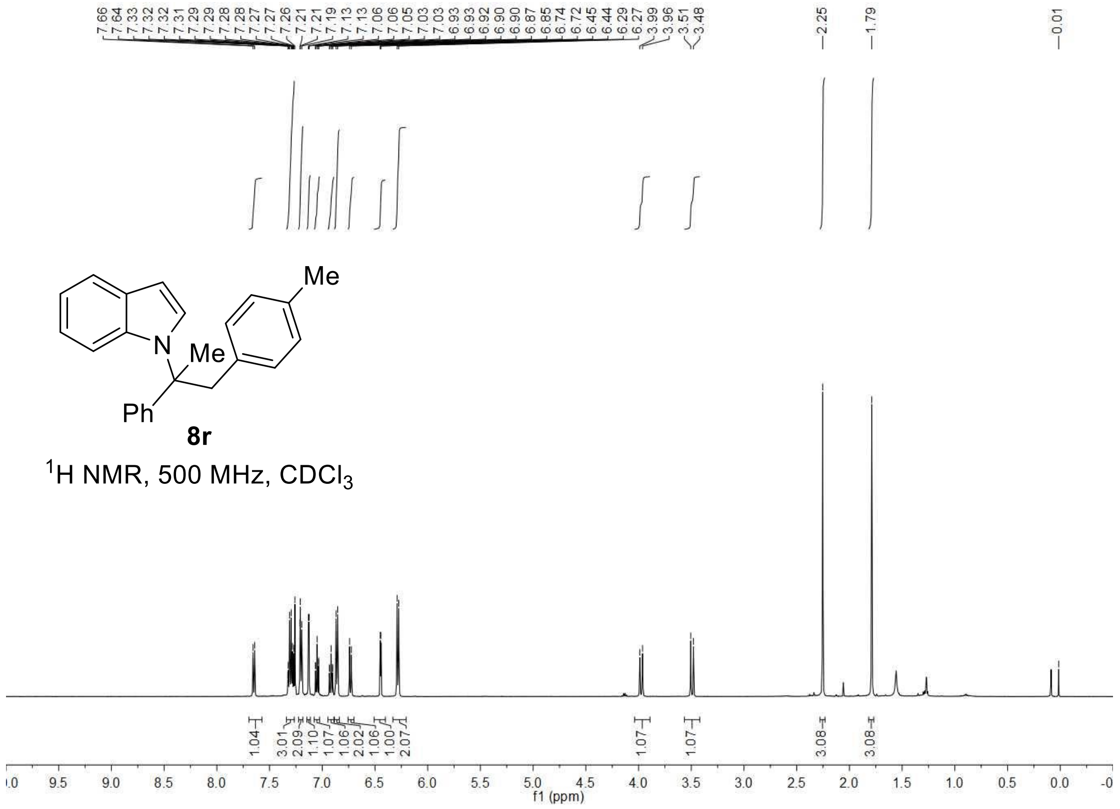

Supplementary Figure 176. ^{1}H NMR spectrum of compound **8r**

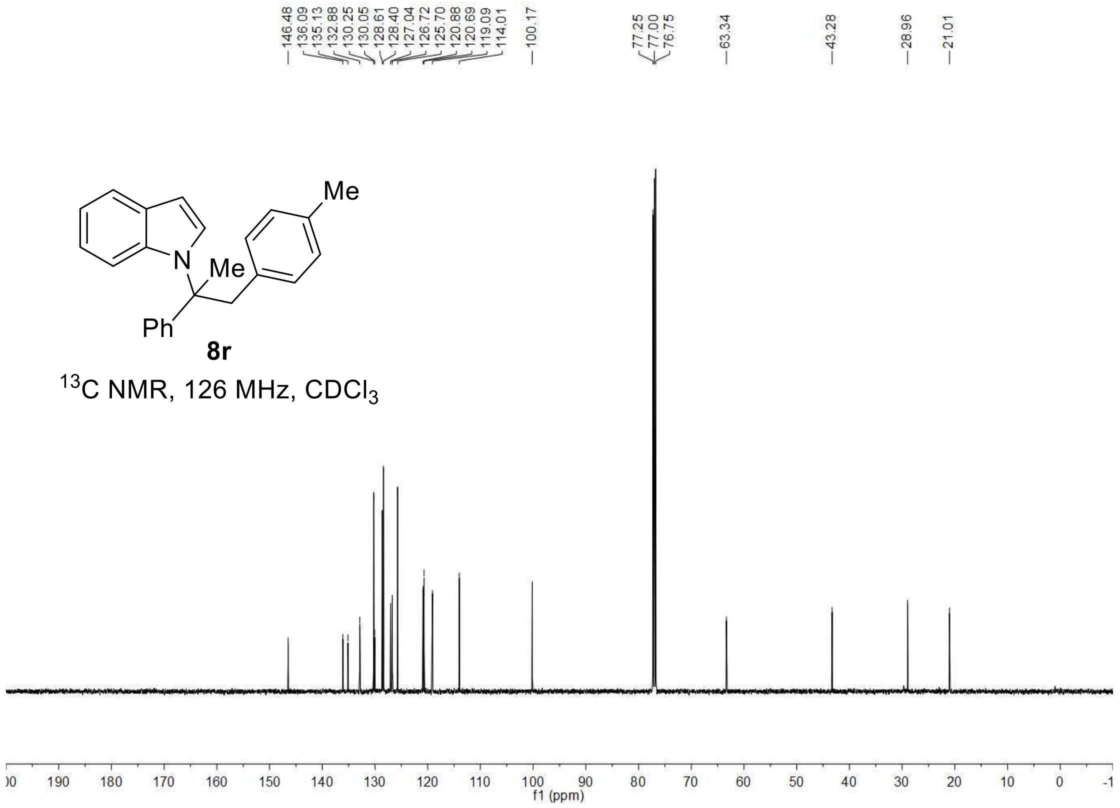

Supplementary Figure 177. ^{13}C NMR spectrum of compound **8r**

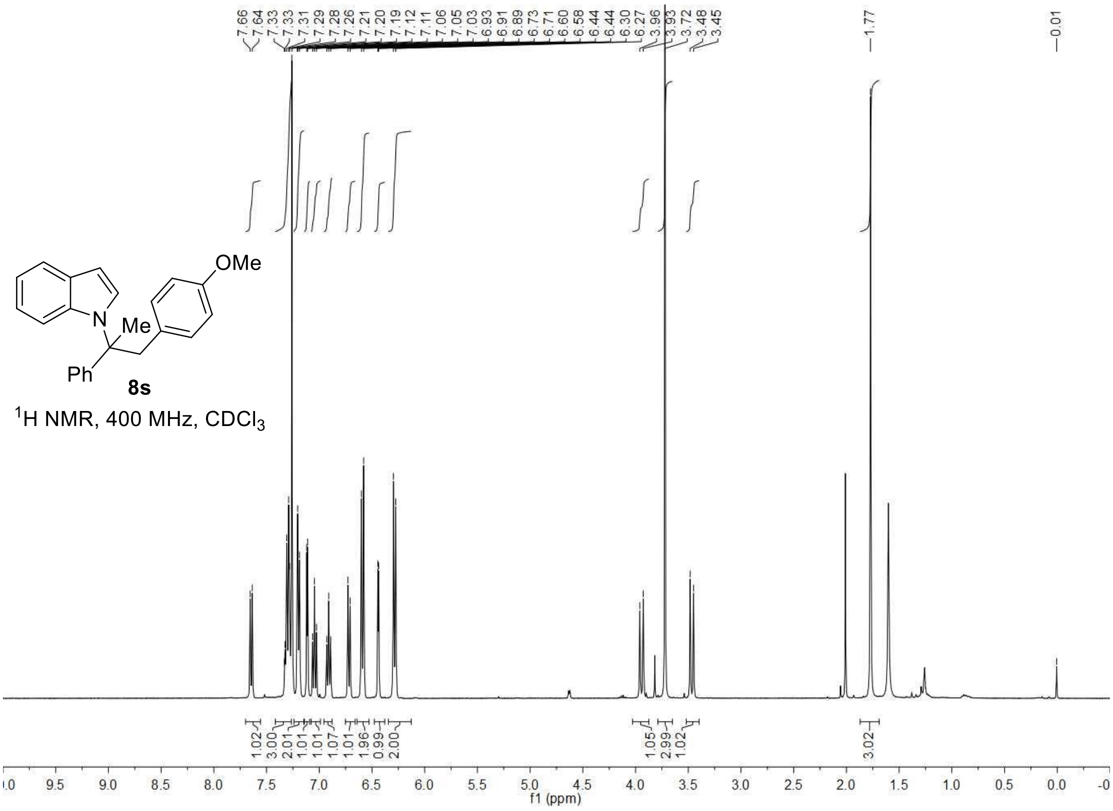

Supplementary Figure 178. ^{1}H NMR spectrum of compound **8s**

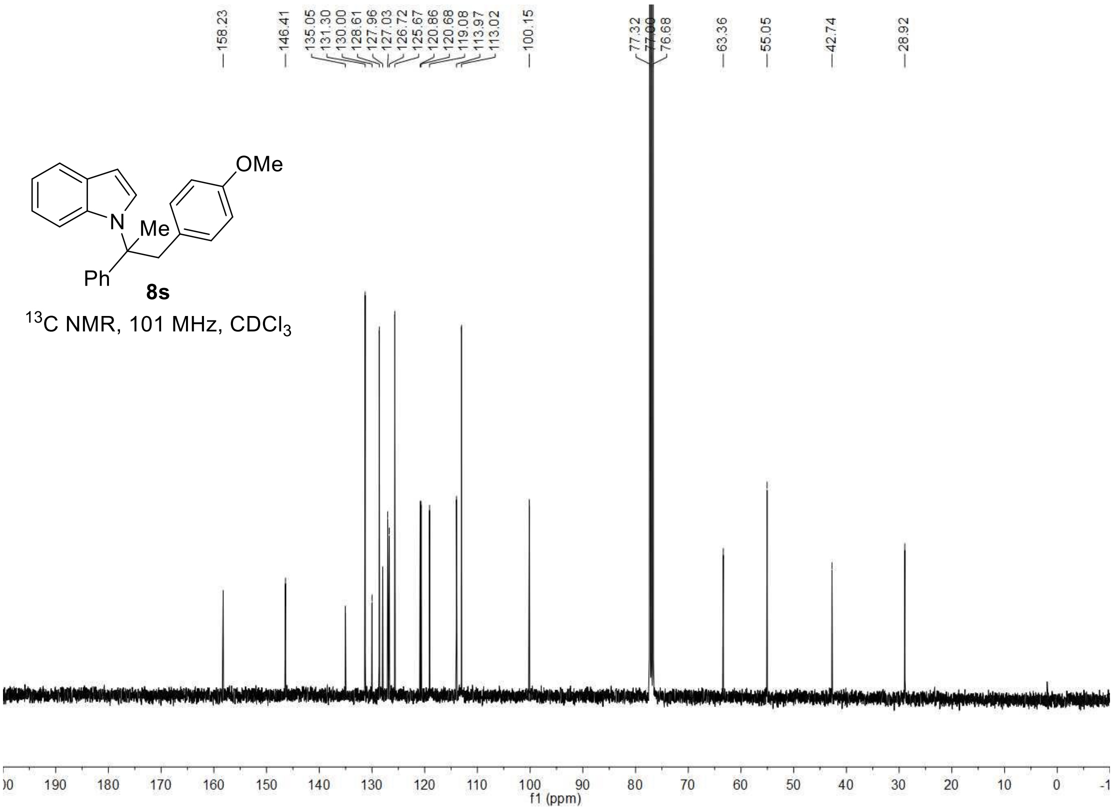

Supplementary Figure 179. ^{13}C NMR spectrum of compound **8s**

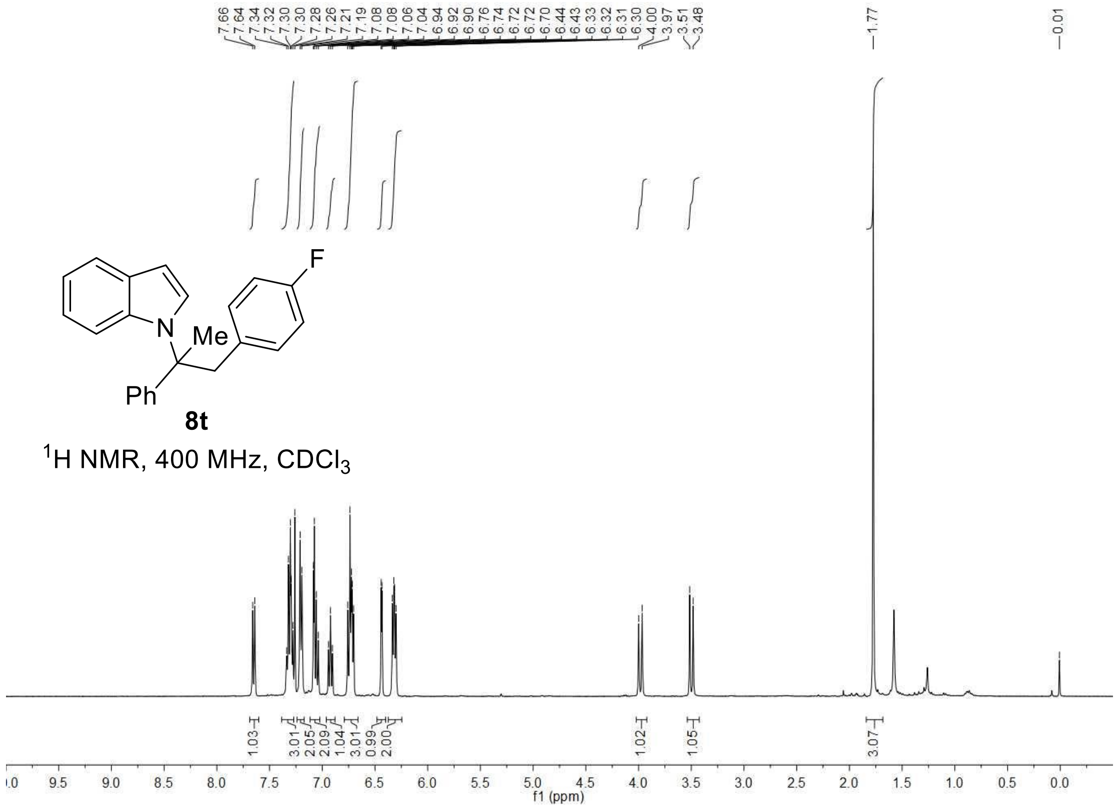

Supplementary Figure 180. ^{1}H NMR spectrum of compound **8t**

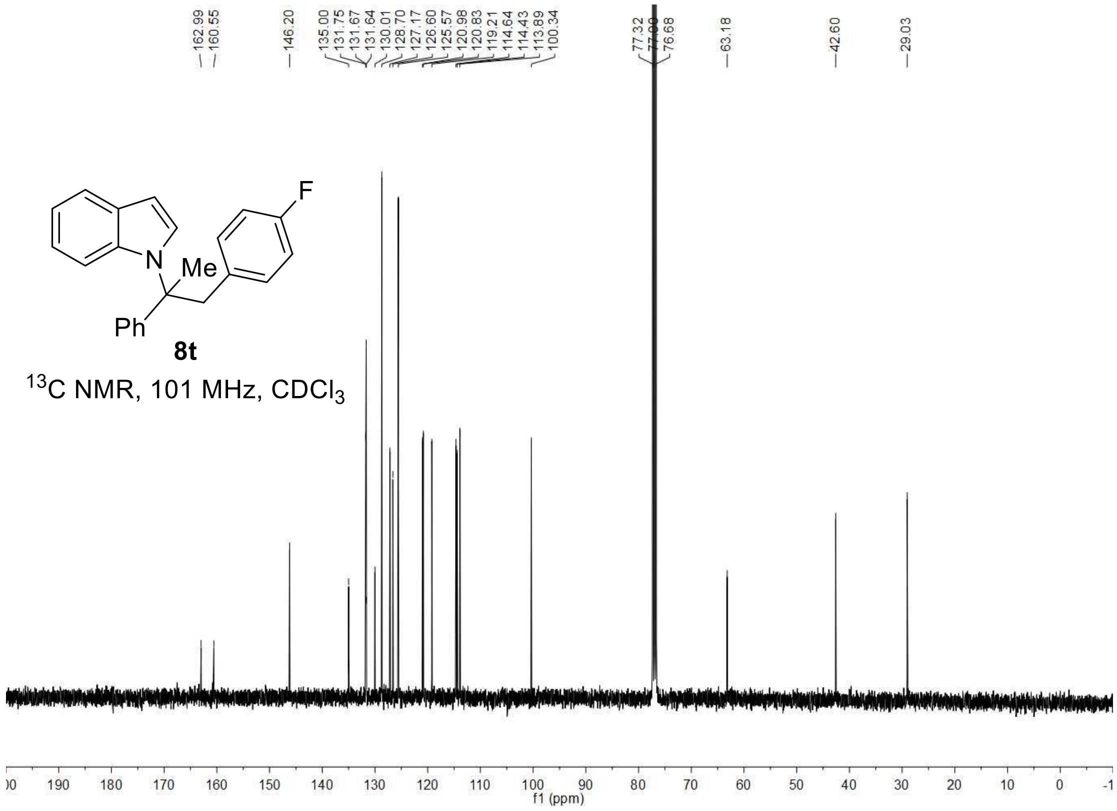

Supplementary Figure 181. ^{13}C NMR spectrum of compound **8t**

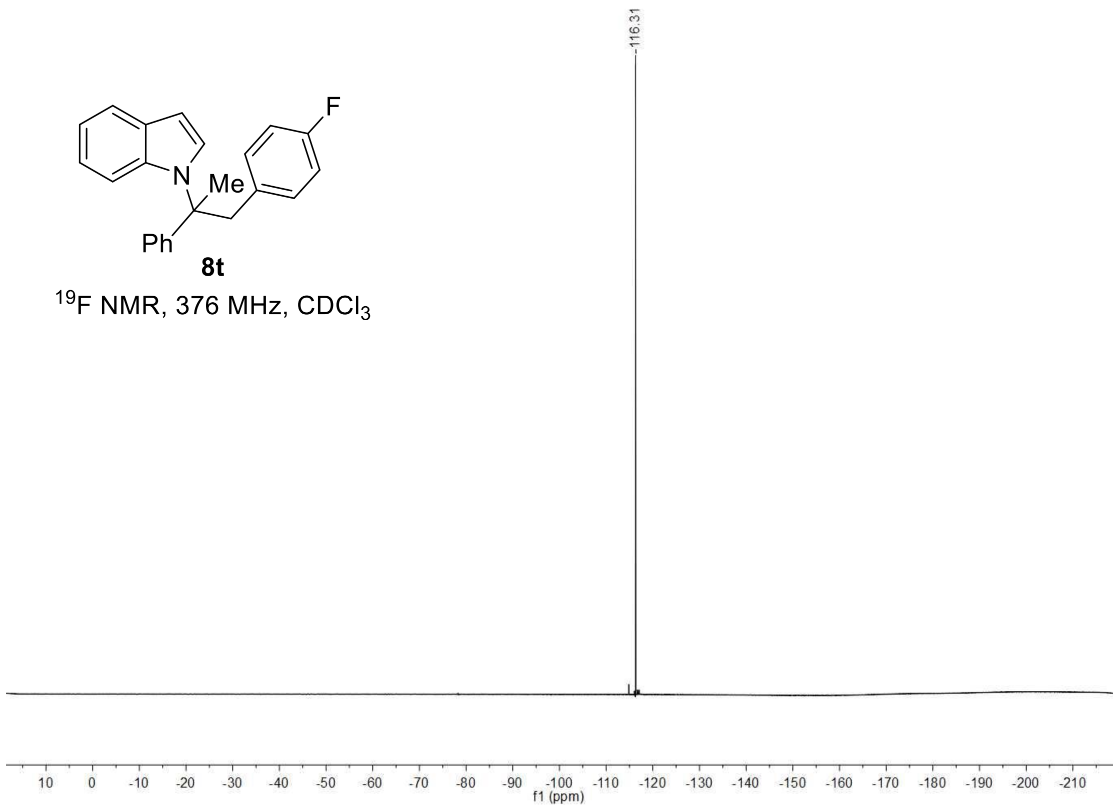

Supplementary Figure 182. ^{19}F NMR spectrum of compound **8t**

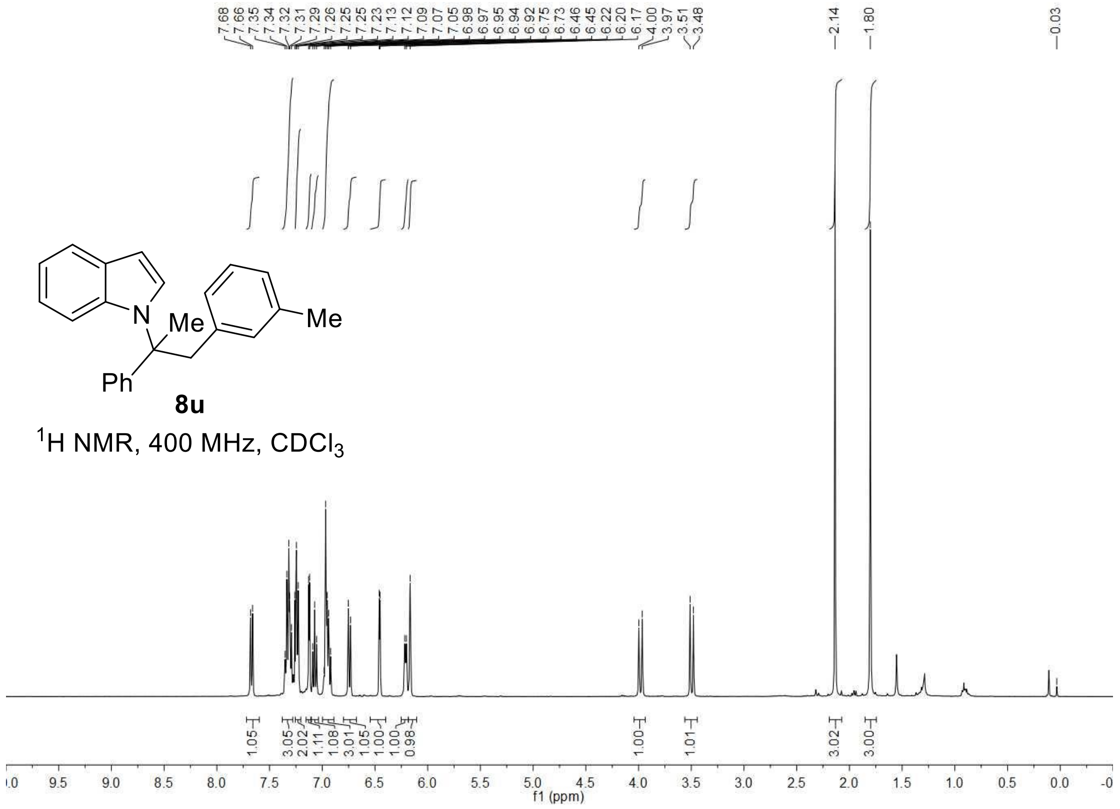

Supplementary Figure 183. ^{1}H NMR spectrum of compound **8u**

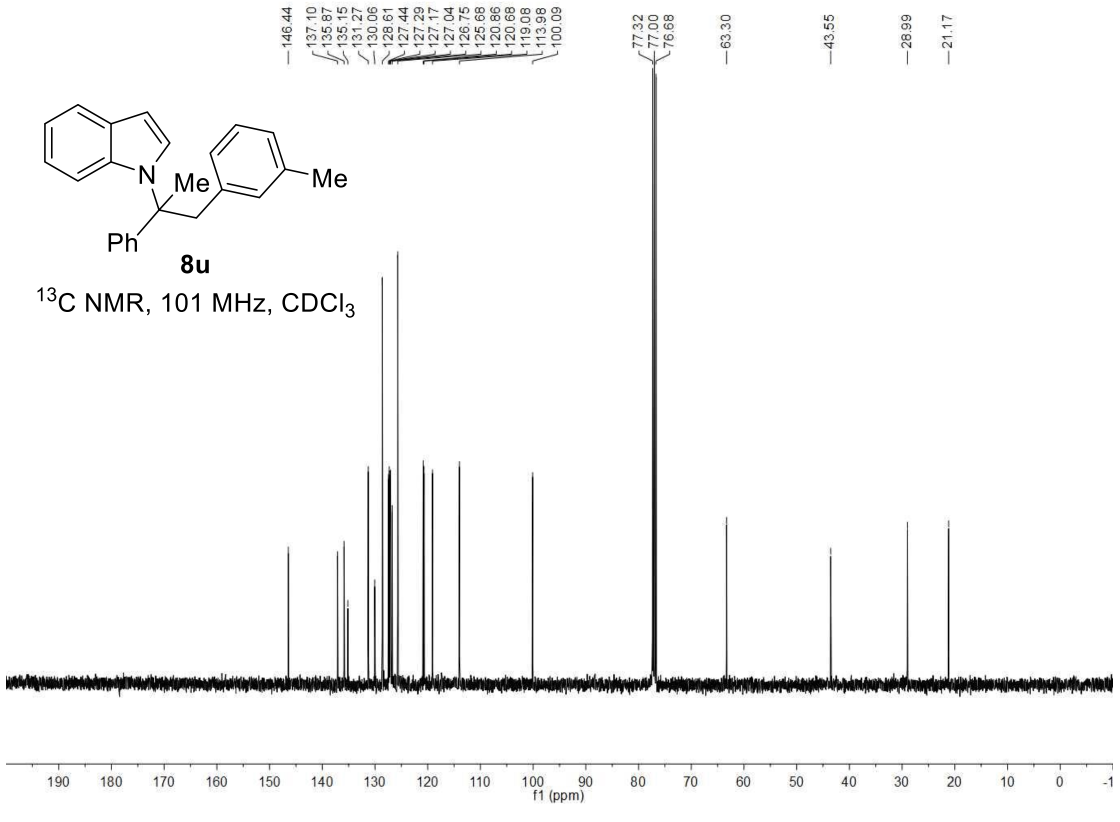

Supplementary Figure 184. ^{13}C NMR spectrum of compound **8u**

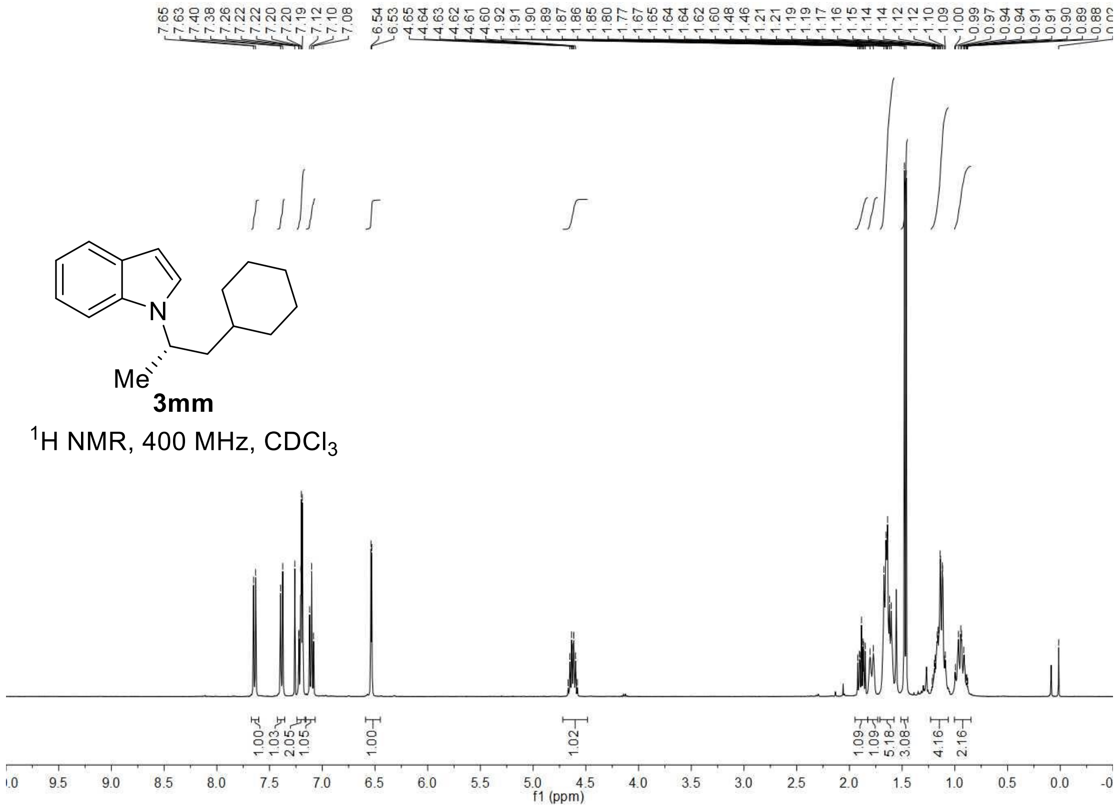

Supplementary Figure 185. ^1H NMR spectrum of compound **3mm**

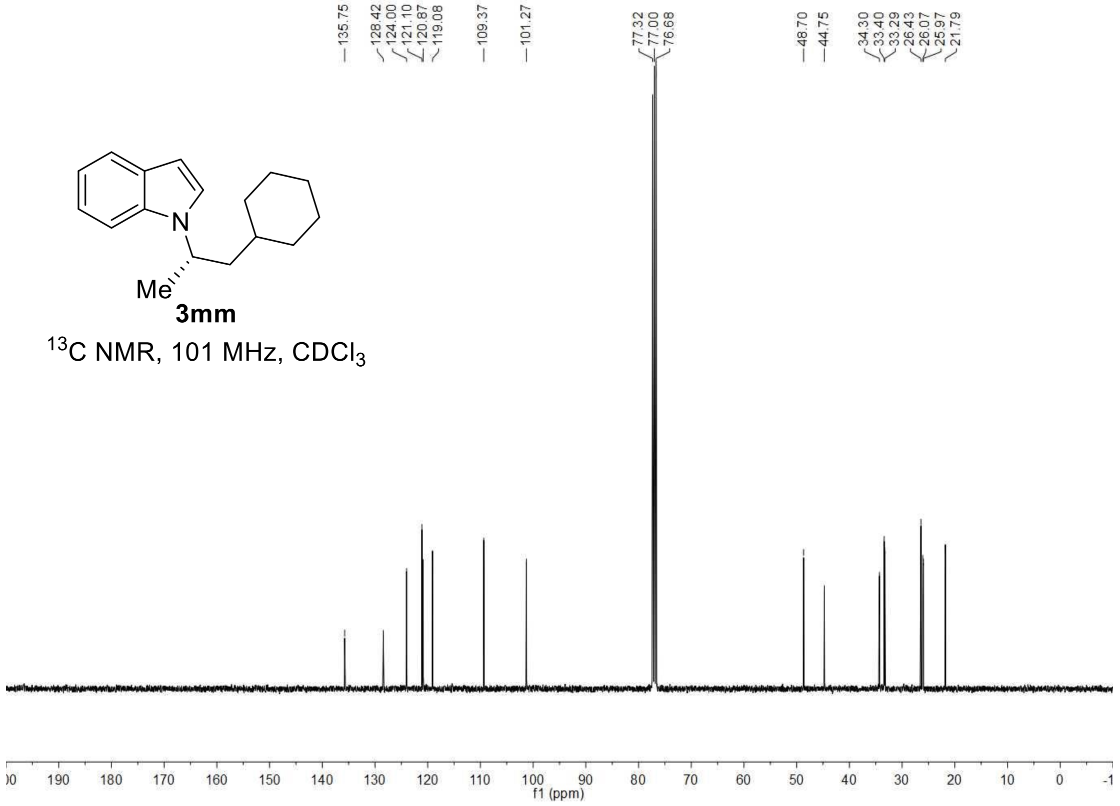

Supplementary Figure 186. ^{13}C NMR spectrum of compound **3mm**

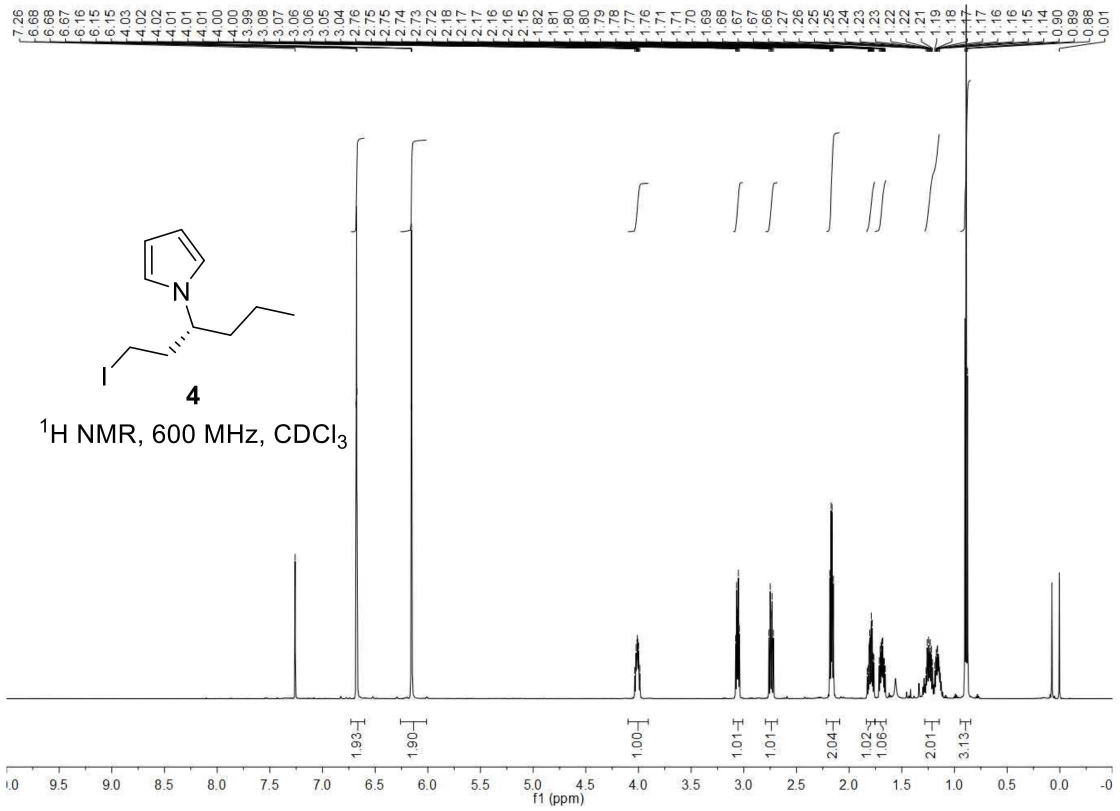

Supplementary Figure 187. ^{1}H NMR spectrum of compound **4**

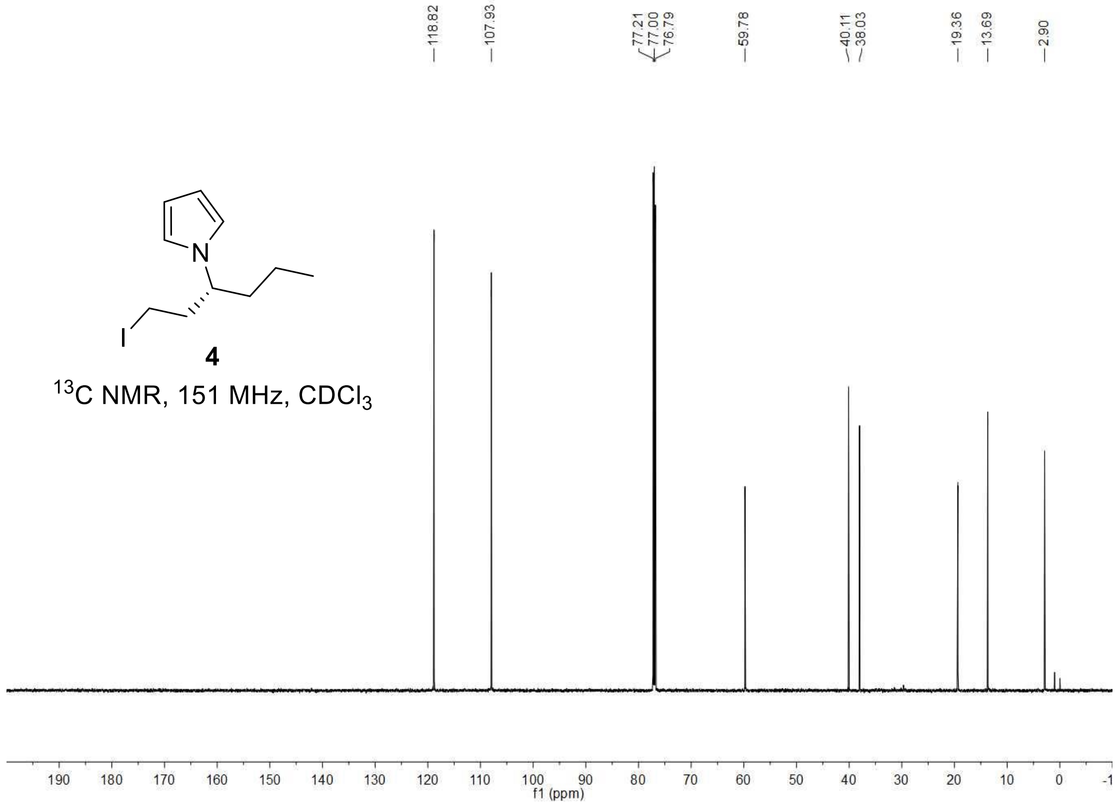

Supplementary Figure 188. ^{13}C NMR spectrum of compound **4**

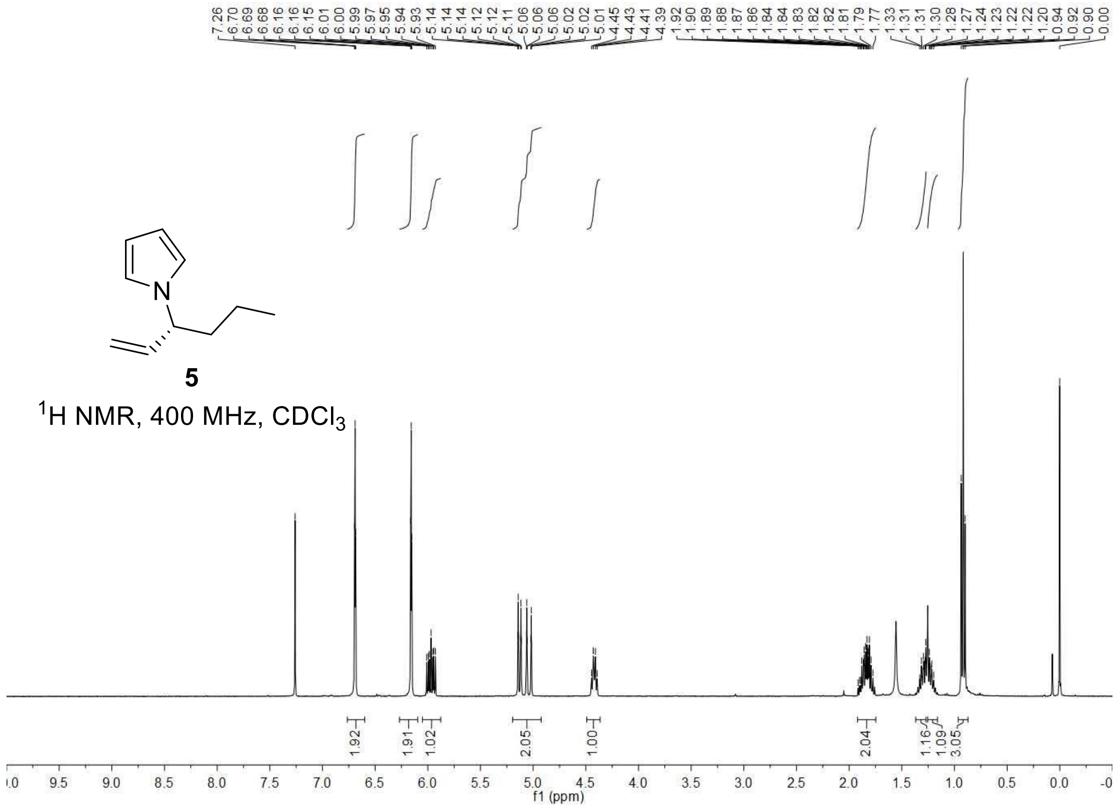

Supplementary Figure 189. ^{1}H NMR spectrum of compound **5**

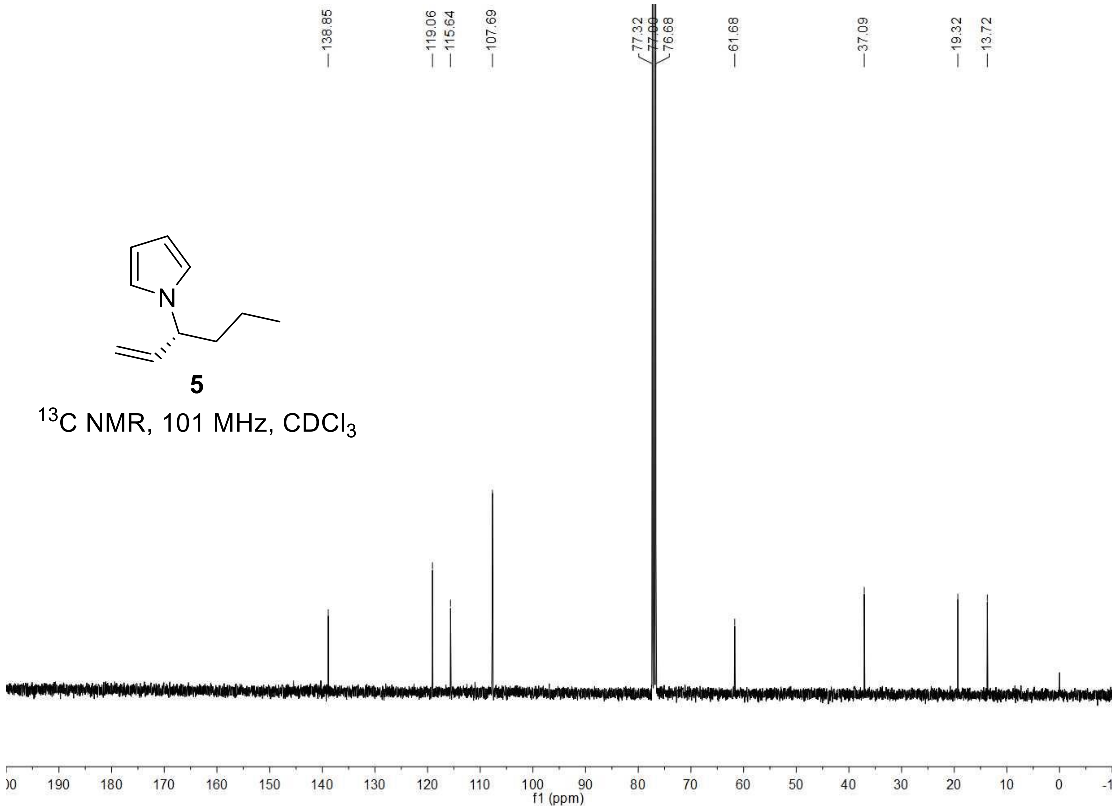

Supplementary Figure 190. ^{13}C NMR spectrum of compound **5**

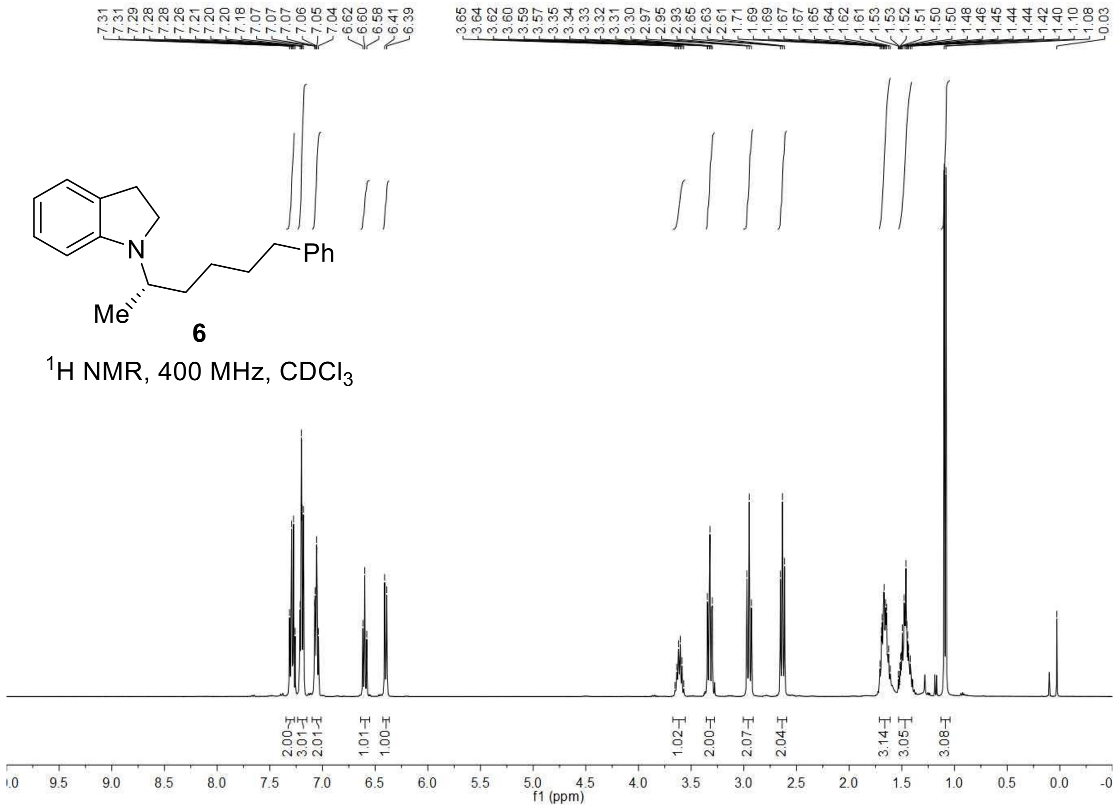

Supplementary Figure 191. ^{1}H NMR spectrum of compound **6**

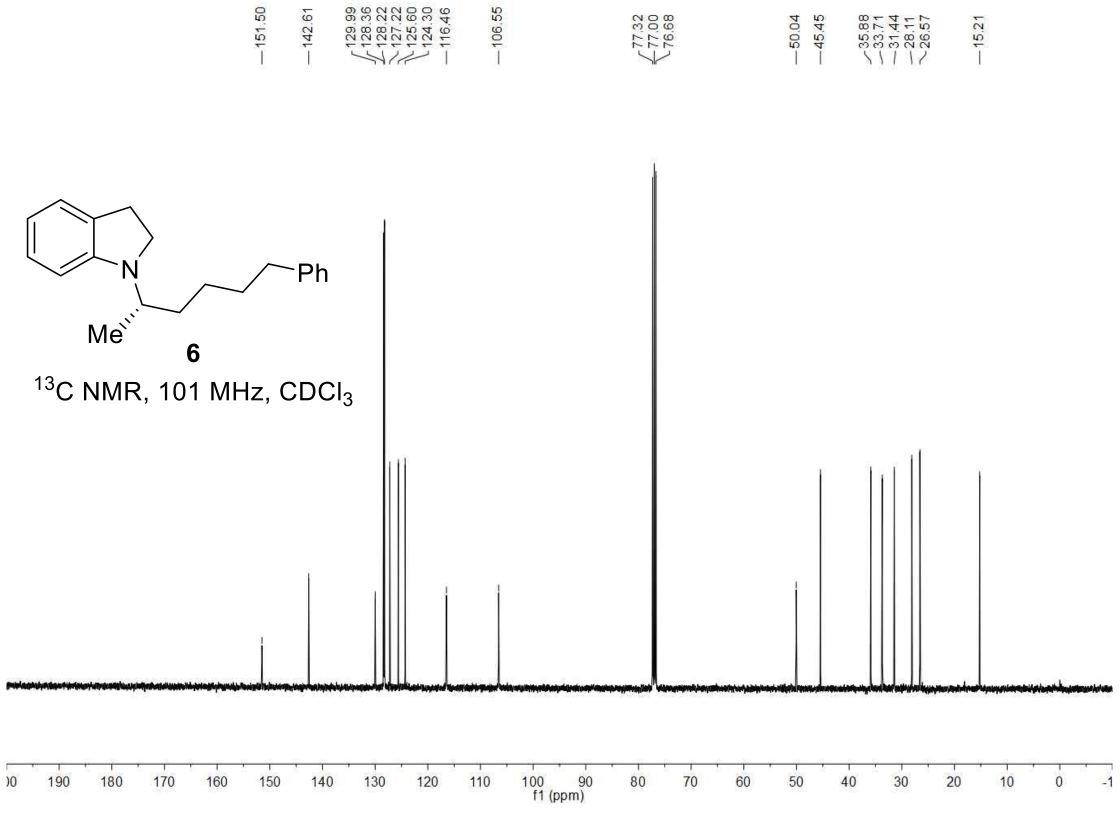

Supplementary Figure 192. ^{13}C NMR spectrum of compound **6**

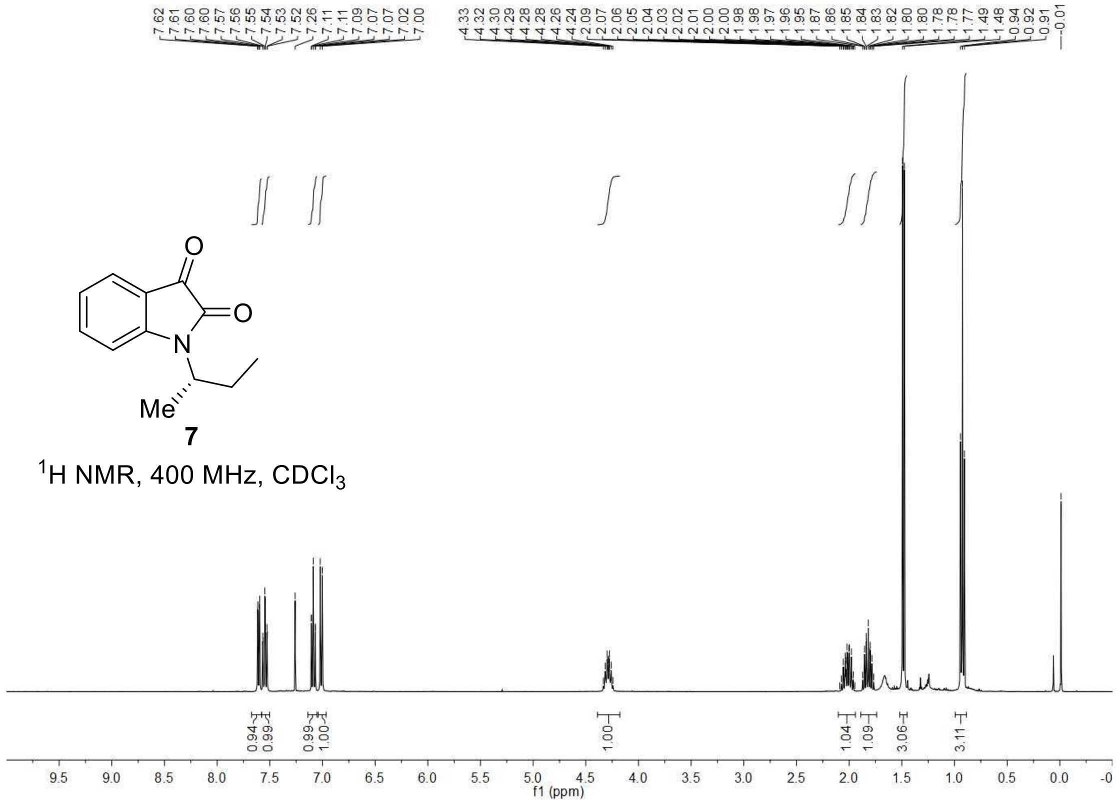

Supplementary Figure 193. ^{1}H NMR spectrum of compound **7**

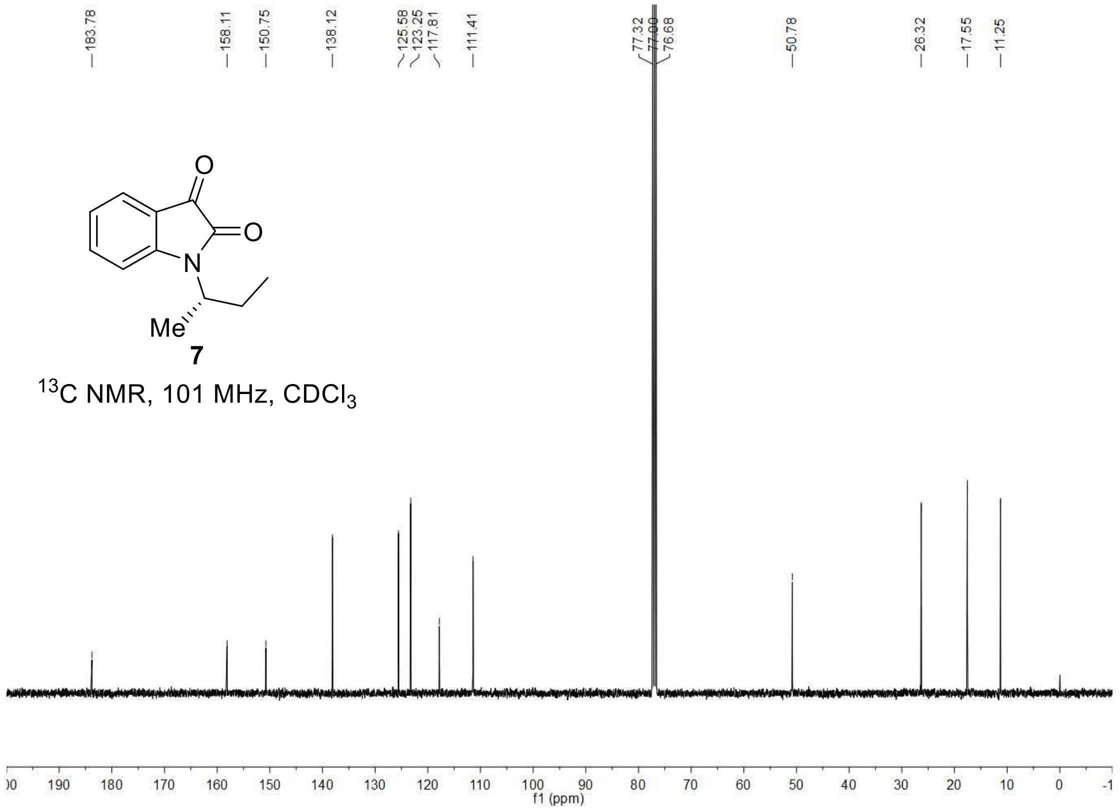

Supplementary Figure 194. ^{13}C NMR spectrum of compound **7**

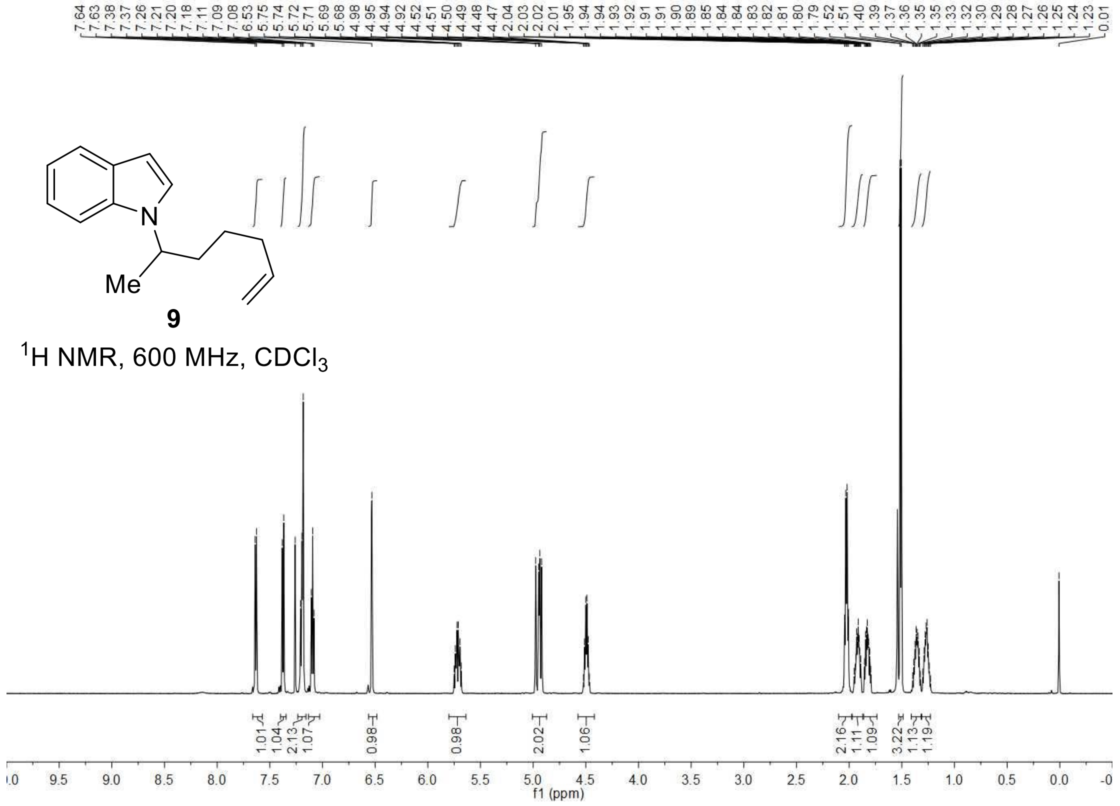

Supplementary Figure 195. ^{1}H NMR spectrum of compound **9**

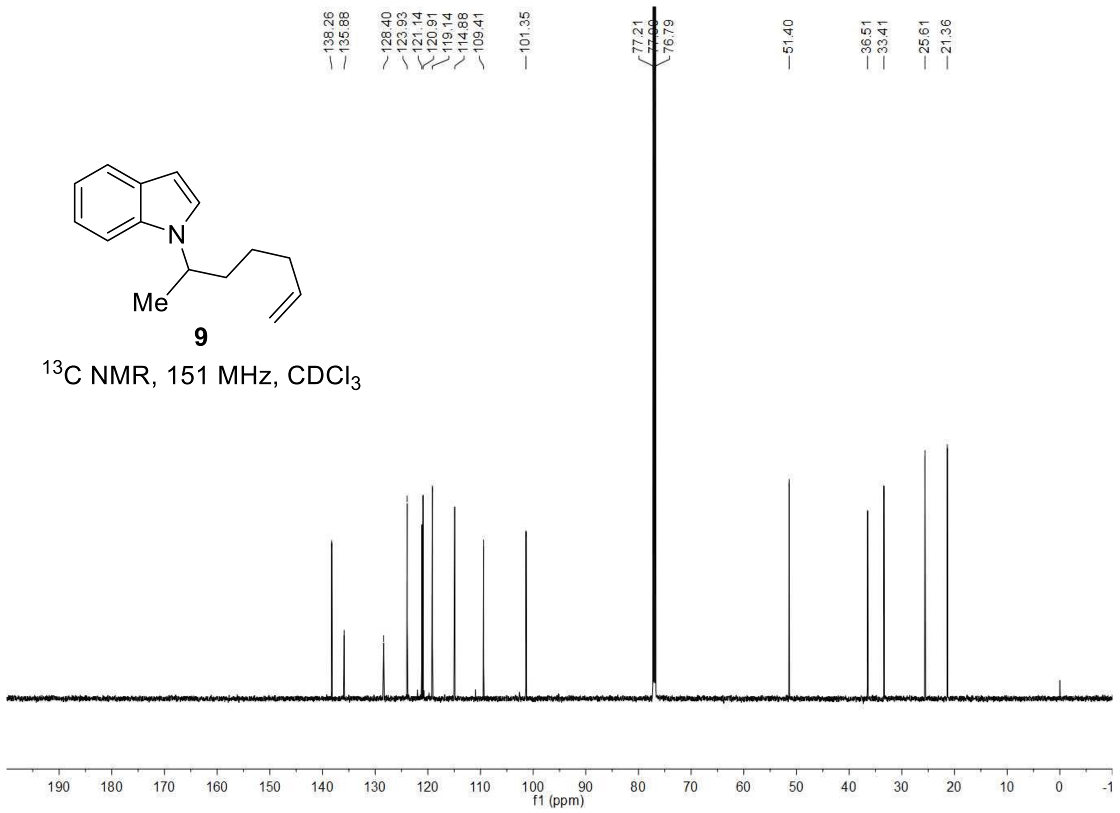

Supplementary Figure 196. ^{13}C NMR spectrum of compound **9**

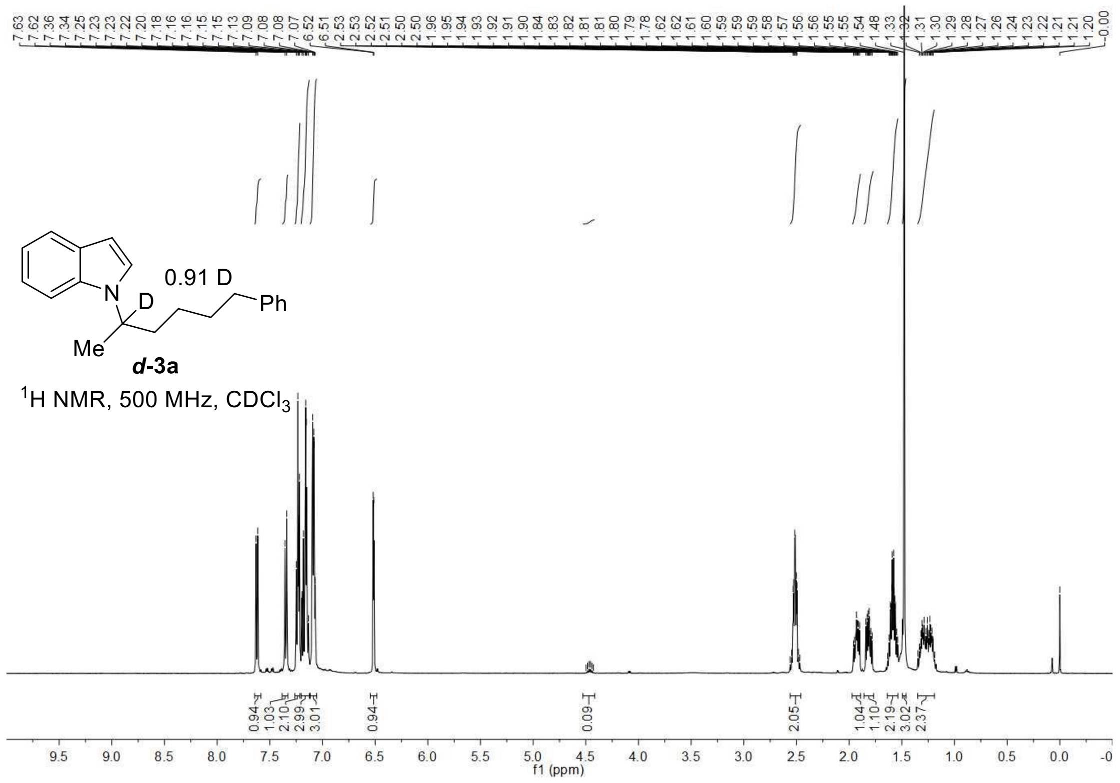

Supplementary Figure 197. ^{1}H NMR spectrum of compound ***d*-3a**

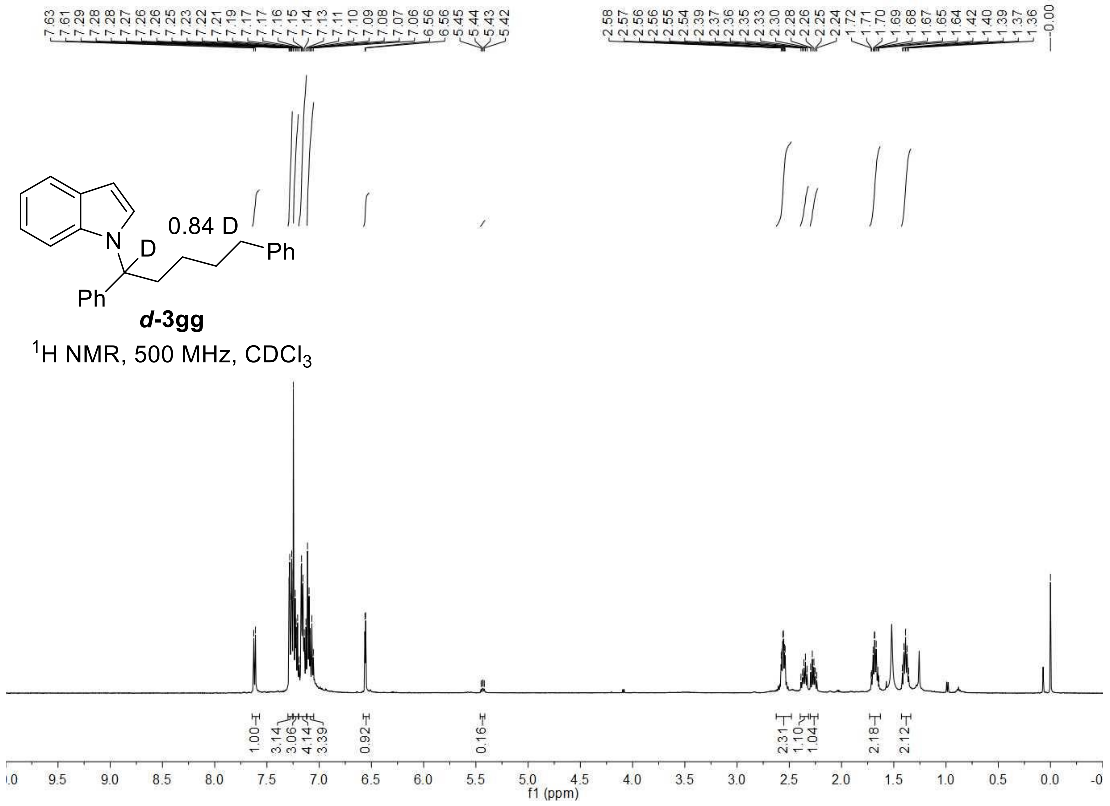

Supplementary Figure 198. ^{1}H NMR spectrum of compound ***d*-3gg**

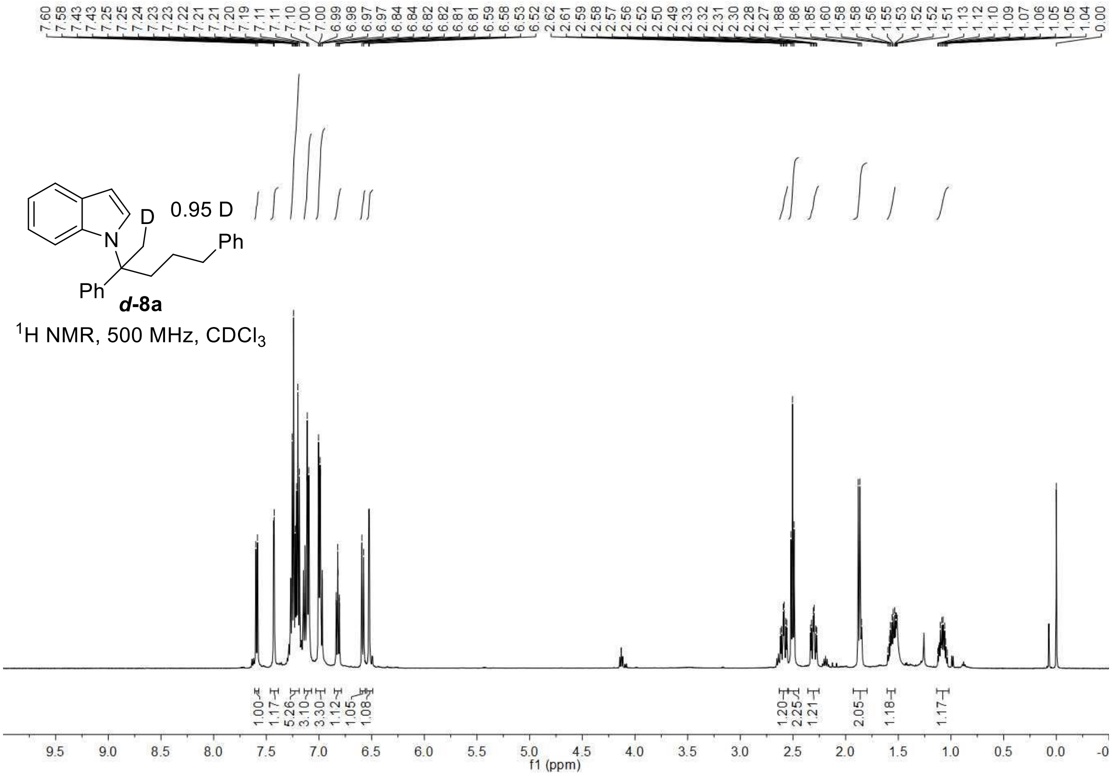

Supplementary Figure 199. ^{1}H NMR spectrum of compound ***d*-8a**

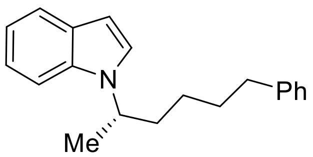

3a

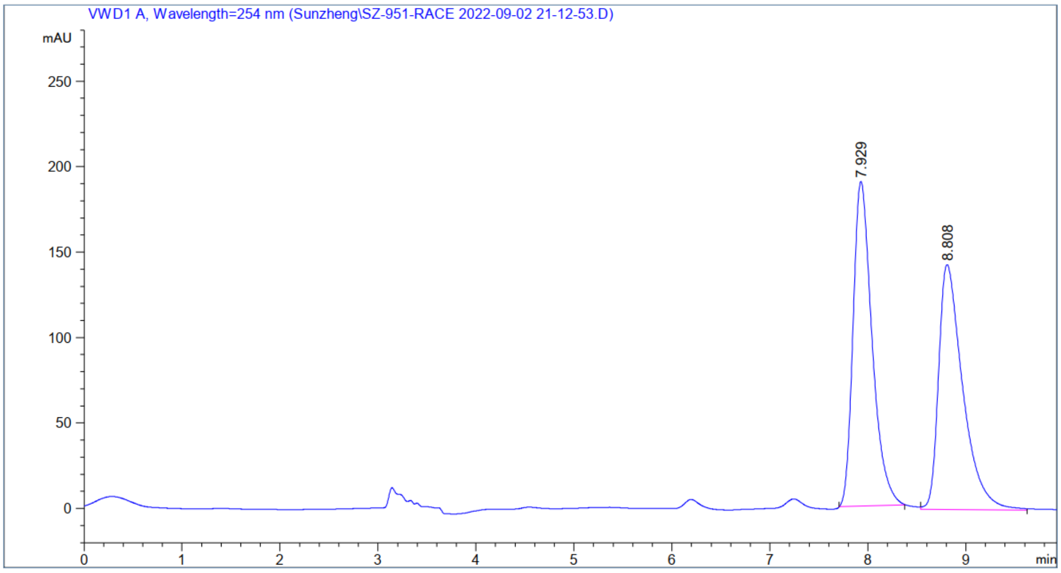

Peak #	RetTime [min]	Type	Width [min]	Area [mAU*s]	Height [mAU]	Area %
1	7.929	MM	0.2241	2553.13013	189.89369	50.9662
2	8.808	MM	0.2857	2456.33032	143.30707	49.0338

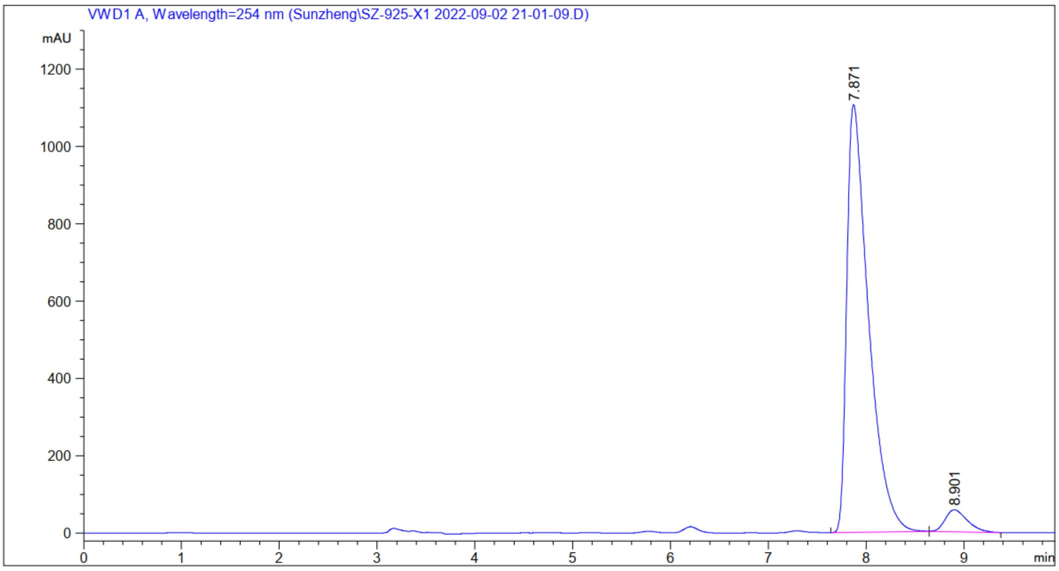

Peak #	RetTime [min]	Type	Width [min]	Area [mAU*s]	Height [mAU]	Area %
1	7.871	MM	0.2567	1.70411e4	1106.39331	94.9507
2	8.901	MM	0.2654	906.20825	56.91570	5.0493

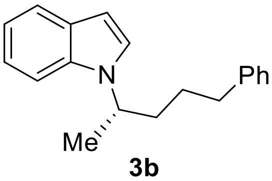

3b

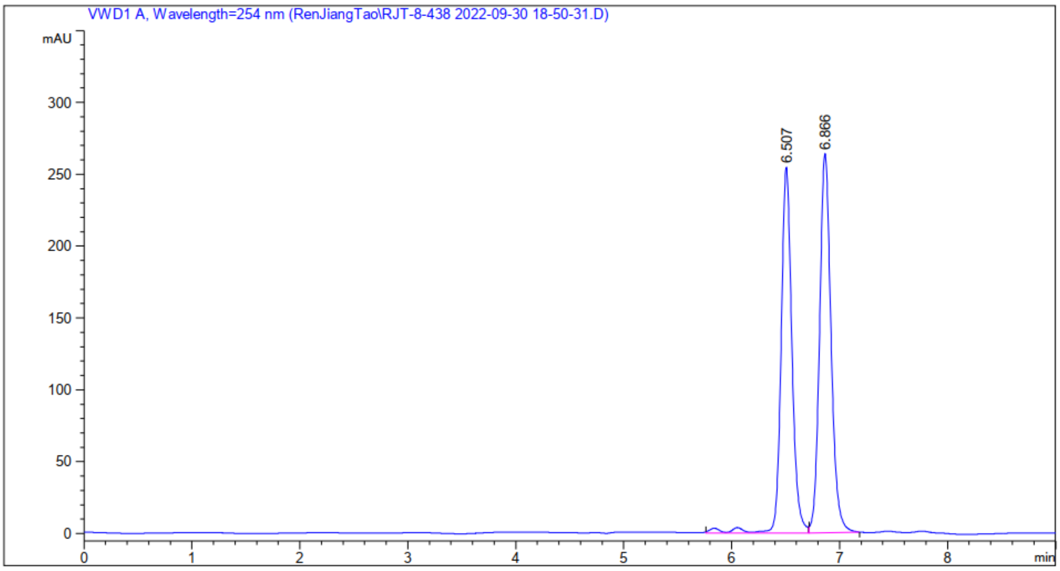

Peak #	RetTime [min]	Type	Width [min]	Area [mAU*s]	Height [mAU]	Area %
1	6.507	MM	0.1176	1796.63770	254.70244	48.0863
2	6.866	MM	0.1226	1939.64294	263.74515	51.9137

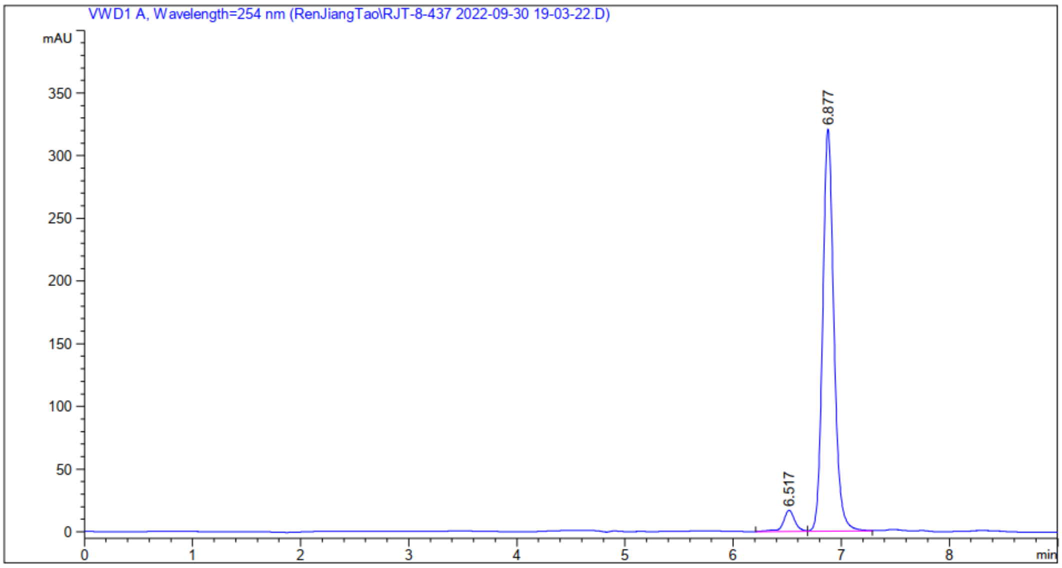

Peak #	RetTime [min]	Type	Width [min]	Area [mAU*s]	Height [mAU]	Area %
1	6.517	MF	0.1227	124.08311	16.85012	5.1038
2	6.877	FM	0.1200	2307.10815	320.47391	94.8962

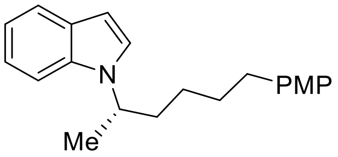

3c

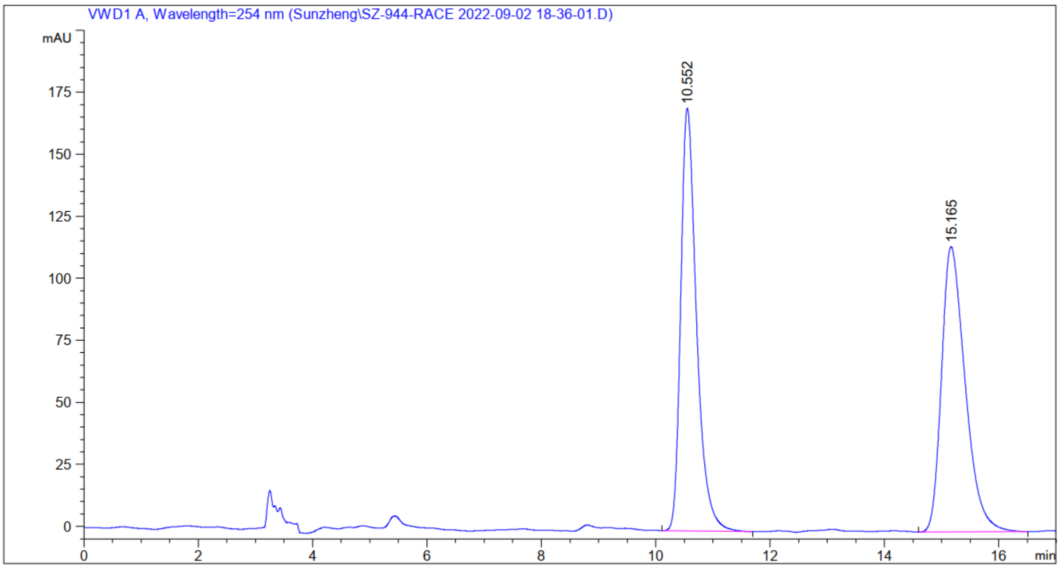

Peak #	RetTime [min]	Type	Width [min]	Area [mAU*s]	Height [mAU]	Area %
1	10.552	BB	0.2912	3275.62964	170.44365	49.7389
2	15.165	BB	0.4408	3310.02026	115.01452	50.2611

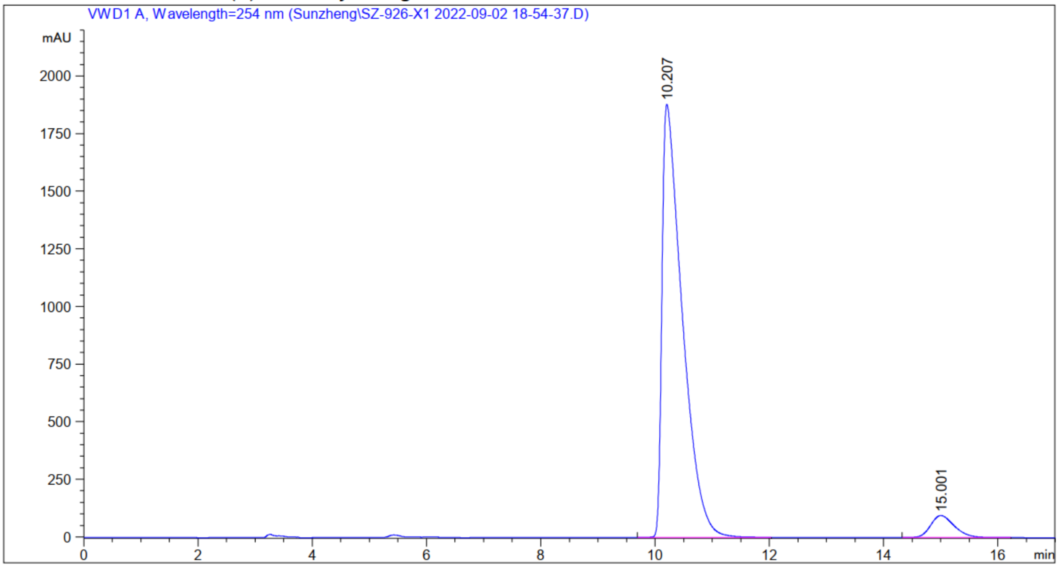

Peak #	RetTime [min]	Type	Width [min]	Area [mAU*s]	Height [mAU]	Area %
1	10.207	BB	0.3631	4.62388e4	1879.11938	94.3938
2	15.001	BB	0.4431	2746.22095	95.04294	5.6062

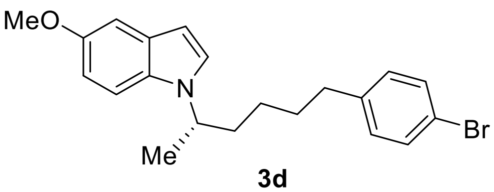

3d

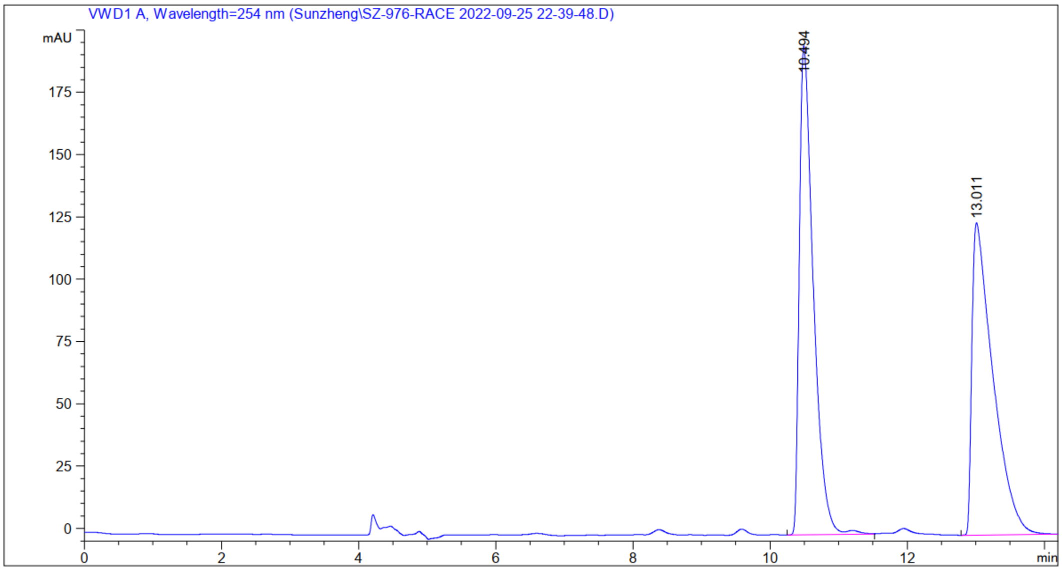

Peak #	RetTime [min]	Type	Width [min]	Area [mAU*s]	Height [mAU]	Area %
1	10.494	BV R	0.2141	2791.50977	196.03961	51.1283
2	13.011	BBA	0.3046	2668.30396	125.24434	48.8717

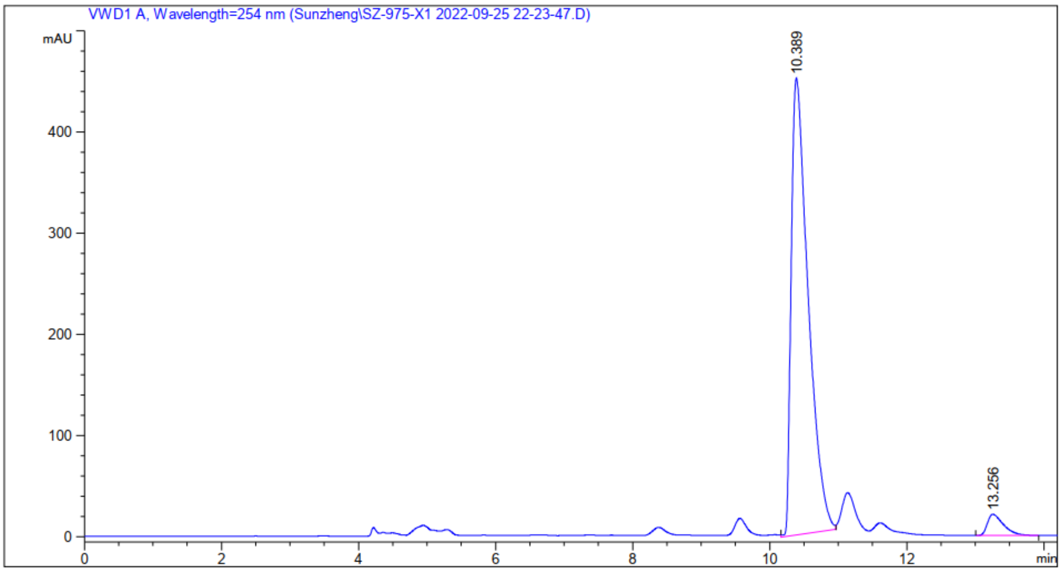

Peak #	RetTime [min]	Type	Width [min]	Area [mAU*s]	Height [mAU]	Area %
1	10.389	MM	0.2858	7740.77979	451.39859	95.7168
2	13.256	BB	0.2485	346.39185	20.99296	4.2832

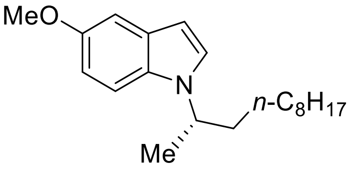

3e

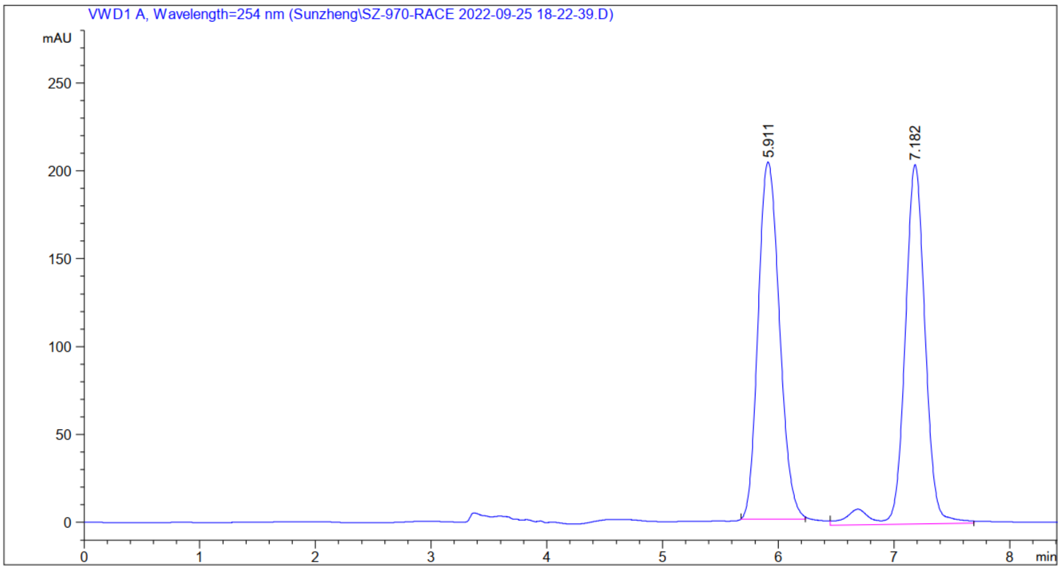

Peak #	RetTime [min]	Type	Width [min]	Area [mAU*s]	Height [mAU]	Area %
1	5.911	MM	0.2130	2595.19727	203.10980	50.4566
2	7.182	MM	0.2076	2548.23096	204.55931	49.5434

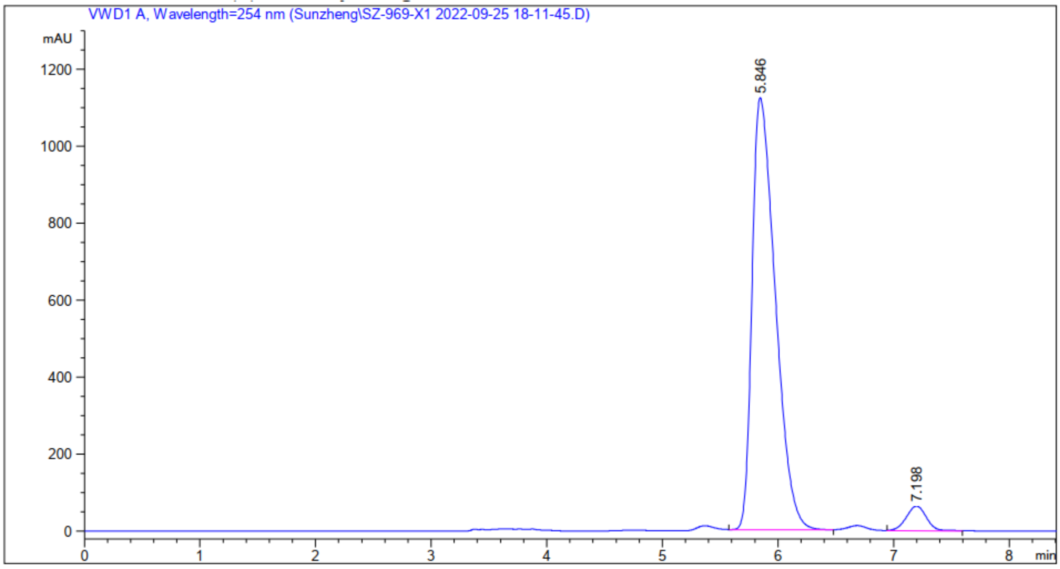

Peak #	RetTime [min]	Type	Width [min]	Area [mAU*s]	Height [mAU]	Area %
1	5.846	MM	0.2318	1.56042e4	1121.82422	95.2157
2	7.198	MM	0.2034	784.06525	64.25070	4.7843

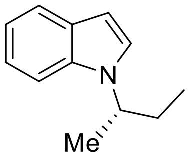

3f

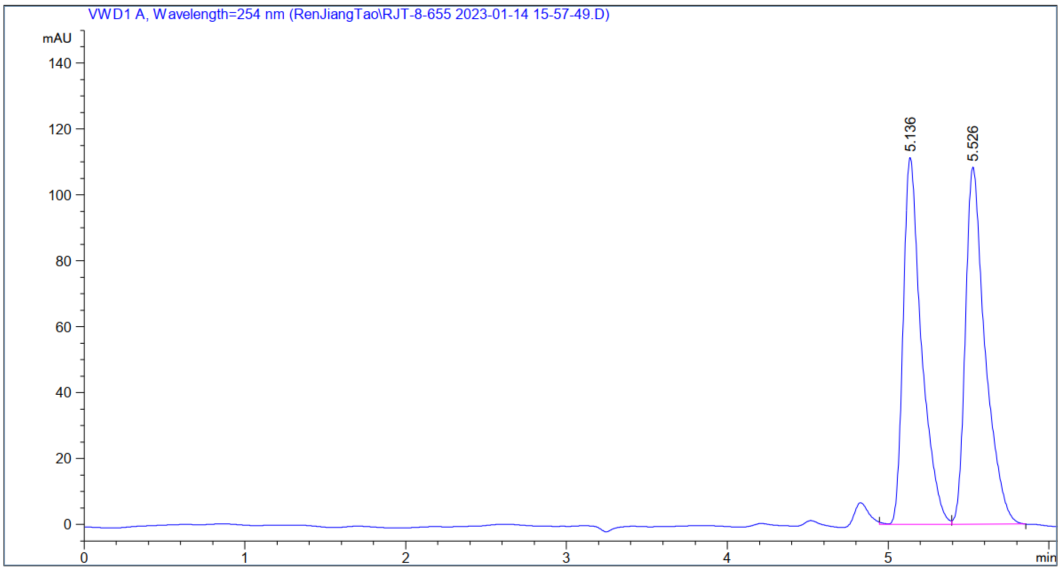

Peak #	RetTime [min]	Type	Width [min]	Area [mAU*s]	Height [mAU]	Area %
1	5.136	MF	0.1318	884.44324	111.83957	48.8130
2	5.526	FM	0.1420	927.45660	108.87420	51.1870

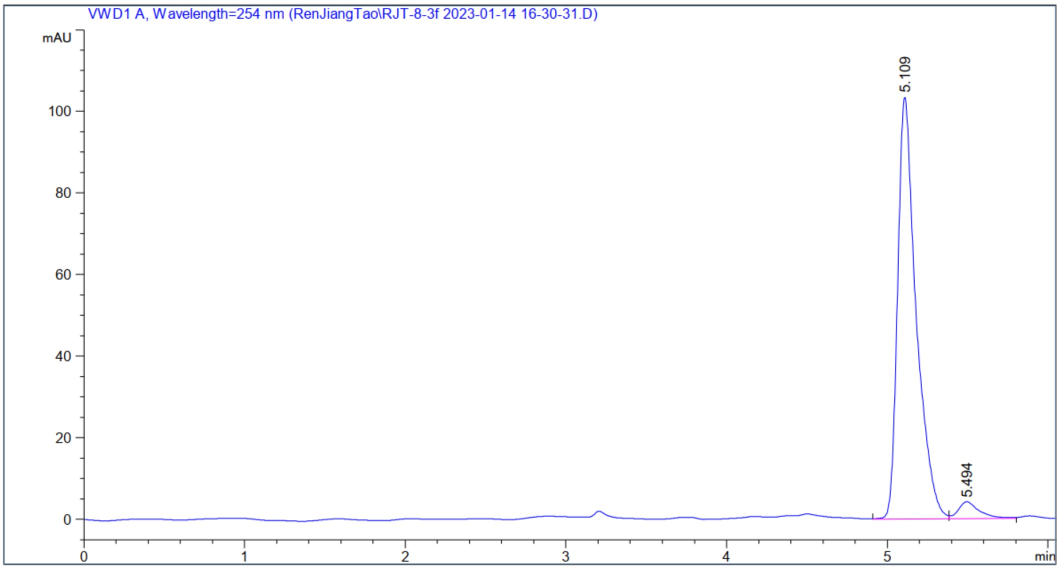

Peak #	RetTime [min]	Type	Width [min]	Area [mAU*s]	Height [mAU]	Area %
1	5.109	MF	0.1333	829.36237	103.67683	95.5560
2	5.494	FM	0.1536	38.57098	4.18610	4.4440

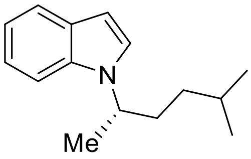

3g

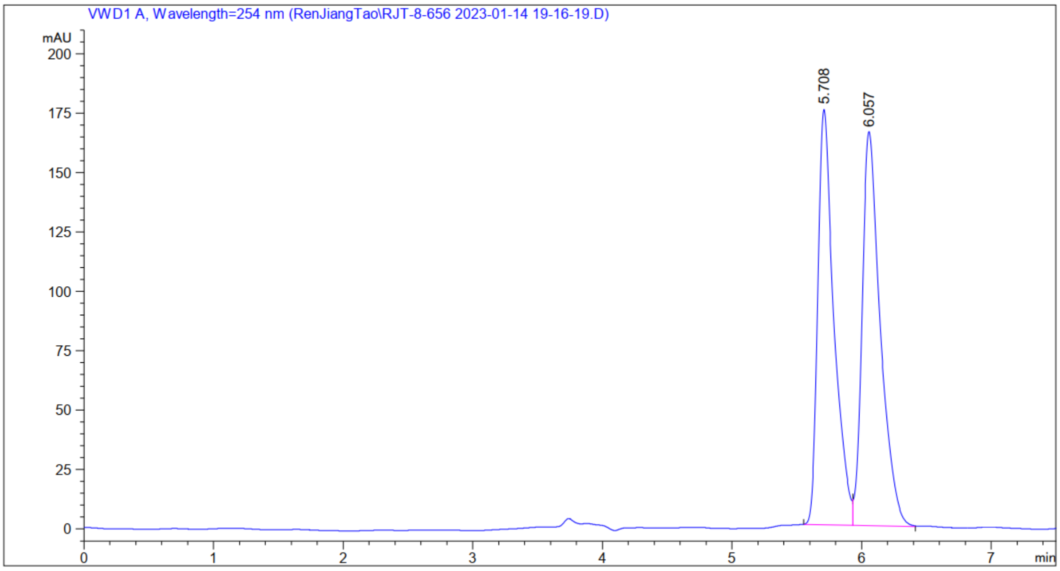

Peak #	RetTime [min]	Type	Width [min]	Area [mAU*s]	Height [mAU]	Area %
1	5.708	MF	0.1438	1513.86133	175.40381	48.3682
2	6.057	FM	0.1623	1616.00818	165.99625	51.6318

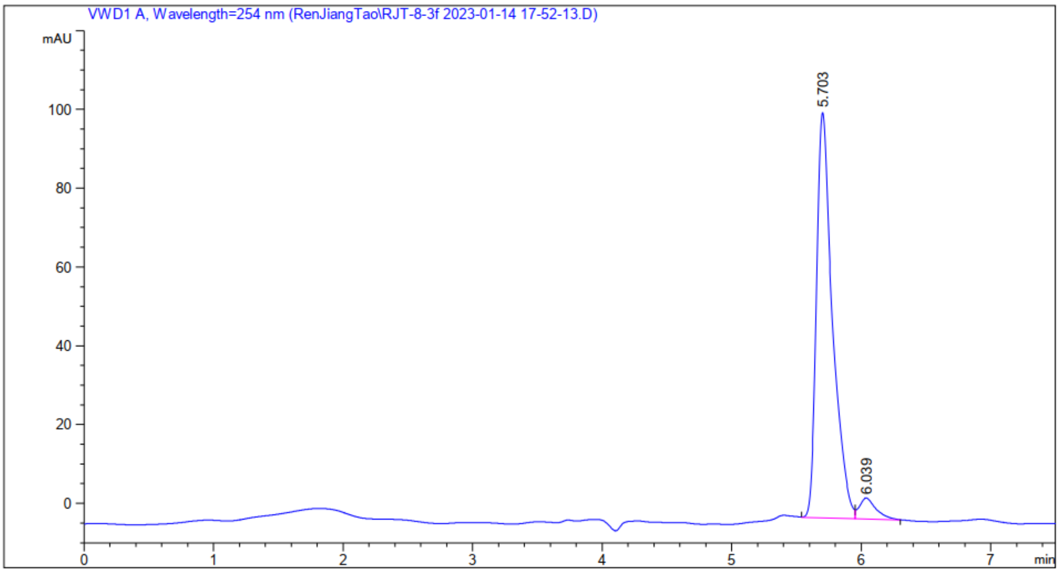

Peak #	RetTime [min]	Type	Width [min]	Area [mAU*s]	Height [mAU]	Area %
1	5.703	MF	0.1422	881.24072	103.31253	94.7621
2	6.039	FM	0.1500	48.70960	5.41234	5.2379

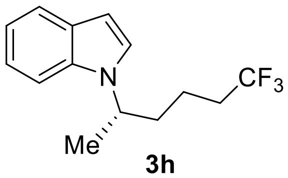

3h

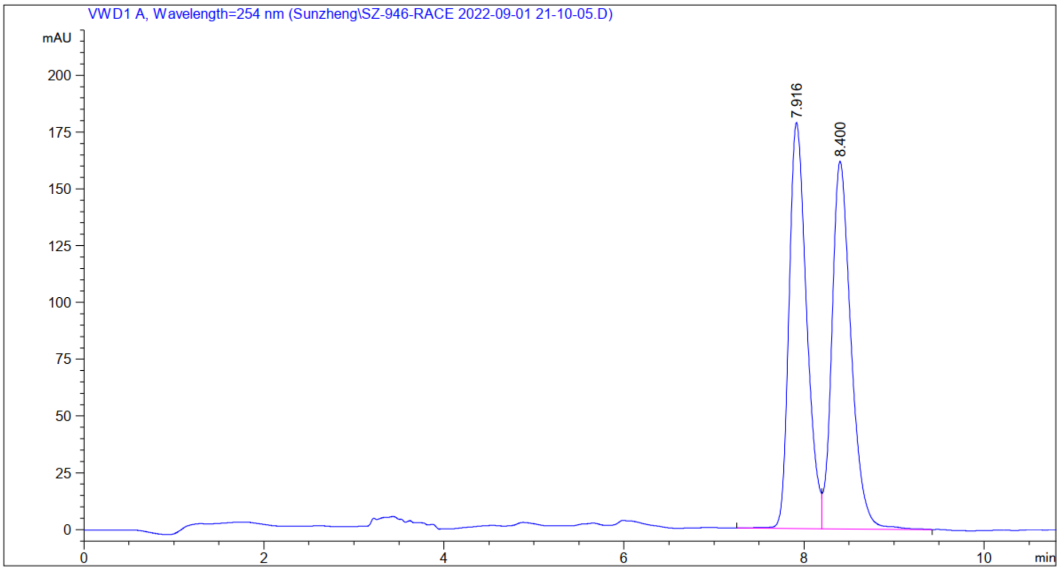

Peak #	RetTime [min]	Type	Width [min]	Area [mAU*s]	Height [mAU]	Area %
1	7.916	BV	0.2085	2427.25610	178.71056	50.4236
2	8.400	VB	0.2251	2386.47607	161.80252	49.5764

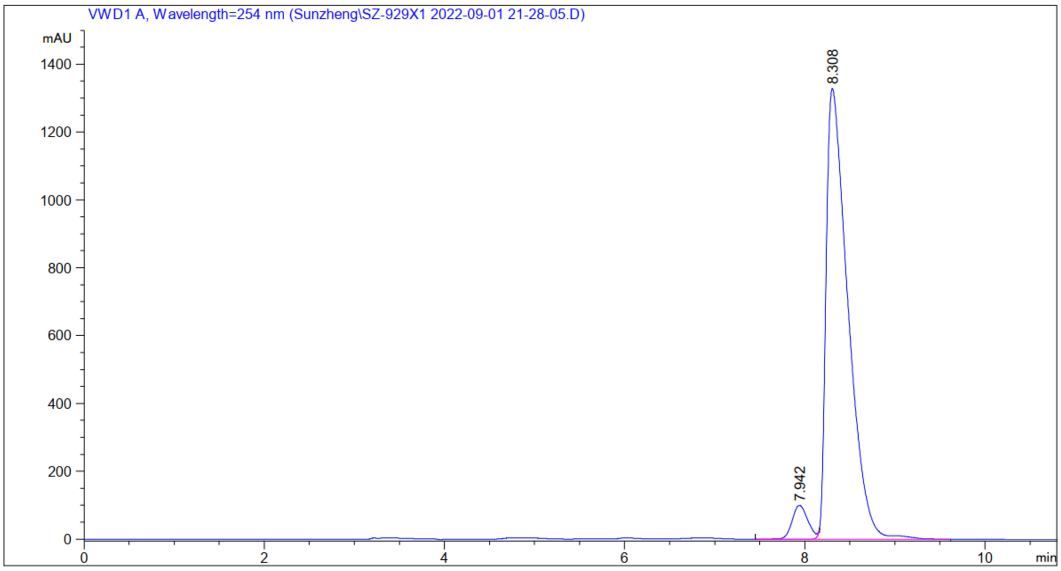

Peak #	RetTime [min]	Type	Width [min]	Area [mAU*s]	Height [mAU]	Area %
1	7.942	BV E	0.1876	1201.24121	99.64764	5.1131
2	8.308	VV R	0.2538	2.22920e4	1328.60522	94.8869

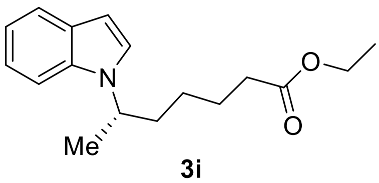

3i

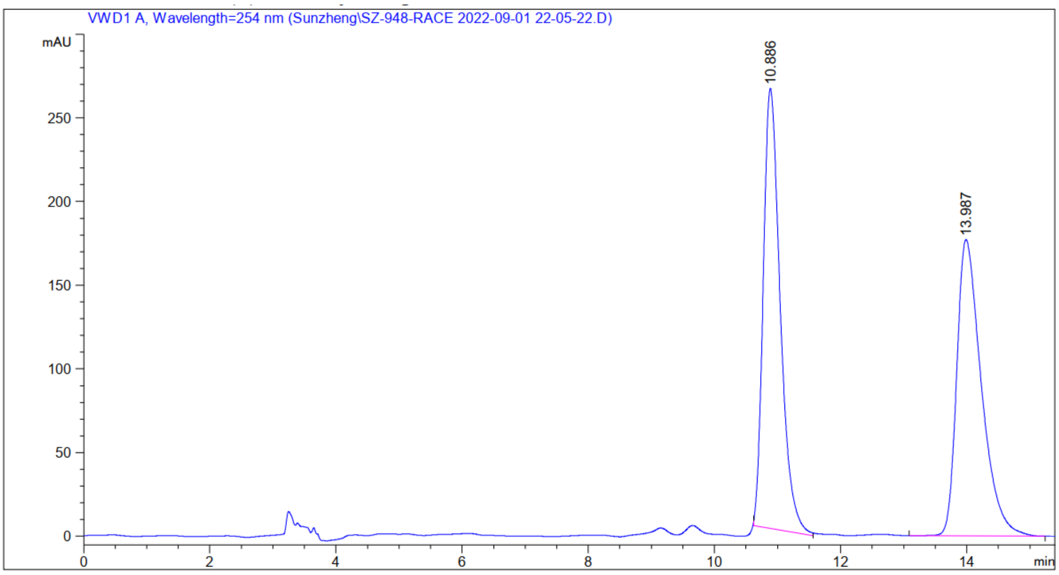

Peak #	RetTime [min]	Type	Width [min]	Area [mAU*s]	Height [mAU]	Area %
1	10.886	MM	0.3101	4890.82422	262.85818	50.4758
2	13.987	BB	0.4093	4798.61719	176.97885	49.5242

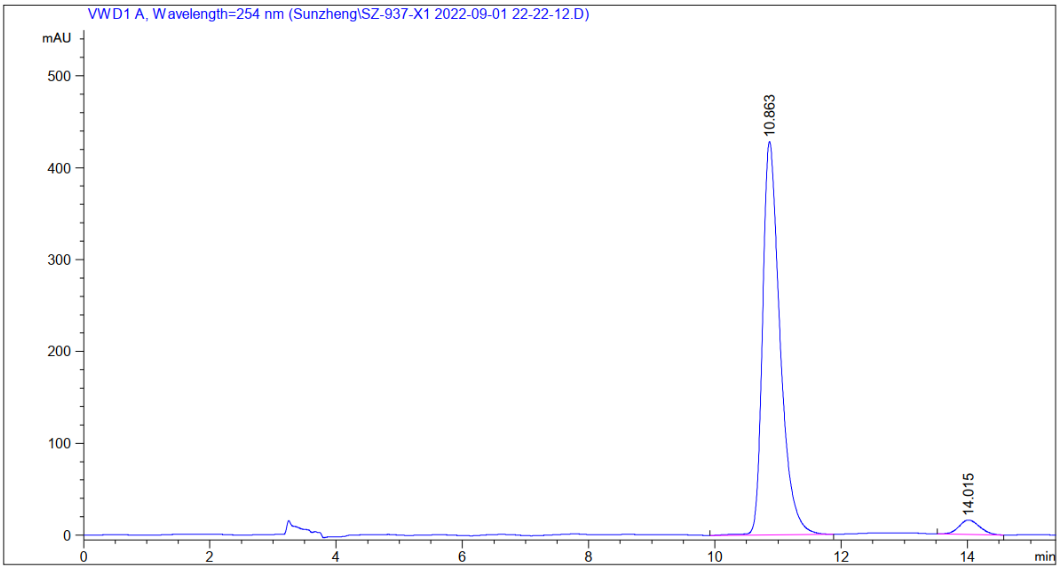

Peak #	RetTime [min]	Type	Width [min]	Area [mAU*s]	Height [mAU]	Area %
1	10.863	BB	0.2911	8225.51270	428.22110	95.9168
2	14.015	BB	0.3502	350.16135	15.51915	4.0832

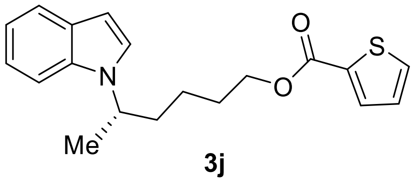

3j

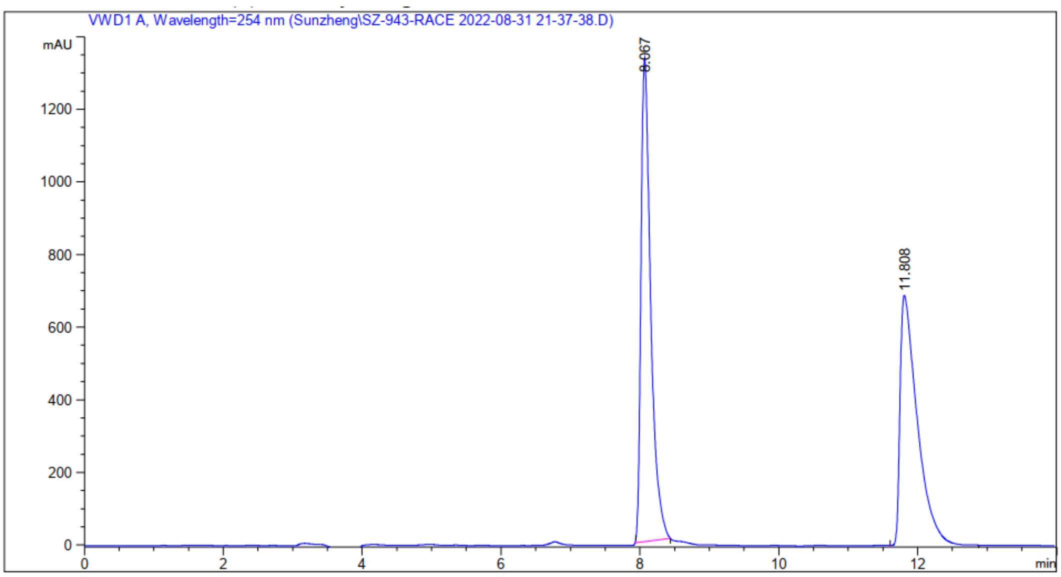

Peak #	RetTime [min]	Type	Width [min]	Area [mAU*s]	Height [mAU]	Area %
1	8.067	MM	0.1616	1.29061e4	1331.25671	50.9405
2	11.808	MM	0.2970	1.24295e4	697.41626	49.0595

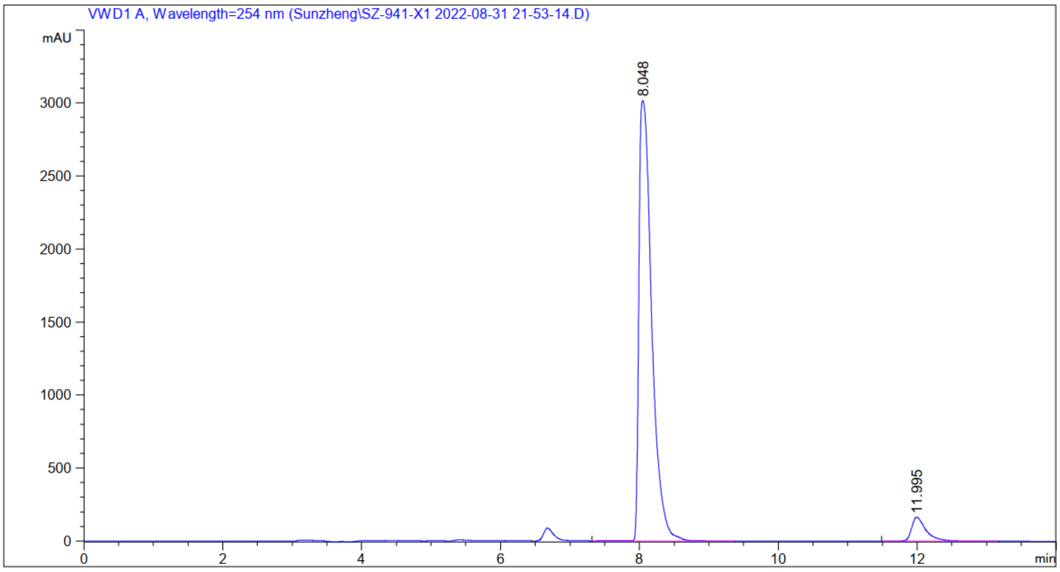

Peak #	RetTime [min]	Type	Width [min]	Area [mAU*s]	Height [mAU]	Area %
1	8.048	VV R	0.1973	3.86192e4	3015.17529	94.2911
2	11.995	BV R	0.2137	2338.20264	162.66753	5.7089

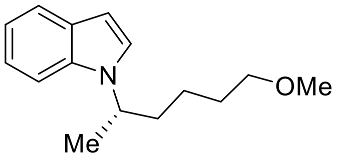

3k

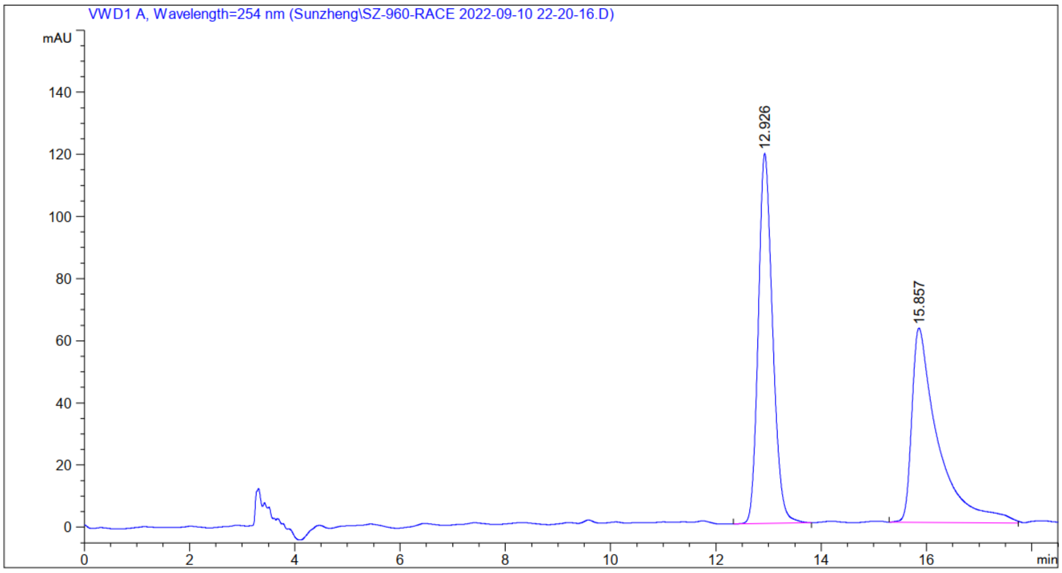

Peak #	RetTime [min]	Type	Width [min]	Area [mAU*s]	Height [mAU]	Area %
1	12.926	BB	0.2877	2252.41797	119.05137	50.9839
2	15.857	MF	0.5771	2165.48242	62.53680	49.0161

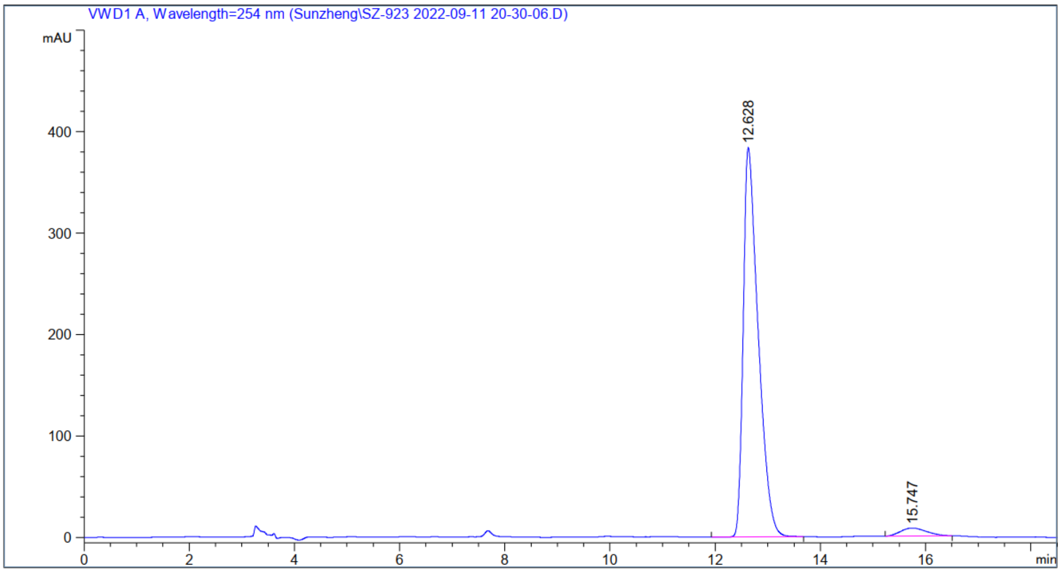

Peak #	RetTime [min]	Type	Width [min]	Area [mAU*s]	Height [mAU]	Area %
1	12.628	BB	0.2905	7575.05420	383.39832	96.4708
2	15.747	MM	0.5801	277.12155	7.96186	3.5292

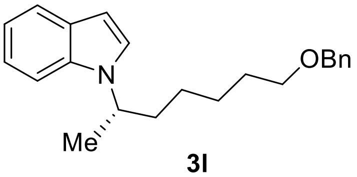

3l

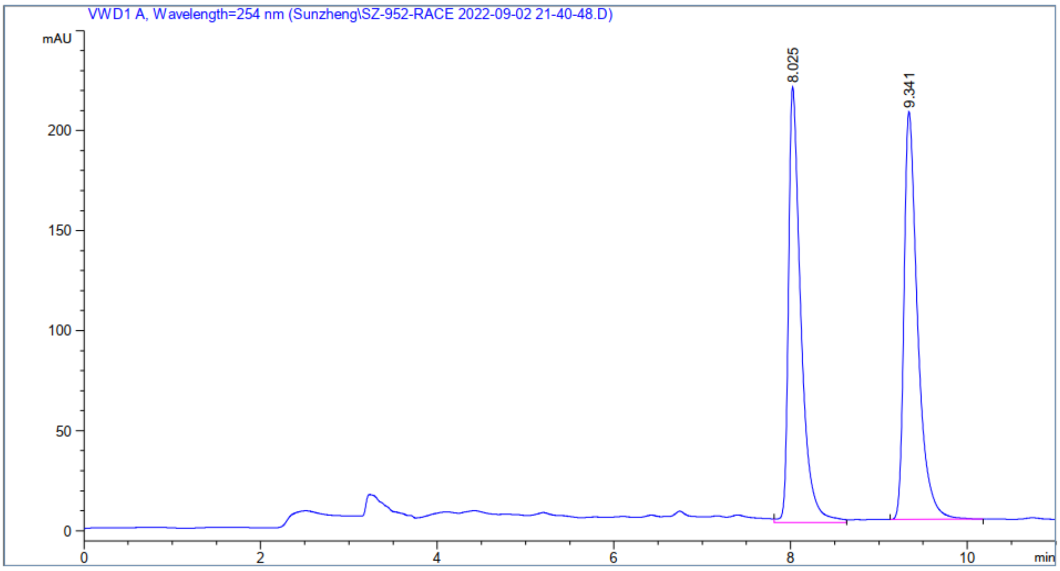

Peak #	RetTime [min]	Type	Width [min]	Area [mAU*s]	Height [mAU]	Area %
1	8.025	MM	0.1626	2126.65479	217.95262	49.3366
2	9.341	BB	0.1589	2183.84473	203.92470	50.6634

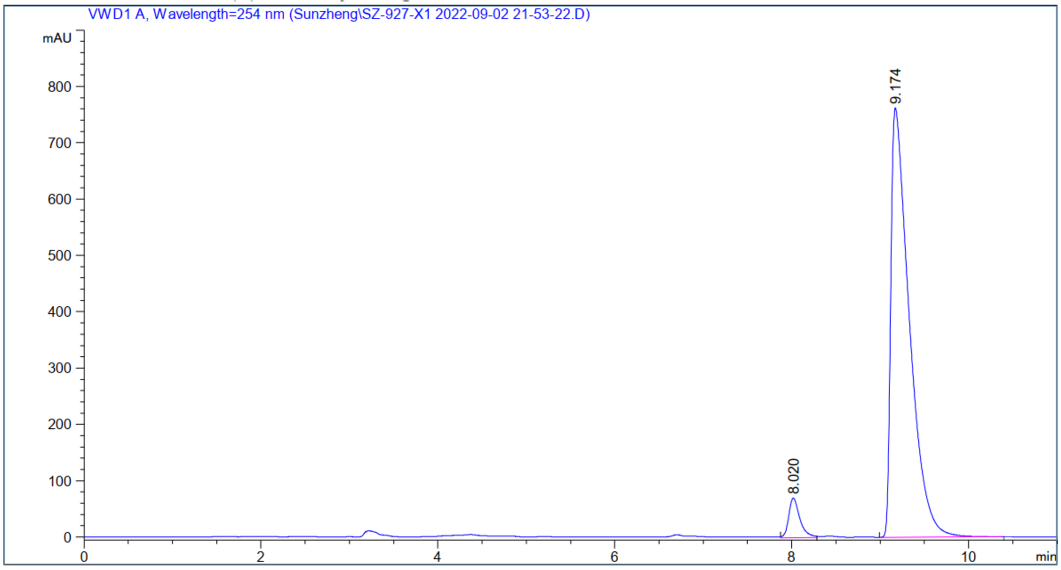

Peak #	RetTime [min]	Type	Width [min]	Area [mAU*s]	Height [mAU]	Area %
1	8.020	MM	0.1455	614.33301	70.37986	5.2413
2	9.174	BB	0.2170	1.11068e4	762.29926	94.7587

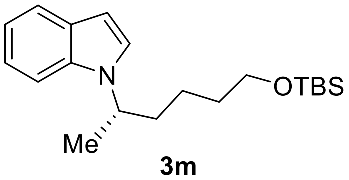

3m

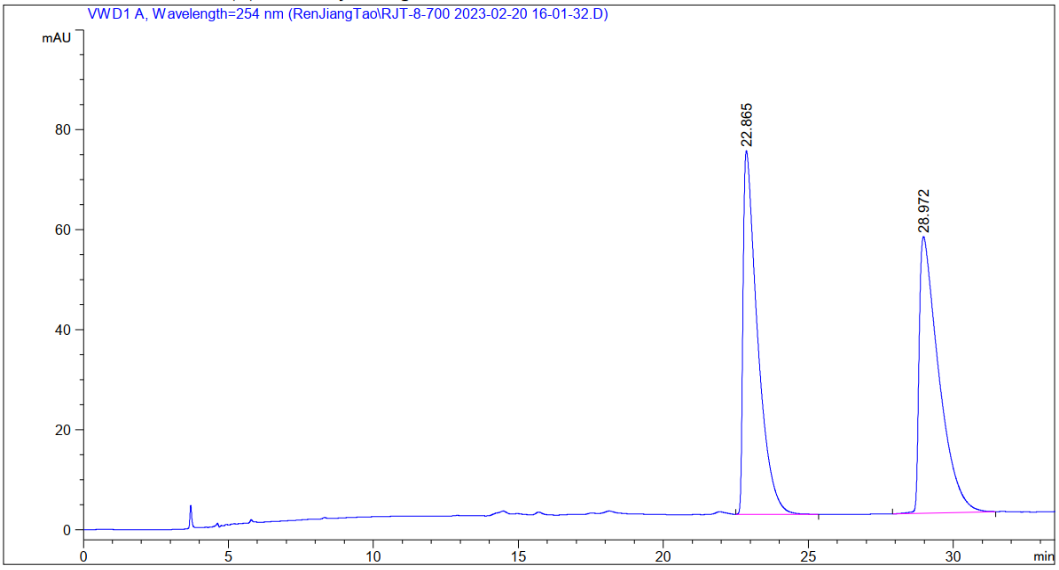

Peak #	RetTime [min]	Type	Width [min]	Area [mAU*s]	Height [mAU]	Area %
1	22.865	BB	0.5044	2522.62280	72.76852	49.2429
2	28.972	BB	0.6759	2600.18872	55.36870	50.7571

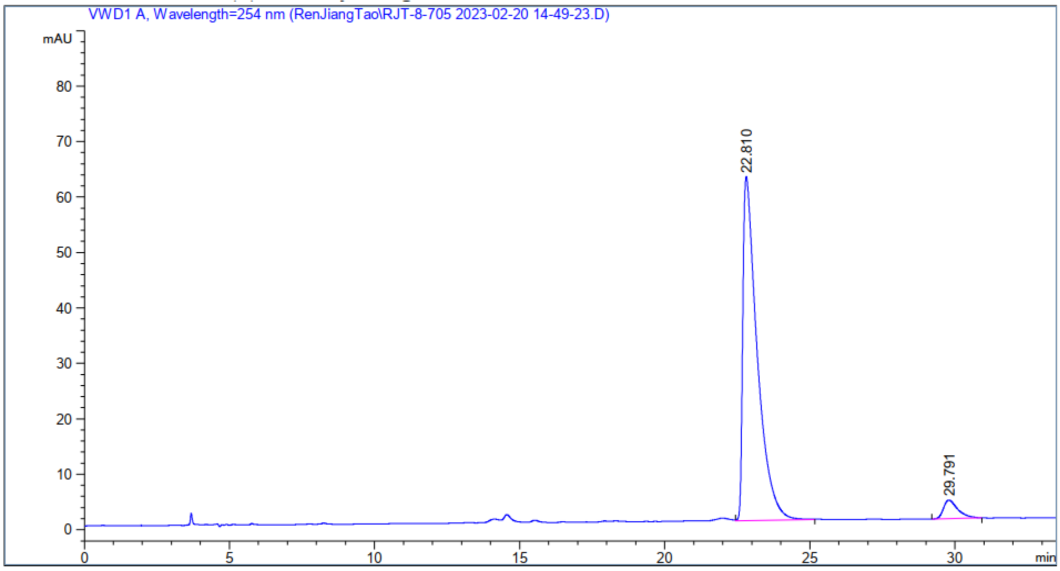

Peak #	RetTime [min]	Type	Width [min]	Area [mAU*s]	Height [mAU]	Area %
1	22.810	MF	0.5751	2140.27026	62.02501	94.8337
2	29.791	MM	0.5772	116.59647	3.36700	5.1663

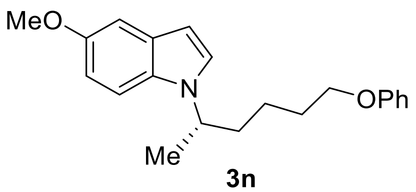

3n

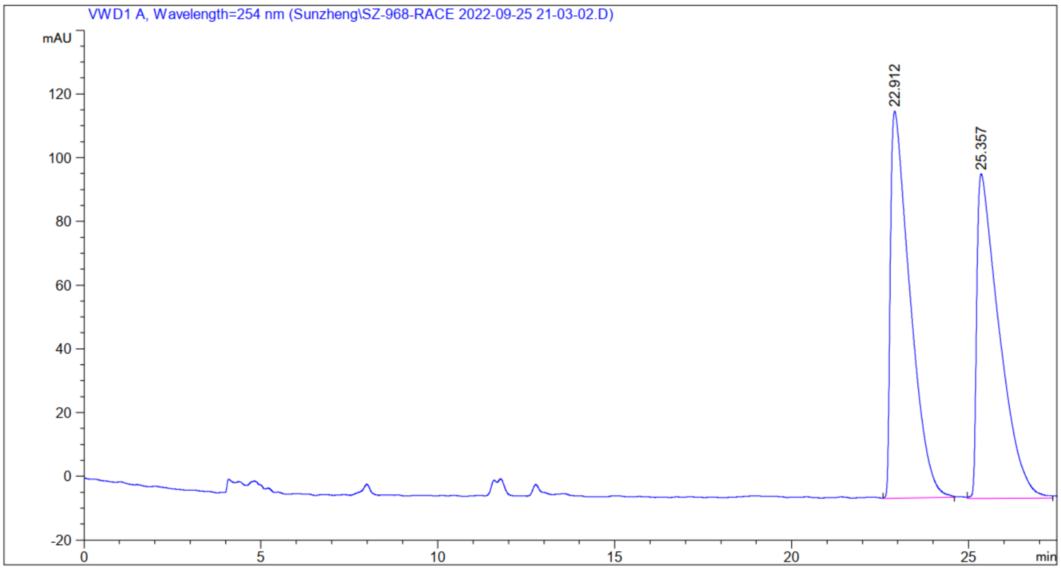

Peak #	RetTime [min]	Type	Width [min]	Area [mAU*s]	Height [mAU]	Area %
1	22.912	MM	0.6417	4677.80371	121.49873	50.9551
2	25.357	MM	0.7366	4502.43994	101.87770	49.0449

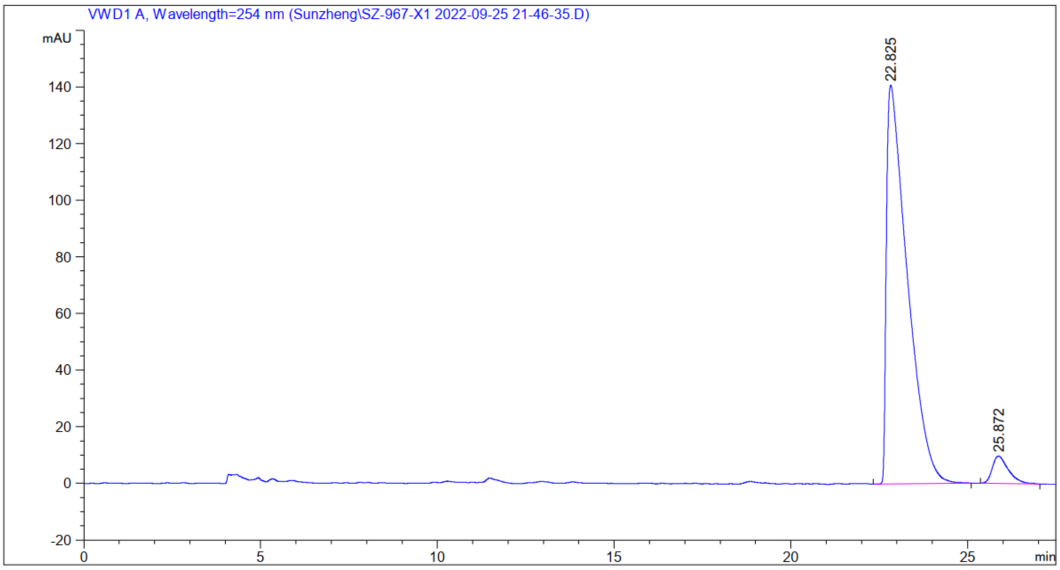

Peak #	RetTime [min]	Type	Width [min]	Area [mAU*s]	Height [mAU]	Area %
1	22.825	BB	0.6055	5763.53906	140.82852	94.9479
2	25.872	BB	0.4711	306.67609	9.64098	5.0521

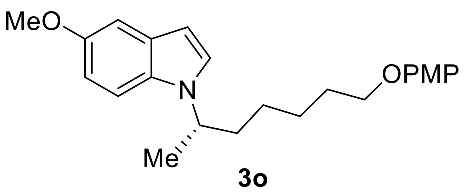

3o

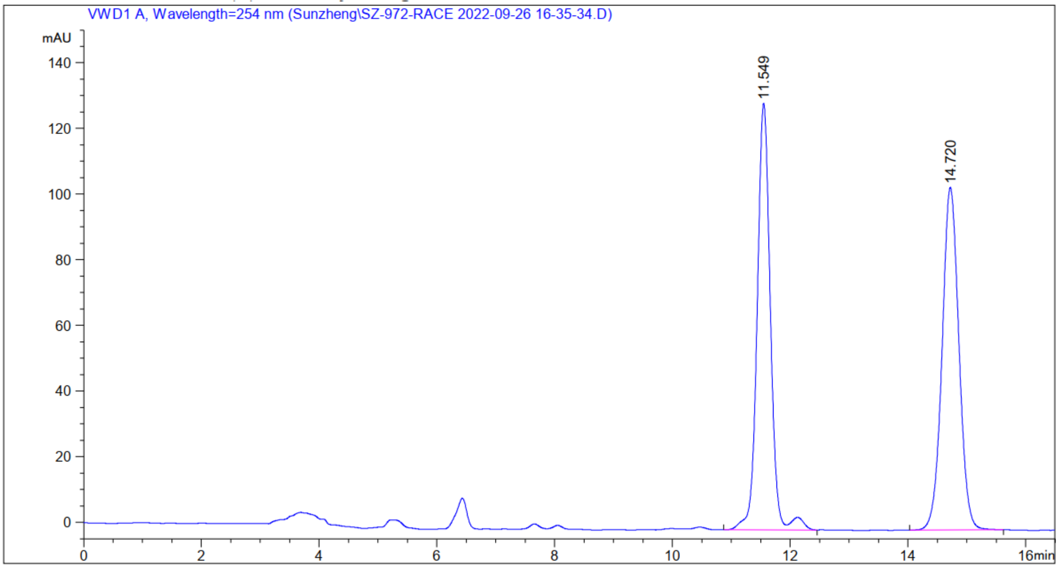

Peak #	RetTime [min]	Type	Width [min]	Area [mAU*s]	Height [mAU]	Area %
1	11.549	BV R	0.2340	2041.39587	130.01230	49.5647
2	14.720	BB	0.3072	2077.25488	104.37664	50.4353

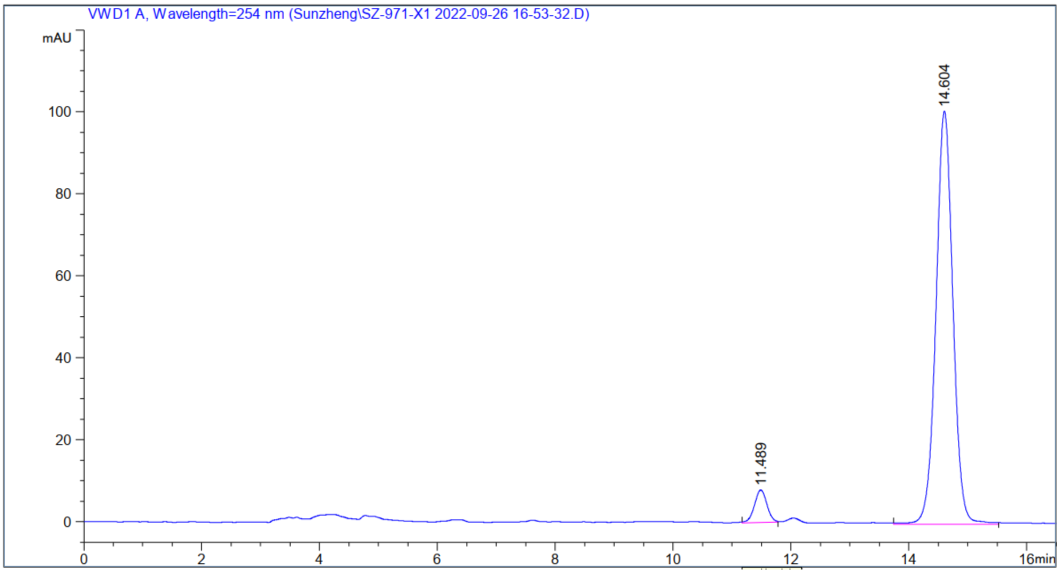

Peak #	RetTime [min]	Type	Width [min]	Area [mAU*s]	Height [mAU]	Area %
1	11.489	MM	0.2453	116.59241	7.92293	5.4599
2	14.604	MM	0.3338	2018.85535	100.79952	94.5401

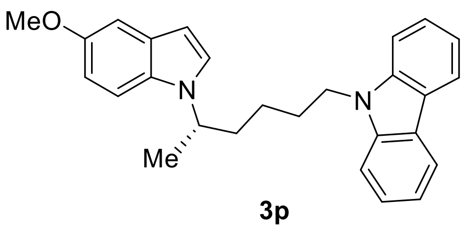

3p

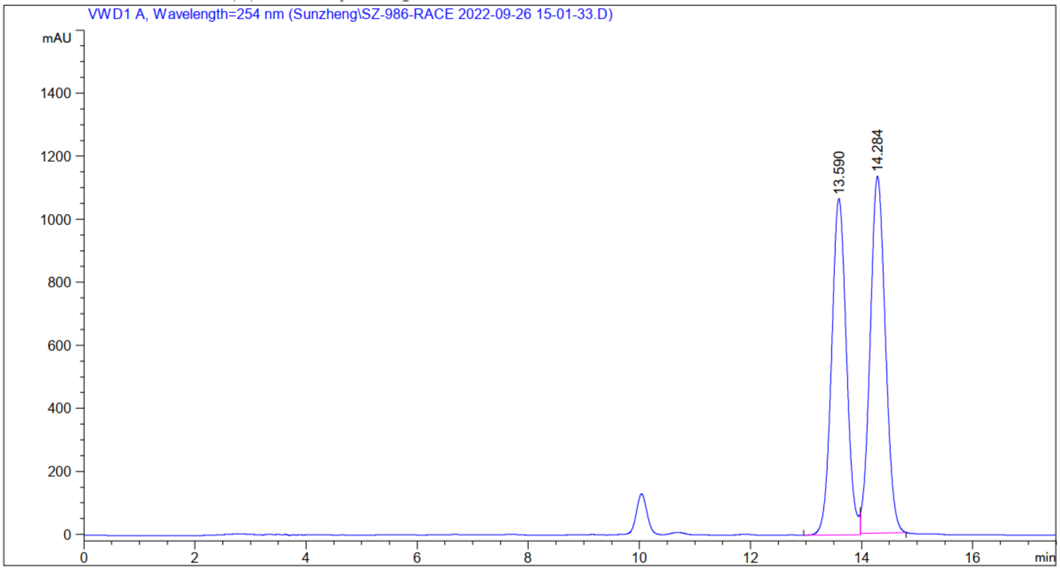

Peak #	RetTime [min]	Type	Width [min]	Area [mAU*s]	Height [mAU]	Area %
1	13.590	MM	0.3101	1.98566e4	1067.22754	48.0138
2	14.284	MM	0.3165	2.14994e4	1132.02893	51.9862

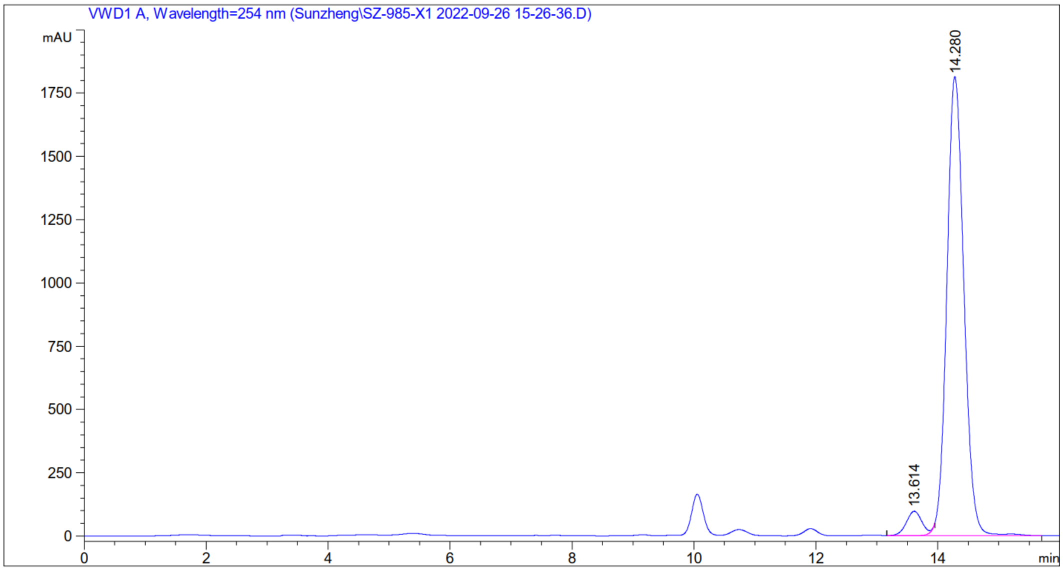

Peak #	RetTime [min]	Type	Width [min]	Area [mAU*s]	Height [mAU]	Area %
1	13.614	BV E	0.2734	1695.82581	96.33540	4.5743
2	14.280	VV R	0.3016	3.53767e4	1813.79968	95.4257

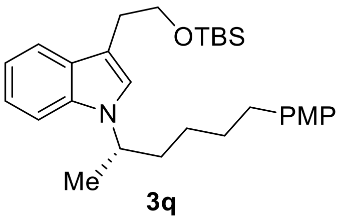

3q

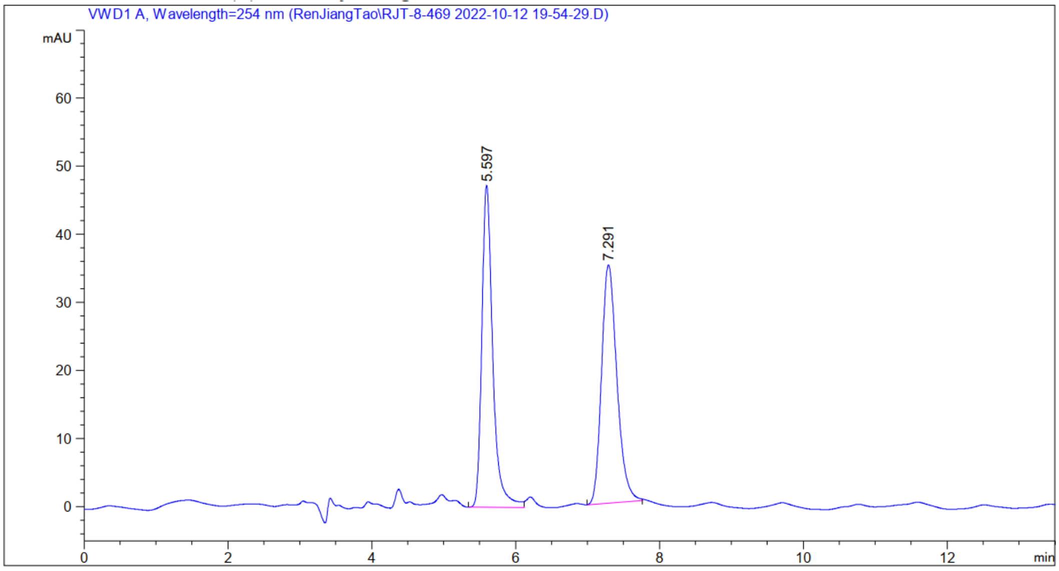

Peak #	RetTime [min]	Type	Width [min]	Area [mAU*s]	Height [mAU]	Area %
1	5.597	MF	0.1763	500.34927	47.30347	49.0550
2	7.291	MM	0.2472	519.62714	35.04106	50.9450

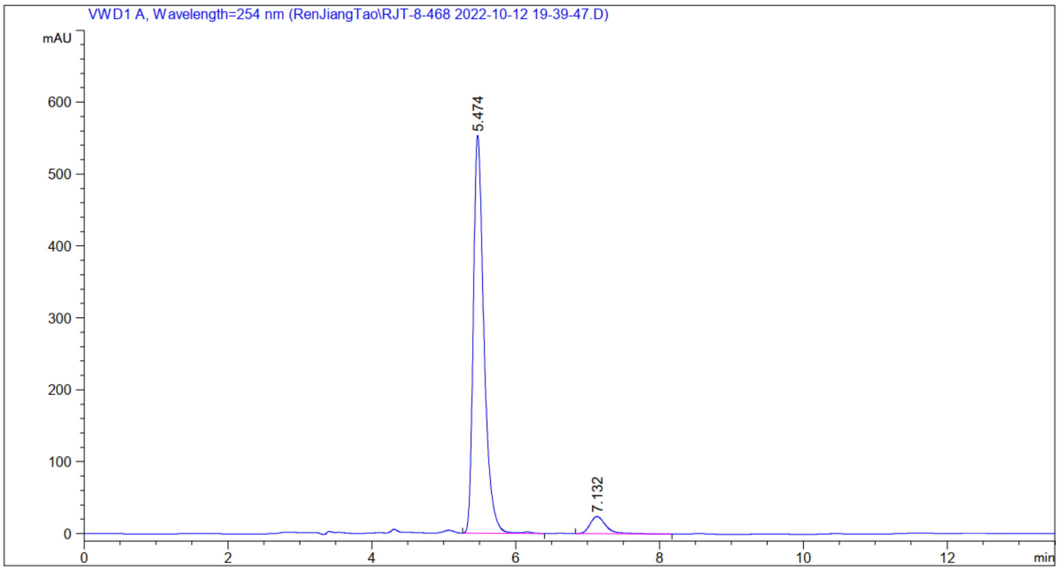

Peak #	RetTime [min]	Type	Width [min]	Area [mAU*s]	Height [mAU]	Area %
1	5.474	BV R	0.1547	5692.96143	553.48779	93.8613
2	7.132	BB	0.2361	372.33130	23.98186	6.1387

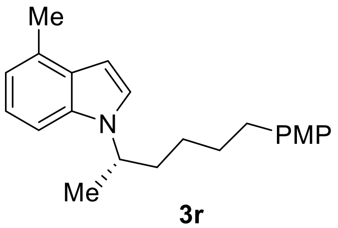

3r

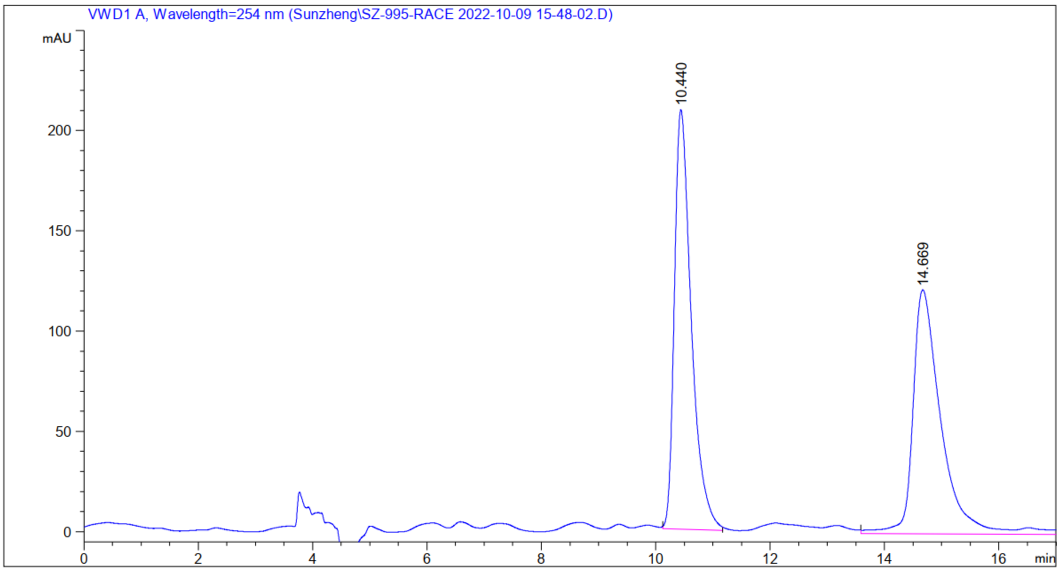

Peak #	RetTime [min]	Type	Width [min]	Area [mAU*s]	Height [mAU]	Area %
1	10.440	MM	0.3424	4296.10938	209.12242	50.2866
2	14.669	MM	0.5815	4247.13818	121.72350	49.7134

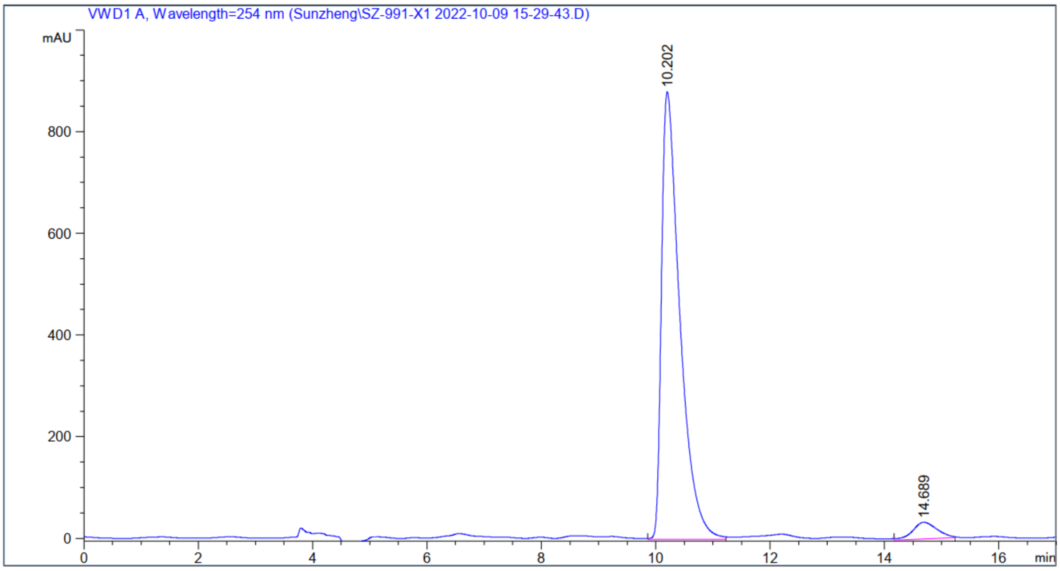

Peak #	RetTime [min]	Type	Width [min]	Area [mAU*s]	Height [mAU]	Area %
1	10.202	MM	0.3715	1.96167e4	880.02393	95.2426
2	14.689	MM	0.4972	979.86157	32.84682	4.7574

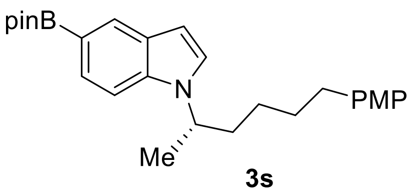

3s

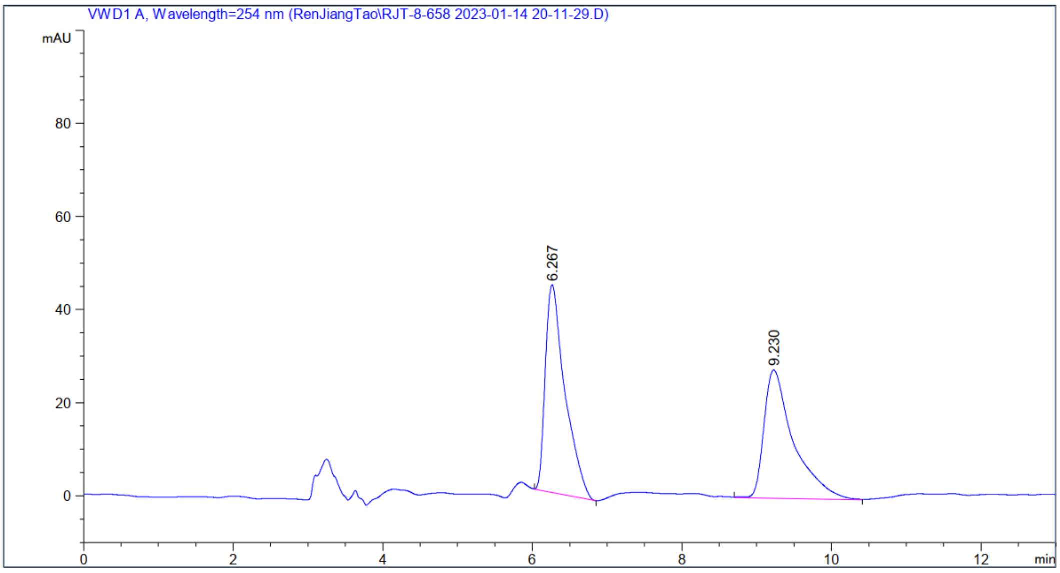

Peak #	RetTime [min]	Type	Width [min]	Area [mAU*s]	Height [mAU]	Area %
1	6.267	MM	0.3044	815.32617	44.63565	51.9194
2	9.230	MM	0.4567	755.04333	27.55400	48.0806

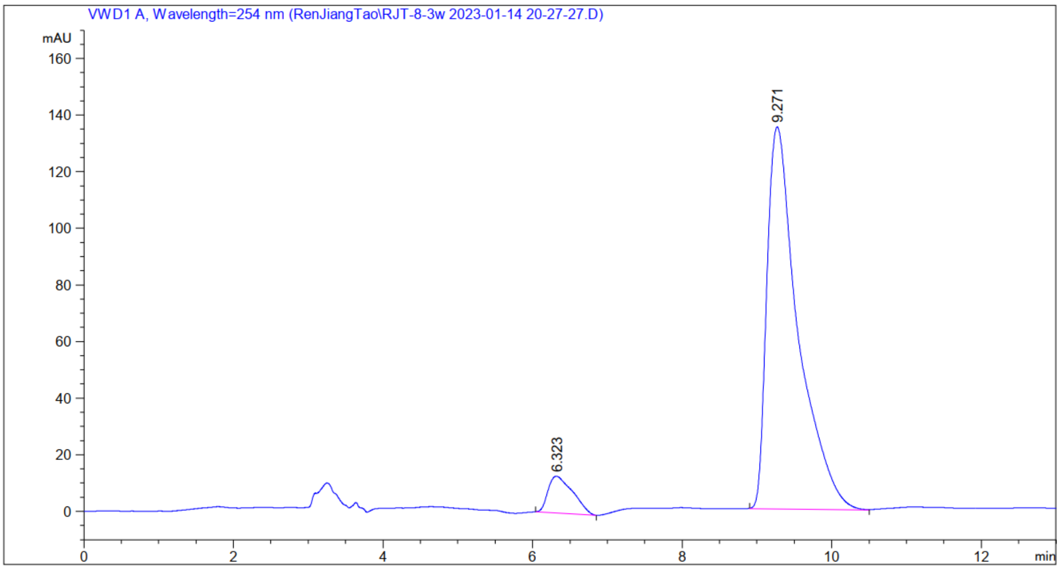

Peak #	RetTime [min]	Type	Width [min]	Area [mAU*s]	Height [mAU]	Area %
1	6.323	MM	0.3833	299.03912	13.00371	7.1068
2	9.271	MM	0.4823	3908.76685	135.06999	92.8932

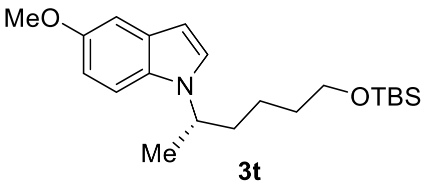

3t

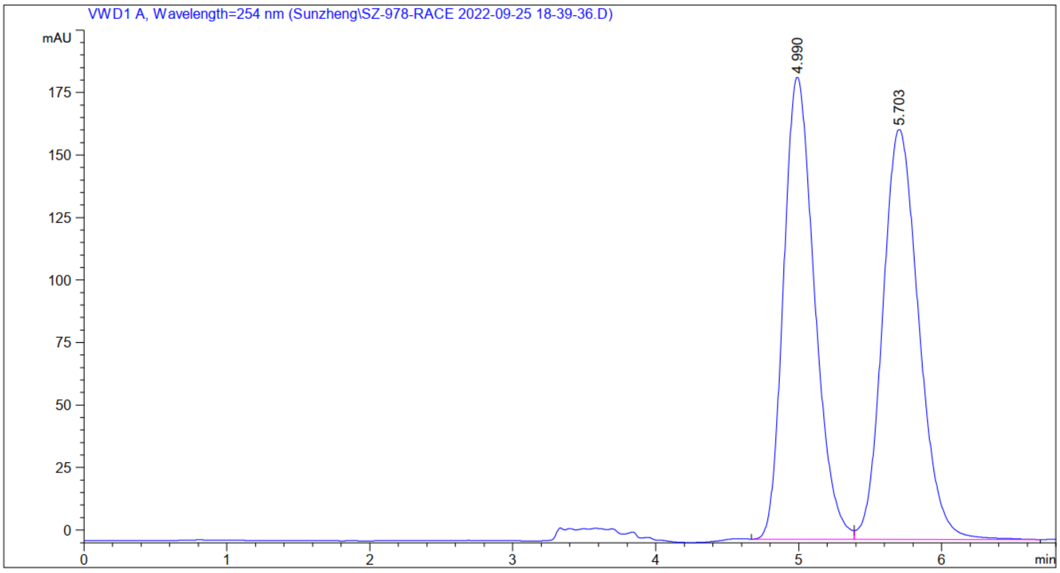

Peak #	RetTime [min]	Type	Width [min]	Area [mAU*s]	Height [mAU]	Area %
1	4.990	BV	0.2325	2779.17334	184.74200	49.1708
2	5.703	VB	0.2724	2872.91309	163.98781	50.8292

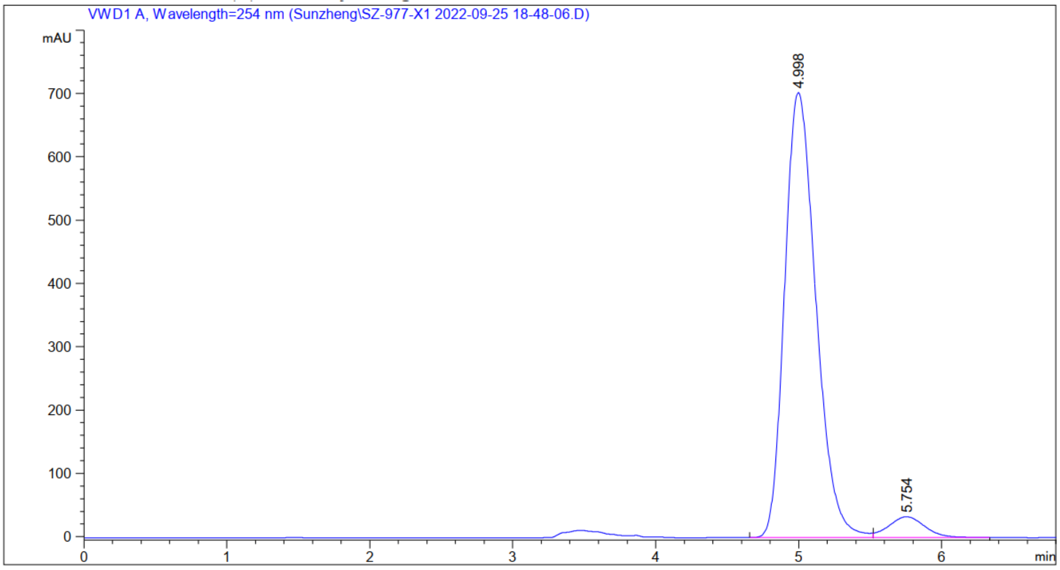

Peak #	RetTime [min]	Type	Width [min]	Area [mAU*s]	Height [mAU]	Area %
1	4.998	MF	0.2533	1.06763e4	702.47797	94.9360
2	5.754	FM	0.2900	569.49072	32.72439	5.0640

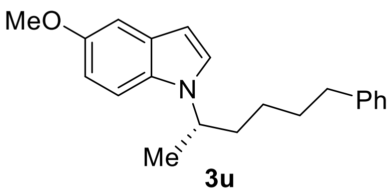

3u

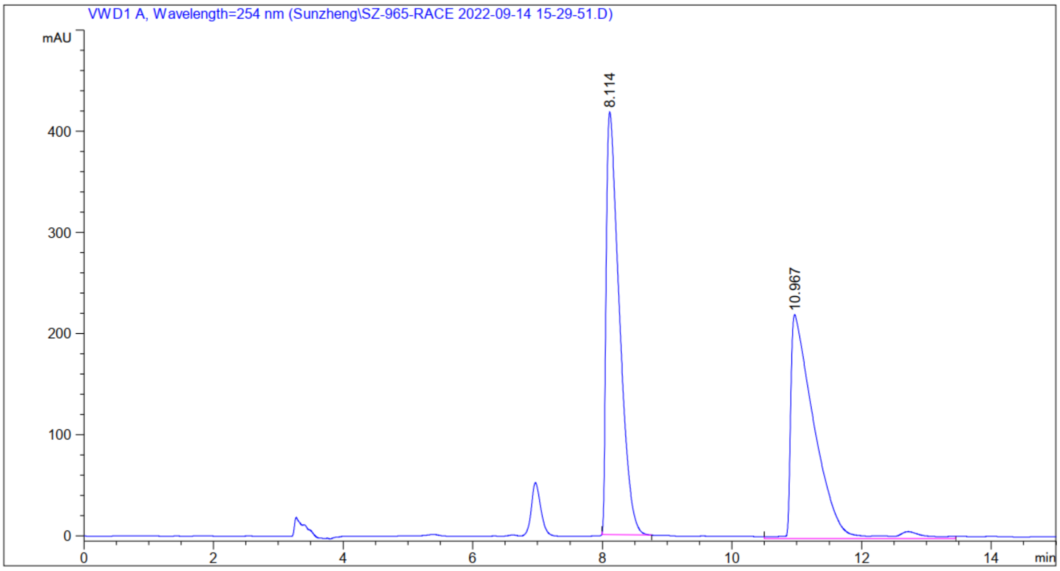

Peak #	RetTime [min]	Type	Width [min]	Area [mAU*s]	Height [mAU]	Area %
1	8.114	MM	0.2353	5898.90088	417.77325	50.8968
2	10.967	MM	0.4290	5691.02539	221.07976	49.1032

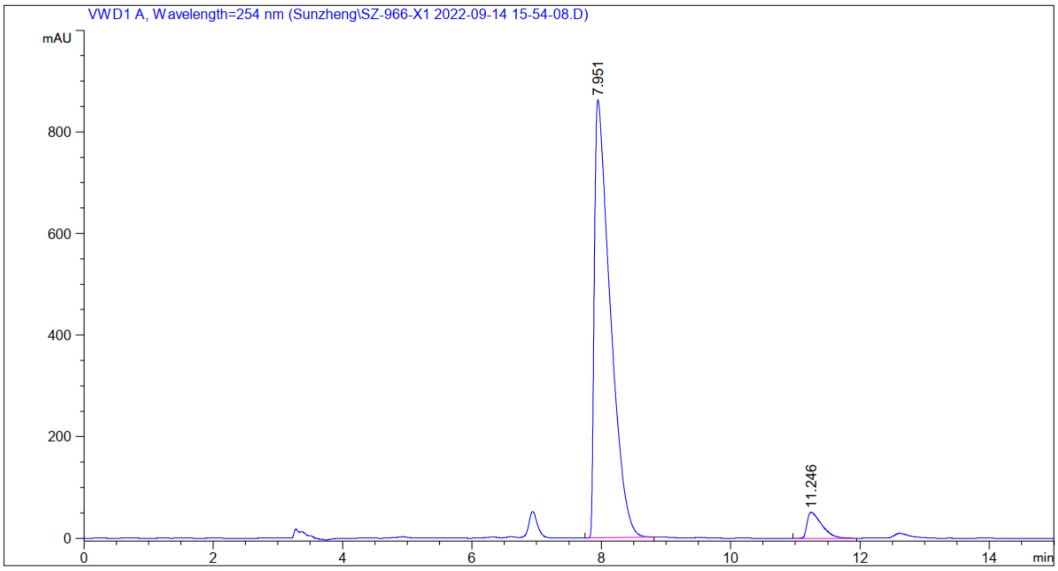

Peak #	RetTime [min]	Type	Width [min]	Area [mAU*s]	Height [mAU]	Area %
1	7.951	BB	0.2512	1.47267e4	862.47687	94.8724
2	11.246	BB	0.2373	795.94110	51.21516	5.1276

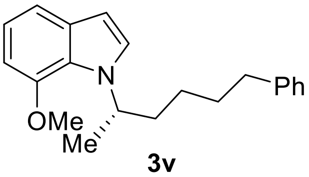

3v

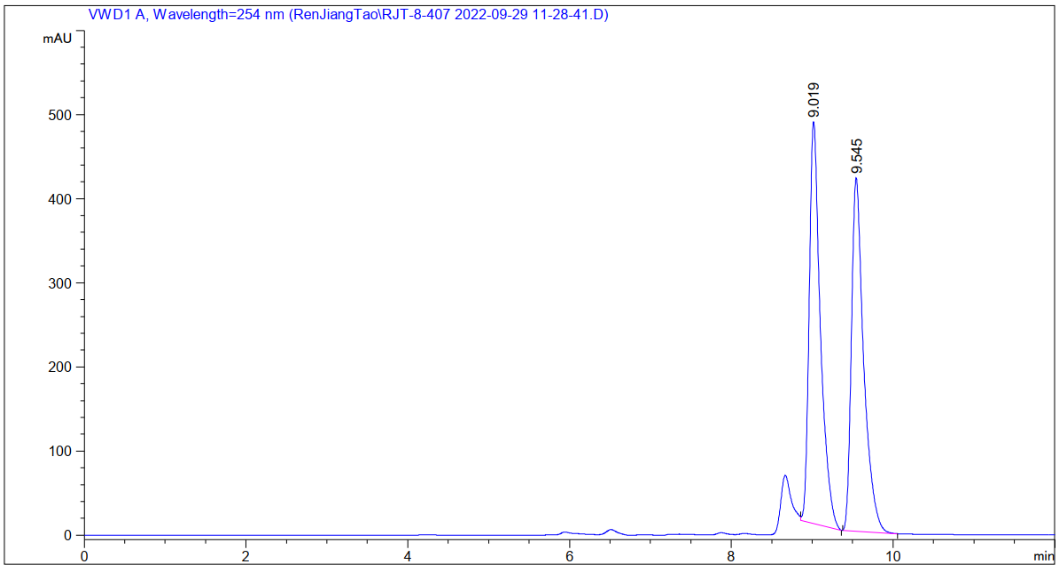

Peak #	RetTime [min]	Type	Width [min]	Area [mAU*s]	Height [mAU]	Area %
1	9.019	MM	0.1607	4605.89014	477.66949	50.9740
2	9.545	MM	0.1758	4429.86914	420.05359	49.0260

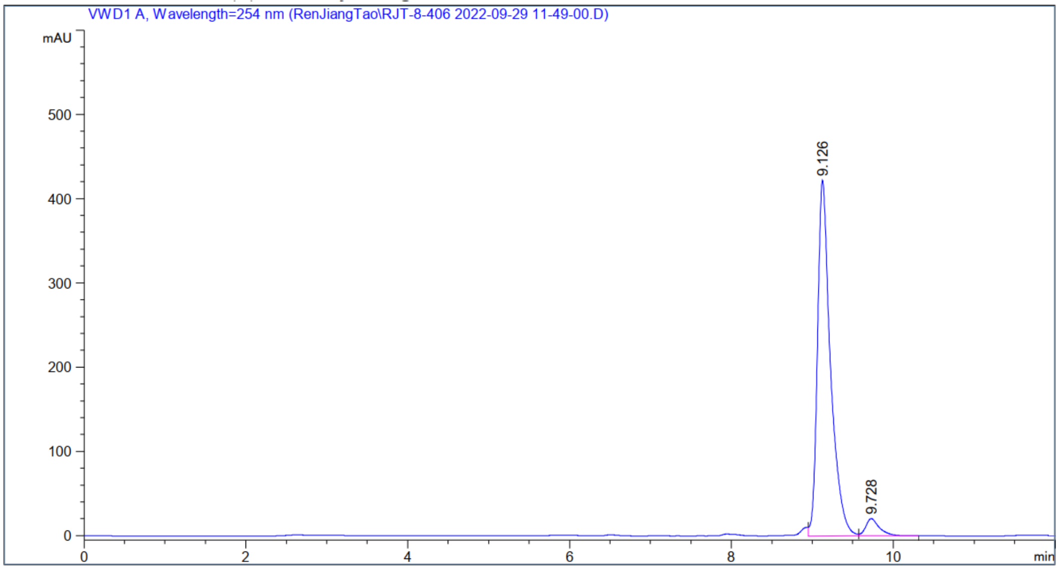

Peak #	RetTime [min]	Type	Width [min]	Area [mAU*s]	Height [mAU]	Area %
1	9.126	MF	0.1857	4699.98193	421.87411	95.1529
2	9.728	FM	0.1956	239.41774	20.40476	4.8471

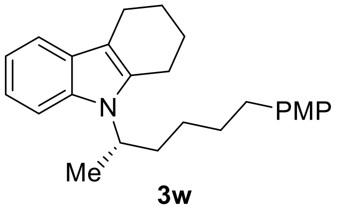

3w

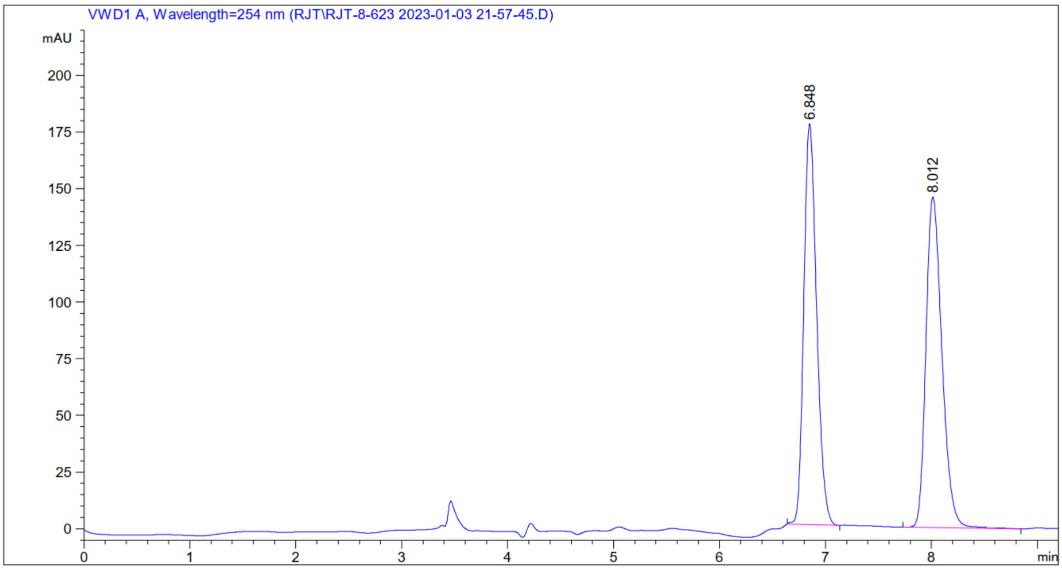

Peak #	RetTime [min]	Type	Width [min]	Area [mAU*s]	Height [mAU]	Area %
1	6.848	MM	0.1397	1482.49536	176.83096	49.1666
2	8.012	BB	0.1627	1532.75244	145.77699	50.8334

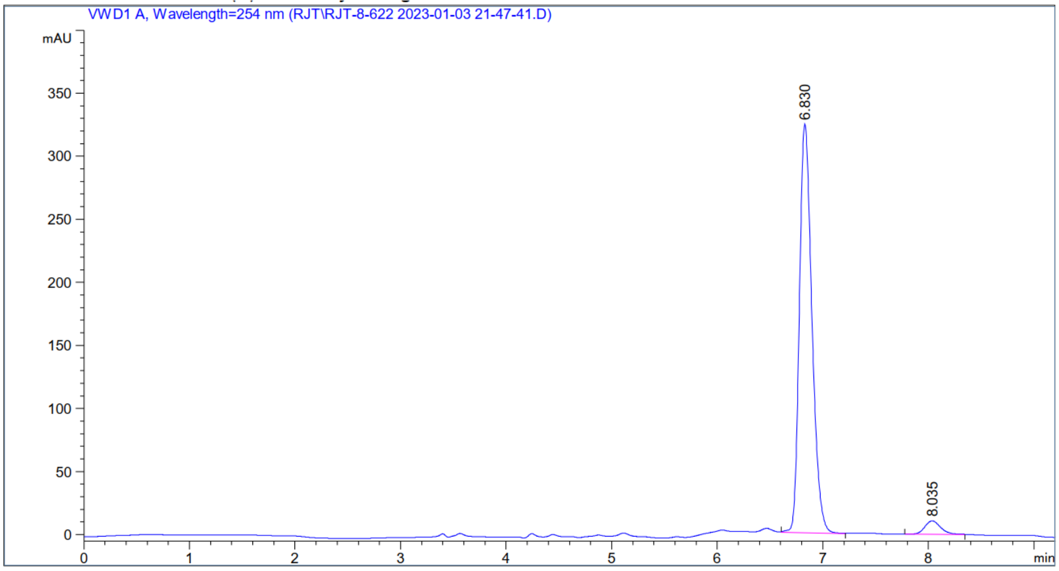

Peak #	RetTime [min]	Type	Width [min]	Area [mAU*s]	Height [mAU]	Area %
1	6.830	FM	0.1372	2669.17285	324.34079	95.9977
2	8.035	MM	0.1735	111.28303	10.68843	4.0023

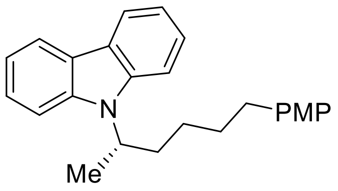

3x

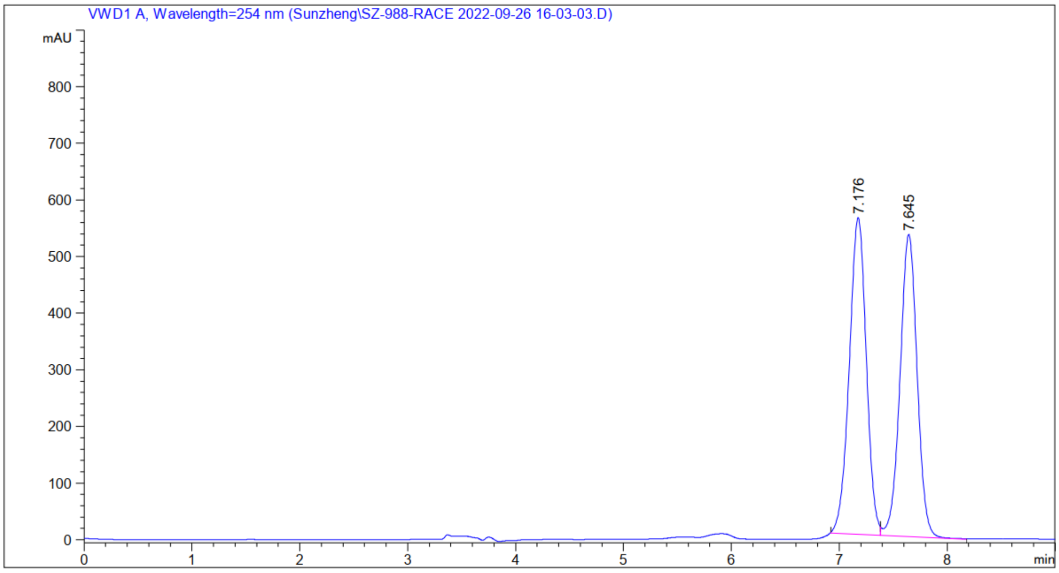

Peak #	RetTime [min]	Type	Width [min]	Area [mAU*s]	Height [mAU]	Area %
1	7.176	MF	0.1827	6130.45996	559.30133	50.9915
2	7.645	FM	0.1839	5892.06494	534.03363	49.0085

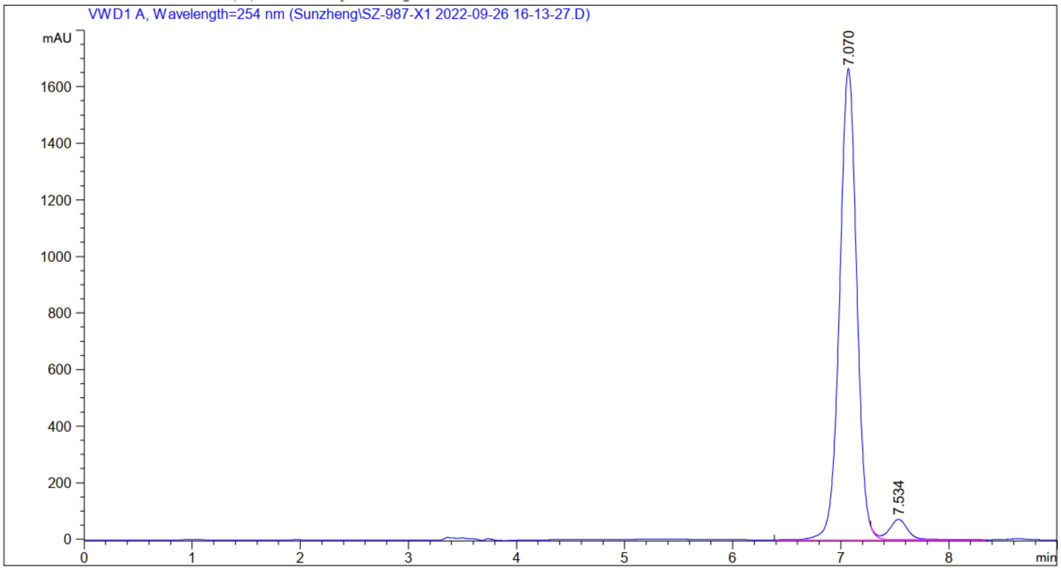

Peak #	RetTime [min]	Type	Width [min]	Area [mAU*s]	Height [mAU]	Area %
1	7.070	BV R	0.1697	1.83766e4	1665.90308	95.5045
2	7.534	VB E	0.1795	865.00317	72.34859	4.4955

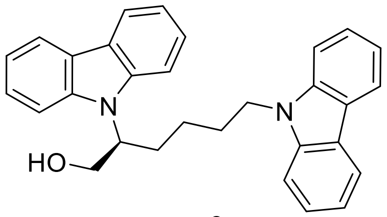

3y

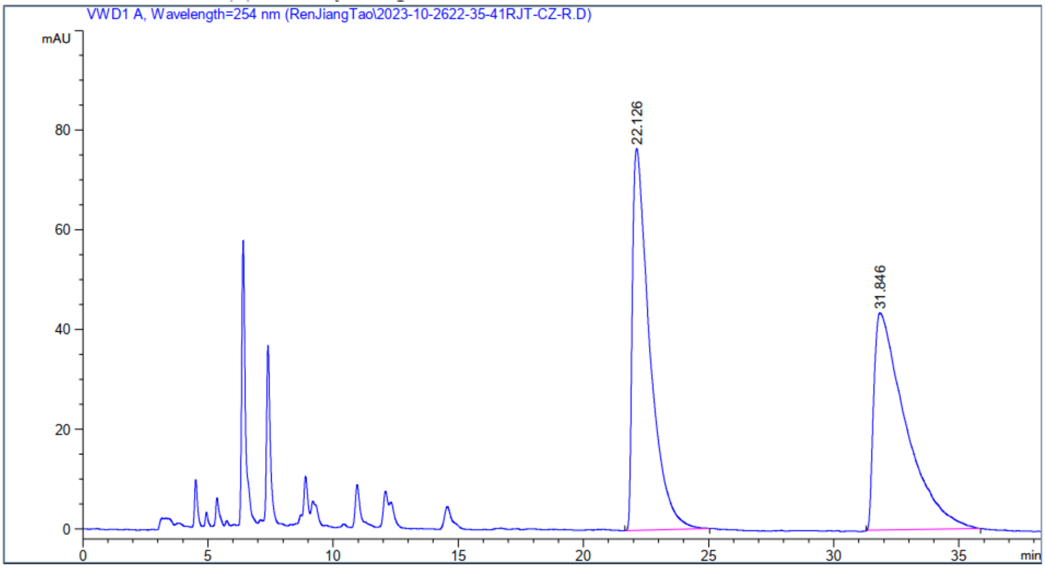

Peak #	RetTime [min]	Type	Width [min]	Area [mAU*s]	Height [mAU]	Area %
1	22.126	BB	0.6842	3700.68359	76.65767	49.1256
2	31.846	MM	1.4665	3832.42065	43.55431	50.8744

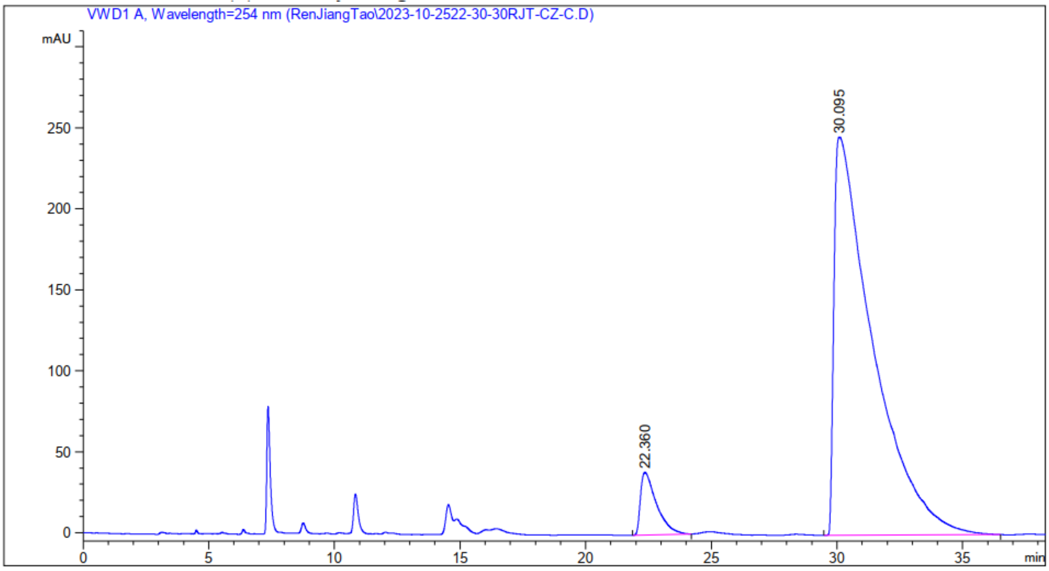

Peak #	RetTime [min]	Type	Width [min]	Area [mAU*s]	Height [mAU]	Area %
1	22.360	BB	0.6549	1717.23596	38.76225	6.1274
2	30.095	BB	1.4111	2.63083e4	245.91444	93.8726

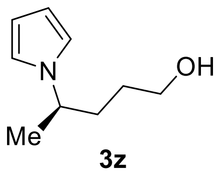

3z

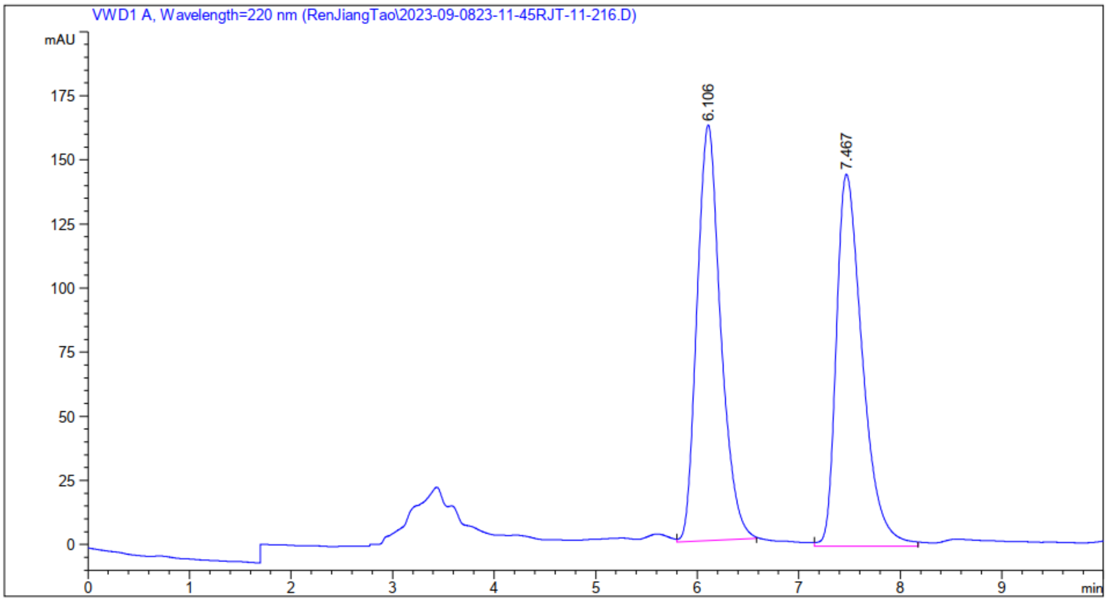

Peak #	RetTime [min]	Type	Width [min]	Area [mAU*s]	Height [mAU]	Area %
1	6.106	MM	0.2700	2627.46484	162.15923	50.3493
2	7.467	MM	0.2977	2591.01123	145.08147	49.6507

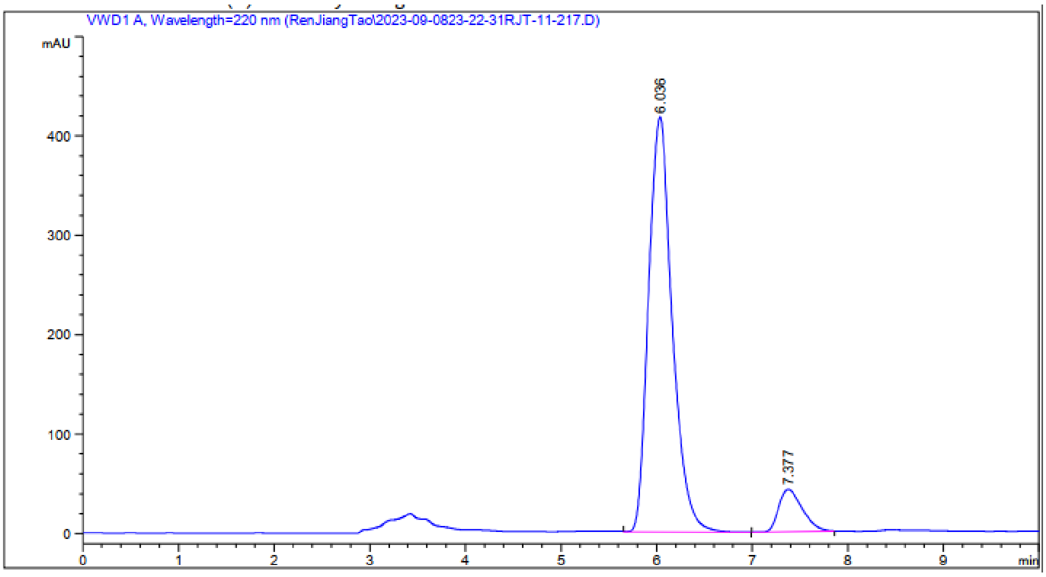

Peak #	RetTime [min]	Type	Width [min]	Area [mAU*s]	Height [mAU]	Area %
1	6.036	FM	0.2864	7174.88574	417.58795	90.8700
2	7.377	MM	0.2836	720.88086	42.36730	9.1300

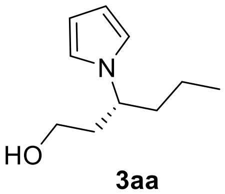

3aa

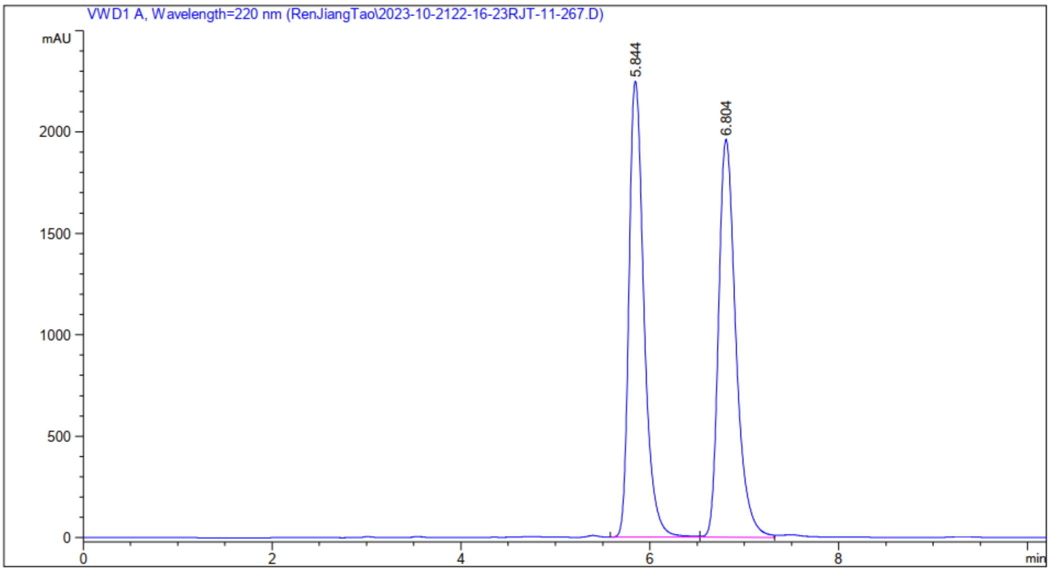

Peak #	RetTime [min]	Type	Width [min]	Area [mAU*s]	Height [mAU]	Area %
1	5.844	BV	0.1696	2.47741e4	2247.02759	49.7970
2	6.804	MF	0.2123	2.49761e4	1961.08264	50.2030

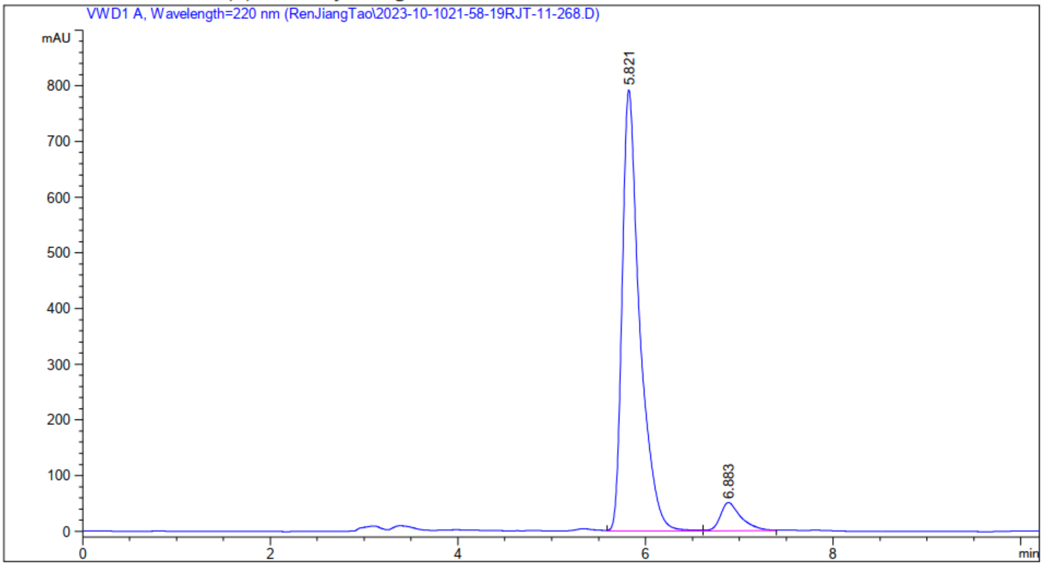

Peak #	RetTime [min]	Type	Width [min]	Area [mAU*s]	Height [mAU]	Area %
1	5.821	MF	0.2161	1.02741e4	792.26111	92.9698
2	6.883	FM	0.2555	776.91248	50.66999	7.0302

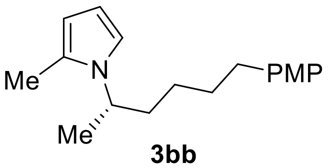

3bb

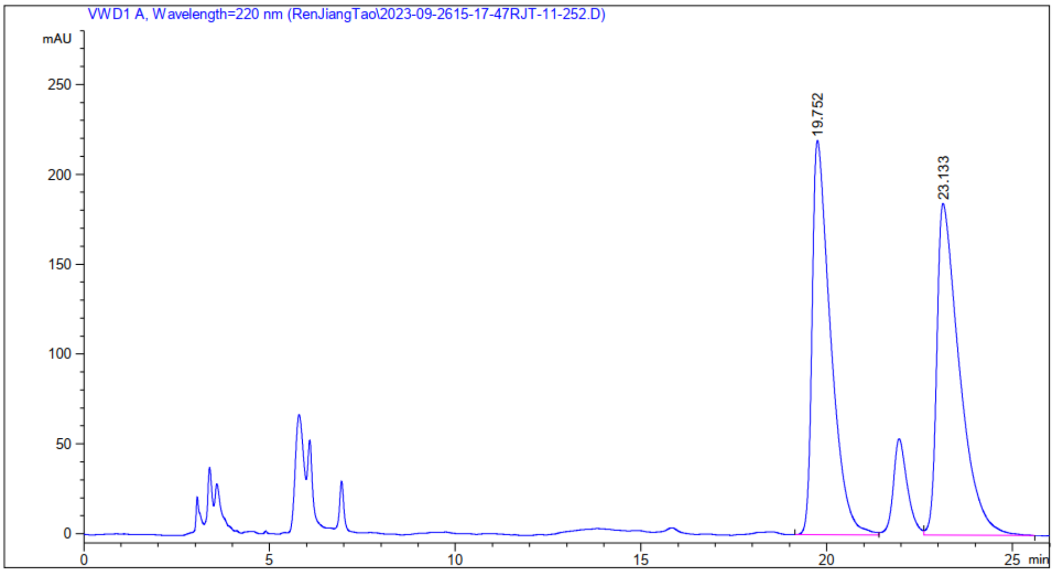

Peak #	RetTime [min]	Type	Width [min]	Area [mAU*s]	Height [mAU]	Area %
1	19.752	BV	0.5131	7602.94531	219.51051	49.1858
2	23.133	VB	0.6222	7854.65869	184.67445	50.8142

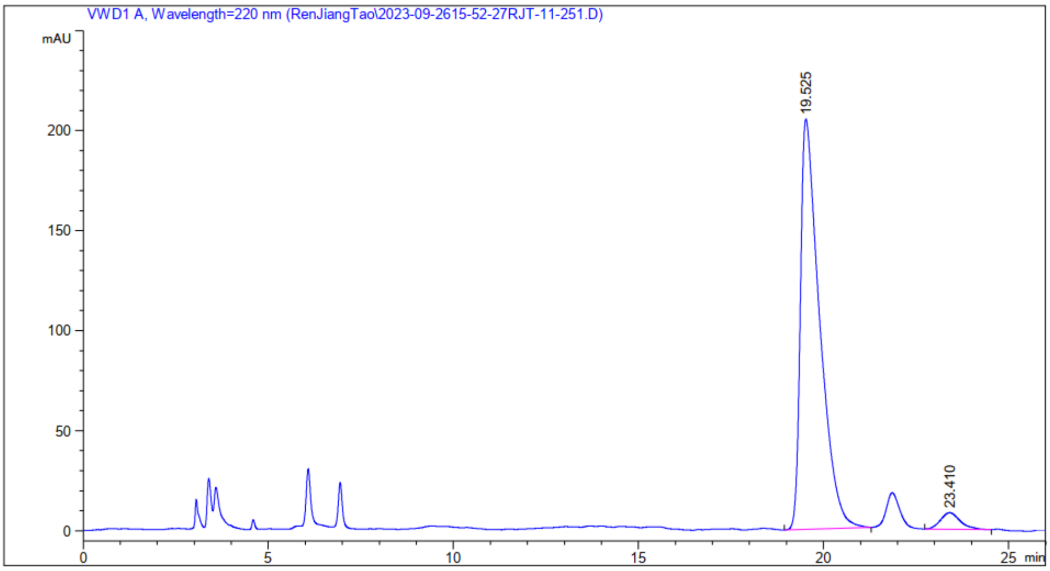

Peak #	RetTime [min]	Type	Width [min]	Area [mAU*s]	Height [mAU]	Area %
1	19.525	BB	0.5257	7377.24707	205.06053	96.0650
2	23.410	BB	0.5573	302.18860	8.29009	3.9350

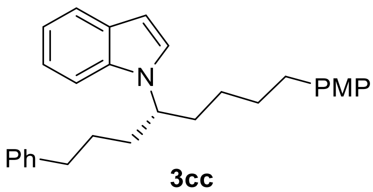

3cc

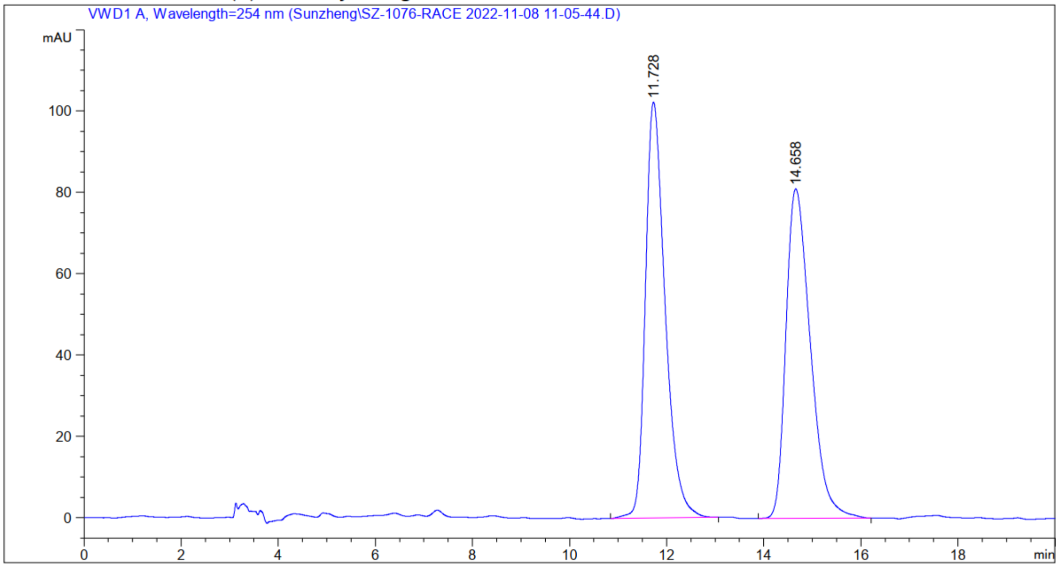

Peak #	RetTime [min]	Type	Width [min]	Area [mAU*s]	Height [mAU]	Area %
1	11.728	BB	0.4276	2882.01904	102.32038	50.4218
2	14.658	BB	0.5400	2833.80005	81.03551	49.5782

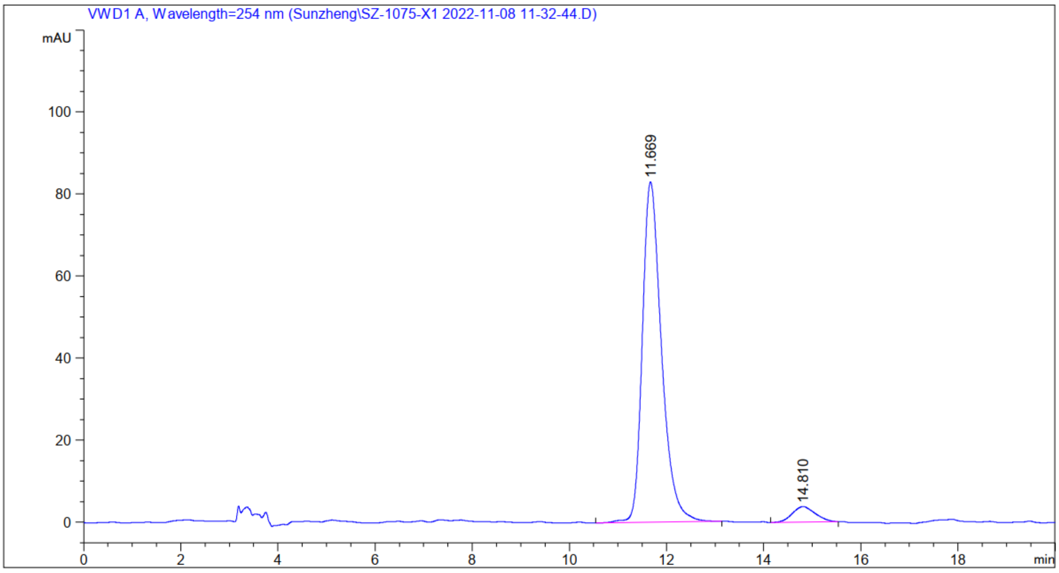

Peak #	RetTime [min]	Type	Width [min]	Area [mAU*s]	Height [mAU]	Area %
1	11.669	BB	0.4166	2279.50513	82.96323	94.8310
2	14.810	BB	0.4792	124.24921	3.82271	5.1690

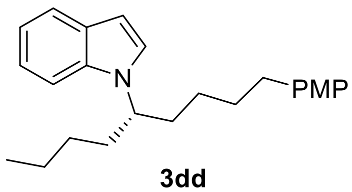

3dd

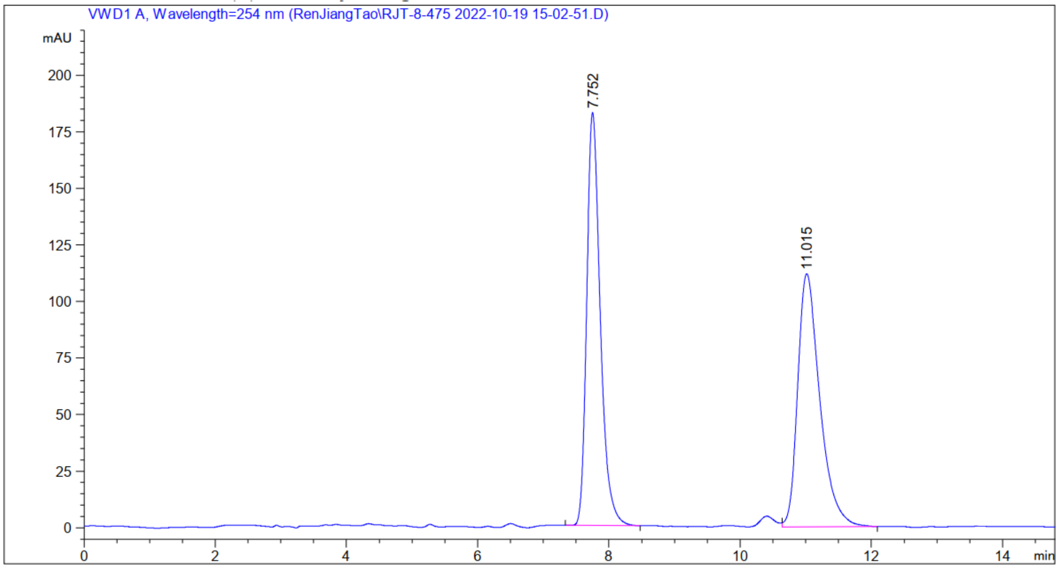

Peak #	RetTime [min]	Type	Width [min]	Area [mAU*s]	Height [mAU]	Area %
1	7.752	BB	0.2193	2617.92944	182.52159	49.9760
2	11.015	FM	0.3904	2620.44775	111.87058	50.0240

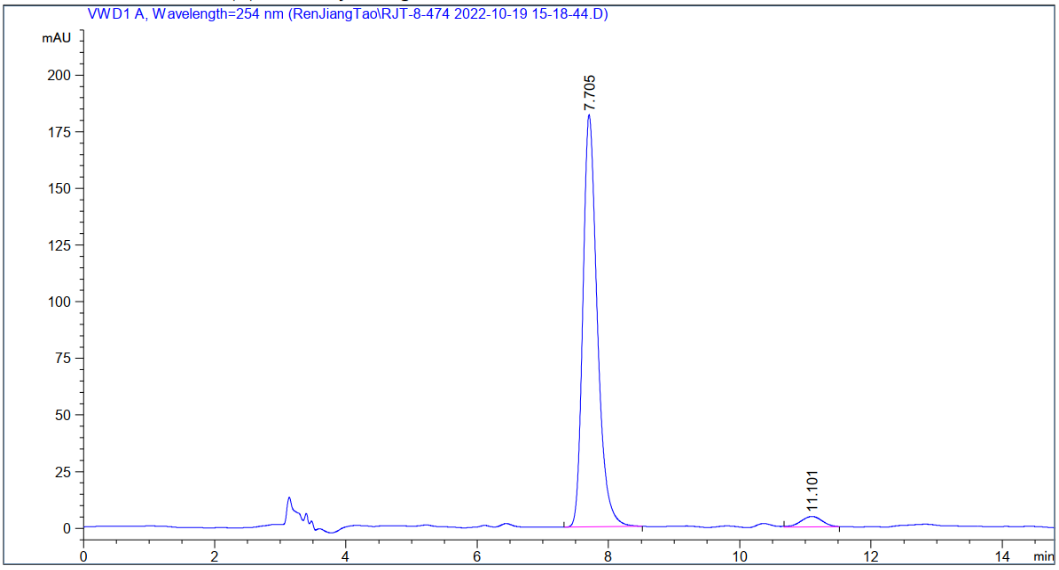

Peak #	RetTime [min]	Type	Width [min]	Area [mAU*s]	Height [mAU]	Area %
1	7.705	MM	0.2544	2775.31274	181.84474	96.4805
2	11.101	FM	0.3702	101.24051	4.55782	3.5195

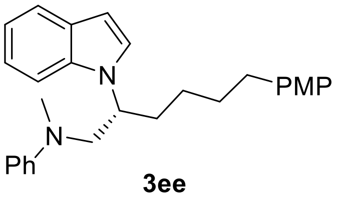

3ee

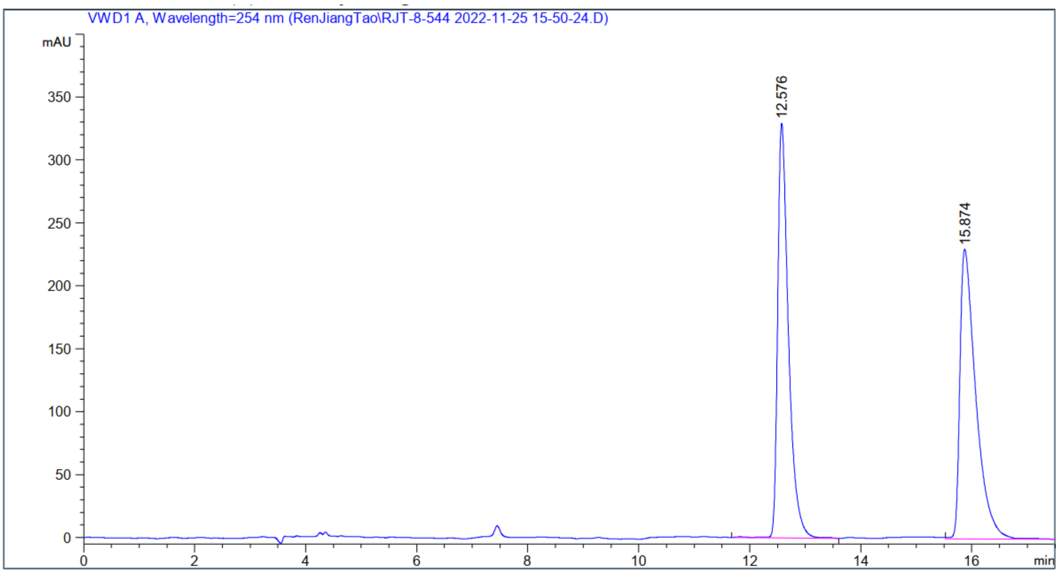

Peak #	RetTime [min]	Type	Width [min]	Area [mAU*s]	Height [mAU]	Area %
1	12.576	VV R	0.2014	4444.71631	329.45547	49.1276
2	15.874	FM	0.3333	4602.56689	230.13330	50.8724

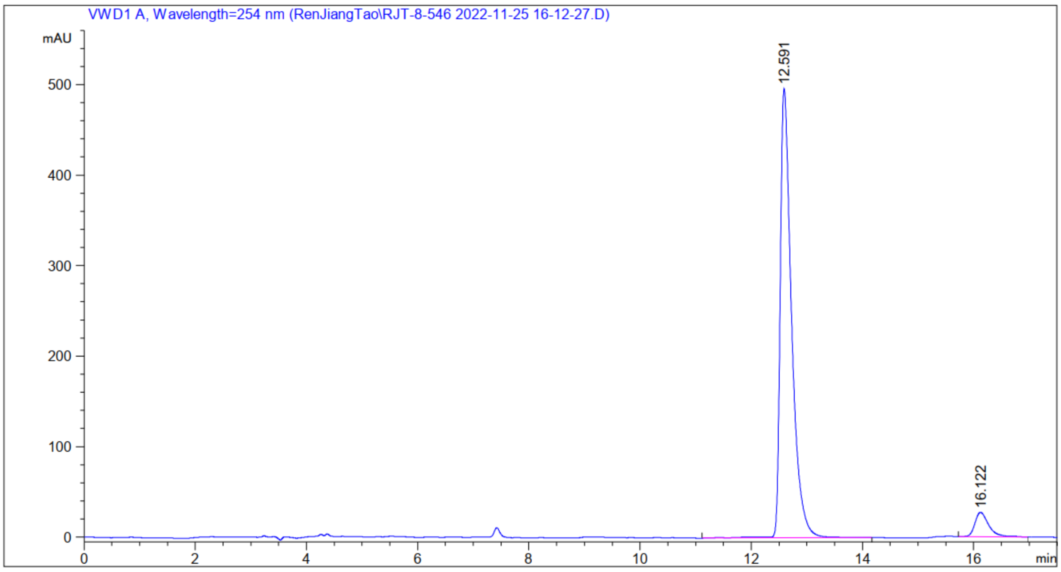

Peak #	RetTime [min]	Type	Width [min]	Area [mAU*s]	Height [mAU]	Area %
1	12.591	VV R	0.2127	7051.81641	496.39084	93.7953
2	16.122	BB	0.2620	466.48499	26.94705	6.2047

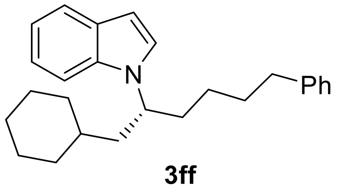

3ff

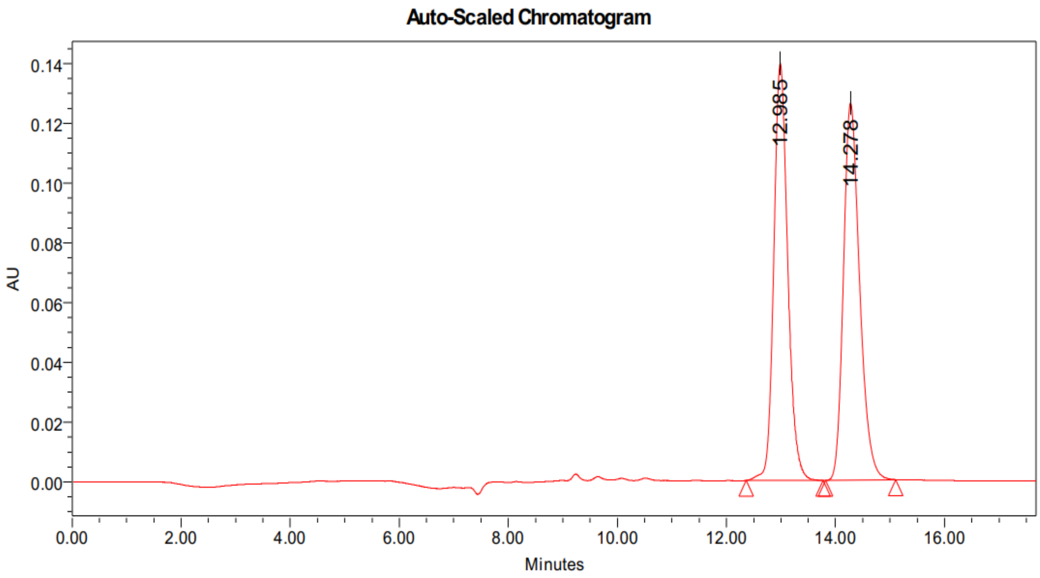

Peak Results

	RT	Area	Height	% Area
1	12.985	2475331	139585	48.86
2	14.278	2591286	126243	51.14

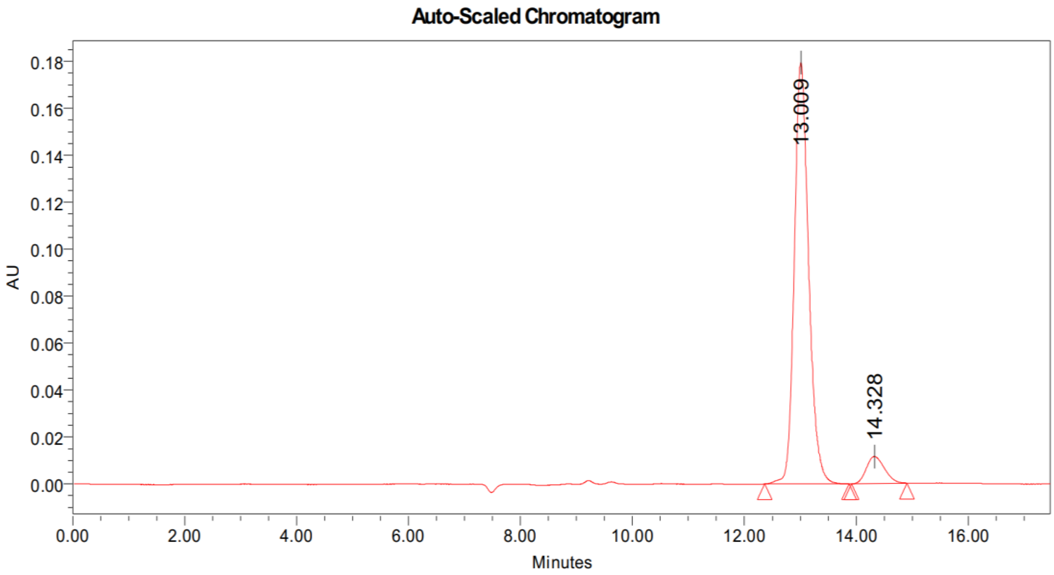

Peak Results

	RT	Area	Height	% Area
1	13.009	3210022	179531	92.53
2	14.328	259318	11560	7.47

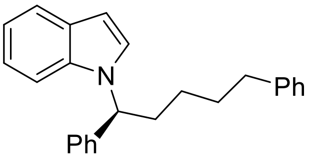

3gg

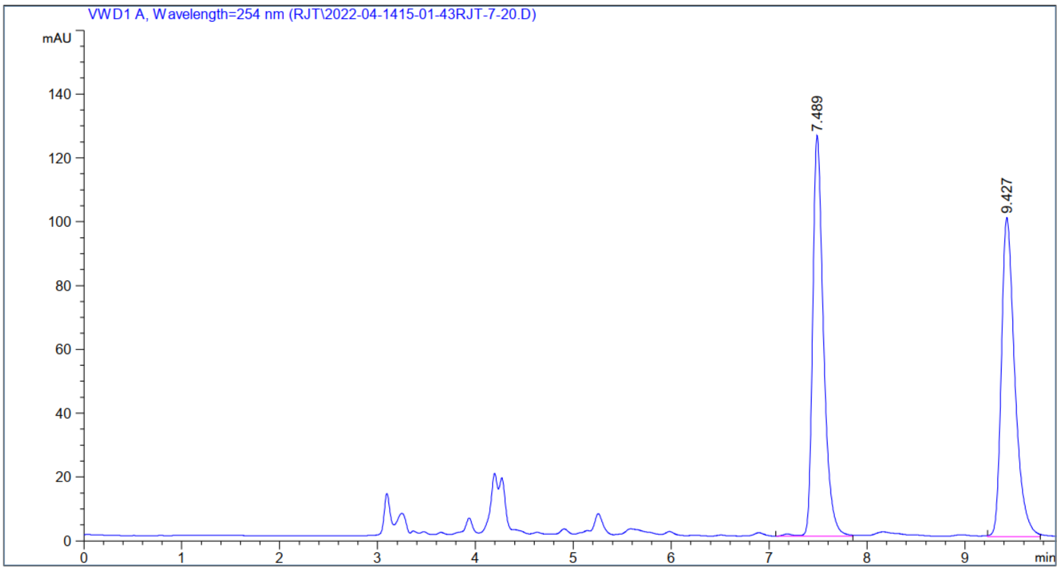

Peak #	RetTime [min]	Type	Width [min]	Area [mAU*s]	Height [mAU]	Area %
1	7.489	MF	0.1285	968.39893	125.58207	50.3740
2	9.427	FM	0.1590	954.01886	100.01637	49.6260

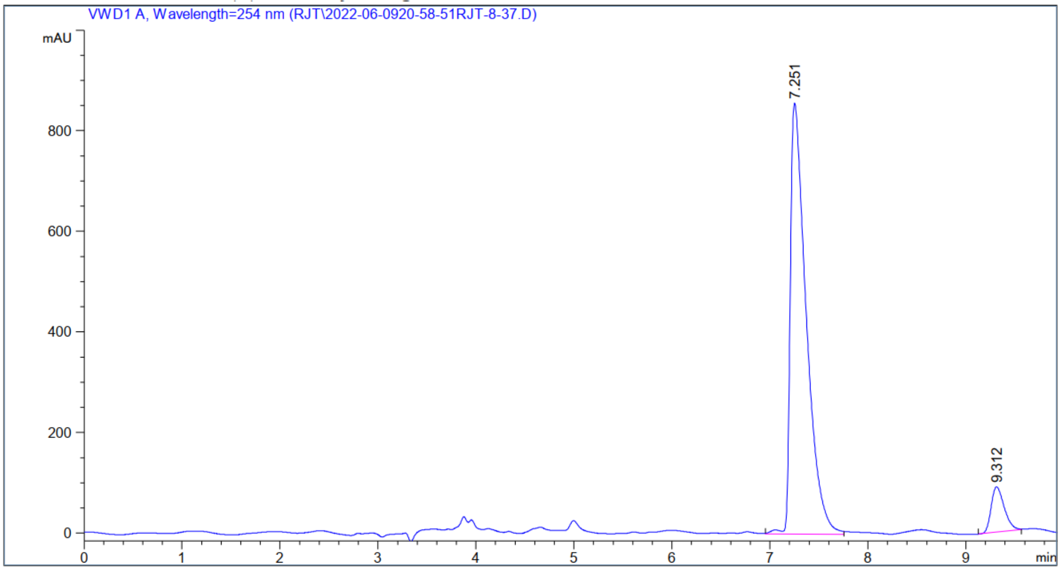

Peak #	RetTime [min]	Type	Width [min]	Area [mAU*s]	Height [mAU]	Area %
1	7.251	MF	0.1889	9711.76758	856.77905	92.0946
2	9.312	MM	0.1542	833.65991	90.12057	7.9054

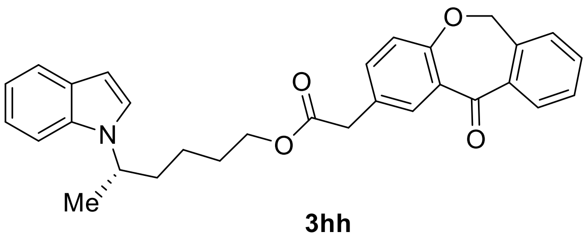

3hh

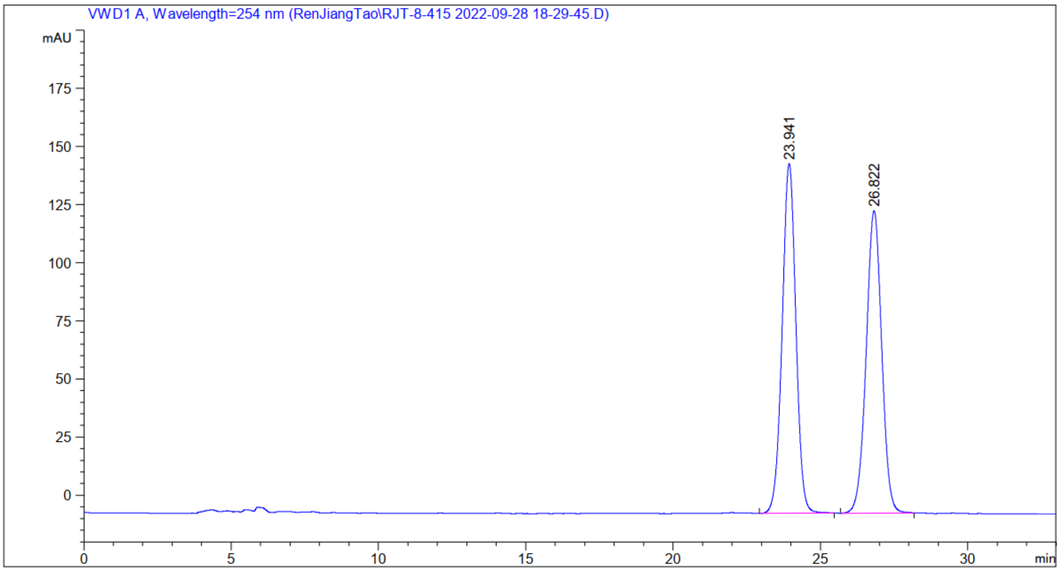

Peak #	RetTime [min]	Type	Width [min]	Area [mAU*s]	Height [mAU]	Area %
1	23.941	BB	0.5018	4869.89600	150.39545	50.5228
2	26.822	BB	0.5632	4769.11914	129.94809	49.4772

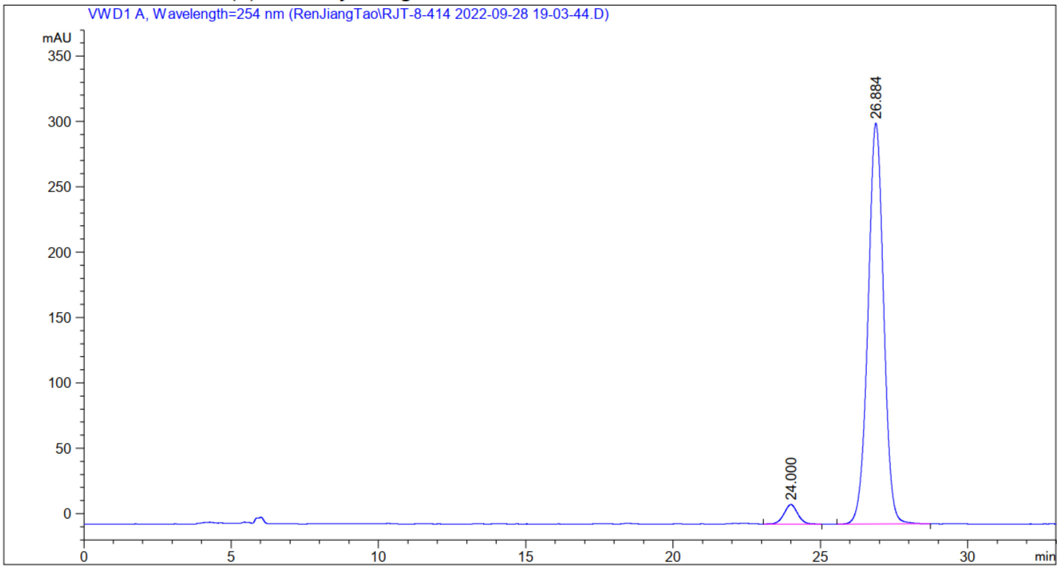

Peak #	RetTime [min]	Type	Width [min]	Area [mAU*s]	Height [mAU]	Area %
1	24.000	BB	0.4936	484.89551	15.02084	4.1114
2	26.884	BB	0.5682	1.13092e4	306.71332	95.8886

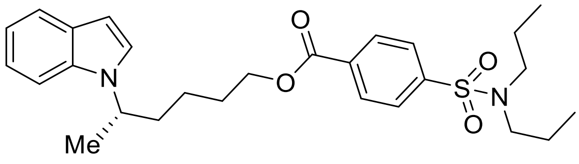

3ii

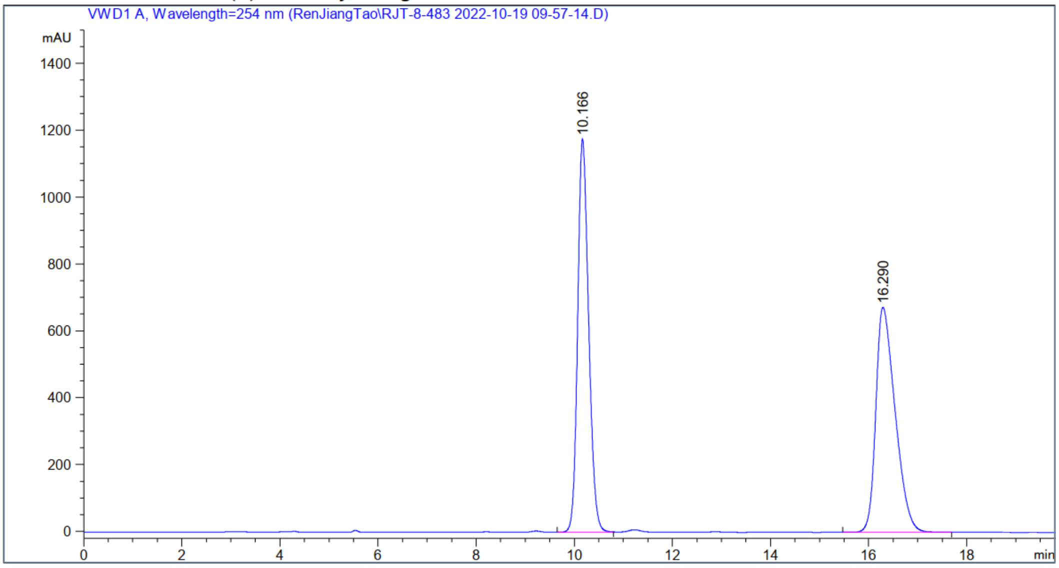

Peak #	RetTime [min]	Type	Width [min]	Area [mAU*s]	Height [mAU]	Area %
1	10.166	MF	0.2584	1.82393e4	1176.49658	49.8092
2	16.290	BB	0.4136	1.83790e4	673.03278	50.1908

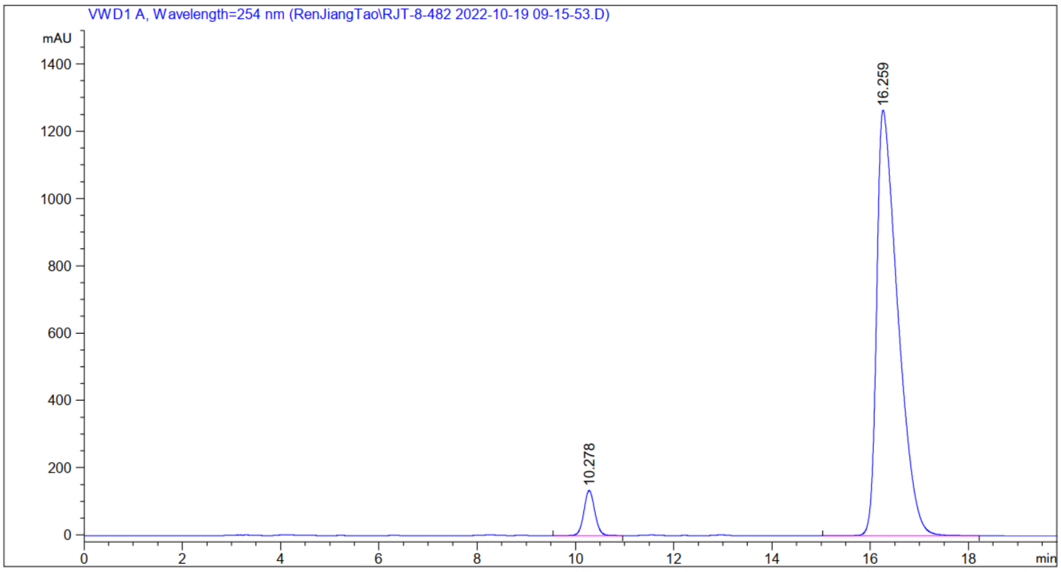

Peak #	RetTime [min]	Type	Width [min]	Area [mAU*s]	Height [mAU]	Area %
1	10.278	BB	0.2380	2053.11841	133.80783	5.1598
2	16.259	BB	0.4530	3.77376e4	1265.17114	94.8402

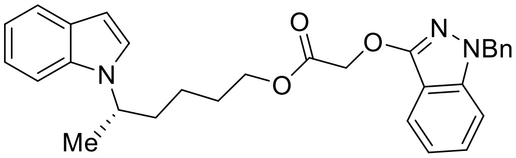

3jj

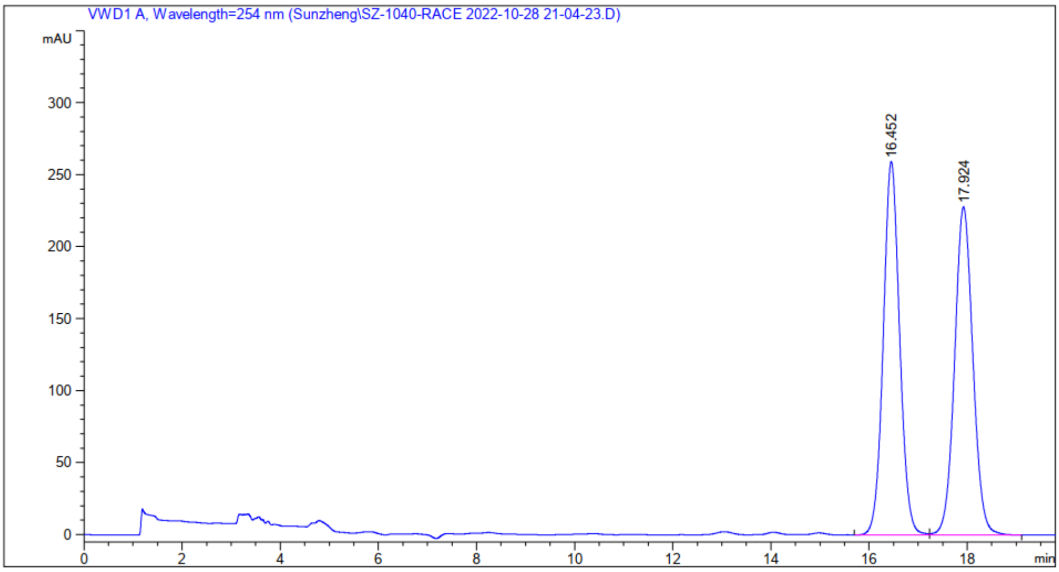

Peak #	RetTime [min]	Type	Width [min]	Area [mAU*s]	Height [mAU]	Area %
1	16.452	BV	0.3540	6005.38232	259.52322	50.4026
2	17.924	VB	0.4006	5909.44678	227.81088	49.5974

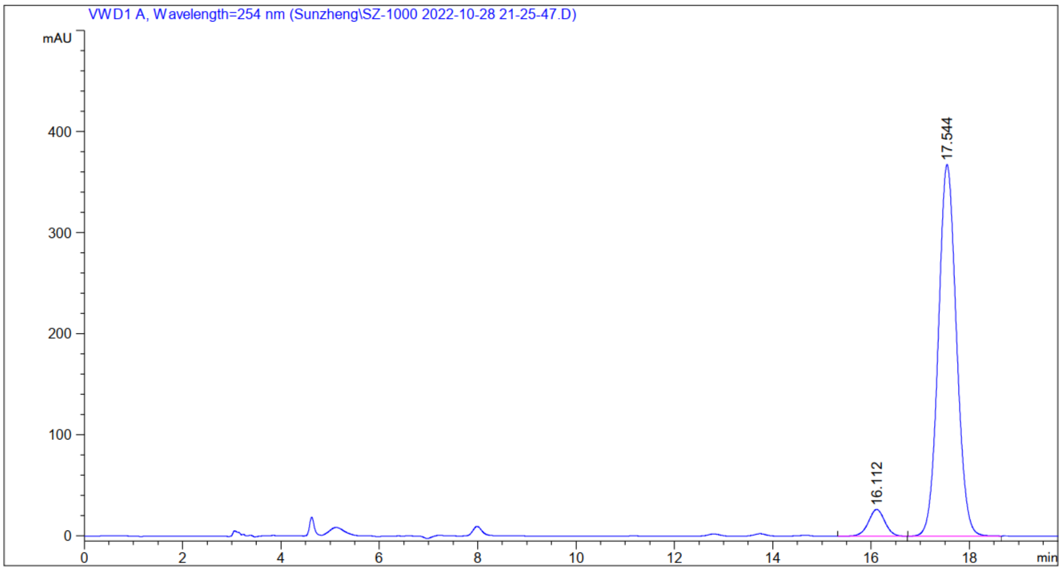

Peak #	RetTime [min]	Type	Width [min]	Area [mAU*s]	Height [mAU]	Area %
1	16.112	BB	0.3586	619.72394	26.52755	6.1773
2	17.544	BB	0.3949	9412.55273	367.42508	93.8227

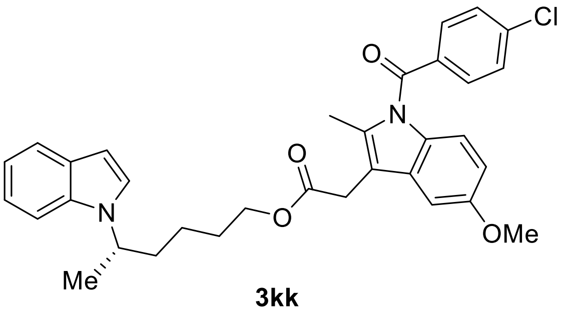

3kk

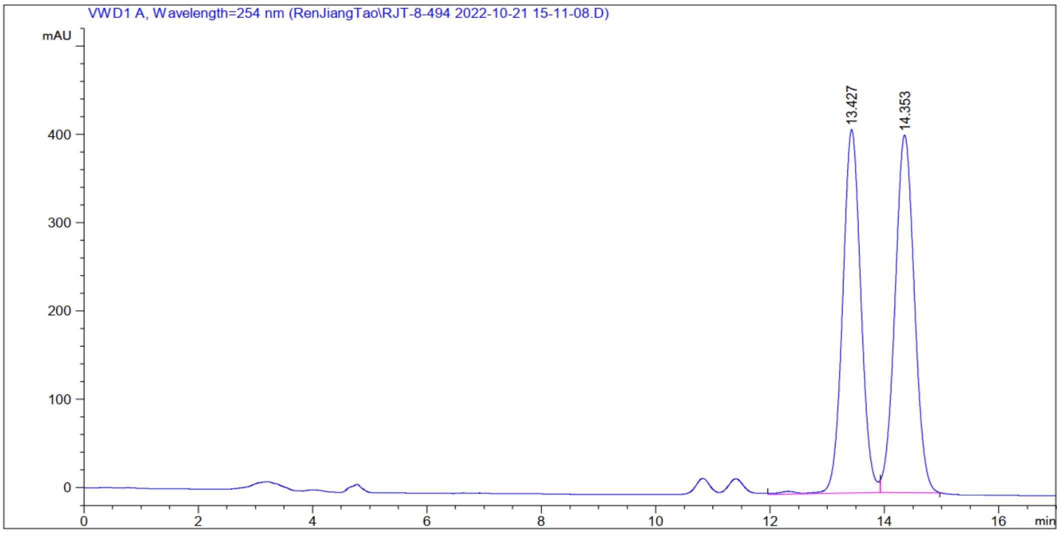

Peak #	RetTime [min]	Type	Width [min]	Area [mAU*s]	Height [mAU]	Area %
1	13.427	FM	0.3695	9120.66797	411.36417	49.0101
2	14.353	MM	0.3908	9489.09180	404.67172	50.9899

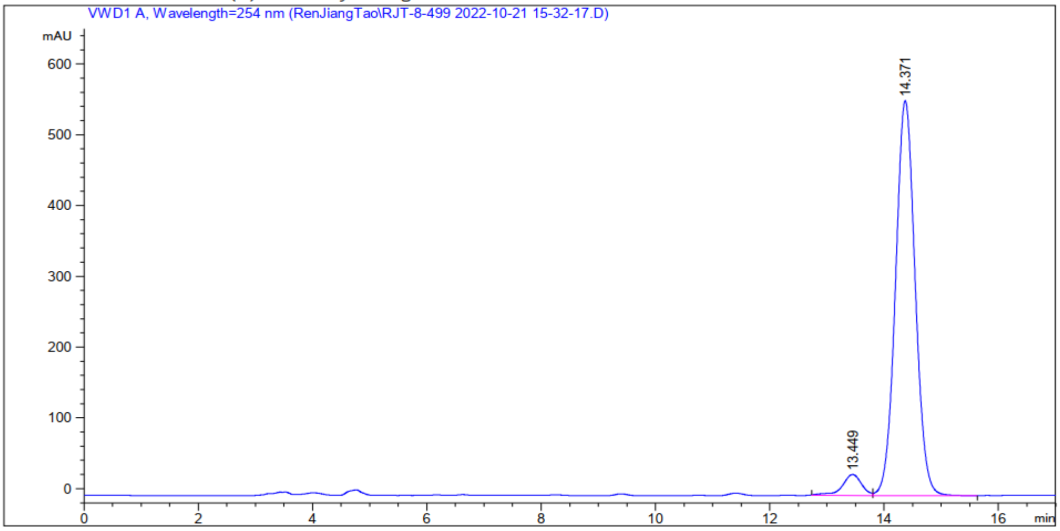

Peak #	RetTime [min]	Type	Width [min]	Area [mAU*s]	Height [mAU]	Area %
1	13.449	MF	0.3940	700.37610	29.62996	4.9918
2	14.371	FM	0.3981	1.33302e4	558.01001	95.0082

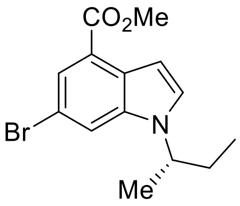

3ll

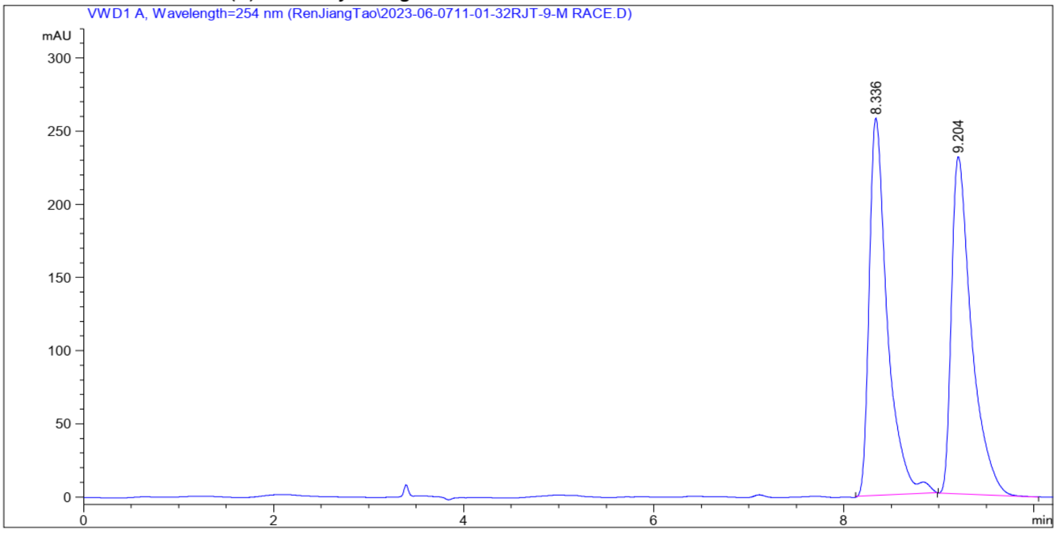

Peak #	RetTime [min]	Type	Width [min]	Area [mAU*s]	Height [mAU]	Area %
1	8.336	MM	0.2209	3417.56323	257.87036	50.2996
2	9.204	MM	0.2443	3376.84863	230.40393	49.7004

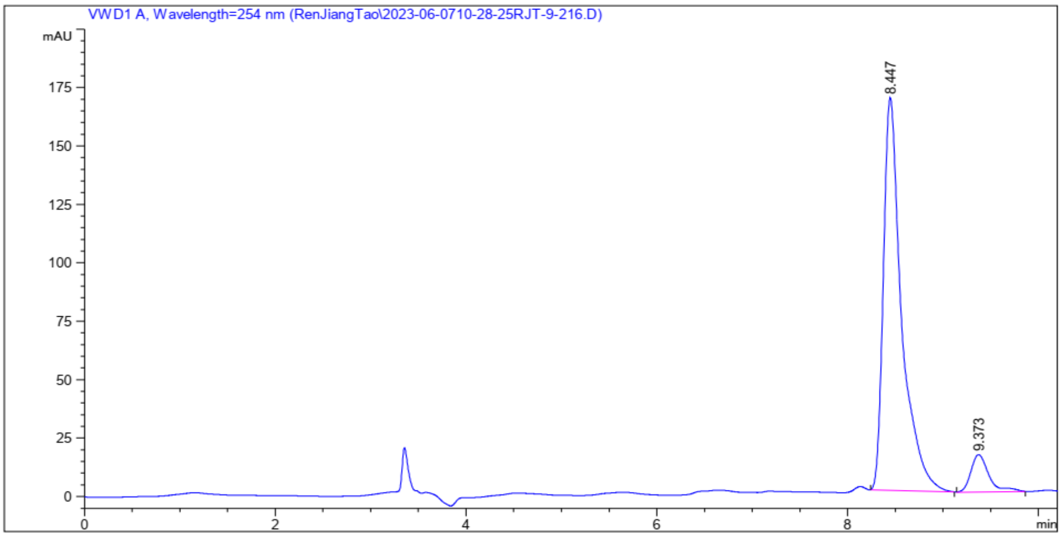

Peak #	RetTime [min]	Type	Width [min]	Area [mAU*s]	Height [mAU]	Area %
1	8.447	MM	0.2215	2232.49805	167.97688	91.3929
2	9.373	MM	0.2190	210.24873	16.00426	8.6071

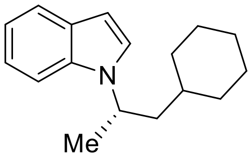

3mm

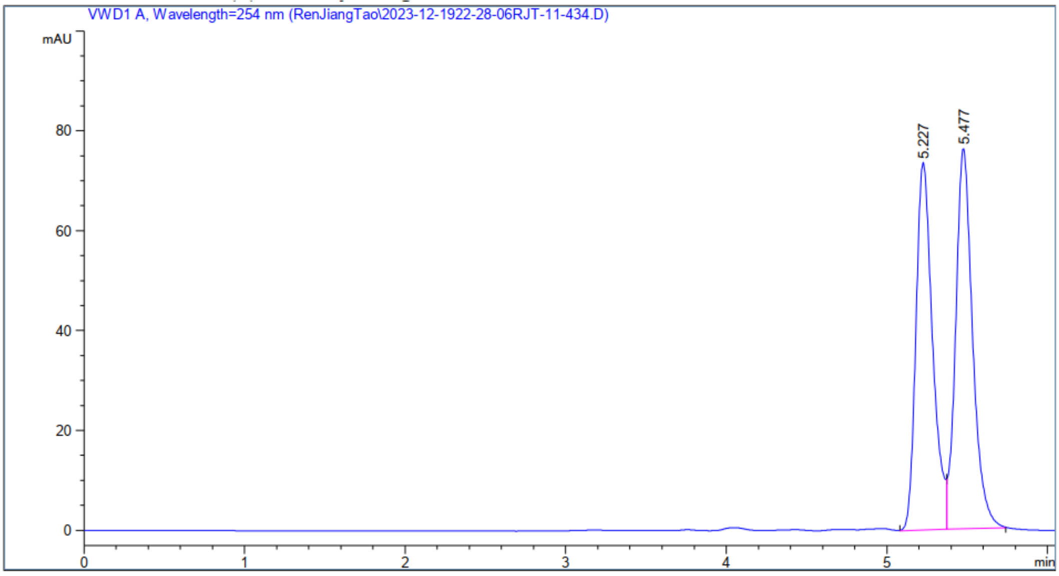

Peak #	RetTime [min]	Type	Width [min]	Area [mAU*s]	Height [mAU]	Area %
1	5.227	MF	0.1200	529.65717	73.57378	48.7373
2	5.477	FM	0.1219	557.10132	76.15025	51.2627

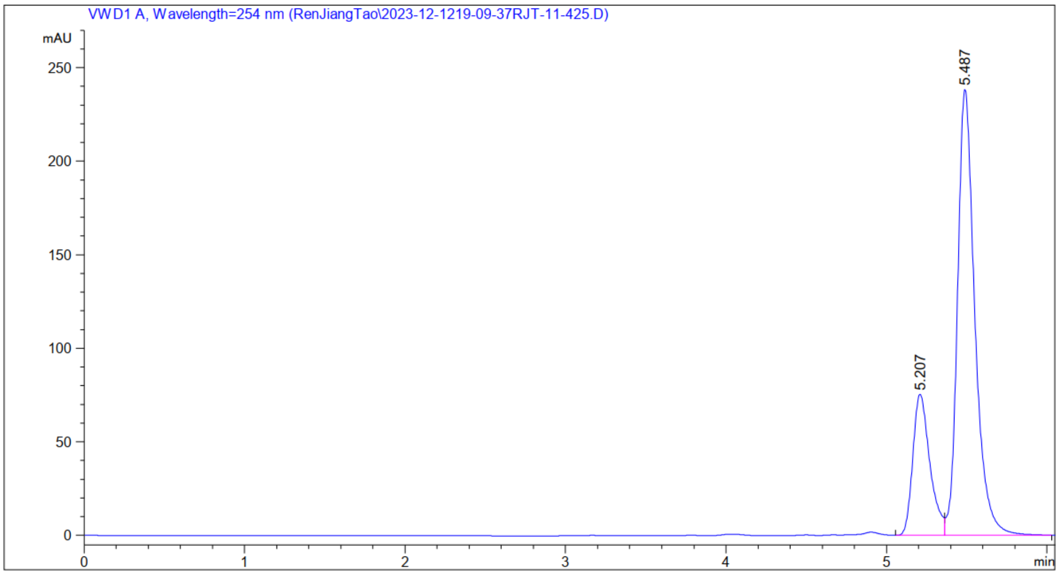

Peak #	RetTime [min]	Type	Width [min]	Area [mAU*s]	Height [mAU]	Area %
1	5.207	BV	0.1124	558.21979	75.34573	23.8322
2	5.487	VB	0.1133	1784.06885	238.32184	76.1678

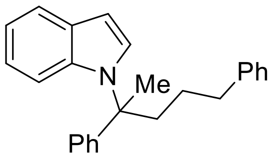

8a

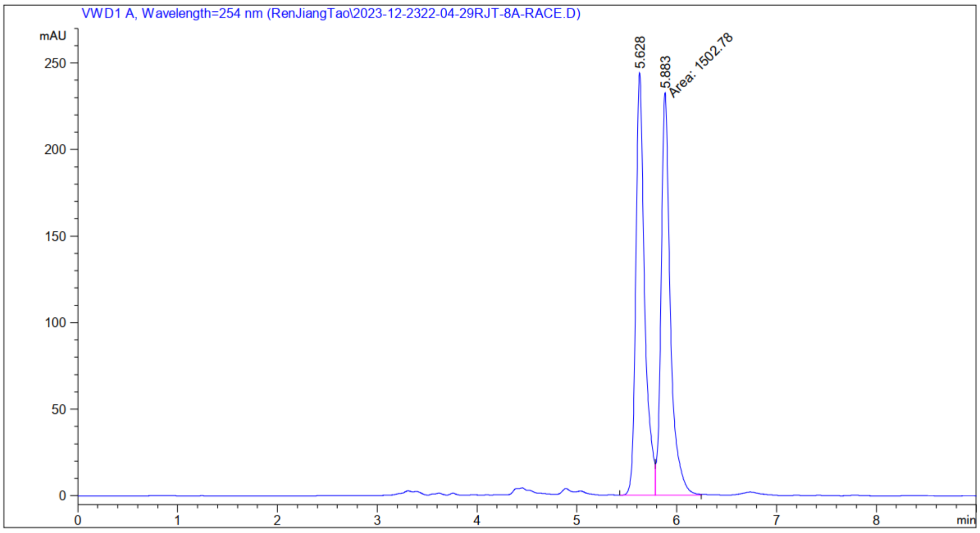

Peak #	RetTime [min]	Type	Width [min]	Area [mAU*s]	Height [mAU]	Area %
1	5.628	BV	0.0912	1509.37390	244.00101	50.1095
2	5.883	MF	0.1077	1502.77612	232.63330	49.8905

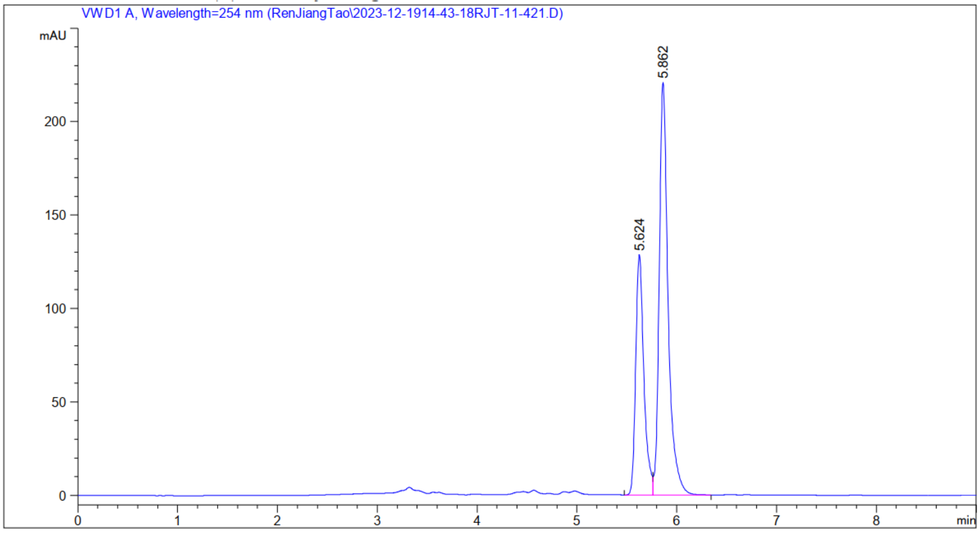

Peak #	RetTime [min]	Type	Width [min]	Area [mAU*s]	Height [mAU]	Area %
1	5.624	BV	0.0855	733.14673	128.62982	34.8814
2	5.862	VB	0.0924	1368.67749	220.62039	65.1186

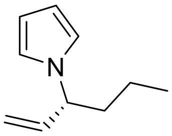

5

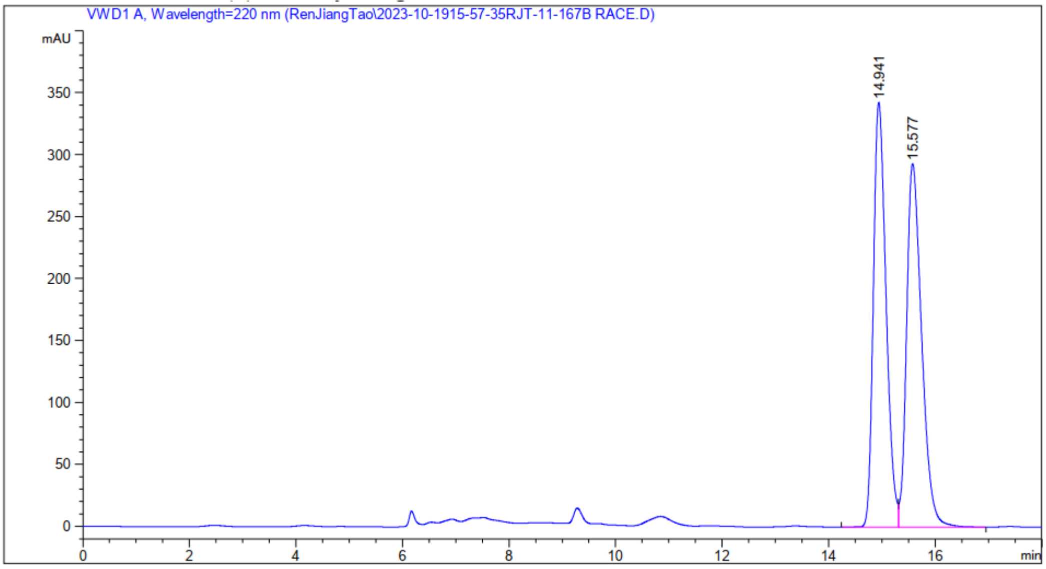

Peak #	RetTime [min]	Type	Width [min]	Area [mAU*s]	Height [mAU]	Area %
1	14.941	BV	0.2557	5659.77148	342.72760	49.5999
2	15.577	VB	0.2997	5751.07910	293.31708	50.4001

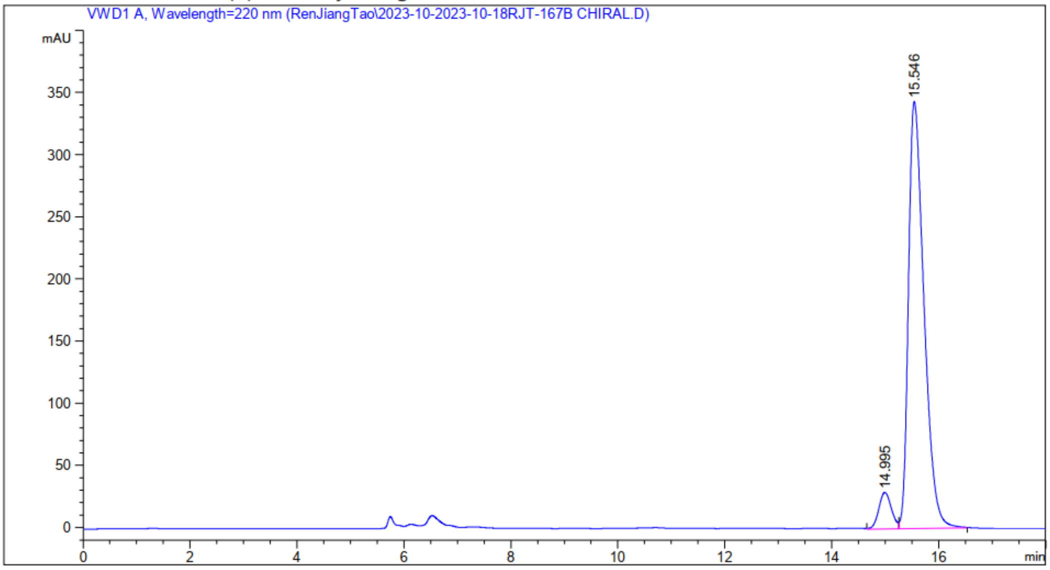

Peak #	RetTime [min]	Type	Width [min]	Area [mAU*s]	Height [mAU]	Area %
1	14.995	MF	0.2735	481.00604	29.30916	6.5152
2	15.546	FM	0.3349	6901.86133	343.49393	93.4848

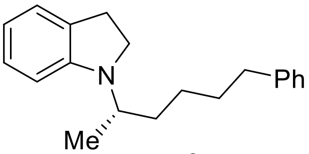

6

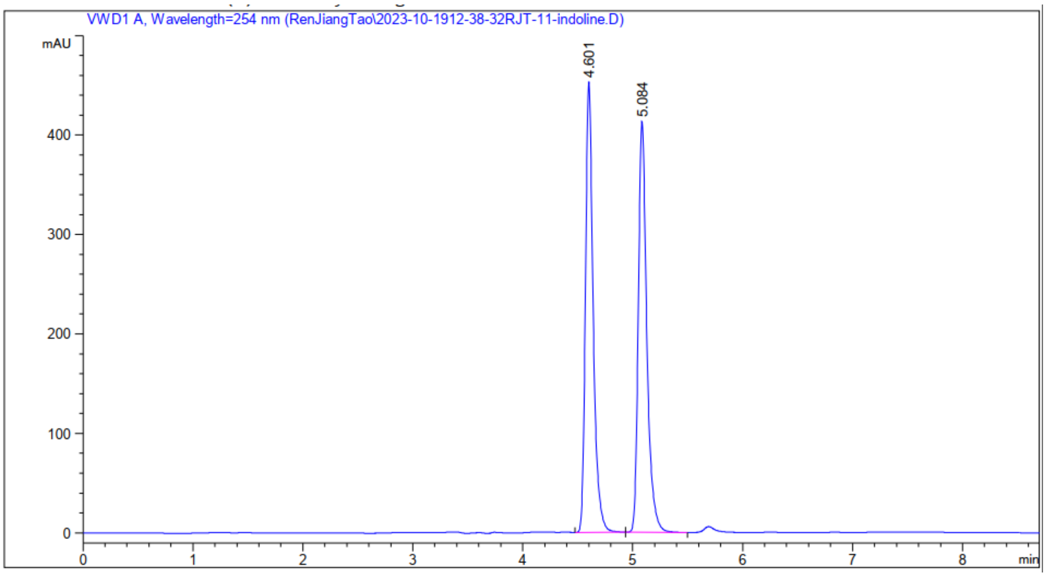

Peak #	RetTime [min]	Type	Width [min]	Area [mAU*s]	Height [mAU]	Area %
1	4.601	BB	0.0740	2214.54980	453.60327	49.9067
2	5.084	BB	0.0817	2222.83154	413.58737	50.0933

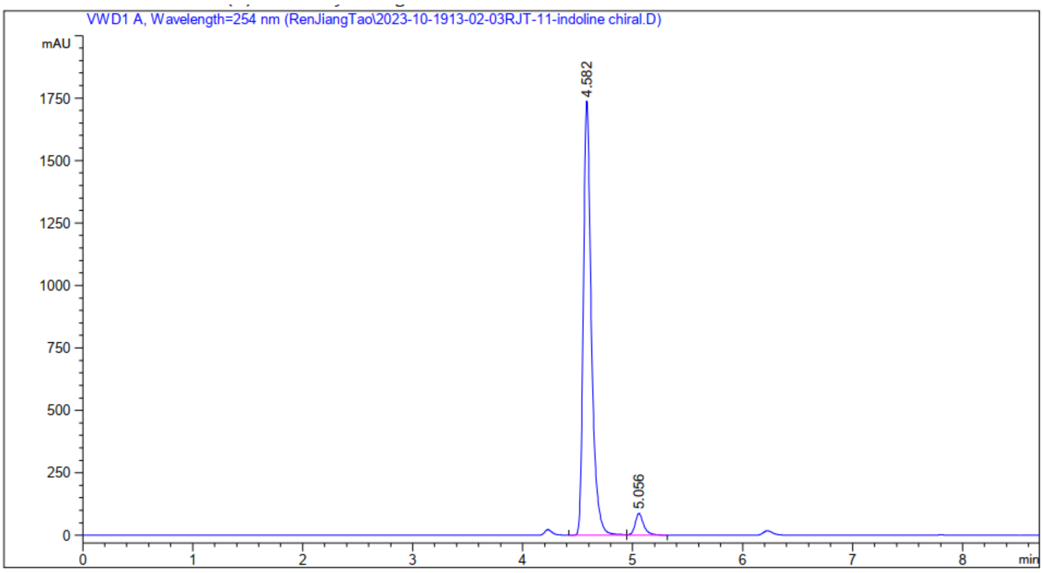

Peak #	RetTime [min]	Type	Width [min]	Area [mAU*s]	Height [mAU]	Area %
1	4.582	BV	0.0772	8819.08789	1738.64893	94.9786
2	5.056	VB	0.0807	466.25378	86.69431	5.0214

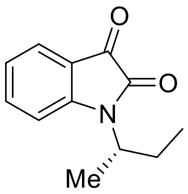

7

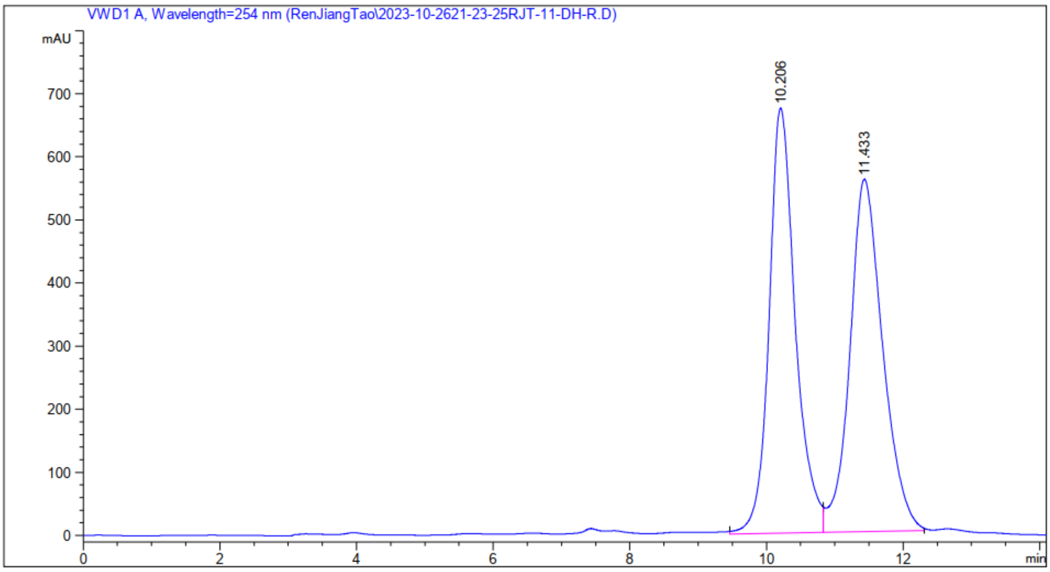

Peak #	RetTime [min]	Type	Width [min]	Area [mAU*s]	Height [mAU]	Area %
1	10.206	MF	0.4450	1.79917e4	673.88007	49.0701
2	11.433	FM	0.5575	1.86736e4	558.28516	50.9299

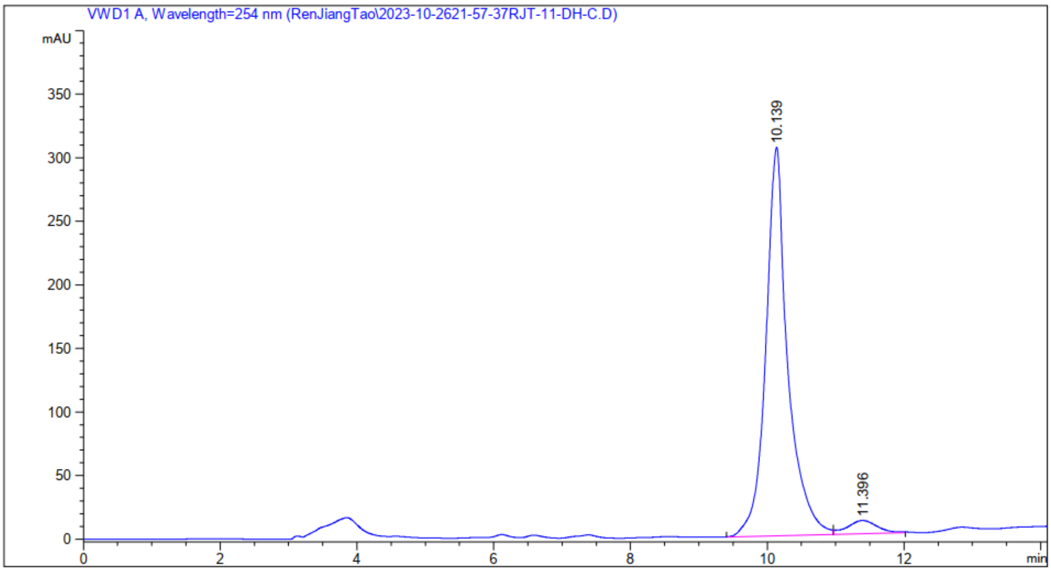

Peak #	RetTime [min]	Type	Width [min]	Area [mAU*s]	Height [mAU]	Area %
1	10.139	MF	0.3721	6820.56299	305.47711	95.6093
2	11.396	FM	0.5039	313.22144	10.35972	4.3907

12. References

1. Liao, Q., Wang, Y., Zhang, L. & Xi, C. A general copper-catalyzed coupling of azoles with vinyl bromides. *J. Org. Chem.* **74**, 6371-6373 (2009).
2. Trofimov, B. A. et al. A novel facile synthesis of 2,5-di- and 2,3,5-trisubstituted pyrroles. *Synthesis* **11**, 1585-7881 (2000).
3. Zeng, X., Cheng, G., Shen, J. & Cui, X. Palladium-catalyzed oxidative cross-coupling of *N*-tosylhydrazones with indoles: Synthesis of *N*-vinylindoles. *Org. Lett.* **15**, 3022-3025 (2013).
4. Andersen, C. et al. Introduction of cyclopropyl and cyclobutyl ring on alkyl iodides through cobalt-catalyzed cross-coupling. *Org. Lett.* **21**, 2285−2289 (2019).
5. Nomura, T., Yokoshima, S. & Fukuyama, T. Total synthesis of huperzine R. *Org. Lett.* **20**, 119–121 (2018).
6. Szeja, W. et al. Synthesis and cytotoxicity of 2,3-enopyranosyl C-linked conjugates of genistein. *Molecules* **19**, 7072-7093 (2014).
7. Liu, Q. et al. Transition-metal-free borylation of alkyl iodides via a radical mechanism. *Org. Lett.* **21**, 6597−6602 (2019).
8. Zhou, F., Zhu, J., Zhang, Y. & Zhu, S. NiH-catalyzed reductive relay hydroalkylation: A strategy for the remote $C(sp^3)$−H alkylation of alkenes. *Angew. Chem. Int. Ed.* **57**, 4058–4062 (2018).
9. Bera, S., Mao, R. & Hu, X. Enantioselective $C(sp^3)$–$C(sp^3)$ cross-coupling of non-activated alkyl electrophiles via nickel hydride catalysis. *Nat. Chem.* **13**, 270-277 (2021).
10. Cheung, C. W., Ren, P. & Hu, X. Mild and phosphine-free Iron-catalyzed cross-coupling of nonactivated secondary alkyl halides with alkynyl grignard reagents. *Org. Lett.* **16**, 2566–2569 (2014).
11. Speck, K. & Magauer, T. Evolution of a polyene cyclization cascade for the total synthesis of (−)-cyclosmenospongine. *Chem. – Eur. J.* **23**, 1157-1165 (2017).
12. Lellouche, J.-P., Pomerantz, Z. & Ghosh, S. Towards hybrid carbazole/pyrrole-based carboxylated monomers: chemical synthesis, characterisation and electro-

oxidation properties. *Tetrahedron Lett.* **52**, 6903-6907 (2011).

13. Pomerantz, Z. et al. The effect of ion-polymer binding on ionic diffusion in dicarbazole-based conducting polymers. *Electrochim. Acta* **52**, 6841-6847 (2007).
14. Artis, D. R., Cho, I.-S., Jaime-Figueroa, S. & Muchowski, J. M. Oxidative radical cyclization of (.omega.-iodoalkyl)indoles and pyrroles. synthesis of (-)-monomorine and three diastereomers. *J. Org. Chem.* **59**, 2456-2466 (1994).
15. McCabe, M. T. et al. EZH2 inhibition as a therapeutic strategy for lymphoma with EZH2-activating mutations. *Nature* **492**, 108−112 (2012).
16. Dou, X., Yao, W., Jiang, C. & Lu, X. Enantioselective *N*-alkylation of isatins and synthesis of chiral *N*-alkylated indoles. *Chem. Commun*. **50**, 11354-11357 (2014).
17. Settambolo, R., Guazzelli, G., Mengali, L., Mandoli, A. & Lazzaroni, R. A new class of optically active pyrrole derivatives: (3*R*)-3-(pyrrol-1-yl)alk-1-enes from d-α-aminoacids. *Tetrahedron: Asymmetry* **14**, 2491-2493 (2003).
18. Guazzelli, G., Lazzaroni, R. & Settambolo. Synthesis of (–)-indolizidine 167B based on domino hydroformylation/cyclization reactions. *Beilstein J. Org. Chem.* **4**, 2 (2008).
19. Zeeli, S. et al. Synthesis and biological evaluation of derivatives of indoline as highly potent antioxidant and anti-inflammatory agents. *J. Med. Chem.* **61**, 4004-4019 (2018).
20. Gao, S., Zheng, J., Ge, G. & Luo, J. Cu–catalyzed tandem oxidation of *N*-substituted indolines to ssatins. *ChemistrySelect* **3**, 13178-13181 (2018).
21. Frisch, M. J., Trucks, G. W., Schlegel, H. B., Scuseria, G. E., Robb, M. A., Cheeseman, J. R., Scalmani, G., Barone, V., Petersson, G. A., Nakatsuji, H., Li, X., Caricato, M., Marenich, A. V., Bloino, J., Janesko, B. G., Gomperts, R., Mennucci, B., Hratchian, H. P., Ortiz, J. V., Izmaylov, A. F., Sonnenberg, J. L.; Williams, Ding, F., Lipparini, F., Egidi, F., Goings, J., Peng, B., Petrone, A., Henderson, T., Ranasinghe, D., Zakrzewski, V. G., Gao, J., Rega, N., Zheng, G., Liang, W., Hada, M., Ehara, M., Toyota, K., Fukuda, R., Hasegawa, J., Ishida, M., Nakajima, T. Honda, Y., Kitao, O., Nakai, H., Vreven, T., Throssell, K., Montgomery Jr., J. A., Peralta, J. E., Ogliaro, F., Bearpark, M. J., Heyd, J. J., Brothers, E. N., Kudin, K. N., Staroverov, V. N., Keith, T. A., Kobayashi, R., Normand, J., Raghavachari, K., Rendell, A. P., Burant, J. C., Iyengar, S. S., Tomasi, J., Cossi, M., Millam, J. M., Klene, M., Adamo, C., Cammi, R., Ochterski, J. W., Martin, R. L., Morokuma, K.; Farkas, O., Foresman, J. B., Fox, D. J. *Gaussian 16 Rev. A.03*, Wallingford, CT,

(2016).
22. Becke, A. D. Density-functional thermochemistry. III. The role of exact exchange. *J. Chem. Phys.* **98**, 5648-5652 (1993).
23. Grimme, S., Antony, J., Ehrlich, S. & Krieg, H. A consistent and accurate ab initio parametrization of density functional dispersion correction (DFT-D) for the 94 elements H-Pu. *J. Chem. Phys.* **132**, 154104 (2010).
24. Weigend, F. & Ahlrichs, R. Balanced basis sets of split valence, triple zeta valence and quadruple zeta valence quality for H to Rn: Design and assessment of accuracy. *Phys. Chem. Chem. Phys.* **7**, 3297-3305 (2005).
25. Fukui, K. The path of chemical reactions-the IRC approach. *Acc. Chem. Res.* **14**, 363-368 (1981).
26. Staroverov, V. N., Scuseria, G. E., Tao, J. & Perdew, J. P. Comparative assessment of a new nonempirical density functional: Molecules and hydrogen-bonded complexes. *J. Chem. Phys.* **119**, 12129-12137 (2003).
27. Tao, J., Perdew, J. P., Staroverov, V. N. & Scuseria, G. E. Climbing the density functional ladder: nonempirical meta-generalized gradient approximation designed for molecules and solids. *Phys. Rev. Lett.* **91**, 146401 (2003).
28. Amabilino, S. & Deeth, R. J. DFT analysis of spin crossover in Mn(III) complexes: is a two-electron S = 2 to S = 0 spin transition feasible? *Inorg. Chem.* **56**, 2602-2613 (2017).
29. Jensen, K. P. Bioinorganic chemistry modeled with the TPSSh density functional. *Inorg. Chem.* **47**, 10357-10365 (2008).
30. Kepp, K. P. Theoretical study of spin crossover in 30 Iron complexes. *Inorg. Chem.* **55**, 2717-2727. (2016).
31. Reimann, M., Bischoff, F. A. & Sauer, J. Thermochemistry of $FeO_mH_n^z$ species: Assessment of some DFT functionals. *J. Chem. Theory Comput.* **16**, 2430-2435 (2020).
32. Tomasi, J., Mennucci, B. & Cammi, R. Quantum mechanical continuum solvation models. *Chem. Rev.* **105**, 2999-3094 (2005).
